# Global, regional, and national burden of esophageal cancer: a systematic analysis of the Global Burden of Disease Study 2021

**DOI:** 10.1186/s40364-024-00718-2

**Published:** 2025-01-06

**Authors:** Weiqiu Jin, Kaichen Huang, Ziyin Ding, Mengwei Zhang, Chongwu Li, Zheng Yuan, Ke Ma, Xiaodan Ye

**Affiliations:** 1 Department of Radiology, Zhongshan Hospital, Fudan University, Shanghai, 200032 China; 2 Shanghai Institute of Medical Imaging, Shanghai, 200032 China; 3 Department of Cancer Center, Zhongshan Hospital, Fudan University, Shanghai, 200032 China; 4 Department of Thoracic Surgery, Sichuan Clinical Research Center for Cancer, Sichuan Cancer Hospital & Institute, Sichuan Cancer Center, Affiliated Cancer Hospital of University of Electronic Science and Technology of China, Chengdu, 610041 China; 5 Department of Cardiovascular Surgery, Ruijin Hospital, Shanghai Jiao Tong University School of Medicine, Shanghai, 200025 China; 6 Center for Reproductive Medicine, Women and Children’s Hospital of Ningbo University, Ningbo, 315012 China; 7 Department of Liver Surgery, Renji Hospital, Shanghai Jiao Tong University School of Medicine, Shanghai, 200127 China; 8 Department of Cardiothoracic Surgery, The First Affiliated Hospital of Chongqing Medical University, Chongqing, 400016 China; 9 Department of Radiology, Shanghai Ninth People’s Hospital, Shanghai Jiao Tong University School of Medicine, Shanghai, 200011 China

**Keywords:** Esophageal cancer, Global Burden of Disease, Disability-adjusted life years, Incidence

## Abstract

**Background and objective:**

Esophageal cancer (EC) is the seventh most prevalent cancer globally and the sixth leading cause of cancer-related mortality. This study aimed to provide an updated stratified assessment of rates in EC incidence, mortality, and disability-adjusted life-years (DALYs) from 1990 to 2021 by sex, age, and Socio-demographic Index (SDI) at global, regional, and national levels, as well as to project the future trends of EC both globally and regionally.

**Methods:**

Data about age-standardized rates (ASRs) of incidence (ASIR), mortality (ASDR), probability of death (ASPoD) and DALYs (ASDALYRs) of EC were obtained from the 2021 Global Burden of Disease (GBD) study. Estimated annual percentage changes (EAPCs) and average annual percentage changes (AAPC) were calculated over certain periods to describe the temporal trends of EC burdens. The analyses were disaggregated by sexes, GBD super-regions and regions, nations/territories, age-groups, and SDI quintiles. A Bayesian age-period-cohort (BAPC) model was constructed to project the global and regional EC ASRs in 2022–2035.

**Results:**

Despite global reductions in EC ASRs, with ASIR, ASDR, and ASDALYR in 2021 of 6.65 [5.88, 7.45] (95% uncertainty interval), 6.25 [5.53, 7.00], and 148.56 [131.71, 166.82], decreasing by 24.9%, 30.7%, and 36.9% in 1990–2021, respectively, the absolute burden numbers were increased from 1990 to 2021, probably because of population growth and aging. Global newly diagnosed cases, deaths, and DALYs of EC increased to 576,529 [509,492, 645,648], 356,263 [319,363, 390,154], and 12,999,265 [11,522,861, 14,605,268] in 2021, by 62.53%, 51.18%, and 33.28% compared to records in 1990. The geographical pattern of EC was consistent: locations with the highest EC incidence and mortality rates were predominantly located in the Asian Esophageal Cancer Belt and African Esophageal Cancer Corridor, with East Asia, Southern Sub-Saharan Africa, and Eastern Sub-Saharan Africa as the GBD regions with the heaviest EC burdens, and Malawi, Eswatini, Mongolia, Zambia, and Zimbabwe with the most EC ASRs in 2021. However, owing to the population size, China, India, the United States, Japan, and Brazil had the heaviest absolute EC burdens. More pronounced alleviations of ASRs were observed in locations with high SDI levels, indicated by their lower AAPC values compared to those of low-SDI locations, while Sub-Saharan Africa regions had increasing EC ASRs, especially in Chad (114.76% in ASDR, for example), Sao Tome and Principe (97.93%), Togo (92.53%), Northern Mariana Islands (84.32%), Liberia (82.33%), etc. Smoking remained the leading contributor to EC ASDALYR globally and across most GBD super-regions in 2021. The EC burden is significantly heavier for males, with incidence and mortality in males in 2021 being 2.89 and 2.88 times higher, respectively, than in females. Across all age groups, EC posed an increasingly significant threat to men aged > 75 years. From 2022 to 2035, the ASR projections show only modest decrease in both global and regional EC burdens, and the absolute burden numbers are expected to increase globally and in nearly all GBD super-regions.

**Conclusion:**

EC burden remains significant, with disparities across sexes, age groups, and regions. Region-specific and age-targeted measures are crucial to addressing these inequalities, especially in light of increasing EC burdens in older men and in African regions. Efforts should be taken in finding more solid attributions to risk factors for EC burdens and to better identify high-risk populations to inform targeted prevention and screening, and ultimately reduce the EC burden in an efficient and cost-effective way.

**Graphical Abstract:**

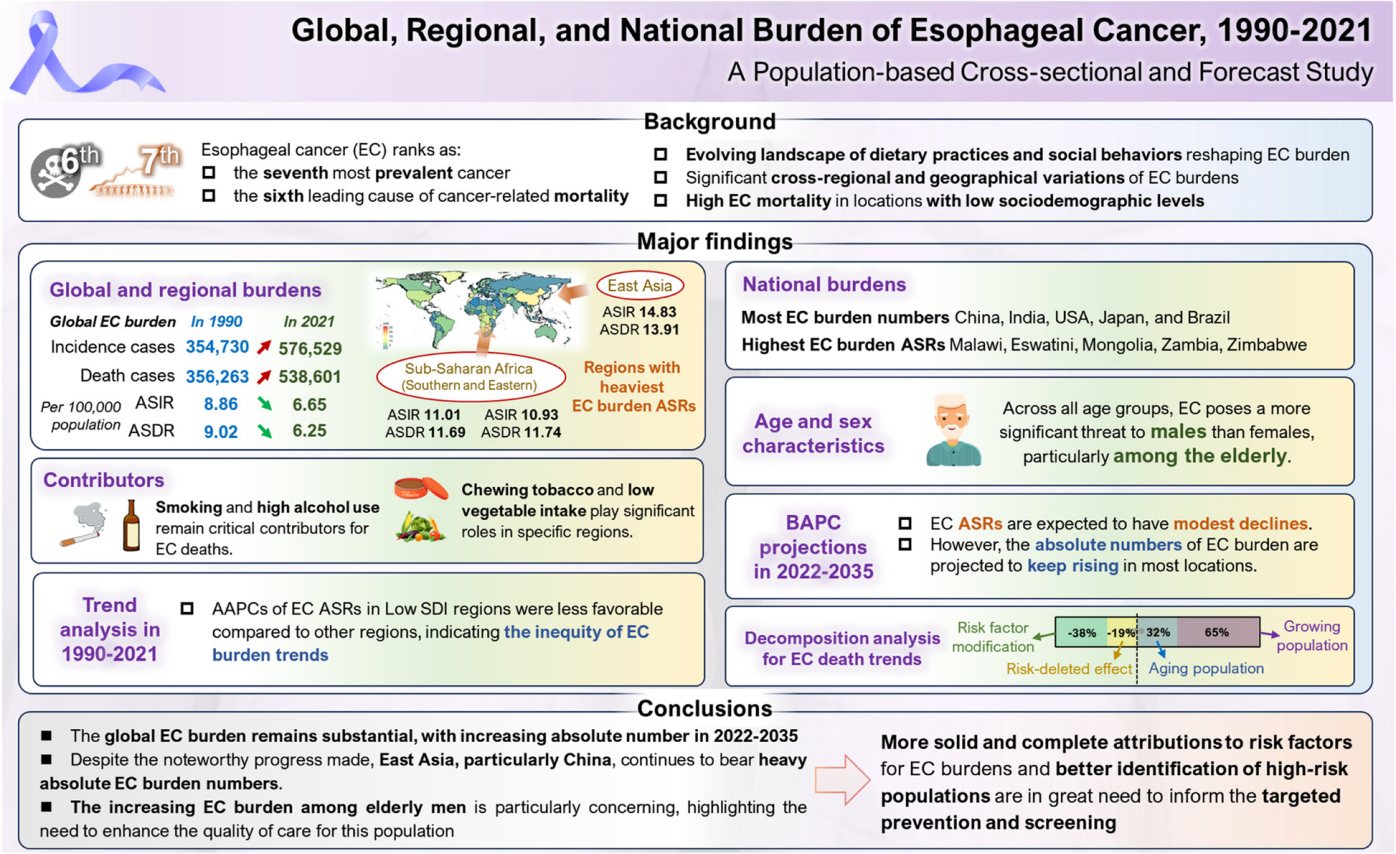

**Supplementary Information:**

The online version contains supplementary material available at 10.1186/s40364-024-00718-2.

## Introduction

Esophageal cancer (EC) ranks as the seventh most prevalent cancer globally and is the sixth leading cause of cancer-related mortality [1]. Although the global pattern of EC burden has been being reshaped and improved by the evolving landscape of dietary practices and social behaviors both globally and regionally, significant cross-national and cross-regional variations of EC burdens and high EC mortality in locations with low socioeconomic levels still pose a huge challenge to global health [1, 2].

In recent years, some intriguing factors have been driving new trends in the EC epidemiology. First, the epidemiological distribution of the two EC subtypes, esophageal squamous cell carcinoma (ESCC) and esophageal adenocarcinoma (EAC), is shifting, a shift characterized by surging EAC and declining ESCC incidence in Western countries, possibly due to the rising incidence of central obesity, which promotes gastroesophageal reflux, and the reduced prevalence of alcohol consumption and tobacco use [1, 3]. Second, the aging of the global population [4] and lifestyle changes [3, 5, 6] are bringing growing uncertainty to the epidemiological characteristics. Third, recently, significant advancements have been made in EC screening and treatment. The use of chemotherapy, targeted therapies, immune checkpoint inhibitors (ICIs), liquid biopsy, and population-based endoscopic screening in EC trials highlights the progress in detecting EC early, conducting multi-modal treatment, and improving patient outcomes [7–10]. Fourth, it appears that the diagnosis and treatment of many cancers were affected by the coronavirus disease 2019 (COVID-19) pandemic [11–15]. The COVID-19 pandemic exacerbated healthcare workforce shortages, as its high transmissibility and widespread prevalence prompted many governments to implement lockdowns or quarantine measures to minimize social contact. These policies likely contributed to delays, inaccuracies, and missed diagnoses in some cancer patients. For instance, a population-based analysis utilizing Surveillance, Epidemiology, and End Results (SEER) data through 2020 reported an 8.7% decline in cancer incidence during the first year of the pandemic [12]. As well as COVID-19 affecting diagnostic timeliness and precision, several studies have demonstrated that COVID-positive patients with cancer face significantly higher mortality rates compared to non-cancer patients, with relative risks (RR) ranging from 1.4 to 2.3 [16–18]. Moreover, lockdowns and quarantine measures may have further restricted access of patients with cancer to healthcare, delaying essential screening, diagnosis, and treatment. A meta-analysis highlighted these challenges, showing that COVID-19 and associated restrictions led to notable declines in cancer screenings and treatment delays [19]. The above four points warrant further exploration and discussion, and the Global Burden of Disease (GBD) study provides an invaluable opportunity for such investigation.

The GBD study offers detailed data on a wide range of diseases for 204 countries and territories in 21 GBD regions and seven GBD super regions worldwide [20, 21]. Previous GBD analyses based on data from the 2017/2019 GBD study have underscored the significant geographic and gender diversity of the global EC burden. However, these analyses were based on data from GBD 2017/2019, that is, pre-COVID-19, and future projections on global and regional levels with updated data are absent from existing literature [2, 6, 22, 23], which limits the investigations on the evolution of global EC epidemiology and the effect of COVID-19 on global EC burden metrics. Indeed, given the profound and rapid transitions in EC burden driven by the aforementioned factors, it is imperative to utilize up-to-date data to refine and update our systematic understanding of the global, regional, and national burdens of EC.

The 2021 GBD study, marking the first post-COVID-19-pandemic GBD update, offers a crucial framework for analyzing disease burden metrics, including incidence, mortality, and disability-adjusted life years (DALYs), and for evaluating the pandemic’s influence on the global burden of specific diseases [20, 21]. Notably, GBD 2021 introduced the estimates of the probability of death (PoD) for specific diseases, accounting for the effects of screening and treatment across various age cohorts. To gain a deeper understanding of the EC burden across different geographic regions, social development indexes (SDIs), age groups, and sexes, as well as the shifts in EC incidence and mortality due to the pandemic, we performed detailed subgroup analyses using the updated GBD 2021 data. Additionally, we utilized a Bayesian age-period-cohort (BAPC) model to forecast and examine future epidemiological trends. These analyses and projections provide vital insights for decision-making and strategic planning in EC prevention and treatment, offering significant benefits to researchers, clinicians, and public health administrators.

## Data and methods

### Data sources and software

This study utilized and analyzed data from the GBD 2021 results, which are publicly accessible via the Global Health Data Exchange 2021 (GHDx 2021, https://vizhub.healthdata.org/gbd-results/, accessed July 1, 2024) [20, 21, 24]. GBD 2021 assessed the burden of 288 causes, 371 diseases and injuries, and 88 risk factors across 204 countries and territories over the period 1990 to 2021. Data on the incidence, mortality, DALYs, and PoD due to EC, disaggregated by sex, age, location, and risk factors, were extracted from the GHDx 2021 [20, 21, 24]. Further details regarding GBD 2021 can be found in the **eNote**. Descriptive analyses and visualizations were conducted using MATLAB R2024a and OriginPro 9.9.5.

### Age-standardized burden metrics

The age-standardized rates of incidence (ASIRs), death (ASDRs), and DALYs (ASDALYRs) in GBD 2021 were defined in a similar way to previous GBD studies and calculated using the GBD standard-population structure **(**definitions in eTable S1a; the standard population structure shown in eNote S1-4 [25]) [24]. GBD 2021 defined PoD as the probability that a person dies during an interval of two ages (e.g., between birth and age five), if the rates of all-cause mortality in a specified year of interest would remain constant into the future [20, 21, 24]. ASIRs, ASDRs, ASDALYs, and age-standardized PoDs (ASPoDs) were presented per 100,000 people in different locations **(**detailed in eTable S1a). The DALYs were defined as sums of years lived with disability (YLDs) and years of life lost (YLLs).

### Models for burden estimation in GBD 2021

The EC burden metrics, including mortality/death, incidence, YLLs, YLDs, DALYs, prevalences and PoD, were estimated by GBD 2021 collaborators using updated registry data from 35 countries/territories combined with data from GBD 2019 [20, 21, 24].

The GBD Cause of Death (CoD) database, generated through the GBD mortality estimation process, integrates cancer mortality data from diverse sources such as vital registration, verbal autopsy, and cancer registry data [20, 21, 24]. In particular, cancer registry incidence data are converted to mortality estimates using mortality-to-incidence ratios (MIRs), which inform the cancer registry mortality estimates subsequently incorporated into the CoD database. The registry data undergo a series of rigorous processing steps prior to inclusion in the CoD database: (1) formatting incidence and mortality data, (2) recalculating subtotals, (3) mapping data to GBD causes, (4) age and sex stratification, (5) disaggregation of causes, (6) redistribution for unspecified International Classification of Diseases (ICD) codes, (7) elimination of duplicates, (8) matching of processed incidence and mortality data by cancer type, age, sex, year, and location to develop MIRs using a three-step modeling approach employing the general GBD spatiotemporal Gaussian process regression (ST-GPR) method, where logit-transformed MIR serves as the outcome with sex, categorical age group, and Healthcare Access and Quality (HAQ) Index as covariates in the linear mixed-effects model, and (9) deriving mortality estimates from incidence and MIRs [20, 21, 24]. A CoD Ensemble model (CODEm) methodology, which consolidates data from the aforementioned sources, was employed to estimate mortality across various sub-models [20, 21, 24]. For EC, mortality estimates across locations, years, and age groups were generated using sex-specific CODEm models, and YLLs were calculated utilizing a standard age-specific GBD life expectancy applied to mortality estimates by age group [20, 21, 24].

Regarding incidence estimation for EC, the final GBD cancer mortality estimates were converted to incidence estimates by applying the MIRs pertinent to each cancer cause [20, 21, 24]. For DALY estimation, prevalence estimates combined with disability weights corresponding to different stages of cancer survival were utilized [20, 21, 24]. DALYs were calculated as the aggregate of YLDs and YLLs. In terms of prevalence, GBD 2021 synthesized information on incidence and anticipated absolute survival to formulate estimates for varying ages, sexes, calendar years, and locations [20, 21, 24]. Specifically, cancer-specific prediction models were constructed to estimate five-year survival from MIRs using SEER data, providing estimations of five-year survival across different demographic and temporal categories based on GBD MIR estimates [20, 21, 24]. Subsequently, GBD 2021 computed a proportional scalar by dividing the projected GBD five-year survival estimate by the SEER five-year survival statistic [20, 21, 24]. Annual survival estimates were then derived by aligning the one- to ten-year SEER curve with the GBD survival projections under the assumption of proportional hazards [20, 21, 24]. For PoDs, they were defined as the likelihood of death from a specific cause within a designated age interval, conditional upon survival at the start of that interval, whereby the comprehensive findings from GBD 2021 were used to evaluate the status of a cause of death within a particular population [20, 21, 24].

### SDI values

The socio-demographic characteristics of regions were encapsulated by SDI values, providing a comprehensive metric that delineates the developmental spectrum of geographical areas. The SDI, ranging from 0 to 1, synthesizes rankings based on per capita income, average educational attainment, and fertility rates across all locations included in the GBD study (eTable S3) [20, 21, 24].

### Selection of regions

Basically, in GBD 2021, the world was segmented into seven GBD super-regions and 21 GBD regions, primarily determined by geographic position and associated demographic factors **(**eTable S2) [20, 21, 24]. To describe the disease burden indicators of locations in a stratified way according to their levels of development, in GBD 2021, regions were also categorized into SDI quintiles according to their SDI values in 2021, classified as low (0 < SDI ≤ 0.570), low-middle (0.570 < SDI ≤ 0.670), middle (0.670 < SDI ≤ 0.812), high-middle (0.812 < SDI ≤ 0.858), and high (0.858 < SDI ≤ 1) (eTable S3, S4) [20, 21, 24].

### Risk factor hierarchy, selection, and modelling

The selection of risk factors was conducted in accordance with the risk factor hierarchy provided by GBD 2021 [24]. Like GBD 2019, GBD 2021 included risk–outcome pairs that met the World Cancer Research Fund (WCRF) grades of convincing or probable evidence, where biologically plausible associations between exposure and disease established from multiple epidemiological studies that were mostly prospective observational studies and (when relevant) randomized controlled trials (RCTs) having sufficient size, duration, and quality that showed consistent effects in different populations were considered [24]. New to GBD 2021, a Burden of Proof (BoP) methodology was applied to include more risk-outcome pairs according to the outputs of BoP functions evaluating the strengths and reliabilities of associations [24], as well as the review and majority vote for inclusion of emerging risk-outcome pairs by the GBD Scientific Council [24].

There are four risk factors involved in this study: smoking, diet low in vegetables, chewing tobacco, and high alcohol use. These factors were retained from GBD 2019 without new pairs included (eNote S1-3) [24]. Smoking was defined as current and former smoking of any tobacco product, where continuous smoking exposure level (pack-years) was considered [24]. A diet low in vegetables was defined as inadequate average daily consumption (in grams per day) of vegetables, including fresh, frozen, cooked, canned, or dried vegetables and excluding legumes and salted or pickled vegetables, juices, nuts and seeds, and starchy vegetables such as potatoes or corn [24]. Current chewing tobacco use, including local products, such as betel quid with tobacco, was defined as current use (use within the last 30 days where possible, or according to the closest definition available from the survey) of any frequency (any, daily, or less than daily) [24]. Updated in GBD 2021, alcohol use, which previously referred to all levels of alcohol consumption, was now specifically defined as high alcohol use, namely consumption of alcohol in excess of the theoretical minimum risk exposure level (TMREL), the level of alcohol consumption at which all-cause risk is minimized [24].

GBD produced estimates of EC burden that is attributable to each risk factor by considering both risk factor exposure levels (prevalence of the risk factor for each age, sex, country, and year) and population attributable fraction (PAF) (the proportion of burden of a disease or condition that is caused by a specific risk factor) [24]. There were two models, ST-GPR and MR-BRT, applied for risk factor exposure level estimation and dose-response RR curves, respectively. The MR-BRT modeling took all data about RRs and synthesized them, which produced a curve describing for an individual with a given level of exposure to a risk factor, what their risk of death is from a particular disease compared to an individual who is not exposed to the risk factor. Meanwhile, ST-GPR uses predictive covariates to inform trends, which allows neighboring countries to be considered epidemiologically similar to each other with an assumption that exposure to risk factors evolves smoothly over time [24].

 The core of the attributable risk factor analysis is the RR estimate that calculates how much more likely individuals are to get a given disease, namely EC, because of a particular risk factor (the above-mentioned four factors) [24]. Generally, GBD 2021 follows a process of crosswalk, age and sex splitting, prevalence modeling, determination of TMREL, evaluation of RR, calculation of PAF, and combination of RR and PAF [24]. For example, the smoking-EC pair is evaluated by continuous smoking exposure level (pack-years), which incorporated aspects of both duration and amount. For the RR of EC from smoking, studies were included if they reported a categorical or continuous dose for smoked tobacco consumption (pack-years) as well as uncertainty measures of the estimated risk, and the population under study was the general population [24]. These studies were comprehensively reviewed and integrated to produce the RR curves, where the TMREL was 0. Using a sex-geography-time-specific reference age pattern, data reported in broader age groups than the GBD five-year age groups or as both sexes combined were split [24]. Then, ST-GPR was applied to model the current and former smoking prevalence. The PAF were subsequently estimated with the prevalence estimates and RRs [24]. The definition of PAF in attributable risk factors analysis determined that some proportion of burdens remained without attribution; this was considered as a part of risk-deleted effect in the decomposition analysis, which will be introduced in the following Sect. [24].

### Calculation of annual percentage changes (APCs)

Three kinds of APCs were calculated in this study, as appropriate. Their strengths, weaknesses, and applied scenarios in this study are detailed in eTable S1(b).

The estimated annual percentage change (EAPC) was calculated by assuming that the natural logarithm values of death, incidence, and DALY rates, namely the age-standardized rates (ASRs), vary linearly with time:

$$Y=\alpha+\beta X+\varepsilon$$

where *Y* = ln(*ASRs*), *X* = calendar years, ε = error terms, $\:\alpha\:$, $\:\beta\:$ denote the intercept and slope, respectively. With the slope values we have:

$$EAPC=100\times\left(\mathrm e^{\mathrm\beta}-1\right).$$

Furthermore, 95% confidence intervals (CIs) were calculated using the linear model. The trends of incidence, death, DALY, and PoD were reflected in the EAPC values. Positive EAPCs corresponded to an upward trend, while the downtrend of metrics was reflected in negative EAPCs.

In some cases, EAPC analysis alone may ignore the segmented changes in ASRs, namely the temporal trends in a certain time period, since it assumes that the changing trend of a metric during the time range is linear. Therefore, we chose segmented (period) epidemiologic slope analysis, when appropriate, to avoid this potential issue. In this context, we calculated the segmented annual percentage change (SAPC), which was defined as:

where Year_2_ and ASR_2_ are the latter calendar year and its corresponding ASR value, and Year_1_ and ASR_1_ are the previous calendar year and its corresponding ASR value. However, the SAPC only considers the values of start and end points, which fails to consider the changes of metrics during the segmented range of years.

$$SAPC=\frac{ASR_2-ASR_1}{\left({\mathrm{Year}}_2-{\mathrm{Year}}_1\right)\cdot ASR_1}$$

The Joinpoint regression model, also known as piecewise or multi-phase regression, divides a long-term trend line into several linear segments. It does not require a predefined trend in the data, making it particularly effective for analyzing long-term disease data with multiple trends. The model’s strength lies in its ability to compute the Average Annual Percent Change (AAPC), a summary measure over a specified interval. When defining *b*_*i*_ as the slope coefficient for the *i*^th^ segment with *i* indexing the segments in the pre-defined range of years, and *w*_*i*_ as the length of each segment in the range of years, we have:

$$AAPC=\exp\;\left(\frac{\sum biWi}{\sum Wi}\right)\times100$$

which represents a weighted average of the APCs derived from the Joinpoint model, with weights proportional to the duration of each APC interval.

### Bayesian age-period-cohort (BAPC) model

The age-period-cohort (APC) models are crucial in capturing the dynamic aspects and components in epidemiological studies. As a method for examining disease registry data, the APC model identifies the influences of age, period, and cohort on time-dependent phenomena. This model differentiates between changes due to varying rates (risk) and those resulting from changes in population structure. We determined the age standardization rate utilizing the average global population structure estimated by GBD researchers in prior studies [26]. For the BAPC model with integrated nested Laplace approximations (INLA), the BAPC (version 0.0.36) package was employed as a wrapper for the INLA (version 22.05.07) package specifically designed for APC analysis in R software. Essentially, Bayesian inference treats uncertain parameters as random, with specific prior distributions. It is assumed that time-adjacent effects are similar. Hence, a second-order random walk (RW2) is typically used to provide smoothing priors for age, period, and cohort effects, assuming that the second differences of all time effects follow independent mean-zero normal distributions. INLA was incorporated into the BAPC for comprehensive Bayesian inference. The principles of INLA can be referenced in previous literature [27, 28]. The strengths of the BAPC model over other models have been demonstrated in previous studies [29, 30].

### Reporting standards

All rates are reported per 100,000 person-years. For all estimates, 95% uncertainty intervals (UIs) are reported. Uncertainty was propagated through each step of the EC burden estimation process, with UIs representing the 2.5th and 97.5th percentiles of the distribution of 1000 draws at each step [20, 21, 24]. The regression results shown in the figures were generated with cubic polynomial regressions with least absolute residual (LAR) optimizations. The UIs in graph lines, text numbers, and table numbers were all 95%.

### Driver of trends in EC death changes

Temporal changes in EC deaths are decomposed into four main component drivers of change: (1) Population factor, namely the population growth; (2) Aging, namely the changes in population age structures; (3) Risk effects, namely the changes in risk factors; (4) Risk-deleted effects, namely the changes due to all other factors, approximated as the risk-deleted deaths [31]. The risk-deleted rate is the death rate that would be observed had we removed all risk factors included in GBD 2021, namely the rate that would be observed had the exposure levels for all risk factors included in GBD 2021 been reduced to the TMREL [24]. The changes in risk-deleted rates might reflect changes in risk–outcome pairs that are not included in GBD 2021, or changes in terms of improved therapeutic agents and modalities, etc [24].

The YLLs due to EC are significantly more than the YLDs. In 2021, the global number of EC-YLLs was 12,839,879 ( [11, 375, 539, 14, 412, 364]), whereas the number of YLDs was 159,386 [114, 680, 207, 787], with YLDs accounting for only 1.23% of DALYs [21]. Therefore, analyzing changes in EC mortality alone is sufficient to represent the driving factors behind changes in the outcome burden of EC (i.e., non-incidence burden) over the past several years. With methods developed by Das Gupta [32, 33], we performed a decomposition analysis for the 32-year period of 1990–2021, and reported the overall differences of deaths related to the above-mentioned four drivers.

### Ethical considerations

This study collected and re-analyzed the data from GBD 2021, which was approved by the institutional review board of the University of Washington School of Medicine [21, 24]. As this study was a secondary analysis, no additional human participant research ethics review or informed consent was required.

### Role of the funding source

The funders had no role in the study design, the collection, analysis and interpretation of data, the writing of the report, or the decision to submit the article for publication.

## Results

### ASRs and absolute numbers of global EC burdens

**Fig. 1:**
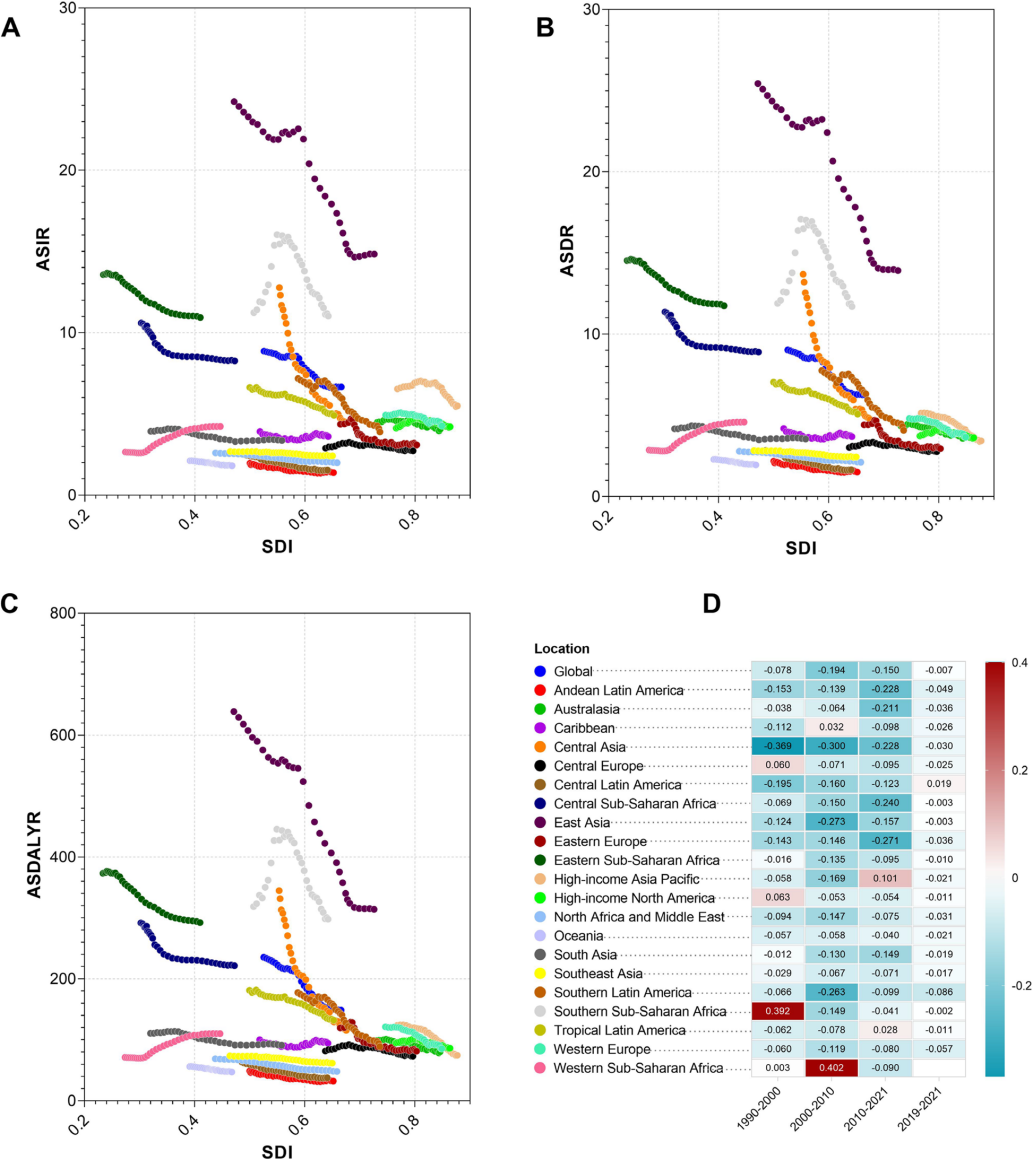
The ASRs (per 100,000 population) of esophageal cancer incidence (**A**), death (**B**), and DALY (**C**) in 2021 worldwide. The segmented annual percentage changes (SAPC) of ASDALY rate of esophageal cancer worldwide and across 21 GBD regions (**D**). Please note that the SAPC value of Western Sub-Saharan Africa from 2019 to 2021 is less than 0.001 and is therefore not labelled in the figure

In 2021, the global incidence of EC cases increased to 576,529 [509, 492, 645, 648], with 428,387 [367, 888, 495, 196] in males and 148,142 [113, 641, 172, 538] in females, from 354,731 [317, 512, 388, 914] in 1990. From 1990 to 2021, the death cases from EC increased from 356,263 [319, 363, 390, 15] to 538,602 [475, 944, 603, 406] (males: 399,796 [343, 473, 459,871], females: 138,806 [107,414, 161,288]). DALY numbers increased from 9,753,566.25 [8,719,319.34, 10,739,560.72] in 1990 to 12,999,264.90 [11,522,861.15, 14,605,268.10] in 2021 (males: 9,889,700.56 [8,502,607.09, 11,434,422.27], females: 3,109,564.35 [2,478,470.60, 3,575,201.61]). However, as for the global age-standardized burdens, the ASIR, ASDR, and ASDALYR in 2021 were 6.65 [5.88, 7.45], 6.25 [5.53, 7.00], and 148.56 [131.71, 166.82], respectively, with a decrease during 1990–2021 by 24.9%, 30.7%, and 36.9%, respectively (Fig. 1; Table 1).

**Table 1:** The absolute burden numbers (incidence, death, and DALY numbers), and their male/female ratios for esophageal cancer at global level, in SDI quintiles, WHO regions and GBD regions in 2021, and their changing trends in 1990–2021

Locations	Incidence number (×10^3^)				Death number (×10^3^)				DALY number (×10^3^)			
	Number		M/F		Number		M/F		Number		M/F	
	Value in 2021	PC	Value in 2021	PC	Value in 2021	PC	Value in 2021	PC	Value in 2021	1990–2021 PC	Value in 2021	PC
**Global**	576.5 [509.5, 645.6]	62.5%	1.27	3.5%	538.6 [475.9, 603.4]	51.2%	1.27	3.8%	12999.3 [11522.9, 14605.3]	33.3%	1.27	2.9%
**SDI quintiles**												
High SDI	102.5 [95.2, 107.3]	74.9%	1.13	5.3%	85.7 [79.2, 89.9]	57.9%	1.14	6.1%	1825.5 [1719.0, 1902.7]	37.7%	1.11	4.9%
High-middle SDI	176.8 [145.1, 214.1]	57.5%	1.48	15.5%	162.4 [134.3, 195.5]	42.1%	1.46	14.6%	3834.3 [3157.5, 4667.6]	23.0%	1.48	15.1%
Middle SDI	217 [182.2, 258.4]	51.6%	1.42	3.3%	207.6 [174.9, 246.5]	42.6%	1.41	3.6%	5011.8 [4233.9, 5964.3]	22.7%	1.41	2.4%
Low-middle SDI	52.1 [47.2, 59.9]	105.7%	1.27	0.2%	53.7 [48.5, 61.8]	105.5%	1.27	0.2%	1491.6 [1348.1, 1724.1]	95.5%	1.28	0.7%
Low SDI	28 [23.8, 32.2]	82.7%	1.35	−0.3%	28.9 [24.6, 33.4]	82.7%	1.36	0.4%	830.1 [701.3, 964.0]	80.4%	1.37	1.0%
**WHO regions**												
African Region	36.8 [31.1, 42.7]	109.4%	1.37	3.3%	38 [32.2, 44.0]	109.2%	1.37	3.5%	1080.1 [915.3, 1264.3]	105.9%	1.38	2.4%
Eastern Mediterranean Region	15.6 [13.3, 17.8]	111.1%	1.34	1.6%	16 [13.6, 18.3]	108.0%	1.34	1.9%	446.7 [378.3, 516.7]	107.3%	1.37	3.2%
European Region	59.5 [55.9, 62]	17.2%	1.11	5.9%	55.4 [51.8, 57.8]	8.6%	1.12	6.2%	1257.6 [1194.7, 1305.3]	−3.0%	1.09	5.2%
Region of the Americas	49.8 [47.1, 51.8]	87.1%	1.10	3.1%	47.3 [44.6, 49.3]	81.4%	1.10	3.4%	1120.9 [1073.9, 1159.6]	70.6%	1.08	3.2%
South-East Asia Region	56.8 [50.8, 66.9]	113.3%	1.32	−0.9%	57.7 [51.5, 68.2]	112.2%	1.33	−0.6%	1592.5 [1417.9, 1869.6]	94.4%	1.32	−0.8%
Western Pacific Region	353.8 [287.3, 426.7]	58.0%	1.49	8.8%	320.6 [260.4, 386.7]	43.0%	1.48	9.0%	7403.5 [5963.3, 9057.7]	19.6%	1.52	9.9%
**GBD regions**												
Andean Latin America	0.8 [0.7, 1]	107.9%	1.50	16.3%	0.9 [0.7, 1.1]	107.0%	1.49	15.7%	19.2 [15.5, 23.6]	87.2%	1.52	17.6%
Australasia	2.2 [2, 2.4]	108.8%	1.19	6.9%	2 [1.8, 2.2]	100.8%	1.19	6.6%	41 [37.6, 43.8]	78.0%	1.16	5.1%
Caribbean	2 [1.7, 2.2]	94.9%	1.29	14.4%	2 [1.8, 2.3]	88.9%	1.28	14.3%	51 [44.5, 58.2]	94.5%	1.31	14.3%
Central Asia	3.6 [3.2, 4]	−40.0%	1.25	13.6%	3.7 [3.3, 4.2]	−40.5%	1.24	13.3%	99.9 [88.5, 112.3]	−40.3%	1.27	16.2%
Central Europe	5.8 [5.3, 6.2]	33.3%	1.18	8.8%	5.9 [5.4, 6.4]	32.2%	1.18	8.8%	146.5 [134.8, 158.4]	19.6%	1.17	8.3%
Central Latin America	3.8 [3.4, 4.3]	87.2%	1.26	18.9%	4 [3.6, 4.5]	84.8%	1.26	18.5%	95.8 [85.5, 107.6]	75.4%	1.26	20.2%
Central Sub-Saharan Africa	4.5 [3.3, 5.9]	88.8%	1.77	4.0%	4.7 [3.4, 6.1]	88.3%	1.79	4.3%	137.6 [100.0, 180.2]	88.0%	1.80	2.7%
East Asia	327.7 [263.6, 401.9]	55.5%	1.52	9.4%	302.6 [243.4, 368.7]	41.4%	1.52	9.5%	7069.8 [5660.3, 8736.1]	19.0%	1.54	10.0%
Eastern Europe	10.7 [9.7, 11.6]	−13.6%	1.20	14.8%	10.3 [9.4, 11.2]	−17.4%	1.19	14.1%	274.1 [247.2, 298.3]	−19.6%	1.21	16.0%
Eastern Sub-Saharan Africa	18.4 [15.3, 22.1]	80.7%	1.44	2.5%	19 [15.9, 22.9]	80.6%	1.44	3.1%	545.4 [452.3, 659.2]	78.9%	1.46	2.6%
High-income Asia Pacific	25.5 [22.8, 27.1]	92.4%	1.19	8.7%	16.9 [15, 18.0]	64.2%	1.20	8.8%	318.6 [290.6, 336.6]	24.6%	1.16	5.8%
High-income North America	27.3 [25.6, 28.4]	93.6%	1.11	3.0%	24 [22.4, 25.0]	85.4%	1.11	3.1%	535.3 [510.5, 552.8]	71.3%	1.08	2.4%
North Africa and Middle East	8.7 [7.4, 9.8]	101.6%	1.33	−7.8%	8.8 [7.5, 10.0]	98.1%	1.33	−7.3%	230.2 [192.0, 263.0]	84.3%	1.37	−7.1%
Oceania	0.1 [0.1, 0.2]	118.7%	1.60	−9.2%	0.1 [0.1, 0.2]	118.3%	1.61	−6.7%	3.9 [3.1, 5.0]	114.0%	1.64	−8.0%
South Asia	50.1 [44.2, 59.9]	115.7%	1.35	−0.6%	51.5 [45.7, 61.7]	116.3%	1.35	−1.4%	1434.8 [1268.8, 1704.6]	100.7%	1.34	−1.7%
Southeast Asia	16.2 [14, 18.6]	132.4%	1.33	−4.8%	15.8 [13.7, 18.2]	122.7%	1.32	−5.0%	437.5 [374.9, 504.0]	111.8%	1.34	−4.3%
Southern Latin America	3.4 [3.2, 3.7]	4.8%	1.15	5.3%	3.6 [3.3, 3.9]	3.6%	1.16	6.0%	76.8 [72.1, 82.1]	−6.7%	1.14	4.8%
Southern Sub-Saharan Africa	6.4 [5.9, 7]	106.8%	1.20	−7.2%	6.6 [6, 7.2]	107.0%	1.20	−7.7%	185.8 [169.4, 204.4]	96.6%	1.21	−5.1%
Tropical Latin America	12.8 [12.1, 13.3]	109.6%	1.10	2.6%	13.1 [12.4, 13.7]	108.2%	1.10	2.8%	348.5 [331.5, 362.4]	96.1%	1.09	3.1%
Western Europe	38.4 [35.5, 40.2]	39.1%	1.13	6.4%	34.4 [31.5, 36.1]	26.1%	1.15	7.1%	712.1 [672.4, 741.3]	9.4%	1.10	4.8%
Western Sub-Saharan Africa	8.1 [6.1, 9.8]	253.0%	1.60	6.1%	8.5 [6.4, 10.2]	250.8%	1.60	6.9%	235.2 [175.3, 283.9]	255.3%	1.62	5.4%

*Abbreviations: PC* percentages of changes in 1990-2021, *M/F* male-to-female ratios, *DALY* disability-adjusted life-years, *SDI* socio-demographic indexes, *WHO* World Health Organization, *GBD* Global Burden of Disease

### ASRs of regional EC burdens

In terms of ASRs, East Asia, Southern Sub-Saharan Africa, and Eastern Sub-Saharan Africa were the GBD regions facing the heaviest EC burdens. In 2021, the GBD regions with the highest EC ASIRs were East Asia, Southern Sub-Saharan Africa, and Eastern Sub-Saharan Africa, with rates of 14.83 [11.94, 18.09], 11.01 [10.06, 11.99], and 10.93 [9.14, 13.0], respectively. Conversely, the regions with the lowest incidence rates were Oceania, Central Latin America, and Andean Latin America, with rates of 1.81 [1.43, 2.28], 1.54 [1.37, 1.73], and 1.38 [1.14, 1.70], respectively. Regarding ASDR, the highest rates were observed in East Asia (13.91 [11.23, 16.84]), Eastern Sub-Saharan Africa (11.74 [9.82, 14.12]), and Southern Sub-Saharan Africa (11.69 [10.68, 12.72]), while the lowest were in Andean Latin America (1.51 [1.24, 1.85]), Central Latin America (1.65 [1.47, 1.85]), and Oceania (1.95 [1.54, 2.45]). In 2021, the regions with the highest ASDALYR were East Asia (313.94 [252.18, 387.12]), Southern Sub-Saharan Africa (297.67 [271.83, 326.47]), and Eastern Sub-Saharan Africa (292.22 [243.43, 352.18]). Conversely, the regions with the lowest ASDALYR were Andean Latin America (32.27 [26.17, 39.76]), Central Latin America (37.71 [33.67, 42.31]), and Oceania (47.1 [37.29, 59.95]). Compared to 1990, the ASIR of EC has decreased in almost all regions, with the most significant declines observed in Central Asia (−65.39%), Southern Latin America (−45.75%), and Central Latin America (−40.08%). In contrast, there was an increase in ASIR in Western Sub-Saharan Africa (59.25%) and high-income North America (1.94%). In terms of ASDR, Western Sub-Saharan Africa experienced a significant increase of 60.14%, whereas Central Asia (−65.35%), Southern Latin America (−47.48%), and East Asia (−45.30%) saw the largest decreases. The most substantial declines in ASDALYR were observed in Central Asia (−66.48%), East Asia (−50.83%), and Southern Latin America (−49.85%). Western Sub-Saharan Africa remained the only GBD region to show an increase in ASDALYR, with an approximate rise of more than half (54.76%) over the past 32 years.

Among the SDI quintiles, the heaviest ASR burdens of EC have been shifting from middle SDI regions to high-middle SDI locations. In 2021, the high-middle SDI region recorded the highest ASIR, ASDR, and ASDALYR, at 8.84 [7.26, 10.70], 8.13 [6.72, 9.77], and 192.56 [158.7, 234.03], respectively. The middle SDI region, once the area with the highest EC incidence rate, saw a 40.79% reduction over the past 32 years, leading to the high-middle SDI region overtaking its position. Similarly, the middle SDI region recorded the largest proportional reduction in ASDR and ASDALYR among the SDI quintiles, with decreases of −44.72% and − 50.69%, respectively (Table 2).

**Table 2:** The age-standardized burden metrics (ASIR, ASDR, and ASDALYR), and their male/female ratios for esophageal cancer at global level, in SDI quintiles, WHO regions and GBD regions in 2021, and their changing trends in 1990–2021

locations	ASIR (per 100,000 population)					ASDR (per 100,000 population)					ASDALYR (per 100,000 population)				
	Rate in 2021	Changing trends		M/F ratio		Rate in 2021	Changing trends		M/F ratio		Rate in 2021	Changing trends		M/F ratio	
		PC	AAPC/%	M/F	PC		PC	AAPC/%	M/F	PC		PC	AAPC/%	M/F	PC
**Global**	6.7 [5.9, 7.5]	−24.9%	−0.9 [−1.0, −0.9]	3.3	25.7%	6.3 [5.5, 7.0]	−30.7%	−1.2 [−1.2, −1.2]	3.4	30.0%	148.6 [131.7, 166.8]	−36.9%	−1.5 [−1.5, −1.5]	3.5	24.4%
**SDI quintiles**															
High SDI	4.9 [4.6, 5.2]	−7.8%	−0.3 [−0.3, −0.3]	4.4	5.5%	4.0 [3.8, 4.2]	−18.5%	−0.7 [−0.7, −0.6]	4.6	8.6%	94.0 [89.3, 97.9]	−24.2%	−0.9 [−0.9, −0.8]	5.0	2.4%
High-middle SDI	8.8 [7.3, 10.7]	−20.9%	−0.8 [−0.8, −0.7]	4.6	33.4%	8.1 [6.7, 9.8]	−29.4%	−1.2 [−1.2, −1.1]	4.7	38.1%	192.6 [158.7, 234.0]	−36.5%	−1.5 [−1.6, −1.4]	5.4	41.3%
Middle SDI	8.1 [6.8, 9.6]	−40.8%	−1.7 [−1.7, −1.6]	3.3	48.4%	7.9 [6.7, 9.3]	−44.7%	−1.9 [−1.9, −1.9]	3.5	57.2%	180.7 [153.2, 214.6]	−50.6%	−2.3 [−2.3, −2.2]	3.7	51.4%
Low-middle SDI	3.6 [3.2, 4.2]	−12.3%	−0.4 [−0.5, −0.4]	1.6	22.4%	3.8 [3.4, 4.4]	−13.0%	−0.4 [−0.5, −0.4]	1.6	22.8%	97.1 [87.7, 111.8]	−14.7%	−0.5 [−0.5, −0.5]	1.7	28.1%
Low SDI	5.5 [4.7, 6.3]	−17.9%	−0.6 [−0.6, −0.6]	1.3	9.2%	5.9 [5.0, 6.8]	−17.6%	−0.6 [−0.6, −0.6]	1.3	9.6%	148.7 [126.1, 172.2]	−19.8%	−0.7 [−0.7, −0.7]	1.4	15.3%
**WHO regions**															
African Region	7.2 [6.1, 8.3]	614.5%	−0.2 [−0.3, −0.2]	1.6	9.1%	7.8 [6.6, 8.9]	−7.2%	−0.2 [−0.3, −0.2]	1.6	9.5%	192.3 [162.9, 222.7]	−11.0%	−0.4 [−0.4, −0.4]	1.8	19.9%
Eastern Mediterranean Region	3.5 [3.0, 4.0]	−15.1%	−0.5 [−0.5, −0.5]	1.1	20.2%	3.7 [3.2, 4.3]	−15.5%	−0.5 [−0.6, −0.5]	1.1	20.2%	90.0 [76.3, 103.1]	−19.1%	−0.7 [−0.7, −0.6]	1.1	18.2%
European Region	3.7 [3.5, 3.8]	−23.2%	−0.8 [−0.9, −0.8]	3.9	1.2%	3.3 [3.1, 3.5]	−30.2%	−1.1 [−1.2, −1.1]	4.0	7.1%	81.7 [77.7, 84.6]	−33.9%	−1.3 [−1.4, −1.2]	4.3	−0.6%
Region of the Americas	3.7 [3.5, 3.8]	−15.8%	−0.6 [−0.6, −0.5]	3.9	18.7%	3.5 [3.3, 3.6]	−18.9%	−0.7 [−0.7, −0.7]	4.1	19.3%	84.7 [81.3, 87.7]	−21.9%	−0.8 [−0.9, −0.8]	4.4	17.8%
South-East Asia Region	3.1 [2.8, 3.7]	−15.7%	−0.5 [−0.5, −0.5]	1.7	28.2%	3.2 [2.9, 3.8]	−17.0%	−0.6 [−0.6, −0.5]	1.7	28.3%	82.5 [73.5, 97.1]	−20.1%	−0.7 [−0.8, −0.7]	1.7	27.0%
Western Pacific Region	12.3 [10.0, 14.8]	−36.1%	−1.4 [−1.5, −1.4]	4.1	51.9%	11.2 [9.1, 13.4]	−43.2%	−1.8 [−1.8, −1.8]	4.3	59.6%	256.1 [206.6, 313.0]	−49.1%	−2.2 [−2.3, −2.1]	4.8	61.7%
**GBD regions**															
Andean Latin America	1.4 [1.1, 1.7]	−38.8%	−1.0 [−1.2, −0.9]	2.8	15.8%	1.5 [1.2, 1.9]	−30.7%	−1.1 [−1.2, −0.9]	2.9	19.6%	32.3 [26.2, 39.8]	−50.8%	−1.2 [−1.4, −1.1]	2.8	16.4%
Australasia	4.1 [3.7, 4.3]	−9.9%	−0.3 [−0.4, −0.2]	3.0	34.1%	3.7 [3.3, 3.9]	−15.2%	−0.5 [−0.6, −0.4]	3.3	34.7%	80.2 [74.1, 85.5]	−16.2%	−0.8 [−0.9, −0.7]	3.9	39.3%
Caribbean	3.6 [3.2, 4.1]	−14.4%	−0.2 [−0.3, −0.1]	4.0	54.3%	3.7 [3.2, 4.2]	−12.0%	−0.3 [−0.4, −0.2]	4.0	54.5%	94.3 [82.3, 107.5]	−16.0%	−0.1 [−0.2, 0.0]	4.3	57.2%
Central Asia	4.4 [4.0, 4.9]	−65.4%	−3.5 [−3.6, −3.3]	1.8	−6.4%	4.7 [4.3, 5.3]	−65.3%	−3.4 [−3.6, −3.3]	1.8	−6.4%	115.5 [102.9, 129.0]	−66.5%	−3.6 [−3.7, −3.4]	1.8	−6.2%
Central Europe	2.7 [2.5, 3.0]	−5.5%	−0.1 [−0.3, −0.1]	5.4	17.7%	2.8 [2.5, 3.0]	−8.4%	−0.3 [−0.3, −0.2]	5.2	18.9%	73.1 [67.2, 79.0]	−10.4%	−0.3 [−0.4, −0.2]	6.1	15.0%
Central Latin America	1.5 [1.4, 1.7]	−29.2%	−1.8 [−1.9, −1.8]	3.1	57.8%	1.7 [1.5, 1.9]	−42.0%	−1.9 [−2.0, −1.8]	3.1	60.0%	37.7 [33.7, 42.3]	−31.9%	−1.9 [−2.0, −1.8]	3.3	57.2%
Central Sub-Saharan Africa	8.3 [6.0, 10.6]	−16.0%	−0.8 [−0.8, −0.8]	1.8	−4.0%	8.9 [6.4, 11.5]	−21.7%	−0.8 [−0.8, −0.8]	1.8	−3.0%	221.5 [161.8, 289.3]	−39.6%	−0.9 [−0.9, −0.8]	1.8	−6.6%
East Asia	14.8 [11.9, 18.1]	−9.4%	−1.6 [−1.6, −1.5]	3.9	54.9%	13.9 [11.2, 16.8]	−45.3%	−2.0 [−2.0, −1.9]	4.2	66.4%	313.9 [252.2, 387.1]	−18.9%	−2.3 [−2.3, −2.2]	4.7	68.7%
Eastern Europe	3.1 [2.8, 3.4]	−13.5%	−1.0 [−1.2, −0.8]	6.9	29.0%	2.9 [2.7, 3.2]	−33.2%	−1.1 [−1.3, −1.0]	6.6	32.1%	81.3 [73.2, 88.5]	−29.5%	−1.1 [−1.3, −0.9]	7.4	20.0%
Eastern Sub-Saharan Africa	10.9 [9.1, 13.1]	−45.8%	−0.7 [−0.7, −0.7]	1.2	−1.0%	11.7 [9.8, 14.1]	−19.1%	−0.7 [−0.7, −0.7]	1.2	0.0%	292.2 [243.4, 352.2]	−49.9%	−0.8 [−0.8, −0.8]	1.3	3.5%
High-income Asia Pacific	5.5 [5.0, 5.8]	2.1%	−0.6 [−0.7, −0.6]	6.5	12.2%	3.4 [3.1, 3.6]	−33.3%	−1.3 [−1.4, −1.3]	6.7	10.9%	74.5 [69.3, 78.5]	−8.9%	−1.7 [−1.7, −1.6]	6.5	−8.6%
High-income North America	4.2 [4.0, 4.4]	−7.9%	0.0 [0.0, 0.1]	4.2	16.8%	3.6 [3.4, 3.8]	−3.0%	−0.1 [−0.2, −0.1]	4.9	21.8%	86.1 [82.5, 88.8]	−5.7%	−0.3 [−0.4, −0.3]	5.1	17.4%
North Africa and Middle East	2.0 [1.7, 2.2]	−29.5%	−0.8 [−0.9, −0.8]	1.5	32.4%	2.1 [1.8, 2.4]	−24.3%	−0.9 [−0.9, −0.9]	1.6	40.1%	48.0 [40.4, 54.4]	−34.0%	−1.2 [−1.2, −1.1]	1.5	32.3%
Oceania	1.8 [1.4, 2.3]	−40.1%	−0.5 [−0.6, −0.5]	2.6	14.9%	2.0 [1.5, 2.5]	−14.9%	−0.5 [−0.6, −0.5]	2.6	18.0%	47.1 [37.3, 60.0]	−40.6%	−0.6 [−0.6, −0.5]	2.8	19.3%
South Asia	3.4 [3.0, 4.0]	−25.9%	−0.4 [−0.5, −0.4]	1.4	21.3%	3.5 [3.1, 4.3]	−15.1%	−0.5 [−0.5, −0.4]	1.5	29.8%	91.1 [80.5, 108.5]	−27.1%	−0.6 [−0.7, −0.6]	1.4	20.9%
Southeast Asia	2.4 [2.1, 2.8]	−23.2%	−0.3 [−0.4, −0.3]	2.6	50.5%	2.4 [2.1, 2.8]	−13.9%	−0.5 [−0.5, −0.5]	2.6	50.9%	61.7 [53.2, 70.8]	−30.3%	−0.6 [−0.6, −0.5]	2.8	58.4%
Southern Latin America	3.9 [3.6, 4.2]	−14.5%	−1.9 [−2.0, −1.8]	2.8	−4.6%	4.1 [3.8, 4.4]	−47.5%	−2.0 [−2.1, −1.9]	2.8	−2.7%	88.9 [83.6, 95.0]	−17.6%	−2.1 [−2.2, −2.1]	3.0	−9.1%
Southern Sub-Saharan Africa	11.0 [10.1, 12.0]	−22.0%	−0.1 [−0.2, 0.1]	2.3	−1.8%	11.7 [10.7, 12.7]	−1.6%	−0.1 [−0.2, 0.1]	2.3	−2.3%	297.7 [271.8, 326.5]	−24.0%	−0.3 [−0.4, −0.1]	2.5	4.8%
Tropical Latin America	4.9 [4.6, 5.1]	−19.4%	−1.0 [−1.0, −0.9]	4.1	16.9%	5.1 [4.8, 5.3]	−27.9%	−1.0 [−1.1, −1.0]	4.0	16.8%	132.1 [125.6, 137.3]	−21.7%	−1.1 [−1.1, −1.0]	4.5	15.6%
Western Europe	4.3 [4.0, 4.4]	−1.9%	−0.5 [−0.6, −0.4]	3.6	−12.6%	3.7 [3.4, 3.8]	−24.0%	−0.9 [−1.0, −0.8]	3.7	−9.1%	85.4 [81.5, 88.6]	−6.5%	−1.1 [−1.2, −1.1]	4.1	−16.7%
Western Sub-Saharan Africa	4.2 [3.2, 5.0]	59.3%	1.5 [1.5, 1.5]	2.4	47.5%	4.6 [3.4, 5.4]	60.1%	1.5 [1.5, 1.6]	2.3	43.7%	110.0 [82.4, 132.2]	54.7%	1.4 [1.4, 1.5]	2.6	50.7%

*Abbreviations:PC *percentages of changes, *M/F* male-to-female ratios, *DALY* disability-adjusted life-years, *SDI* socio-demographic indexes, *WHO* World Health Organization, *GBD* Global Burden of Disease, *ASIR* age-standardized rate of incidence, *ASDR* age-standardized rate of death, *ASDALYRs* age-standardized rate of DALYs

The EC ASPoD given by GBD 2021 is shown in eFigure S1. It could be seen that the effect of COVID-19 on deaths in the population led to a decrease in the proportion of deaths due to EC in the population in 2020–2021 in the majority of regions, with this feature being most pronounced in Sub-Saharan Africa regions. During the pre-pandemic period, there appeared to be a gradual increase in the EC PoD in the vast majority of regions, but in Central Asia and Southern Latin America, the EC ASPoD has declined considerably in recent years. East Asia, on the other hand, had a very specific ASPoD profile. EC ASPoD in East Asia showed a significant decline in the early part of the 21st century, but since as recently as 2015, EC ASPoD has shown a small increase in East Asia, even in the period 2020–2021, and this paradoxical change in the proportion of all-cause deaths accounted for by EC in East Asia hints at the specificity of the epidemiological characteristics of EC in East Asia.

### Absolute numbers of regional EC burdens

As for the absolute burden numbers, in 2021, the GBD regions with significant EC incidence cases included East Asia, South Asia, and Western Europe, with respective case numbers of 327,706 [263,648, 401,882], 50,081 [44,230, 59,870], and 38,417 [35,454, 40,217]. Conversely, the GBD regions with the lowest EC incidence numbers in 2021 were the Caribbean, Andean Latin America, and Oceania, with numbers of 1,952 [1,713, 2,207], 802 [656, 987], and 131 [103, 166], respectively. Regarding deaths, the highest numbers in 2021 were observed in East Asia (302,582 [243,363, 368,743]), South Asia (51,542 [45,651, 61,688]), and Western Europe (34,397 [31,525, 36,115]), while the lowest numbers of deaths in 2021 were observed in Oceania (134 [106, 170]), Andean Latin America (866 [712, 1,063]), and the Caribbean (1,997 [1,755, 2,254]). In 2021, the regions with the highest DALY numbers were East Asia (7,069,760.58 [5,660,280.66, 8,736,103.07]), South Asia (1,434,796.86 [1,268,757.00, 1,704,573.72]), and Western Europe (712,093.47 [672,400.78, 741,301.38]). Conversely, the regions with the lowest DALYs in 2021 were Oceania (3,905.66 [3,055.62, 5,016.37]), Andean Latin America (19,167.84 [15,515.38, 23,633.94]), and Australasia (41,016.96 [37,625.99, 43,814.77]). Compared to 1990, the change in the number of EC cases was less optimistic than the change in ASIR, with only Central Asia (−39.95%) and Eastern Europe (−13.59%) showing a decline. In contrast, regions such as Western Sub-Saharan Africa (252.95%), Southeast Asia (132.36%), Oceania (118.71%), and South Asia (115.66%) experienced an increase in the number of cases exceeding 110%. In terms of deaths, compared to 1990, Central Asia (−40.52%) and Eastern Europe (−17.36%) saw decreases. However, in other regions, the number of deaths increased, with Western Sub-Saharan Africa (250.76%), Southeast Asia (122.70%), Oceania (118.31%), and South Asia (116.32%) experiencing death toll increases of more than 110%. As for DALYs, decreases were observed in Central Asia (−40.33%), Eastern Europe (−19.59%), and Southern Latin America (−6.73%). However, other regions experienced increases, with Western Sub-Saharan Africa (255.26%), Oceania (114.02%), Southeast Asia (111.79%), and South Asia (100.73%) showing growth of over 100%.

Among the SDI quintiles, the absolute EC burden measured in incidence, deaths, and DALY numbers has shown distinct patterns of change from 1990 to 2021. All SDI quintiles recorded substantial increases across three metrics, while the low-middle SDI region experienced the most obvious increases in proportion; its EC incident cases surged by 105.73% (from 25,326 [22,893, 29,038] in 1990 to 52,104 [47,166, 59,926] in 2021), death cases by 105.52% (from 26,141 [23,617, 30,035] in 1990 to 53,724 [48,513, 61,806] in 2021), and DALY numbers by 95.47% (from 763,111 [692,292, 878,969] in 1990 to 1,491,634 [1,348,142, 1,724,084] in 2021), where the population growth, aging, and the improvement of diagnostic systems play certain roles. Although the middle SDI region and high-middle SDI region showed modest growths over all three metrics compared to the low-middle and low SDI regions, they contributed large proportions of the global EC burden numbers. Taking DALYs as an example, the middle and high-middle SDI regions accounted for 38.57% (5,011,783 [4,233,898, 5,964,294]) and 29.51% (3,834,291 [3,157,464, 4,667,629]) of global EC DALYs, respectively (Table 1).

### ASRs of EC burdens among countries and territories

**Fig. 2:**
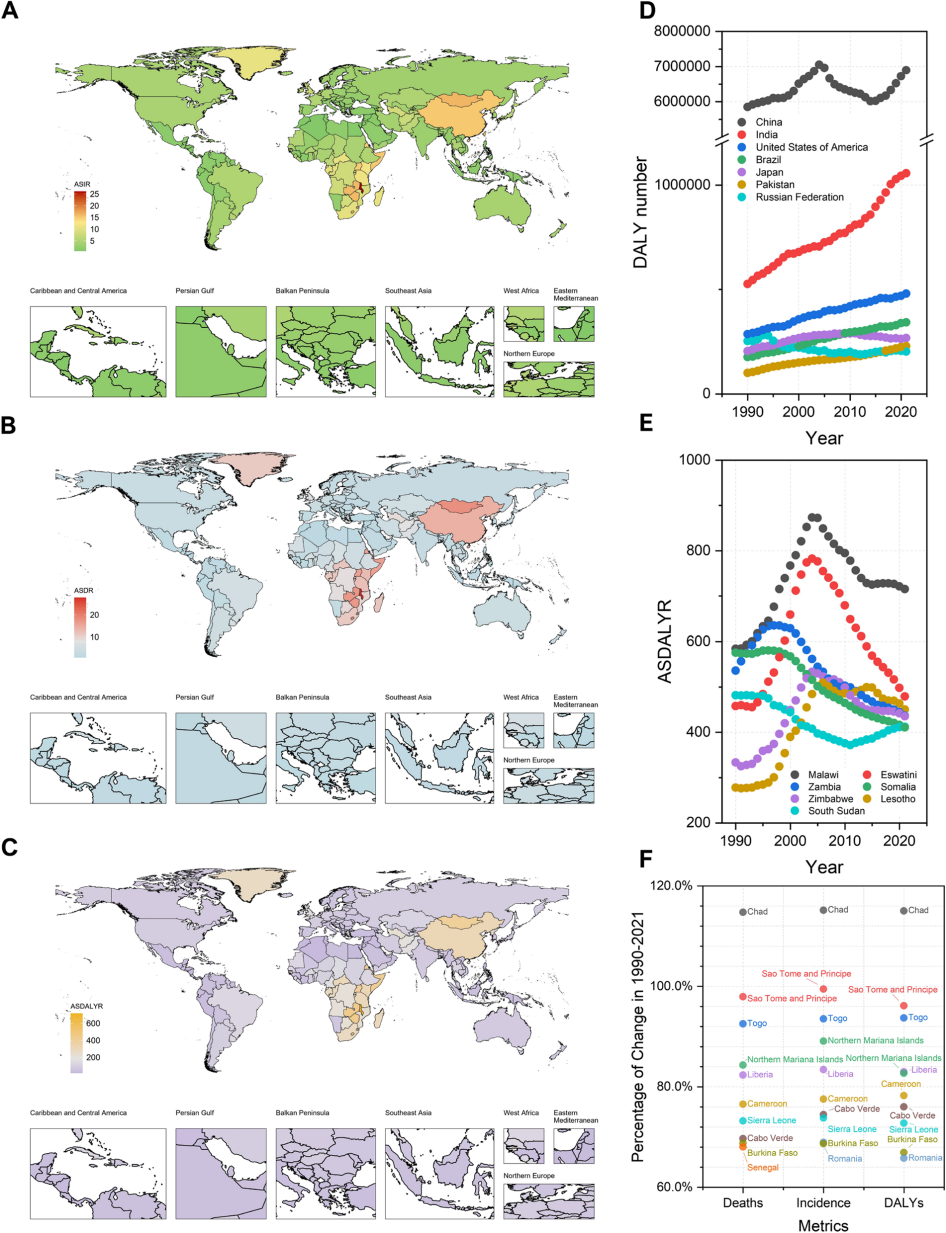
Global esophageal cancer burdens in 204 countries and territories and ranks of the leading locations. The ASRs (per 100,000 population) of esophageal cancer incidence (**A**), death (**B**), and DALY (**C**) in 2021 worldwide. The trend of DALY number (**D**) and ASDALYR (**E**) of the top seven countries with the highest ASRs in 2021 for esophageal cancer from 1990 to 2021. The percentage change in deaths, incidences, and DALYs (**F**) for countries with the highest increases in ASR burdens for esophageal cancer from 1990 to 2021

Consistent with previous research findings, our study indicates that the regions with the highest incidence and mortality rates of EC are predominantly located in the Asian Esophageal Cancer Belt, which stretches from northern China through Central Asia to northern Iran [32, 34], and the African Esophageal Cancer Corridor, which extends from northern to southern parts of eastern Africa, running from Ethiopia to eastern South Africa [35]. In 2021, people in Malawi, Eswatini, Mongolia, Zambia, and Zimbabwe had the top five EC ASIRs: 26.06 [21.02, 32.46], 16.68 [11.80, 22.42], 16.25 [13.15, 19.45], 16.02 [10.95, 24.45], and 15.93 [12.31, 19.64] per 100,000 population, respectively (Fig. 2A, eTable S6). They also had among the highest EC ASDRs (Malawi 27.77 [22.45, 34.72], Mongolia 17.98 [14.50, 21.49], Eswatini 17.47 [12.43, 23.38], Zambia 17.13 [11.78, 26.09], Zimbabwe 17.02 [13.23, 21.11] per 100,000 population) (Fig. 2B, eTable S6). Malawi, Eswatini, Lesotho, Zambia, and Zimbabwe were the countries with the most EC ASDALYRs: 715.28 [572.77, 904.41], 478.85 [332.56, 665.21], 450.01 [327.62, 588.49], 436.30 [290.09, 677.10], and 435.15 [329.44, 549.19] per 100,000 population, respectively (Fig. 2C, eTable S6).

With the exception of countries like the Northern Mariana Islands, the vast majority of nations that experienced significant growth in the burden of EC ASR between 1990 and 2021 are located in Africa, such as Chad (114.76% in ASDR, for example), Sao Tome and Principe (97.93%), Togo (92.53%), and Liberia (82.33%). Regarding ASDR, 52 out of 204 countries (25.5%) saw an increase, with fifteen (7.4%) experiencing less than a 10% rise, ten (4.9%) a 10–20% rise, twelve (5.9%) a 20–50% rise, and fifteen (7.4%) a significant increase of over 50%. In contrast, 152 countries (74.5%) showed a decrease: 23 (11.3%) with a modest 0–10% decline, 38 (18.6%) with a 10–20% decline, 77 (37.7%) with a 20–50% decline, and 14 (6.9%) with a dramatic drop of more than 50%. For ASDALYRs, the top five nations with the highest increases were Chad (115.03%), São Tomé and Príncipe (96.13%), Togo (93.71%), Liberia (82.96%), and the Northern Mariana Islands (82.68%). Compared to 1990, 157 countries (76.96%) reduced EC ASDALYR by 2021. Among these, 25 countries (12.25%) had ASDALYR declines between 0 and 10%, 39 countries (19.12%) experienced declines between 10 and 20%, 74 countries (36.27%) saw declines between 20 and 50%, and fifteen countries (7.35%) had declines of more than 50%. As for the EC ASIRs in 2021, there was an increase in 64 countries (31.37%) compared to 1990. Among these, 23 countries (11.27%) experienced ASIR increases between 0 and 10%, ten countries (4.90%) had increases between 10 and 20%, sixteen countries (7.84%) had increases between 20 and 50%, and fifteen countries (7.35%) had increases of more than a half. On the other hand, 22 countries (10.78%) had ASIR declines between 0 and 10%, 36 countries (17.65%) saw declines between 10 and 20%, 73 countries (35.78%) experienced declines between 20 and 50%, and nine countries (4.41%) had declines of more than 50%. Chad (115.15%), São Tomé and Príncipe (99.46%), Togo (93.51%), the Northern Mariana Islands (89.09%), and Liberia (83.41%) experienced the most ASIR increases (eTable S5-7, Fig. 2F). The mitigation trends of EC burden in 1990–2021 have significant geographical patterns. Taking ASIR as an example, four countries with the highest reduction rates (Kazakhstan − 75.17%, Uzbekistan − 73.83%, Turkmenistan − 71.49%, and Kyrgyzstan − 65.91%) were all located in Central Asia. In countries with > 30% EC ASIR mitigation during 1990–2021, the Americas accounted for 27.08% of them, including Puerto Rico (−60.05%), Chile (−58.57%), and Colombia (−56.55%), with Europe accounting for 14.58% (e.g., Italy − 46.86%, France − 40.79%, Andorra − 32.51%), Africa accounting for 12.50% (Ethiopia − 47.24%, Rwanda − 46.46%, Burundi − 44.20%, etc.), Central Asia accounting for 10.42%, Middle East accounting for 10.42% (Qatar − 48.47%, Kuwait − 48.42%, Bahrain − 43.42%, etc.), and other regions, including South Asia (Maldives − 60.24%, Bangladesh − 36.27%), East Asia (Republic of Korea − 41.48%, China − 39.35%, Mongolia − 30.17%), and Southeast Asia (Singapore − 45.10%, Lao People’s Democratic Republic − 42.90%, Cambodia − 30.02%, etc.), comprising the remaining proportion. The data revealed a notable concentration of countries with the highest reduction rates in Central Asia, indicating exceptional regional performance in EC control strategies. The Americas and Europe also showed significant representation in locations with the most EC ASIR reductions, particularly with the inclusion of Latin American countries. Meanwhile, Africa, the Middle East, and other regions demonstrated a relatively balanced distribution.

To further investigate the changes of temporal trends for EC burden metrics among 204 countries and territories during the COVID-19 pandemic in 2020–2021, using Joinpoint analysis (detailed in Supplementary Note S2), we identified locations with linear epidemiological trends in 72.5% (148/204) and 71.1% (145/204) of 204 countries/territories for EC ASRs, respectively. To quantify the deviation between projected and actual values, we defined the Percentages of Predicted-value and Real-value Difference (PPRD%). A smaller absolute PPRD% reflects a closer alignment between the Joinpoint prediction and observed data, while a positive PPRD% indicates an increase in metrics compared to the projection, and a negative value indicates a decrease.

As for the ASIR, the great EC incidence losses or surges compared to the Joinpoint forecasting happened in some locations during 2020–2021, coinciding with the global COVID-19 outbreak and pandemic. In 2020–2021, significant increases in ASIR exceeding 5% were observed in three countries: Bolivarian Republic of Venezuela (9.46%), Grenada (7.68%), and Republic of Belarus (6.39%). The PPRD distribution for ASIR in other countries was as follows: nine countries had increases between 2 and 5%, two between 1 and 2%, 49 between 0 and 1%, 51 between − 1% and 0%, 16 between − 2% and − 1%, and 16 between − 5% and − 2%. The sole country with an ASIR decline exceeding 5% was Republic of Trinidad and Tobago (−5.31%). Notable fluctuations occurred in the Republic of San Marino (−11.81% in 2020, 8.93% in 2021), United Arab Emirates (−8.07% in 2020, 2.58% in 2021), Principality of Andorra (−6.45% in 2020, 3.61% in 2021), and Guam (−5.28% in 2020, 2.90% in 2021). Conversely, Grenada (4.13% in 2020, 11.53% in 2021) and the Republic of Belarus (6.67% in 2020, 6.10% in 2021) showed substantial relative increases, while Republic of Trinidad and Tobago (−4.11% in 2020, −6.48% in 2021), Republic of Mauritius (−3.14% in 2020, −5.91% in 2021), and Republic of Guyana (−3.64% in 2020, −5.30% in 2021) exhibited marked declines.

For EC ASDR in 2020–2021, significant increases or declines compared to the projections also existed in some locations. Significant increases exceeding 5% were noted in Grenada (7.19%) and Republic of Belarus (6.52%). The PPRD distribution for ASDR across other countries was as follows: eight countries had increases between 2 and 5%, four between 1 and 2%, 49 between 0 and 1%, 46 between − 1% and 0%, 14 between − 2% and − 1%, and 19 between − 5% and − 2%. The two countries with ASDR losses exceeding 5% were Grand Duchy of Luxembourg (−5.17%) and Republic of Trinidad and Tobago (−5.10%). Pronounced fluctuations were observed in the Republic of San Marino (−12.35% in 2020, 10.04% in 2021), United Arab Emirates (−8.18% in 2020, 3.02% in 2021), and Principality of Andorra (−6.82% in 2020, 3.15% in 2021). Conversely, significant relative increases were recorded in Republic of Belarus (6.67% in 2020, 6.37% in 2021) and Grenada (3.81% in 2020, 10.86% in 2021), while Republic of Trinidad and Tobago (−3.93% in 2020, −6.26% in 2021), Republic of Mauritius (−3.15% in 2020, −5.50% in 2021), and Grand Duchy of Luxembourg (−5.88% in 2020, −4.45% in 2021) experienced notable relative declines.

### Absolute numbers of EC burdens among countries and territories

Owing to differences in population sizes, high EC burden rates do not necessarily equate to high absolute numbers. In terms of absolute incidence in 2021, the countries with the highest numbers were China (320,805 [256,102, 394,756]), India (37,007 [32,443, 44,293]), the United States (24,329 [22,769, 25,279]), Japan (21,932.89 [19,705, 23,180]), Brazil (12,532.77 [11,855, 13,069]), the United Kingdom (9,949 [9,232, 10,365]), and Germany (8,290 [7,570, 8,901]), each with more than 8,000 cases. Regarding mortality, the countries with the highest numbers were China (296,443 [236,648, 362,831]), India (38,002 [33,296, 45,562]), the United States (21,341 [19,927, 22,216]), Japan (14,565 [12,957, 15,419]), Brazil (12,870 [12,150, 13,441]), the United Kingdom (9,746 [8,990, 10,170]), Pakistan (7,913 [6,283, 9,969]), and the Russian Federation (7,709 [7,022, 8,377]), each with more than 7,000 deaths. The countries with the highest DALYs were China (6,898,666 [5,471,181, 8,553,365]), India (1,056,497 [927,426, 1,250,177]), the United States (479,504 [457,854, 495,409]), Brazil (342,230 [325,426, 356,454]), Japan (267,046 [244,223, 279,927]), Pakistan (228,390 [182,178, 289,878]), and the Russian Federation (201,900 [182,635, 219,875]), each exceeding 200,000 DALYs (eTable S9).

The population growth and aging are producing increasing numbers of EC absolute burdens in most locations, especially in countries with simultaneous increasing ASRs and growing populations, such as Togo and Cameroon. In some locations, such as the Northern Mariana Islands, the increasing ASR played a vital role, while in some other countries/territories, the population growth greatly accounted for the increasing burden numbers, such as in the United Arab Emirates. As for the death numbers, 184 out of 204 countries (90.2%) experienced an increase, with six (2.9%) showing a rise of less than 10%, 10 (4.9%) with a 10–20% rise, 28 (13.7%) with a 20–50% rise, and a significant 140 (68.6%) showing an increase of over 50%. In contrast, 20 countries (9.8%) showed a decrease: nine (4.4%) with a modest 0–10% decline, three (1.5%) with a 10–20% decline, six (2.9%) with a 20–50% decline, and two (1.0%) with a dramatic drop of more than 50%. The locations with the most significant increases in death numbers were Togo (509.42%), the United Arab Emirates (492.77%), the Northern Mariana Islands (473.17%), Cameroon (405.44%), and Honduras (341.43%). As for DALY numbers, 180 out of 204 countries (88.2%) experienced an increase. Among them, eleven countries (5.4%) saw an increase of less than 10%, eight countries (3.9%) experienced a 10–20% increase, 29 countries (14.2%) had an increase between 20 and 50%, and a significant 132 countries (64.7%) showed an increase of over 50%. In contrast, 24 countries (11.8%) showed a decrease. Of these, seven countries (3.4%) had a decline of 0–10%, six countries (2.9%) experienced a 10–20% decline, nine countries (4.4%) had a decline of 20–50%, and two countries (1.0%) showed a dramatic drop of more than 50%. The locations with the most significant increases in DALY numbers were Togo (528.35%), the United Arab Emirates (510.42%), Cameroon (422.64%), the Northern Mariana Islands (377.79%), and Chad (369.48%). For incidence number, 188 countries (92.2%) experienced an increase. Among them, eight countries (3.9%) saw an increase of less than 10%, nine countries (4.4%) experienced a 10–20% increase, 23 countries (11.3%) had an increase between 20 and 50%, and a significant 148 countries (72.6%) showed an increase of over 50%. In contrast, sixteen countries (7.8%) showed a decrease. Of these, six countries (2.9%) had a decline of 0–10%, two countries (1.0%) experienced a 10–20% decline, six countries (2.9%) had a decline of 20–50%, and two countries (1.0%) showed a dramatic drop of more than 50%. The locations with the most significant increases in incident case numbers were the United Arab Emirates (522.55%), Togo (517.55%), the Northern Mariana Islands (461.54%), Cameroon (410.77%), and Qatar (352.19%) (eTable S8-10).

### Distribution of EC burdens among the different sexes and age groups

**Fig. 3:**
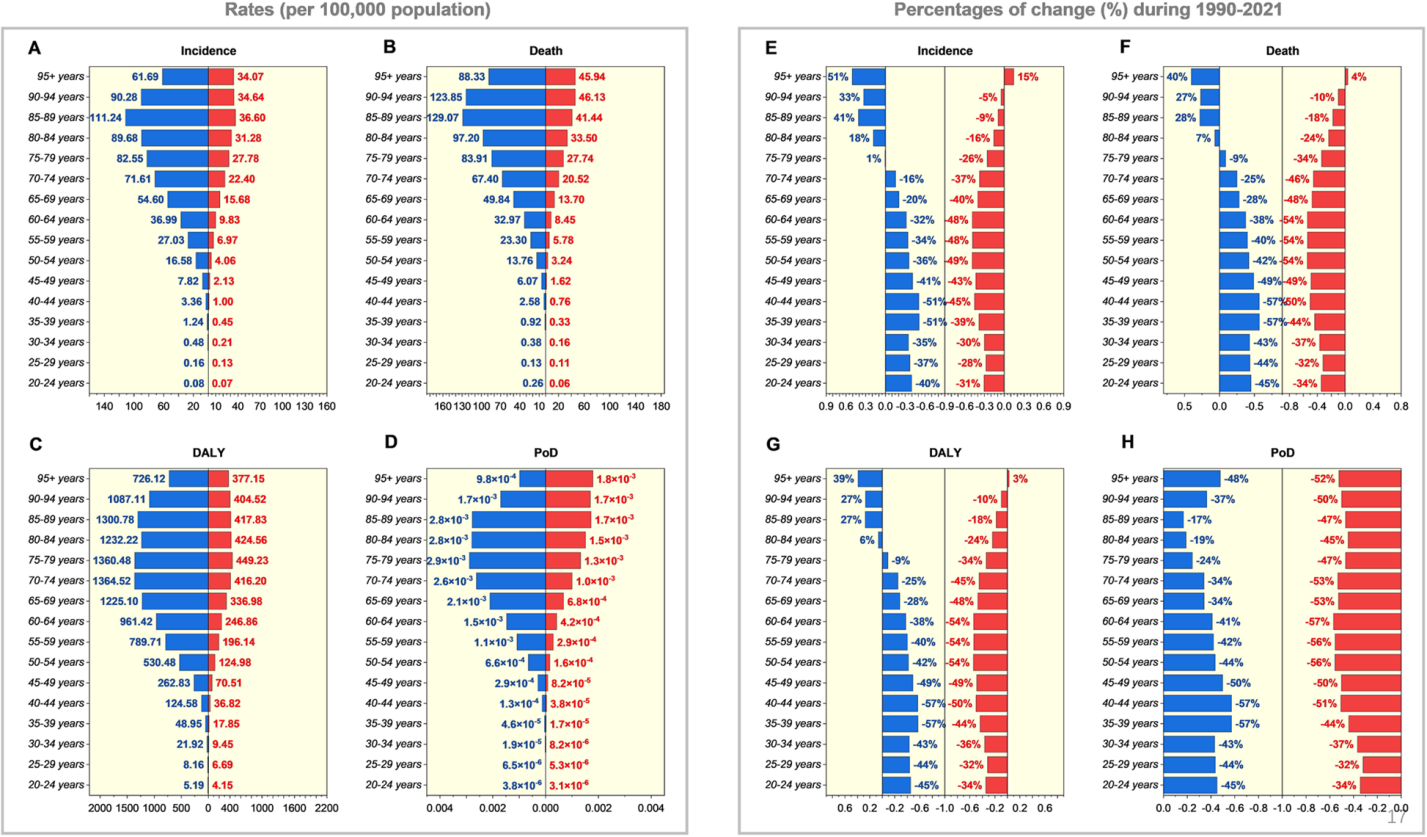
The esophageal cancer burden in different sexes and age-groups. Incidence (**A**), death (**B**), DALY (**C**) rates (per 100,000 population), and PoDs (**D**) of esophageal cancers among sex and age-groups in 2021 and their percentages of change during 1990–2021 (**E**-**H**)

Across all age groups, EC poses a more significant threat to males than females, particularly among older adults (Fig. 3). In the 16 age groups, categorized by five-year intervals, the highest incidence rates for both sexes were observed in the 85–89 age group, reaching 112.24 per 100,000 population in men and 34.64 in women. For those aged 70–94, the incidence ratio between men and women was approximately 3:1, indicating that older men are a key demographic for EC incidence (Fig. 3A). From 1990 to 2021, the incidence of EC among men over 75 has increased, with a notable rise of 51% and 41% in those over 95 and 85–89 years old, respectively. In contrast, among women, the incidence increase is limited to 15% in those over 95. EC incidence in middle-aged and younger populations has significantly declined over the past 32 years, with a reduction of nearly 50% in men aged 35–44 and women aged 50–64 (Fig. 3E). The age and sex distribution of mortality closely mirrors that of incidence (Fig. 3B and F), while the distribution of DALYR shows some differences (Fig. 3C and G). Among men aged 65–94, DALYR exceeds 1,000 per 100,000 population, suggesting that although EC may not be as lethal for men aged 65–80 as for older individuals, the disease still results in considerable healthy life years lost. The trends in death and DALY are similar, with declines observed in males under 80 and females under 95. However, the deaths and DALY due to EC have increased among older men in recent years (Fig. 3F and G). The probability of death (PoD) from EC is highest in men aged 70–89, exceeding 0.0025, whereas in women, PoD increases with age but never surpasses 0.002. Notably, PoD has decreased by 17–57% across all age groups in recent years, for both males and females (Fig. 3D and H).

### Trends of global EC burden metrices

**Fig. 4:**
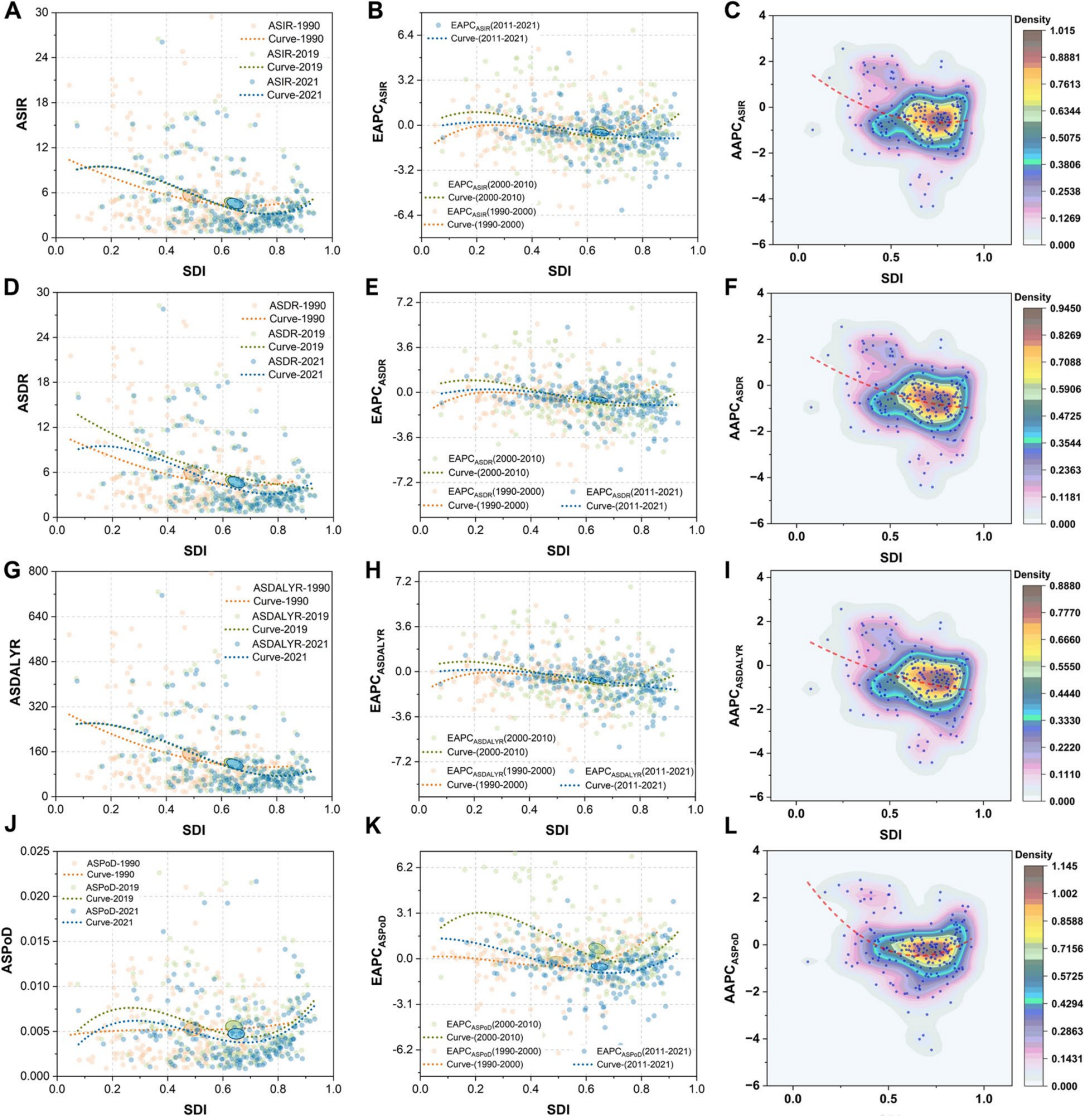
Trend analysis of esophageal cancer burdens in 204 nations and territories. The ASIR (**A**), ASDR (**D**), ASDALY rate (**G**), ASPoD (**J**) of esophageal cancers for both sexes in 204 nations and territories with different SDI values in 1990. 2019, and 2021. The EAPCs of ASIR (**B**), ASDR (**E**), ASDALY rate (**H**), and ASPoD (**K**) of esophageal cancers for both sexes in 204 nations and territories with different SDI values in three periods, 1990–2000, 2000–2010, and 2011–2021. The AAPCs of ASIR (**C**), ASDR (**F**), ASDALY rate (**I**), and ASPoD (**L**) of esophageal cancers for both sexes during 1990–2021 in 204 nations and territories with different SDI values. The AAPC values were obtained with Joinpoint epidemiological analysis in 204 locations. The cubic polynomial regressions with least absolute residual (LAR) optimizations were performed in (**A**-**H**) to depict the relationships between SDI values and the variables on the vertical axes, and the 95% confidence ellipse marking the centroid of the data points for that year were shown in (**A**-**B**), (**D**-**E**), (**G**-**H**), and (**J**-**K**). In (**C**), (**F**), (**I**), and (**L**), scatter density plot was given to show the kernel density estimation (KDE) of the data in contouring form, and the SDI values in these four panels were from data in 2021

The current status and mitigation trends of the EC burdens in 204 GBD countries and territories exhibit significant inequalities. Figure 4 illustrates the relationship between disease burden metrics, as well as their average trends, and SDI values using cubic polynomial regression curves, where each point stands for a country or territory. Each row in Fig. 4 delineates the analysis results for EC ASIR, ASDR, ASDALYR, and ASPoD. The first column of Fig. 4 illustrates the relationship between SDI and ASRs for the years 1990, 2019, and 2021, based on fitted results. Each point in the scatterplot represents one of the 204 countries, providing a cross-sectional analysis across three time points to depict the longitudinal association between EC ASR burden and national sociodemographic levels. The second column demonstrates the fitted relationship between EAPC and SDI during three distinct periods (1990–2000, 2000–2010, 2011–2021), aiming to capture the trends in EC ASR burden changes in relation to SDI over these intervals. The third column displays the results of AAPC derived from Joinpoint regression analysis of ASRs across 204 countries from 1990 to 2021. Presented through fitted curves and scatter density plots, it highlights the average temporal trends in EC ASR burden over 32 years and their associations with sociodemographic levels.

The regression results showed that in 1990, 2019, and 2021, EC ASIR, ASDR, and ASDALYR were higher in locations with lower SDI, indicating a significantly heavier EC burden in socioeconomically disadvantaged areas **(**Fig. 4A, D and G**)**. However, when examining the ASRs corresponding to SDI quintiles, it becomes evident that the highest ASRs are observed in high-middle, middle, and low SDI regions. This discrepancy arises from two key factors. First, the results depicted in Fig. 4A and D, and 4G are derived from regression analyses where individual countries are treated as observational units, whereas the ASRs corresponding to SDI quintiles reflect EC burden levels weighted by population within countries at the same SDI level. Second, this pattern underscores the pronounced geographical distribution of EC burden. A significant proportion of EC ASR burden is contributed by countries in East Asia, Central Asia, and Sub-Saharan Africa, most of which fall within these SDI ranges. For instance, considering ASIR, countries such as Malawi (26.06 [21.02, 32.46]) in Eastern Sub-Saharan Africa, Zambia (16.02 [10.95, 24.45]), Cabo Verde (15.15 [11.90, 18.91]) in Western Sub-Saharan Africa, Eswatini (16.68 [11.80, 22.42]), Zimbabwe (15.93 [12.31, 19.64]), and Lesotho (15.82 [11.67, 20.12]) in Southern Sub-Saharan Africa, Mongolia (16.25 [13.15, 19.45]) in Central Asia, and China (15.04 [12.04, 18.43]) in East Asia collectively contribute substantially to the ASIR burden. These countries, predominantly situated in the aforementioned SDI ranges, result in elevated EC ASIR levels within their respective SDI quintiles. This supports prior observations suggesting that while lower SDI levels are acknowledged to be associated with higher EC burdens, the high burden of EC may be more closely linked to specific geographical patterns, such as the Asian Esophageal Cancer Belt and African Esophageal Cancer Corridor [32].

Segmental trend changes depicted by EAPC reveal that from 1990 to 2000, ASIR, ASDR, and ASDALYR increased in high SDI regions but decreased in low SDI regions, particularly for ASIR, which reflected the disparities in progress. In 2000–2010, countries with extremely high and low SDI levels experienced an increase in ASIR, more pronounced in low SDI countries, while EAPC_ASDR_ and EAPC_ASDALYR_ were positive only in low SDI regions, suggesting a rise in ASRs in these areas. Given their already high baseline ASRs, this indicates growing inequality in the global EC burden during this period. From 2011 to 2021, EAPC_ASIR_ in low SDI regions approached zero, indicating no significant average change in incidence rates, whereas high SDI regions showed negative EAPC values, reflecting a declining trend in EC ASIR. The trends for ASDR and ASDALYR were similar to ASIR, demonstrating a more pronounced reduction in incidence, mortality, and healthy life years lost in high SDI countries compared to low SDI locations, thus highlighting the persistent global inequality in EC burden. Compared to other ASRs, the relationship between ASPoD and SDI is unique (Fig. 4J). In 1990, the global average relationship between ASPoD and SDI was relatively “flat.” However, in recent years, it has shown higher levels in both low-middle and high SDI regions. The EAPC_ASPoD_ on the fitted SDI-EAPC_ASPoD_ curve from 1990 to 2000 was positive for high SDI values but near zero for other SDI ranges, indicating an increase in EC PoD of high SDI countries and territories during that period. However, in the past decade, low SDI ranges have corresponded to positive EAPC_ASPoD_ values on the fitted relationship, while middle to high SDI countries tended to have negative EAPC_ASPoD_ values, suggesting an increased probability of death due to EC in low SDI locations, whereas this probability has, on average, decreased in high SDI areas (Fig. 4B, E and H, and 4K).

The AAPC results offered an overview of the average trends in the EC burden over the past 32 years. In regions with higher SDI levels, both incidence, mortality, and DALYR were associated with lower AAPCs, suggesting a more pronounced alleviation of the EC burden in areas with higher SDI levels. In terms of density, the majority of countries exhibited AAPC values within the range of [−2%, 1%], indicating that, at the national level, changes in the EC burden were not particularly significant in most countries. This observation (Fig. 4C, F and I, and 4L) aligns with the regional-level trends we have identified in Fig. 1. Our findings indicate that, although high-middle, middle, and low SDI regions exhibit relatively high EC ASRs, the AAPC trends in low SDI regions are less favorable compared to other regions (Table 2; Fig. 4C, F and I). Moreover, this disparity has become more pronounced during the period from 2011 to 2021 (Fig. 4B, E and H).

### Attributable risks and their trends during 1990–2021

**Fig. 5:**
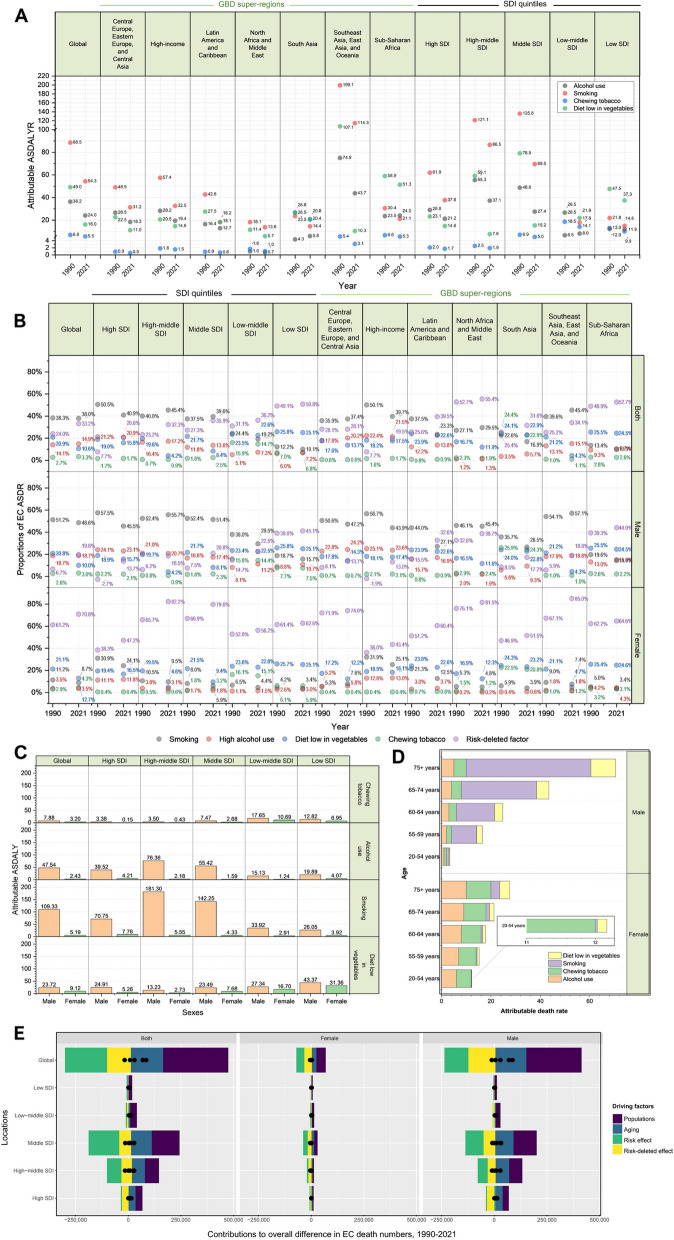
The risk factors of esophageal cancer burdens. The ASDALY rate (**A**) of EC attributable to four most detailed-level risk factors among seven GBD super regions and different SDI quintiles in 1990 and 2021. The proportions of contribution from four most detailed-level risk factors to EC ASDR among seven GBD super regions and SDI quintiles in 1990 and 2021 (**B**). The ASDALY (**C**) of esophageal cancer attributable to four most detailed-level risk factors in both sexes for different SDI quintiles in 2021. (**D**) The global death rate attributable to different three-level risks among five age-groups for both sexes in 2021. (**E**) Decomposition analysis results for drivers of the overall differences in EC death numbers in 1990–2021

The profile of EC burden attributable to four detailed GBD risk factors, smoking, high alcohol use, diet low in vegetables, and chewing tobacco, exhibited significant variation across regions and between sexes. In 2021, among the GBD super-regions, Southeast Asia, East Asia, and Oceania (11.40 [9.31, 13.65]) and Sub-Saharan Africa (8.45 [7.17, 9.68]) had the highest EC ASDRs, more than four times higher than the ASDR of North Africa and the Middle East (2.11 [1.81, 2.36]). Southeast Asia, East Asia, and Oceania also had the highest number of EC deaths, 318,545 [259,203, 384,006] in 2021. In Southeast Asia, East Asia, and Oceania, smoking had a significantly higher proportional contribution (5.18 [3.85, 6.71], with a percentage of 45.4%) to the EC ASDR compared to the other three risk factors, high alcohol use (1.73 [1.18, 2.40], 15.1%), diet low in vegetables (0.49 [−0.09, 1.23], 4.3%), and chewing tobacco (0.12 [0.07, 0.18], 1.1%). As for Sub-Saharan Africa, diet low in vegetables was the leading contributor to attributable EC ASDR, accounting for 24.5%, which surpassed the contributions of high alcohol use (10.5%), smoking (9.7%), and chewing tobacco (2.6%) (Fig. 5A and B; Table 2).

Among the four identified risk factors, smoking remained the leading contributor to attributable EC ASDALYR globally and across all GBD super-regions except South Asia and Sub-Saharan Africa, with the highest impact observed in Southeast Asia, East Asia, and Oceania in 2021. In South Asia, however, chewing tobacco and low vegetable intake contributed more to ASDALYR than smoking, while in Sub-Saharan Africa, low vegetable intake and alcohol use surpassed smoking as the primary risk factors for ASDALYR. Notably, alcohol use in Sub-Saharan Africa has increased its contribution to ASDALYR since 1990, marking the only instance of rising attributable ASDALYR in the GBD super-regions. Historically, a low-vegetable diet significantly contributed to ASDALYR in Southeast Asia, East Asia, and Oceania, but this rate declined by over 90% by 2021, allowing alcohol use to become the second leading contributor to ASDALYR (after smoking) in these regions. As for SDI quintiles, a low-vegetable diet remained the top contributor to ASDALYR in low-middle and low SDI regions, whereas smoking dominated in all other quintiles, particularly in high-middle and middle SDI regions. Except for the increase in alcohol use’s contribution to ASDALYR in low-middle SDI regions from 1990 to 2021, other risk factors’ contributions to ASDALYR have declined across the SDI quintiles, with significant improvements in low-vegetable diet and smoking observed in high-middle and middle SDI regions (Fig. 5A, eTable S11-13). However, these regions continue to bear a substantial EC ASR burden (Table 2). The contributions of smoking and high alcohol consumption to EC ASDRs were especially noted among males, whose 45.4% and 32.3% of EC ASDRs in the high-middle SDI region, and 39.6% and 35.9% in the middle SDI region, were attributable to smoking and high alcohol use, respectively (Fig. 5B). Targeted interventions should be prioritized in these regions in an efficient way, especially among males, to mitigate the EC burden effectively.

In the GBD regions, Central Asia has the highest reduction proportions in EC ASRs. In 1990, the attributable EC ASDR in Central Asia was 3.37 [2.66, 4.10] to smoking, 2.31 [−0.50, 4.76] to diet low in vegetables, 1.34 [0.85, 1.85] to alcohol use, and 0.17 [0.11, 0.25] to chewing tobacco. Between 1990 and 2021, the change ratios for various risk factors in Central Asia were − 86.08% for diet low in vegetables, −65.60% for alcohol use, −65.26% for chewing tobacco, and − 64.05% for smoking. The high percentage of alleviation in leading risk factors, especially diet low in vegetables and smoking, contributed to the huge reduction of EC burden in Central Asia, which provided valuable lesson for other regions, especially in Sub-Saharan Africa as one of the regions with highest EC ASRs. In 2021, attributable EC ASDR burden in Sub-Saharan Africa was 0.88 [0.58, 1.18] to alcohol use, 2.08 [−0.42, 3.98] to diet low in vegetables, 0.82 [0.63, 1.04] to smoking, and 0.22 [0.14, 0.30] to chewing tobacco, with percentage changes of + 5.73%, −8.82%, −31.78%, and − 14.12% in 1990–2021, respectively, which were insufficient relative to the high baseline burden in the region.

Distinct patterns of risk factors were observed between males and females, with variations across age groups and regional SDI levels. Globally, smoking was the leading contributor to male EC ASDALY in 2021, while in females, a low-vegetable diet was the most significant contributor. In high SDI regions, smoking has a more pronounced effect on female ASDALY compared to other regions and was the primary contributing factor, with chewing tobacco contributing minimally. In high-middle SDI regions, smoking was the top ASDALY contributor for both males and females, particularly for male EC ASDALYR, where its effect surpassed that in any other SDI quintile. Alcohol use also contributed most to male ASDALYR in these regions. In middle SDI regions, a low-vegetable diet was the leading contributor to female EC ASDALY, while smoking remained dominant in male ASDALY. In low-middle SDI regions, smoking and a low-vegetable diet were the primary contributors to male ASDALY, though smoking’s contribution was more than three times lower than in middle SDI regions. For females in the same regions, a low-vegetable diet and chewing tobacco were the major contributors, with chewing tobacco having a greater effect than in any other SDI quintile in 2021. In low SDI regions, a low-vegetable diet emerged as the most significant risk factor for both sexes (Fig. 5C, eTable S11-13). Globally, smoking contributed the highest ASDR among men over the age of 55. Conversely, in all five age groups, a low-vegetable diet consistently contributed the most to female ASDALYR. Chewing tobacco’s contribution was relatively small across all age groups for males, whereas for females, its effect was similar to that of smoking and a low-vegetable diet, especially in the 20–54 age group, where the contributions of the three risk factors were very close, in contrast to that of alcohol use (Fig. 5D, eTable S11-13).

Notably, in certain regions, especially in Sub-Saharan Africa, North Africa and Middle East, a substantial proportion of the attributable EC burden stems from currently unaccounted factors beyond the four risk factors included in the analysis (Fig. 5B). These unaccounted burdens, described as risk-deleted factors in Fig. 5B, were particularly pronounced among females, both in 1990 and 2021. In 2021, 70.8% of the EC ASDR among females globally remained unattributed, compared to only 19.8% for males. Across all GBD super-regions and SDI quintiles, the proportions of unattributed ASDR exceeded 40% for females and showed an increase in 2021 compared to proportions in 1990 (Fig. 5B). This trend may reflect, on one hand, progress in controlling exposure to the four identified risk factors included in GBD 2021 study, while on the other hand, it underscores the possibility that numerous unexamined risk factors are playing an increasingly critical role in shaping the EC ASDR globally, especially in females and for Sub-Saharan Africa, North Africa, and the Middle East.

### Driver of trends in EC death changes

Figure 5E demonstrates the result of decomposition analysis for the temporal change in EC death cases in the world and regions of different SDI quintiles, where the population size, aging, risk factor changes, and risk-deleted factor were considered as four possible driving factors. In terms of the contributions to the number of deaths, among four driving factors, population growth accounted for an increase of 136,096 EC deaths (percentage of change: 65.14%) in 1990–2021, while aging population contributed to a decrease of 67,655 deaths (32.38%), and changes of risk factors led to a reduction of 78,904 deaths (−37.76%). The risk-deleted effect, considering the treatment advances and other possible risk factors not included in GBD 2021, contributed the 39,509 reduction of EC deaths (−18.91%). For males, population growth and aging increased EC deaths by 114,285 (66.54%) and 63,784 (37.14%), whereas effects from risk factor and risk-deleted factor decreased them by 36,173.35 (−21.06%) and 55,289 (−32.19%) respectively. For females, changes of risk factors reduced EC deaths by 16,444 (−44.22%), and risk-deleted factors lowered deaths by 16,007 (−43.05%), while population growth and aging increased EC deaths by 21,003 (56.48%) and 10,181 (27.38%), respectively.

In low SDI regions, both males and females saw decreases in EC deaths, −929 (−21.44%) in males and − 933 (−38.71%) in females, owing to the changes of risk factors, with population growth contributing significantly to increased mortality numbers, + 5249 (121.17%) in males and + 2956 (122.62%) in females. Compared to the population growth, population aging contributed to the EC death increase with limited numbers and proportions, −549 (−12.67%) in males and − 77 (−3.19%) in females, in 1990–2021. In contrast, low-middle SDI regions exhibited mixed effects where risk factors continue to reduce EC deaths for females, −1,460 (−32.85%), which was approximately counterbalanced by EC deaths changes related to population aging, + 1,331 (29.94%), while males experienced minor increases, + 1,008 (10.51%) because of changing risk factors, with population aging contributing + 1,880 (19.59%) of EC death increase. Similarly in low-SDI regions, the population growth, as the most obvious driving factor, contributed to EC deaths by + 9,493 (98.96%) and + 4,516 (101.62%) in low-middle SDI region. For middle SDI regions, there were notable negative effects, −9,720 (−71.13%) from changing risk factors and − 8,216 (−60.12%) from risk-deleted effects, on female mortality, but males faced more threat of mortality from population growth, −8,216 (−60.12%), and aging, + 37,723 (54.44%), with less mitigation from risk reductions, −31,777 (−45.86%), and risk-deleted effect, −21,728 (−31.36%). This led to the huge differences between male and female EC death trends in 1990–2021 (male: +32,384 by 46.74%, female: −4,209 by −30.8%). High-middle SDI regions showed a similar pattern to middle-SDI regions, with males experiencing increasing EC deaths, + 27,710 (47.68%), largely influenced by aging, + 28,141 (48.42%), and population increases, + 26,988 (46.44%), whereas females benefited more significantly from risk-deleted effects, −6,771 (−69.25%), and risk reductions, −3,144 (−32.16%), which led to the overall reduction of EC deaths, −3,970 (−40.61%). In high-SDI regions, both males and females showed overall increases in EC deaths (males: +13,558, by 44.67%; females: +1,363, by 19.85%). However, females in high-SDI regions achieved more reduced EC deaths from risk factor modification, −1,295 (−18.86%), while risk factors contributed to increased EC deaths, + 3,954 (13.03%), in males. Meanwhile, although males achieved more reduction in EC mortality by risk-deleted effects, −17,385 by −57.28%, than females, only − 2,075 by −30.21%, males had more EC deaths due to aging than females (males: +14,356 by 47.3%; females: +2,465 by 35.88%) and population growth (males: +12,634 by 41.63%; females: +2,269 by 33.03%), which is why the males still faced more overall increase, + 2,465 by 35.88%, in EC deaths than females, + 1,363 by 19.85%. Overall, changes in risk factors tend to exert a mitigating effect on mortality across most SDI groups, except for males in low-middle and high SDI regions, while aging and population dynamics contributed substantially to increased death rates, particularly at lower SDI levels.

Among the four individual risk factors, the overall differences of risk-specific deaths in 1990–2021 were − 17514.63 (−23.52%), 69012.58 (50.58%), 29603.26 (57.34%), and 8214.19 (84.46%) for diet low in vegetables, smoking, alcohol use, and chewing tobacco, respectively, where the aging and growth of global population were increasing the numbers greatly, and the changes of risk effects themselves were decreasing the death numbers, with effects of −18878.05 (−25.36%), −65388.39 (−47.92%), −31071.58 (−60.19%), and − 6400.61 (−65.81%) for diet low in vegetables, smoking, alcohol use, and chewing tobacco, respectively. The risk-deleted effect of smoking and high alcohol use played limited roles in temporal changes, contributing only − 4,272 (−3.13%) and 8,437 (16.34%), respectively, in EC death changes. However, the risk-deleted effects of diet low in vegetables and chewing tobaccos were obvious contributors, with contrary roles where diet low in vegetables-deleted effect served a protective role decreasing 56,165 EC deaths, while chewing tobacco-deleted effect was a harmful factor that contributed 3,800 EC deaths. These suggest the dominant driving roles of smoking and alcohol use in the trend of EC burden, as well as the increased risks caused by population growth and aging.

### The projections of global EC burdens in 2022–2035

**Fig. 6:**
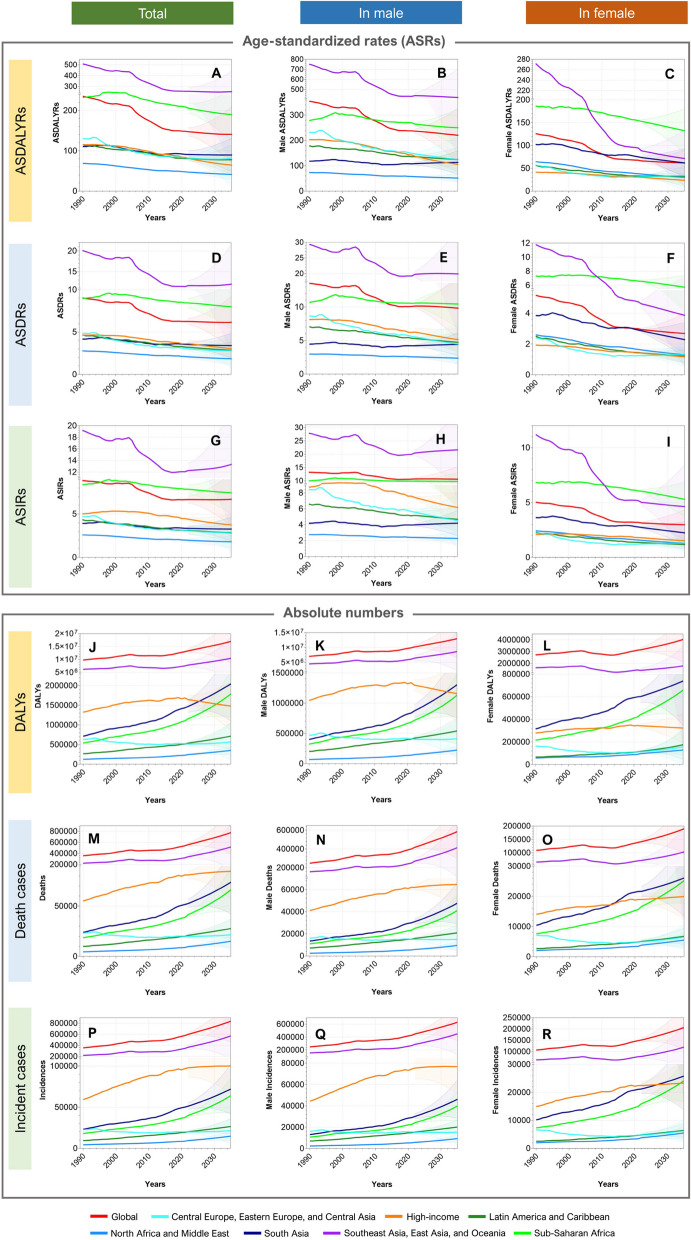
The BAPC forecast of both, male, and female esophageal cancer burdens in world and the seven GBD super-regions. The ASDALYRs of both sexes (**A**), males (**B**), and females (**C**), the ASDRs of both sexes (**D**), males (**E**), and females (**F**), the ASIRs of both sexes (**G**), males (**H**), and females (**I**) in the world and seven super-regions. The DALY numbers of both sexes (**J**), males (**K**), and females (**L**), the death cases of both sexes (**M**), males (**N**), and females (**O**), the incident cases of both sexes (**P**), males (**Q**), and females (**R**) in the world and seven super-regions, where both historical data in 1990–2021 and the BAPC projections in 2022–2035 were plotted with 95% UI

Figure 6A further presents the projected EC ASR burdens by BAPC. From 2021 to 2035, the global ASDALYR for EC is projected to decrease modestly by 5.2%, from 148.56 (the UIs of baseline burdens in 2021 are omitted here, since the UIs provided for 2035 were based on BAPC principles, while the UIs in 2021 were calculated by GBD 2021 study with different principles mentioned in Methods) in 2021 to 140.82 [71.71, 209.94] in 2035 (Fig. 6A and C, eTable S14). During the same period, ASIR is expected to change slightly from 6.65 in 2021 to 6.69 [3.65, 9.73] in 2035 (Fig. 6G and I, eTable S14), while ASDR is anticipated to decline from 6.25 in 2021 to 6.14 [3.10, 9.19] in 2035 (Fig. 6D and F, eTable S14). The reduction in ASRs among males is generally less pronounced than in females, particularly in certain regions. For instance, in Southeast Asia, East Asia, and Oceania, significant declines in ASRs are observed among females, with no comparable improvement in males (Fig. 6B, E-F and H-I, eTable S14).

From 2021 to 2035, the most notable reductions in male ASDALYRs are expected in high-income regions (−26.10%. 105.73 [63.33, 148.13] in 2035), Central Europe, Eastern Europe, and Central Asia (−16.66%, 124.30 [33.43, 215.17] in 2035), and North Africa and the Middle East (−10.95%, 51.54 [35.41, 67.67] in 2035), whereas an increase of 5.28% is projected in South Asia (113.25 [67.34, 159.16] in 2035) (Fig. 6B, eTable S14). For females, the greatest declines in ASDALYRs are anticipated in Southeast Asia, East Asia, and Oceania (−24.36%, 71.59 [10.44, 132.74] in 2035), high-income regions (−22.20%, 23.89 [14.28, 33.50] in 2035), and North Africa and the Middle East (−20.55%, 30.19 [20.73, 39.64] in 2035), with a 9.18% increase expected in Central Europe, Eastern Europe, and Central Asia (33.98 [1.72, 66.24] in 2035) (Fig. 6C, eTable S14). In terms of ASDR, the most significant reductions for males are forecasted in Central Europe, Eastern Europe, and Central Asia (−20.67%, 4.45 [1.42, 7.48] in 2035), high-income regions (−17.46%, 5.14 [3.46, 6.82] in 2035), and Latin America and the Caribbean (−11.27%, 4.74 [3.27, 6.21] in 2035), while increases are expected in Southeast Asia, East Asia, and Oceania (3.36%, 19.88 [5.61, 34.14] in 2035) and South Asia (4.76%, 4.41 [2.74, 6.08] in 2035) (Fig. 6E, eTable S14). Among females, ASDR is projected to decline notably in North Africa and the Middle East (−21.32%, 1.30 [0.79, 1.81] in 2035) and South Asia (−21.23%, 2.29 [1.20, 3.38] in 2035), while Central Europe, Eastern Europe, and Central Asia are the only regions where an increase (5.26%, 1.31 [0.54, 2.08] in 2035) is anticipated (Fig. 6F, eTable S14). Regarding ASIR, the most substantial decreases among males are expected in Central Europe, Eastern Europe, and Central Asia (−20.13%, 4.53 [1.26, 7.80] in 2035) and high-income regions (−18.57%, 6.18 [4.30, 8.06] in 2035), while increases are projected in South Asia (5.87%, 4.20 [2.61, 5.80] in 2035) and Southeast Asia, East Asia, and Oceania (8.57%, 21.64 [6.80, 36.48] in 2035) (Fig. 6H, eTable S14). Among females, ASIR is anticipated to decline in South Asia (−19.41%. 2.24 [1.22, 3.26] in 2035), North Africa and the Middle East (−18.68%, 1.28 [0.77, 1.80] in 2035), and high-income regions (−15.98%, 1.51 [0.97, 2.05] in 2035), while an increase of 7.54% is expected among females in Central Europe, Eastern Europe, and Central Asia (1.31 [0.53, 2.08] in 2035) (Fig. 6I, eTable S14). These findings suggest that females in Central Europe, Eastern Europe, and Central Asia, as well as males in South Asia, are key populations at risk for an increased EC burden. Overall, the global EC burden of ASRs is not expected to see significant alleviation in 2021–2035, and the reduction in ASRs among males is projected to be less pronounced than among females.

Figure 6J further presents the projected absolute EC burden. From 2021 to 2035, the global EC DALY number is projected to increase significantly by approximately 27.3%, from 12,999,265 in 2021 to 16,959,947 [8,662,895, 25,256,999] in 2035 (Fig. 6J, eTable S15). During the same period, the global EC incident case number is expected to increase from 576,529 in 2021 to 834,407 [455,610, 1,213,205] in 2035, while the death number is anticipated to increase from 538,602 to 773,808 [389,781, 1,157,834] in 2021–2035 (Fig. 6J, eTable S15). Aside from a slight improvement in EC DALY within high-income regions (from 1,683,834 in 2021 to 1,480,322 [872,803, 2,087,842] in 2035), the absolute EC burdens are expected to rise globally and across other GBD super-regions in 2021–2035 (Fig. 6J, eTable S15). From 2022 to 2035, Sub-Saharan Africa is predicted to experience the most significant rise in male DALY numbers, at 58.29% (1,130,906 [781,567, 1,480,245] in 2035), followed by South Asia at 49.84% (1,303,889 [752,009, 1,855,768] in 2035) and North Africa and the Middle East at 49.48% (222,640 [151,485, 293,796] in 2035) (Fig. 6K, eTable S15). For females, the largest increases in DALY numbers are expected in Latin America and the Caribbean (50.98%, 176,003 [43,872, 308,134] in 2035), Sub-Saharan Africa (48.90%, 658,511 [418,926, 898,097] in 2035), and Central Europe, Eastern Europe, and Central Asia (37.70%, 157,353 [0, 325545] in 2035) (Fig. 6L, eTable S15). In contrast, high-income regions are projected to see declines, with DALYs for males decreasing by 10.86% (1,157,424 [682,245, 1,632,602] in 2035) and for females by 6.04% (322,899 [190,558, 455,239] in 2035). As for mortality numbers, the most pronounced increases among males are anticipated in Sub-Saharan Africa (by 64.41%, 40,890 [26,664, 55,115] in 2035), North Africa and the Middle East (62.86%, 9,526 [5,974, 13,078] in 2035), Southeast Asia, East Asia, and Oceania (53.98%, 413,486 [100,377, 726,621] in 2035), and South Asia (53.89%, 584,160 [295,222, 873,098] in 2035) (Fig. 6N, eTable S15). For females, notable rises in death numbers are expected in Sub-Saharan Africa (51.33%, 25,214 [17,646, 32,782] in 2035), Southeast Asia, East Asia, and Oceania (45.90%, 103,455 [6,982, 199,943] in 2035), and North Africa and the Middle East (43.15%, 5,516 [3,214, 7,820] in 2035) (Fig. 6O, eTable S15). With regard to incidence numbers, the steepest climbs among males are projected in Sub-Saharan Africa (65.32%, 39,885 [26,015, 53,756] in 2035), North Africa and the Middle East (64.30%, 9,348 [5,845, 12,851] in 2035), and Southeast Asia, East Asia, and Oceania (57.21%, 450,380 [121,133, 779,657] in 2035) (Fig. 6Q, eTable S15). Among females, incidence numbers are anticipated to increase greatly in Southeast Asia, East Asia, and Oceania (52.74%, 119,820 [15,899, 223,754] in 2035), Sub-Saharan Africa (51.74%, 24,085 [16,770, 31,399] in 2035), and North Africa and the Middle East (44.82%, 5,482 [3,171, 7,794] in 2035) (Fig. 6R, eTable S15).

## Discussion

This study analyzed the epidemiological characteristics of EC and forecasted future trends using updated data from GBD 2021. It explored the potential effect of the COVID-19 pandemic on global EC diagnoses and treatments, and highlighted the significant changes in the EC burden and its associated risk factors from 1990 to 2021 on both global and regional scales. The study also emphasized the inequities in EC burdens across regions, nations, sexes, and age groups, and projected future trends in the burden of esophageal cancer at both global and regional levels.

EC is commonly perceived as a disease linked to poverty [35–51]; deep-rooted regional and national inequalities are evident in the EC burden and its changing trends. The geographical pattern of the global EC burden distribution, characterized by the Asian Esophageal Cancer Belt and the African Esophageal Cancer Corridor [52–54], remained evident in 2021, with many countries exhibiting high EC ASRs located within these regions. The high prevalence of EC in East Asia and Sub-Saharan Africa, which is indicated in our results, can be attributed to a complex interplay of environmental, genetic, and lifestyle factors [55, 56]. In East Asia, the “Esophageal Cancer Belt” encompasses countries such as China, Mongolia, and portions of Central Asia [53], where traditional dietary patterns exert a considerable influence, with environmental exposures being an underlying factor. The consumption of foods and beverages prepared at high temperatures, salted or preserved meats, and leftover vegetables has been linked to an elevated risk of EC, especially ESCCs that are of high prevalence in these locations, partly because of exposure to nitrosamines and other chemical carcinogens [1, 57]. Indoor air pollution from the use of biomass fuels and limited socioeconomic resources also serve to exacerbate the risks, by restricting access to healthier alternatives and preventive healthcare [1]. Genetic predisposition can also amplify these risks, as evidenced by the familial clustering observed in high-risk areas.

The interaction between genetic susceptibility and environmental exposures is likely to contribute to epigenetic alterations that drive disease progression [1]. In Sub-Saharan Africa where ESCCs also predominate, the primary contributors to EC are alcohol consumption, tobacco use, and exposure to indoor air pollution from biomass fuels. High alcohol consumption and tobacco use initially failed to account for the differences in EC burden between the Eastern African corridor and the rest of Africa [35]. However, an increasing body of evidence is now providing more reliable attribution of the EC burden in these locations to alcohol and tobacco use [58]. For example, in 2022, results from a case-control study conducted in Kenya, Tanzania, and Malawi revealed the role of alcohol use as a substantial contributor to ESCC risk in East Africa, particularly among men, which could be prevented by cessation or reduction of alcohol consumption [59]. Moreover, an increasing number of studies have found a role of indoor air pollution in the occurrence of African EC, probably due to the biomass smoke exposures [60, 61]. Poor oral hygiene, the consumption of beverages prepared at high temperatures, and nutritional deficiencies contribute to the complexity of the EC etiology in these African countries [1, 62, 63].

Although there are similarities between East Asia and Sub-Saharan Africa, such as the role of environmental carcinogens and lifestyle risks, there are also notable differences. In East Asia, dietary habits and genetic factors play a more prominent role [64, 65], whereas in Sub-Saharan Africa, alcohol and tobacco use are the primary contributors to the risk landscape [1, 62, 63]. Both regions face challenges posed by limited medical infrastructure and socioeconomic barriers, which hinder early detection and intervention. To address these issues, targeted public health strategies, international collaboration, and an emphasis on reducing risk exposures, improving nutrition, and enhancing early screening programmers are required. However, the rising EC incidence number in Sub-Saharan region should also be viewed as a possibly positive signal, as diagnostic and monitoring capabilities in some African countries have improved over the past few decades, resulting in more cases being documented [66]. In other regions, the limited improvement of the EC ASR burden in regions such as high-income North America during recent years is noteworthy, which may highlight the significant role of EAC in these areas [1, 3]. Risk factors for EAC, such as acid or bile reflux, Barrett’s esophagus, and central or visceral obesity, appear to be closely associated with genetic and metabolic factors, which seem more resistant to intervention or modification than the well-recognized risk factors for ESCC, such as tobacco smoking, alcohol use, and thermal injury [34, 67]. Considering the above circumstances, the most important EC primary prevention measures currently focus on two key aspects. First, in high-income regions, there is a need to strengthen lifestyle interventions, emphasize obesity control, and prioritize follow-up for Barrett’s esophagus and effective management of gastroesophageal reflux disease (GERD) [68]. Second, it is essential to continue community-based interventions aimed at reducing smoking and alcohol prevalence, especially for sociodemographically disadvantaged locations [69, 70].

Over the past three decades, significant reductions in the EC burden have been observed in East Asia and Central Asia. However, in recent years, the decline in the EC burden in East Asia has become less marked, which is a notable phenomenon [65]. Factors related to human behavior and consumption habits have exhibited clear global convergence in recent years, causing a “Westernization” of risk factors in Eastern societies [55]. This suggests that in regions like East Asia, a timely shift in focus based on current risk factors and disease burden is necessary to achieve further reductions [65]. In terms of absolute numbers, the EC burdens are particularly heavy in China, India, the United States, Japan, and Brazil, with China being the most prominent. A study comparing the clinical characteristics of EC patients in China and the United States has highlighted notable disparities between the two countries [71]. For instance, the percentage of EC cases receiving surgery in China was significantly higher than in the US (53.7% vs. 22.6%); ESCC accounts for the majority of EC cases in China (85.5%), whereas EAC is more prevalent in the United States (58.9%) [71]. These important differences highlight the necessity for tailored, context-specific prevention, diagnosis, and treatment strategies. Countries need to develop measures suited to their own patient profiles, emphasizing the importance of collaboration between epidemiologists, policymakers, and clinicians to obtain a true and comprehensive understanding of patient characteristics.

As the country with the largest absolute burden of EC, China has made significant strides in screening and treatment over the past two decades [71–73]. The development of socioeconomic and medical technologies, alongside primary prevention measures targeting key risk factors, has played a significant role in reducing the EC burden in China [72, 73]. However, what may offer more valuable lessons to the world is the widespread, intensive, resource-sensitive, and regionally tailored endoscopic screening implemented in China [73–75], which is informed by extensive researches in China on the EC screening and early detection approaches, as well as their clinical and epidemiological cost-effectiveness [43, 72, 76, 77]. In 2006, the Chinese government initiated National Cancer Control Programs including EC screening in counties of four provinces in central China to improve the rate of early detection and early treatment, and to inform cancer screening strategies for the Chinese rural population, which were designed and implemented by China National Cancer Center [72]. This approach has made the timely diagnosis of precancerous lesions and early-stage cancers more common [72]. Moreover, with advancements in artificial intelligence [78–80], there is vast potential for further expanding the effectiveness and reach of such screening efforts. Based on well-established frameworks and emerging technologies, EC screening in China takes full account of regional and population factors, ensuring high efficiency and detection rates. In rural Hua county, Henan Province, a high-incidence area of EC in China, a carefully conducted prospective randomized clinical trial on EC revealed that baseline endoscopic EC screening achieved an early detection rate of 69.9% [81]. The practice of precision EC screening has benefited from the development and utilization of risk-stratification/identification/prediction models integrating the demographic information, family history, smoking status, alarm symptoms, dietary habits, and related disease history, such as peptic ulcer or esophagitis [43], since these models serve as important pre-screening tool with low costs for mass ESCC screening in China to identify a limited group of high-risk individuals who are most likely to benefit from EC endoscopic screening.

Meanwhile, other factors, such as optimal starting age [82], low-cost techniques in resource-limited settings [83], and potential for joint screening [84] were also clearly investigated in high-risk regions and populations, which can improve the cost-effectiveness of the screening plans. China has also made significant progress in raising public awareness of EC prevention and control [85]. The Chinese Anti-Cancer Association (CACA) and the Chinese Society of Clinical Oncology (CSCO) have established dedicated committees to promote cancer prevention and treatment by initiatives like the annual Anti-Cancer Week and the use of various media platforms to educate the public on healthy lifestyles and the importance of early detection and standardized treatment [85]. Chinese health officers and administrators have also made great efforts on the standardization and uniformity of EC screening, diagnosis, and care across different Chinese regions. In China, starting from 2012, the National Health Committee of China led the establishment of the National Cancer Quality Control Center to implement quality control of cancer diagnosis and treatment for a standardized EC diagnosis and treatment, which has covered all key areas in EC diagnosis and treatment, such as medical oncology, radiation oncology, endoscopy, and pathology [86]. This effort also serves as an important contributor to the improvement of overall survival and quality of life of patients with EC across China. However, regions must tailor these efforts according to the local resources and cost-effectiveness. A Korean retrospective population-based study using the Korean National Cancer Screening Program database reported that the workload of endoscopists increased excessively with the rising number of endoscopies, reflected by the decreased EC detection rates during 2015–2019 [87]. The cost-effectiveness analysis for the ESECC trial in China suggested that precision EC screening strategy based on risk stratification presents superior cost-effectiveness compared with traditional screening strategy. This highlights the importance of risk prediction model in pre-screening settings, a valuable lesson for other locations to learn [88].

Overall, the threat of EC is notably less for women than for men, and in recent years, the EC burden in women has shown greater improvement than in men. This phenomenon is observed in most gastrointestinal cancers and has a biological basis, which is generally believed to be associated with differences in sex steroid hormones [65, 89–92]. Among males, although there is a decline in the disease burden for those under the age of 75, the older population has been confronted with an increasingly severe burden of EC in recent decades. The diagnosis and treatment of EC in older adults present highly complex clinical challenges [93]. Although EC is typically considered a disease closely associated with advanced age, older patients are often excluded from major clinical trials [4, 93]. Even when the disease is in a potentially curable stage, it appears that only 50–69% of older patients with EC received effective treatment, despite various therapeutic modalities offering greater survival benefits than best supportive care alone [93]. In decisions to forego treatment, both physicians and patients seem to play an equally dominant role [94], reflecting limited confidence and willingness from both parties towards treatment. There is a pressing need to extend the coverage of clinical trials to include older populations and to conduct more comprehensive cost-benefit analyses of treatment options for this demographic. This would enhance the evidence-based medicine framework in clinical practice and bolster patients’ willingness to pursue treatment. Additionally, there should be a stronger emphasis on multidisciplinary collaboration among anesthesiology, radiotherapy, medical oncology, and critical care specialties to diversify treatment modalities and accumulate practical experience in managing older patients; this has been successfully practiced in China, where the concept of multidisciplinary teams (MDTs) has emerged as a fundamental principle in EC treatment [85]. Furthermore, perioperative support should be reinforced to elevate the status of surgical and neoadjuvant therapies in the current treatment paradigm for older patients with EC [48].

Among the factors contributing to death or the loss of healthy life years due to EC, there are many modifiable risks. Although the GBD 2021 study included only four risk factors—alcohol use, smoking, chewing tobacco, and low vegetable intake—these risk factors can be significantly reduced through intervention. As revealed in our decomposition analysis, improvements in these four risk factors have resulted in a global reduction of 78,904 EC-related deaths (−37.76%), with the decrease being particularly pronounced among males (−16,444 deaths, −44.22%). As in 1990, smoking is still the most important EC risk factor in 2021, contributing most EC ASDALYR. Obvious inequality of smoking-related EC burdens was observed across the world. In the Southeast Asia, East Asia, and Oceania region, smoking contributed to 199.1 and 144.3 ASDALYRs in males and females, respectively, more than the total global EC age-standardized rates. The World Health Organization (WHO) has proposed the MPOWER policy package to urge countries worldwide to implement necessary and comprehensive measures to reduce the health risks associated with smoking [95]. Our research suggests that in regions with high-middle and medium SDI, smoking significantly increases the risk of EC in men. It is imperative to enhance EC screening among middle-aged and elderly men in these regions to lower mortality risks. Additionally, efforts to strengthen the implementation of MPOWER measures are crucial in reducing smoking prevalence and minimizing tobacco exposure within the population [95, 96].

Alcohol consumption has contributed to a significant loss in ASDALY among countries with SDI levels above the middle range. Additionally, both alcohol intake and chewing tobacco account for a substantial proportion of attributable EC deaths among women across all age groups. It is crucial to emphasize the health risks associated with alcohol use, particularly among women and in high-middle SDI regions, to mitigate the EC burden. Low vegetable intake has resulted in a higher EC burden in low SDI regions compared to other factors, particularly among women. The loss of healthy life years attributed to this factor can be approximately three times that caused by both smoking and chewing tobacco combined. It is imperative to promote scientifically balanced diets in these regions and to strengthen agricultural and logistical infrastructure to improve access to vegetables. Compared to other factors, the influence of chewing tobacco is relatively minor, yet it contributes notably to ASDALY in populations within low and lower-middle SDI regions. For a long time, the health risks of chewing tobacco have been inadequately researched and poorly understood. In reality, chewing tobacco and other smokeless tobacco forms are far more harmful and addictive than is generally believed [97]. In many communities, chewing tobacco is even considered a traditional remedy for digestive issues, pain alleviation, and stress relief [97]. It is essential to enhance public awareness in these regions about the significant health hazards posed by chewing tobacco, ensuring that its harmful effects and the risks of prolonged use are well understood. Overall, aside from low vegetable intake, the critical role of addictive factors—such as tobacco and alcohol consumption—in the EC burden should be thoroughly considered. In the future, it will be essential to examine the link between exposure to these substances and genetic susceptibility [98, 99], enabling more targeted EC screening efforts.

Although EC ASRs have decreased from their level in the 1990s, increases in absolute incident and death cases, as well as the active life loss described by DALY numbers, were observed in this work. In line with existing literature [23], the BAPC projections further demonstrated that although the global EC ASRs in the next decade slightly improved, the absolute numbers of EC burden were increasing globally and in most GBD super-regions. Compared with those among females, the reductions of EC ASRs among males are projected to be generally less significant, particularly in certain regions such as Southeast Asia, East Asia, and Oceania. The possible reason for this phenomenon is the different relative magnitudes of risk factor reductions and improvements of risk-deleted factors, including the treatment advances and the decreased effect from other latent risk-EC pairs, compared to the adverse effect from population-related factors [24, 100] (the supplementary decomposition result shown in eFigure S3). However, there remained a huge proportion of female EC burden not attributable to established risk-EC pairs in GBD 2021. Currently, prevention strategies aimed at reducing the EC incidence, along with advancements in screening and treatment technologies to mitigate adverse outcomes, are of paramount importance. Leveraging recent developments in areas such as liquid biopsy [101] and deep learning [80] is crucial to enhancing the quality of screening and treatment, ultimately reducing the global EC burden.

Our study found that during 2020–2021, certain countries exhibited notable deviations in EC burden metrics from their historical linear trends. These deviations may reflect the multifaceted effects of the COVID-19 pandemic on EC diagnosis and treatment. The influence on diagnostic rates, likely stemming from disruptions to routine screening programs or an increase in opportunistic diagnoses [11], could be significant in some locations, and these opposing factors complicate the interpretation of trends. Furthermore, maintaining the operational quality of cancer registries during the pandemic posed a critical challenge to accurately estimating EC incidence [16]. The effect on mortality rates could be even more complex [102–111]. On one hand, COVID-19 may have disrupted EC treatment systems, leading to delays or interruptions in regular anti-cancer therapies. Additionally, comorbidities and complications associated with COVID-19 in patients with EC could have increased mortality risks for EC patients [112–115], potentially elevating overall death rates from EC. On the other hand, COVID-19 may have interfered with the accurate attribution of EC-related deaths, since patients with probable EC may have died without receiving adequate pathological confirmation of their diagnosis, or their deaths may have been underreported in overwhelmed and low-resilience public health systems [14]. These factors revealed the intricate interplay of factors affecting EC burden during the global COVID-19 pandemic. For countries with excessively high increases in ASIRs during pandemic era, such as Bolivarian Republic of Venezuela (ASIR-PPRD 9.46%), Grenada (7.68%) and Republic of Belarus (6.39%), they should evaluate whether their pre-pandemic cancer screening efforts were sufficient, as many of these additional diagnoses could be from opportunistic exposures of patients to their healthcare systems [11], which suggests an underlying insufficiency in early cancer screening capacity or efforts before the pandemic. For locations where excessive deaths were reported, such as Grenada (ASDR-PPRD 7.19%) and Republic of Belarus (6.52%), functioning of EC care systems in the pandemic should be thoroughly examined to assess whether there is sufficient health system resilience and capacity to withstand potential future waves and other challenges. However, for countries with significant declines in incidence and death rates, including Grand Duchy of Luxembourg (ASDR-PPRD − 5.17%) and Republic of Trinidad and Tobago (−5.10%) with great ASDR decreases, as well as Republic of San Marino (ASIR-PPRD − 12.35%), United Arab Emirates (−8.18%), Democratic Socialist Republic of Sri Lanka (−8.05%), Principality of Andorra (−6.82%) and Grand Duchy of Luxembourg (−5.88%) where huge declines in EC incidence were reported, inappropriate or incorrect mortality attributions or instances of under- or misdiagnoses may exist, which probably have led to an underestimation of cancer-related deaths [106]. Overall, we should not only focus on the decline in the functionality of cancer registries during the pandemic but also consider the potential adverse impacts of delayed or reduced quality of clinical diagnosis and treatment on patient outcomes. In this context, factors such as the allocation of healthcare resources and the decline in patients’ functional roles due to social distancing policies also play significant roles [116–118]. Therefore, although our study reveals changes in EC incidence and mortality rates at the national level during 2020–2021, discussions based on specific cultural contexts and sub-national quarantine/lock-down policies, as well as the specific health and economic conditions, still require further subnational-level researches with more follow-up and registry data [14, 106, 119]. Additionally, distinguishing whether excess deaths in the EC population were due to healthcare system disruptions caused by COVID-19 or the increased risk of complications in patients with EC from COVID-19 itself remains an unresolved, or, at least, inadequately disentangled issue [120, 121].

The estimated numbers of EC new and death cases based on GBD methodology were lower than those estimated with the Global Cancer Observatory (GLOBOCAN) 2020 [122], by 65,498 new cases and 32,453 deaths in the total population, respectively. Specifically, GBD estimated 138,806 female and 399,796 male new cases (total incident cases: 538,602), and 148,142 female and 428,387 male deaths (total deaths: 576,529) in 2020, versus 418,350 male and 185,750 female (total: 604,100) new cases and 374,313 male and 169,763 female deaths (total: 544,076) in GLOBOCAN data. These discrepancies also existed in previous GBD and GLOBOCAN rounds [32], mostly because of incomplete data in both datasets and their different modelling. For example, in GLOBOCAN, incidence data were primarily produced by population-based cancer registries (PBCR), while in GBD 2021, MIR conversions were applied in some scenarios to produce the incidence estimates; in GBD study, the weighting of data according to the level of their completeness might assign more weight to data from high-SDI locations, where the incidence data could be more complete and, for EC, the incidence rates were relatively lower than those in low-income countries and territories, as we revealed in this study [43].

This study has certain limitations. First, GBD 2021 considered only four EC risk factors—low vegetable intake, smoking, chewing tobacco, and alcohol use—while excluding other potential factors such as nitrosamine intake, obesity, infectious agents, genetic mutations, consumption of very hot beverage, etc [32]. Second, owing to data limitations, epidemiological metrics for some countries were based on modelling, particularly in nations with lower sociodemographic levels, potentially contributing to variations in data quality. Third, limited access to healthcare services in regions with predominantly low SDI likely hinders adequate screening and accurate diagnosis of EC [44], potentially leading to underreported case numbers, particularly among economically and socially disadvantaged populations. This resource scarcity may have been further exacerbated by the COVID-19 pandemic, as these regions often exhibit weaker health system resilience. Furthermore, as emphasized in our discussion, pandemic-induced delays or cancellations of screening programs, disruptions or inefficiencies in diagnostic and treatment systems, and the dysfunction of cancer registries may introduce significant uncertainties into the recording and estimation of the EC disease burden. Fourth, the GBD database lacks detailed information on pathological types, disease stages, medical treatments, exposure classifications, and cohort specifics, limiting in-depth analysis of EC burden, especially the subgroup analyses focused on ESCC and EAC [123]. Fifth, the relationships between certain risk factors and EC burdens were established and assessed with approaches and principles from BoP studies and WCRF grades, which require support from high-quality epidemiological data and clinical trials. Currently, the heterogeneity among studies still limits the strength of evidence. Although the GBD Study and BoP collaborators has not yet provided an evidence-level evaluation for alcohol consumption in GHDx database, the available data suggest that the risk-outcome (RO) score for smoking is 0.26, with a grade of 3, while chewing tobacco and a diet low in vegetables have RO scores of 0.0051 and 0.0015, respectively, both graded at 2 [24]. This appears to indicate that the evidence linking smoking to EC is of a higher quality. Merely incorporating more trials or observational results is not a suitable way to overcome research heterogeneity. We look forward to more high-quality trial outcomes that can support stronger evidence, enabling more responsible and valuable attributable risk analyses, and reducing the impact of falsified or poor-quality clinical trial data. This will undoubtedly be a challenging yet fascinating and important direction for future research.

## Conclusions

The global EC burden remains substantial, with significant increases in absolute burden numbers expected over the next decade, possibly because of the expanding and aging populations globally. Geographical distribution patterns of EC burden, characterized by the Asian Esophageal Cancer Belt and the African Esophageal Cancer Corridor, call for targeted and in-depth analyses of region-specific risk factors and pathological transitions. Despite the noteworthy progress made, East Asia, particularly China, continues to bear a heavy EC burden. The increasing EC burden among older men is particularly concerning, highlighting the urgent need to enhance the quality of care for this population. Smoking and alcohol consumption remain critical risk factors for EC, but chewing tobacco and low vegetable intake may play significant roles in specific regions, underscoring the importance of region-specific EC prevention strategies. Globally, the high EC burden and slow alleviation are closely tied to lower SDI levels, represented by regions like Sub-Saharan Africa. Moving forward, it is essential to not only conduct higher-quality clinical trials to improve survival outcomes for patients with EC, but also place greater emphasis on precise and comprehensive epidemiological studies to enhance our understanding of EC risk factors. Considering high proportions of EC burden not attributable to established risk factors in GBD 2021 study, as well as the limited positive rate in EC screening, more solid and complete attributions to risk factors for EC burdens and better identification of high-risk populations in specific areas are still greatly needed to inform the targeted prevention and screening. Moreover, harnessing technological advancements, such as artificial intelligence and liquid biopsy, is critical for improving the coverage and accuracy of early EC diagnosis, ultimately helping to reduce the global burden of EC.

## Supplementary Information

Supplementary Material 1.

## Data Availability

The data supporting the findings of this study are openly available in the GBD 2021 results (https://vizhub.healthdata.org/gbd-results/). Reasonable access to all codes that produce secondary results in this study can be obtained by contacting the lead corresponding author (X.D.Y., yuanyxd@163.com).
