# Supplementary material for: Global, regional, and national burden of esophageal cancer: a systematic analysis of the Global Burden of Disease Study 2021
Source: Biomark Res. 2025 Jan 6;13:3. doi: 10.1186/s40364-024-00718-2 (PMC11702276; doi:10.1186/s40364-024-00718-2)
Supplement: Supplementary file 1 — Supplementary Material 1. [file 40364_2024_718_MOESM1_ESM.pdf]

ELECTRICAL SUPPLEMENTS for

Global, Regional, and National Burdens of Esophageal Cancer: A Systematic Analysis of the Global Burden of Disease Study 2021

TABLE OF CONTENTS

Items	Page	Content
**Note S1**	3	An introduction to GBD 2021
**Note S2**	13	Analysis of the EC diagnoses, deaths, and DALY changes in pandemic with Joinpoint regression and PPRD values
**Figure S1**	17	The trends in ASPoD of esophageal cancer globally and across 21 GBD regions by SDI, 1990-2021
**Figure S2**	18	The changing patterns of age-standardized EC incidence, death, and DALY rates of countries and territories in 2020-2021
**Figure S3**	19	Decomposition result for the death changes in seven GBD super-regions during 1990-2021 by sexes
**Table S1**	20	Metrics definitions involved in this study
**Table S2**	21	Location hierarchies involved in this study
**Table S3**	34	SDI values in 1990, 2019, and 2021
**Table S4**	40	SDI quintiles in GBD 2021 version
**Table S5**	46	The ASRs (per 100,000 population) of esophageal cancer deaths, incidence, and DALYs in 1990 worldwide
**Table S6**	51	The ASRs (per 100,000 population) of esophageal cancer deaths, incidence, and DALYs in 2021 worldwide
**Table S7**	56	The percentage change of ASRs (per 100,000 population) of esophageal cancer deaths, incidence, and DALYs from 1990 to 2021 worldwide
**Table S8**	61	The absolute numbers of esophageal cancer deaths, incidence, and DALYs in 1990 worldwide

**Table S9**	69	The absolute numbers of esophageal cancer deaths, incidence, and DALYs in 2021 worldwide
**Table S10**	77	The percentage change of absolute numbers of esophageal cancer deaths, incidence, and DALYs from 1990 to 2021 worldwide
**Table S11**	82	The ASDR and ASDALYR in world and seven GBD super regions, and regions with five SDI quintiles of males, females, and both sexes attributable to four most detailed risks and their proportion of contribution in 1990 and 2021
**Table S12**	126	The death rate (DR) and DALYR of males, females, and both sexes of five age-groups in world and regions with five SDI quintiles attributable to the four most detailed risks in 1990 and 2021
**Table S13**	176	Result of decomposition analysis for the driving factor of EC deaths globally and among SDI quintiles, where all risk factors and individual factors were included. The risk-deleted effect for individual factors demonstrates the influences from both total risk-deleted effect and the effects from other individual risk factors
**Table S14**	181	The BAPC prediction results (predicted values and 95% uncertainty intervals) for ASRs of esophageal cancer in world and seven GBD super regions from 2022 to 2035
**Table S15**	185	The BAPC prediction results (predicted values and 95% uncertainty intervals) for absolute numbers of incidence, deaths and DALYs of esophageal cancer in world and seven GBD super regions from 2022 to 2035
**Table S16**	189	The PPRD values of global neoplasm ASIR in 204 countries for both sexes in 2020 and 2021.
**Table S17**	198	The PPRD values of global neoplasm ASDR in 204 countries for both sexes in 2020 and 2021.
**Table S18**	207	The PPRD values of global neoplasm ASDALYR in 204 countries for both sexes in 2020 and 2021.

Supplementary Note S1 | An introduction to GBD 2021

S1-1 Overview

The Global Burden of Diseases, Injuries, and Risk Factors Study (GBD) provides a systematic scientific assessment of published, publicly available, and contributed data on disease and injury incidence, prevalence, and mortality for a mutually exclusive and collectively exhaustive list of diseases and injuries. Released on May 2023, GBD 2021 produced estimates for 204 countries and territories that were grouped into 21 regions and seven super regions, The seven super-regions are central Europe, eastern Europe, and central Asia; high income; Latin America and the Caribbean; north Africa and the Middle East; south Asia; southeast Asia, east Asia, and Oceania; and sub-Saharan Africa[1,2].

Results from GBD 2021 are available through an interactive data downloading tool on the Global Health Data Exchange (GHDx). The GHDx is the world's most comprehensive catalogue of surveys, censuses, vital statistics, and other health-related data. The latest version of the data download tool, available here: http://ghdx.healthdata.org/GBD-resultstool, contains core summary results for GBD 2021. These results include deaths, years of life lost (YLLs), YLDs, disability-adjusted life-years (DALYs), prevalence, incidence, and rate of change. The GHDx includes data for causes, risks, cause-risk attribution, etiologies, and impairments [1,2].

S1-2 Data input source

The GBD estimation process relies on a comprehensive identification of multiple relevant data sources for each disease or injury. These sources encompass censuses, household surveys, civil registration and vital statistics, disease registries, health service utilization records, air pollution monitors, satellite imaging, disease notifications, and various other datasets. Data are gathered through a review of published studies, searches on government and international organization websites, published reports, primary data sources such as the Demographic and Health Surveys, and contributions from GBD collaborators. An international network of over 10 000 collaborators from more than 150 countries and territories provided, reviewed, or analyzed the available data to generate GBD metrics. This methodology ensures the inclusion of a wide variety of data sources, enhancing the robustness and comprehensiveness of the GBD estimates [1,2].

As with previous GBD rounds, GBD 2021 integrates a large and growing number of data input sources, including surveys, censuses, vital statistics, and other health-related datasets. These sources provide the basis for estimating morbidity, illness, injury, and attributable risk for 204 countries and territories from 1990 to 2021, with mortality data estimated from 1980 to 2021. The compilation of these data sources is facilitated

by an interactive citation tool available on the Global Health Data Exchange (GHDx) platform. Citations for specific GBD components, causes and risks, and locations can be accessed via the Data Input Sources Tool in GHDx: http://ghdx.healthdata.org/gbd/2020/data-input-sources [1,2].

S1-3 Causes organization

The GBD cause and sequelae list is structured hierarchically to address the diverse requirements of various users. This hierarchical organization begins with broad categorizations and progresses to more detailed levels. At Level 1, causes are grouped into three primary categories: communicable, maternal, neonatal, and nutritional diseases (Group 1 diseases); non-communicable diseases (Group 2); and injuries (Group 3). These broad categories are further divided at Level 2 into 22 specific groupings, such as neonatal disorders, neurological disorders, and transport injuries [1,2]. The hierarchy extends to Levels 3 and 4, which provide the most granular detail for causes included in GBD 2021. Certain causes, such as anxiety disorders or rheumatoid arthritis, are detailed at Level 3, while others, like depressive disorders, are classified at Level 3 with further specification at Level 4, encompassing major depressive disorders and dysthymia. Sequelae of diseases and injuries are categorized at Levels 5 and 6. Within the GBD framework, sequelae are defined as distinct, mutually exclusive health consequences that can be directly attributed to a specific cause. This hierarchical organization allows for a comprehensive and detailed understanding of the causes and effects of various health conditions, accommodating the needs of different users and facilitating nuanced analysis and interpretation. Below is the risk factor hierarchy related to this study:

eNote-Table S1-3-1. Risk factor hierarchy related to this study

First-level risks	Second-level risks	Third-level risks	Detailed risks
Behavioral risks	Alcohol use	/	Alcohol use
	Dietary risks	Diet low in vegetables	Diet low in vegetables
	Tobacco	Smoking	Smoking
		Chewing tobacco	Chewing tobacco

eNote-Table S1-3-2. Relative risk estimates of risk factors

(A) Relative risk of esophageal cancer from Chewing tobacco

Risk-Outcome Score	Star rating	Sex	Age group	Location	Log relative risk of outcome							Minimum standard error	Maximum standard error
					lower fixed & random effects uncertainty	lower fixed effects uncertainty	Estimation	upper fixed effects uncertainty	upper fixed & random effects uncertainty	lower funnel boundary	upper funnel boundary	lower boundary of funnel standard error	upper boundary of funnel standard error
0.00514	2 stars	Both	All Ages	Global	-0.12775	0.544462	0.731168	0.917873	1.590083	-1.07831	2.540643	0	0.923202

(B) Relative risk of esophageal cancer from Diet low in vegetables

Risk-Outcome Score	Star rating	Sex	Age group	Location	Log relative risk of outcome					Risk units	Risk
					lower fixed & random effects uncertainty	lower fixed effects uncertainty	Estimation	upper fixed effects uncertainty	upper fixed & random effects uncertainty		
0.001	2 stars	Both	All Ages	Global	0.000	0.000	0.000	0.000	0.000	g/day	0.000
0.001	2 stars	Both	All Ages	Global	-0.011	-0.002	-0.005	-0.008	0.001	g/day	3.853
0.001	2 stars	Both	All Ages	Global	-0.022	-0.003	-0.010	-0.017	0.002	g/day	7.706
0.001	2 stars	Both	All Ages	Global	-0.032	-0.005	-0.015	-0.025	0.003	g/day	11.559
0.001	2 stars	Both	All Ages	Global	-0.042	-0.006	-0.019	-0.033	0.003	g/day	15.412
0.001	2 stars	Both	All Ages	Global	-0.052	-0.008	-0.024	-0.040	0.004	g/day	19.265
0.001	2 stars	Both	All Ages	Global	-0.062	-0.009	-0.028	-0.048	0.005	g/day	23.118
0.001	2 stars	Both	All Ages	Global	-0.071	-0.011	-0.033	-0.055	0.006	g/day	26.971
0.001	2 stars	Both	All Ages	Global	-0.081	-0.012	-0.037	-0.062	0.007	g/day	30.823
0.001	2 stars	Both	All Ages	Global	-0.090	-0.013	-0.041	-0.069	0.007	g/day	34.676
0.001	2 stars	Both	All Ages	Global	-0.098	-0.015	-0.045	-0.076	0.008	g/day	38.529
0.001	2 stars	Both	All Ages	Global	-0.107	-0.016	-0.049	-0.082	0.009	g/day	42.382
0.001	2 stars	Both	All Ages	Global	-0.115	-0.017	-0.053	-0.088	0.009	g/day	46.235
0.001	2 stars	Both	All Ages	Global	-0.123	-0.018	-0.056	-0.094	0.010	g/day	50.088
0.001	2 stars	Both	All Ages	Global	-0.130	-0.019	-0.060	-0.100	0.011	g/day	53.941
0.001	2 stars	Both	All Ages	Global	-0.138	-0.021	-0.063	-0.106	0.011	g/day	57.794
0.001	2 stars	Both	All Ages	Global	-0.145	-0.022	-0.067	-0.111	0.012	g/day	61.647
0.001	2 stars	Both	All Ages	Global	-0.152	-0.023	-0.070	-0.117	0.012	g/day	65.500
0.001	2 stars	Both	All Ages	Global	-0.158	-0.024	-0.073	-0.122	0.013	g/day	69.353
0.001	2 stars	Both	All Ages	Global	-0.164	-0.025	-0.075	-0.126	0.013	g/day	73.206
0.001	2 stars	Both	All Ages	Global	-0.170	-0.025	-0.078	-0.131	0.014	g/day	77.059
0.001	2 stars	Both	All Ages	Global	-0.176	-0.026	-0.081	-0.135	0.014	g/day	80.912
0.001	2 stars	Both	All Ages	Global	-0.181	-0.027	-0.083	-0.140	0.015	g/day	84.765
0.001	2 stars	Both	All Ages	Global	-0.187	-0.028	-0.086	-0.143	0.015	g/day	88.617
0.001	2 stars	Both	All Ages	Global	-0.191	-0.029	-0.088	-0.147	0.016	g/day	92.470
0.001	2 stars	Both	All Ages	Global	-0.196	-0.029	-0.090	-0.151	0.016	g/day	96.323
0.001	2 stars	Both	All Ages	Global	-0.200	-0.030	-0.092	-0.154	0.016	g/day	100.176
0.001	2 stars	Both	All Ages	Global	-0.204	-0.030	-0.094	-0.157	0.017	g/day	104.029
0.001	2 stars	Both	All Ages	Global	-0.208	-0.031	-0.095	-0.160	0.017	g/day	107.882
0.001	2 stars	Both	All Ages	Global	-0.211	-0.031	-0.097	-0.162	0.017	g/day	111.735
0.001	2 stars	Both	All Ages	Global	-0.214	-0.032	-0.098	-0.165	0.018	g/day	115.588
0.001	2 stars	Both	All Ages	Global	-0.217	-0.032	-0.100	-0.167	0.018	g/day	119.441
0.001	2 stars	Both	All Ages	Global	-0.219	-0.033	-0.101	-0.169	0.018	g/day	123.294

0.001	2 stars	Both	All Ages	Global	-0.221	-0.033	-0.102	-0.170	0.018	g/day	127.147
0.001	2 stars	Both	All Ages	Global	-0.223	-0.033	-0.103	-0.172	0.018	g/day	131.000
0.001	2 stars	Both	All Ages	Global	-0.225	-0.034	-0.103	-0.173	0.018	g/day	134.853
0.001	2 stars	Both	All Ages	Global	-0.227	-0.034	-0.104	-0.174	0.019	g/day	138.706
0.001	2 stars	Both	All Ages	Global	-0.228	-0.034	-0.105	-0.175	0.019	g/day	142.559
0.001	2 stars	Both	All Ages	Global	-0.229	-0.034	-0.105	-0.176	0.019	g/day	146.411
0.001	2 stars	Both	All Ages	Global	-0.230	-0.034	-0.106	-0.177	0.019	g/day	150.264
0.001	2 stars	Both	All Ages	Global	-0.232	-0.035	-0.106	-0.178	0.019	g/day	154.117
0.001	2 stars	Both	All Ages	Global	-0.233	-0.035	-0.107	-0.179	0.019	g/day	157.970
0.001	2 stars	Both	All Ages	Global	-0.234	-0.035	-0.107	-0.180	0.019	g/day	161.823
0.001	2 stars	Both	All Ages	Global	-0.236	-0.035	-0.108	-0.182	0.019	g/day	165.676
0.001	2 stars	Both	All Ages	Global	-0.238	-0.036	-0.109	-0.183	0.020	g/day	169.529
0.001	2 stars	Both	All Ages	Global	-0.241	-0.036	-0.111	-0.186	0.020	g/day	173.382
0.001	2 stars	Both	All Ages	Global	-0.245	-0.037	-0.112	-0.188	0.020	g/day	177.235
0.001	2 stars	Both	All Ages	Global	-0.249	-0.037	-0.114	-0.192	0.020	g/day	181.088
0.001	2 stars	Both	All Ages	Global	-0.254	-0.038	-0.117	-0.196	0.021	g/day	184.941
0.001	2 stars	Both	All Ages	Global	-0.260	-0.039	-0.119	-0.200	0.021	g/day	188.794
0.001	2 stars	Both	All Ages	Global	-0.267	-0.040	-0.123	-0.205	0.022	g/day	192.647
0.001	2 stars	Both	All Ages	Global	-0.275	-0.041	-0.126	-0.211	0.022	g/day	196.500
0.001	2 stars	Both	All Ages	Global	-0.283	-0.042	-0.130	-0.218	0.023	g/day	200.353
0.001	2 stars	Both	All Ages	Global	-0.292	-0.044	-0.134	-0.225	0.024	g/day	204.205
0.001	2 stars	Both	All Ages	Global	-0.302	-0.045	-0.139	-0.232	0.025	g/day	208.058
0.001	2 stars	Both	All Ages	Global	-0.313	-0.047	-0.144	-0.241	0.026	g/day	211.911
0.001	2 stars	Both	All Ages	Global	-0.324	-0.048	-0.149	-0.249	0.027	g/day	215.764
0.001	2 stars	Both	All Ages	Global	-0.336	-0.050	-0.154	-0.258	0.028	g/day	219.617
0.001	2 stars	Both	All Ages	Global	-0.348	-0.052	-0.160	-0.268	0.029	g/day	223.470
0.001	2 stars	Both	All Ages	Global	-0.361	-0.054	-0.166	-0.277	0.030	g/day	227.323
0.001	2 stars	Both	All Ages	Global	-0.374	-0.056	-0.171	-0.287	0.031	g/day	231.176
0.001	2 stars	Both	All Ages	Global	-0.387	-0.058	-0.177	-0.297	0.032	g/day	235.029
0.001	2 stars	Both	All Ages	Global	-0.400	-0.060	-0.184	-0.308	0.033	g/day	238.882
0.001	2 stars	Both	All Ages	Global	-0.413	-0.062	-0.190	-0.318	0.034	g/day	242.735
0.001	2 stars	Both	All Ages	Global	-0.427	-0.064	-0.196	-0.328	0.035	g/day	246.588
0.001	2 stars	Both	All Ages	Global	-0.440	-0.066	-0.202	-0.339	0.036	g/day	250.441
0.001	2 stars	Both	All Ages	Global	-0.454	-0.068	-0.208	-0.349	0.037	g/day	254.294
0.001	2 stars	Both	All Ages	Global	-0.467	-0.070	-0.215	-0.359	0.038	g/day	258.147
0.001	2 stars	Both	All Ages	Global	-0.481	-0.072	-0.221	-0.370	0.039	g/day	261.999
0.001	2 stars	Both	All Ages	Global	-0.495	-0.074	-0.227	-0.381	0.041	g/day	265.852
0.001	2 stars	Both	All Ages	Global	-0.509	-0.076	-0.234	-0.391	0.042	g/day	269.705
0.001	2 stars	Both	All Ages	Global	-0.523	-0.078	-0.240	-0.402	0.043	g/day	273.558
0.001	2 stars	Both	All Ages	Global	-0.537	-0.080	-0.246	-0.413	0.044	g/day	277.411
0.001	2 stars	Both	All Ages	Global	-0.551	-0.082	-0.253	-0.424	0.045	g/day	281.264
0.001	2 stars	Both	All Ages	Global	-0.565	-0.084	-0.259	-0.435	0.046	g/day	285.117
0.001	2 stars	Both	All Ages	Global	-0.579	-0.086	-0.266	-0.446	0.047	g/day	288.970

0.001	2 stars	Both	All Ages	Global	-0.594	-0.088	-0.273	-0.457	0.049	g/day	292.823
0.001	2 stars	Both	All Ages	Global	-0.608	-0.091	-0.279	-0.468	0.050	g/day	296.676
0.001	2 stars	Both	All Ages	Global	-0.623	-0.093	-0.286	-0.479	0.051	g/day	300.529
0.001	2 stars	Both	All Ages	Global	-0.637	-0.095	-0.292	-0.490	0.052	g/day	304.382
0.001	2 stars	Both	All Ages	Global	-0.652	-0.097	-0.299	-0.501	0.053	g/day	308.235
0.001	2 stars	Both	All Ages	Global	-0.667	-0.099	-0.306	-0.513	0.055	g/day	312.088
0.001	2 stars	Both	All Ages	Global	-0.681	-0.102	-0.313	-0.524	0.056	g/day	315.941
0.001	2 stars	Both	All Ages	Global	-0.696	-0.104	-0.320	-0.535	0.057	g/day	319.793
0.001	2 stars	Both	All Ages	Global	-0.711	-0.106	-0.327	-0.547	0.058	g/day	323.646
0.001	2 stars	Both	All Ages	Global	-0.726	-0.108	-0.333	-0.559	0.059	g/day	327.499
0.001	2 stars	Both	All Ages	Global	-0.741	-0.111	-0.340	-0.570	0.061	g/day	331.352
0.001	2 stars	Both	All Ages	Global	-0.757	-0.113	-0.347	-0.582	0.062	g/day	335.205
0.001	2 stars	Both	All Ages	Global	-0.772	-0.115	-0.354	-0.594	0.063	g/day	339.058
0.001	2 stars	Both	All Ages	Global	-0.788	-0.117	-0.362	-0.606	0.064	g/day	342.911
0.001	2 stars	Both	All Ages	Global	-0.803	-0.120	-0.369	-0.618	0.066	g/day	346.764
0.001	2 stars	Both	All Ages	Global	-0.819	-0.122	-0.376	-0.630	0.067	g/day	350.617
0.001	2 stars	Both	All Ages	Global	-0.834	-0.124	-0.383	-0.642	0.068	g/day	354.470
0.001	2 stars	Both	All Ages	Global	-0.850	-0.127	-0.390	-0.654	0.070	g/day	358.323
0.001	2 stars	Both	All Ages	Global	-0.866	-0.129	-0.398	-0.666	0.071	g/day	362.176
0.001	2 stars	Both	All Ages	Global	-0.882	-0.131	-0.405	-0.678	0.072	g/day	366.029
0.001	2 stars	Both	All Ages	Global	-0.898	-0.134	-0.412	-0.691	0.074	g/day	369.882
0.001	2 stars	Both	All Ages	Global	-0.915	-0.136	-0.420	-0.703	0.075	g/day	373.735
0.001	2 stars	Both	All Ages	Global	-0.931	-0.139	-0.427	-0.716	0.076	g/day	377.587
0.001	2 stars	Both	All Ages	Global	-0.947	-0.141	-0.435	-0.729	0.078	g/day	381.440

(C) Relative risk of esophageal cancer from smoking

Risk name	Risk-Outcome Score	Star rating	Sex	Age group	Location	Log relative risk of outcome					Risk units	Risk
						lower fixed & random effects uncertainty	lower fixed effects uncertainty	Estimation	upper fixed effects uncertainty	upper fixed & random effects uncertainty		
Smoking	0.257	3 stars	Both	All Ages	Global	0.000	0.000	0.000	0.000	0.000	pack-year	0.000
Smoking	0.257	3 stars	Both	All Ages	Global	0.011	0.042	0.053	0.063	0.094	pack-year	1.136
Smoking	0.257	3 stars	Both	All Ages	Global	0.021	0.082	0.102	0.122	0.183	pack-year	2.273
Smoking	0.257	3 stars	Both	All Ages	Global	0.031	0.120	0.149	0.178	0.268	pack-year	3.409
Smoking	0.257	3 stars	Both	All Ages	Global	0.040	0.157	0.195	0.232	0.349	pack-year	4.545
Smoking	0.257	3 stars	Both	All Ages	Global	0.049	0.192	0.238	0.284	0.426	pack-year	5.682
Smoking	0.257	3 stars	Both	All Ages	Global	0.058	0.226	0.280	0.334	0.501	pack-year	6.818
Smoking	0.257	3 stars	Both	All Ages	Global	0.066	0.258	0.320	0.382	0.574	pack-year	7.955
Smoking	0.257	3 stars	Both	All Ages	Global	0.075	0.290	0.359	0.429	0.644	pack-year	9.091
Smoking	0.257	3 stars	Both	All Ages	Global	0.082	0.320	0.397	0.474	0.712	pack-year	10.227
Smoking	0.257	3 stars	Both	All Ages	Global	0.090	0.350	0.434	0.518	0.778	pack-year	11.364
Smoking	0.257	3 stars	Both	All Ages	Global	0.097	0.379	0.469	0.560	0.841	pack-year	12.500
Smoking	0.257	3 stars	Both	All Ages	Global	0.105	0.406	0.504	0.601	0.903	pack-year	13.636
Smoking	0.257	3 stars	Both	All Ages	Global	0.111	0.433	0.537	0.640	0.962	pack-year	14.773

Smoking	0.257	3 stars	Both	All Ages	Global	0.118	0.459	0.569	0.679	1.019	pack-year	15.909
Smoking	0.257	3 stars	Both	All Ages	Global	0.124	0.483	0.599	0.715	1.074	pack-year	17.045
Smoking	0.257	3 stars	Both	All Ages	Global	0.131	0.507	0.629	0.750	1.127	pack-year	18.182
Smoking	0.257	3 stars	Both	All Ages	Global	0.136	0.530	0.657	0.784	1.178	pack-year	19.318
Smoking	0.257	3 stars	Both	All Ages	Global	0.142	0.552	0.684	0.816	1.226	pack-year	20.455
Smoking	0.257	3 stars	Both	All Ages	Global	0.147	0.572	0.709	0.847	1.272	pack-year	21.591
Smoking	0.257	3 stars	Both	All Ages	Global	0.152	0.592	0.734	0.876	1.315	pack-year	22.727
Smoking	0.257	3 stars	Both	All Ages	Global	0.157	0.610	0.757	0.903	1.356	pack-year	23.864
Smoking	0.257	3 stars	Both	All Ages	Global	0.161	0.628	0.778	0.928	1.394	pack-year	25.000
Smoking	0.257	3 stars	Both	All Ages	Global	0.166	0.643	0.798	0.952	1.430	pack-year	26.136
Smoking	0.257	3 stars	Both	All Ages	Global	0.169	0.658	0.815	0.973	1.461	pack-year	27.273
Smoking	0.257	3 stars	Both	All Ages	Global	0.173	0.671	0.831	0.992	1.490	pack-year	28.409
Smoking	0.257	3 stars	Both	All Ages	Global	0.176	0.684	0.848	1.012	1.519	pack-year	29.545
Smoking	0.257	3 stars	Both	All Ages	Global	0.180	0.699	0.866	1.034	1.553	pack-year	30.682
Smoking	0.257	3 stars	Both	All Ages	Global	0.184	0.716	0.888	1.060	1.592	pack-year	31.818
Smoking	0.257	3 stars	Both	All Ages	Global	0.189	0.736	0.913	1.089	1.636	pack-year	32.955
Smoking	0.257	3 stars	Both	All Ages	Global	0.195	0.759	0.941	1.123	1.687	pack-year	34.091
Smoking	0.257	3 stars	Both	All Ages	Global	0.202	0.785	0.973	1.161	1.744	pack-year	35.227
Smoking	0.257	3 stars	Both	All Ages	Global	0.209	0.813	1.008	1.203	1.806	pack-year	36.364
Smoking	0.257	3 stars	Both	All Ages	Global	0.217	0.843	1.045	1.247	1.874	pack-year	37.500
Smoking	0.257	3 stars	Both	All Ages	Global	0.225	0.876	1.086	1.295	1.946	pack-year	38.636
Smoking	0.257	3 stars	Both	All Ages	Global	0.234	0.910	1.128	1.346	2.022	pack-year	39.773
Smoking	0.257	3 stars	Both	All Ages	Global	0.243	0.946	1.172	1.399	2.101	pack-year	40.909
Smoking	0.257	3 stars	Both	All Ages	Global	0.253	0.983	1.218	1.454	2.184	pack-year	42.045
Smoking	0.257	3 stars	Both	All Ages	Global	0.263	1.021	1.266	1.511	2.269	pack-year	43.182
Smoking	0.257	3 stars	Both	All Ages	Global	0.273	1.060	1.314	1.568	2.356	pack-year	44.318
Smoking	0.257	3 stars	Both	All Ages	Global	0.283	1.100	1.364	1.627	2.445	pack-year	45.455
Smoking	0.257	3 stars	Both	All Ages	Global	0.294	1.141	1.414	1.687	2.534	pack-year	46.591
Smoking	0.257	3 stars	Both	All Ages	Global	0.304	1.181	1.464	1.747	2.625	pack-year	47.727
Smoking	0.257	3 stars	Both	All Ages	Global	0.315	1.222	1.515	1.808	2.716	pack-year	48.864
Smoking	0.257	3 stars	Both	All Ages	Global	0.325	1.263	1.566	1.869	2.807	pack-year	50.000
Smoking	0.257	3 stars	Both	All Ages	Global	0.336	1.304	1.617	1.929	2.898	pack-year	51.136
Smoking	0.257	3 stars	Both	All Ages	Global	0.346	1.345	1.668	1.990	2.989	pack-year	52.273
Smoking	0.257	3 stars	Both	All Ages	Global	0.357	1.386	1.718	2.050	3.079	pack-year	53.409
Smoking	0.257	3 stars	Both	All Ages	Global	0.367	1.426	1.768	2.110	3.169	pack-year	54.545
Smoking	0.257	3 stars	Both	All Ages	Global	0.377	1.466	1.817	2.168	3.257	pack-year	55.682
Smoking	0.257	3 stars	Both	All Ages	Global	0.387	1.505	1.865	2.226	3.343	pack-year	56.818
Smoking	0.257	3 stars	Both	All Ages	Global	0.397	1.542	1.911	2.281	3.426	pack-year	57.955
Smoking	0.257	3 stars	Both	All Ages	Global	0.406	1.577	1.954	2.332	3.503	pack-year	59.091
Smoking	0.257	3 stars	Both	All Ages	Global	0.414	1.609	1.994	2.380	3.575	pack-year	60.227
Smoking	0.257	3 stars	Both	All Ages	Global	0.422	1.639	2.031	2.424	3.641	pack-year	61.364
Smoking	0.257	3 stars	Both	All Ages	Global	0.429	1.666	2.065	2.464	3.701	pack-year	62.500
Smoking	0.257	3 stars	Both	All Ages	Global	0.435	1.691	2.096	2.501	3.757	pack-year	63.636

Smoking	0.257	3 stars	Both	All Ages	Global	0.441	1.714	2.125	2.536	3.809	pack-year	64.773
Smoking	0.257	3 stars	Both	All Ages	Global	0.447	1.736	2.151	2.567	3.856	pack-year	65.909
Smoking	0.257	3 stars	Both	All Ages	Global	0.452	1.755	2.176	2.596	3.900	pack-year	67.045
Smoking	0.257	3 stars	Both	All Ages	Global	0.456	1.773	2.198	2.623	3.940	pack-year	68.182
Smoking	0.257	3 stars	Both	All Ages	Global	0.460	1.790	2.218	2.647	3.976	pack-year	69.318
Smoking	0.257	3 stars	Both	All Ages	Global	0.464	1.805	2.237	2.669	4.009	pack-year	70.455
Smoking	0.257	3 stars	Both	All Ages	Global	0.468	1.818	2.254	2.689	4.040	pack-year	71.591
Smoking	0.257	3 stars	Both	All Ages	Global	0.471	1.830	2.269	2.708	4.067	pack-year	72.727
Smoking	0.257	3 stars	Both	All Ages	Global	0.474	1.841	2.283	2.724	4.091	pack-year	73.864
Smoking	0.257	3 stars	Both	All Ages	Global	0.476	1.851	2.295	2.738	4.113	pack-year	75.000
Smoking	0.257	3 stars	Both	All Ages	Global	0.479	1.860	2.305	2.751	4.132	pack-year	76.136
Smoking	0.257	3 stars	Both	All Ages	Global	0.480	1.867	2.314	2.762	4.148	pack-year	77.273
Smoking	0.257	3 stars	Both	All Ages	Global	0.482	1.873	2.322	2.771	4.162	pack-year	78.409
Smoking	0.257	3 stars	Both	All Ages	Global	0.483	1.879	2.329	2.779	4.174	pack-year	79.545
Smoking	0.257	3 stars	Both	All Ages	Global	0.484	1.883	2.334	2.785	4.183	pack-year	80.682
Smoking	0.257	3 stars	Both	All Ages	Global	0.485	1.886	2.337	2.789	4.189	pack-year	81.818
Smoking	0.257	3 stars	Both	All Ages	Global	0.486	1.888	2.340	2.792	4.194	pack-year	82.955
Smoking	0.257	3 stars	Both	All Ages	Global	0.486	1.889	2.341	2.794	4.196	pack-year	84.091
Smoking	0.257	3 stars	Both	All Ages	Global	0.486	1.889	2.342	2.794	4.197	pack-year	85.227
Smoking	0.257	3 stars	Both	All Ages	Global	0.486	1.889	2.342	2.794	4.197	pack-year	86.364
Smoking	0.257	3 stars	Both	All Ages	Global	0.486	1.889	2.342	2.794	4.197	pack-year	87.500
Smoking	0.257	3 stars	Both	All Ages	Global	0.486	1.889	2.342	2.794	4.197	pack-year	88.636
Smoking	0.257	3 stars	Both	All Ages	Global	0.486	1.889	2.342	2.794	4.197	pack-year	89.773
Smoking	0.257	3 stars	Both	All Ages	Global	0.486	1.889	2.342	2.794	4.197	pack-year	90.909
Smoking	0.257	3 stars	Both	All Ages	Global	0.486	1.889	2.342	2.794	4.197	pack-year	92.045
Smoking	0.257	3 stars	Both	All Ages	Global	0.486	1.889	2.342	2.794	4.197	pack-year	93.182
Smoking	0.257	3 stars	Both	All Ages	Global	0.486	1.889	2.342	2.794	4.197	pack-year	94.318
Smoking	0.257	3 stars	Both	All Ages	Global	0.486	1.889	2.342	2.794	4.197	pack-year	95.455
Smoking	0.257	3 stars	Both	All Ages	Global	0.486	1.889	2.342	2.794	4.197	pack-year	96.591
Smoking	0.257	3 stars	Both	All Ages	Global	0.486	1.889	2.342	2.794	4.197	pack-year	97.727
Smoking	0.257	3 stars	Both	All Ages	Global	0.486	1.889	2.342	2.794	4.197	pack-year	98.864
Smoking	0.257	3 stars	Both	All Ages	Global	0.486	1.889	2.342	2.794	4.197	pack-year	100.000
Smoking	0.257	3 stars	Both	All Ages	Global	0.486	1.889	2.342	2.794	4.197	pack-year	101.136
Smoking	0.257	3 stars	Both	All Ages	Global	0.486	1.889	2.342	2.794	4.197	pack-year	102.273
Smoking	0.257	3 stars	Both	All Ages	Global	0.486	1.889	2.342	2.794	4.197	pack-year	103.409
Smoking	0.257	3 stars	Both	All Ages	Global	0.486	1.889	2.342	2.794	4.197	pack-year	104.545
Smoking	0.257	3 stars	Both	All Ages	Global	0.486	1.889	2.342	2.794	4.197	pack-year	105.682
Smoking	0.257	3 stars	Both	All Ages	Global	0.486	1.889	2.342	2.794	4.197	pack-year	106.818
Smoking	0.257	3 stars	Both	All Ages	Global	0.486	1.889	2.342	2.794	4.197	pack-year	107.955
Smoking	0.257	3 stars	Both	All Ages	Global	0.486	1.889	2.342	2.794	4.197	pack-year	109.091
Smoking	0.257	3 stars	Both	All Ages	Global	0.486	1.889	2.342	2.794	4.197	pack-year	110.227
Smoking	0.257	3 stars	Both	All Ages	Global	0.486	1.889	2.342	2.794	4.197	pack-year	111.364
Smoking	0.257	3 stars	Both	All Ages	Global	0.486	1.889	2.342	2.794	4.197	pack-year	112.500

S1-4 Data analysis

ASRs were calculated on the basis of the following formula:

$$ASR=\frac{\sum_{i=1}^{A} a_i\omega_i}{\sum_{i=1}^{A}\omega_i}\times 100{,}000$$

The ASR (per 100,000 population) is equal to the sum of the product of the specific age ratio a_i in age group i and the number (or weight) ω_i of the selected reference standard population group i divided by the sum of number (or weight) of the standard population [1,2]. Meanwhile, EAPC, which is approximately equal to the annual change for a specified range, was calculated using the regression model to assess the trends in ASR: $Y = \alpha + \beta X + \varepsilon$, where Y refers to ln(ASR), X represents calendar year, ε means error term, and β denotes the slope, which determines the positive or negative trends in ASR. The EAPC could be given by $EAPC = 100 \times (e^{\beta} - 1)$ as well as its 95% uncertainty interval (CI) [1,2]. For the GBD world population age-standard, please find the following table for your reference.

**GBD world population age standard**		
**Age Group**	**Percent of Population**	**Rounded**
Early Neonatal	0.0396232	0.04
Late Neonatal	0.117777	0.12
1 to 5 months	0.864776	0.86
6 to 11 months	1.01009	1.01
12 to 23 months	2.01613	2.02
2 to 4	5.9934	5.99
5 to 9	9.65824	9.66
10 to 14	8.99361	8.99
15 to 19	8.28913	8.29
20 to 24	7.80122	7.8
25 to 29	7.59144	7.59
30 to 34	7.32171	7.32
35 to 39	6.82805	6.83
40 to 44	6.14735	6.15
45 to 49	5.51133	5.51
50 to 54	4.91312	4.91
55 to 59	4.34586	4.35
60 to 64	3.68223	3.68
65 to 69	2.98509	2.99
70 to 74	2.26526	2.27
75 to 79	1.59758	1.6
80 to 84	1.09729	1.1
85 to 89	0.604519	0.6
90 to 94	0.246663	0.25
95 plus	0.0785092	0.08

S1-5 Modelling

Similar to GBD 2019, for most diseases and injuries in GBD 2021, processed data are modeled using standardized tools to generate estimates of each quantity of interest by age, sex, location, and year. There are three main standardized tools: Cause of Death Ensemble model (CODEm), spatiotemporal Gaussian process regression (ST-GPR), and disease model-Bayesian meta-regression (DisMod-MR) [1,2]. Briefly, CODEm is a highly systematized tool to analyze the cause of death data using an ensemble of different modelling methods for rates or cause fractions with varying choices of covariates that perform best with out-of-sample predictive validity testing. DisMod-MR is a Bayesian meta-regression tool that allows the evaluation of all available data on incidence, prevalence, remission, and mortality for a disease, enforcing consistency between epidemiological parameters. ST-GPR is a set of regression methods that borrow strength between locations and over time for single metrics of interest, such as risk factor exposure or mortality rates. In addition, for select diseases, particularly for rarer outcomes, alternative modelling strategies have been developed [1,2].

S1-6 Socio-demographic Index, annual rate of change, and data presentation

GBD 2021 metrics were estimated as counts, all-age and age-specific rates per 100,000 population, and age-standardized rates per 100,000 population, calculated using the GBD standard population structure. Percentage changes over specified time periods (e.g., 2010–2021) and annualized rates of change were presented, calculated as the difference in the natural logarithm of the values at the start and end of the time interval divided by the number of years in the interval. Each calculation was performed 500 times to generate draw-level estimates. The number of computations per process was reduced from 1000, as in previous GBD rounds, to 500 for GBD 2021 because simulation testing indicated that this reduction did not affect the final estimates and their uncertainty. Final estimates represent the mean across 500 draws, with 95% uncertainty intervals (UIs) represented by the 2.5th and 97.5th percentile values across the draws. Uncertainty was propagated at each step of the estimation process [1,2]. Sociodemographic development has been a key contributor to health gains over the three decades during which GBD has tracked changes in burden by location. The Socio-demographic Index (SDI) is a composite indicator representing the geometric mean of three parameters: lag-distributed income per capita, average years of schooling, and the fertility rate in females younger than 25 years. SDI scores were rescaled from 0 (lowest income and years of schooling, and highest fertility) to 100 (highest income and years of schooling, and lowest fertility). In the GBD 2021 study, SDI scores are defined within the range of [0, 100]. To maintain consistency with previous SDI presentations, this study converts the range to [0, 1] [1,2].

*S1-7 Definition of GBD s*uper regions and regions

GBD created super regions based on two criteria: epidemiological similarity and geographic closeness. The seven super regions are further sub-divided into 21 GBD regions [1,2].

(1) **South-East Asia, East Asia and Oceania:** Southeast Asia, East Asia, Oceania

(2) **Central Europe, Eastern Europe and Central Asia:** Central Europe, Eastern Europe, Central Asia

(3) **High Income:** Western Europe, Southern Latin America, High-income North America, High-income Asia Pacific, Australasia

(4) **Latin America and Caribbean:** Tropical Latin America, Andean Latin America, Central Latin America, Caribbean

(5) **North Africa and Middle East**

(6) **South Asia**

(7) **Sub-Saharan Africa:** Western Sub-Saharan Africa, Southern Sub-Saharan Africa, Central Sub-Saharan Africa, Eastern Sub-Saharan Africa

References

1. Brauer M, Roth GA, Aravkin AY, et al. Global burden and strength of evidence for 88 risk factors in 204 countries and 811 subnational locations, 1990–2021: a systematic analysis for the Global Burden of Disease Study 2021. *The Lancet*. 2024;403(10440):2162-2203. doi:10.1016/S0140-6736(24)00933-4
2. Naghavi M, Ong KL, Aali A, et al. Global burden of 288 causes of death and life expectancy decomposition in 204 countries and territories and 811 subnational locations, 1990–2021: a systematic analysis for the Global Burden of Disease Study 2021. *The Lancet*. 2024;403(10440):2100-2132. doi:10.1016/S0140-6736(24)00367-2

Supplementary Note S2 | Analysis of the EC diagnoses, deaths, and DALY changes in pandemic with Joinpoint regression and PPRD values.

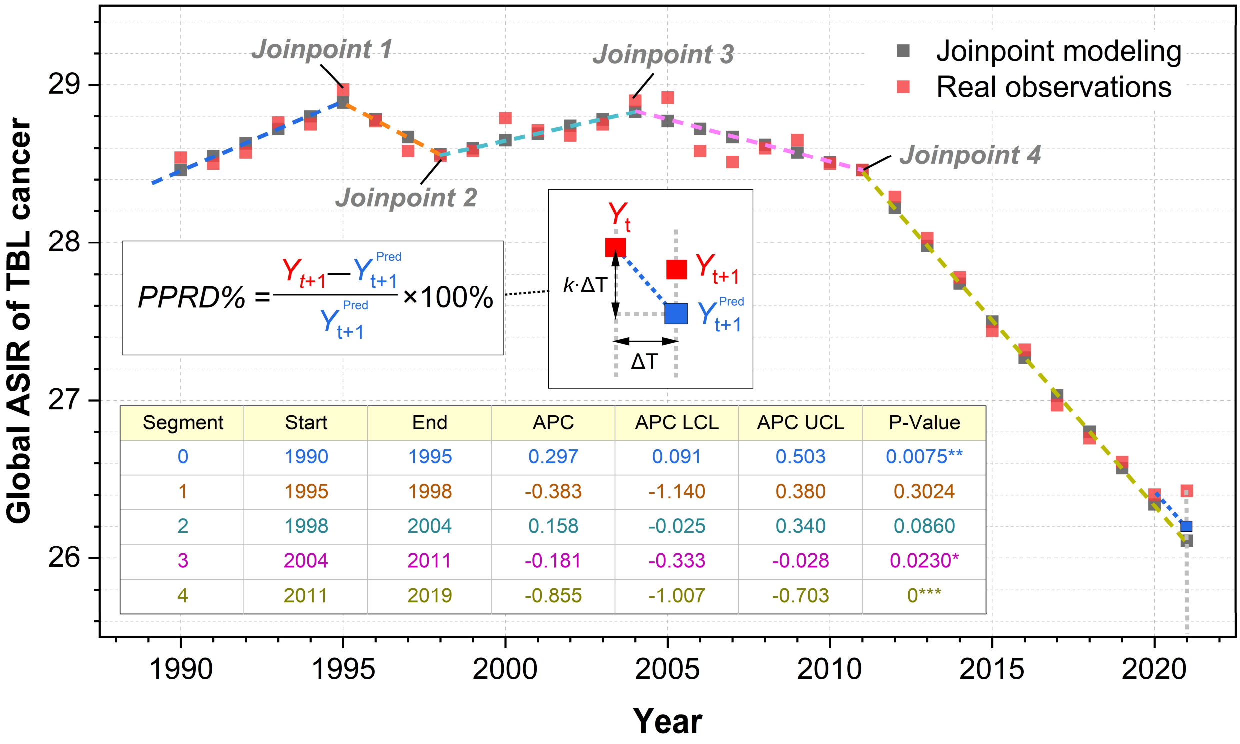

Segment	Start	End	APC	APC LCL	APC UCL	P-Value
0	1990	1995	0.297	0.091	0.503	0.0075**
1	1995	1998	-0.383	-1.140	0.380	0.3024
2	1998	2004	0.158	-0.025	0.340	0.0860
3	2004	2011	-0.181	-0.333	-0.028	0.0230*
4	2011	2019	-0.855	-1.007	-0.703	0***

The above figure gives an example explaining how the Joinpoint algorithm describes the epidemiological temporal trends of a certain burden of disease. As is shown in this figure, the algorithm automatically finds the connecting points in a time series, and divides the curve into several segments. In each segment, linear regression was performed, and the average percentages of change could be estimated with the regression results. The principle of Joinpoint algorithm allows it to find the connection points in the data with the least sum of errors. The slope of the last segment actually describes the most recent linear temporal trends of certain disease burden metrics. **Supplementary Figure S1** showed an example of Joinpoint analysis for the global ASIR of TBL cancer in 1990-2019. The last segment was from 2011 to 2019, where the annual percent change (APC) was estimated to be -0.855 [-1.007, -0.703], with a p value less than 0.001. By extending the linear trends to 2020 and 2021, we obtain the forecast value of ASIR. By comparing the projected value and the real data, we get the Percentages of Predicted value and Real value Difference (PPRD%):

$$PPRD\% = \frac{Y_{t+1}^{\text{real}} - Y_{t+1}^{\text{pred}}}{Y_{t+1}^{\text{pred}}} \times 100\%$$

where $Y_{t+1}^{\text{pred}} = Y_t^{\text{real}} + k \cdot \Delta T = Y_t^{\text{real}} + k$, Y_t represented the incidence rate in a given year t, while Y_t^{pred} and Y_t^{real} denoted the predicted and actual values, respectively; k represents the slope of the final linear segment in the model. By setting t to 2019 and 2020, we obtained the $PPRD\%$ for the years 2020 and 2021. The PPRD%

values describe the relative difference between the actual reported incidence rates and the model-predicted incidence rates for the corresponding years. The statistical significance of the final segment in the Joinpoint segmented model is crucial, as it determines whether the slope *k* used for linear projection, is reliable. For locations where the final segment failed to meet the threshold for statistical significance ($p < 0.05$), we excluded them from the calculation of PPRD%, as their recent epidemiological trends lack linear characteristics. In such cases, the PPRD% values derived should be considered invalid. There were 72.5% (148/204), 71.1% (145/204) and 72.5% (148/204) locations of significant linear epidemiological trends included in PPRD-ASIR, PPRD-ASDR and PPRD-ASDALYR analysis respectively.

In the analysis of 148 countries with linear trends in incidence rates, the distribution of PPRD values for 2020 (**eTable S16**) was as follows: countries where PPRD-ASIR exceeded 5% included: Bolivarian Republic of Venezuela (8.42%), Republic of Belarus (6.67%) and American Samoa (6.15%).The distribution of other countries' PPRD values was as follows: 10 countries between 2%-5%,9 countries between 1%-2%, 51 countries between 0%-1%, 27 countries between -1% and 0%, 18 countries between -2% and -1%, and 23 countries between -5% and -2%. Significant losses exceeding 5% occurred in countries such as Republic of San Marino (-11.81%), United Arab Emirates (-8.07%), Democratic Socialist Republic of Sri Lanka (-7.30%), Principality of Andorra (-6.45%), Guam (-5.28%) and Kingdom of Belgium (-5.05%). In 2021, 73 countries experienced a loss in ASIR. The number of countries with losses between -1% and 0%, -2% and -1% and -5% and -2% were 40, 14, and 15, respectively. Four countries, Republic of Peru (-7.60%), Republic of Trinidad and Tobago (-6.48%), Republic of Mauritius (-5.91%) and Republic of Guyana (-5.30%) experienced losses exceeding 5%. Conversely, Grenada (11.53%), Bolivarian Republic of Venezuela (10.51%), Republic of San Marino (8.93%), Puerto Rico (8.04%) and Republic of Belarus (6.10%) had the most significant relative increases in ASIR, while other locations showed increases of less than 5%. Specifically, 27 countries had increases between 1%-5% and 42 countries between 0%-1%.

Over the two years, significant increases exceeding 5% occurred in three countries: Bolivarian Republic of Venezuela (9.46%), Grenada (7.68%) and Republic of Belarus (6.39%). The distribution of other countries' PPRD values was as follows: 9 countries between 2%-5%, 2 countries between 1%-2%, 49 countries between 0%-1%, 51 countries between -1% and 0%, 16 countries between -2% and -1%, and 16 countries between -5% and -2%. The only country that experienced PPRD-ASIR loss exceeding 5% was Republic of Trinidad and Tobago (-5.31%). Notable fluctuations were observed in Republic of San Marino (-11.81% in 2020 and 8.93% in 2021), United Arab Emirates (-8.07% in 2020 and 2.58% in 2021), Principality of Andorra (-6.45% in 2020 and 3.61% in 2021) and Guam (-5.28% in 2020 and 2.90% in 2021). Conversely, Grenada (4.13% in 2020

and 11.53% in 2021) and Republic of Belarus (6.67% in 2020 and 6.10% in 2021) showed significant relative increases over this period, while Republic of Trinidad and Tobago (-4.11% in 2020 and -6.48% in 2021), Republic of Mauritius (-3.14% in 2020 and -5.91% in 2021) and Republic of Guyana (-3.64% in 2020 and -5.30% in 2021) showed significant relative losses.

In the analysis of 145 countries with linear trends in death rates, the distribution of PPRD values for 2020 (**eTable S17**) was as follows: countries where PPRD-ASDR exceeded 5% included: Republic of Belarus (6.67%) and American Samoa (5.60%). The distribution of other countries' PPRD values was as follows: 10 countries between 2%-5%, 6 countries between 1%-2%, 56 countries between 0%-1%, 25 countries between -1% and 0%, 15 countries between -2% and -1%, and 25 countries between -5% and -2%. Significant losses exceeding 5% occurred in countries such as Republic of San Marino (-12.35%), United Arab Emirates (-8.18%), Democratic Socialist Republic of Sri Lanka (-8.05%), Principality of Andorra (-6.82%) and Grand Duchy of Luxembourg (-5.88%). In 2021, 82 countries experienced a loss in ASDR. The number of countries with losses between -1% and 0%, -2% and -1% and -5% and -2% were 50, 10, and 18, respectively. Four countries, Montenegro (-7.36%), Republic of Peru (-7.32%), Republic of Trinidad and Tobago (-6.26%) and Republic of Mauritius (-5.50%) experienced losses exceeding 5%. Conversely, Grenada (10.86%), Republic of San Marino (10.04%), Puerto Rico (7.92%), Malaysia (6.55%) and Republic of Belarus (6.37%) had the most significant relative increases in ASDR, while other locations showed increases of less than 5%. Specifically, 30 countries had increases between 1%-5% and 27 countries between 0%-1%.

Over the two years, significant increases exceeding 5% occurred in two countries: Grenada (7.19%) and Republic of Belarus (6.52%). The distribution of other countries' PPRD values was as follows: 8 countries between 2%-5%, 4 countries between 1%-2%, 49 countries between 0%-1%, 46 countries between -1% and 0%, 14 countries between -2% and -1%, and 19 countries between -5% and -2%. The two countries that experienced PPRD-ASDR losses exceeding 5% was Grand Duchy of Luxembourg (-5.17%) and Republic of Trinidad and Tobago (-5.10%). Notable fluctuations were observed in Republic of San Marino (-12.35% in 2020 and 10.04% in 2021), United Arab Emirates (-8.18% in 2020 and 3.02% in 2021) and Principality of Andorra (-6.82% in 2020 and 3.15% in 2021). Conversely, Republic of Belarus (6.67% in 2020 and 6.37% in 2021) and Grenada (3.81% in 2020 and 10.86% in 2021) showed significant relative increases over this period, while Republic of Trinidad and Tobago (-3.93% in 2020 and -6.26% in 2021), Republic of Mauritius (-3.15% in 2020 and -5.50% in 2021) and Grand Duchy of Luxembourg (-5.88% in 2020 and -4.45% in 2021) showed significant relative losses.

In the analysis of 148 countries with linear trends in DALYs rates, the distribution of PPRD values for 2020 (**eTable S18**) was as follows: countries where PPRD-ASDALYR exceeded 5% included: Bolivarian Republic of Venezuela (9.18%), Republic of Belarus (6.48%) and American Samoa (6.33%). The distribution of other countries' PPRD values was as follows: 11 countries between 2%-5%, 5 countries between 1%-2%, 52 countries between 0%-1%, 30 countries between -1% and 0%, 14 countries between -2% and -1%, and 23 countries between -5% and -2%. Significant losses exceeding 5% occurred in countries such as Republic of San Marino (-11.33%), United Arab Emirates (-7.74%), Principality of Andorra (-6.75%), Grand Duchy of Luxembourg (-6.02%), Kingdom of Belgium (-5.64%), Republic of Trinidad and Tobago (-5.64%), Democratic Socialist Republic of Sri Lanka (-5.48%), Republic of Moldova (-5.33%) and Malaysia (-5.31%). In 2021, 64 countries experienced a loss in ASDALYR. The number of countries with losses between -1% and 0%, -2% and -1% and -5% and -2% were 36, 7, and 16, respectively. Five countries, Republic of Trinidad and Tobago (-8.62%), Republic of Peru (-6.68%), Saint Lucia (-6.27%), Republic of Moldova (-6.21%) and Republic of Guyana (-6.15%) experienced losses exceeding 5%. Conversely, Bolivarian Republic of Venezuela (11.42%), Grenada (11.41%), Republic of San Marino (8.26%), Republic of Costa Rica (8.01%), Puerto Rico (7.40%) and Republic of Belarus (6.08%) had the most significant relative increases in ASDALYR, while other locations showed increases of less than 5%. Specifically, 34 countries had increases between 1%-5% and 43 countries between 0%-1%.

Over the two years, significant increases exceeding 5% occurred in three countries: Bolivarian Republic of Venezuela (10.29%), Grenada (7.43%) and Republic of Belarus (6.28%). The distribution of other countries' PPRD values was as follows: 6 countries between 2%-5%, 9 countries between 1%-2%, 52 countries between 0%-1%, 46 countries between -1% and 0%, 14 countries between -2% and -1%, and 14 countries between -5% and -2%. The countries that experienced PPRD-ASDALYR losses exceeding 5% was Republic of Trinidad and Tobago (-7.14%), Republic of Moldova (-5.77%) and Republic of Guyana (-5.31%). Notable fluctuations were observed in Republic of San Marino (-11.33% in 2020 and 8.26% in 2021), United Arab Emirates (-7.74% in 2020 and 2.66% in 2021), Principality of Andorra (-6.75% in 2020 and 3.50% in 2021) and Kingdom of Belgium (-5.64% in 2020 and 3.62% in 2021). Conversely, Bolivarian Republic of Venezuela (9.18% in 2020 and 11.42% in 2021), Republic of Belarus (6.48% in 2020 and 6.08% in 2021) and Grenada (3.77% in 2020 and 11.41% in 2021) showed significant relative increases over this period, while Republic of Trinidad and Tobago (-5.64% in 2020 and -8.62% in 2021), Saint Lucia (-3.63% in 2020 and -6.27% in 2021), Republic of Moldova (-5.33% in 2020 and -6.21% in 2021) and Republic of Guyana (-4.46% in 2020 and -6.15% in 2021) showed significant relative losses.

Supplementary Figure S1 | The trends in ASPoD of esophageal cancer globally and across 21 GBD regions by SDI, 1990-2021

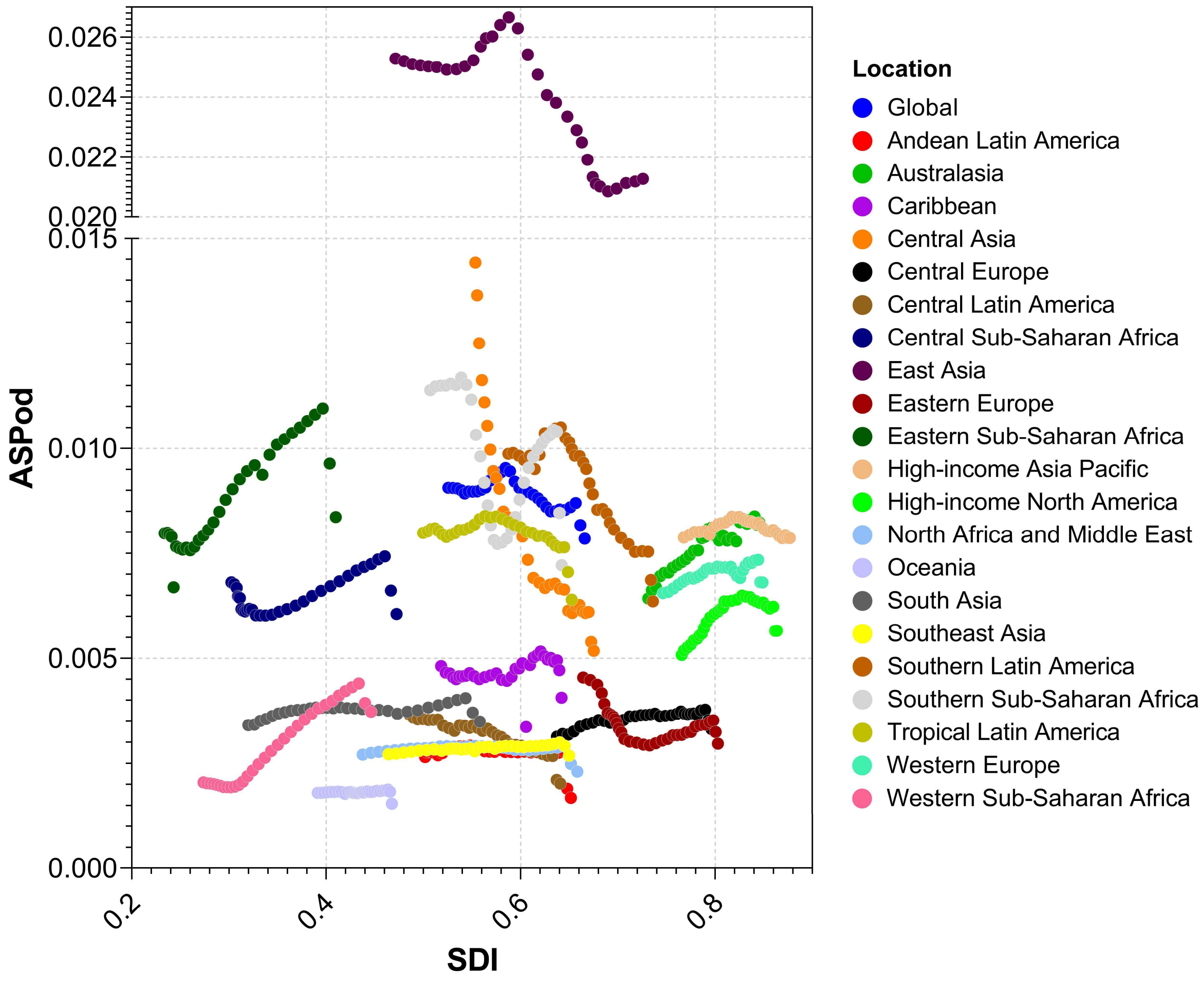

Supplementary Figure S2 | The changing patterns of age-standardized EC incidence, death, and DALY rates of countries and territories. The relationship between SDI and sum of PPRD% of ASRs for esophageal cancer in 2020-2021 (A-C). The relationship between PPRD-ASIR in 2020 and PPRD-ASIR in 2021 for countries and territories with different SDI value (D). The relationship between PPRD-ASDR in 2020 and PPRD-ASDR in 2021 in countries and territories with different SDI value (E).

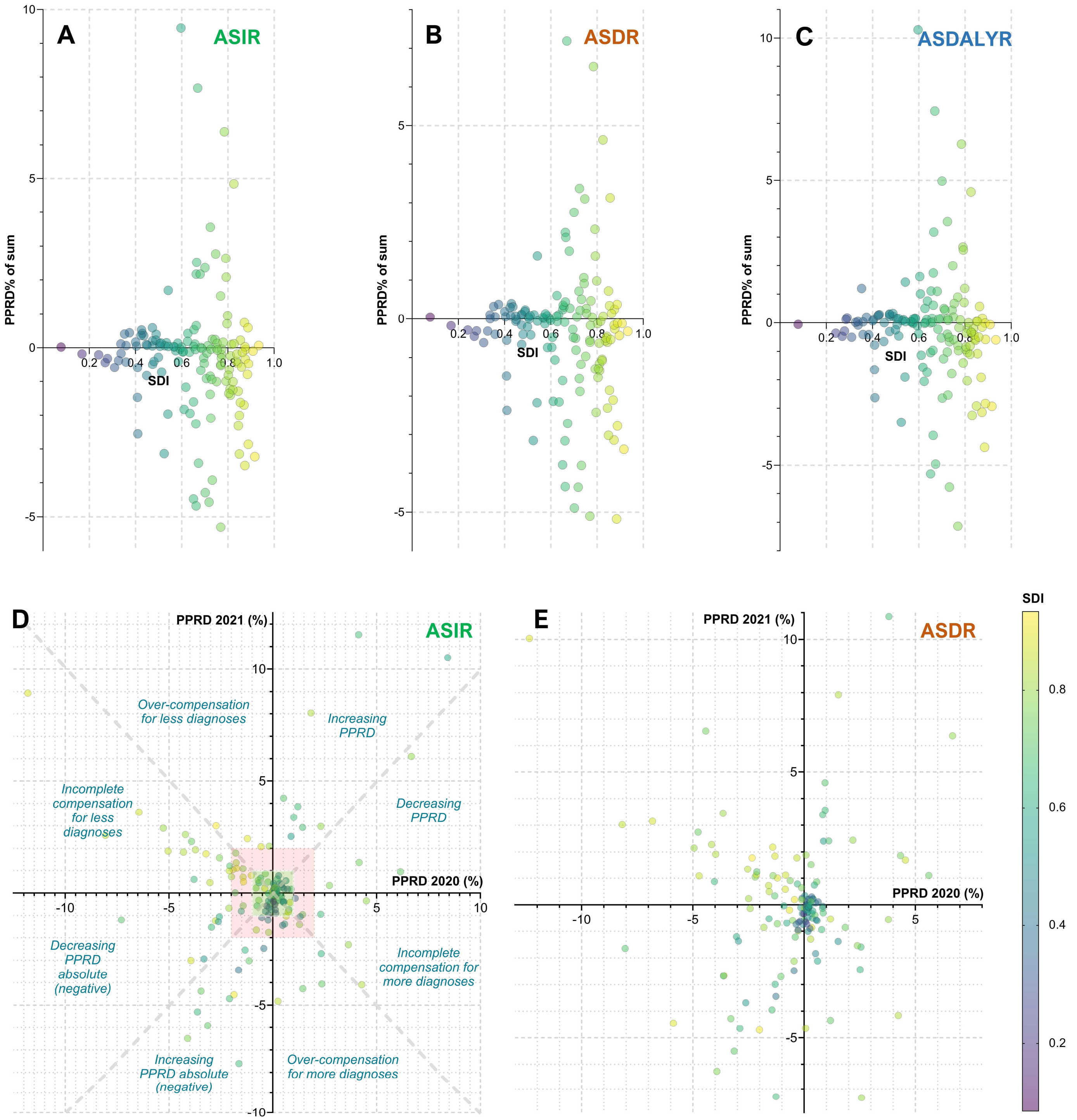

Supplementary Figure S3 | Decomposition result for the death changes in seven GBD super-regions during 1990-2021 by sexes

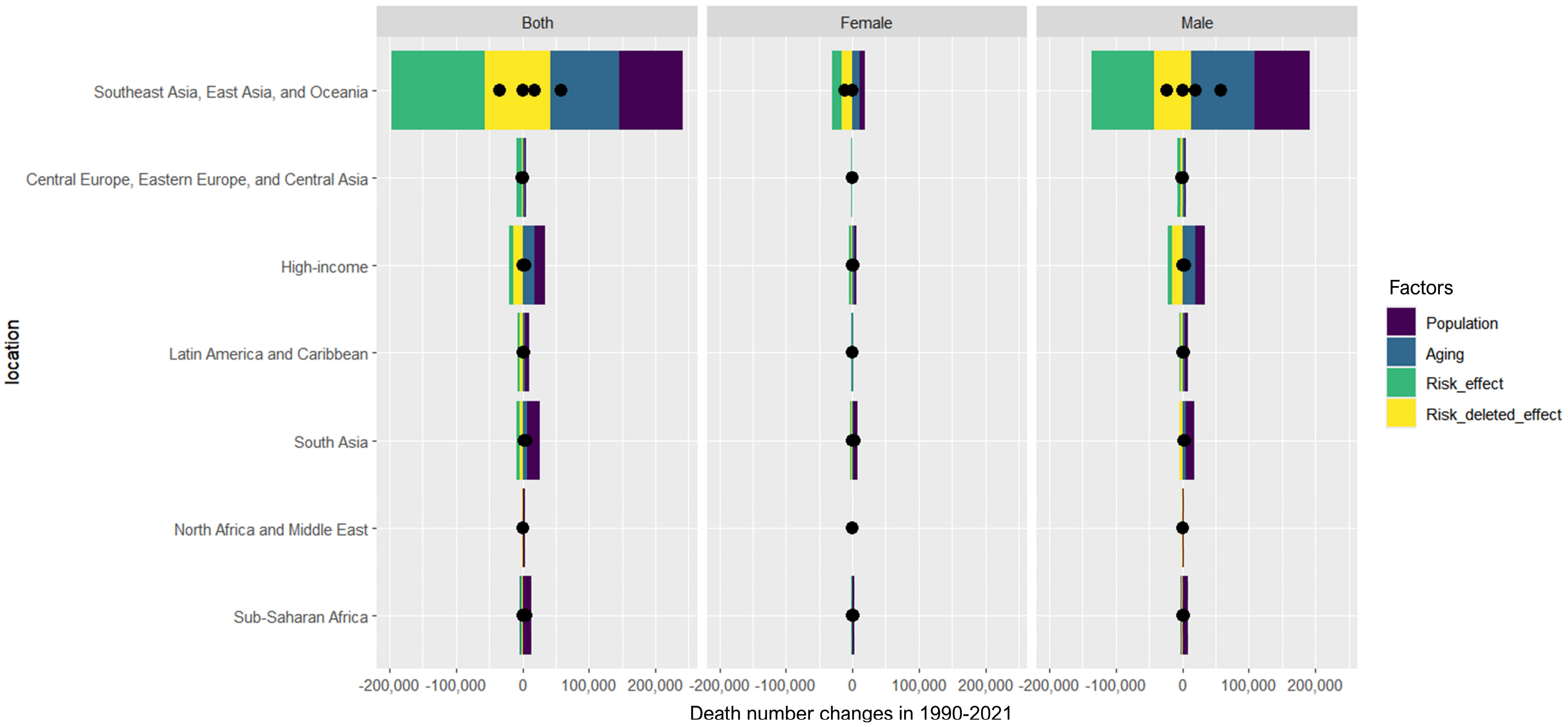

Supplementary Table S1 | Metrics definitions involved in this study

(a) Primary measure definitions

**Measure**	**Number**	**Rate**
Deaths	Number of deaths in the population	Deaths per 100,000 population
Disability adjusted life years (DALYs)<br>DALYs = YLLs + YLDs	Number of DALYs in the population	DALYs per 100,000 population
Years of life lost (YLLs)	Number of YLLs in the population	YLLs per 100,000 population
Years lived with disability (YLDs)	Number of YLDs in the population	YLDs per 100,000 population
Incidence	Number new of cases in the population	New cases per 100,000 population
Probability of death	Probability of dying due to a specific cause between a given age start and end, contingent upon being alive at age start	

(b) Secondary outputs of trend analysis (defined in main text)

**Trend indicators**	**Strengths**	**Weaknesses**	**Applied scenarios**
average annual percent change (AAPC)	△ Comprehensive description of average trends over a period by weighting different stable linear trends	△ May lack timeliness since distant time periods may dominate the weight sometimes<br>△ Linear trends in each period may not be significant<br>△ Requires sufficient data points to determine Joinpoints by algorithm	△ Best for describing overall average change directions and magnitudes over a long timeframe
segmented annual percentage changes (SAPC)	△ Focuses solely on the difference between two points, free from assumptions about linear changes	△ Cannot capture intermediate changes between two time points	△ Suitable when analyzing simple point-to-point changes without considering intermediate trends
estimated annual percentage change (EAPC)	△ Flexible and reflects trends over a specific period with fewer data points required compared to AAPC	△ Limited time span<br>△ Less comprehensive than AAPC when epidemiological burden transitions occur within the study period	△ Useful for short-term trend comparisons across multiple locations<br>△ More comprehensive than SAPC yet more flexible than AAPC

Supplementary Table S2 | Location hierarchies involved in this study

Location Set	Location ID	Location Name	Parent ID	Level
**GBD super-regions and regions**	1	Global	1	0
**GBD super-regions and regions**	31	Central Europe, Eastern Europe, and Central Asia	1	1
**GBD super-regions and regions**	32	Central Asia	31	2
**GBD super-regions and regions**	33	Armenia	32	3
**GBD super-regions and regions**	34	Azerbaijan	32	3
**GBD super-regions and regions**	35	Georgia	32	3
**GBD super-regions and regions**	36	Kazakhstan	32	3
**GBD super-regions and regions**	37	Kyrgyzstan	32	3
**GBD super-regions and regions**	38	Mongolia	32	3
**GBD super-regions and regions**	39	Tajikistan	32	3
**GBD super-regions and regions**	40	Turkmenistan	32	3
**GBD super-regions and regions**	41	Uzbekistan	32	3
**GBD super-regions and regions**	42	Central Europe	31	2
**GBD super-regions and regions**	43	Albania	42	3
**GBD super-regions and regions**	44	Bosnia and Herzegovina	42	3
**GBD super-regions and regions**	45	Bulgaria	42	3
**GBD super-regions and regions**	46	Croatia	42	3
**GBD super-regions and regions**	47	Czechia	42	3
**GBD super-regions and regions**	48	Hungary	42	3
**GBD super-regions and regions**	50	Montenegro	42	3
**GBD super-regions and regions**	49	North Macedonia	42	3
**GBD super-regions and regions**	51	Poland	42	3
**GBD super-regions and regions**	52	Romania	42	3
**GBD super-regions and regions**	53	Serbia	42	3
**GBD super-regions and regions**	54	Slovakia	42	3
**GBD super-regions and regions**	55	Slovenia	42	3
**GBD super-regions and regions**	56	Eastern Europe	31	2
**GBD super-regions and regions**	57	Belarus	56	3
**GBD super-regions and regions**	58	Estonia	56	3
**GBD super-regions and regions**	59	Latvia	56	3
**GBD super-regions and regions**	60	Lithuania	56	3
**GBD super-regions and regions**	61	Republic of Moldova	56	3
**GBD super-regions and regions**	62	Russian Federation	56	3
**GBD super-regions and regions**	63	Ukraine	56	3

**GBD super-regions and regions**	64	High-income	1	1
**GBD super-regions and regions**	70	Australasia	64	2
**GBD super-regions and regions**	71	Australia	70	3
**GBD super-regions and regions**	72	New Zealand	70	3
**GBD super-regions and regions**	65	High-income Asia Pacific	64	2
**GBD super-regions and regions**	66	Brunei Darussalam	65	3
**GBD super-regions and regions**	67	Japan	65	3
**GBD super-regions and regions**	68	Republic of Korea	65	3
**GBD super-regions and regions**	69	Singapore	65	3
**GBD super-regions and regions**	100	High-income North America	64	2
**GBD super-regions and regions**	101	Canada	100	3
**GBD super-regions and regions**	349	Greenland	100	3
**GBD super-regions and regions**	102	United States of America	100	3
**GBD super-regions and regions**	96	Southern Latin America	64	2
**GBD super-regions and regions**	97	Argentina	96	3
**GBD super-regions and regions**	98	Chile	96	3
**GBD super-regions and regions**	99	Uruguay	96	3
**GBD super-regions and regions**	73	Western Europe	64	2
**GBD super-regions and regions**	74	Andorra	73	3
**GBD super-regions and regions**	75	Austria	73	3
**GBD super-regions and regions**	76	Belgium	73	3
**GBD super-regions and regions**	77	Cyprus	73	3
**GBD super-regions and regions**	78	Denmark	73	3
**GBD super-regions and regions**	79	Finland	73	3
**GBD super-regions and regions**	80	France	73	3
**GBD super-regions and regions**	81	Germany	73	3
**GBD super-regions and regions**	82	Greece	73	3
**GBD super-regions and regions**	83	Iceland	73	3
**GBD super-regions and regions**	84	Ireland	73	3
**GBD super-regions and regions**	85	Israel	73	3
**GBD super-regions and regions**	86	Italy	73	3
**GBD super-regions and regions**	87	Luxembourg	73	3
**GBD super-regions and regions**	88	Malta	73	3
**GBD super-regions and regions**	367	Monaco	73	3
**GBD super-regions and regions**	89	Netherlands	73	3
**GBD super-regions and regions**	90	Norway	73	3

**GBD super-regions and regions**	91	Portugal	73	3
**GBD super-regions and regions**	396	San Marino	73	3
**GBD super-regions and regions**	92	Spain	73	3
**GBD super-regions and regions**	93	Sweden	73	3
**GBD super-regions and regions**	94	Switzerland	73	3
**GBD super-regions and regions**	95	United Kingdom	73	3
**GBD super-regions and regions**	103	Latin America and Caribbean	1	1
**GBD super-regions and regions**	120	Andean Latin America	103	2
**GBD super-regions and regions**	121	Bolivia (Plurinational State of)	120	3
**GBD super-regions and regions**	122	Ecuador	120	3
**GBD super-regions and regions**	123	Peru	120	3
**GBD super-regions and regions**	104	Caribbean	103	2
**GBD super-regions and regions**	105	Antigua and Barbuda	104	3
**GBD super-regions and regions**	106	Bahamas	104	3
**GBD super-regions and regions**	107	Barbados	104	3
**GBD super-regions and regions**	108	Belize	104	3
**GBD super-regions and regions**	305	Bermuda	104	3
**GBD super-regions and regions**	109	Cuba	104	3
**GBD super-regions and regions**	110	Dominica	104	3
**GBD super-regions and regions**	111	Dominican Republic	104	3
**GBD super-regions and regions**	112	Grenada	104	3
**GBD super-regions and regions**	113	Guyana	104	3
**GBD super-regions and regions**	114	Haiti	104	3
**GBD super-regions and regions**	115	Jamaica	104	3
**GBD super-regions and regions**	385	Puerto Rico	104	3
**GBD super-regions and regions**	393	Saint Kitts and Nevis	104	3
**GBD super-regions and regions**	116	Saint Lucia	104	3
**GBD super-regions and regions**	117	Saint Vincent and the Grenadines	104	3
**GBD super-regions and regions**	118	Suriname	104	3
**GBD super-regions and regions**	119	Trinidad and Tobago	104	3
**GBD super-regions and regions**	422	United States Virgin Islands	104	3
**GBD super-regions and regions**	124	Central Latin America	103	2
**GBD super-regions and regions**	125	Colombia	124	3
**GBD super-regions and regions**	126	Costa Rica	124	3
**GBD super-regions and regions**	127	El Salvador	124	3
**GBD super-regions and regions**	128	Guatemala	124	3

**GBD super-regions and regions**	129	Honduras	124	3
**GBD super-regions and regions**	130	Mexico	124	3
**GBD super-regions and regions**	131	Nicaragua	124	3
**GBD super-regions and regions**	132	Panama	124	3
**GBD super-regions and regions**	133	Venezuela (Bolivarian Republic of)	124	3
**GBD super-regions and regions**	134	Tropical Latin America	103	2
**GBD super-regions and regions**	135	Brazil	134	3
**GBD super-regions and regions**	136	Paraguay	134	3
**GBD super-regions and regions**	137	North Africa and Middle East	1	1
**GBD super-regions and regions**	138	North Africa and Middle East	137	2
**GBD super-regions and regions**	160	Afghanistan	138	3
**GBD super-regions and regions**	139	Algeria	138	3
**GBD super-regions and regions**	140	Bahrain	138	3
**GBD super-regions and regions**	141	Egypt	138	3
**GBD super-regions and regions**	142	Iran (Islamic Republic of)	138	3
**GBD super-regions and regions**	143	Iraq	138	3
**GBD super-regions and regions**	144	Jordan	138	3
**GBD super-regions and regions**	145	Kuwait	138	3
**GBD super-regions and regions**	146	Lebanon	138	3
**GBD super-regions and regions**	147	Libya	138	3
**GBD super-regions and regions**	148	Morocco	138	3
**GBD super-regions and regions**	150	Oman	138	3
**GBD super-regions and regions**	149	Palestine	138	3
**GBD super-regions and regions**	151	Qatar	138	3
**GBD super-regions and regions**	152	Saudi Arabia	138	3
**GBD super-regions and regions**	522	Sudan	138	3
**GBD super-regions and regions**	153	Syrian Arab Republic	138	3
**GBD super-regions and regions**	154	Tunisia	138	3
**GBD super-regions and regions**	155	Türkiye	138	3
**GBD super-regions and regions**	156	United Arab Emirates	138	3
**GBD super-regions and regions**	157	Yemen	138	3
**GBD super-regions and regions**	158	South Asia	1	1
**GBD super-regions and regions**	159	South Asia	158	2
**GBD super-regions and regions**	161	Bangladesh	159	3
**GBD super-regions and regions**	162	Bhutan	159	3
**GBD super-regions and regions**	163	India	159	3

**GBD super-regions and regions**	164	Nepal	159	3
**GBD super-regions and regions**	165	Pakistan	159	3
**GBD super-regions and regions**	4	Southeast Asia, East Asia, and Oceania	1	1
**GBD super-regions and regions**	5	East Asia	4	2
**GBD super-regions and regions**	6	China	5	3
**GBD super-regions and regions**	7	Democratic People's Republic of Korea	5	3
**GBD super-regions and regions**	8	Taiwan (Province of China)	5	3
**GBD super-regions and regions**	21	Oceania	4	2
**GBD super-regions and regions**	298	American Samoa	21	3
**GBD super-regions and regions**	320	Cook Islands	21	3
**GBD super-regions and regions**	22	Fiji	21	3
**GBD super-regions and regions**	351	Guam	21	3
**GBD super-regions and regions**	23	Kiribati	21	3
**GBD super-regions and regions**	24	Marshall Islands	21	3
**GBD super-regions and regions**	25	Micronesia (Federated States of)	21	3
**GBD super-regions and regions**	369	Nauru	21	3
**GBD super-regions and regions**	374	Niue	21	3
**GBD super-regions and regions**	376	Northern Mariana Islands	21	3
**GBD super-regions and regions**	380	Palau	21	3
**GBD super-regions and regions**	26	Papua New Guinea	21	3
**GBD super-regions and regions**	27	Samoa	21	3
**GBD super-regions and regions**	28	Solomon Islands	21	3
**GBD super-regions and regions**	413	Tokelau	21	3
**GBD super-regions and regions**	29	Tonga	21	3
**GBD super-regions and regions**	416	Tuvalu	21	3
**GBD super-regions and regions**	30	Vanuatu	21	3
**GBD super-regions and regions**	9	Southeast Asia	4	2
**GBD super-regions and regions**	10	Cambodia	9	3
**GBD super-regions and regions**	11	Indonesia	9	3
**GBD super-regions and regions**	12	Lao People's Democratic Republic	9	3
**GBD super-regions and regions**	13	Malaysia	9	3
**GBD super-regions and regions**	14	Maldives	9	3
**GBD super-regions and regions**	183	Mauritius	9	3
**GBD super-regions and regions**	15	Myanmar	9	3
**GBD super-regions and regions**	16	Philippines	9	3
**GBD super-regions and regions**	186	Seychelles	9	3

**GBD super-regions and regions**	17	Sri Lanka	9	3
**GBD super-regions and regions**	18	Thailand	9	3
**GBD super-regions and regions**	19	Timor-Leste	9	3
**GBD super-regions and regions**	20	Viet Nam	9	3
**GBD super-regions and regions**	166	Sub-Saharan Africa	1	1
**GBD super-regions and regions**	167	Central Sub-Saharan Africa	166	2
**GBD super-regions and regions**	168	Angola	167	3
**GBD super-regions and regions**	169	Central African Republic	167	3
**GBD super-regions and regions**	170	Congo	167	3
**GBD super-regions and regions**	171	Democratic Republic of the Congo	167	3
**GBD super-regions and regions**	172	Equatorial Guinea	167	3
**GBD super-regions and regions**	173	Gabon	167	3
**GBD super-regions and regions**	174	Eastern Sub-Saharan Africa	166	2
**GBD super-regions and regions**	175	Burundi	174	3
**GBD super-regions and regions**	176	Comoros	174	3
**GBD super-regions and regions**	177	Djibouti	174	3
**GBD super-regions and regions**	178	Eritrea	174	3
**GBD super-regions and regions**	179	Ethiopia	174	3
**GBD super-regions and regions**	180	Kenya	174	3
**GBD super-regions and regions**	181	Madagascar	174	3
**GBD super-regions and regions**	182	Malawi	174	3
**GBD super-regions and regions**	184	Mozambique	174	3
**GBD super-regions and regions**	185	Rwanda	174	3
**GBD super-regions and regions**	187	Somalia	174	3
**GBD super-regions and regions**	435	South Sudan	174	3
**GBD super-regions and regions**	190	Uganda	174	3
**GBD super-regions and regions**	189	United Republic of Tanzania	174	3
**GBD super-regions and regions**	191	Zambia	174	3
**GBD super-regions and regions**	192	Southern Sub-Saharan Africa	166	2
**GBD super-regions and regions**	193	Botswana	192	3
**GBD super-regions and regions**	197	Eswatini	192	3
**GBD super-regions and regions**	194	Lesotho	192	3
**GBD super-regions and regions**	195	Namibia	192	3
**GBD super-regions and regions**	196	South Africa	192	3
**GBD super-regions and regions**	198	Zimbabwe	192	3
**GBD super-regions and regions**	199	Western Sub-Saharan Africa	166	2

**GBD super-regions and regions**	200	Benin	199	3
**GBD super-regions and regions**	201	Burkina Faso	199	3
**GBD super-regions and regions**	203	Cabo Verde	199	3
**GBD super-regions and regions**	202	Cameroon	199	3
**GBD super-regions and regions**	204	Chad	199	3
**GBD super-regions and regions**	205	Côte d'Ivoire	199	3
**GBD super-regions and regions**	206	Gambia	199	3
**GBD super-regions and regions**	207	Ghana	199	3
**GBD super-regions and regions**	208	Guinea	199	3
**GBD super-regions and regions**	209	Guinea-Bissau	199	3
**GBD super-regions and regions**	210	Liberia	199	3
**GBD super-regions and regions**	211	Mali	199	3
**GBD super-regions and regions**	212	Mauritania	199	3
**GBD super-regions and regions**	213	Niger	199	3
**GBD super-regions and regions**	214	Nigeria	199	3
**GBD super-regions and regions**	215	Sao Tome and Principe	199	3
**GBD super-regions and regions**	216	Senegal	199	3
**GBD super-regions and regions**	217	Sierra Leone	199	3
**GBD super-regions and regions**	218	Togo	199	3
**WHO regions**	479	WHO region	479	0
**WHO regions**	44563	African Region	479	1
**WHO regions**	139	Algeria	44563	2
**WHO regions**	168	Angola	44563	2
**WHO regions**	200	Benin	44563	2
**WHO regions**	193	Botswana	44563	2
**WHO regions**	201	Burkina Faso	44563	2
**WHO regions**	175	Burundi	44563	2
**WHO regions**	203	Cabo Verde	44563	2
**WHO regions**	202	Cameroon	44563	2
**WHO regions**	169	Central African Republic	44563	2
**WHO regions**	204	Chad	44563	2
**WHO regions**	176	Comoros	44563	2
**WHO regions**	170	Congo	44563	2
**WHO regions**	205	Côte d'Ivoire	44563	2
**WHO regions**	171	Democratic Republic of the Congo	44563	2
**WHO regions**	172	Equatorial Guinea	44563	2

**WHO regions**	178	Eritrea	44563	2
**WHO regions**	197	Eswatini	44563	2
**WHO regions**	179	Ethiopia	44563	2
**WHO regions**	173	Gabon	44563	2
**WHO regions**	206	Gambia	44563	2
**WHO regions**	207	Ghana	44563	2
**WHO regions**	208	Guinea	44563	2
**WHO regions**	209	Guinea-Bissau	44563	2
**WHO regions**	180	Kenya	44563	2
**WHO regions**	194	Lesotho	44563	2
**WHO regions**	210	Liberia	44563	2
**WHO regions**	181	Madagascar	44563	2
**WHO regions**	182	Malawi	44563	2
**WHO regions**	211	Mali	44563	2
**WHO regions**	212	Mauritania	44563	2
**WHO regions**	183	Mauritius	44563	2
**WHO regions**	184	Mozambique	44563	2
**WHO regions**	195	Namibia	44563	2
**WHO regions**	213	Niger	44563	2
**WHO regions**	214	Nigeria	44563	2
**WHO regions**	185	Rwanda	44563	2
**WHO regions**	215	Sao Tome and Principe	44563	2
**WHO regions**	216	Senegal	44563	2
**WHO regions**	186	Seychelles	44563	2
**WHO regions**	217	Sierra Leone	44563	2
**WHO regions**	196	South Africa	44563	2
**WHO regions**	435	South Sudan	44563	2
**WHO regions**	218	Togo	44563	2
**WHO regions**	190	Uganda	44563	2
**WHO regions**	189	United Republic of Tanzania	44563	2
**WHO regions**	191	Zambia	44563	2
**WHO regions**	198	Zimbabwe	44563	2
**WHO regions**	44567	Eastern Mediterranean Region	479	1
**WHO regions**	160	Afghanistan	44567	2
**WHO regions**	140	Bahrain	44567	2
**WHO regions**	177	Djibouti	44567	2

**WHO regions**	141	Egypt	44567	2
**WHO regions**	142	Iran (Islamic Republic of)	44567	2
**WHO regions**	143	Iraq	44567	2
**WHO regions**	144	Jordan	44567	2
**WHO regions**	145	Kuwait	44567	2
**WHO regions**	146	Lebanon	44567	2
**WHO regions**	147	Libya	44567	2
**WHO regions**	148	Morocco	44567	2
**WHO regions**	150	Oman	44567	2
**WHO regions**	165	Pakistan	44567	2
**WHO regions**	149	Palestine	44567	2
**WHO regions**	151	Qatar	44567	2
**WHO regions**	152	Saudi Arabia	44567	2
**WHO regions**	187	Somalia	44567	2
**WHO regions**	522	Sudan	44567	2
**WHO regions**	153	Syrian Arab Republic	44567	2
**WHO regions**	154	Tunisia	44567	2
**WHO regions**	156	United Arab Emirates	44567	2
**WHO regions**	157	Yemen	44567	2
**WHO regions**	44566	European Region	479	1
**WHO regions**	43	Albania	44566	2
**WHO regions**	74	Andorra	44566	2
**WHO regions**	33	Armenia	44566	2
**WHO regions**	75	Austria	44566	2
**WHO regions**	34	Azerbaijan	44566	2
**WHO regions**	57	Belarus	44566	2
**WHO regions**	76	Belgium	44566	2
**WHO regions**	44	Bosnia and Herzegovina	44566	2
**WHO regions**	45	Bulgaria	44566	2
**WHO regions**	46	Croatia	44566	2
**WHO regions**	77	Cyprus	44566	2
**WHO regions**	47	Czechia	44566	2
**WHO regions**	78	Denmark	44566	2
**WHO regions**	58	Estonia	44566	2
**WHO regions**	79	Finland	44566	2
**WHO regions**	80	France	44566	2

**WHO regions**	35	Georgia	44566	2
**WHO regions**	81	Germany	44566	2
**WHO regions**	82	Greece	44566	2
**WHO regions**	48	Hungary	44566	2
**WHO regions**	83	Iceland	44566	2
**WHO regions**	84	Ireland	44566	2
**WHO regions**	85	Israel	44566	2
**WHO regions**	86	Italy	44566	2
**WHO regions**	36	Kazakhstan	44566	2
**WHO regions**	37	Kyrgyzstan	44566	2
**WHO regions**	59	Latvia	44566	2
**WHO regions**	60	Lithuania	44566	2
**WHO regions**	87	Luxembourg	44566	2
**WHO regions**	88	Malta	44566	2
**WHO regions**	367	Monaco	44566	2
**WHO regions**	50	Montenegro	44566	2
**WHO regions**	89	Netherlands	44566	2
**WHO regions**	49	North Macedonia	44566	2
**WHO regions**	90	Norway	44566	2
**WHO regions**	51	Poland	44566	2
**WHO regions**	91	Portugal	44566	2
**WHO regions**	61	Republic of Moldova	44566	2
**WHO regions**	52	Romania	44566	2
**WHO regions**	62	Russian Federation	44566	2
**WHO regions**	396	San Marino	44566	2
**WHO regions**	53	Serbia	44566	2
**WHO regions**	54	Slovakia	44566	2
**WHO regions**	55	Slovenia	44566	2
**WHO regions**	92	Spain	44566	2
**WHO regions**	93	Sweden	44566	2
**WHO regions**	94	Switzerland	44566	2
**WHO regions**	39	Tajikistan	44566	2
**WHO regions**	155	Türkiye	44566	2
**WHO regions**	40	Turkmenistan	44566	2
**WHO regions**	63	Ukraine	44566	2
**WHO regions**	95	United Kingdom	44566	2

**WHO regions**	41	Uzbekistan	44566	2
**WHO regions**	44564	Region of the Americas	479	1
**WHO regions**	105	Antigua and Barbuda	44564	2
**WHO regions**	97	Argentina	44564	2
**WHO regions**	106	Bahamas	44564	2
**WHO regions**	107	Barbados	44564	2
**WHO regions**	108	Belize	44564	2
**WHO regions**	121	Bolivia (Plurinational State of)	44564	2
**WHO regions**	135	Brazil	44564	2
**WHO regions**	101	Canada	44564	2
**WHO regions**	98	Chile	44564	2
**WHO regions**	125	Colombia	44564	2
**WHO regions**	126	Costa Rica	44564	2
**WHO regions**	109	Cuba	44564	2
**WHO regions**	110	Dominica	44564	2
**WHO regions**	111	Dominican Republic	44564	2
**WHO regions**	122	Ecuador	44564	2
**WHO regions**	127	El Salvador	44564	2
**WHO regions**	112	Grenada	44564	2
**WHO regions**	128	Guatemala	44564	2
**WHO regions**	113	Guyana	44564	2
**WHO regions**	114	Haiti	44564	2
**WHO regions**	129	Honduras	44564	2
**WHO regions**	115	Jamaica	44564	2
**WHO regions**	130	Mexico	44564	2
**WHO regions**	131	Nicaragua	44564	2
**WHO regions**	132	Panama	44564	2
**WHO regions**	136	Paraguay	44564	2
**WHO regions**	123	Peru	44564	2
**WHO regions**	393	Saint Kitts and Nevis	44564	2
**WHO regions**	116	Saint Lucia	44564	2
**WHO regions**	117	Saint Vincent and the Grenadines	44564	2
**WHO regions**	118	Suriname	44564	2
**WHO regions**	119	Trinidad and Tobago	44564	2
**WHO regions**	102	United States of America	44564	2
**WHO regions**	99	Uruguay	44564	2

**WHO regions**	133	Venezuela (Bolivarian Republic of)	44564	2
**WHO regions**	44565	South-East Asia Region	479	1
**WHO regions**	161	Bangladesh	44565	2
**WHO regions**	162	Bhutan	44565	2
**WHO regions**	7	Democratic People's Republic of Korea	44565	2
**WHO regions**	163	India	44565	2
**WHO regions**	11	Indonesia	44565	2
**WHO regions**	14	Maldives	44565	2
**WHO regions**	15	Myanmar	44565	2
**WHO regions**	164	Nepal	44565	2
**WHO regions**	17	Sri Lanka	44565	2
**WHO regions**	18	Thailand	44565	2
**WHO regions**	19	Timor-Leste	44565	2
**WHO regions**	44568	Western Pacific Region	479	1
**WHO regions**	71	Australia	44568	2
**WHO regions**	66	Brunei Darussalam	44568	2
**WHO regions**	10	Cambodia	44568	2
**WHO regions**	6	China	44568	2
**WHO regions**	320	Cook Islands	44568	2
**WHO regions**	22	Fiji	44568	2
**WHO regions**	67	Japan	44568	2
**WHO regions**	23	Kiribati	44568	2
**WHO regions**	12	Lao People's Democratic Republic	44568	2
**WHO regions**	13	Malaysia	44568	2
**WHO regions**	24	Marshall Islands	44568	2
**WHO regions**	25	Micronesia (Federated States of)	44568	2
**WHO regions**	38	Mongolia	44568	2
**WHO regions**	369	Nauru	44568	2
**WHO regions**	72	New Zealand	44568	2
**WHO regions**	374	Niue	44568	2
**WHO regions**	380	Palau	44568	2
**WHO regions**	26	Papua New Guinea	44568	2
**WHO regions**	16	Philippines	44568	2
**WHO regions**	68	Republic of Korea	44568	2
**WHO regions**	27	Samoa	44568	2
**WHO regions**	69	Singapore	44568	2

**WHO regions**	28	Solomon Islands	44568	2
**WHO regions**	29	Tonga	44568	2
**WHO regions**	416	Tuvalu	44568	2
**WHO regions**	30	Vanuatu	44568	2
**WHO regions**	20	Viet Nam	44568	2

Supplementary Table S3 | SDI values in 1990, 2019, and 2021

location	location_name	SDI1990	SDI2019	SDI2021
**6**	China	0.4587	0.7037	0.7216
**7**	Democratic People's Republic of Korea	0.4978	0.5668	0.5699
**8**	Taiwan (Province of China)	0.6676	0.8674	0.8747
**10**	Cambodia	0.2891	0.4636	0.4736
**11**	Indonesia	0.4571	0.6469	0.6569
**12**	Lao People's Democratic Republic	0.2643	0.4788	0.4891
**13**	Malaysia	0.5458	0.7346	0.7425
**14**	Maldives	0.3316	0.6431	0.6509
**15**	Myanmar	0.3192	0.5241	0.5339
**16**	Philippines	0.5100	0.6386	0.6512
**17**	Sri Lanka	0.5226	0.6935	0.7015
**18**	Thailand	0.5066	0.6753	0.6825
**19**	Timor-Leste	0.2625	0.4402	0.4447
**20**	Viet Nam	0.4076	0.6177	0.6279
**22**	Fiji	0.5346	0.6671	0.6751
**23**	Kiribati	0.4104	0.5194	0.5272
**24**	Marshall Islands	0.4308	0.5634	0.5741
**25**	Micronesia (Federated States of)	0.4625	0.5812	0.5875
**26**	Papua New Guinea	0.3107	0.4123	0.4178
**27**	Samoa	0.4875	0.5863	0.5934
**28**	Solomon Islands	0.3012	0.4227	0.4294
**29**	Tonga	0.4918	0.6173	0.6263
**30**	Vanuatu	0.3531	0.4663	0.4731
**33**	Armenia	0.5444	0.6948	0.7018
**34**	Azerbaijan	0.5960	0.6898	0.6949
**35**	Georgia	0.6561	0.7245	0.7325
**36**	Kazakhstan	0.5894	0.7208	0.7251
**37**	Kyrgyzstan	0.5194	0.5965	0.6040
**38**	Mongolia	0.4666	0.6113	0.6176
**39**	Tajikistan	0.4662	0.5338	0.5415
**40**	Turkmenistan	0.5631	0.6743	0.6822
**41**	Uzbekistan	0.5002	0.6558	0.6626
**43**	Albania	0.5578	0.7003	0.7068

**44**	Bosnia and Herzegovina	0.5411	0.7169	0.7231
**45**	Bulgaria	0.6334	0.7624	0.7682
**46**	Croatia	0.6689	0.7925	0.7983
**47**	Czechia	0.6818	0.8248	0.8285
**48**	Hungary	0.6494	0.7849	0.7908
**49**	North Macedonia	0.6090	0.7452	0.7506
**50**	Montenegro	0.6742	0.7890	0.7958
**51**	Poland	0.6272	0.8048	0.8120
**52**	Romania	0.6193	0.7603	0.7685
**53**	Serbia	0.6305	0.7858	0.7924
**54**	Slovakia	0.6539	0.8065	0.8106
**55**	Slovenia	0.7275	0.8383	0.8424
**57**	Belarus	0.6224	0.7805	0.7845
**58**	Estonia	0.6750	0.8386	0.8449
**59**	Latvia	0.6802	0.8248	0.8307
**60**	Lithuania	0.6685	0.8479	0.8565
**61**	Republic of Moldova	0.6043	0.7230	0.7322
**62**	Russian Federation	0.6716	0.8037	0.8085
**63**	Ukraine	0.6475	0.7580	0.7608
**66**	Brunei Darussalam	0.6661	0.8045	0.8102
**67**	Japan	0.7903	0.8672	0.8712
**68**	Republic of Korea	0.6923	0.8807	0.8867
**69**	Singapore	0.6864	0.8525	0.8561
**71**	Australia	0.7260	0.8393	0.8443
**72**	New Zealand	0.7523	0.8445	0.8494
**74**	Andorra	0.7615	0.8656	0.8694
**75**	Austria	0.7499	0.8499	0.8538
**76**	Belgium	0.7374	0.8495	0.8537
**77**	Cyprus	0.6482	0.8310	0.8356
**78**	Denmark	0.8012	0.8916	0.8964
**79**	Finland	0.7562	0.8554	0.8598
**80**	France	0.7307	0.8338	0.8384
**81**	Germany	0.8171	0.8997	0.9030
**82**	Greece	0.6742	0.7881	0.7919
**83**	Iceland	0.7642	0.8724	0.8764
**84**	Ireland	0.7199	0.8694	0.8738

**85**	Israel	0.7092	0.8032	0.8090
**86**	Italy	0.7063	0.8015	0.8058
**87**	Luxembourg	0.7811	0.8806	0.8844
**88**	Malta	0.6565	0.7952	0.8016
**89**	Netherlands	0.7946	0.8846	0.8885
**90**	Norway	0.7959	0.9123	0.9161
**91**	Portugal	0.5998	0.7381	0.7442
**92**	Spain	0.6367	0.7639	0.7693
**93**	Sweden	0.7855	0.8830	0.8869
**94**	Switzerland	0.8628	0.9307	0.9331
**95**	United Kingdom	0.7443	0.8541	0.8590
**97**	Argentina	0.5874	0.7190	0.7231
**98**	Chile	0.5865	0.7651	0.7715
**99**	Uruguay	0.5819	0.7125	0.7193
**101**	Canada	0.7820	0.8706	0.8732
**102**	United States of America	0.7636	0.8586	0.8624
**105**	Antigua and Barbuda	0.6121	0.7431	0.7499
**106**	Bahamas	0.6935	0.8010	0.8050
**107**	Barbados	0.6536	0.7422	0.7467
**108**	Belize	0.4237	0.6034	0.6102
**109**	Cuba	0.5580	0.6617	0.6687
**110**	Dominica	0.5636	0.7421	0.7470
**111**	Dominican Republic	0.4427	0.6119	0.6194
**112**	Grenada	0.4367	0.6608	0.6690
**113**	Guyana	0.4604	0.6336	0.6508
**114**	Haiti	0.3103	0.4443	0.4483
**115**	Jamaica	0.5348	0.6778	0.6833
**116**	Saint Lucia	0.4963	0.6668	0.6725
**117**	Saint Vincent and the Grenadines	0.4759	0.6283	0.6372
**118**	Suriname	0.5021	0.6279	0.6337
**119**	Trinidad and Tobago	0.6240	0.7642	0.7688
**121**	Bolivia (Plurinational State of)	0.4239	0.5907	0.5990
**122**	Ecuador	0.5184	0.6518	0.6610
**123**	Peru	0.5104	0.6554	0.6621
**125**	Colombia	0.4807	0.6460	0.6554
**126**	Costa Rica	0.5341	0.6901	0.7003

**127**	El Salvador	0.3731	0.5540	0.5638
**128**	Guatemala	0.3118	0.5286	0.5400
**129**	Honduras	0.3320	0.5042	0.5130
**130**	Mexico	0.5050	0.6551	0.6646
**131**	Nicaragua	0.3460	0.5166	0.5240
**132**	Panama	0.5460	0.6967	0.7089
**133**	Venezuela (Bolivarian Republic of)	0.5169	0.6023	0.5965
**135**	Brazil	0.5001	0.6453	0.6530
**136**	Paraguay	0.4695	0.6261	0.6357
**139**	Algeria	0.4605	0.6482	0.6595
**140**	Bahrain	0.5846	0.7429	0.7530
**141**	Egypt	0.4172	0.5877	0.6068
**142**	Iran (Islamic Republic of)	0.4538	0.6867	0.6972
**143**	Iraq	0.4120	0.6436	0.6626
**144**	Jordan	0.5391	0.7150	0.7253
**145**	Kuwait	0.6645	0.8397	0.8467
**146**	Lebanon	0.5367	0.7386	0.7447
**147**	Libya	0.5280	0.7162	0.7258
**148**	Morocco	0.3581	0.5478	0.5627
**149**	Palestine	0.4018	0.6165	0.6310
**150**	Oman	0.4293	0.7645	0.7734
**151**	Qatar	0.6512	0.8358	0.8469
**152**	Saudi Arabia	0.5390	0.8061	0.8151
**153**	Syrian Arab Republic	0.4305	0.6133	0.6230
**154**	Tunisia	0.4711	0.6732	0.6824
**155**	Turkey	0.4616	0.6983	0.7127
**156**	United Arab Emirates	0.6444	0.8430	0.8493
**157**	Yemen	0.2157	0.4440	0.4504
**160**	Afghanistan	0.1738	0.3225	0.3372
**161**	Bangladesh	0.2285	0.4733	0.4924
**162**	Bhutan	0.2150	0.4646	0.4731
**163**	India	0.3326	0.5608	0.5754
**164**	Nepal	0.1996	0.4220	0.4332
**165**	Pakistan	0.3105	0.4910	0.5040
**168**	Angola	0.2707	0.4405	0.4537
**169**	Central African Republic	0.2168	0.3020	0.3092

**170**	Congo	0.4207	0.5734	0.5831
**171**	Democratic Republic of the Congo	0.2898	0.3693	0.3832
**172**	Equatorial Guinea	0.2688	0.6453	0.6579
**173**	Gabon	0.4554	0.6199	0.6347
**175**	Burundi	0.2059	0.2834	0.2894
**176**	Comoros	0.2700	0.4674	0.4760
**177**	Djibouti	0.3378	0.4753	0.4880
**178**	Eritrea	0.2160	0.3963	0.4039
**179**	Ethiopia	0.1480	0.3464	0.3588
**180**	Kenya	0.3339	0.5080	0.5238
**181**	Madagascar	0.2799	0.3842	0.4002
**182**	Malawi	0.2040	0.3701	0.3846
**183**	Mauritius	0.5446	0.7109	0.7183
**184**	Mozambique	0.1731	0.3132	0.3265
**185**	Rwanda	0.2751	0.4231	0.4356
**186**	Seychelles	0.5755	0.7221	0.7302
**187**	Somalia	0.0488	0.0750	0.0777
**189**	United Republic of Tanzania	0.2593	0.4302	0.4466
**190**	Uganda	0.1870	0.4084	0.4233
**191**	Zambia	0.3040	0.4879	0.5059
**193**	Botswana	0.4181	0.6353	0.6427
**194**	Lesotho	0.3392	0.5025	0.5104
**195**	Namibia	0.4500	0.6109	0.6176
**196**	South Africa	0.5416	0.6740	0.6796
**197**	Eswatini	0.3994	0.5772	0.5855
**198**	Zimbabwe	0.3986	0.4684	0.4738
**200**	Benin	0.2189	0.3600	0.3735
**201**	Burkina Faso	0.1297	0.2743	0.2851
**202**	Cameroon	0.3031	0.4643	0.4797
**203**	Cabo Verde	0.2767	0.5225	0.5335
**204**	Chad	0.1146	0.2317	0.2404
**205**	Cete d'Ivoire	0.2793	0.4117	0.4259
**206**	Gambia	0.2387	0.3992	0.4097
**207**	Ghana	0.3731	0.5516	0.5649
**208**	Guinea	0.1783	0.3228	0.3364
**209**	Guinea-Bissau	0.2076	0.3422	0.3531

**210**	Liberia	0.2353	0.3434	0.3524
**211**	Mali	0.1265	0.2563	0.2686
**212**	Mauritania	0.3358	0.4838	0.4989
**213**	Niger	0.0809	0.1609	0.1681
**214**	Nigeria	0.3059	0.4888	0.5034
**215**	Sao Tome and Principe	0.3095	0.4898	0.5054
**216**	Senegal	0.2380	0.3940	0.4081
**217**	Sierra Leone	0.2116	0.3458	0.3587
**218**	Togo	0.2697	0.3957	0.4085
**298**	American Samoa	0.6136	0.7173	0.7237
**305**	Bermuda	0.6965	0.8170	0.8214
**320**	Cook Islands	0.5645	0.7698	0.7791
**349**	Greenland	0.7323	0.8223	0.8262
**351**	Guam	0.6762	0.7968	0.8040
**367**	Monaco	0.8455	0.9050	0.9083
**369**	Nauru	0.5391	0.6127	0.6252
**374**	Niue	0.5875	0.7191	0.7262
**376**	Northern Mariana Islands	0.7086	0.7658	0.7715
**380**	Palau	0.6629	0.7507	0.7540
**385**	Puerto Rico	0.6588	0.8188	0.8255
**393**	Saint Kitts and Nevis	0.5807	0.7482	0.7550
**396**	San Marino	0.8132	0.8862	0.8880
**413**	Tokelau	0.5219	0.6793	0.6864
**416**	Tuvalu	0.4062	0.5661	0.5766
**422**	United States Virgin Islands	0.6552	0.8174	0.8218
**435**	South Sudan	0.2067	0.2757	0.2784
**522**	Sudan	0.2922	0.5250	0.5419

Supplementary Table S4 | SDI quintiles in GBD 2021 version

location_name	SDI2021	SDI quintiles
**Somalia**	0.0777	Low
**Niger**	0.1681	Low
**Chad**	0.2404	Low
**Mali**	0.2686	Low
**South Sudan**	0.2784	Low
**Burkina Faso**	0.2851	Low
**Burundi**	0.2894	Low
**Central African Republic**	0.3092	Low
**Mozambique**	0.3265	Low
**Guinea**	0.3364	Low
**Afghanistan**	0.3372	Low
**Liberia**	0.3524	Low
**Guinea-Bissau**	0.3531	Low
**Sierra Leone**	0.3587	Low
**Ethiopia**	0.3588	Low
**Benin**	0.3735	Low
**Democratic Republic of the Congo**	0.3832	Low
**Malawi**	0.3846	Low
**Madagascar**	0.4002	Low
**Eritrea**	0.4039	Low
**Senegal**	0.4081	Low
**Togo**	0.4085	Low
**Gambia**	0.4097	Low
**Papua New Guinea**	0.4178	Low
**Uganda**	0.4233	Low
**Cete d'Ivoire**	0.4259	Low
**Solomon Islands**	0.4294	Low
**Nepal**	0.4332	Low
**Rwanda**	0.4356	Low
**Timor-Leste**	0.4447	Low
**United Republic of Tanzania**	0.4466	Low
**Haiti**	0.4483	Low
**Yemen**	0.4504	Low

**Angola**	0.4537	Low
**Vanuatu**	0.4731	Low
**Bhutan**	0.4731	Low
**Cambodia**	0.4736	Low
**Zimbabwe**	0.4738	Low
**Comoros**	0.476	Low
**Cameroon**	0.4797	Low middle
**Djibouti**	0.488	Low middle
**Lao People's Democratic Republic**	0.4891	Low middle
**Bangladesh**	0.4924	Low middle
**Mauritania**	0.4989	Low middle
**Nigeria**	0.5034	Low middle
**Pakistan**	0.504	Low middle
**Sao Tome and Principe**	0.5054	Low middle
**Zambia**	0.5059	Low middle
**Lesotho**	0.5104	Low middle
**Honduras**	0.513	Low middle
**Kenya**	0.5238	Low middle
**Nicaragua**	0.524	Low middle
**Kiribati**	0.5272	Low middle
**Cabo Verde**	0.5335	Low middle
**Myanmar**	0.5339	Low middle
**Guatemala**	0.54	Low middle
**Tajikistan**	0.5415	Low middle
**Sudan**	0.5419	Low middle
**Morocco**	0.5627	Low middle
**El Salvador**	0.5638	Low middle
**Ghana**	0.5649	Low middle
**Democratic People's Republic of Korea**	0.5699	Low middle
**Marshall Islands**	0.5741	Low middle
**India**	0.5754	Low middle
**Tuvalu**	0.5766	Low middle
**Congo**	0.5831	Low middle
**Eswatini**	0.5855	Low middle
**Micronesia (Federated States of)**	0.5875	Low middle
**Samoa**	0.5934	Low middle

**Venezuela (Bolivarian Republic of)**	0.5965	Low middle
**Bolivia (Plurinational State of)**	0.599	Low middle
**Kyrgyzstan**	0.604	Low middle
**Egypt**	0.6068	Low middle
**Belize**	0.6102	Low middle
**Mongolia**	0.6176	Low middle
**Namibia**	0.6176	Low middle
**Dominican Republic**	0.6194	Low middle
**Syrian Arab Republic**	0.623	Low middle
**Nauru**	0.6252	Low middle
**Tonga**	0.6263	Low middle
**Viet Nam**	0.6279	Low middle
**Palestine**	0.631	Middle
**Suriname**	0.6337	Middle
**Gabon**	0.6347	Middle
**Paraguay**	0.6357	Middle
**Saint Vincent and the Grenadines**	0.6372	Middle
**Botswana**	0.6427	Middle
**Guyana**	0.6508	Middle
**Maldives**	0.6509	Middle
**Philippines**	0.6512	Middle
**Brazil**	0.653	Middle
**Colombia**	0.6554	Middle
**Indonesia**	0.6569	Middle
**Equatorial Guinea**	0.6579	Middle
**Algeria**	0.6595	Middle
**Ecuador**	0.661	Middle
**Peru**	0.6621	Middle
**Uzbekistan**	0.6626	Middle
**Iraq**	0.6626	Middle
**Mexico**	0.6646	Middle
**Cuba**	0.6687	Middle
**Grenada**	0.669	Middle
**Saint Lucia**	0.6725	Middle
**Fiji**	0.6751	Middle
**South Africa**	0.6796	Middle

**Turkmenistan**	0.6822	Middle
**Tunisia**	0.6824	Middle
**Thailand**	0.6825	Middle
**Jamaica**	0.6833	Middle
**Tokelau**	0.6864	Middle
**Azerbaijan**	0.6949	Middle
**Iran (Islamic Republic of)**	0.6972	Middle
**Costa Rica**	0.7003	Middle
**Sri Lanka**	0.7015	Middle
**Armenia**	0.7018	Middle
**Albania**	0.7068	Middle
**Panama**	0.7089	Middle
**Turkey**	0.7127	Middle
**Mauritius**	0.7183	Middle
**Uruguay**	0.7193	Middle
**China**	0.7216	Middle
**Bosnia and Herzegovina**	0.7231	High-middle
**Argentina**	0.7231	High-middle
**American Samoa**	0.7237	High-middle
**Kazakhstan**	0.7251	High-middle
**Jordan**	0.7253	High-middle
**Libya**	0.7258	High-middle
**Niue**	0.7262	High-middle
**Seychelles**	0.7302	High-middle
**Republic of Moldova**	0.7322	High-middle
**Georgia**	0.7325	High-middle
**Malaysia**	0.7425	High-middle
**Portugal**	0.7442	High-middle
**Lebanon**	0.7447	High-middle
**Barbados**	0.7467	High-middle
**Dominica**	0.747	High-middle
**Antigua and Barbuda**	0.7499	High-middle
**North Macedonia**	0.7506	High-middle
**Bahrain**	0.753	High-middle
**Palau**	0.754	High-middle
**Saint Kitts and Nevis**	0.755	High-middle

**Ukraine**	0.7608	High-middle
**Bulgaria**	0.7682	High-middle
**Romania**	0.7685	High-middle
**Trinidad and Tobago**	0.7688	High-middle
**Spain**	0.7693	High-middle
**Chile**	0.7715	High-middle
**Northern Mariana Islands**	0.7715	High-middle
**Oman**	0.7734	High-middle
**Cook Islands**	0.7791	High-middle
**Belarus**	0.7845	High-middle
**Hungary**	0.7908	High-middle
**Greece**	0.7919	High-middle
**Serbia**	0.7924	High-middle
**Montenegro**	0.7958	High-middle
**Croatia**	0.7983	High-middle
**Malta**	0.8016	High-middle
**Guam**	0.804	High-middle
**Bahamas**	0.805	High-middle
**Italy**	0.8058	High-middle
**Russian Federation**	0.8085	High-middle
**Israel**	0.809	High-middle
**Brunei Darussalam**	0.8102	High
**Slovakia**	0.8106	High
**Poland**	0.812	High
**Saudi Arabia**	0.8151	High
**Bermuda**	0.8214	High
**United States Virgin Islands**	0.8218	High
**Puerto Rico**	0.8255	High
**Greenland**	0.8262	High
**Czechia**	0.8285	High
**Latvia**	0.8307	High
**Cyprus**	0.8356	High
**France**	0.8384	High
**Slovenia**	0.8424	High
**Australia**	0.8443	High
**Estonia**	0.8449	High

**Kuwait**	0.8467	High
**Qatar**	0.8469	High
**United Arab Emirates**	0.8493	High
**New Zealand**	0.8494	High
**Belgium**	0.8537	High
**Austria**	0.8538	High
**Singapore**	0.8561	High
**Lithuania**	0.8565	High
**United Kingdom**	0.859	High
**Finland**	0.8598	High
**United States of America**	0.8624	High
**Andorra**	0.8694	High
**Japan**	0.8712	High
**Canada**	0.8732	High
**Ireland**	0.8738	High
**Taiwan (Province of China)**	0.8747	High
**Iceland**	0.8764	High
**Luxembourg**	0.8844	High
**Republic of Korea**	0.8867	High
**Sweden**	0.8869	High
**San Marino**	0.888	High
**Netherlands**	0.8885	High
**Denmark**	0.8964	High
**Germany**	0.903	High
**Monaco**	0.9083	High
**Norway**	0.9161	High
**Switzerland**	0.9331	High

Supplementary Table S5 | The ASRs (per 100,000 population) of esophageal cancer deaths, incidence, and DALYs in 1990 worldwide (Generated from data available from http://ghdx.healthdata.org/gbd-results-tool)

Location	***ASDR***			***ASIR***			***ASDALYR***		
	val	upper	lower	val	upper	lower	val	upper	lower
China	26.06	30.10	21.77	24.80	28.73	20.71	653.31	758.88	543.18
Democratic People's Republic of Korea	10.99	14.31	8.11	10.49	13.72	7.67	283.37	378.62	203.89
Taiwan (Province of China)	8.90	9.40	8.44	9.05	9.58	8.61	233.26	247.00	221.89
Cambodia	4.48	5.55	3.46	4.23	5.28	3.28	118.87	149.47	92.05
Indonesia	1.95	2.39	1.47	1.84	2.24	1.38	50.90	61.80	37.89
Lao People's Democratic Republic	4.60	6.12	3.30	4.34	5.83	3.07	124.04	167.07	88.60
Malaysia	2.76	3.19	2.32	2.61	3.04	2.18	68.38	79.31	57.28
Maldives	3.20	4.06	2.38	3.04	3.87	2.23	84.30	108.35	59.82
Myanmar	4.14	5.27	3.17	3.92	5.00	2.96	111.85	144.67	83.35
Philippines	1.46	1.69	1.29	1.37	1.59	1.21	38.02	44.34	33.47
Sri Lanka	6.03	7.17	5.10	5.67	6.78	4.80	148.16	177.69	125.13
Thailand	5.03	6.72	3.88	4.83	6.45	3.68	131.03	176.00	102.87
Timor-Leste	2.76	3.58	1.99	2.58	3.36	1.85	70.60	93.55	49.61
Viet Nam	2.13	2.78	1.61	2.03	2.66	1.52	55.34	73.59	40.99
Fiji	2.46	3.07	1.97	2.26	2.83	1.81	59.35	74.89	47.16
Kiribati	6.57	8.20	5.18	6.09	7.54	4.80	168.80	209.92	133.19
Marshall Islands	3.00	3.88	2.11	2.77	3.63	1.94	74.80	97.96	51.59
Micronesia (Federated States of)	3.39	4.24	2.69	3.15	3.93	2.48	86.36	110.45	67.35
Papua New Guinea	2.08	3.00	1.46	1.93	2.81	1.33	51.52	74.89	35.86
Samoa	1.35	1.63	1.06	1.26	1.53	1.00	32.79	40.76	25.57
Solomon Islands	2.96	3.85	2.16	2.74	3.60	1.95	74.67	101.06	49.92
Tonga	2.18	2.71	1.74	2.01	2.50	1.60	51.39	63.54	41.12
Vanuatu	2.67	3.51	2.01	2.47	3.26	1.85	66.35	90.05	48.88
Armenia	2.98	3.24	2.72	2.77	3.00	2.54	72.45	78.16	66.54
Azerbaijan	8.93	10.24	7.74	8.33	9.56	7.25	227.43	260.20	197.38
Georgia	2.23	2.56	1.94	2.10	2.40	1.83	56.36	64.47	49.21
Kazakhstan	20.32	21.94	18.80	18.88	20.34	17.51	500.50	537.45	465.90
Kyrgyzstan	8.80	10.00	7.66	8.27	9.44	7.17	229.89	264.05	198.38
Mongolia	25.54	31.41	20.87	23.27	28.49	18.96	600.56	739.28	490.34
Tajikistan	12.34	14.66	10.23	11.40	13.62	9.40	304.18	365.23	249.15
Turkmenistan	31.58	33.60	29.53	29.45	31.34	27.59	793.70	842.70	742.55
Uzbekistan	15.10	16.50	13.64	14.18	15.51	12.85	388.45	426.79	351.91
Albania	2.05	2.45	1.71	1.88	2.25	1.58	48.87	58.74	40.84
Bosnia and Herzegovina	2.45	2.88	2.08	2.31	2.71	1.96	63.47	74.97	54.41
Bulgaria	2.63	2.83	2.46	2.51	2.69	2.35	70.23	75.44	65.64
Croatia	3.81	4.08	3.56	3.76	4.01	3.51	101.29	108.65	94.13
Czechia	2.90	3.19	2.65	2.85	3.12	2.61	77.90	85.33	71.25
Hungary	4.26	4.55	3.95	4.20	4.50	3.90	128.98	138.43	119.34
North Macedonia	1.50	1.73	1.31	1.41	1.64	1.24	38.65	45.33	33.62
Montenegro	2.00	2.48	1.65	1.96	2.41	1.61	55.12	67.48	45.34

Poland	3.78	3.91	3.63	3.54	3.66	3.40	99.09	102.18	95.77
Romania	1.56	1.66	1.48	1.49	1.59	1.41	43.51	46.47	40.92
Serbia	2.52	3.36	1.92	2.37	3.17	1.80	64.34	87.42	48.13
Slovakia	4.14	5.32	3.27	4.13	5.32	3.23	120.37	157.57	92.09
Slovenia	3.43	3.65	3.22	3.42	3.64	3.21	93.81	100.27	87.64
Belarus	2.91	3.16	2.67	2.86	3.10	2.63	81.19	88.02	75.21
Estonia	3.38	3.62	3.15	3.32	3.56	3.09	91.85	98.88	84.92
Latvia	3.13	3.36	2.91	3.05	3.28	2.82	86.01	92.72	79.21
Lithuania	3.22	3.44	3.02	3.19	3.41	2.98	88.39	94.34	82.73
Republic of Moldova	2.56	2.70	2.41	2.46	2.59	2.32	68.16	71.99	64.00
Russian Federation	5.14	5.23	5.02	4.99	5.08	4.88	136.17	138.67	133.43
Ukraine	3.18	3.40	2.99	3.36	3.59	3.15	91.80	98.63	85.83
Brunei Darussalam	3.20	3.86	2.55	3.15	3.82	2.50	75.66	92.82	59.98
Japan	4.99	5.12	4.73	6.59	6.80	6.28	118.07	120.60	113.93
Republic of Korea	5.80	6.98	4.72	6.02	7.28	4.91	148.53	180.03	120.20
Singapore	4.30	4.57	4.05	4.71	5.02	4.42	102.43	109.17	96.45
Australia	4.24	4.51	4.00	4.35	4.62	4.10	97.45	103.33	91.92
New Zealand	4.82	5.11	4.51	5.09	5.39	4.76	105.92	111.83	99.66
Andorra	1.93	2.75	1.34	2.00	2.87	1.39	48.79	70.61	33.29
Austria	2.57	2.71	2.43	2.67	2.82	2.52	67.10	71.20	63.07
Belgium	4.06	4.31	3.80	4.10	4.35	3.84	101.20	107.16	95.31
Cyprus	1.56	1.90	1.26	1.43	1.75	1.16	31.56	38.38	25.72
Denmark	4.84	5.12	4.59	4.82	5.08	4.57	121.96	128.49	115.50
Finland	3.34	3.53	3.14	3.69	3.87	3.47	79.66	83.41	75.91
France	7.96	8.43	7.48	8.43	8.94	7.92	216.10	229.80	203.46
Germany	3.35	3.53	3.16	3.58	3.79	3.39	92.32	98.16	86.78
Greece	2.27	2.39	2.12	2.25	2.37	2.11	49.29	51.86	46.57
Iceland	4.53	4.82	4.19	4.71	5.03	4.37	107.79	115.44	100.13
Ireland	7.92	8.44	7.40	7.77	8.28	7.29	179.70	191.24	169.37
Israel	2.10	2.25	1.94	2.00	2.13	1.85	46.08	49.15	43.36
Italy	3.32	3.43	3.15	3.34	3.45	3.17	82.26	84.94	79.19
Luxembourg	4.89	5.14	4.61	4.87	5.14	4.59	122.96	130.07	115.87
Malta	2.92	3.15	2.70	2.87	3.10	2.66	68.93	74.69	63.81
Netherlands	4.82	5.08	4.50	4.79	5.05	4.47	114.56	120.99	108.27
Norway	2.56	2.65	2.45	2.63	2.72	2.51	61.12	63.18	59.14
Portugal	4.88	5.16	4.57	4.64	4.92	4.35	118.92	126.43	112.03
Spain	4.15	4.38	3.90	4.28	4.52	4.03	109.78	116.07	103.33
Sweden	2.69	2.82	2.52	2.76	2.90	2.61	60.57	63.51	57.53
Switzerland	4.38	4.62	4.14	4.58	4.83	4.31	109.73	116.11	103.02
United Kingdom	7.35	7.51	7.06	7.30	7.45	7.03	168.03	170.78	163.68
Argentina	7.52	7.96	7.12	6.98	7.37	6.61	175.58	185.25	166.22
Chile	8.12	8.55	7.64	7.42	7.80	6.99	173.88	182.72	164.81
Uruguay	8.97	9.49	8.41	8.39	8.87	7.87	206.97	220.05	194.57
Canada	3.53	3.74	3.31	3.79	4.01	3.56	83.43	88.06	78.87
United States of America	3.75	3.84	3.57	4.16	4.25	3.97	95.83	97.55	92.74
Antigua and Barbuda	3.51	3.75	3.26	3.28	3.51	3.05	83.79	89.39	78.07

Bahamas	6.65	7.25	6.11	6.40	6.97	5.88	179.95	196.85	164.40
Barbados	6.08	6.47	5.71	5.70	6.08	5.36	146.57	156.58	137.37
Belize	2.07	2.21	1.93	1.94	2.07	1.81	51.42	55.10	48.05
Cuba	3.95	4.17	3.74	3.71	3.92	3.52	92.20	97.36	87.44
Dominica	5.24	6.19	4.52	4.83	5.68	4.17	122.57	144.72	104.64
Dominican Republic	2.06	2.48	1.69	1.89	2.29	1.56	49.95	60.75	40.42
Grenada	6.78	7.55	6.08	6.41	7.13	5.73	176.76	197.80	157.85
Guyana	2.24	2.48	2.00	2.10	2.33	1.87	57.26	64.15	50.98
Haiti	5.63	7.21	4.12	5.23	6.74	3.80	143.74	187.88	102.63
Jamaica	3.12	3.35	2.91	2.91	3.13	2.71	72.98	78.47	67.92
Saint Lucia	5.54	5.89	5.23	5.11	5.44	4.82	134.25	143.14	126.24
Saint Vincent and the Grenadines	2.47	2.67	2.26	2.31	2.48	2.12	61.23	66.44	56.16
Suriname	1.86	2.15	1.61	1.74	2.01	1.50	47.04	54.30	40.21
Trinidad and Tobago	2.51	2.65	2.37	2.33	2.46	2.21	61.50	65.12	58.06
Bolivia (Plurinational State of)	3.06	3.88	2.35	2.78	3.53	2.13	70.78	90.88	53.88
Ecuador	2.27	2.41	2.11	2.03	2.16	1.90	50.08	52.82	46.98
Peru	1.91	2.29	1.58	1.72	2.07	1.42	42.46	51.78	34.83
Colombia	4.45	4.69	4.18	4.06	4.28	3.83	101.21	106.16	96.24
Costa Rica	2.65	2.84	2.44	2.47	2.65	2.28	59.94	64.07	55.43
El Salvador	2.00	2.24	1.77	1.83	2.04	1.63	47.60	52.96	42.67
Guatemala	2.89	3.01	2.77	2.56	2.66	2.45	63.30	65.80	60.73
Honduras	1.10	1.30	0.91	1.00	1.19	0.83	26.18	31.23	21.55
Mexico	2.48	2.53	2.38	2.21	2.26	2.13	53.72	54.73	52.44
Nicaragua	1.12	1.27	0.97	1.02	1.15	0.87	25.71	29.05	22.02
Panama	2.02	2.16	1.87	1.84	1.97	1.71	44.50	47.50	41.67
Venezuela (Bolivarian Republic of)	2.74	2.87	2.58	2.50	2.62	2.36	62.42	65.51	59.10
Brazil	7.14	7.37	6.77	6.71	6.93	6.39	183.52	188.74	176.81
Paraguay	3.35	4.06	2.77	3.12	3.75	2.59	82.18	99.24	68.75
Algeria	0.82	0.97	0.67	0.74	0.88	0.61	18.22	21.72	14.88
Bahrain	4.22	4.90	3.64	3.84	4.45	3.29	90.46	105.13	76.76
Egypt	1.29	1.57	1.11	1.18	1.43	1.02	31.36	36.69	27.39
Iran (Islamic Republic of)	5.47	6.14	4.65	5.02	5.64	4.28	126.69	141.56	108.34
Iraq	1.25	1.56	0.98	1.18	1.49	0.92	32.28	40.91	25.03
Jordan	1.19	1.46	0.94	1.12	1.37	0.88	28.83	35.83	22.64
Kuwait	1.86	2.03	1.69	1.82	1.98	1.66	44.60	48.49	40.96
Lebanon	1.37	1.73	1.08	1.28	1.64	1.01	32.88	41.80	25.63
Libya	1.48	1.97	1.08	1.39	1.87	1.02	36.80	49.75	26.81
Morocco	0.87	1.06	0.68	0.81	1.00	0.63	21.48	26.50	16.75
Palestine	1.50	1.93	1.12	1.37	1.76	1.01	33.29	43.63	24.42
Oman	2.03	2.71	1.48	1.92	2.54	1.39	49.98	66.88	36.00
Qatar	5.53	6.84	4.40	5.03	6.22	3.98	114.93	143.11	90.52
Saudi Arabia	2.32	3.07	1.70	2.15	2.87	1.56	54.80	73.76	39.31
Syrian Arab Republic	0.92	1.14	0.73	0.85	1.06	0.67	22.00	27.57	17.12
Tunisia	0.71	0.88	0.57	0.66	0.82	0.53	16.52	20.36	13.18
Turkey	2.56	3.08	2.11	2.39	2.87	1.95	64.08	77.56	51.68
United Arab Emirates	2.56	3.40	1.90	2.38	3.19	1.75	60.25	81.00	44.32

Yemen	5.37	7.71	3.05	5.01	7.18	2.86	135.25	193.01	78.31
Afghanistan	9.91	14.04	4.94	9.32	13.26	4.62	261.91	382.36	124.81
Bangladesh	5.44	7.19	3.88	5.14	6.81	3.66	146.09	197.09	104.47
Bhutan	5.34	7.22	3.78	5.01	6.79	3.58	139.78	189.61	100.36
India	3.68	4.65	3.18	3.49	4.38	3.02	99.41	124.39	86.45
Nepal	5.13	6.62	3.89	4.82	6.22	3.66	135.13	176.70	102.53
Pakistan	6.60	7.57	5.49	6.16	7.06	5.18	167.82	193.56	141.76
Angola	12.44	16.54	8.69	11.66	15.62	8.07	326.22	440.80	220.63
Central African Republic	14.66	17.78	10.37	13.81	16.94	9.73	392.40	483.74	274.19
Congo	16.70	21.64	12.72	15.68	20.39	11.83	439.79	577.56	325.80
Democratic Republic of the Congo	10.37	13.65	7.44	9.63	12.61	6.88	262.82	350.30	183.46
Equatorial Guinea	13.97	18.16	10.01	13.15	17.19	9.28	371.13	487.55	257.16
Gabon	13.19	16.50	9.87	12.32	15.45	9.23	337.16	426.97	251.22
Burundi	20.27	25.34	14.65	19.07	23.80	13.76	536.74	675.73	383.17
Comoros	17.74	23.24	12.84	16.61	21.70	11.98	458.77	611.41	323.17
Djibouti	15.76	21.07	11.49	14.72	19.93	10.59	401.33	552.42	283.99
Eritrea	21.36	27.90	14.31	20.19	26.41	13.46	578.22	764.92	380.21
Ethiopia	10.06	13.03	7.10	9.42	12.21	6.65	262.78	339.39	183.93
Kenya	9.58	13.92	6.90	8.97	12.97	6.42	242.78	348.16	175.43
Madagascar	15.40	18.62	11.33	14.45	17.47	10.71	401.58	489.61	294.99
Malawi	22.60	27.08	18.55	21.08	25.27	17.24	584.02	700.61	479.77
Mauritius	3.76	3.96	3.56	3.61	3.80	3.42	95.65	100.54	90.86
Mozambique	8.35	10.10	6.68	7.58	9.25	5.99	192.17	236.15	153.19
Rwanda	22.38	28.06	15.44	21.07	26.51	14.53	596.51	751.87	407.81
Seychelles	5.59	6.56	4.73	5.41	6.38	4.54	153.16	181.98	127.60
Somalia	21.52	28.90	15.20	20.31	27.19	14.23	575.46	782.86	400.10
United Republic of Tanzania	17.19	21.35	12.96	16.05	20.06	12.16	438.82	549.77	327.37
Uganda	15.62	19.03	12.53	14.52	17.73	11.61	391.45	485.10	309.40
Zambia	20.60	25.52	16.02	19.30	23.88	14.97	535.95	662.05	408.88
Botswana	12.84	16.85	9.28	12.02	15.78	8.68	330.63	447.50	236.54
Lesotho	10.92	14.02	8.51	10.20	13.05	7.89	277.58	357.19	213.01
Namibia	2.49	3.00	2.07	2.35	2.85	1.95	65.41	80.22	53.15
South Africa	11.75	14.28	10.35	11.16	13.42	9.82	322.87	380.88	286.32
Eswatini	17.39	21.76	12.95	16.40	20.70	12.26	457.53	581.34	339.53
Zimbabwe	13.54	16.14	10.92	12.58	15.06	10.21	333.04	399.56	265.98
Benin	3.37	4.09	2.81	3.14	3.80	2.58	84.44	102.55	69.36
Burkina Faso	3.48	4.31	2.70	3.22	4.01	2.47	86.25	107.78	65.40
Cameroon	3.80	4.74	2.97	3.53	4.41	2.73	95.07	119.96	72.19
Cabo Verde	9.39	11.11	7.75	8.69	10.29	7.21	226.31	266.42	191.19
Chad	2.73	3.30	2.22	2.53	3.07	2.05	67.98	82.53	54.73
Côte d'Ivoire	1.39	1.65	1.15	1.29	1.54	1.06	34.79	42.08	27.84
Gambia	1.60	2.02	1.25	1.50	1.88	1.16	40.26	50.99	30.78
Ghana	2.59	3.26	2.01	2.41	3.02	1.87	65.06	83.15	49.68
Guinea	1.29	1.58	1.01	1.20	1.47	0.93	32.31	39.97	25.39
Guinea-Bissau	5.01	6.23	3.78	4.71	5.87	3.55	131.96	166.91	97.58
Liberia	3.33	4.07	2.66	3.08	3.81	2.44	82.31	102.16	64.48

Mali	2.59	3.03	2.20	2.41	2.83	2.03	65.48	77.26	55.03
Mauritania	3.81	4.91	2.95	3.54	4.54	2.74	94.91	121.28	71.89
Niger	3.01	3.76	2.30	2.80	3.49	2.11	75.41	95.12	56.69
Nigeria	2.86	3.80	2.21	2.64	3.53	2.04	70.90	96.33	54.16
Sao Tome and Principe	2.49	3.05	2.00	2.31	2.85	1.84	61.40	76.95	48.23
Senegal	3.25	3.89	2.64	3.02	3.64	2.45	81.55	99.27	65.56
Sierra Leone	3.00	3.69	2.35	2.78	3.43	2.19	74.50	93.79	57.13
Togo	3.23	3.97	2.59	3.01	3.72	2.39	81.08	101.20	63.99
American Samoa	1.30	1.55	1.09	1.19	1.44	1.01	30.78	37.44	25.63
Bermuda	7.32	7.88	6.74	6.98	7.54	6.41	176.39	190.78	162.35
Cook Islands	3.17	3.79	2.62	2.94	3.53	2.42	73.56	89.23	59.53
Greenland	17.01	19.72	14.66	16.31	18.85	14.01	437.48	506.61	374.32
Guam	2.49	2.83	2.14	2.32	2.64	2.00	57.08	65.26	49.27
Monaco	4.92	6.14	3.75	5.12	6.40	3.88	116.38	145.11	89.73
Nauru	4.27	5.50	3.19	3.99	5.17	2.94	109.70	143.94	80.34
Niue	2.48	3.01	1.99	2.30	2.81	1.85	60.02	74.57	46.91
Northern Mariana Islands	1.47	1.89	1.20	1.37	1.76	1.11	34.36	44.63	27.65
Palau	3.45	4.34	2.78	3.14	3.97	2.51	79.08	100.58	62.52
Puerto Rico	6.44	6.83	6.07	6.06	6.44	5.72	151.96	161.67	143.54
Saint Kitts and Nevis	4.78	5.06	4.48	4.43	4.70	4.15	118.42	125.95	110.68
San Marino	1.61	1.99	1.27	1.67	2.07	1.31	38.56	48.47	30.09
Tokelau	2.34	3.04	1.61	2.14	2.80	1.47	55.38	73.34	37.63
Tuvalu	2.79	3.39	2.22	2.58	3.13	2.05	69.71	85.32	54.67
United States Virgin Islands	3.55	4.25	2.92	3.33	4.00	2.73	86.76	105.05	69.88
South Sudan	18.71	25.25	13.92	17.52	23.53	12.94	481.42	661.88	353.65
Sudan	5.10	6.88	3.20	4.74	6.29	2.99	128.60	173.70	79.43

Supplementary Table S6 | The ASRs (per 100,000 population) of esophageal cancer deaths, incidence, and DALYs in 2021 worldwide

Location	***ASDR***			***ASIR***			***ASDALYR***		
	**val**	**upper**	**lower**	**val**	**upper**	**lower**	**val**	**upper**	**lower**
China	14.13	17.18	11.36	15.04	18.43	12.04	317.18	392.42	252.46
Democratic People's Republic of Korea	9.13	11.98	6.80	9.19	12.05	6.86	241.08	322.41	176.36
Taiwan (Province of China)	7.45	8.07	6.72	9.30	10.06	8.41	219.55	237.21	198.14
Cambodia	3.12	4.07	2.32	2.96	3.87	2.17	77.76	102.65	56.89
Indonesia	1.71	2.14	1.32	1.62	2.03	1.25	41.44	52.06	31.71
Lao People's Democratic Republic	2.64	3.63	1.99	2.48	3.42	1.86	66.11	92.57	48.58
Malaysia	2.69	3.11	2.30	2.65	3.08	2.25	64.90	76.09	54.96
Maldives	1.22	1.49	0.97	1.21	1.48	0.95	27.83	34.75	21.73
Myanmar	2.38	3.22	1.83	2.25	3.04	1.75	59.51	81.41	45.59
Philippines	1.39	1.66	1.15	1.34	1.60	1.10	36.37	43.52	29.66
Sri Lanka	4.16	5.69	2.76	4.26	5.80	2.82	101.75	140.49	65.82
Thailand	4.44	5.75	3.33	4.72	6.18	3.56	124.84	164.75	92.39
Timor-Leste	2.23	3.05	1.65	2.09	2.84	1.54	54.95	76.40	39.45
Viet Nam	2.30	2.90	1.75	2.33	2.94	1.77	60.59	77.89	44.43
Fiji	2.86	3.72	2.01	2.63	3.42	1.88	66.24	86.77	46.72
Kiribati	5.96	7.64	4.23	5.52	7.07	3.95	150.72	197.39	108.34
Marshall Islands	2.67	3.54	2.03	2.48	3.33	1.86	66.78	89.36	49.64
Micronesia (Federated States of)	2.85	3.72	2.22	2.67	3.50	2.06	72.06	95.92	54.08
Papua New Guinea	1.72	2.43	1.20	1.58	2.24	1.11	41.07	58.69	28.72
Samoa	1.17	1.47	0.92	1.12	1.42	0.88	28.91	36.88	22.24
Solomon Islands	2.58	3.55	1.81	2.40	3.33	1.65	65.57	91.53	44.23
Tonga	2.12	2.74	1.63	1.98	2.57	1.52	49.66	64.90	37.64
Vanuatu	2.39	3.05	1.88	2.21	2.82	1.73	59.09	76.54	45.86
Armenia	1.08	1.24	0.94	1.00	1.15	0.88	24.94	28.62	21.88
Azerbaijan	5.79	7.43	4.25	5.35	6.87	3.96	135.65	174.28	99.95
Georgia	1.13	1.33	0.96	1.06	1.25	0.89	27.43	32.09	23.33
Kazakhstan	5.02	5.80	4.34	4.69	5.40	4.04	119.34	137.70	103.25
Kyrgyzstan	3.03	3.64	2.44	2.82	3.44	2.25	72.51	88.94	56.99
Mongolia	17.98	21.49	14.50	16.25	19.45	13.15	397.98	481.22	317.66
Tajikistan	6.77	8.66	5.07	6.13	7.87	4.58	153.52	197.77	113.81
Turkmenistan	8.98	11.48	6.94	8.40	10.73	6.42	223.55	288.61	170.85
Uzbekistan	3.98	4.76	3.31	3.71	4.42	3.09	98.60	118.15	81.21
Albania	1.52	2.00	1.11	1.41	1.85	1.03	35.34	46.37	25.92
Bosnia and Herzegovina	2.22	2.82	1.65	2.13	2.71	1.59	56.57	71.86	41.56
Bulgaria	2.30	2.63	1.98	2.26	2.60	1.93	62.88	72.67	53.55
Croatia	2.58	2.91	2.24	2.67	3.03	2.33	66.80	75.87	58.14
Czechia	3.02	3.48	2.59	3.23	3.71	2.75	77.31	89.43	65.69
Hungary	3.19	3.60	2.81	3.17	3.59	2.80	87.03	98.84	76.55
North Macedonia	1.46	1.81	1.14	1.37	1.74	1.06	35.28	45.06	26.69
Montenegro	2.29	2.92	1.78	2.22	2.85	1.71	57.88	74.13	44.64
Poland	3.14	3.42	2.84	2.98	3.25	2.69	80.97	88.34	73.46

Romania	2.54	2.89	2.24	2.52	2.86	2.20	72.12	82.08	63.27
Serbia	2.17	2.98	1.57	2.14	2.94	1.54	56.21	78.21	40.26
Slovakia	3.21	4.27	2.47	3.25	4.33	2.47	87.88	121.55	65.22
Slovenia	2.21	2.49	1.88	2.44	2.76	2.08	55.03	62.73	46.46
Belarus	2.86	3.55	2.32	3.05	3.75	2.50	81.52	101.55	65.90
Estonia	2.58	2.99	2.21	2.75	3.18	2.35	65.92	77.00	56.24
Latvia	2.90	3.33	2.49	2.91	3.33	2.50	78.33	90.89	66.70
Lithuania	3.56	4.08	3.06	3.68	4.21	3.17	97.28	111.39	83.82
Republic of Moldova	1.84	2.05	1.65	1.86	2.09	1.67	50.04	55.89	44.81
Russian Federation	3.22	3.49	2.93	3.33	3.61	3.01	86.83	94.59	78.57
Ukraine	2.21	3.08	1.52	2.45	3.42	1.68	66.15	92.64	44.54
Brunei Darussalam	2.15	2.60	1.72	2.26	2.73	1.81	50.19	61.35	40.09
Japan	3.81	3.99	3.49	6.22	6.48	5.73	84.34	87.57	78.99
Republic of Korea	2.29	2.81	1.83	3.52	4.31	2.81	50.15	61.89	40.36
Singapore	1.71	1.84	1.53	2.59	2.81	2.33	37.68	40.64	34.22
Australia	3.75	4.03	3.37	4.06	4.37	3.67	82.27	88.24	75.67
New Zealand	3.33	3.59	3.03	4.04	4.36	3.69	69.67	74.50	64.87
Andorra	1.18	1.69	0.76	1.35	1.95	0.87	28.60	41.58	18.34
Austria	2.31	2.48	2.11	2.67	2.87	2.44	55.39	59.59	50.91
Belgium	4.11	4.44	3.77	4.59	4.95	4.23	97.70	105.10	90.31
Cyprus	1.37	1.72	1.08	1.47	1.85	1.15	29.77	37.84	23.23
Denmark	4.70	5.05	4.26	5.10	5.46	4.64	106.78	114.86	97.80
Finland	2.82	3.02	2.58	3.62	3.86	3.32	66.18	70.66	61.74
France	3.65	3.97	3.30	4.99	5.41	4.54	88.64	95.95	80.73
Germany	3.69	3.93	3.39	4.60	4.91	4.21	90.66	96.82	84.22
Greece	1.66	1.76	1.54	1.82	1.93	1.69	40.62	43.13	38.21
Iceland	3.85	4.27	3.41	4.53	5.05	4.04	89.55	99.09	80.22
Ireland	5.39	5.96	4.76	6.13	6.74	5.45	120.15	131.85	108.17
Israel	1.47	1.58	1.32	1.54	1.67	1.41	32.43	34.82	29.73
Italy	1.61	1.69	1.48	1.77	1.87	1.64	37.13	38.91	35.08
Luxembourg	3.35	3.70	3.02	3.73	4.13	3.37	78.00	86.25	70.60
Malta	2.17	2.41	1.93	2.41	2.68	2.15	52.06	57.45	46.47
Netherlands	6.21	6.69	5.63	6.88	7.40	6.30	137.08	147.01	126.38
Norway	2.92	3.07	2.70	3.32	3.50	3.09	64.09	67.04	60.46
Portugal	3.24	3.52	2.96	3.44	3.73	3.14	85.41	92.66	78.58
Spain	2.40	2.60	2.18	2.81	3.03	2.57	60.24	65.15	55.22
Sweden	2.62	2.93	2.28	2.86	3.19	2.50	56.48	63.53	49.49
Switzerland	2.84	3.07	2.54	3.22	3.49	2.90	64.24	69.19	58.66
United Kingdom	7.16	7.44	6.69	7.54	7.83	7.08	154.57	159.75	147.55
Argentina	4.34	4.69	4.01	4.13	4.47	3.82	97.43	105.34	90.62
Chile	3.18	3.44	2.87	3.07	3.31	2.79	63.99	68.78	58.76
Uruguay	5.64	6.11	5.19	5.45	5.90	5.00	126.68	136.86	116.38
Canada	3.55	3.82	3.27	4.20	4.51	3.87	82.27	88.31	76.51
United States of America	3.62	3.76	3.40	4.20	4.36	3.95	86.55	89.36	82.92
Antigua and Barbuda	2.99	3.19	2.81	2.83	3.02	2.65	68.17	73.05	63.54
Bahamas	5.33	6.63	4.25	5.13	6.40	4.06	139.84	175.88	110.50

Barbados	5.28	6.44	4.17	5.01	6.18	3.94	123.56	153.38	95.66
Belize	2.24	2.55	1.95	2.13	2.43	1.85	56.64	64.71	48.89
Cuba	4.95	5.75	4.20	4.97	5.72	4.22	128.61	149.64	108.42
Dominica	5.24	6.60	4.24	4.91	6.27	3.95	127.81	167.10	101.27
Dominican Republic	2.30	2.96	1.74	2.17	2.80	1.64	58.46	75.31	43.94
Grenada	4.84	5.60	4.12	4.63	5.32	3.93	122.78	143.29	103.70
Guyana	2.15	2.74	1.63	2.04	2.62	1.56	57.17	73.71	43.20
Haiti	4.26	5.88	3.02	3.95	5.50	2.78	106.31	150.33	74.13
Jamaica	3.13	4.01	2.36	2.97	3.84	2.25	75.25	98.34	55.67
Saint Lucia	4.38	5.25	3.59	4.14	4.98	3.39	108.84	131.40	87.96
Saint Vincent and the Grenadines	2.62	2.97	2.30	2.47	2.81	2.17	66.24	76.24	58.15
Suriname	1.69	2.16	1.26	1.61	2.08	1.20	44.12	56.74	32.81
Trinidad and Tobago	2.01	2.54	1.55	1.93	2.44	1.47	51.79	66.65	39.32
Bolivia (Plurinational State of)	2.45	3.27	1.82	2.21	2.95	1.64	52.85	71.77	38.37
Ecuador	1.32	1.64	1.05	1.19	1.49	0.94	27.39	34.22	21.40
Peru	1.36	1.79	1.02	1.27	1.68	0.94	29.10	38.92	21.30
Colombia	1.88	2.25	1.56	1.77	2.10	1.47	40.93	49.21	33.94
Costa Rica	1.63	1.83	1.42	1.56	1.75	1.37	36.83	41.46	32.09
El Salvador	1.78	2.24	1.42	1.69	2.13	1.34	42.50	53.92	33.81
Guatemala	1.81	2.12	1.56	1.65	1.95	1.41	40.65	47.76	34.54
Honduras	1.60	2.00	1.26	1.45	1.83	1.14	35.29	45.12	27.38
Mexico	1.53	1.74	1.33	1.43	1.63	1.24	36.05	41.27	31.08
Nicaragua	0.89	1.09	0.72	0.82	1.02	0.66	20.45	25.24	16.57
Panama	1.28	1.55	1.00	1.20	1.46	0.94	28.40	34.44	21.95
Venezuela (Bolivarian Republic of)	1.75	2.32	1.28	1.65	2.19	1.20	41.29	55.37	29.45
Brazil	5.09	5.32	4.80	4.93	5.14	4.65	132.78	138.35	126.26
Paraguay	4.22	5.65	3.14	4.01	5.40	2.99	104.67	139.44	77.00
Algeria	0.75	0.94	0.59	0.70	0.87	0.55	16.36	20.52	12.54
Bahrain	2.31	2.86	1.86	2.17	2.67	1.74	45.61	57.24	36.18
Egypt	1.11	1.39	0.88	1.03	1.30	0.82	25.33	31.93	19.97
Iran (Islamic Republic of)	3.99	4.35	3.58	3.78	4.11	3.40	86.71	94.51	78.90
Iraq	1.23	1.56	0.92	1.18	1.51	0.87	29.09	37.44	20.94
Jordan	0.88	1.12	0.69	0.85	1.08	0.66	20.17	25.91	15.53
Kuwait	0.95	1.17	0.76	0.94	1.15	0.76	19.70	24.31	15.89
Lebanon	0.94	1.14	0.75	0.92	1.13	0.73	20.85	25.70	16.58
Libya	1.76	2.33	1.25	1.70	2.26	1.20	43.79	59.01	30.39
Morocco	0.90	1.12	0.66	0.85	1.06	0.62	21.36	27.30	15.36
Palestine	0.96	1.18	0.78	0.90	1.10	0.73	20.82	25.57	16.83
Oman	1.62	2.04	1.22	1.59	2.00	1.19	36.96	47.50	27.45
Qatar	2.65	3.51	1.98	2.59	3.43	1.93	52.60	70.86	38.83
Saudi Arabia	1.81	2.33	1.43	1.75	2.23	1.38	41.63	54.55	32.40
Syrian Arab Republic	0.95	1.20	0.72	0.89	1.15	0.66	20.90	27.38	15.14
Tunisia	0.69	0.96	0.48	0.67	0.93	0.46	15.83	22.28	10.75
Turkey	1.67	2.12	1.28	1.63	2.07	1.24	38.74	48.69	29.16
United Arab Emirates	2.36	2.93	1.85	2.14	2.67	1.65	46.26	58.38	35.71
Yemen	4.16	5.92	2.19	3.85	5.50	2.03	98.89	142.83	51.16

Afghanistan	7.77	11.29	3.85	7.26	10.67	3.56	197.42	295.41	92.41
Bangladesh	3.48	4.82	2.46	3.27	4.51	2.34	86.66	121.66	61.04
Bhutan	3.82	5.27	2.76	3.56	4.86	2.60	91.87	128.50	65.63
India	3.23	3.89	2.83	3.07	3.69	2.69	83.40	99.11	73.11
Nepal	4.04	5.73	2.89	3.77	5.32	2.71	100.19	142.84	70.67
Pakistan	6.70	8.40	5.36	6.26	7.79	4.97	168.00	212.35	133.54
Angola	8.58	11.20	6.13	7.97	10.37	5.67	212.85	279.88	153.26
Central African Republic	11.46	15.35	7.83	10.72	14.50	7.36	299.91	413.70	203.48
Congo	11.26	14.92	8.25	10.48	14.00	7.65	281.50	380.86	198.91
Democratic Republic of the Congo	8.58	11.65	5.91	7.96	10.74	5.48	213.24	291.29	143.54
Equatorial Guinea	9.42	13.41	6.55	8.78	12.50	6.13	229.69	333.53	155.89
Gabon	11.20	14.38	8.24	10.46	13.41	7.65	277.42	360.92	199.59
Burundi	11.50	14.73	8.29	10.64	13.65	7.73	285.03	369.84	205.13
Comoros	13.47	18.11	10.01	12.50	16.79	9.29	329.86	444.83	242.64
Djibouti	13.06	18.52	8.89	12.11	17.11	8.24	320.56	464.52	213.23
Eritrea	15.60	22.48	10.93	14.47	20.82	10.20	391.03	566.88	270.15
Ethiopia	5.38	7.22	4.35	4.97	6.66	4.01	129.60	172.95	104.33
Kenya	12.69	17.97	9.20	11.75	16.66	8.54	306.12	429.01	222.37
Madagascar	11.59	15.79	7.93	10.79	14.78	7.47	291.34	398.82	199.80
Malawi	27.77	34.72	22.45	26.06	32.46	21.02	715.28	904.41	572.77
Mauritius	3.51	3.70	3.26	3.55	3.75	3.30	92.68	97.68	85.24
Mozambique	9.79	12.22	7.56	8.98	11.19	6.90	232.62	293.13	175.47
Rwanda	12.22	16.33	9.01	11.28	14.89	8.26	295.32	395.44	214.93
Seychelles	4.77	5.76	3.88	4.73	5.73	3.85	124.35	152.45	100.17
Somalia	15.97	21.10	11.10	14.91	19.71	10.25	410.58	546.22	287.39
United Republic of Tanzania	12.24	16.06	9.12	11.36	14.77	8.38	300.14	394.93	220.09
Uganda	15.90	20.93	12.41	14.81	19.57	11.47	395.91	521.13	302.09
Zambia	17.13	26.09	11.78	16.02	24.45	10.95	436.30	677.10	290.09
Botswana	9.22	11.95	7.01	8.59	11.12	6.53	226.60	296.06	168.23
Lesotho	16.67	21.26	12.38	15.82	20.12	11.67	450.01	588.49	327.62
Namibia	2.46	3.19	1.93	2.35	3.08	1.83	65.51	88.59	49.50
South Africa	11.06	12.30	10.06	10.43	11.60	9.45	279.79	313.69	254.98
Eswatini	17.47	23.38	12.43	16.68	22.42	11.80	478.85	665.21	332.56
Zimbabwe	17.02	21.11	13.23	15.93	19.64	12.31	435.15	549.19	329.44
Benin	5.41	7.02	4.05	5.02	6.50	3.75	132.87	174.31	99.51
Burkina Faso	5.87	7.56	4.10	5.44	7.00	3.78	143.94	185.19	100.00
Cameroon	6.71	9.12	4.67	6.27	8.52	4.33	169.45	235.52	115.01
Cabo Verde	15.93	19.70	12.58	15.15	18.91	11.90	398.29	491.88	311.28
Chad	5.85	7.49	4.39	5.44	7.04	4.00	146.17	192.43	107.52
Côte d'Ivoire	1.39	1.84	1.06	1.30	1.73	0.99	34.70	46.77	25.72
Gambia	2.07	2.62	1.58	1.93	2.43	1.49	51.54	65.80	38.88
Ghana	3.80	4.97	2.53	3.51	4.55	2.33	90.52	117.85	61.71
Guinea	1.44	1.94	1.03	1.34	1.80	0.96	36.04	49.30	25.48
Guinea-Bissau	7.80	9.72	5.81	7.31	9.12	5.45	201.13	253.61	149.66
Liberia	6.07	8.44	4.24	5.65	7.88	3.97	150.61	214.96	103.61
Mali	2.63	3.39	2.02	2.45	3.12	1.87	65.07	84.49	49.53

Mauritania	5.62	7.63	4.00	5.21	7.09	3.70	132.93	184.97	93.64
Niger	4.48	5.89	3.08	4.11	5.43	2.84	107.27	143.73	73.68
Nigeria	4.64	6.04	3.22	4.25	5.58	2.92	108.92	146.09	74.14
Sao Tome and Principe	4.93	6.26	3.76	4.62	5.86	3.55	120.42	156.99	91.75
Senegal	5.46	7.05	4.09	5.06	6.54	3.82	132.95	173.99	99.51
Sierra Leone	5.19	6.89	3.75	4.83	6.39	3.47	128.69	174.10	92.70
Togo	6.22	8.23	4.33	5.82	7.74	4.10	157.05	213.04	111.16
American Samoa	1.67	2.01	1.38	1.56	1.89	1.29	39.92	48.77	32.07
Bermuda	4.06	4.84	3.42	4.19	5.03	3.53	95.35	114.35	79.29
Cook Islands	2.54	3.06	2.01	2.54	3.09	1.99	59.74	74.30	46.20
Greenland	11.00	13.72	8.74	10.81	13.41	8.59	272.77	334.96	217.03
Guam	1.84	2.17	1.55	1.88	2.22	1.58	51.70	60.85	43.22
Monaco	5.48	6.71	4.33	6.21	7.70	4.91	127.03	157.38	98.32
Nauru	3.44	4.38	2.58	3.25	4.19	2.41	89.22	115.44	64.21
Niue	2.54	3.10	1.99	2.41	2.95	1.87	60.59	76.26	45.97
Northern Mariana Islands	2.71	3.24	2.22	2.60	3.09	2.14	62.76	73.90	52.12
Palau	3.13	3.88	2.55	2.89	3.58	2.35	71.13	89.51	56.17
Puerto Rico	2.39	2.80	1.95	2.42	2.85	1.98	59.50	70.75	48.62
Saint Kitts and Nevis	4.03	4.66	3.35	3.81	4.44	3.17	97.62	115.04	80.24
San Marino	0.85	1.25	0.53	0.96	1.42	0.60	20.32	30.60	12.09
Tokelau	1.94	2.42	1.46	1.82	2.28	1.37	45.87	58.52	34.06
Tuvalu	2.26	2.83	1.78	2.11	2.63	1.67	55.90	70.42	43.15
United States Virgin Islands	2.42	3.09	1.86	2.32	3.00	1.77	61.04	80.44	46.48
South Sudan	16.21	21.42	11.98	15.16	20.13	11.11	411.79	551.71	298.26
Sudan	4.56	6.31	2.97	4.24	5.95	2.70	108.35	154.98	66.93

Supplementary Table S7 | The percentage change of ASRs (per 100,000 population) of esophageal cancer deaths, incidence, and DALYs from 1990 to 2021 worldwide (Generated from data available from http://ghdx.healthdata.org/gbd-results-tool)

Location	***ASIR***	***ASDR***	***ASDALYR***
China	-0.393501793	-0.457762042	-0.514498473
Democratic People's Republic of Korea	-0.124179445	-0.168888207	-0.149231412
Taiwan (Province of China)	0.027640179	-0.161958528	-0.05875734
Cambodia	-0.300206396	-0.30403543	-0.345834415
Indonesia	-0.12028354	-0.122147308	-0.186018863
Lao People's Democratic Republic	-0.428967459	-0.426377887	-0.466977913
Malaysia	0.012414322	-0.027448855	-0.050958926
Maldives	-0.602414137	-0.618668683	-0.669920673
Myanmar	-0.425595282	-0.42628284	-0.467940653
Philippines	-0.023536898	-0.04702254	-0.043440634
Sri Lanka	-0.248842777	-0.310133232	-0.31325177
Thailand	-0.022776929	-0.118445362	-0.047211023
Timor-Leste	-0.187426535	-0.191116304	-0.221597332
Viet Nam	0.14533558	0.076768728	0.094887164
Fiji	0.159550697	0.162281727	0.11620782
Kiribati	-0.094595868	-0.092001144	-0.107113636
Marshall Islands	-0.103622823	-0.110194993	-0.107302176
Micronesia (Federated States of)	-0.151752162	-0.160636311	-0.165558051
Papua New Guinea	-0.179950284	-0.176869518	-0.202857105
Samoa	-0.110775921	-0.134026024	-0.118230627
Solomon Islands	-0.122084369	-0.128803785	-0.121918828
Tonga	-0.012260942	-0.02794056	-0.033554987
Vanuatu	-0.103443722	-0.105794466	-0.109519221
Armenia	-0.637095407	-0.638794854	-0.655731665
Azerbaijan	-0.357465736	-0.352040676	-0.403539931
Georgia	-0.494254924	-0.492978977	-0.51329033
Kazakhstan	-0.751681217	-0.752889496	-0.761566014
Kyrgyzstan	-0.659082834	-0.6556692	-0.684607465
Mongolia	-0.301668597	-0.295954345	-0.337318277
Tajikistan	-0.462235701	-0.45143151	-0.495283773
Turkmenistan	-0.71485146	-0.715660708	-0.718348019
Uzbekistan	-0.738288997	-0.736635304	-0.746164938
Albania	-0.250338786	-0.260031787	-0.276839199
Bosnia and Herzegovina	-0.077863973	-0.094097497	-0.108800803
Bulgaria	-0.099126449	-0.125445075	-0.104656193
Croatia	-0.289038965	-0.324037329	-0.340451787
Czechia	0.132379194	0.041874206	-0.00762017
Hungary	-0.245529401	-0.251528959	-0.325305438
North Macedonia	-0.028520664	-0.026801854	-0.087158911
Montenegro	0.133614014	0.146165983	0.050173722

Poland	-0.157482004	-0.171288042	-0.182822269
Romania	0.6893263	0.628417638	0.657601223
Serbia	-0.096737078	-0.140442585	-0.126411094
Slovakia	-0.214013979	-0.224937283	-0.269925689
Slovenia	-0.287217313	-0.357118831	-0.413431896
Belarus	0.063203228	-0.017325659	0.004096621
Estonia	-0.171193314	-0.234823856	-0.282316924
Latvia	-0.043951415	-0.073648383	-0.089275909
Lithuania	0.155514154	0.107053718	0.100515412
Republic of Moldova	-0.242014528	-0.281436802	-0.265891803
Russian Federation	-0.333768085	-0.374258198	-0.362343399
Ukraine	-0.269494864	-0.304816152	-0.279367404
Brunei Darussalam	-0.281379383	-0.330086321	-0.336729549
Japan	-0.057024637	-0.234820923	-0.285659098
Republic of Korea	-0.414768331	-0.605320941	-0.66236025
Singapore	-0.450975579	-0.603233275	-0.632108487
Australia	-0.066476277	-0.115826023	-0.155779674
New Zealand	-0.206691352	-0.309999934	-0.342240484
Andorra	-0.325143869	-0.391556421	-0.413890201
Austria	8.77868E-05	-0.101290494	-0.174531965
Belgium	0.120512598	0.012677068	-0.034658842
Cyprus	0.028009569	-0.121750566	-0.056848939
Denmark	0.057729073	-0.029582374	-0.124478313
Finland	-0.019408924	-0.156467942	-0.169224522
France	-0.407948185	-0.541273632	-0.589822739
Germany	0.284323238	0.101892875	-0.017993478
Greece	-0.191861109	-0.266303239	-0.175907094
Iceland	-0.038078452	-0.150532925	-0.169255395
Ireland	-0.210237264	-0.319231092	-0.331380552
Israel	-0.226300806	-0.302706288	-0.296207703
Italy	-0.468569658	-0.515803822	-0.548612729
Luxembourg	-0.233315272	-0.31390539	-0.365693404
Malta	-0.160786426	-0.257055893	-0.244677033
Netherlands	0.438597005	0.289601826	0.196545001
Norway	0.265054752	0.139350476	0.048633942
Portugal	-0.258437221	-0.336282395	-0.281798388
Spain	-0.343213203	-0.420557619	-0.451308843
Sweden	0.035510832	-0.022982543	-0.067608821
Switzerland	-0.29558981	-0.352357692	-0.414586955
United Kingdom	0.033642841	-0.025743227	-0.080058645
Argentina	-0.408718037	-0.422615897	-0.445079367
Chile	-0.585658046	-0.607687312	-0.632001492
Uruguay	-0.351043443	-0.371054191	-0.387922877
Canada	0.108927703	0.006277009	-0.013909424
United States of America	0.011357565	-0.03519048	-0.096795029
Antigua and Barbuda	-0.138063089	-0.146292524	-0.186403312

Bahamas	-0.198887043	-0.198089738	-0.222876227
Barbados	-0.122066998	-0.132801931	-0.156969821
Belize	0.09755145	0.084097568	0.101576532
Cuba	0.339649975	0.254321762	0.39492307
Dominica	0.016716374	-0.000664781	0.042776096
Dominican Republic	0.148506566	0.114552891	0.170565043
Grenada	-0.278201937	-0.285650927	-0.305416555
Guyana	-0.028688631	-0.042415323	-0.001494093
Haiti	-0.246087345	-0.243946555	-0.260402659
Jamaica	0.020281709	0.00065124	0.031123985
Saint Lucia	-0.189079206	-0.208904687	-0.189237197
Saint Vincent and the Grenadines	0.07215453	0.057816259	0.081822514
Suriname	-0.078277703	-0.0939304	-0.062039133
Trinidad and Tobago	-0.174654694	-0.200777733	-0.157955768
Bolivia (Plurinational State of)	-0.204075453	-0.198046139	-0.253346314
Ecuador	-0.414388044	-0.41572716	-0.452956433
Peru	-0.26294776	-0.285225017	-0.314759527
Colombia	-0.565496571	-0.576508581	-0.595578303
Costa Rica	-0.367306927	-0.384888233	-0.385527467
El Salvador	-0.079193113	-0.107362398	-0.107258617
Guatemala	-0.355133709	-0.372615068	-0.35787666
Honduras	0.445906944	0.456871752	0.347909729
Mexico	-0.351738467	-0.382108532	-0.329019593
Nicaragua	-0.191172586	-0.210193311	-0.2048067
Panama	-0.345149543	-0.364735708	-0.361769326
Venezuela (Bolivarian Republic of)	-0.341190739	-0.359250109	-0.338521278
Brazil	-0.266035948	-0.286233564	-0.276488679
Paraguay	0.285717782	0.258838015	0.273562068
Algeria	-0.057136887	-0.082674193	-0.101845443
Bahrain	-0.434180497	-0.451897931	-0.495798276
Egypt	-0.125960092	-0.138452886	-0.192338433
Iran (Islamic Republic of)	-0.248368791	-0.270589611	-0.315598496
Iraq	-0.004396844	-0.012565651	-0.098812055
Jordan	-0.235722993	-0.257907826	-0.300411048
Kuwait	-0.48421878	-0.491468683	-0.558182384
Lebanon	-0.285685474	-0.315106811	-0.365733597
Libya	0.218850578	0.193235506	0.189999924
Morocco	0.04653062	0.036247587	-0.005407402
Palestine	-0.343431205	-0.357909674	-0.374681279
Oman	-0.175306088	-0.200648255	-0.260404018
Qatar	-0.484729147	-0.522071743	-0.542347384
Saudi Arabia	-0.185630914	-0.222076833	-0.240343222
Syrian Arab Republic	0.050996548	0.035922084	-0.049770082
Tunisia	0.003986597	-0.038534497	-0.041928106
Turkey	-0.318121106	-0.345934187	-0.395388637
United Arab Emirates	-0.098325973	-0.078183033	-0.232212938

Yemen	-0.232779964	-0.226160472	-0.268847719
Afghanistan	-0.220810671	-0.2158866	-0.246231611
Bangladesh	-0.362737975	-0.359065761	-0.406806646
Bhutan	-0.29026902	-0.284051791	-0.342719187
India	-0.11938336	-0.124324241	-0.160970069
Nepal	-0.216405584	-0.211764499	-0.258602519
Pakistan	0.016059795	0.015421762	0.001094897
Angola	-0.316777537	-0.310428029	-0.347530885
Central African Republic	-0.223750198	-0.2182478	-0.235698492
Congo	-0.331360138	-0.325573944	-0.359916847
Democratic Republic of the Congo	-0.173204957	-0.172632485	-0.18864224
Equatorial Guinea	-0.332387161	-0.32533475	-0.381116035
Gabon	-0.150825291	-0.150842486	-0.177169983
Burundi	-0.44204092	-0.432673759	-0.468958785
Comoros	-0.247523556	-0.240788259	-0.280986804
Djibouti	-0.177178439	-0.171537532	-0.201234785
Eritrea	-0.283224906	-0.269636538	-0.323737492
Ethiopia	-0.472395459	-0.464822303	-0.506804215
Kenya	0.309352363	0.324213387	0.260868356
Madagascar	-0.253330786	-0.247315976	-0.27452638
Malawi	0.236723205	0.229188858	0.224745896
Mauritius	-0.01729896	-0.068373914	-0.031023529
Mozambique	0.184259818	0.172215095	0.210530219
Rwanda	-0.464597563	-0.45406587	-0.504930064
Seychelles	-0.125981226	-0.147004548	-0.188076997
Somalia	-0.265980101	-0.258291797	-0.286507553
United Republic of Tanzania	-0.292528861	-0.288010929	-0.316017858
Uganda	0.0199509	0.017991015	0.011398873
Zambia	-0.169803484	-0.168722936	-0.185923812
Botswana	-0.285967035	-0.281441337	-0.314636577
Lesotho	0.550559115	0.526494015	0.62120884
Namibia	0.00164724	-0.00965764	0.001512248
South Africa	-0.065815475	-0.058679033	-0.133430379
Eswatini	0.017196708	0.004857429	0.046593634
Zimbabwe	0.265854278	0.257360937	0.306586141
Benin	0.600509312	0.603285283	0.57345639
Burkina Faso	0.686543609	0.687651747	0.668926965
Cameroon	0.775167846	0.765266644	0.782395132
Cabo Verde	0.74444361	0.696588038	0.759950325
Chad	1.151538755	1.147557964	1.150298542
Côte d'Ivoire	0.006191697	0.002932505	-0.002686872
Gambia	0.291368225	0.28750644	0.280202402
Ghana	0.454777418	0.46386195	0.39133479
Guinea	0.121788127	0.119834768	0.115673442
Guinea-Bissau	0.55091962	0.557155919	0.524157632
Liberia	0.834139384	0.823258901	0.829644248

Mali	0.013175461	0.015405685	-0.006195808
Mauritania	0.472673866	0.477092884	0.400607405
Niger	0.471687103	0.484735728	0.422576272
Nigeria	0.6091917	0.625664465	0.53622275
Sao Tome and Principe	0.994641502	0.979253518	0.96125336
Senegal	0.673397792	0.680105954	0.630206043
Sierra Leone	0.73769818	0.731919786	0.727448126
Togo	0.935083255	0.925339514	0.937051749
American Samoa	0.31202798	0.287814687	0.297178166
Bermuda	-0.400033468	-0.445574552	-0.459413631
Cook Islands	-0.136701529	-0.196763983	-0.187878472
Greenland	-0.337158177	-0.353163561	-0.376501566
Guam	-0.188907643	-0.262225823	-0.094223506
Monaco	0.214352239	0.113443942	0.09146569
Nauru	-0.185023108	-0.195506812	-0.186689763
Niue	0.047459349	0.026608524	0.00956157
Northern Mariana Islands	0.890907597	0.843208239	0.826776512
Palau	-0.080709429	-0.092646519	-0.100416078
Puerto Rico	-0.600518953	-0.629591239	-0.608452117
Saint Kitts and Nevis	-0.141377143	-0.156936823	-0.175653927
San Marino	-0.425053275	-0.472200525	-0.473050171
Tokelau	-0.15190754	-0.171975356	-0.171802702
Tuvalu	-0.180855253	-0.190272661	-0.198066258
United States Virgin Islands	-0.302884241	-0.317069016	-0.296456563
South Sudan	-0.135156483	-0.133510173	-0.144628023
Sudan	-0.106821401	-0.105038195	-0.157486769

Supplementary Table S8 | The absolute numbers of esophageal cancer deaths, incidence, and DALYs in 1990 worldwide

Location	***Deaths***			***Incidence***			***DALYs***		
	val	upper	lower	val	upper	lower	val	upper	lower
China	210820.63	244586.52	176081.38	207494.92	241458.64	172673.51	5852132.32	6818927.28	4841614.21
Democratic People's Republic of Korea	1745.44	2317.25	1252.35	1721.56	2303.43	1232.19	50293.31	68122.92	35683.15
Taiwan (Province of China)	1407.23	1491.32	1335.82	1470.52	1556.97	1395.04	39779.80	42192.32	37785.96
Cambodia	200.98	252.92	156.04	195.51	246.76	151.49	5978.84	7536.79	4578.98
Indonesia	1881.72	2288.61	1400.85	1843.65	2236.36	1368.87	56368.14	67913.82	41756.23
Lao People's Democratic Republic	95.63	128.87	68.02	93.01	126.12	64.99	2847.80	3854.33	2025.95
Malaysia	245.67	285.31	205.11	237.27	275.11	197.79	6594.92	7661.44	5523.87
Maldives	2.86	3.68	2.03	2.83	3.65	2.00	87.65	113.58	60.80
Myanmar	961.23	1234.49	722.62	936.08	1201.35	698.42	28670.69	37268.28	20982.62
Philippines	422.68	492.13	372.00	416.15	484.59	366.60	13013.14	15085.51	11510.08
Sri Lanka	618.30	743.52	521.46	602.37	726.61	507.93	17175.75	20695.32	14415.40
Thailand	1776.89	2384.70	1384.98	1759.87	2358.59	1358.92	51500.00	69513.66	40451.49
Timor-Leste	7.47	9.94	5.20	7.34	9.79	5.10	234.16	318.37	159.45
Viet Nam	854.78	1119.69	641.50	823.05	1081.92	611.94	22971.55	30619.03	16926.96
Fiji	7.89	9.92	6.25	7.73	9.71	6.15	235.07	298.58	185.89
Kiribati	2.30	2.85	1.81	2.23	2.77	1.74	68.87	86.24	53.87
Marshall Islands	0.45	0.60	0.31	0.44	0.58	0.30	13.14	17.47	8.89
Micronesia (Federated States of)	1.56	1.98	1.23	1.50	1.91	1.18	44.37	57.35	34.22
Papua New Guinea	34.43	50.28	23.86	33.80	49.85	23.27	1036.83	1537.95	709.34
Samoa	1.09	1.34	0.85	1.05	1.30	0.82	29.18	36.64	22.69
Solomon Islands	3.75	5.08	2.48	3.67	4.95	2.41	113.77	157.02	71.96
Tonga	1.12	1.40	0.90	1.07	1.33	0.85	29.45	36.57	23.66
Vanuatu	1.54	2.09	1.13	1.51	2.04	1.10	46.10	63.59	33.06
Armenia	78.50	84.96	72.00	74.53	80.27	68.52	2064.57	2222.32	1902.34
Azerbaijan	437.03	496.95	378.24	416.89	473.04	361.33	12063.30	13714.53	10484.06

Georgia	140.04	161.25	121.60	132.86	152.42	115.90	3628.27	4155.75	3162.34
Kazakhstan	2490.02	2686.50	2308.55	2355.13	2537.31	2187.85	65421.35	69983.71	60928.72
Kyrgyzstan	259.80	296.73	224.73	247.50	283.80	213.43	7116.56	8171.01	6112.28
Mongolia	253.97	311.91	208.80	236.16	288.61	193.72	6456.53	7954.80	5243.05
Tajikistan	327.47	392.16	269.03	307.71	368.92	252.41	8645.63	10352.77	7050.77
Turkmenistan	584.17	621.28	546.84	556.93	592.62	521.77	15948.27	16971.31	14862.38
Uzbekistan	1708.50	1869.89	1546.05	1623.14	1776.37	1474.75	46168.33	50573.77	41815.84
Albania	40.36	48.34	33.70	38.03	45.54	31.81	1057.86	1270.76	883.98
Bosnia and Herzegovina	101.50	120.09	86.63	98.21	116.12	83.53	2864.23	3396.66	2443.35
Bulgaria	317.71	343.25	295.76	307.09	331.25	286.15	8662.17	9318.29	8065.67
Croatia	235.03	251.77	218.24	234.46	251.02	217.64	6477.41	6971.05	5993.17
Czechia	395.15	433.97	361.88	386.88	423.68	354.39	10312.25	11267.15	9450.24
Hungary	603.91	646.85	560.10	594.28	637.17	550.02	17829.39	19165.54	16519.02
North Macedonia	28.02	32.69	24.32	26.85	31.52	23.33	765.34	904.34	659.09
Montenegro	12.78	15.81	10.50	12.64	15.49	10.39	363.32	444.28	298.95
Poland	1632.87	1685.78	1571.03	1534.61	1584.87	1476.58	42848.84	44177.94	41396.16
Romania	433.10	461.62	408.71	418.81	447.56	395.19	12329.19	13205.02	11593.28
Serbia	278.44	377.16	208.66	268.88	363.53	201.24	7668.89	10486.03	5682.09
Slovakia	245.06	315.67	192.88	243.73	313.89	189.86	7007.20	9182.98	5332.78
Slovenia	85.81	91.37	80.49	85.31	90.92	80.02	2325.97	2489.96	2170.95
Belarus	382.81	414.03	352.09	377.09	407.54	347.24	10667.00	11552.71	9863.48
Estonia	69.52	74.54	64.75	68.32	73.26	63.52	1876.58	2024.22	1722.84
Latvia	113.04	121.48	105.11	109.82	118.00	101.86	3068.55	3305.25	2826.80
Lithuania	146.48	156.94	137.11	145.02	155.49	135.63	3995.86	4266.81	3734.63
Republic of Moldova	112.95	119.27	106.25	110.37	116.34	103.65	3144.27	3327.70	2944.31
Russian Federation	9346.64	9516.03	9150.25	9161.77	9327.00	8974.07	252619.83	257196.41	247639.26
Ukraine	2298.27	2463.85	2156.49	2422.73	2596.80	2268.18	65566.93	70556.04	61174.23
Brunei Darussalam	2.99	3.64	2.39	3.03	3.70	2.42	79.22	97.40	63.30
Japan	8493.33	8716.17	8105.25	11335.28	11675.71	10815.97	204566.68	208796.50	197740.15

Republic of Korea	1716.26	2075.16	1389.77	1839.89	2230.41	1490.69	48742.16	59170.55	39278.99
Singapore	91.08	96.71	85.71	102.23	108.77	95.97	2339.70	2493.65	2205.03
Australia	830.83	886.42	781.23	853.75	909.02	803.83	18924.47	20075.07	17861.20
New Zealand	190.12	201.14	178.04	201.06	213.02	187.88	4123.82	4344.74	3879.08
Andorra	1.10	1.58	0.76	1.15	1.66	0.80	28.65	41.49	19.53
Austria	294.43	311.59	278.09	301.05	318.11	283.54	7182.86	7602.04	6752.92
Belgium	613.59	651.92	572.96	610.57	649.20	569.85	14396.29	15319.90	13514.67
Cyprus	10.86	13.37	8.84	10.38	12.75	8.45	242.53	297.97	195.85
Denmark	383.46	406.07	363.03	374.45	395.65	354.89	8967.62	9469.34	8484.95
Finland	238.13	251.39	223.39	260.87	274.41	244.86	5457.34	5711.15	5198.03
France	6279.68	6663.77	5890.82	6523.12	6913.23	6118.55	159850.71	169863.88	150363.84
Germany	4090.08	4313.95	3869.38	4308.80	4553.93	4075.95	107035.05	113370.11	100726.23
Greece	347.60	367.29	325.32	345.14	364.25	323.62	7438.41	7844.13	7023.27
Iceland	12.95	13.80	11.98	13.29	14.20	12.29	294.54	315.35	273.66
Ireland	322.67	345.40	302.04	316.32	338.12	297.41	7141.60	7609.93	6727.62
Israel	100.19	107.52	92.77	95.59	102.51	88.89	2179.33	2327.63	2044.07
Italy	2920.05	3019.37	2765.44	2910.45	3011.79	2763.16	69321.13	71519.27	66589.17
Luxembourg	26.31	27.71	24.85	26.09	27.50	24.63	642.44	679.35	605.23
Malta	12.41	13.42	11.46	12.27	13.24	11.34	295.06	320.43	272.56
Netherlands	959.19	1013.32	896.38	943.20	997.74	883.77	21875.85	23064.81	20653.25
Norway	173.62	180.52	164.82	174.28	181.00	165.77	3767.38	3896.93	3626.56
Portugal	662.93	701.91	621.94	632.24	669.36	592.27	15946.02	16935.08	15023.10
Spain	2199.17	2321.85	2068.75	2246.88	2374.21	2115.45	55592.27	58824.02	52507.72
Sweden	415.08	438.52	385.33	416.46	438.67	389.28	8487.76	8904.13	8009.87
Switzerland	449.99	475.41	422.26	459.80	485.71	433.02	10511.30	11095.56	9901.43
United Kingdom	6748.03	6906.75	6456.21	6609.19	6755.53	6337.31	143873.60	146437.12	139692.82
Argentina	2378.49	2516.96	2256.13	2230.53	2356.93	2112.29	56905.32	60083.91	53872.79
Chile	769.96	810.60	726.29	716.87	753.09	677.90	17478.36	18351.10	16576.98
Uruguay	351.64	372.29	329.75	328.31	347.84	308.51	7944.83	8441.23	7461.50

Canada	1146.96	1215.97	1075.72	1227.62	1302.40	1153.90	26659.88	28170.05	25185.80
United States of America	11773.69	12076.60	11160.60	12883.46	13191.47	12245.16	285637.33	291098.13	275507.90
Antigua and Barbuda	1.89	2.02	1.76	1.75	1.87	1.62	42.59	45.49	39.66
Bahamas	10.25	11.17	9.42	10.02	10.92	9.20	295.03	323.78	269.48
Barbados	17.79	18.96	16.63	16.37	17.43	15.34	393.38	419.40	370.52
Belize	1.90	2.03	1.77	1.79	1.91	1.67	48.27	51.73	45.14
Cuba	398.68	422.10	377.68	376.83	398.94	357.29	9413.83	9945.03	8929.21
Dominica	3.10	3.66	2.67	2.85	3.34	2.46	71.04	84.05	60.66
Dominican Republic	71.84	86.74	58.49	68.14	82.80	55.44	1952.05	2373.64	1584.53
Grenada	4.78	5.32	4.30	4.42	4.91	3.96	115.19	128.18	103.10
Guyana	8.28	9.23	7.38	7.93	8.88	7.09	230.63	260.03	205.09
Haiti	175.32	228.13	125.48	168.67	221.15	120.30	5031.99	6622.50	3588.57
Jamaica	56.21	60.48	52.36	51.90	55.87	48.34	1264.82	1360.23	1177.89
Saint Lucia	4.61	4.91	4.36	4.32	4.60	4.07	115.79	123.53	108.79
Saint Vincent and the Grenadines	1.73	1.87	1.59	1.62	1.75	1.48	42.97	46.60	39.33
Suriname	4.67	5.39	4.03	4.45	5.13	3.82	126.64	146.64	107.33
Trinidad and Tobago	20.27	21.46	19.14	19.20	20.31	18.13	522.29	552.51	493.18
Bolivia (Plurinational State of)	90.81	116.07	69.69	84.86	107.91	64.61	2337.82	3018.51	1779.87
Ecuador	112.32	118.92	105.00	103.16	109.13	96.64	2710.64	2855.85	2549.63
Peru	215.35	261.03	177.76	197.50	239.20	162.61	5189.26	6331.52	4261.86
Colombia	728.21	765.82	687.75	683.81	716.16	647.30	18455.53	19345.79	17617.29
Costa Rica	44.74	47.88	41.34	42.15	45.10	38.98	1058.70	1132.50	979.36
El Salvador	57.96	64.80	51.50	53.71	59.81	47.88	1457.60	1620.76	1306.36
Guatemala	84.56	88.00	81.09	79.43	82.69	76.11	2283.17	2375.44	2193.29
Honduras	21.24	25.22	17.52	19.97	23.77	16.39	565.33	675.00	464.38
Mexico	950.72	969.42	923.05	880.99	897.21	855.65	23541.85	23946.59	23054.17
Nicaragua	16.07	18.07	13.88	15.03	16.90	12.91	412.95	467.07	353.46
Panama	28.70	30.73	26.66	26.52	28.35	24.67	670.55	712.27	629.98
Venezuela (Bolivarian Republic of)	247.69	259.77	233.61	231.60	242.85	218.99	6197.29	6501.36	5889.26

Brazil	6224.83	6409.72	5970.09	6021.33	6196.17	5776.08	175844.88	180734.59	170377.58
Paraguay	72.44	87.48	60.16	68.47	82.34	56.90	1878.75	2274.62	1570.81
Algeria	86.34	104.13	70.34	82.06	98.52	67.17	2243.97	2700.77	1832.35
Bahrain	5.90	6.88	4.99	5.75	6.69	4.84	163.01	189.89	137.17
Egypt	317.46	374.43	277.55	309.36	360.76	270.46	9419.33	10874.43	8248.71
Iran (Islamic Republic of)	1262.63	1413.53	1084.94	1221.82	1367.08	1049.22	34925.42	39099.12	29950.52
Iraq	97.03	121.92	76.09	93.68	118.19	72.35	2693.76	3415.44	2087.51
Jordan	14.79	18.37	11.62	14.50	18.06	11.44	421.37	527.23	327.73
Kuwait	10.32	11.20	9.48	10.67	11.54	9.80	302.97	327.51	279.06
Lebanon	28.45	36.28	22.30	27.46	35.09	21.44	748.17	957.18	578.04
Libya	27.00	36.29	19.71	26.03	35.20	18.95	734.50	1001.82	528.98
Morocco	120.19	147.41	93.76	114.19	140.49	88.92	3173.46	3919.81	2481.97
Palestine	11.95	15.46	8.82	11.18	14.54	8.20	291.99	386.25	211.90
Oman	12.94	17.25	9.30	12.66	16.88	9.08	361.97	487.71	258.30
Qatar	4.46	5.57	3.51	4.47	5.61	3.49	135.39	171.35	105.54
Saudi Arabia	124.94	167.07	90.01	120.15	161.43	84.95	3420.66	4663.44	2430.91
Syrian Arab Republic	44.97	56.17	35.06	42.99	53.87	33.53	1235.33	1559.38	953.75
Tunisia	33.63	41.44	26.88	32.21	39.62	25.71	856.17	1058.92	679.33
Turkey	855.14	1026.83	695.81	823.26	989.28	669.79	24054.09	29224.63	19012.78
United Arab Emirates	10.09	13.73	7.49	10.09	13.84	7.40	323.03	441.44	234.82
Yemen	251.34	358.14	144.86	243.44	347.12	140.13	7210.93	10344.26	4173.85
Afghanistan	684.74	986.62	333.49	659.05	949.86	317.76	19373.44	28546.45	8993.86
Bangladesh	2549.00	3398.44	1820.74	2461.90	3274.14	1757.18	74614.36	100295.70	53817.08
Bhutan	12.76	17.36	9.16	12.46	17.07	8.87	387.20	527.80	276.54
India	17155.50	21464.34	14894.89	16820.82	20952.46	14654.08	525762.49	653743.51	458716.70
Nepal	474.71	617.29	360.71	461.93	605.34	349.01	14211.58	18714.43	10736.46
Pakistan	3635.37	4180.59	3051.56	3464.73	3984.79	2919.17	99797.04	115914.78	83926.17
Angola	477.04	645.60	320.51	466.74	636.93	312.02	14593.35	19971.11	9760.48
Central African Republic	167.82	207.28	117.08	164.59	203.99	114.58	5168.62	6451.58	3553.17

Congo	174.33	229.07	131.08	169.58	224.00	126.31	5151.94	6813.52	3778.21
Democratic Republic of the Congo	1551.61	2079.24	1080.98	1505.11	1998.57	1044.91	45473.52	61627.79	31044.14
Equatorial Guinea	26.99	35.54	18.74	26.29	34.74	18.22	804.82	1062.25	552.76
Gabon	73.67	92.46	55.13	70.18	88.29	52.61	1999.17	2542.37	1485.68
Burundi	463.14	581.36	334.14	445.42	558.48	321.34	13276.92	16823.81	9416.96
Comoros	34.28	45.58	24.28	33.19	43.84	23.48	995.81	1330.66	696.24
Djibouti	20.75	28.81	14.67	20.43	28.59	14.31	641.27	909.42	440.97
Eritrea	247.70	327.83	161.70	246.81	330.07	160.09	8111.01	10824.65	5216.44
Ethiopia	1959.08	2517.58	1375.40	1903.48	2449.57	1332.97	58050.17	74818.46	40275.78
Kenya	765.21	1102.20	552.27	736.53	1059.44	530.15	21445.99	30697.00	15566.27
Madagascar	768.90	933.78	565.62	742.38	906.05	546.74	22207.25	27217.65	16247.06
Malawi	837.84	1010.05	689.78	811.94	982.00	660.48	24766.88	29765.01	20298.30
Mauritius	26.55	27.91	25.19	26.21	27.61	24.87	741.33	780.02	703.30
Mozambique	448.07	550.51	356.64	422.73	524.65	334.72	11761.13	14541.08	9328.61
Rwanda	628.37	793.79	428.57	611.04	773.75	418.24	18736.08	23689.68	12719.64
Seychelles	3.13	3.68	2.65	3.02	3.57	2.54	85.15	100.97	70.91
Somalia	515.60	709.38	354.82	509.88	698.52	346.93	16532.13	23158.53	11199.91
United Republic of Tanzania	1815.31	2262.65	1351.98	1748.16	2185.27	1309.50	51398.37	64755.65	38241.15
Uganda	965.80	1189.61	764.58	924.29	1142.27	733.12	26809.90	33339.12	21023.81
Zambia	575.68	711.53	441.99	557.52	687.85	431.21	16956.93	20880.39	12808.37
Botswana	69.29	92.74	49.79	67.33	89.77	48.19	2011.39	2755.22	1435.58
Lesotho	90.58	116.01	70.44	86.22	109.51	66.75	2446.77	3151.41	1874.08
Namibia	15.96	19.41	13.18	15.51	18.98	12.72	462.15	567.71	371.22
South Africa	2439.09	2917.86	2149.81	2379.39	2812.48	2101.28	73765.93	86961.66	65650.77
Eswatini	49.28	62.59	36.65	48.07	61.40	35.75	1464.41	1886.79	1083.52
Zimbabwe	524.72	629.85	420.81	503.27	606.77	404.44	14375.86	17331.85	11465.20
Benin	65.20	78.77	54.47	61.58	74.65	50.44	1729.77	2105.62	1412.77
Burkina Faso	145.26	180.80	110.74	138.41	172.83	104.71	3946.40	4954.58	2982.19
Cameroon	162.70	205.23	124.23	156.18	197.36	118.54	4570.44	5809.65	3429.31

Cabo Verde	21.74	25.70	17.70	19.91	23.73	16.38	502.57	592.91	422.73
Chad	75.06	90.92	60.79	70.75	85.67	57.02	1968.95	2398.09	1587.05
Côte d'Ivoire	52.51	63.68	41.80	51.15	62.00	40.69	1559.74	1913.91	1224.14
Gambia	5.39	6.81	4.12	5.18	6.54	3.94	151.41	194.47	114.17
Ghana	155.42	198.02	119.61	149.83	189.59	114.70	4454.37	5775.69	3369.02
Guinea	41.87	51.59	32.93	39.60	48.62	30.87	1110.38	1373.67	871.48
Guinea-Bissau	19.64	24.73	14.62	18.99	23.85	14.10	571.68	726.03	419.12
Liberia	37.16	45.91	29.31	35.11	43.73	27.48	980.99	1220.67	761.27
Mali	99.83	117.53	83.94	96.09	113.52	80.42	2821.63	3356.08	2354.96
Mauritania	37.01	47.42	28.61	35.07	44.86	26.96	982.69	1255.65	740.95
Niger	79.41	100.17	59.47	76.53	96.39	57.20	2284.55	2911.14	1692.89
Nigeria	1222.04	1643.78	937.63	1159.65	1563.25	888.39	33104.36	45424.15	25128.94
Sao Tome and Principe	1.58	1.96	1.27	1.49	1.85	1.18	40.80	51.45	31.84
Senegal	101.85	122.54	82.41	97.04	117.58	77.90	2780.46	3397.67	2214.43
Sierra Leone	59.58	73.86	46.43	56.15	69.89	43.83	1564.38	1976.16	1188.15
Togo	38.12	47.35	30.26	36.71	45.73	29.02	1087.73	1358.35	855.89
American Samoa	0.26	0.32	0.22	0.26	0.31	0.21	7.56	9.22	6.26
Bermuda	4.49	4.85	4.13	4.33	4.69	3.98	111.86	121.30	102.88
Cook Islands	0.36	0.44	0.30	0.35	0.42	0.28	9.43	11.52	7.58
Greenland	5.67	6.55	4.85	5.66	6.54	4.82	169.60	197.84	143.99
Guam	1.65	1.91	1.42	1.65	1.91	1.42	46.75	53.75	40.29
Monaco	3.47	4.36	2.62	3.47	4.38	2.62	71.19	88.79	54.21
Nauru	0.19	0.25	0.14	0.18	0.24	0.13	5.75	7.72	4.17
Niue	0.06	0.07	0.04	0.05	0.06	0.04	1.28	1.58	1.01
Northern Mariana Islands	0.22	0.29	0.17	0.23	0.30	0.18	7.27	9.75	5.59
Palau	0.29	0.37	0.23	0.28	0.36	0.22	7.94	10.27	6.25
Puerto Rico	230.69	245.39	216.30	218.15	232.00	204.87	5462.20	5820.10	5153.46
Saint Kitts and Nevis	1.76	1.86	1.64	1.62	1.72	1.51	40.93	43.52	38.28
San Marino	0.56	0.70	0.44	0.58	0.71	0.45	12.78	16.01	9.97

Tokelau	0.03	0.04	0.02	0.03	0.04	0.02	0.75	1.00	0.50
Tuvalu	0.18	0.22	0.14	0.17	0.21	0.13	5.00	6.19	3.87
United States Virgin Islands	2.88	3.49	2.34	2.81	3.39	2.27	79.04	96.25	63.12
South Sudan	469.36	639.60	347.39	449.15	610.84	331.06	12962.99	17892.41	9507.79
Sudan	459.46	617.26	288.00	439.23	584.95	273.70	12728.49	17328.06	7721.13

Supplementary Table S9 | The absolute numbers of esophageal cancer deaths, incidence, and DALYs in 2021 worldwide

Location	***Deaths***			***Incidence***			***DALYs***		
	val	upper	lower	val	upper	lower	val	upper	lower
China	296443.04	362831.35	236647.81	320805.43	394756.17	256102.37	6898666.18	8553365.98	5471181.02
Democratic People's Republic of Korea	3046.19	4013.57	2259.72	3101.60	4093.05	2303.65	83332.08	111995.91	60440.99
Taiwan (Province of China)	3092.54	3356.42	2777.99	3798.95	4120.22	3434.33	87762.32	94875.02	78833.88
Cambodia	375.16	493.91	275.90	368.11	485.93	267.75	10455.08	13840.17	7574.49
Indonesia	3889.04	4884.69	2981.00	3846.73	4854.81	2948.58	108596.77	136774.42	83024.20
Lao People's Democratic Republic	118.15	165.37	87.13	115.40	160.58	85.38	3386.70	4752.21	2451.13
Malaysia	742.88	864.99	632.21	749.03	873.28	636.39	19234.32	22633.46	16300.90
Maldives	3.90	4.83	3.06	4.04	5.04	3.11	105.52	134.24	80.59
Myanmar	1133.80	1546.74	869.06	1103.75	1499.90	847.42	30961.37	42549.73	23496.57
Philippines	1140.50	1364.36	931.38	1130.96	1345.81	923.42	33042.87	39534.25	26837.32
Sri Lanka	1121.91	1528.61	733.19	1163.80	1581.90	763.65	28218.89	39075.13	18168.27
Thailand	4848.53	6300.69	3619.31	5144.47	6747.48	3850.43	134350.33	177193.04	99350.61
Timor-Leste	18.58	25.68	13.56	17.85	24.32	12.88	490.85	691.43	349.83
Viet Nam	2344.36	2997.04	1757.11	2425.57	3097.43	1797.50	66139.74	86249.46	47736.42
Fiji	19.35	25.21	13.72	18.85	24.82	13.50	532.04	703.56	375.64
Kiribati	4.02	5.26	2.89	3.95	5.17	2.82	123.05	163.28	86.51
Marshall Islands	0.87	1.18	0.64	0.86	1.19	0.62	26.84	36.46	19.08
Micronesia (Federated States of)	2.00	2.64	1.50	1.98	2.64	1.48	59.67	80.31	43.91
Papua New Guinea	78.27	112.16	54.61	76.74	109.00	53.30	2321.54	3322.04	1613.07
Samoa	1.63	2.07	1.27	1.60	2.05	1.25	44.07	56.32	33.57
Solomon Islands	8.44	11.80	5.69	8.35	11.75	5.58	261.27	371.28	174.05
Tonga	1.65	2.14	1.27	1.57	2.04	1.20	40.46	53.26	30.48
Vanuatu	3.85	4.98	3.00	3.77	4.88	2.92	114.10	150.28	87.16
Armenia	48.05	55.20	42.14	44.91	51.40	39.42	1109.34	1274.09	972.18
Azerbaijan	559.28	718.14	411.62	534.50	686.86	394.68	14738.75	18781.58	10874.83

Georgia	67.63	79.72	57.23	62.81	74.00	52.60	1576.35	1849.43	1338.84
Kazakhstan	868.04	1004.88	747.05	831.61	960.93	712.08	22414.58	25881.50	19347.37
Kyrgyzstan	138.03	168.68	108.96	132.35	164.38	103.75	3693.56	4569.70	2886.23
Mongolia	359.35	434.71	286.62	338.61	410.85	269.79	9354.08	11600.70	7332.62
Tajikistan	345.95	445.25	257.19	327.98	428.03	241.84	9340.97	12297.27	6786.29
Turkmenistan	349.96	451.05	268.11	336.56	432.78	255.27	9667.20	12530.02	7336.51
Uzbekistan	998.97	1200.85	826.44	964.13	1152.30	795.11	28051.95	33988.69	22930.55
Albania	65.48	85.93	47.92	60.96	80.12	44.50	1492.30	1971.03	1088.32
Bosnia and Herzegovina	137.74	175.24	102.70	131.15	167.32	98.10	3362.01	4283.24	2481.82
Bulgaria	306.73	351.88	263.27	295.11	341.41	252.99	7724.63	8877.38	6591.17
Croatia	221.66	250.47	192.72	226.23	255.59	195.96	5248.63	5938.81	4582.17
Czechia	631.97	727.15	542.03	663.40	760.86	565.58	14920.43	17271.52	12685.12
Hungary	582.73	659.72	514.58	569.65	645.21	502.77	14800.85	16774.22	13070.83
North Macedonia	46.83	59.06	36.10	44.99	57.56	34.48	1184.97	1521.29	893.21
Montenegro	22.13	28.34	17.14	21.57	27.74	16.55	557.71	715.82	427.51
Poland	2201.00	2398.60	1994.92	2061.77	2248.62	1863.63	53221.09	58062.58	48089.91
Romania	878.69	991.06	774.86	856.04	970.43	750.83	23274.36	26424.17	20437.11
Serbia	349.61	477.50	255.19	340.29	464.93	245.95	8522.05	11826.55	6121.88
Slovakia	298.98	393.61	231.12	299.68	398.03	229.52	7863.60	10776.87	5901.37
Slovenia	96.42	109.17	82.87	103.89	117.44	89.11	2179.97	2485.60	1849.05
Belarus	461.15	573.10	373.16	487.37	601.99	398.47	12754.16	15907.62	10231.73
Estonia	66.72	76.71	57.32	69.58	80.19	59.47	1554.44	1813.72	1325.26
Latvia	107.65	123.10	92.83	106.02	120.75	90.89	2669.46	3077.98	2277.02
Lithuania	191.42	219.39	163.96	194.57	222.77	167.70	4859.59	5576.84	4179.28
Republic of Moldova	111.09	124.37	99.25	112.33	126.30	100.44	2970.43	3329.06	2651.91
Russian Federation	7709.34	8377.31	7022.19	7919.24	8607.86	7176.80	201900.78	219875.16	182635.80
Ukraine	1657.73	2299.26	1139.26	1821.08	2535.77	1257.95	47424.47	66342.38	31907.79
Brunei Darussalam	6.98	8.49	5.54	7.76	9.48	6.12	191.91	235.41	152.19
Japan	14565.16	15419.32	12956.99	21932.89	23180.45	19704.52	267046.36	279927.74	244223.37

Republic of Korea	2195.94	2698.35	1757.30	3382.70	4139.33	2698.84	48069.63	59204.59	38607.55
Singapore	146.12	157.81	131.19	223.73	243.71	201.84	3305.38	3574.10	2995.74
Australia	1757.22	1891.50	1574.10	1853.12	1995.60	1654.72	35246.54	37878.42	32133.15
New Zealand	292.65	316.72	265.18	348.97	377.64	317.67	5770.42	6180.82	5348.12
Andorra	1.84	2.64	1.20	2.08	3.01	1.34	43.61	63.54	27.84
Austria	416.88	449.86	376.30	467.44	503.96	423.56	9094.85	9795.42	8313.55
Belgium	961.41	1046.89	871.28	1030.72	1116.40	940.32	20428.02	22068.06	18838.58
Cyprus	27.69	35.33	21.70	30.03	37.94	23.37	598.35	765.15	465.45
Denmark	565.79	610.69	508.17	595.20	640.12	537.51	11680.10	12530.26	10641.30
Finland	361.02	388.58	326.80	440.79	474.41	401.18	7367.68	7881.07	6792.81
France	5108.43	5570.62	4567.82	6594.04	7184.39	5937.47	107528.81	117303.84	97129.17
Germany	6948.59	7476.90	6330.79	8290.29	8901.19	7570.40	152265.15	162754.48	140344.67
Greece	399.38	426.40	361.97	409.37	435.36	373.14	8175.68	8674.41	7590.56
Iceland	22.69	25.23	19.96	25.82	28.81	22.94	485.01	539.31	434.83
Ireland	434.74	481.48	381.88	485.92	536.36	429.75	9183.94	10069.02	8233.83
Israel	185.24	200.67	165.06	190.34	205.86	171.08	3806.05	4093.71	3476.17
Italy	2395.33	2539.89	2149.03	2510.25	2658.02	2268.34	47549.58	50005.10	44307.81
Luxembourg	36.23	39.96	32.65	39.49	43.65	35.58	800.00	884.64	724.53
Malta	20.88	23.24	18.35	22.29	24.82	19.61	441.47	489.10	393.64
Netherlands	2262.73	2450.10	2042.18	2440.35	2635.22	2219.06	46289.55	49717.61	42366.81
Norway	301.45	318.42	277.02	334.57	353.50	307.62	6093.77	6391.33	5701.23
Portugal	744.83	813.38	677.09	756.15	820.39	689.00	17156.79	18607.44	15727.65
Spain	2292.10	2476.38	2054.65	2563.41	2769.59	2312.09	51240.70	55433.46	46889.35
Sweden	596.51	665.64	515.77	624.52	697.46	541.43	11286.06	12626.23	9814.13
Switzerland	531.79	577.94	467.24	574.70	623.61	511.38	10709.27	11580.88	9631.38
United Kingdom	9745.53	10170.22	8990.11	9949.15	10365.29	9232.38	189121.10	195972.03	178951.45
Argentina	2468.52	2672.66	2277.81	2327.49	2526.06	2151.95	53732.92	58118.79	49964.13
Chile	833.11	899.88	750.31	799.76	862.39	723.98	16434.60	17666.71	15098.90
Uruguay	325.25	352.04	298.30	304.72	329.35	279.77	6618.19	7188.04	6079.51

Canada	2611.02	2808.69	2380.99	2994.30	3214.30	2742.77	55597.12	59559.04	51416.83
United States of America	21340.66	22215.89	19926.58	24328.54	25278.69	22769.16	479504.47	495409.91	457854.35
Antigua and Barbuda	3.12	3.34	2.92	3.01	3.23	2.81	75.56	81.34	70.27
Bahamas	21.97	27.52	17.43	21.58	27.15	17.02	618.86	781.77	487.34
Barbados	27.41	33.46	21.60	25.87	31.86	20.33	621.75	768.90	479.39
Belize	6.64	7.57	5.75	6.46	7.40	5.60	183.86	209.94	157.58
Cuba	979.57	1134.88	830.96	973.43	1121.86	829.70	24758.46	28811.66	20903.82
Dominica	4.39	5.58	3.55	4.16	5.36	3.33	110.20	144.51	87.12
Dominican Republic	229.32	294.97	173.22	218.27	281.71	164.53	6007.98	7753.03	4529.46
Grenada	5.66	6.59	4.80	5.49	6.35	4.63	150.22	176.51	126.38
Guyana	13.97	17.93	10.58	13.59	17.58	10.35	403.38	522.70	302.99
Haiti	294.13	414.91	205.80	283.74	399.59	196.04	8474.83	12126.35	5893.76
Jamaica	97.14	124.43	73.50	91.77	119.15	69.57	2321.06	3036.21	1714.62
Saint Lucia	10.57	12.69	8.64	10.07	12.10	8.20	267.07	323.26	214.85
Saint Vincent and the Grenadines	3.72	4.22	3.26	3.55	4.05	3.11	96.04	110.87	84.31
Suriname	10.85	13.96	8.10	10.44	13.65	7.77	294.54	381.25	218.53
Trinidad and Tobago	38.92	49.44	29.91	37.48	47.62	28.59	1002.81	1291.19	761.19
Bolivia (Plurinational State of)	206.99	278.10	152.03	191.43	257.34	140.37	4871.75	6693.94	3510.10
Ecuador	209.24	260.95	166.10	190.75	239.39	150.05	4496.25	5632.29	3509.50
Peru	450.06	591.33	338.17	419.34	556.97	312.22	9799.83	13139.27	7156.84
Colombia	1040.54	1238.59	861.26	973.24	1156.93	809.83	22601.34	27249.95	18700.80
Costa Rica	89.80	100.77	78.23	85.95	96.28	74.93	2032.89	2291.62	1769.38
El Salvador	112.17	140.69	89.28	104.85	132.39	83.55	2600.07	3293.68	2072.19
Guatemala	190.83	223.40	163.15	176.93	209.97	151.33	4582.91	5387.48	3881.08
Honduras	93.75	118.91	73.63	87.38	111.23	67.91	2268.03	2937.98	1766.78
Mexico	1888.10	2150.23	1639.74	1796.48	2051.36	1552.83	46851.53	53725.80	40310.86
Nicaragua	41.48	51.25	33.53	39.44	48.69	31.72	1030.76	1283.01	833.44
Panama	57.04	68.85	44.50	53.33	64.49	41.55	1254.53	1519.34	969.50
Venezuela (Bolivarian Republic of)	515.07	684.79	375.74	489.57	653.42	354.41	12625.34	16988.74	8928.06

Brazil	12869.95	13441.01	12150.28	12532.77	13069.12	11854.86	342230.08	356454.79	325426.76
Paraguay	243.02	325.08	180.45	234.17	314.87	173.73	6314.15	8426.40	4626.10
Algeria	241.11	302.99	185.36	234.13	294.78	179.94	5982.93	7551.51	4532.69
Bahrain	15.14	19.41	11.90	15.71	20.10	12.31	418.30	548.67	325.45
Egypt	619.02	783.30	485.69	611.13	774.84	477.50	17043.20	21442.89	13289.97
Iran (Islamic Republic of)	2869.74	3129.26	2594.56	2791.16	3035.00	2528.06	68678.43	74957.47	62630.37
Iraq	273.94	351.98	198.01	274.30	353.58	196.82	7509.78	9849.36	5367.94
Jordan	60.19	77.38	46.35	61.35	78.61	47.30	1637.14	2111.97	1243.83
Kuwait	24.06	29.69	19.50	25.56	31.27	20.71	633.93	779.93	510.05
Lebanon	57.74	70.21	46.20	55.83	68.95	44.42	1234.26	1517.48	982.28
Libya	90.38	120.96	63.06	90.26	121.54	62.69	2570.99	3512.31	1755.56
Morocco	301.57	380.32	219.11	290.49	367.02	210.24	7681.12	9926.48	5482.63
Palestine	21.26	26.10	17.19	20.81	25.54	16.74	546.02	674.74	438.61
Oman	28.91	37.24	21.26	30.08	38.71	21.92	822.62	1086.20	599.44
Qatar	18.02	24.59	12.97	20.21	27.66	14.56	568.62	795.48	400.74
Saudi Arabia	311.48	418.90	238.22	331.42	445.89	253.49	9773.16	13484.03	7331.44
Syrian Arab Republic	111.53	144.86	80.84	110.32	145.67	79.34	2872.35	3834.14	2026.87
Tunisia	89.37	124.90	61.67	88.18	123.95	60.57	2166.40	3059.34	1459.47
Turkey	1526.11	1932.19	1155.48	1509.53	1918.15	1143.00	37098.07	46777.31	27841.76
United Arab Emirates	59.83	77.68	44.36	62.82	81.33	47.41	1971.84	2561.99	1468.97
Yemen	546.18	783.44	283.16	526.43	756.10	273.47	15081.71	22075.08	7659.00
Afghanistan	733.06	1099.53	346.77	716.75	1081.89	331.27	22621.89	34777.66	10196.87
Bangladesh	4694.63	6524.79	3318.21	4507.90	6246.77	3207.49	124792.87	175595.81	87649.82
Bhutan	22.61	31.45	16.24	21.38	29.59	15.45	576.92	807.53	410.75
India	38002.11	45561.82	33296.32	37006.84	44293.13	32442.81	1056497.11	1250177.01	927426.25
Nepal	910.38	1297.60	648.48	872.63	1234.34	624.26	24539.28	35170.26	17263.28
Pakistan	7912.71	9968.76	6283.42	7672.59	9653.42	6079.73	228390.68	289878.53	182178.13
Angola	958.73	1264.14	688.78	937.05	1234.79	671.39	28517.75	38319.14	20271.30
Central African Republic	252.23	348.21	168.62	248.68	342.79	166.66	7962.67	11168.82	5243.36

Congo	292.90	398.23	206.55	286.83	390.51	201.96	8758.13	12181.19	6049.82
Democratic Republic of the Congo	2990.85	4074.08	2015.83	2909.45	3907.42	1970.65	87852.43	121143.97	58112.92
Equatorial Guinea	45.03	65.49	30.68	44.04	63.24	30.14	1314.32	1938.13	869.95
Gabon	113.58	147.43	82.20	110.28	142.30	79.51	3209.22	4263.03	2280.75
Burundi	533.56	692.67	383.55	515.89	670.22	369.77	15545.95	20454.09	11158.77
Comoros	63.44	85.34	46.62	60.68	82.22	44.69	1728.69	2336.07	1265.15
Djibouti	79.23	115.38	52.43	77.69	113.53	51.27	2363.88	3492.74	1529.78
Eritrea	410.00	594.24	282.83	401.82	579.86	278.93	12510.15	18170.90	8538.71
Ethiopia	2208.32	2946.89	1784.20	2105.16	2803.99	1688.97	60062.97	80044.00	47898.69
Kenya	2736.43	3846.52	1989.02	2642.50	3713.25	1923.38	76416.53	107056.91	55651.23
Madagascar	1256.68	1726.80	862.14	1233.01	1700.88	850.48	38197.85	52767.12	26089.48
Malawi	2017.24	2540.78	1625.49	1965.11	2478.71	1578.83	59610.56	76806.17	47284.02
Mauritius	65.02	68.79	60.16	66.19	70.13	61.38	1737.64	1838.45	1594.27
Mozambique	993.47	1247.91	749.23	949.91	1194.75	715.69	27406.21	35071.42	20218.86
Rwanda	724.22	968.68	526.65	698.16	924.86	507.06	20353.30	27401.63	14498.36
Seychelles	5.63	6.87	4.54	5.70	6.95	4.59	157.24	193.74	126.64
Somalia	940.27	1263.65	657.18	923.03	1242.68	642.10	29090.92	39371.97	20093.29
United Republic of Tanzania	2994.51	3927.97	2201.54	2877.06	3764.24	2113.19	83555.55	110436.35	60796.67
Uganda	2252.80	2971.59	1721.14	2180.52	2867.36	1672.91	64889.67	85796.24	48346.31
Zambia	1159.46	1800.82	770.04	1133.41	1742.53	751.73	34918.92	55564.82	22964.52
Botswana	129.28	167.83	96.24	125.21	163.02	93.72	3659.86	4834.25	2690.40
Lesotho	180.57	233.70	133.84	175.82	225.07	129.87	5307.80	6987.96	3839.22
Namibia	34.37	45.76	26.35	33.76	45.15	25.60	1014.99	1407.65	744.24
South Africa	5008.86	5609.08	4543.59	4856.66	5432.83	4409.77	138651.21	155949.66	125929.52
Eswatini	100.36	138.63	70.24	99.15	136.95	69.45	3104.83	4386.97	2112.13
Zimbabwe	1148.38	1443.91	870.62	1119.57	1401.82	843.97	34110.09	43725.38	25271.66
Benin	267.75	348.75	200.98	256.52	335.59	192.58	7435.66	9901.92	5516.97
Burkina Faso	521.75	672.29	361.63	497.81	639.32	346.98	14271.05	18427.52	9911.97
Cameroon	822.35	1140.37	559.91	797.73	1099.30	537.56	23887.02	33363.42	16061.75

Cabo Verde	69.65	85.79	55.08	67.26	83.15	52.57	1856.43	2296.43	1451.22
Chad	326.84	427.55	242.77	314.04	413.74	229.26	9243.89	12289.40	6676.33
Côte d'Ivoire	149.59	201.60	110.69	145.60	197.70	107.44	4369.97	6061.09	3160.22
Gambia	19.48	24.83	14.78	18.69	23.56	14.33	538.75	696.92	406.58
Ghana	594.54	772.26	404.61	571.68	736.94	386.53	16371.39	21334.37	11320.74
Guinea	78.83	106.67	55.95	75.20	101.40	52.95	2161.51	2953.90	1522.05
Guinea-Bissau	55.25	69.56	41.13	54.04	68.14	40.26	1677.72	2154.40	1234.66
Liberia	123.32	175.81	84.93	119.78	170.49	82.37	3618.30	5287.40	2463.06
Mali	222.86	288.98	168.65	214.37	275.77	163.36	6241.40	8157.94	4724.97
Mauritania	115.90	159.49	82.01	110.19	151.57	77.78	2997.45	4202.57	2119.39
Niger	344.67	458.69	236.11	329.11	440.88	226.93	9468.88	12924.17	6440.51
Nigeria	3946.92	5274.37	2694.96	3764.40	5059.16	2542.65	107905.02	147643.87	72624.31
Sao Tome and Principe	5.27	6.80	4.02	5.10	6.64	3.90	145.85	194.50	108.97
Senegal	407.00	528.64	307.19	387.74	505.88	291.97	10960.42	14607.84	8177.90
Sierra Leone	189.12	254.19	136.36	181.20	241.72	129.99	5246.46	7196.18	3778.23
Togo	232.34	314.80	164.48	226.71	309.32	160.61	6834.70	9350.29	4850.45
American Samoa	0.74	0.90	0.60	0.73	0.88	0.59	19.92	24.49	15.85
Bermuda	5.62	6.70	4.76	5.66	6.77	4.79	121.36	144.90	101.11
Cook Islands	0.65	0.78	0.51	0.65	0.79	0.51	15.14	18.71	11.71
Greenland	7.78	9.59	6.18	7.88	9.68	6.22	210.66	258.34	167.27
Guam	3.93	4.63	3.29	3.98	4.70	3.32	106.90	125.89	89.48
Monaco	5.46	6.66	4.31	5.84	7.15	4.64	107.65	132.72	84.84
Nauru	0.19	0.25	0.14	0.19	0.25	0.14	5.92	7.81	4.19
Niue	0.05	0.07	0.04	0.05	0.06	0.04	1.32	1.66	1.00
Northern Mariana Islands	1.26	1.49	1.04	1.29	1.52	1.06	34.75	40.77	28.64
Palau	0.59	0.75	0.47	0.59	0.74	0.47	16.60	21.25	12.96
Puerto Rico	168.91	198.01	137.97	164.12	193.01	134.65	3635.25	4285.00	2966.89
Saint Kitts and Nevis	2.77	3.26	2.29	2.71	3.20	2.23	74.47	88.66	60.77
San Marino	0.64	0.92	0.41	0.68	1.00	0.44	13.32	19.82	8.26

Tokelau	0.03	0.04	0.02	0.03	0.03	0.02	0.68	0.86	0.50
Tuvalu	0.22	0.28	0.17	0.22	0.27	0.17	6.07	7.69	4.62
United States Virgin Islands	4.29	5.50	3.25	4.06	5.25	3.07	99.51	130.01	75.88
South Sudan	614.05	820.35	442.95	599.44	799.21	429.64	18316.47	24573.24	13027.96
Sudan	838.96	1188.55	526.25	809.69	1154.48	497.96	23043.26	33219.64	13703.86

Supplementary Table S10 | The percentage change of absolute numbers of esophageal cancer deaths, incidence, and DALYs from 1990 to 2021 worldwide (Generated from data available from http://ghdx.healthdata.org/gbd-results-tool)

Location	***Incidence***	***Deaths***	***DALYs***
China	0.546088116	0.406138669	0.178829494
Democratic People's Republic of Korea	0.801621785	0.745227564	0.656921766
Taiwan (Province of China)	1.58340587	1.197608067	1.206203148
Cambodia	0.882819293	0.866653398	0.748680346
Indonesia	1.086475199	1.066747444	0.926562949
Lao People's Democratic Republic	0.240726804	0.235490955	0.189233795
Malaysia	2.156867703	2.023893841	1.916535758
Maldives	0.427561837	0.363636364	0.203879064
Myanmar	0.179119306	0.179530393	0.079896229
Philippines	1.717673916	1.69825873	1.539192693
Sri Lanka	0.932035128	0.814507521	0.642949507
Thailand	1.923210237	1.72866075	1.608744272
Timor-Leste	1.431880109	1.487282463	1.096216262
Viet Nam	1.947050604	1.742647231	1.879202318
Fiji	1.4385511	1.452471483	1.263325818
Kiribati	0.771300448	0.747826087	0.786699579
Marshall Islands	0.954545455	0.933333333	1.04261796
Micronesia (Federated States of)	0.32	0.282051282	0.344827586
Papua New Guinea	1.270414201	1.273308161	1.239074872
Samoa	0.523809524	0.495412844	0.510281014
Solomon Islands	1.27520436	1.250666667	1.296475345
Tonga	0.46728972	0.473214286	0.37385399
Vanuatu	1.496688742	1.5	1.47505423
Armenia	-0.397423856	-0.387898089	-0.462677458
Azerbaijan	0.282112788	0.27972908	0.221784255
Georgia	-0.527246726	-0.517066552	-0.565536743
Kazakhstan	-0.646894227	-0.651392358	-0.657381268
Kyrgyzstan	-0.465252525	-0.468706697	-0.480990816
Mongolia	0.433816057	0.414930897	0.448778214
Tajikistan	0.065873712	0.05643265	0.080426759
Turkmenistan	-0.39568707	-0.400927812	-0.39384021
Uzbekistan	-0.40600934	-0.415294118	-0.392398426
Albania	0.602945043	0.622398414	0.410678162
Bosnia and Herzegovina	0.335403727	0.357044335	0.173791909
Bulgaria	-0.039011365	-0.034559819	-0.108233849
Croatia	-0.035101936	-0.056886355	-0.189702366
Czechia	0.71474359	0.599316715	0.446864651
Hungary	-0.04144511	-0.035071451	-0.169862233

North Macedonia	0.675605214	0.67130621	0.548292262
Montenegro	0.706487342	0.731611894	0.535037983
Poland	0.343513987	0.347933393	0.242066063
Romania	1.043981758	1.028838605	0.88774445
Serbia	0.26558316	0.255602643	0.111249477
Slovakia	0.229557297	0.220027748	0.122217148
Slovenia	0.217793928	0.123645263	-0.062769511
Belarus	0.292450078	0.204644602	0.195665135
Estonia	0.018442623	-0.04027618	-0.171663345
Latvia	-0.034602076	-0.047682236	-0.130058171
Lithuania	0.34167701	0.306799563	0.216156222
Republic of Moldova	0.017758449	-0.016467463	-0.055287873
Russian Federation	-0.135621174	-0.17517525	-0.200772243
Ukraine	-0.248335555	-0.278705287	-0.27670138
Brunei Darussalam	1.561056106	1.334448161	1.42249432
Japan	0.934922649	0.714893923	0.30542452
Republic of Korea	0.838533825	0.279491452	-0.013797706
Singapore	1.188496527	0.604303909	0.412736676
Australia	1.170565154	1.115017513	0.86248492
New Zealand	0.735651049	0.539290974	0.399289979
Andorra	0.808695652	0.672727273	0.522164049
Austria	0.552698887	0.415888327	0.266187842
Belgium	0.688127487	0.566860607	0.418978084
Cyprus	1.893063584	1.549723757	1.46711747
Denmark	0.589531313	0.475486361	0.302474904
Finland	0.689692184	0.516062655	0.350049658
France	0.010872098	-0.186514281	-0.327317282
Germany	0.924036855	0.69888853	0.422572793
Greece	0.186098395	0.148964327	0.099116612
Iceland	0.942814146	0.752123552	0.646669383
Ireland	0.536165908	0.347320792	0.285977932
Israel	0.99121247	0.848887114	0.746431243
Italy	-0.13750451	-0.179695553	-0.314068019
Luxembourg	0.513606746	0.377042949	0.245252475
Malta	0.816625917	0.682514102	0.496204162
Netherlands	1.58730916	1.359000824	1.116011492
Norway	0.919726876	0.736263103	0.61750872
Portugal	0.195985702	0.123542455	0.075929291
Spain	0.140875347	0.042256851	-0.07827653
Sweden	0.499591798	0.437096463	0.329686513
Switzerland	0.249891257	0.181781817	0.018834017
United Kingdom	0.505350883	0.444203716	0.314494807
Argentina	0.043469489	0.037851746	-0.055748742
Chile	0.115627659	0.082017248	-0.059717273

Uruguay	-0.071852822	-0.075048345	-0.166981546
Canada	1.439109822	1.276469973	1.08542274
United States of America	0.888354526	0.812571929	0.678717799
Antigua and Barbuda	0.72	0.650793651	0.774125382
Bahamas	1.153692615	1.143414634	1.097617191
Barbados	0.580329872	0.540753232	0.580532818
Belize	2.608938547	2.494736842	2.808991092
Cuba	1.583207282	1.45703321	1.630009252
Dominica	0.459649123	0.416129032	0.551238739
Dominican Republic	2.203257998	2.192093541	2.07777977
Grenada	0.242081448	0.184100418	0.304106259
Guyana	0.713745271	0.687198068	0.749035251
Haiti	0.682219719	0.677675108	0.684190549
Jamaica	0.768208092	0.728162249	0.835091159
Saint Lucia	1.331018519	1.292841649	1.306503152
Saint Vincent and the Grenadines	1.191358025	1.150289017	1.235047708
Suriname	1.346067416	1.323340471	1.325805433
Trinidad and Tobago	0.952083333	0.920078934	0.920025273
Bolivia (Plurinational State of)	1.255833137	1.279374518	1.083885842
Ecuador	0.849069407	0.862891738	0.658741109
Peru	1.123240506	1.089900163	0.888483136
Colombia	0.423260847	0.428901004	0.224637819
Costa Rica	1.039145907	1.007152436	0.920175687
El Salvador	0.952150438	0.935300207	0.783802141
Guatemala	1.227495908	1.256740776	1.007257453
Honduras	3.375563345	3.413841808	3.011869174
Mexico	1.03916049	0.985968529	0.990137988
Nicaragua	1.624085163	1.581207218	1.496089115
Panama	1.010935143	0.987456446	0.870897025
Venezuela (Bolivarian Republic of)	1.113860104	1.079494529	1.03723563
Brazil	1.081395639	1.06751831	0.946204405
Paraguay	2.420037973	2.354776367	2.360825017
Algeria	1.853156227	1.792564281	1.666225484
Bahrain	1.732173913	1.566101695	1.566100239
Egypt	0.975465477	0.94991495	0.809385593
Iran (Islamic Republic of)	1.284428148	1.272827352	0.966431041
Iraq	1.928052946	1.823250541	1.787843015
Jordan	3.231034483	3.06964165	2.885278971
Kuwait	1.395501406	1.331395349	1.092385385
Lebanon	1.033139111	1.029525483	0.649705281
Libya	2.467537457	2.347407407	2.500326753
Morocco	1.543918031	1.509110575	1.420424395
Palestine	0.861359571	0.779079498	0.869995548
Oman	1.375987362	1.234157651	1.272619278

Qatar	3.521252796	3.040358744	3.199867051
Saudi Arabia	1.758385352	1.493036658	1.857097753
Syrian Arab Republic	1.566178181	1.480097843	1.325168174
Tunisia	1.737659112	1.657448707	1.530338601
Turkey	0.833600564	0.784631756	0.54227701
United Arab Emirates	5.225966303	4.9296333	5.104200848
Yemen	1.16246303	1.173072332	1.091506921
Afghanistan	0.087550262	0.070566931	0.167675436
Bangladesh	0.831065437	0.841753629	0.67250473
Bhutan	0.715890851	0.771943574	0.489979339
India	1.20006159	1.215156072	1.009456989
Nepal	0.88909575	0.917760317	0.72671019
Pakistan	1.214484246	1.176590003	1.288551644
Angola	1.007648798	1.00974761	0.954160628
Central African Republic	0.510905887	0.502979383	0.540579497
Congo	0.691414082	0.680146848	0.699967391
Democratic Republic of the Congo	0.933048083	0.927578451	0.931946988
Equatorial Guinea	0.675161658	0.668395702	0.633060809
Gabon	0.57138786	0.541740193	0.60527619
Burundi	0.158210229	0.152049056	0.170900329
Comoros	0.828261525	0.850641774	0.735963688
Djibouti	2.802741067	2.818313253	2.686247602
Eritrea	0.628053969	0.655228099	0.542366487
Ethiopia	0.105953307	0.127222982	0.034673456
Kenya	2.587769677	2.576051019	2.56320832
Madagascar	0.660887955	0.634386786	0.720062142
Malawi	1.420265044	1.407667335	1.406865944
Mauritius	1.525371995	1.448964218	1.343949388
Mozambique	1.247084427	1.217220524	1.330236125
Rwanda	0.142576591	0.152537518	0.086315814
Seychelles	0.887417219	0.798722045	0.846623605
Somalia	0.810288695	0.823642358	0.759659524
United Republic of Tanzania	0.64576469	0.649586021	0.625645911
Uganda	1.35912971	1.332574032	1.420362254
Zambia	1.032949491	1.014070317	1.059271342
Botswana	0.859646517	0.865781498	0.819567563
Lesotho	1.039202041	0.993486421	1.169308926
Namibia	1.176660219	1.153508772	1.196234989
South Africa	1.041136594	1.053577359	0.879610411
Eswatini	1.062617017	1.036525974	1.12019175
Zimbabwe	1.224591174	1.188557707	1.372733875
Benin	3.165638194	3.106595092	3.29864086
Burkina Faso	2.596633191	2.59183533	2.616219846
Cameroon	4.107760277	4.054394591	4.226415837

Cabo Verde	2.378201909	2.203771849	2.69387349
Chad	3.438727915	3.35438316	3.694832271
Côte d'Ivoire	1.846529814	1.848790707	1.801729775
Gambia	2.608108108	2.614100186	2.558219404
Ghana	2.815524261	2.825376399	2.675354764
Guinea	0.898989899	0.882732267	0.946639889
Guinea-Bissau	1.845708268	1.813136456	1.934718724
Liberia	2.411563657	2.318622174	2.688416803
Mali	1.230929337	1.232395072	1.211983853
Mauritania	2.142001711	2.131586058	2.050249824
Niger	3.30040507	3.340385342	3.14474623
Nigeria	2.246151856	2.229779713	2.259541039
Sao Tome and Principe	2.422818792	2.335443038	2.574754902
Senegal	2.995671888	2.996072656	2.941944858
Sierra Leone	2.227070347	2.174219537	2.353699229
Togo	5.175701444	5.094963274	5.283452695
American Samoa	1.807692308	1.846153846	1.634920635
Bermuda	0.307159353	0.251670379	0.084927588
Cook Islands	0.857142857	0.805555556	0.605514316
Greenland	0.392226148	0.372134039	0.242099057
Guam	1.412121212	1.381818182	1.286631016
Monaco	0.682997118	0.573487032	0.512150583
Nauru	0.055555556	0	0.029565217
Niue	0	-0.166666667	0.03125
Northern Mariana Islands	4.608695652	4.727272727	3.779917469
Palau	1.107142857	1.034482759	1.090680101
Puerto Rico	-0.247673619	-0.26780528	-0.334471458
Saint Kitts and Nevis	0.672839506	0.573863636	0.819447838
San Marino	0.172413793	0.142857143	0.042253521
Tokelau	0	0	-0.093333333
Tuvalu	0.294117647	0.222222222	0.214
United States Virgin Islands	0.444839858	0.489583333	0.258982794
South Sudan	0.334609819	0.308270837	0.412981881
Sudan	0.843430549	0.825969617	0.810368708

Supplementary Table S11 | The ASDR and ASDALYR in world and seven GBD super regions, and regions with five SDI quintiles of males, females, and both sexes attributable to four most detailed risks and their proportion of contribution in 1990 and 2021

(A) In 1990, both sexes

measure_name	location_name	sex_name	rei_name	year	val	upper	lower
**ASDALYR**	Global	Both	Alcohol use	1990	36.21	47.62	26.35
**ASDALYR**	Global	Both	Smoking	1990	88.47	106.51	71.26
**ASDALYR**	Global	Both	Diet low in vegetables	1990	49.01	96.93	-10.74
**ASDALYR**	Latin America and Caribbean	Both	Smoking	1990	42.61	50.63	34.34
**ASDALYR**	Global	Both	Chewing tobacco	1990	6.76	8.86	4.77
**ASDALYR**	Latin America and Caribbean	Both	Chewing tobacco	1990	0.86	1.23	0.54
**ASDALYR**	Central Europe, Eastern Europe, and Central Asia	Both	Alcohol use	1990	26.50	33.67	18.99
**ASDALYR**	Southeast Asia, East Asia, and Oceania	Both	Smoking	1990	199.07	249.02	155.45
**ASDALYR**	Southeast Asia, East Asia, and Oceania	Both	Diet low in vegetables	1990	107.05	211.36	-23.09
**ASDALYR**	Latin America and Caribbean	Both	Alcohol use	1990	16.36	21.15	11.58
**ASDALYR**	Latin America and Caribbean	Both	Diet low in vegetables	1990	27.45	53.23	-6.24
**ASDALYR**	Southeast Asia, East Asia, and Oceania	Both	Alcohol use	1990	74.88	103.65	52.62
**ASDALYR**	Central Europe, Eastern Europe, and Central Asia	Both	Diet low in vegetables	1990	22.49	45.50	-4.85
**ASDALYR**	Central Europe, Eastern Europe, and Central Asia	Both	Smoking	1990	48.91	56.58	40.75
**ASDALYR**	High-income	Both	Diet low in vegetables	1990	20.52	41.16	-4.46
**ASDALYR**	High-income	Both	Alcohol use	1990	28.18	35.69	20.77
**ASDALYR**	Southeast Asia, East Asia, and Oceania	Both	Chewing tobacco	1990	5.38	7.88	3.28
**ASDALYR**	Central Europe, Eastern Europe, and Central Asia	Both	Chewing tobacco	1990	0.87	1.29	0.53
**ASDALYR**	High-income	Both	Smoking	1990	57.37	66.57	46.97
**ASDALYR**	Sub-Saharan Africa	Both	Smoking	1990	30.43	37.17	23.57
**ASDALYR**	Sub-Saharan Africa	Both	Alcohol use	1990	23.47	31.93	12.47
**ASDALYR**	Sub-Saharan Africa	Both	Diet low in vegetables	1990	58.93	112.07	-13.36
**ASDALYR**	High-income	Both	Chewing tobacco	1990	1.80	2.67	1.08
**ASDALYR**	Sub-Saharan Africa	Both	Chewing tobacco	1990	6.56	9.13	4.33

**ASDALYR**	North Africa and Middle East	Both	Smoking	1990	18.14	22.23	14.13
**ASDALYR**	South Asia	Both	Smoking	1990	23.48	29.21	18.31
**ASDALYR**	North Africa and Middle East	Both	Alcohol use	1990	0.98	1.40	0.59
**ASDALYR**	North Africa and Middle East	Both	Chewing tobacco	1990	1.65	2.44	1.04
**ASDALYR**	South Asia	Both	Chewing tobacco	1990	26.76	34.45	19.41
**ASDALYR**	South Asia	Both	Alcohol use	1990	4.32	6.52	1.78
**ASDALYR**	North Africa and Middle East	Both	Diet low in vegetables	1990	11.40	23.59	-2.28
**ASDALYR**	South Asia	Both	Diet low in vegetables	1990	26.48	52.53	-6.35
**ASDALYR**	Low SDI	Both	Chewing tobacco	1990	13.03	17.44	9.02
**ASDALYR**	Middle SDI	Both	Diet low in vegetables	1990	78.94	156.03	-17.05
**ASDALYR**	High-middle SDI	Both	Diet low in vegetables	1990	59.05	117.94	-12.82
**ASDALYR**	High-middle SDI	Both	Smoking	1990	121.09	148.22	96.00
**ASDALYR**	Middle SDI	Both	Smoking	1990	135.81	169.80	107.56
**ASDALYR**	Middle SDI	Both	Alcohol use	1990	48.59	67.50	34.18
**ASDALYR**	High-middle SDI	Both	Alcohol use	1990	55.33	72.05	39.81
**ASDALYR**	High SDI	Both	Diet low in vegetables	1990	23.12	46.44	-4.97
**ASDALYR**	Middle SDI	Both	Chewing tobacco	1990	6.93	9.49	4.74
**ASDALYR**	High-middle SDI	Both	Chewing tobacco	1990	2.50	3.81	1.44
**ASDALYR**	High SDI	Both	Smoking	1990	61.87	72.11	50.84
**ASDALYR**	Low-middle SDI	Both	Diet low in vegetables	1990	26.53	51.78	-6.29
**ASDALYR**	High SDI	Both	Alcohol use	1990	28.84	36.55	21.29
**ASDALYR**	Low-middle SDI	Both	Smoking	1990	26.54	32.26	21.08
**ASDALYR**	Low SDI	Both	Diet low in vegetables	1990	47.54	90.97	-11.14
**ASDALYR**	High SDI	Both	Chewing tobacco	1990	2.04	3.02	1.21
**ASDALYR**	Low SDI	Both	Smoking	1990	21.82	27.06	16.63
**ASDALYR**	Low SDI	Both	Alcohol use	1990	12.05	16.65	5.74
**ASDALYR**	Low-middle SDI	Both	Chewing tobacco	1990	18.48	23.69	13.14
**ASDALYR**	Low-middle SDI	Both	Alcohol use	1990	6.47	8.89	4.08
**ASDR**	Central Europe, Eastern Europe, and Central Asia	Both	Alcohol use	1990	0.87	1.12	0.59

**ASDR**	Latin America and Caribbean	Both	Smoking	1990	1.75	2.10	1.40
**ASDR**	Southeast Asia, East Asia, and Oceania	Both	Smoking	1990	7.96	9.91	6.22
**ASDR**	High-income	Both	Smoking	1990	2.32	2.70	1.88
**ASDR**	Central Europe, Eastern Europe, and Central Asia	Both	Smoking	1990	1.74	2.03	1.45
**ASDR**	Southeast Asia, East Asia, and Oceania	Both	Alcohol use	1990	2.62	3.64	1.83
**ASDR**	Latin America and Caribbean	Both	Chewing tobacco	1990	0.04	0.05	0.02
**ASDR**	Southeast Asia, East Asia, and Oceania	Both	Diet low in vegetables	1990	4.26	8.41	-0.90
**ASDR**	Southeast Asia, East Asia, and Oceania	Both	Chewing tobacco	1990	0.19	0.28	0.12
**ASDR**	High-income	Both	Diet low in vegetables	1990	0.84	1.68	-0.18
**ASDR**	High-income	Both	Alcohol use	1990	1.03	1.33	0.74
**ASDR**	High-income	Both	Chewing tobacco	1990	0.07	0.11	0.04
**ASDR**	Central Europe, Eastern Europe, and Central Asia	Both	Chewing tobacco	1990	0.03	0.04	0.02
**ASDR**	Global	Both	Smoking	1990	3.46	4.13	2.79
**ASDR**	Global	Both	Diet low in vegetables	1990	1.89	3.73	-0.42
**ASDR**	Global	Both	Alcohol use	1990	1.27	1.67	0.92
**ASDR**	Global	Both	Chewing tobacco	1990	0.24	0.32	0.17
**ASDR**	South Asia	Both	Chewing tobacco	1990	1.02	1.31	0.73
**ASDR**	Central Europe, Eastern Europe, and Central Asia	Both	Diet low in vegetables	1990	0.85	1.73	-0.18
**ASDR**	Latin America and Caribbean	Both	Alcohol use	1990	0.57	0.74	0.39
**ASDR**	North Africa and Middle East	Both	Smoking	1990	0.75	0.93	0.58
**ASDR**	Latin America and Caribbean	Both	Diet low in vegetables	1990	1.12	2.16	-0.25
**ASDR**	North Africa and Middle East	Both	Diet low in vegetables	1990	0.47	0.96	-0.09
**ASDR**	South Asia	Both	Diet low in vegetables	1990	1.01	2.00	-0.24
**ASDR**	Sub-Saharan Africa	Both	Diet low in vegetables	1990	2.28	4.32	-0.52
**ASDR**	South Asia	Both	Smoking	1990	0.94	1.18	0.73
**ASDR**	North Africa and Middle East	Both	Chewing tobacco	1990	0.06	0.09	0.04
**ASDR**	Sub-Saharan Africa	Both	Alcohol use	1990	0.84	1.14	0.46
**ASDR**	North Africa and Middle East	Both	Alcohol use	1990	0.03	0.05	0.02
**ASDR**	Sub-Saharan Africa	Both	Smoking	1990	1.20	1.47	0.93

**ASDR**	South Asia	Both	Alcohol use	1990	0.14	0.22	0.06
**ASDR**	Sub-Saharan Africa	Both	Chewing tobacco	1990	0.25	0.35	0.17
**ASDR**	Low-middle SDI	Both	Diet low in vegetables	1990	1.02	1.99	-0.24
**ASDR**	High-middle SDI	Both	Diet low in vegetables	1990	2.26	4.54	-0.49
**ASDR**	Low SDI	Both	Alcohol use	1990	0.43	0.60	0.21
**ASDR**	Middle SDI	Both	Alcohol use	1990	1.69	2.35	1.19
**ASDR**	Low-middle SDI	Both	Smoking	1990	1.06	1.30	0.84
**ASDR**	High-middle SDI	Both	Smoking	1990	4.61	5.64	3.67
**ASDR**	High SDI	Both	Alcohol use	1990	1.05	1.35	0.77
**ASDR**	Low SDI	Both	Diet low in vegetables	1990	1.84	3.50	-0.44
**ASDR**	Low-middle SDI	Both	Chewing tobacco	1990	0.70	0.89	0.49
**ASDR**	High SDI	Both	Diet low in vegetables	1990	0.94	1.88	-0.20
**ASDR**	Middle SDI	Both	Diet low in vegetables	1990	3.11	6.14	-0.66
**ASDR**	High-middle SDI	Both	Chewing tobacco	1990	0.08	0.12	0.05
**ASDR**	Low SDI	Both	Smoking	1990	0.87	1.08	0.66
**ASDR**	Low SDI	Both	Chewing tobacco	1990	0.50	0.66	0.34
**ASDR**	Middle SDI	Both	Smoking	1990	5.36	6.69	4.24
**ASDR**	High-middle SDI	Both	Alcohol use	1990	1.89	2.46	1.35
**ASDR**	High SDI	Both	Smoking	1990	2.49	2.90	2.03
**ASDR**	Low-middle SDI	Both	Alcohol use	1990	0.22	0.30	0.14
**ASDR**	Middle SDI	Both	Chewing tobacco	1990	0.26	0.35	0.18
**ASDR**	High SDI	Both	Chewing tobacco	1990	0.08	0.12	0.05

(B) In 1990, male

measure_name	location_name	sex_name	rei_name	year	val	upper	lower
**ASDALYR**	Global	Male	Alcohol use	1990	70.52	92.80	51.50
**ASDALYR**	Global	Male	Smoking	1990	173.77	210.22	139.43
**ASDALYR**	Global	Male	Diet low in vegetables	1990	73.45	146.74	-16.22
**ASDALYR**	Latin America and Caribbean	Male	Smoking	1990	76.08	89.66	61.84

**ASDALYR**	Global	Male	Chewing tobacco	1990	9.68	13.70	6.03
**ASDALYR**	Latin America and Caribbean	Male	Chewing tobacco	1990	1.38	2.19	0.77
**ASDALYR**	Central Europe, Eastern Europe, and Central Asia	Male	Alcohol use	1990	57.41	73.23	40.72
**ASDALYR**	Southeast Asia, East Asia, and Oceania	Male	Smoking	1990	388.37	489.79	299.69
**ASDALYR**	Southeast Asia, East Asia, and Oceania	Male	Diet low in vegetables	1990	158.58	318.99	-35.10
**ASDALYR**	Latin America and Caribbean	Male	Alcohol use	1990	31.69	40.74	22.47
**ASDALYR**	Latin America and Caribbean	Male	Diet low in vegetables	1990	42.69	82.78	-9.74
**ASDALYR**	Southeast Asia, East Asia, and Oceania	Male	Alcohol use	1990	146.18	203.24	102.33
**ASDALYR**	Central Europe, Eastern Europe, and Central Asia	Male	Diet low in vegetables	1990	40.69	82.17	-8.84
**ASDALYR**	Central Europe, Eastern Europe, and Central Asia	Male	Smoking	1990	115.00	132.84	95.94
**ASDALYR**	High-income	Male	Diet low in vegetables	1990	36.00	72.13	-7.85
**ASDALYR**	High-income	Male	Alcohol use	1990	54.35	68.83	39.46
**ASDALYR**	Southeast Asia, East Asia, and Oceania	Male	Chewing tobacco	1990	8.19	13.08	4.41
**ASDALYR**	Central Europe, Eastern Europe, and Central Asia	Male	Chewing tobacco	1990	1.77	2.67	1.00
**ASDALYR**	High-income	Male	Smoking	1990	111.34	128.30	92.00
**ASDALYR**	Sub-Saharan Africa	Male	Smoking	1990	52.84	65.33	41.12
**ASDALYR**	Sub-Saharan Africa	Male	Alcohol use	1990	38.70	52.20	21.07
**ASDALYR**	Sub-Saharan Africa	Male	Diet low in vegetables	1990	70.84	135.89	-17.39
**ASDALYR**	High-income	Male	Chewing tobacco	1990	3.88	5.88	2.26
**ASDALYR**	Sub-Saharan Africa	Male	Chewing tobacco	1990	7.42	11.34	4.27
**ASDALYR**	North Africa and Middle East	Male	Smoking	1990	32.40	39.84	25.12
**ASDALYR**	South Asia	Male	Smoking	1990	39.73	49.52	31.19
**ASDALYR**	North Africa and Middle East	Male	Alcohol use	1990	1.75	2.52	1.03
**ASDALYR**	North Africa and Middle East	Male	Chewing tobacco	1990	2.33	3.71	1.28
**ASDALYR**	South Asia	Male	Chewing tobacco	1990	31.31	42.59	20.59
**ASDALYR**	South Asia	Male	Alcohol use	1990	7.76	11.74	3.25
**ASDALYR**	North Africa and Middle East	Male	Diet low in vegetables	1990	11.79	24.61	-2.44
**ASDALYR**	South Asia	Male	Diet low in vegetables	1990	28.25	56.94	-6.33
**ASDALYR**	Low SDI	Male	Chewing tobacco	1990	16.12	23.14	10.35

**ASDALYR**	Middle SDI	Male	Diet low in vegetables	1990	112.81	223.33	-25.48
**ASDALYR**	High-middle SDI	Male	Diet low in vegetables	1990	98.76	202.82	-21.31
**ASDALYR**	High-middle SDI	Male	Smoking	1990	254.66	314.26	201.19
**ASDALYR**	Middle SDI	Male	Smoking	1990	261.97	329.65	205.07
**ASDALYR**	Middle SDI	Male	Alcohol use	1990	93.78	129.90	65.87
**ASDALYR**	High-middle SDI	Male	Alcohol use	1990	113.65	148.57	81.79
**ASDALYR**	High SDI	Male	Diet low in vegetables	1990	40.62	81.84	-8.72
**ASDALYR**	Middle SDI	Male	Chewing tobacco	1990	9.97	14.43	6.00
**ASDALYR**	High-middle SDI	Male	Chewing tobacco	1990	4.51	7.26	2.26
**ASDALYR**	High SDI	Male	Smoking	1990	120.77	139.62	100.09
**ASDALYR**	Low-middle SDI	Male	Diet low in vegetables	1990	30.07	60.81	-6.84
**ASDALYR**	High SDI	Male	Alcohol use	1990	56.09	70.86	41.31
**ASDALYR**	Low-middle SDI	Male	Smoking	1990	46.64	56.47	37.40
**ASDALYR**	Low SDI	Male	Diet low in vegetables	1990	52.21	102.15	-12.71
**ASDALYR**	High SDI	Male	Chewing tobacco	1990	4.37	6.63	2.54
**ASDALYR**	Low SDI	Male	Smoking	1990	36.50	45.20	27.89
**ASDALYR**	Low SDI	Male	Alcohol use	1990	19.13	26.40	9.42
**ASDALYR**	Low-middle SDI	Male	Chewing tobacco	1990	21.25	29.31	13.97
**ASDALYR**	Low-middle SDI	Male	Alcohol use	1990	11.59	15.90	7.40
**ASDR**	Central Europe, Eastern Europe, and Central Asia	Male	Alcohol use	1990	1.97	2.56	1.34
**ASDR**	Latin America and Caribbean	Male	Smoking	1990	3.10	3.69	2.51
**ASDR**	Southeast Asia, East Asia, and Oceania	Male	Smoking	1990	15.88	19.92	12.35
**ASDR**	High-income	Male	Smoking	1990	4.61	5.32	3.79
**ASDR**	Central Europe, Eastern Europe, and Central Asia	Male	Smoking	1990	4.38	5.09	3.62
**ASDR**	Southeast Asia, East Asia, and Oceania	Male	Alcohol use	1990	5.25	7.33	3.67
**ASDR**	Latin America and Caribbean	Male	Chewing tobacco	1990	0.06	0.09	0.03
**ASDR**	Southeast Asia, East Asia, and Oceania	Male	Diet low in vegetables	1990	6.23	12.55	-1.40
**ASDR**	Southeast Asia, East Asia, and Oceania	Male	Chewing tobacco	1990	0.28	0.44	0.15
**ASDR**	High-income	Male	Diet low in vegetables	1990	1.47	2.94	-0.32

**ASDR**	High-income	Male	Alcohol use	1990	2.04	2.63	1.47
**ASDR**	High-income	Male	Chewing tobacco	1990	0.17	0.25	0.10
**ASDR**	Central Europe, Eastern Europe, and Central Asia	Male	Chewing tobacco	1990	0.06	0.10	0.04
**ASDR**	Global	Male	Smoking	1990	6.93	8.29	5.57
**ASDR**	Global	Male	Diet low in vegetables	1990	2.82	5.66	-0.63
**ASDR**	Global	Male	Alcohol use	1990	2.52	3.30	1.83
**ASDR**	Global	Male	Chewing tobacco	1990	0.35	0.50	0.22
**ASDR**	South Asia	Male	Chewing tobacco	1990	1.15	1.56	0.76
**ASDR**	Central Europe, Eastern Europe, and Central Asia	Male	Diet low in vegetables	1990	1.54	3.12	-0.33
**ASDR**	Latin America and Caribbean	Male	Alcohol use	1990	1.11	1.45	0.77
**ASDR**	North Africa and Middle East	Male	Smoking	1990	1.37	1.68	1.05
**ASDR**	Latin America and Caribbean	Male	Diet low in vegetables	1990	1.69	3.27	-0.39
**ASDR**	North Africa and Middle East	Male	Diet low in vegetables	1990	0.49	1.01	-0.10
**ASDR**	South Asia	Male	Diet low in vegetables	1990	1.07	2.16	-0.24
**ASDR**	Sub-Saharan Africa	Male	Diet low in vegetables	1990	2.72	5.26	-0.67
**ASDR**	South Asia	Male	Smoking	1990	1.59	2.00	1.25
**ASDR**	North Africa and Middle East	Male	Chewing tobacco	1990	0.09	0.14	0.05
**ASDR**	Sub-Saharan Africa	Male	Alcohol use	1990	1.39	1.88	0.77
**ASDR**	North Africa and Middle East	Male	Alcohol use	1990	0.06	0.09	0.03
**ASDR**	Sub-Saharan Africa	Male	Smoking	1990	2.09	2.60	1.63
**ASDR**	South Asia	Male	Alcohol use	1990	0.26	0.39	0.11
**ASDR**	Sub-Saharan Africa	Male	Chewing tobacco	1990	0.27	0.42	0.15
**ASDR**	Low-middle SDI	Male	Diet low in vegetables	1990	1.15	2.33	-0.26
**ASDR**	High-middle SDI	Male	Diet low in vegetables	1990	3.78	7.67	-0.82
**ASDR**	Low SDI	Male	Alcohol use	1990	0.68	0.93	0.34
**ASDR**	Middle SDI	Male	Alcohol use	1990	3.32	4.63	2.33
**ASDR**	Low-middle SDI	Male	Smoking	1990	1.87	2.28	1.48
**ASDR**	High-middle SDI	Male	Smoking	1990	10.05	12.36	7.91
**ASDR**	High SDI	Male	Alcohol use	1990	2.09	2.68	1.51

**ASDR**	Low SDI	Male	Diet low in vegetables	1990	2.00	3.90	-0.49
**ASDR**	Low-middle SDI	Male	Chewing tobacco	1990	0.78	1.07	0.51
**ASDR**	High SDI	Male	Diet low in vegetables	1990	1.64	3.30	-0.35
**ASDR**	Middle SDI	Male	Diet low in vegetables	1990	4.36	8.75	-1.00
**ASDR**	High-middle SDI	Male	Chewing tobacco	1990	0.15	0.24	0.08
**ASDR**	Low SDI	Male	Smoking	1990	1.45	1.79	1.09
**ASDR**	Low SDI	Male	Chewing tobacco	1990	0.59	0.84	0.38
**ASDR**	Middle SDI	Male	Smoking	1990	10.49	13.21	8.25
**ASDR**	High-middle SDI	Male	Alcohol use	1990	4.02	5.24	2.88
**ASDR**	High SDI	Male	Smoking	1990	4.99	5.76	4.10
**ASDR**	Low-middle SDI	Male	Alcohol use	1990	0.40	0.55	0.26
**ASDR**	Middle SDI	Male	Chewing tobacco	1990	0.35	0.51	0.21
**ASDR**	High SDI	Male	Chewing tobacco	1990	0.19	0.28	0.11

(C) In 1990, female

measure_name	location_name	sex_name	rei_name	year	val	upper	lower
**ASDALYR**	Global	Female	Alcohol use	1990	4.40	6.07	2.97
**ASDALYR**	Global	Female	Smoking	1990	12.33	15.94	8.82
**ASDALYR**	Global	Female	Diet low in vegetables	1990	26.66	53.99	-5.13
**ASDALYR**	Latin America and Caribbean	Female	Smoking	1990	11.85	15.06	8.98
**ASDALYR**	Global	Female	Chewing tobacco	1990	4.07	5.96	2.48
**ASDALYR**	Latin America and Caribbean	Female	Chewing tobacco	1990	0.38	0.62	0.20
**ASDALYR**	Central Europe, Eastern Europe, and Central Asia	Female	Alcohol use	1990	3.60	5.02	2.41
**ASDALYR**	Southeast Asia, East Asia, and Oceania	Female	Smoking	1990	21.24	30.80	11.01
**ASDALYR**	Southeast Asia, East Asia, and Oceania	Female	Diet low in vegetables	1990	57.25	119.43	-11.16
**ASDALYR**	Latin America and Caribbean	Female	Alcohol use	1990	2.07	2.95	1.32
**ASDALYR**	Latin America and Caribbean	Female	Diet low in vegetables	1990	13.35	26.10	-3.00
**ASDALYR**	Southeast Asia, East Asia, and Oceania	Female	Alcohol use	1990	5.23	8.40	2.52
**ASDALYR**	Central Europe, Eastern Europe, and Central Asia	Female	Diet low in vegetables	1990	9.44	19.30	-1.98

**ASDALYR**	Central Europe, Eastern Europe, and Central Asia	Female	Smoking	1990	3.34	4.16	2.56
**ASDALYR**	High-income	Female	Diet low in vegetables	1990	7.65	15.41	-1.67
**ASDALYR**	High-income	Female	Alcohol use	1990	5.96	7.78	4.24
**ASDALYR**	Southeast Asia, East Asia, and Oceania	Female	Chewing tobacco	1990	2.49	3.98	1.35
**ASDALYR**	Central Europe, Eastern Europe, and Central Asia	Female	Chewing tobacco	1990	0.21	0.33	0.11
**ASDALYR**	High-income	Female	Smoking	1990	13.50	16.52	10.52
**ASDALYR**	Sub-Saharan Africa	Female	Smoking	1990	8.48	11.48	5.41
**ASDALYR**	Sub-Saharan Africa	Female	Alcohol use	1990	8.34	12.14	3.95
**ASDALYR**	Sub-Saharan Africa	Female	Diet low in vegetables	1990	47.09	90.48	-8.92
**ASDALYR**	High-income	Female	Chewing tobacco	1990	0.15	0.25	0.09
**ASDALYR**	Sub-Saharan Africa	Female	Chewing tobacco	1990	5.63	8.77	3.00
**ASDALYR**	North Africa and Middle East	Female	Smoking	1990	3.48	4.90	1.90
**ASDALYR**	South Asia	Female	Smoking	1990	5.53	8.51	3.87
**ASDALYR**	North Africa and Middle East	Female	Alcohol use	1990	0.18	0.29	0.09
**ASDALYR**	North Africa and Middle East	Female	Chewing tobacco	1990	0.94	1.62	0.34
**ASDALYR**	South Asia	Female	Chewing tobacco	1990	21.64	33.99	13.23
**ASDALYR**	South Asia	Female	Alcohol use	1990	0.49	0.87	0.17
**ASDALYR**	North Africa and Middle East	Female	Diet low in vegetables	1990	11.00	23.77	-2.17
**ASDALYR**	South Asia	Female	Diet low in vegetables	1990	24.49	51.12	-6.37
**ASDALYR**	Low SDI	Female	Chewing tobacco	1990	9.76	15.15	5.54
**ASDALYR**	Middle SDI	Female	Diet low in vegetables	1990	45.72	95.63	-8.80
**ASDALYR**	High-middle SDI	Female	Diet low in vegetables	1990	25.67	53.66	-4.95
**ASDALYR**	High-middle SDI	Female	Smoking	1990	12.44	17.12	7.93
**ASDALYR**	Middle SDI	Female	Smoking	1990	15.30	21.37	8.50
**ASDALYR**	Middle SDI	Female	Alcohol use	1990	3.99	6.19	2.09
**ASDALYR**	High-middle SDI	Female	Alcohol use	1990	5.28	7.58	3.47
**ASDALYR**	High SDI	Female	Diet low in vegetables	1990	8.46	16.98	-1.86
**ASDALYR**	Middle SDI	Female	Chewing tobacco	1990	3.83	5.62	2.14
**ASDALYR**	High-middle SDI	Female	Chewing tobacco	1990	0.69	1.17	0.33

**ASDALYR**	High SDI	Female	Smoking	1990	13.73	16.99	10.67
**ASDALYR**	Low-middle SDI	Female	Diet low in vegetables	1990	22.83	46.22	-5.73
**ASDALYR**	High SDI	Female	Alcohol use	1990	5.47	7.20	3.84
**ASDALYR**	Low-middle SDI	Female	Smoking	1990	5.77	7.83	4.22
**ASDALYR**	Low SDI	Female	Diet low in vegetables	1990	42.59	82.21	-9.09
**ASDALYR**	High SDI	Female	Chewing tobacco	1990	0.19	0.30	0.11
**ASDALYR**	Low SDI	Female	Smoking	1990	6.54	8.74	4.74
**ASDALYR**	Low SDI	Female	Alcohol use	1990	4.65	6.93	1.95
**ASDALYR**	Low-middle SDI	Female	Chewing tobacco	1990	15.55	24.52	9.43
**ASDALYR**	Low-middle SDI	Female	Alcohol use	1990	1.13	1.67	0.61
**ASDR**	Central Europe, Eastern Europe, and Central Asia	Female	Alcohol use	1990	0.13	0.18	0.08
**ASDR**	Latin America and Caribbean	Female	Smoking	1990	0.53	0.69	0.39
**ASDR**	Southeast Asia, East Asia, and Oceania	Female	Smoking	1990	1.07	1.57	0.56
**ASDR**	High-income	Female	Smoking	1990	0.62	0.77	0.47
**ASDR**	Central Europe, Eastern Europe, and Central Asia	Female	Smoking	1990	0.13	0.16	0.10
**ASDR**	Southeast Asia, East Asia, and Oceania	Female	Alcohol use	1990	0.21	0.35	0.11
**ASDR**	Latin America and Caribbean	Female	Chewing tobacco	1990	0.02	0.03	0.01
**ASDR**	Southeast Asia, East Asia, and Oceania	Female	Diet low in vegetables	1990	2.49	5.17	-0.48
**ASDR**	Southeast Asia, East Asia, and Oceania	Female	Chewing tobacco	1990	0.11	0.17	0.06
**ASDR**	High-income	Female	Diet low in vegetables	1990	0.36	0.73	-0.08
**ASDR**	High-income	Female	Alcohol use	1990	0.25	0.33	0.17
**ASDR**	High-income	Female	Chewing tobacco	1990	0.01	0.01	0.00
**ASDR**	Central Europe, Eastern Europe, and Central Asia	Female	Chewing tobacco	1990	0.01	0.01	0.00
**ASDR**	Global	Female	Smoking	1990	0.58	0.75	0.41
**ASDR**	Global	Female	Diet low in vegetables	1990	1.09	2.22	-0.21
**ASDR**	Global	Female	Alcohol use	1990	0.18	0.25	0.12
**ASDR**	Global	Female	Chewing tobacco	1990	0.15	0.22	0.09
**ASDR**	South Asia	Female	Chewing tobacco	1990	0.86	1.34	0.52
**ASDR**	Central Europe, Eastern Europe, and Central Asia	Female	Diet low in vegetables	1990	0.41	0.84	-0.09

**ASDR**	Latin America and Caribbean	Female	Alcohol use	1990	0.07	0.11	0.05
**ASDR**	North Africa and Middle East	Female	Smoking	1990	0.14	0.20	0.08
**ASDR**	Latin America and Caribbean	Female	Diet low in vegetables	1990	0.60	1.16	-0.13
**ASDR**	North Africa and Middle East	Female	Diet low in vegetables	1990	0.45	0.94	-0.09
**ASDR**	South Asia	Female	Diet low in vegetables	1990	0.93	1.97	-0.24
**ASDR**	Sub-Saharan Africa	Female	Diet low in vegetables	1990	1.85	3.54	-0.36
**ASDR**	South Asia	Female	Smoking	1990	0.23	0.35	0.16
**ASDR**	North Africa and Middle East	Female	Chewing tobacco	1990	0.04	0.07	0.01
**ASDR**	Sub-Saharan Africa	Female	Alcohol use	1990	0.31	0.44	0.15
**ASDR**	North Africa and Middle East	Female	Alcohol use	1990	0.01	0.01	0.00
**ASDR**	Sub-Saharan Africa	Female	Smoking	1990	0.37	0.50	0.24
**ASDR**	South Asia	Female	Alcohol use	1990	0.02	0.03	0.01
**ASDR**	Sub-Saharan Africa	Female	Chewing tobacco	1990	0.23	0.36	0.12
**ASDR**	Low-middle SDI	Female	Diet low in vegetables	1990	0.89	1.80	-0.22
**ASDR**	High-middle SDI	Female	Diet low in vegetables	1990	1.10	2.29	-0.21
**ASDR**	Low SDI	Female	Alcohol use	1990	0.17	0.25	0.08
**ASDR**	Middle SDI	Female	Alcohol use	1990	0.15	0.24	0.08
**ASDR**	Low-middle SDI	Female	Smoking	1990	0.25	0.33	0.18
**ASDR**	High-middle SDI	Female	Smoking	1990	0.59	0.82	0.37
**ASDR**	High SDI	Female	Alcohol use	1990	0.23	0.30	0.15
**ASDR**	Low SDI	Female	Diet low in vegetables	1990	1.67	3.21	-0.36
**ASDR**	Low-middle SDI	Female	Chewing tobacco	1990	0.61	0.97	0.37
**ASDR**	High SDI	Female	Diet low in vegetables	1990	0.40	0.80	-0.09
**ASDR**	Middle SDI	Female	Diet low in vegetables	1990	1.94	4.03	-0.37
**ASDR**	High-middle SDI	Female	Chewing tobacco	1990	0.03	0.05	0.01
**ASDR**	Low SDI	Female	Smoking	1990	0.27	0.37	0.20
**ASDR**	Low SDI	Female	Chewing tobacco	1990	0.39	0.60	0.22
**ASDR**	Middle SDI	Female	Smoking	1990	0.72	1.02	0.40
**ASDR**	High-middle SDI	Female	Alcohol use	1990	0.21	0.31	0.14

**ASDR**	High SDI	Female	Smoking	1990	0.63	0.80	0.48
**ASDR**	Low-middle SDI	Female	Alcohol use	1990	0.04	0.06	0.02
**ASDR**	Middle SDI	Female	Chewing tobacco	1990	0.16	0.24	0.09
**ASDR**	High SDI	Female	Chewing tobacco	1990	0.01	0.01	0.00

(D) In 2021, both sexes

measure_name	location_name	sex_name	rei_name	year	val	upper	lower
**ASDALYR**	Global	Both	Alcohol use	2021	24.02	31.75	17.33
**ASDALYR**	Global	Both	Smoking	2021	54.26	68.02	41.29
**ASDALYR**	Global	Both	Diet low in vegetables	2021	16.02	33.09	-3.37
**ASDALYR**	Latin America and Caribbean	Both	Smoking	2021	18.08	22.94	13.61
**ASDALYR**	Global	Both	Chewing tobacco	2021	5.46	7.23	3.74
**ASDALYR**	Latin America and Caribbean	Both	Chewing tobacco	2021	0.64	0.93	0.41
**ASDALYR**	Central Europe, Eastern Europe, and Central Asia	Both	Alcohol use	2021	18.28	23.53	13.31
**ASDALYR**	Southeast Asia, East Asia, and Oceania	Both	Smoking	2021	114.29	149.27	84.52
**ASDALYR**	Southeast Asia, East Asia, and Oceania	Both	Diet low in vegetables	2021	10.31	25.51	-1.96
**ASDALYR**	Latin America and Caribbean	Both	Alcohol use	2021	12.72	16.48	9.08
**ASDALYR**	Latin America and Caribbean	Both	Diet low in vegetables	2021	18.16	36.16	-4.13
**ASDALYR**	Southeast Asia, East Asia, and Oceania	Both	Alcohol use	2021	43.65	60.65	29.78
**ASDALYR**	Central Europe, Eastern Europe, and Central Asia	Both	Diet low in vegetables	2021	11.00	22.95	-2.15
**ASDALYR**	Central Europe, Eastern Europe, and Central Asia	Both	Smoking	2021	31.23	36.71	25.13
**ASDALYR**	High-income	Both	Diet low in vegetables	2021	14.58	29.63	-3.14
**ASDALYR**	High-income	Both	Alcohol use	2021	19.39	24.75	14.25
**ASDALYR**	Southeast Asia, East Asia, and Oceania	Both	Chewing tobacco	2021	3.05	4.65	1.77
**ASDALYR**	Central Europe, Eastern Europe, and Central Asia	Both	Chewing tobacco	2021	0.54	0.79	0.32
**ASDALYR**	High-income	Both	Smoking	2021	32.50	39.57	25.69
**ASDALYR**	Sub-Saharan Africa	Both	Smoking	2021	21.06	26.62	16.32
**ASDALYR**	Sub-Saharan Africa	Both	Alcohol use	2021	23.99	31.91	15.70
**ASDALYR**	Sub-Saharan Africa	Both	Diet low in vegetables	2021	51.28	97.52	-10.47

**ASDALYR**	High-income	Both	Chewing tobacco	2021	1.52	2.32	0.88
**ASDALYR**	Sub-Saharan Africa	Both	Chewing tobacco	2021	5.33	7.49	3.53
**ASDALYR**	North Africa and Middle East	Both	Smoking	2021	13.63	17.04	10.40
**ASDALYR**	South Asia	Both	Smoking	2021	14.38	17.91	11.10
**ASDALYR**	North Africa and Middle East	Both	Alcohol use	2021	0.71	1.01	0.41
**ASDALYR**	North Africa and Middle East	Both	Chewing tobacco	2021	1.02	1.47	0.66
**ASDALYR**	South Asia	Both	Chewing tobacco	2021	20.40	26.65	14.41
**ASDALYR**	South Asia	Both	Alcohol use	2021	5.82	8.04	3.46
**ASDALYR**	North Africa and Middle East	Both	Diet low in vegetables	2021	5.70	11.99	-1.13
**ASDALYR**	South Asia	Both	Diet low in vegetables	2021	20.83	42.95	-4.50
**ASDALYR**	Low SDI	Both	Chewing tobacco	2021	9.85	13.10	6.83
**ASDALYR**	Middle SDI	Both	Diet low in vegetables	2021	15.18	33.25	-3.34
**ASDALYR**	High-middle SDI	Both	Diet low in vegetables	2021	7.56	17.54	-1.46
**ASDALYR**	High-middle SDI	Both	Smoking	2021	86.54	113.38	64.83
**ASDALYR**	Middle SDI	Both	Smoking	2021	69.51	90.83	51.30
**ASDALYR**	Middle SDI	Both	Alcohol use	2021	27.44	38.07	19.11
**ASDALYR**	High-middle SDI	Both	Alcohol use	2021	37.14	52.12	26.12
**ASDALYR**	High SDI	Both	Diet low in vegetables	2021	14.61	29.63	-3.11
**ASDALYR**	Middle SDI	Both	Chewing tobacco	2021	5.01	6.81	3.36
**ASDALYR**	High-middle SDI	Both	Chewing tobacco	2021	1.90	3.06	1.00
**ASDALYR**	High SDI	Both	Smoking	2021	37.57	45.36	29.85
**ASDALYR**	Low-middle SDI	Both	Diet low in vegetables	2021	21.86	44.45	-4.63
**ASDALYR**	High SDI	Both	Alcohol use	2021	21.16	26.95	15.76
**ASDALYR**	Low-middle SDI	Both	Smoking	2021	17.83	21.83	13.95
**ASDALYR**	Low SDI	Both	Diet low in vegetables	2021	37.33	72.77	-7.59
**ASDALYR**	High SDI	Both	Chewing tobacco	2021	1.69	2.57	0.99
**ASDALYR**	Low SDI	Both	Smoking	2021	14.79	18.59	11.21
**ASDALYR**	Low SDI	Both	Alcohol use	2021	11.88	16.55	7.45
**ASDALYR**	Low-middle SDI	Both	Chewing tobacco	2021	14.07	18.83	9.92

**ASDALYR**	Low-middle SDI	Both	Alcohol use	2021	8.00	10.66	5.52
**ASDRs**	Central Europe, Eastern Europe, and Central Asia	Both	Alcohol use	2021	0.62	0.81	0.43
**ASDRs**	Latin America and Caribbean	Both	Smoking	2021	0.75	0.96	0.57
**ASDRs**	Southeast Asia, East Asia, and Oceania	Both	Smoking	2021	5.18	6.71	3.85
**ASDRs**	High-income	Both	Smoking	2021	1.44	1.77	1.12
**ASDRs**	Central Europe, Eastern Europe, and Central Asia	Both	Smoking	2021	1.15	1.36	0.92
**ASDRs**	Southeast Asia, East Asia, and Oceania	Both	Alcohol use	2021	1.73	2.40	1.18
**ASDRs**	Latin America and Caribbean	Both	Chewing tobacco	2021	0.03	0.04	0.02
**ASDRs**	Southeast Asia, East Asia, and Oceania	Both	Diet low in vegetables	2021	0.49	1.23	-0.09
**ASDRs**	Southeast Asia, East Asia, and Oceania	Both	Chewing tobacco	2021	0.12	0.18	0.07
**ASDRs**	High-income	Both	Diet low in vegetables	2021	0.63	1.29	-0.14
**ASDRs**	High-income	Both	Alcohol use	2021	0.78	0.99	0.57
**ASDRs**	High-income	Both	Chewing tobacco	2021	0.06	0.10	0.04
**ASDRs**	Central Europe, Eastern Europe, and Central Asia	Both	Chewing tobacco	2021	0.02	0.03	0.01
**ASDRs**	Global	Both	Smoking	2021	2.38	2.98	1.81
**ASDRs**	Global	Both	Diet low in vegetables	2021	0.66	1.38	-0.14
**ASDRs**	Global	Both	Alcohol use	2021	0.93	1.24	0.68
**ASDRs**	Global	Both	Chewing tobacco	2021	0.21	0.27	0.14
**ASDRs**	South Asia	Both	Chewing tobacco	2021	0.81	1.05	0.57
**ASDRs**	Central Europe, Eastern Europe, and Central Asia	Both	Diet low in vegetables	2021	0.42	0.87	-0.08
**ASDRs**	Latin America and Caribbean	Both	Alcohol use	2021	0.44	0.59	0.31
**ASDRs**	North Africa and Middle East	Both	Smoking	2021	0.62	0.78	0.47
**ASDRs**	Latin America and Caribbean	Both	Diet low in vegetables	2021	0.73	1.45	-0.17
**ASDRs**	North Africa and Middle East	Both	Diet low in vegetables	2021	0.25	0.54	-0.05
**ASDRs**	South Asia	Both	Diet low in vegetables	2021	0.81	1.68	-0.18
**ASDRs**	Sub-Saharan Africa	Both	Diet low in vegetables	2021	2.08	3.98	-0.42
**ASDRs**	South Asia	Both	Smoking	2021	0.60	0.75	0.46
**ASDRs**	North Africa and Middle East	Both	Chewing tobacco	2021	0.04	0.06	0.03
**ASDRs**	Sub-Saharan Africa	Both	Alcohol use	2021	0.88	1.18	0.58

**ASDRs**	North Africa and Middle East	Both	Alcohol use	2021	0.03	0.04	0.01
**ASDRs**	Sub-Saharan Africa	Both	Smoking	2021	0.82	1.04	0.63
**ASDRs**	South Asia	Both	Alcohol use	2021	0.20	0.28	0.12
**ASDRs**	Sub-Saharan Africa	Both	Chewing tobacco	2021	0.22	0.30	0.14
**ASDRs**	Low-middle SDI	Both	Diet low in vegetables	2021	0.86	1.74	-0.18
**ASDRs**	High-middle SDI	Both	Diet low in vegetables	2021	0.34	0.79	-0.07
**ASDRs**	Low SDI	Both	Alcohol use	2021	0.43	0.59	0.27
**ASDRs**	Middle SDI	Both	Alcohol use	2021	1.08	1.50	0.75
**ASDRs**	Low-middle SDI	Both	Smoking	2021	0.73	0.89	0.57
**ASDRs**	High-middle SDI	Both	Smoking	2021	3.70	4.83	2.77
**ASDRs**	High SDI	Both	Alcohol use	2021	0.84	1.07	0.62
**ASDRs**	Low SDI	Both	Diet low in vegetables	2021	1.48	2.88	-0.30
**ASDRs**	Low-middle SDI	Both	Chewing tobacco	2021	0.56	0.74	0.39
**ASDRs**	High SDI	Both	Diet low in vegetables	2021	0.64	1.29	-0.14
**ASDRs**	Middle SDI	Both	Diet low in vegetables	2021	0.66	1.48	-0.14
**ASDRs**	High-middle SDI	Both	Chewing tobacco	2021	0.07	0.11	0.04
**ASDRs**	Low SDI	Both	Smoking	2021	0.59	0.74	0.45
**ASDRs**	Low SDI	Both	Chewing tobacco	2021	0.40	0.52	0.28
**ASDRs**	Middle SDI	Both	Smoking	2021	3.14	4.11	2.31
**ASDRs**	High-middle SDI	Both	Alcohol use	2021	1.40	1.96	1.00
**ASDRs**	High SDI	Both	Smoking	2021	1.65	2.00	1.29
**ASDRs**	Low-middle SDI	Both	Alcohol use	2021	0.28	0.37	0.19
**ASDRs**	Middle SDI	Both	Chewing tobacco	2021	0.20	0.27	0.14
**ASDRs**	High SDI	Both	Chewing tobacco	2021	0.07	0.11	0.04

(E) In 2021, male

measure_name	location_name	sex_name	rei_name	year	val	upper	lower
**ASDALYR**	Global	Male	Alcohol use	2021	47.54	63.48	34.39
**ASDALYR**	Global	Male	Smoking	2021	109.33	136.76	83.18

**ASDALYR**	Global	Male	Diet low in vegetables	2021	23.72	49.91	-5.05
**ASDALYR**	Latin America and Caribbean	Male	Smoking	2021	34.31	43.17	25.83
**ASDALYR**	Global	Male	Chewing tobacco	2021	7.88	11.57	4.86
**ASDALYR**	Latin America and Caribbean	Male	Chewing tobacco	2021	1.12	1.74	0.62
**ASDALYR**	Central Europe, Eastern Europe, and Central Asia	Male	Alcohol use	2021	38.59	49.54	27.77
**ASDALYR**	Southeast Asia, East Asia, and Oceania	Male	Smoking	2021	234.65	306.53	173.50
**ASDALYR**	Southeast Asia, East Asia, and Oceania	Male	Diet low in vegetables	2021	17.18	43.04	-3.48
**ASDALYR**	Latin America and Caribbean	Male	Alcohol use	2021	25.46	32.99	18.20
**ASDALYR**	Latin America and Caribbean	Male	Diet low in vegetables	2021	30.46	60.78	-6.92
**ASDALYR**	Southeast Asia, East Asia, and Oceania	Male	Alcohol use	2021	88.60	123.32	60.39
**ASDALYR**	Central Europe, Eastern Europe, and Central Asia	Male	Diet low in vegetables	2021	20.74	42.92	-4.06
**ASDALYR**	Central Europe, Eastern Europe, and Central Asia	Male	Smoking	2021	68.94	81.12	55.51
**ASDALYR**	High-income	Male	Diet low in vegetables	2021	24.75	50.62	-5.33
**ASDALYR**	High-income	Male	Alcohol use	2021	35.91	45.54	26.38
**ASDALYR**	Southeast Asia, East Asia, and Oceania	Male	Chewing tobacco	2021	5.00	8.29	2.62
**ASDALYR**	Central Europe, Eastern Europe, and Central Asia	Male	Chewing tobacco	2021	1.05	1.61	0.59
**ASDALYR**	High-income	Male	Smoking	2021	60.48	73.12	48.01
**ASDALYR**	Sub-Saharan Africa	Male	Smoking	2021	39.39	49.48	30.66
**ASDALYR**	Sub-Saharan Africa	Male	Alcohol use	2021	42.98	56.50	28.89
**ASDALYR**	Sub-Saharan Africa	Male	Diet low in vegetables	2021	66.11	126.56	-13.83
**ASDALYR**	High-income	Male	Chewing tobacco	2021	3.09	4.77	1.74
**ASDALYR**	Sub-Saharan Africa	Male	Chewing tobacco	2021	6.09	9.42	3.53
**ASDALYR**	North Africa and Middle East	Male	Smoking	2021	25.19	31.32	19.35
**ASDALYR**	South Asia	Male	Smoking	2021	26.63	32.75	20.59
**ASDALYR**	North Africa and Middle East	Male	Alcohol use	2021	1.31	1.87	0.75
**ASDALYR**	North Africa and Middle East	Male	Chewing tobacco	2021	1.56	2.39	0.90
**ASDALYR**	South Asia	Male	Chewing tobacco	2021	26.39	37.06	16.65
**ASDALYR**	South Asia	Male	Alcohol use	2021	11.16	15.61	6.52
**ASDALYR**	North Africa and Middle East	Male	Diet low in vegetables	2021	6.55	13.77	-1.44

**ASDALYR**	South Asia	Male	Diet low in vegetables	2021	24.47	48.91	-5.13
**ASDALYR**	Low SDI	Male	Chewing tobacco	2021	12.82	17.87	8.02
**ASDALYR**	Middle SDI	Male	Diet low in vegetables	2021	23.49	52.40	-5.21
**ASDALYR**	High-middle SDI	Male	Diet low in vegetables	2021	13.23	30.94	-2.61
**ASDALYR**	High-middle SDI	Male	Smoking	2021	181.30	239.30	134.77
**ASDALYR**	Middle SDI	Male	Smoking	2021	142.25	185.81	104.67
**ASDALYR**	Middle SDI	Male	Alcohol use	2021	55.42	77.19	38.54
**ASDALYR**	High-middle SDI	Male	Alcohol use	2021	76.36	107.56	53.69
**ASDALYR**	High SDI	Male	Diet low in vegetables	2021	24.91	50.57	-5.30
**ASDALYR**	Middle SDI	Male	Chewing tobacco	2021	7.47	10.70	4.41
**ASDALYR**	High-middle SDI	Male	Chewing tobacco	2021	3.50	5.91	1.72
**ASDALYR**	High SDI	Male	Smoking	2021	70.75	85.01	56.44
**ASDALYR**	Low-middle SDI	Male	Diet low in vegetables	2021	27.34	54.98	-5.83
**ASDALYR**	High SDI	Male	Alcohol use	2021	39.52	50.08	29.42
**ASDALYR**	Low-middle SDI	Male	Smoking	2021	33.92	41.45	26.58
**ASDALYR**	Low SDI	Male	Diet low in vegetables	2021	43.37	84.33	-8.95
**ASDALYR**	High SDI	Male	Chewing tobacco	2021	3.38	5.22	1.93
**ASDALYR**	Low SDI	Male	Smoking	2021	26.05	32.81	19.69
**ASDALYR**	Low SDI	Male	Alcohol use	2021	19.89	27.49	13.02
**ASDALYR**	Low-middle SDI	Male	Chewing tobacco	2021	17.65	24.80	11.06
**ASDALYR**	Low-middle SDI	Male	Alcohol use	2021	15.13	20.21	10.34
**ASDRs**	Central Europe, Eastern Europe, and Central Asia	Male	Alcohol use	2021	1.36	1.77	0.95
**ASDRs**	Latin America and Caribbean	Male	Smoking	2021	1.44	1.83	1.09
**ASDRs**	Southeast Asia, East Asia, and Oceania	Male	Smoking	2021	10.98	14.23	8.15
**ASDRs**	High-income	Male	Smoking	2021	2.74	3.33	2.14
**ASDRs**	Central Europe, Eastern Europe, and Central Asia	Male	Smoking	2021	2.65	3.13	2.13
**ASDRs**	Southeast Asia, East Asia, and Oceania	Male	Alcohol use	2021	3.61	5.02	2.45
**ASDRs**	Latin America and Caribbean	Male	Chewing tobacco	2021	0.05	0.08	0.03
**ASDRs**	Southeast Asia, East Asia, and Oceania	Male	Diet low in vegetables	2021	0.83	2.20	-0.16

**ASDRs**	Southeast Asia, East Asia, and Oceania	Male	Chewing tobacco	2021	0.19	0.31	0.10
**ASDRs**	High-income	Male	Diet low in vegetables	2021	1.08	2.21	-0.24
**ASDRs**	High-income	Male	Alcohol use	2021	1.47	1.86	1.07
**ASDRs**	High-income	Male	Chewing tobacco	2021	0.13	0.21	0.08
**ASDRs**	Central Europe, Eastern Europe, and Central Asia	Male	Chewing tobacco	2021	0.04	0.06	0.02
**ASDRs**	Global	Male	Smoking	2021	4.90	6.13	3.74
**ASDRs**	Global	Male	Diet low in vegetables	2021	1.00	2.10	-0.22
**ASDRs**	Global	Male	Alcohol use	2021	1.89	2.51	1.36
**ASDRs**	Global	Male	Chewing tobacco	2021	0.30	0.43	0.18
**ASDRs**	South Asia	Male	Chewing tobacco	2021	1.02	1.42	0.64
**ASDRs**	Central Europe, Eastern Europe, and Central Asia	Male	Diet low in vegetables	2021	0.80	1.64	-0.16
**ASDRs**	Latin America and Caribbean	Male	Alcohol use	2021	0.90	1.18	0.64
**ASDRs**	North Africa and Middle East	Male	Smoking	2021	1.17	1.46	0.89
**ASDRs**	Latin America and Caribbean	Male	Diet low in vegetables	2021	1.21	2.41	-0.27
**ASDRs**	North Africa and Middle East	Male	Diet low in vegetables	2021	0.30	0.63	-0.07
**ASDRs**	South Asia	Male	Diet low in vegetables	2021	0.96	1.93	-0.20
**ASDRs**	Sub-Saharan Africa	Male	Diet low in vegetables	2021	2.59	4.96	-0.54
**ASDRs**	South Asia	Male	Smoking	2021	1.12	1.38	0.86
**ASDRs**	North Africa and Middle East	Male	Chewing tobacco	2021	0.06	0.09	0.03
**ASDRs**	Sub-Saharan Africa	Male	Alcohol use	2021	1.58	2.10	1.06
**ASDRs**	North Africa and Middle East	Male	Alcohol use	2021	0.05	0.07	0.03
**ASDRs**	Sub-Saharan Africa	Male	Smoking	2021	1.52	1.89	1.18
**ASDRs**	South Asia	Male	Alcohol use	2021	0.39	0.55	0.23
**ASDRs**	Sub-Saharan Africa	Male	Chewing tobacco	2021	0.23	0.35	0.13
**ASDRs**	Low-middle SDI	Male	Diet low in vegetables	2021	1.07	2.14	-0.23
**ASDRs**	High-middle SDI	Male	Diet low in vegetables	2021	0.60	1.47	-0.12
**ASDRs**	Low SDI	Male	Alcohol use	2021	0.71	0.98	0.47
**ASDRs**	Middle SDI	Male	Alcohol use	2021	2.22	3.10	1.54
**ASDRs**	Low-middle SDI	Male	Smoking	2021	1.40	1.70	1.09

**ASDRs**	High-middle SDI	Male	Smoking	2021	8.01	10.55	5.99
**ASDRs**	High SDI	Male	Alcohol use	2021	1.61	2.03	1.18
**ASDRs**	Low SDI	Male	Diet low in vegetables	2021	1.67	3.26	-0.34
**ASDRs**	Low-middle SDI	Male	Chewing tobacco	2021	0.68	0.97	0.42
**ASDRs**	High SDI	Male	Diet low in vegetables	2021	1.09	2.23	-0.24
**ASDRs**	Middle SDI	Male	Diet low in vegetables	2021	1.04	2.42	-0.23
**ASDRs**	High-middle SDI	Male	Chewing tobacco	2021	0.13	0.21	0.06
**ASDRs**	Low SDI	Male	Smoking	2021	1.04	1.30	0.78
**ASDRs**	Low SDI	Male	Chewing tobacco	2021	0.49	0.70	0.30
**ASDRs**	Middle SDI	Male	Smoking	2021	6.59	8.61	4.81
**ASDRs**	High-middle SDI	Male	Alcohol use	2021	2.97	4.17	2.09
**ASDRs**	High SDI	Male	Smoking	2021	3.17	3.82	2.49
**ASDRs**	Low-middle SDI	Male	Alcohol use	2021	0.53	0.70	0.36
**ASDRs**	Middle SDI	Male	Chewing tobacco	2021	0.29	0.41	0.17
**ASDRs**	High SDI	Male	Chewing tobacco	2021	0.14	0.22	0.08

(F) In 2021, female

measure_name	location_name	sex_name	rei_name	year	val	upper	lower
**ASDALYR**	Global	Female	Alcohol use	2021	2.43	3.31	1.69
**ASDALYR**	Global	Female	Smoking	2021	5.19	6.86	3.66
**ASDALYR**	Global	Female	Diet low in vegetables	2021	9.12	18.55	-1.91
**ASDALYR**	Latin America and Caribbean	Female	Smoking	2021	4.17	5.44	3.07
**ASDALYR**	Global	Female	Chewing tobacco	2021	3.20	4.84	1.97
**ASDALYR**	Latin America and Caribbean	Female	Chewing tobacco	2021	0.23	0.36	0.13
**ASDALYR**	Central Europe, Eastern Europe, and Central Asia	Female	Alcohol use	2021	2.12	2.95	1.36
**ASDALYR**	Southeast Asia, East Asia, and Oceania	Female	Smoking	2021	6.23	9.37	3.67
**ASDALYR**	Southeast Asia, East Asia, and Oceania	Female	Diet low in vegetables	2021	4.25	10.11	-0.86
**ASDALYR**	Latin America and Caribbean	Female	Alcohol use	2021	1.49	2.17	0.98
**ASDALYR**	Latin America and Caribbean	Female	Diet low in vegetables	2021	7.44	14.80	-1.70

**ASDALYR**	Southeast Asia, East Asia, and Oceania	Female	Alcohol use	2021	1.79	2.75	0.99
**ASDALYR**	Central Europe, Eastern Europe, and Central Asia	Female	Diet low in vegetables	2021	3.49	7.29	-0.71
**ASDALYR**	Central Europe, Eastern Europe, and Central Asia	Female	Smoking	2021	2.52	3.18	1.94
**ASDALYR**	High-income	Female	Diet low in vegetables	2021	5.47	11.07	-1.18
**ASDALYR**	High-income	Female	Alcohol use	2021	4.43	5.79	3.07
**ASDALYR**	Southeast Asia, East Asia, and Oceania	Female	Chewing tobacco	2021	1.17	1.84	0.64
**ASDALYR**	Central Europe, Eastern Europe, and Central Asia	Female	Chewing tobacco	2021	0.13	0.20	0.07
**ASDALYR**	High-income	Female	Smoking	2021	7.77	9.87	5.86
**ASDALYR**	Sub-Saharan Africa	Female	Smoking	2021	4.90	6.80	3.29
**ASDALYR**	Sub-Saharan Africa	Female	Alcohol use	2021	7.15	10.74	3.94
**ASDALYR**	Sub-Saharan Africa	Female	Diet low in vegetables	2021	38.00	75.57	-7.07
**ASDALYR**	High-income	Female	Chewing tobacco	2021	0.12	0.19	0.07
**ASDALYR**	Sub-Saharan Africa	Female	Chewing tobacco	2021	4.58	7.33	2.45
**ASDALYR**	North Africa and Middle East	Female	Smoking	2021	1.85	2.54	1.09
**ASDALYR**	South Asia	Female	Smoking	2021	2.59	4.24	1.75
**ASDALYR**	North Africa and Middle East	Female	Alcohol use	2021	0.07	0.11	0.04
**ASDALYR**	North Africa and Middle East	Female	Chewing tobacco	2021	0.46	0.78	0.18
**ASDALYR**	South Asia	Female	Chewing tobacco	2021	14.50	22.56	8.88
**ASDALYR**	South Asia	Female	Alcohol use	2021	0.57	0.93	0.31
**ASDALYR**	North Africa and Middle East	Female	Diet low in vegetables	2021	4.87	10.63	-0.96
**ASDALYR**	South Asia	Female	Diet low in vegetables	2021	17.31	38.78	-4.11
**ASDALYR**	Low SDI	Female	Chewing tobacco	2021	6.95	11.31	3.84
**ASDALYR**	Middle SDI	Female	Diet low in vegetables	2021	7.68	16.63	-1.62
**ASDALYR**	High-middle SDI	Female	Diet low in vegetables	2021	2.73	6.18	-0.52
**ASDALYR**	High-middle SDI	Female	Smoking	2021	5.55	8.00	3.69
**ASDALYR**	Middle SDI	Female	Smoking	2021	4.33	6.20	2.72
**ASDALYR**	Middle SDI	Female	Alcohol use	2021	1.59	2.29	0.98
**ASDALYR**	High-middle SDI	Female	Alcohol use	2021	2.18	3.14	1.40
**ASDALYR**	High SDI	Female	Diet low in vegetables	2021	5.26	10.70	-1.12

**ASDALYR**	Middle SDI	Female	Chewing tobacco	2021	2.68	3.90	1.56
**ASDALYR**	High-middle SDI	Female	Chewing tobacco	2021	0.43	0.73	0.22
**ASDALYR**	High SDI	Female	Smoking	2021	7.76	9.85	5.83
**ASDALYR**	Low-middle SDI	Female	Diet low in vegetables	2021	16.70	35.36	-3.67
**ASDALYR**	High SDI	Female	Alcohol use	2021	4.21	5.52	2.89
**ASDALYR**	Low-middle SDI	Female	Smoking	2021	2.91	4.18	2.09
**ASDALYR**	Low SDI	Female	Diet low in vegetables	2021	31.36	60.85	-6.39
**ASDALYR**	High SDI	Female	Chewing tobacco	2021	0.15	0.23	0.08
**ASDALYR**	Low SDI	Female	Smoking	2021	3.92	5.54	2.71
**ASDALYR**	Low SDI	Female	Alcohol use	2021	4.07	6.33	2.20
**ASDALYR**	Low-middle SDI	Female	Chewing tobacco	2021	10.69	17.14	6.46
**ASDALYR**	Low-middle SDI	Female	Alcohol use	2021	1.24	1.83	0.76
**ASDRs**	Central Europe, Eastern Europe, and Central Asia	Female	Alcohol use	2021	0.07	0.10	0.05
**ASDRs**	Latin America and Caribbean	Female	Smoking	2021	0.18	0.24	0.13
**ASDRs**	Southeast Asia, East Asia, and Oceania	Female	Smoking	2021	0.35	0.53	0.20
**ASDRs**	High-income	Female	Smoking	2021	0.36	0.46	0.26
**ASDRs**	Central Europe, Eastern Europe, and Central Asia	Female	Smoking	2021	0.09	0.12	0.07
**ASDRs**	Southeast Asia, East Asia, and Oceania	Female	Alcohol use	2021	0.08	0.13	0.04
**ASDRs**	Latin America and Caribbean	Female	Chewing tobacco	2021	0.01	0.02	0.01
**ASDRs**	Southeast Asia, East Asia, and Oceania	Female	Diet low in vegetables	2021	0.22	0.54	-0.04
**ASDRs**	Southeast Asia, East Asia, and Oceania	Female	Chewing tobacco	2021	0.06	0.09	0.03
**ASDRs**	High-income	Female	Diet low in vegetables	2021	0.26	0.52	-0.06
**ASDRs**	High-income	Female	Alcohol use	2021	0.18	0.24	0.13
**ASDRs**	High-income	Female	Chewing tobacco	2021	0.01	0.01	0.00
**ASDRs**	Central Europe, Eastern Europe, and Central Asia	Female	Chewing tobacco	2021	0.00	0.01	0.00
**ASDRs**	Global	Female	Smoking	2021	0.26	0.35	0.18
**ASDRs**	Global	Female	Diet low in vegetables	2021	0.38	0.79	-0.08
**ASDRs**	Global	Female	Alcohol use	2021	0.10	0.14	0.07
**ASDRs**	Global	Female	Chewing tobacco	2021	0.13	0.19	0.08

**ASDRs**	South Asia	Female	Chewing tobacco	2021	0.60	0.93	0.37
**ASDRs**	Central Europe, Eastern Europe, and Central Asia	Female	Diet low in vegetables	2021	0.15	0.32	-0.03
**ASDRs**	Latin America and Caribbean	Female	Alcohol use	2021	0.05	0.08	0.03
**ASDRs**	North Africa and Middle East	Female	Smoking	2021	0.08	0.11	0.05
**ASDRs**	Latin America and Caribbean	Female	Diet low in vegetables	2021	0.33	0.65	-0.08
**ASDRs**	North Africa and Middle East	Female	Diet low in vegetables	2021	0.21	0.45	-0.04
**ASDRs**	South Asia	Female	Diet low in vegetables	2021	0.67	1.51	-0.16
**ASDRs**	Sub-Saharan Africa	Female	Diet low in vegetables	2021	1.63	3.21	-0.30
**ASDRs**	South Asia	Female	Smoking	2021	0.11	0.20	0.07
**ASDRs**	North Africa and Middle East	Female	Chewing tobacco	2021	0.02	0.03	0.01
**ASDRs**	Sub-Saharan Africa	Female	Alcohol use	2021	0.28	0.42	0.16
**ASDRs**	North Africa and Middle East	Female	Alcohol use	2021	0.00	0.00	0.00
**ASDRs**	Sub-Saharan Africa	Female	Smoking	2021	0.22	0.31	0.15
**ASDRs**	South Asia	Female	Alcohol use	2021	0.02	0.03	0.01
**ASDRs**	Sub-Saharan Africa	Female	Chewing tobacco	2021	0.21	0.34	0.11
**ASDRs**	Low-middle SDI	Female	Diet low in vegetables	2021	0.67	1.41	-0.15
**ASDRs**	High-middle SDI	Female	Diet low in vegetables	2021	0.14	0.33	-0.03
**ASDRs**	Low SDI	Female	Alcohol use	2021	0.15	0.24	0.08
**ASDRs**	Middle SDI	Female	Alcohol use	2021	0.07	0.10	0.04
**ASDRs**	Low-middle SDI	Female	Smoking	2021	0.13	0.19	0.09
**ASDRs**	High-middle SDI	Female	Smoking	2021	0.29	0.43	0.18
**ASDRs**	High SDI	Female	Alcohol use	2021	0.18	0.23	0.12
**ASDRs**	Low SDI	Female	Diet low in vegetables	2021	1.30	2.52	-0.26
**ASDRs**	Low-middle SDI	Female	Chewing tobacco	2021	0.44	0.71	0.27
**ASDRs**	High SDI	Female	Diet low in vegetables	2021	0.25	0.51	-0.05
**ASDRs**	Middle SDI	Female	Diet low in vegetables	2021	0.35	0.78	-0.07
**ASDRs**	High-middle SDI	Female	Chewing tobacco	2021	0.02	0.03	0.01
**ASDRs**	Low SDI	Female	Smoking	2021	0.18	0.25	0.12
**ASDRs**	Low SDI	Female	Chewing tobacco	2021	0.30	0.49	0.17

**ASDRs**	Middle SDI	Female	Smoking	2021	0.22	0.32	0.13
**ASDRs**	High-middle SDI	Female	Alcohol use	2021	0.09	0.14	0.06
**ASDRs**	High SDI	Female	Smoking	2021	0.36	0.47	0.27
**ASDRs**	Low-middle SDI	Female	Alcohol use	2021	0.04	0.07	0.03
**ASDRs**	Middle SDI	Female	Chewing tobacco	2021	0.12	0.17	0.07
**ASDRs**	High SDI	Female	Chewing tobacco	2021	0.01	0.01	0.00

(G) Proportion of contribution of four most detailed risks for ASRs in males, females, and both sexes

**Measure**	**Location**	**Sex**	**Cause**	**Rei_name**	**Metric**	**Year**	**Val**	**upper**	**lower**
**ASDR**	Global	Male	Esophageal cancer	Smoking	Percent	1990	51.21%	58.70%	42.86%
**ASDR**	Global	Female	Esophageal cancer	Smoking	Percent	1990	11.23%	14.24%	8.53%
**ASDR**	Global	Both	Esophageal cancer	Smoking	Percent	1990	38.33%	44.54%	31.84%
**ASDR**	Global	Male	Esophageal cancer	High alcohol use	Percent	1990	18.65%	23.54%	14.06%
**ASDR**	Global	Female	Esophageal cancer	High alcohol use	Percent	1990	3.52%	4.80%	2.39%
**ASDR**	Global	Both	Esophageal cancer	High alcohol use	Percent	1990	14.09%	18.14%	10.56%
**ASDR**	Global	Male	Esophageal cancer	Diet low in vegetables	Percent	1990	20.81%	41.18%	-4.64%
**ASDR**	Global	Female	Esophageal cancer	Diet low in vegetables	Percent	1990	21.12%	41.78%	-4.70%
**ASDR**	Global	Both	Esophageal cancer	Diet low in vegetables	Percent	1990	20.90%	41.32%	-4.65%
**ASDR**	Global	Male	Esophageal cancer	Chewing tobacco	Percent	1990	2.60%	3.65%	1.64%
**ASDR**	Global	Female	Esophageal cancer	Chewing tobacco	Percent	1990	2.95%	4.77%	1.71%
**ASDR**	Global	Both	Esophageal cancer	Chewing tobacco	Percent	1990	2.70%	3.51%	1.85%
**ASDR**	Southeast Asia, East Asia, and Oceania	Male	Esophageal cancer	Smoking	Percent	1990	54.10%	62.08%	45.31%
**ASDR**	Southeast Asia, East Asia, and Oceania	Female	Esophageal cancer	Smoking	Percent	1990	9.05%	11.87%	6.77%
**ASDR**	Southeast Asia, East Asia, and Oceania	Both	Esophageal cancer	Smoking	Percent	1990	39.58%	46.61%	32.34%
**ASDR**	Southeast Asia, East Asia, and Oceania	Male	Esophageal cancer	High alcohol use	Percent	1990	17.88%	22.76%	13.28%
**ASDR**	Southeast Asia, East Asia, and Oceania	Female	Esophageal cancer	High alcohol use	Percent	1990	1.80%	2.64%	1.08%
**ASDR**	Southeast Asia, East Asia, and Oceania	Both	Esophageal cancer	High alcohol use	Percent	1990	13.05%	17.16%	9.55%
**ASDR**	Southeast Asia, East Asia, and Oceania	Male	Esophageal cancer	Diet low in vegetables	Percent	1990	21.20%	42.10%	-4.75%
**ASDR**	Southeast Asia, East Asia, and Oceania	Female	Esophageal cancer	Diet low in vegetables	Percent	1990	21.06%	41.76%	-4.69%

**ASDR**	Southeast Asia, East Asia, and Oceania	Both	Esophageal cancer	Diet low in vegetables	Percent	1990	21.16%	41.93%	-4.71%
**ASDR**	Southeast Asia, East Asia, and Oceania	Male	Esophageal cancer	Chewing tobacco	Percent	1990	0.95%	1.44%	0.51%
**ASDR**	Southeast Asia, East Asia, and Oceania	Female	Esophageal cancer	Chewing tobacco	Percent	1990	0.95%	1.55%	0.54%
**ASDR**	Southeast Asia, East Asia, and Oceania	Both	Esophageal cancer	Chewing tobacco	Percent	1990	0.97%	1.36%	0.64%
**ASDR**	Central Europe, Eastern Europe, and Central Asia	Male	Esophageal cancer	Smoking	Percent	1990	50.57%	58.44%	42.05%
**ASDR**	Central Europe, Eastern Europe, and Central Asia	Female	Esophageal cancer	Smoking	Percent	1990	5.31%	6.71%	3.97%
**ASDR**	Central Europe, Eastern Europe, and Central Asia	Both	Esophageal cancer	Smoking	Percent	1990	35.86%	41.55%	29.79%
**ASDR**	Central Europe, Eastern Europe, and Central Asia	Male	Esophageal cancer	High alcohol use	Percent	1990	22.75%	29.33%	15.48%
**ASDR**	Central Europe, Eastern Europe, and Central Asia	Female	Esophageal cancer	High alcohol use	Percent	1990	5.23%	7.57%	3.20%
**ASDR**	Central Europe, Eastern Europe, and Central Asia	Both	Esophageal cancer	High alcohol use	Percent	1990	17.83%	22.90%	12.29%
**ASDR**	Central Europe, Eastern Europe, and Central Asia	Male	Esophageal cancer	Diet low in vegetables	Percent	1990	17.82%	35.88%	-3.91%
**ASDR**	Central Europe, Eastern Europe, and Central Asia	Female	Esophageal cancer	Diet low in vegetables	Percent	1990	17.18%	34.61%	-3.70%
**ASDR**	Central Europe, Eastern Europe, and Central Asia	Both	Esophageal cancer	Diet low in vegetables	Percent	1990	17.57%	35.29%	-3.83%
**ASDR**	Central Europe, Eastern Europe, and Central Asia	Male	Esophageal cancer	Chewing tobacco	Percent	1990	0.73%	1.11%	0.41%
**ASDR**	Central Europe, Eastern Europe, and Central Asia	Female	Esophageal cancer	Chewing tobacco	Percent	1990	0.35%	0.56%	0.20%
**ASDR**	Central Europe, Eastern Europe, and Central Asia	Both	Esophageal cancer	Chewing tobacco	Percent	1990	0.62%	0.89%	0.38%
**ASDR**	High-income	Male	Esophageal cancer	Smoking	Percent	1990	56.70%	65.34%	46.63%
**ASDR**	High-income	Female	Esophageal cancer	Smoking	Percent	1990	31.92%	39.47%	24.30%
**ASDR**	High-income	Both	Esophageal cancer	Smoking	Percent	1990	50.14%	58.25%	40.82%
**ASDR**	High-income	Male	Esophageal cancer	High alcohol use	Percent	1990	25.10%	32.20%	17.98%
**ASDR**	High-income	Female	Esophageal cancer	High alcohol use	Percent	1990	12.81%	17.07%	8.60%
**ASDR**	High-income	Both	Esophageal cancer	High alcohol use	Percent	1990	22.36%	28.62%	16.08%
**ASDR**	High-income	Male	Esophageal cancer	Diet low in vegetables	Percent	1990	18.05%	35.89%	-3.97%
**ASDR**	High-income	Female	Esophageal cancer	Diet low in vegetables	Percent	1990	18.85%	37.58%	-4.11%
**ASDR**	High-income	Both	Esophageal cancer	Diet low in vegetables	Percent	1990	18.17%	36.14%	-3.99%
**ASDR**	High-income	Male	Esophageal cancer	Chewing tobacco	Percent	1990	2.08%	3.07%	1.23%
**ASDR**	High-income	Female	Esophageal cancer	Chewing tobacco	Percent	1990	0.38%	0.63%	0.22%
**ASDR**	High-income	Both	Esophageal cancer	Chewing tobacco	Percent	1990	1.60%	2.32%	0.97%
**ASDR**	Latin America and Caribbean	Male	Esophageal cancer	Smoking	Percent	1990	43.98%	52.26%	35.27%

**ASDR**	Latin America and Caribbean	Female	Esophageal cancer	Smoking	Percent	1990	21.29%	27.15%	15.86%
**ASDR**	Latin America and Caribbean	Both	Esophageal cancer	Smoking	Percent	1990	37.47%	44.75%	29.72%
**ASDR**	Latin America and Caribbean	Male	Esophageal cancer	High alcohol use	Percent	1990	15.73%	20.49%	10.95%
**ASDR**	Latin America and Caribbean	Female	Esophageal cancer	High alcohol use	Percent	1990	2.97%	4.32%	1.82%
**ASDR**	Latin America and Caribbean	Both	Esophageal cancer	High alcohol use	Percent	1990	12.19%	16.02%	8.46%
**ASDR**	Latin America and Caribbean	Male	Esophageal cancer	Diet low in vegetables	Percent	1990	23.94%	46.46%	-5.43%
**ASDR**	Latin America and Caribbean	Female	Esophageal cancer	Diet low in vegetables	Percent	1990	23.80%	46.20%	-5.41%
**ASDR**	Latin America and Caribbean	Both	Esophageal cancer	Diet low in vegetables	Percent	1990	23.90%	46.38%	-5.43%
**ASDR**	Latin America and Caribbean	Male	Esophageal cancer	Chewing tobacco	Percent	1990	0.83%	1.31%	0.46%
**ASDR**	Latin America and Caribbean	Female	Esophageal cancer	Chewing tobacco	Percent	1990	0.74%	1.23%	0.40%
**ASDR**	Latin America and Caribbean	Both	Esophageal cancer	Chewing tobacco	Percent	1990	0.80%	1.15%	0.51%
**ASDR**	North Africa and Middle East	Male	Esophageal cancer	Smoking	Percent	1990	46.05%	55.04%	36.51%
**ASDR**	North Africa and Middle East	Female	Esophageal cancer	Smoking	Percent	1990	5.30%	6.93%	3.72%
**ASDR**	North Africa and Middle East	Both	Esophageal cancer	Smoking	Percent	1990	27.09%	34.01%	20.85%
**ASDR**	North Africa and Middle East	Male	Esophageal cancer	High alcohol use	Percent	1990	2.03%	2.89%	1.21%
**ASDR**	North Africa and Middle East	Female	Esophageal cancer	High alcohol use	Percent	1990	0.23%	0.33%	0.13%
**ASDR**	North Africa and Middle East	Both	Esophageal cancer	High alcohol use	Percent	1990	1.20%	1.75%	0.71%
**ASDR**	North Africa and Middle East	Male	Esophageal cancer	Diet low in vegetables	Percent	1990	16.45%	33.18%	-3.63%
**ASDR**	North Africa and Middle East	Female	Esophageal cancer	Diet low in vegetables	Percent	1990	16.94%	33.72%	-3.56%
**ASDR**	North Africa and Middle East	Both	Esophageal cancer	Diet low in vegetables	Percent	1990	16.71%	33.71%	-3.66%
**ASDR**	North Africa and Middle East	Male	Esophageal cancer	Chewing tobacco	Percent	1990	2.92%	4.40%	1.61%
**ASDR**	North Africa and Middle East	Female	Esophageal cancer	Chewing tobacco	Percent	1990	1.48%	2.32%	0.76%
**ASDR**	North Africa and Middle East	Both	Esophageal cancer	Chewing tobacco	Percent	1990	2.27%	3.12%	1.49%
**ASDR**	South Asia	Male	Esophageal cancer	Smoking	Percent	1990	35.74%	42.09%	29.06%
**ASDR**	South Asia	Female	Esophageal cancer	Smoking	Percent	1990	5.91%	7.67%	4.36%
**ASDR**	South Asia	Both	Esophageal cancer	Smoking	Percent	1990	22.55%	27.04%	17.69%
**ASDR**	South Asia	Male	Esophageal cancer	High alcohol use	Percent	1990	5.83%	8.86%	2.41%
**ASDR**	South Asia	Female	Esophageal cancer	High alcohol use	Percent	1990	0.40%	0.71%	0.15%
**ASDR**	South Asia	Both	Esophageal cancer	High alcohol use	Percent	1990	3.45%	5.35%	1.42%

**ASDR**	South Asia	Male	Esophageal cancer	Diet low in vegetables	Percent	1990	24.03%	46.59%	-5.52%
**ASDR**	South Asia	Female	Esophageal cancer	Diet low in vegetables	Percent	1990	24.29%	47.16%	-5.58%
**ASDR**	South Asia	Both	Esophageal cancer	Diet low in vegetables	Percent	1990	24.14%	46.83%	-5.55%
**ASDR**	South Asia	Male	Esophageal cancer	Chewing tobacco	Percent	1990	25.93%	34.28%	16.82%
**ASDR**	South Asia	Female	Esophageal cancer	Chewing tobacco	Percent	1990	22.47%	30.16%	14.30%
**ASDR**	South Asia	Both	Esophageal cancer	Chewing tobacco	Percent	1990	24.44%	30.18%	17.95%
**ASDR**	Sub-Saharan Africa	Male	Esophageal cancer	Smoking	Percent	1990	19.64%	23.54%	15.46%
**ASDR**	Sub-Saharan Africa	Female	Esophageal cancer	Smoking	Percent	1990	5.03%	6.42%	3.83%
**ASDR**	Sub-Saharan Africa	Both	Esophageal cancer	Smoking	Percent	1990	13.42%	16.25%	10.50%
**ASDR**	Sub-Saharan Africa	Male	Esophageal cancer	High alcohol use	Percent	1990	13.04%	17.43%	6.91%
**ASDR**	Sub-Saharan Africa	Female	Esophageal cancer	High alcohol use	Percent	1990	4.16%	5.79%	2.41%
**ASDR**	Sub-Saharan Africa	Both	Esophageal cancer	High alcohol use	Percent	1990	9.34%	12.57%	5.25%
**ASDR**	Sub-Saharan Africa	Male	Esophageal cancer	Diet low in vegetables	Percent	1990	25.48%	49.16%	-6.13%
**ASDR**	Sub-Saharan Africa	Female	Esophageal cancer	Diet low in vegetables	Percent	1990	25.40%	49.00%	-6.06%
**ASDR**	Sub-Saharan Africa	Both	Esophageal cancer	Diet low in vegetables	Percent	1990	25.45%	49.09%	-6.10%
**ASDR**	Sub-Saharan Africa	Male	Esophageal cancer	Chewing tobacco	Percent	1990	2.55%	3.84%	1.46%
**ASDR**	Sub-Saharan Africa	Female	Esophageal cancer	Chewing tobacco	Percent	1990	3.18%	4.76%	1.88%
**ASDR**	Sub-Saharan Africa	Both	Esophageal cancer	Chewing tobacco	Percent	1990	2.84%	3.82%	1.93%
**ASDR**	High-middle SDI	Male	Esophageal cancer	Smoking	Percent	1990	52.42%	60.43%	43.86%
**ASDR**	High-middle SDI	Female	Esophageal cancer	Smoking	Percent	1990	10.49%	13.41%	7.88%
**ASDR**	High-middle SDI	Both	Esophageal cancer	Smoking	Percent	1990	40.03%	46.78%	33.39%
**ASDR**	High-middle SDI	Male	Esophageal cancer	High alcohol use	Percent	1990	20.95%	26.30%	16.02%
**ASDR**	High-middle SDI	Female	Esophageal cancer	High alcohol use	Percent	1990	3.81%	5.26%	2.56%
**ASDR**	High-middle SDI	Both	Esophageal cancer	High alcohol use	Percent	1990	16.41%	21.19%	12.44%
**ASDR**	High-middle SDI	Male	Esophageal cancer	Diet low in vegetables	Percent	1990	19.70%	39.48%	-4.39%
**ASDR**	High-middle SDI	Female	Esophageal cancer	Diet low in vegetables	Percent	1990	19.49%	38.94%	-4.30%
**ASDR**	High-middle SDI	Both	Esophageal cancer	Diet low in vegetables	Percent	1990	19.63%	39.11%	-4.36%
**ASDR**	High-middle SDI	Male	Esophageal cancer	Chewing tobacco	Percent	1990	0.77%	1.20%	0.41%
**ASDR**	High-middle SDI	Female	Esophageal cancer	Chewing tobacco	Percent	1990	0.49%	0.80%	0.27%

**ASDR**	High-middle SDI	Both	Esophageal cancer	Chewing tobacco	Percent	1990	0.71%	1.04%	0.44%
**ASDR**	High SDI	Male	Esophageal cancer	Smoking	Percent	1990	57.48%	65.93%	47.61%
**ASDR**	High SDI	Female	Esophageal cancer	Smoking	Percent	1990	30.85%	38.18%	23.37%
**ASDR**	High SDI	Both	Esophageal cancer	Smoking	Percent	1990	50.49%	58.48%	41.23%
**ASDR**	High SDI	Male	Esophageal cancer	High alcohol use	Percent	1990	24.08%	30.87%	17.55%
**ASDR**	High SDI	Female	Esophageal cancer	High alcohol use	Percent	1990	11.05%	14.81%	7.42%
**ASDR**	High SDI	Both	Esophageal cancer	High alcohol use	Percent	1990	21.22%	27.12%	15.50%
**ASDR**	High SDI	Male	Esophageal cancer	Diet low in vegetables	Percent	1990	18.90%	37.51%	-4.15%
**ASDR**	High SDI	Female	Esophageal cancer	Diet low in vegetables	Percent	1990	19.35%	38.50%	-4.23%
**ASDR**	High SDI	Both	Esophageal cancer	Diet low in vegetables	Percent	1990	18.95%	37.69%	-4.15%
**ASDR**	High SDI	Male	Esophageal cancer	Chewing tobacco	Percent	1990	2.19%	3.21%	1.30%
**ASDR**	High SDI	Female	Esophageal cancer	Chewing tobacco	Percent	1990	0.42%	0.69%	0.24%
**ASDR**	High SDI	Both	Esophageal cancer	Chewing tobacco	Percent	1990	1.68%	2.42%	1.01%
**ASDR**	Low-middle SDI	Male	Esophageal cancer	Smoking	Percent	1990	37.95%	44.54%	31.10%
**ASDR**	Low-middle SDI	Female	Esophageal cancer	Smoking	Percent	1990	6.50%	8.25%	4.83%
**ASDR**	Low-middle SDI	Both	Esophageal cancer	Smoking	Percent	1990	24.39%	29.15%	19.50%
**ASDR**	Low-middle SDI	Male	Esophageal cancer	High alcohol use	Percent	1990	8.13%	11.15%	5.09%
**ASDR**	Low-middle SDI	Female	Esophageal cancer	High alcohol use	Percent	1990	1.07%	1.60%	0.62%
**ASDR**	Low-middle SDI	Both	Esophageal cancer	High alcohol use	Percent	1990	5.11%	7.06%	3.23%
**ASDR**	Low-middle SDI	Male	Esophageal cancer	Diet low in vegetables	Percent	1990	23.41%	45.47%	-5.46%
**ASDR**	Low-middle SDI	Female	Esophageal cancer	Diet low in vegetables	Percent	1990	23.63%	45.73%	-5.47%
**ASDR**	Low-middle SDI	Both	Esophageal cancer	Diet low in vegetables	Percent	1990	23.50%	45.57%	-5.46%
**ASDR**	Low-middle SDI	Male	Esophageal cancer	Chewing tobacco	Percent	1990	15.79%	21.42%	10.18%
**ASDR**	Low-middle SDI	Female	Esophageal cancer	Chewing tobacco	Percent	1990	16.05%	22.35%	10.38%
**ASDR**	Low-middle SDI	Both	Esophageal cancer	Chewing tobacco	Percent	1990	15.93%	19.80%	11.59%
**ASDR**	Low SDI	Male	Esophageal cancer	Smoking	Percent	1990	18.71%	22.34%	14.80%
**ASDR**	Low SDI	Female	Esophageal cancer	Smoking	Percent	1990	4.23%	5.58%	3.13%
**ASDR**	Low SDI	Both	Esophageal cancer	Smoking	Percent	1990	12.15%	14.65%	9.44%
**ASDR**	Low SDI	Male	Esophageal cancer	High alcohol use	Percent	1990	8.79%	12.03%	4.18%

**ASDR**	Low SDI	Female	Esophageal cancer	High alcohol use	Percent	1990	2.58%	3.78%	1.29%
**ASDR**	Low SDI	Both	Esophageal cancer	High alcohol use	Percent	1990	6.00%	8.29%	2.99%
**ASDR**	Low SDI	Male	Esophageal cancer	Diet low in vegetables	Percent	1990	25.81%	49.58%	-6.25%
**ASDR**	Low SDI	Female	Esophageal cancer	Diet low in vegetables	Percent	1990	25.70%	49.56%	-6.14%
**ASDR**	Low SDI	Both	Esophageal cancer	Diet low in vegetables	Percent	1990	25.77%	49.56%	-6.20%
**ASDR**	Low SDI	Male	Esophageal cancer	Chewing tobacco	Percent	1990	7.69%	10.74%	4.92%
**ASDR**	Low SDI	Female	Esophageal cancer	Chewing tobacco	Percent	1990	6.05%	9.45%	3.42%
**ASDR**	Low SDI	Both	Esophageal cancer	Chewing tobacco	Percent	1990	6.95%	9.08%	4.78%
**ASDR**	Middle SDI	Male	Esophageal cancer	Smoking	Percent	1990	52.43%	60.24%	44.11%
**ASDR**	Middle SDI	Female	Esophageal cancer	Smoking	Percent	1990	8.02%	10.44%	5.83%
**ASDR**	Middle SDI	Both	Esophageal cancer	Smoking	Percent	1990	37.47%	43.92%	30.85%
**ASDR**	Middle SDI	Male	Esophageal cancer	High alcohol use	Percent	1990	16.57%	21.40%	12.15%
**ASDR**	Middle SDI	Female	Esophageal cancer	High alcohol use	Percent	1990	1.72%	2.54%	1.07%
**ASDR**	Middle SDI	Both	Esophageal cancer	High alcohol use	Percent	1990	11.81%	15.67%	8.52%
**ASDR**	Middle SDI	Male	Esophageal cancer	Diet low in vegetables	Percent	1990	21.74%	43.05%	-4.91%
**ASDR**	Middle SDI	Female	Esophageal cancer	Diet low in vegetables	Percent	1990	21.51%	42.66%	-4.80%
**ASDR**	Middle SDI	Both	Esophageal cancer	Diet low in vegetables	Percent	1990	21.66%	42.85%	-4.87%
**ASDR**	Middle SDI	Male	Esophageal cancer	Chewing tobacco	Percent	1990	1.76%	2.54%	1.07%
**ASDR**	Middle SDI	Female	Esophageal cancer	Chewing tobacco	Percent	1990	1.83%	2.78%	1.10%
**ASDR**	Middle SDI	Both	Esophageal cancer	Chewing tobacco	Percent	1990	1.80%	2.38%	1.23%
**ASDALYR**	Global	Male	Esophageal cancer	Smoking	Percent	1990	48.99%	56.22%	41.19%
**ASDALYR**	Global	Female	Esophageal cancer	Smoking	Percent	1990	9.82%	12.39%	7.53%
**ASDALYR**	Global	Both	Esophageal cancer	Smoking	Percent	1990	37.59%	43.63%	31.45%
**ASDALYR**	Global	Male	Esophageal cancer	High alcohol use	Percent	1990	19.89%	25.19%	15.06%
**ASDALYR**	Global	Female	Esophageal cancer	High alcohol use	Percent	1990	3.51%	4.75%	2.38%
**ASDALYR**	Global	Both	Esophageal cancer	High alcohol use	Percent	1990	15.39%	19.80%	11.62%
**ASDALYR**	Global	Male	Esophageal cancer	Diet low in vegetables	Percent	1990	20.69%	40.90%	-4.59%
**ASDALYR**	Global	Female	Esophageal cancer	Diet low in vegetables	Percent	1990	21.12%	41.75%	-4.71%
**ASDALYR**	Global	Both	Esophageal cancer	Diet low in vegetables	Percent	1990	20.80%	41.10%	-4.62%

**ASDALYR**	Global	Male	Esophageal cancer	Chewing tobacco	Percent	1990	2.74%	3.82%	1.74%
**ASDALYR**	Global	Female	Esophageal cancer	Chewing tobacco	Percent	1990	3.27%	5.46%	1.90%
**ASDALYR**	Global	Both	Esophageal cancer	Chewing tobacco	Percent	1990	2.88%	3.76%	1.96%
**ASDALYR**	Southeast Asia, East Asia, and Oceania	Male	Esophageal cancer	Smoking	Percent	1990	51.65%	59.05%	43.53%
**ASDALYR**	Southeast Asia, East Asia, and Oceania	Female	Esophageal cancer	Smoking	Percent	1990	7.82%	10.22%	5.85%
**ASDALYR**	Southeast Asia, East Asia, and Oceania	Both	Esophageal cancer	Smoking	Percent	1990	39.19%	46.01%	32.29%
**ASDALYR**	Southeast Asia, East Asia, and Oceania	Male	Esophageal cancer	High alcohol use	Percent	1990	19.44%	24.84%	14.60%
**ASDALYR**	Southeast Asia, East Asia, and Oceania	Female	Esophageal cancer	High alcohol use	Percent	1990	1.92%	2.76%	1.16%
**ASDALYR**	Southeast Asia, East Asia, and Oceania	Both	Esophageal cancer	High alcohol use	Percent	1990	14.74%	19.30%	10.94%
**ASDALYR**	Southeast Asia, East Asia, and Oceania	Male	Esophageal cancer	Diet low in vegetables	Percent	1990	21.06%	41.73%	-4.68%
**ASDALYR**	Southeast Asia, East Asia, and Oceania	Female	Esophageal cancer	Diet low in vegetables	Percent	1990	20.99%	41.58%	-4.67%
**ASDALYR**	Southeast Asia, East Asia, and Oceania	Both	Esophageal cancer	Diet low in vegetables	Percent	1990	21.04%	41.70%	-4.67%
**ASDALYR**	Southeast Asia, East Asia, and Oceania	Male	Esophageal cancer	Chewing tobacco	Percent	1990	1.09%	1.68%	0.58%
**ASDALYR**	Southeast Asia, East Asia, and Oceania	Female	Esophageal cancer	Chewing tobacco	Percent	1990	0.93%	1.56%	0.54%
**ASDALYR**	Southeast Asia, East Asia, and Oceania	Both	Esophageal cancer	Chewing tobacco	Percent	1990	1.06%	1.52%	0.68%
**ASDALYR**	Central Europe, Eastern Europe, and Central Asia	Male	Esophageal cancer	Smoking	Percent	1990	49.67%	57.23%	41.64%
**ASDALYR**	Central Europe, Eastern Europe, and Central Asia	Female	Esophageal cancer	Smoking	Percent	1990	5.88%	7.33%	4.48%
**ASDALYR**	Central Europe, Eastern Europe, and Central Asia	Both	Esophageal cancer	Smoking	Percent	1990	37.62%	43.40%	31.45%
**ASDALYR**	Central Europe, Eastern Europe, and Central Asia	Male	Esophageal cancer	High alcohol use	Percent	1990	24.79%	31.17%	17.48%
**ASDALYR**	Central Europe, Eastern Europe, and Central Asia	Female	Esophageal cancer	High alcohol use	Percent	1990	6.34%	8.91%	4.28%
**ASDALYR**	Central Europe, Eastern Europe, and Central Asia	Both	Esophageal cancer	High alcohol use	Percent	1990	20.38%	25.72%	14.50%
**ASDALYR**	Central Europe, Eastern Europe, and Central Asia	Male	Esophageal cancer	Diet low in vegetables	Percent	1990	17.57%	35.45%	-3.86%
**ASDALYR**	Central Europe, Eastern Europe, and Central Asia	Female	Esophageal cancer	Diet low in vegetables	Percent	1990	16.65%	33.56%	-3.58%
**ASDALYR**	Central Europe, Eastern Europe, and Central Asia	Both	Esophageal cancer	Diet low in vegetables	Percent	1990	17.29%	34.87%	-3.78%
**ASDALYR**	Central Europe, Eastern Europe, and Central Asia	Male	Esophageal cancer	Chewing tobacco	Percent	1990	0.76%	1.15%	0.43%
**ASDALYR**	Central Europe, Eastern Europe, and Central Asia	Female	Esophageal cancer	Chewing tobacco	Percent	1990	0.36%	0.57%	0.20%
**ASDALYR**	Central Europe, Eastern Europe, and Central Asia	Both	Esophageal cancer	Chewing tobacco	Percent	1990	0.67%	0.98%	0.41%
**ASDALYR**	High-income	Male	Esophageal cancer	Smoking	Percent	1990	54.77%	63.08%	45.35%
**ASDALYR**	High-income	Female	Esophageal cancer	Smoking	Percent	1990	32.53%	39.58%	25.24%

**ASDALYR**	High-income	Both	Esophageal cancer	Smoking	Percent	1990	49.80%	57.66%	40.85%
**ASDALYR**	High-income	Male	Esophageal cancer	High alcohol use	Percent	1990	26.74%	33.75%	19.45%
**ASDALYR**	High-income	Female	Esophageal cancer	High alcohol use	Percent	1990	14.37%	18.83%	10.08%
**ASDALYR**	High-income	Both	Esophageal cancer	High alcohol use	Percent	1990	24.47%	30.95%	17.87%
**ASDALYR**	High-income	Male	Esophageal cancer	Diet low in vegetables	Percent	1990	17.71%	35.17%	-3.91%
**ASDALYR**	High-income	Female	Esophageal cancer	Diet low in vegetables	Percent	1990	18.43%	36.75%	-4.03%
**ASDALYR**	High-income	Both	Esophageal cancer	Diet low in vegetables	Percent	1990	17.80%	35.41%	-3.93%
**ASDALYR**	High-income	Male	Esophageal cancer	Chewing tobacco	Percent	1990	1.91%	2.87%	1.12%
**ASDALYR**	High-income	Female	Esophageal cancer	Chewing tobacco	Percent	1990	0.37%	0.60%	0.22%
**ASDALYR**	High-income	Both	Esophageal cancer	Chewing tobacco	Percent	1990	1.56%	2.28%	0.94%
**ASDALYR**	Latin America and Caribbean	Male	Esophageal cancer	Smoking	Percent	1990	42.53%	50.22%	34.19%
**ASDALYR**	Latin America and Caribbean	Female	Esophageal cancer	Smoking	Percent	1990	21.01%	26.52%	15.85%
**ASDALYR**	Latin America and Caribbean	Both	Esophageal cancer	Smoking	Percent	1990	36.95%	43.77%	29.61%
**ASDALYR**	Latin America and Caribbean	Male	Esophageal cancer	High alcohol use	Percent	1990	17.72%	22.90%	12.66%
**ASDALYR**	Latin America and Caribbean	Female	Esophageal cancer	High alcohol use	Percent	1990	3.68%	5.29%	2.34%
**ASDALYR**	Latin America and Caribbean	Both	Esophageal cancer	High alcohol use	Percent	1990	14.19%	18.48%	10.08%
**ASDALYR**	Latin America and Caribbean	Male	Esophageal cancer	Diet low in vegetables	Percent	1990	23.86%	46.30%	-5.41%
**ASDALYR**	Latin America and Caribbean	Female	Esophageal cancer	Diet low in vegetables	Percent	1990	23.65%	45.97%	-5.38%
**ASDALYR**	Latin America and Caribbean	Both	Esophageal cancer	Diet low in vegetables	Percent	1990	23.80%	46.21%	-5.40%
**ASDALYR**	Latin America and Caribbean	Male	Esophageal cancer	Chewing tobacco	Percent	1990	0.77%	1.23%	0.43%
**ASDALYR**	Latin America and Caribbean	Female	Esophageal cancer	Chewing tobacco	Percent	1990	0.67%	1.08%	0.36%
**ASDALYR**	Latin America and Caribbean	Both	Esophageal cancer	Chewing tobacco	Percent	1990	0.74%	1.06%	0.47%
**ASDALYR**	North Africa and Middle East	Male	Esophageal cancer	Smoking	Percent	1990	44.35%	52.48%	35.40%
**ASDALYR**	North Africa and Middle East	Female	Esophageal cancer	Smoking	Percent	1990	5.42%	6.97%	3.90%
**ASDALYR**	North Africa and Middle East	Both	Esophageal cancer	Smoking	Percent	1990	26.44%	33.28%	20.49%
**ASDALYR**	North Africa and Middle East	Male	Esophageal cancer	High alcohol use	Percent	1990	2.39%	3.40%	1.46%
**ASDALYR**	North Africa and Middle East	Female	Esophageal cancer	High alcohol use	Percent	1990	0.28%	0.42%	0.16%
**ASDALYR**	North Africa and Middle East	Both	Esophageal cancer	High alcohol use	Percent	1990	1.43%	2.08%	0.87%
**ASDALYR**	North Africa and Middle East	Male	Esophageal cancer	Diet low in vegetables	Percent	1990	16.11%	32.66%	-3.54%

**ASDALYR**	North Africa and Middle East	Female	Esophageal cancer	Diet low in vegetables	Percent	1990	16.86%	33.45%	-3.56%
**ASDALYR**	North Africa and Middle East	Both	Esophageal cancer	Diet low in vegetables	Percent	1990	16.49%	33.55%	-3.62%
**ASDALYR**	North Africa and Middle East	Male	Esophageal cancer	Chewing tobacco	Percent	1990	3.18%	4.81%	1.76%
**ASDALYR**	North Africa and Middle East	Female	Esophageal cancer	Chewing tobacco	Percent	1990	1.44%	2.27%	0.75%
**ASDALYR**	North Africa and Middle East	Both	Esophageal cancer	Chewing tobacco	Percent	1990	2.39%	3.34%	1.56%
**ASDALYR**	South Asia	Male	Esophageal cancer	Smoking	Percent	1990	33.63%	39.45%	27.36%
**ASDALYR**	South Asia	Female	Esophageal cancer	Smoking	Percent	1990	5.41%	6.98%	4.02%
**ASDALYR**	South Asia	Both	Esophageal cancer	Smoking	Percent	1990	21.26%	25.35%	16.67%
**ASDALYR**	South Asia	Male	Esophageal cancer	High alcohol use	Percent	1990	6.59%	9.97%	2.70%
**ASDALYR**	South Asia	Female	Esophageal cancer	High alcohol use	Percent	1990	0.48%	0.83%	0.18%
**ASDALYR**	South Asia	Both	Esophageal cancer	High alcohol use	Percent	1990	3.93%	6.08%	1.59%
**ASDALYR**	South Asia	Male	Esophageal cancer	Diet low in vegetables	Percent	1990	23.89%	46.37%	-5.48%
**ASDALYR**	South Asia	Female	Esophageal cancer	Diet low in vegetables	Percent	1990	24.07%	46.77%	-5.54%
**ASDALYR**	South Asia	Both	Esophageal cancer	Diet low in vegetables	Percent	1990	23.97%	46.54%	-5.51%
**ASDALYR**	South Asia	Male	Esophageal cancer	Chewing tobacco	Percent	1990	26.52%	34.89%	17.22%
**ASDALYR**	South Asia	Female	Esophageal cancer	Chewing tobacco	Percent	1990	21.21%	28.90%	13.47%
**ASDALYR**	South Asia	Both	Esophageal cancer	Chewing tobacco	Percent	1990	24.22%	30.13%	17.77%
**ASDALYR**	Sub-Saharan Africa	Male	Esophageal cancer	Smoking	Percent	1990	18.99%	22.67%	15.01%
**ASDALYR**	Sub-Saharan Africa	Female	Esophageal cancer	Smoking	Percent	1990	4.55%	5.78%	3.46%
**ASDALYR**	Sub-Saharan Africa	Both	Esophageal cancer	Smoking	Percent	1990	13.13%	15.97%	10.29%
**ASDALYR**	Sub-Saharan Africa	Male	Esophageal cancer	High alcohol use	Percent	1990	13.91%	18.34%	7.35%
**ASDALYR**	Sub-Saharan Africa	Female	Esophageal cancer	High alcohol use	Percent	1990	4.46%	6.27%	2.53%
**ASDALYR**	Sub-Saharan Africa	Both	Esophageal cancer	High alcohol use	Percent	1990	10.11%	13.56%	5.51%
**ASDALYR**	Sub-Saharan Africa	Male	Esophageal cancer	Diet low in vegetables	Percent	1990	25.44%	49.04%	-6.12%
**ASDALYR**	Sub-Saharan Africa	Female	Esophageal cancer	Diet low in vegetables	Percent	1990	25.34%	48.89%	-6.04%
**ASDALYR**	Sub-Saharan Africa	Both	Esophageal cancer	Diet low in vegetables	Percent	1990	25.40%	48.97%	-6.09%
**ASDALYR**	Sub-Saharan Africa	Male	Esophageal cancer	Chewing tobacco	Percent	1990	2.67%	4.04%	1.53%
**ASDALYR**	Sub-Saharan Africa	Female	Esophageal cancer	Chewing tobacco	Percent	1990	3.03%	4.57%	1.81%
**ASDALYR**	Sub-Saharan Africa	Both	Esophageal cancer	Chewing tobacco	Percent	1990	2.83%	3.78%	1.90%

**ASDALYR**	High-middle SDI	Male	Esophageal cancer	Smoking	Percent	1990	50.37%	57.82%	42.38%
**ASDALYR**	High-middle SDI	Female	Esophageal cancer	Smoking	Percent	1990	9.44%	12.08%	7.07%
**ASDALYR**	High-middle SDI	Both	Esophageal cancer	Smoking	Percent	1990	39.93%	46.33%	33.56%
**ASDALYR**	High-middle SDI	Male	Esophageal cancer	High alcohol use	Percent	1990	22.49%	28.25%	17.17%
**ASDALYR**	High-middle SDI	Female	Esophageal cancer	High alcohol use	Percent	1990	4.03%	5.56%	2.72%
**ASDALYR**	High-middle SDI	Both	Esophageal cancer	High alcohol use	Percent	1990	18.25%	23.37%	13.91%
**ASDALYR**	High-middle SDI	Male	Esophageal cancer	Diet low in vegetables	Percent	1990	19.53%	39.14%	-4.34%
**ASDALYR**	High-middle SDI	Female	Esophageal cancer	Diet low in vegetables	Percent	1990	19.34%	38.71%	-4.26%
**ASDALYR**	High-middle SDI	Both	Esophageal cancer	Diet low in vegetables	Percent	1990	19.46%	38.80%	-4.31%
**ASDALYR**	High-middle SDI	Male	Esophageal cancer	Chewing tobacco	Percent	1990	0.89%	1.40%	0.47%
**ASDALYR**	High-middle SDI	Female	Esophageal cancer	Chewing tobacco	Percent	1990	0.52%	0.85%	0.29%
**ASDALYR**	High-middle SDI	Both	Esophageal cancer	Chewing tobacco	Percent	1990	0.82%	1.23%	0.48%
**ASDALYR**	High SDI	Male	Esophageal cancer	Smoking	Percent	1990	55.36%	63.53%	46.07%
**ASDALYR**	High SDI	Female	Esophageal cancer	Smoking	Percent	1990	30.72%	37.45%	23.69%
**ASDALYR**	High SDI	Both	Esophageal cancer	Smoking	Percent	1990	49.89%	57.67%	41.10%
**ASDALYR**	High SDI	Male	Esophageal cancer	High alcohol use	Percent	1990	25.71%	32.42%	19.14%
**ASDALYR**	High SDI	Female	Esophageal cancer	High alcohol use	Percent	1990	12.25%	16.02%	8.56%
**ASDALYR**	High SDI	Both	Esophageal cancer	High alcohol use	Percent	1990	23.26%	29.41%	17.26%
**ASDALYR**	High SDI	Male	Esophageal cancer	Diet low in vegetables	Percent	1990	18.61%	36.95%	-4.09%
**ASDALYR**	High SDI	Female	Esophageal cancer	Diet low in vegetables	Percent	1990	18.93%	37.82%	-4.14%
**ASDALYR**	High SDI	Both	Esophageal cancer	Diet low in vegetables	Percent	1990	18.64%	37.05%	-4.09%
**ASDALYR**	High SDI	Male	Esophageal cancer	Chewing tobacco	Percent	1990	2.00%	3.01%	1.18%
**ASDALYR**	High SDI	Female	Esophageal cancer	Chewing tobacco	Percent	1990	0.41%	0.68%	0.24%
**ASDALYR**	High SDI	Both	Esophageal cancer	Chewing tobacco	Percent	1990	1.64%	2.40%	0.98%
**ASDALYR**	Low-middle SDI	Male	Esophageal cancer	Smoking	Percent	1990	36.03%	42.15%	29.61%
**ASDALYR**	Low-middle SDI	Female	Esophageal cancer	Smoking	Percent	1990	5.93%	7.46%	4.44%
**ASDALYR**	Low-middle SDI	Both	Esophageal cancer	Smoking	Percent	1990	23.33%	27.78%	18.61%
**ASDALYR**	Low-middle SDI	Male	Esophageal cancer	High alcohol use	Percent	1990	8.98%	12.33%	5.60%
**ASDALYR**	Low-middle SDI	Female	Esophageal cancer	High alcohol use	Percent	1990	1.16%	1.74%	0.65%

**ASDALYR**	Low-middle SDI	Both	Esophageal cancer	High alcohol use	Percent	1990	5.70%	7.87%	3.59%
**ASDALYR**	Low-middle SDI	Male	Esophageal cancer	Diet low in vegetables	Percent	1990	23.20%	45.12%	-5.41%
**ASDALYR**	Low-middle SDI	Female	Esophageal cancer	Diet low in vegetables	Percent	1990	23.47%	45.44%	-5.44%
**ASDALYR**	Low-middle SDI	Both	Esophageal cancer	Diet low in vegetables	Percent	1990	23.31%	45.23%	-5.42%
**ASDALYR**	Low-middle SDI	Male	Esophageal cancer	Chewing tobacco	Percent	1990	16.41%	22.22%	10.57%
**ASDALYR**	Low-middle SDI	Female	Esophageal cancer	Chewing tobacco	Percent	1990	15.90%	22.46%	10.17%
**ASDALYR**	Low-middle SDI	Both	Esophageal cancer	Chewing tobacco	Percent	1990	16.22%	20.33%	11.88%
**ASDALYR**	Low SDI	Male	Esophageal cancer	Smoking	Percent	1990	18.02%	21.41%	14.26%
**ASDALYR**	Low SDI	Female	Esophageal cancer	Smoking	Percent	1990	3.93%	5.11%	2.93%
**ASDALYR**	Low SDI	Both	Esophageal cancer	Smoking	Percent	1990	11.79%	14.19%	9.22%
**ASDALYR**	Low SDI	Male	Esophageal cancer	High alcohol use	Percent	1990	9.45%	12.88%	4.46%
**ASDALYR**	Low SDI	Female	Esophageal cancer	High alcohol use	Percent	1990	2.77%	4.11%	1.33%
**ASDALYR**	Low SDI	Both	Esophageal cancer	High alcohol use	Percent	1990	6.50%	9.03%	3.17%
**ASDALYR**	Low SDI	Male	Esophageal cancer	Diet low in vegetables	Percent	1990	25.75%	49.44%	-6.25%
**ASDALYR**	Low SDI	Female	Esophageal cancer	Diet low in vegetables	Percent	1990	25.59%	49.37%	-6.09%
**ASDALYR**	Low SDI	Both	Esophageal cancer	Diet low in vegetables	Percent	1990	25.69%	49.41%	-6.18%
**ASDALYR**	Low SDI	Male	Esophageal cancer	Chewing tobacco	Percent	1990	7.96%	11.13%	5.09%
**ASDALYR**	Low SDI	Female	Esophageal cancer	Chewing tobacco	Percent	1990	5.89%	9.57%	3.31%
**ASDALYR**	Low SDI	Both	Esophageal cancer	Chewing tobacco	Percent	1990	7.04%	9.32%	4.92%
**ASDALYR**	Middle SDI	Male	Esophageal cancer	Smoking	Percent	1990	50.27%	57.89%	42.43%
**ASDALYR**	Middle SDI	Female	Esophageal cancer	Smoking	Percent	1990	7.18%	9.35%	5.35%
**ASDALYR**	Middle SDI	Both	Esophageal cancer	Smoking	Percent	1990	37.14%	43.73%	30.61%
**ASDALYR**	Middle SDI	Male	Esophageal cancer	High alcohol use	Percent	1990	17.99%	23.09%	13.23%
**ASDALYR**	Middle SDI	Female	Esophageal cancer	High alcohol use	Percent	1990	1.87%	2.72%	1.18%
**ASDALYR**	Middle SDI	Both	Esophageal cancer	High alcohol use	Percent	1990	13.28%	17.50%	9.62%
**ASDALYR**	Middle SDI	Male	Esophageal cancer	Diet low in vegetables	Percent	1990	21.61%	42.77%	-4.84%
**ASDALYR**	Middle SDI	Female	Esophageal cancer	Diet low in vegetables	Percent	1990	21.40%	42.46%	-4.78%
**ASDALYR**	Middle SDI	Both	Esophageal cancer	Diet low in vegetables	Percent	1990	21.54%	42.62%	-4.82%
**ASDALYR**	Middle SDI	Male	Esophageal cancer	Chewing tobacco	Percent	1990	1.92%	2.78%	1.17%

**ASDALYR**	Middle SDI	Female	Esophageal cancer	Chewing tobacco	Percent	1990	1.82%	2.79%	1.06%
**ASDALYR**	Middle SDI	Both	Esophageal cancer	Chewing tobacco	Percent	1990	1.90%	2.58%	1.29%
**ASDR**	Global	Male	Esophageal cancer	Smoking	Percent	2021	48.58%	56.54%	39.96%
**ASDR**	Global	Female	Esophageal cancer	Smoking	Percent	2021	8.72%	11.36%	6.40%
**ASDR**	Global	Both	Esophageal cancer	Smoking	Percent	2021	37.99%	44.52%	30.79%
**ASDR**	Global	Male	Esophageal cancer	High alcohol use	Percent	2021	18.71%	23.69%	14.01%
**ASDR**	Global	Female	Esophageal cancer	High alcohol use	Percent	2021	3.48%	4.80%	2.33%
**ASDR**	Global	Both	Esophageal cancer	High alcohol use	Percent	2021	14.92%	19.14%	11.06%
**ASDR**	Global	Male	Esophageal cancer	Diet low in vegetables	Percent	2021	9.98%	20.62%	-2.28%
**ASDR**	Global	Female	Esophageal cancer	Diet low in vegetables	Percent	2021	12.72%	26.11%	-2.88%
**ASDR**	Global	Both	Esophageal cancer	Diet low in vegetables	Percent	2021	10.62%	21.71%	-2.37%
**ASDR**	Global	Male	Esophageal cancer	Chewing tobacco	Percent	2021	2.96%	4.21%	1.82%
**ASDR**	Global	Female	Esophageal cancer	Chewing tobacco	Percent	2021	4.27%	6.78%	2.53%
**ASDR**	Global	Both	Esophageal cancer	Chewing tobacco	Percent	2021	3.31%	4.42%	2.29%
**ASDR**	Southeast Asia, East Asia, and Oceania	Male	Esophageal cancer	Smoking	Percent	2021	57.08%	65.35%	48.08%
**ASDR**	Southeast Asia, East Asia, and Oceania	Female	Esophageal cancer	Smoking	Percent	2021	7.43%	10.53%	5.01%
**ASDR**	Southeast Asia, East Asia, and Oceania	Both	Esophageal cancer	Smoking	Percent	2021	45.39%	52.89%	37.30%
**ASDR**	Southeast Asia, East Asia, and Oceania	Male	Esophageal cancer	High alcohol use	Percent	2021	18.76%	23.91%	14.25%
**ASDR**	Southeast Asia, East Asia, and Oceania	Female	Esophageal cancer	High alcohol use	Percent	2021	1.75%	2.60%	1.03%
**ASDR**	Southeast Asia, East Asia, and Oceania	Both	Esophageal cancer	High alcohol use	Percent	2021	15.13%	19.82%	11.26%
**ASDR**	Southeast Asia, East Asia, and Oceania	Male	Esophageal cancer	Diet low in vegetables	Percent	2021	4.34%	10.75%	-0.87%
**ASDR**	Southeast Asia, East Asia, and Oceania	Female	Esophageal cancer	Diet low in vegetables	Percent	2021	4.65%	11.44%	-0.83%
**ASDR**	Southeast Asia, East Asia, and Oceania	Both	Esophageal cancer	Diet low in vegetables	Percent	2021	4.30%	10.01%	-0.83%
**ASDR**	Southeast Asia, East Asia, and Oceania	Male	Esophageal cancer	Chewing tobacco	Percent	2021	0.99%	1.50%	0.53%
**ASDR**	Southeast Asia, East Asia, and Oceania	Female	Esophageal cancer	Chewing tobacco	Percent	2021	1.22%	2.05%	0.68%
**ASDR**	Southeast Asia, East Asia, and Oceania	Both	Esophageal cancer	Chewing tobacco	Percent	2021	1.06%	1.49%	0.67%
**ASDR**	Central Europe, Eastern Europe, and Central Asia	Male	Esophageal cancer	Smoking	Percent	2021	47.19%	55.65%	38.50%
**ASDR**	Central Europe, Eastern Europe, and Central Asia	Female	Esophageal cancer	Smoking	Percent	2021	7.60%	9.46%	5.81%
**ASDR**	Central Europe, Eastern Europe, and Central Asia	Both	Esophageal cancer	Smoking	Percent	2021	37.38%	43.94%	30.53%

**ASDR**	Central Europe, Eastern Europe, and Central Asia	Male	Esophageal cancer	High alcohol use	Percent	2021	24.23%	31.29%	17.50%
**ASDR**	Central Europe, Eastern Europe, and Central Asia	Female	Esophageal cancer	High alcohol use	Percent	2021	5.82%	8.00%	3.67%
**ASDR**	Central Europe, Eastern Europe, and Central Asia	Both	Esophageal cancer	High alcohol use	Percent	2021	20.20%	25.97%	14.54%
**ASDR**	Central Europe, Eastern Europe, and Central Asia	Male	Esophageal cancer	Diet low in vegetables	Percent	2021	14.26%	29.29%	-2.92%
**ASDR**	Central Europe, Eastern Europe, and Central Asia	Female	Esophageal cancer	Diet low in vegetables	Percent	2021	12.15%	24.97%	-2.48%
**ASDR**	Central Europe, Eastern Europe, and Central Asia	Both	Esophageal cancer	Diet low in vegetables	Percent	2021	13.70%	28.03%	-2.79%
**ASDR**	Central Europe, Eastern Europe, and Central Asia	Male	Esophageal cancer	Chewing tobacco	Percent	2021	0.66%	1.01%	0.37%
**ASDR**	Central Europe, Eastern Europe, and Central Asia	Female	Esophageal cancer	Chewing tobacco	Percent	2021	0.39%	0.62%	0.23%
**ASDR**	Central Europe, Eastern Europe, and Central Asia	Both	Esophageal cancer	Chewing tobacco	Percent	2021	0.61%	0.89%	0.37%
**ASDR**	High-income	Male	Esophageal cancer	Smoking	Percent	2021	43.92%	53.61%	34.02%
**ASDR**	High-income	Female	Esophageal cancer	Smoking	Percent	2021	25.14%	31.46%	18.78%
**ASDR**	High-income	Both	Esophageal cancer	Smoking	Percent	2021	39.73%	48.37%	30.74%
**ASDR**	High-income	Male	Esophageal cancer	High alcohol use	Percent	2021	23.62%	30.05%	17.08%
**ASDR**	High-income	Female	Esophageal cancer	High alcohol use	Percent	2021	13.00%	17.31%	8.91%
**ASDR**	High-income	Both	Esophageal cancer	High alcohol use	Percent	2021	21.49%	27.49%	15.70%
**ASDR**	High-income	Male	Esophageal cancer	Diet low in vegetables	Percent	2021	17.38%	35.14%	-3.74%
**ASDR**	High-income	Female	Esophageal cancer	Diet low in vegetables	Percent	2021	18.10%	36.38%	-3.93%
**ASDR**	High-income	Both	Esophageal cancer	Diet low in vegetables	Percent	2021	17.51%	35.16%	-3.77%
**ASDR**	High-income	Male	Esophageal cancer	Chewing tobacco	Percent	2021	2.12%	3.29%	1.22%
**ASDR**	High-income	Female	Esophageal cancer	Chewing tobacco	Percent	2021	0.39%	0.63%	0.22%
**ASDR**	High-income	Both	Esophageal cancer	Chewing tobacco	Percent	2021	1.74%	2.65%	1.02%
**ASDR**	Latin America and Caribbean	Male	Esophageal cancer	Smoking	Percent	2021	27.05%	34.05%	20.36%
**ASDR**	Latin America and Caribbean	Female	Esophageal cancer	Smoking	Percent	2021	12.54%	16.50%	9.03%
**ASDR**	Latin America and Caribbean	Both	Esophageal cancer	Smoking	Percent	2021	23.34%	29.45%	17.78%
**ASDR**	Latin America and Caribbean	Male	Esophageal cancer	High alcohol use	Percent	2021	16.86%	21.90%	11.84%
**ASDR**	Latin America and Caribbean	Female	Esophageal cancer	High alcohol use	Percent	2021	3.69%	5.38%	2.36%
**ASDR**	Latin America and Caribbean	Both	Esophageal cancer	High alcohol use	Percent	2021	13.76%	18.03%	9.62%
**ASDR**	Latin America and Caribbean	Male	Esophageal cancer	Diet low in vegetables	Percent	2021	22.55%	44.32%	-5.04%
**ASDR**	Latin America and Caribbean	Female	Esophageal cancer	Diet low in vegetables	Percent	2021	22.58%	44.37%	-5.02%

**ASDR**	Latin America and Caribbean	Both	Esophageal cancer	Diet low in vegetables	Percent	2021	22.55%	44.30%	-5.02%
**ASDR**	Latin America and Caribbean	Male	Esophageal cancer	Chewing tobacco	Percent	2021	0.90%	1.42%	0.47%
**ASDR**	Latin America and Caribbean	Female	Esophageal cancer	Chewing tobacco	Percent	2021	0.77%	1.25%	0.42%
**ASDR**	Latin America and Caribbean	Both	Esophageal cancer	Chewing tobacco	Percent	2021	0.86%	1.23%	0.53%
**ASDR**	North Africa and Middle East	Male	Esophageal cancer	Smoking	Percent	2021	45.40%	54.96%	35.72%
**ASDR**	North Africa and Middle East	Female	Esophageal cancer	Smoking	Percent	2021	4.79%	6.38%	3.40%
**ASDR**	North Africa and Middle East	Both	Esophageal cancer	Smoking	Percent	2021	29.52%	35.86%	23.31%
**ASDR**	North Africa and Middle East	Male	Esophageal cancer	High alcohol use	Percent	2021	1.91%	2.74%	1.07%
**ASDR**	North Africa and Middle East	Female	Esophageal cancer	High alcohol use	Percent	2021	0.17%	0.28%	0.09%
**ASDR**	North Africa and Middle East	Both	Esophageal cancer	High alcohol use	Percent	2021	1.25%	1.84%	0.72%
**ASDR**	North Africa and Middle East	Male	Esophageal cancer	Diet low in vegetables	Percent	2021	11.63%	24.56%	-2.46%
**ASDR**	North Africa and Middle East	Female	Esophageal cancer	Diet low in vegetables	Percent	2021	12.32%	25.77%	-2.33%
**ASDR**	North Africa and Middle East	Both	Esophageal cancer	Diet low in vegetables	Percent	2021	11.91%	24.91%	-2.51%
**ASDR**	North Africa and Middle East	Male	Esophageal cancer	Chewing tobacco	Percent	2021	2.36%	3.56%	1.32%
**ASDR**	North Africa and Middle East	Female	Esophageal cancer	Chewing tobacco	Percent	2021	1.18%	1.92%	0.62%
**ASDR**	North Africa and Middle East	Both	Esophageal cancer	Chewing tobacco	Percent	2021	1.92%	2.67%	1.24%
**ASDR**	South Asia	Male	Esophageal cancer	Smoking	Percent	2021	26.52%	31.88%	21.19%
**ASDR**	South Asia	Female	Esophageal cancer	Smoking	Percent	2021	3.92%	5.29%	2.81%
**ASDR**	South Asia	Both	Esophageal cancer	Smoking	Percent	2021	16.91%	20.71%	13.27%
**ASDR**	South Asia	Male	Esophageal cancer	High alcohol use	Percent	2021	9.31%	12.95%	5.46%
**ASDR**	South Asia	Female	Esophageal cancer	High alcohol use	Percent	2021	0.63%	0.96%	0.35%
**ASDR**	South Asia	Both	Esophageal cancer	High alcohol use	Percent	2021	5.70%	8.03%	3.36%
**ASDR**	South Asia	Male	Esophageal cancer	Diet low in vegetables	Percent	2021	22.76%	44.70%	-5.08%
**ASDR**	South Asia	Female	Esophageal cancer	Diet low in vegetables	Percent	2021	23.15%	45.20%	-5.15%
**ASDR**	South Asia	Both	Esophageal cancer	Diet low in vegetables	Percent	2021	22.92%	44.87%	-5.11%
**ASDR**	South Asia	Male	Esophageal cancer	Chewing tobacco	Percent	2021	24.25%	32.91%	16.17%
**ASDR**	South Asia	Female	Esophageal cancer	Chewing tobacco	Percent	2021	20.79%	28.29%	13.60%
**ASDR**	South Asia	Both	Esophageal cancer	Chewing tobacco	Percent	2021	22.85%	28.88%	16.71%
**ASDR**	Sub-Saharan Africa	Male	Esophageal cancer	Smoking	Percent	2021	14.39%	17.61%	11.28%

**ASDR**	Sub-Saharan Africa	Female	Esophageal cancer	Smoking	Percent	2021	3.37%	4.37%	2.54%
**ASDR**	Sub-Saharan Africa	Both	Esophageal cancer	Smoking	Percent	2021	9.72%	11.93%	7.56%
**ASDR**	Sub-Saharan Africa	Male	Esophageal cancer	High alcohol use	Percent	2021	15.01%	19.56%	10.38%
**ASDR**	Sub-Saharan Africa	Female	Esophageal cancer	High alcohol use	Percent	2021	4.27%	5.85%	2.80%
**ASDR**	Sub-Saharan Africa	Both	Esophageal cancer	High alcohol use	Percent	2021	10.48%	13.88%	7.28%
**ASDR**	Sub-Saharan Africa	Male	Esophageal cancer	Diet low in vegetables	Percent	2021	24.47%	47.33%	-5.62%
**ASDR**	Sub-Saharan Africa	Female	Esophageal cancer	Diet low in vegetables	Percent	2021	24.61%	47.64%	-5.64%
**ASDR**	Sub-Saharan Africa	Both	Esophageal cancer	Diet low in vegetables	Percent	2021	24.53%	47.46%	-5.63%
**ASDR**	Sub-Saharan Africa	Male	Esophageal cancer	Chewing tobacco	Percent	2021	2.16%	3.22%	1.24%
**ASDR**	Sub-Saharan Africa	Female	Esophageal cancer	Chewing tobacco	Percent	2021	3.13%	4.89%	1.74%
**ASDR**	Sub-Saharan Africa	Both	Esophageal cancer	Chewing tobacco	Percent	2021	2.59%	3.48%	1.75%
**ASDR**	High-middle SDI	Male	Esophageal cancer	Smoking	Percent	2021	55.72%	63.82%	46.65%
**ASDR**	High-middle SDI	Female	Esophageal cancer	Smoking	Percent	2021	9.50%	13.14%	6.49%
**ASDR**	High-middle SDI	Both	Esophageal cancer	Smoking	Percent	2021	45.43%	53.26%	37.41%
**ASDR**	High-middle SDI	Male	Esophageal cancer	High alcohol use	Percent	2021	20.72%	26.39%	15.61%
**ASDR**	High-middle SDI	Female	Esophageal cancer	High alcohol use	Percent	2021	3.10%	4.47%	1.91%
**ASDR**	High-middle SDI	Both	Esophageal cancer	High alcohol use	Percent	2021	17.24%	22.39%	12.96%
**ASDR**	High-middle SDI	Male	Esophageal cancer	Diet low in vegetables	Percent	2021	4.19%	9.70%	-0.89%
**ASDR**	High-middle SDI	Female	Esophageal cancer	Diet low in vegetables	Percent	2021	4.56%	11.37%	-0.95%
**ASDR**	High-middle SDI	Both	Esophageal cancer	Diet low in vegetables	Percent	2021	4.18%	9.29%	-0.86%
**ASDR**	High-middle SDI	Male	Esophageal cancer	Chewing tobacco	Percent	2021	0.88%	1.38%	0.48%
**ASDR**	High-middle SDI	Female	Esophageal cancer	Chewing tobacco	Percent	2021	0.63%	1.08%	0.33%
**ASDR**	High-middle SDI	Both	Esophageal cancer	Chewing tobacco	Percent	2021	0.85%	1.24%	0.49%
**ASDR**	High SDI	Male	Esophageal cancer	Smoking	Percent	2021	45.51%	54.94%	35.99%
**ASDR**	High SDI	Female	Esophageal cancer	Smoking	Percent	2021	24.13%	30.47%	17.80%
**ASDR**	High SDI	Both	Esophageal cancer	Smoking	Percent	2021	40.93%	49.49%	32.17%
**ASDR**	High SDI	Male	Esophageal cancer	High alcohol use	Percent	2021	23.07%	29.23%	16.95%
**ASDR**	High SDI	Female	Esophageal cancer	High alcohol use	Percent	2021	11.76%	15.55%	8.02%
**ASDR**	High SDI	Both	Esophageal cancer	High alcohol use	Percent	2021	20.93%	26.73%	15.43%

**ASDR**	High SDI	Male	Esophageal cancer	Diet low in vegetables	Percent	2021	15.68%	32.01%	-3.39%
**ASDR**	High SDI	Female	Esophageal cancer	Diet low in vegetables	Percent	2021	16.52%	33.46%	-3.62%
**ASDR**	High SDI	Both	Esophageal cancer	Diet low in vegetables	Percent	2021	15.81%	32.05%	-3.42%
**ASDR**	High SDI	Male	Esophageal cancer	Chewing tobacco	Percent	2021	2.07%	3.23%	1.18%
**ASDR**	High SDI	Female	Esophageal cancer	Chewing tobacco	Percent	2021	0.44%	0.70%	0.25%
**ASDR**	High SDI	Both	Esophageal cancer	Chewing tobacco	Percent	2021	1.73%	2.63%	1.02%
**ASDR**	Low-middle SDI	Male	Esophageal cancer	Smoking	Percent	2021	29.47%	35.25%	23.55%
**ASDR**	Low-middle SDI	Female	Esophageal cancer	Smoking	Percent	2021	4.40%	5.81%	3.21%
**ASDR**	Low-middle SDI	Both	Esophageal cancer	Smoking	Percent	2021	19.21%	23.30%	15.10%
**ASDR**	Low-middle SDI	Male	Esophageal cancer	High alcohol use	Percent	2021	11.16%	14.93%	7.63%
**ASDR**	Low-middle SDI	Female	Esophageal cancer	High alcohol use	Percent	2021	1.53%	2.26%	0.91%
**ASDR**	Low-middle SDI	Both	Esophageal cancer	High alcohol use	Percent	2021	7.31%	9.86%	5.02%
**ASDR**	Low-middle SDI	Male	Esophageal cancer	Diet low in vegetables	Percent	2021	22.45%	44.19%	-5.05%
**ASDR**	Low-middle SDI	Female	Esophageal cancer	Diet low in vegetables	Percent	2021	22.82%	44.62%	-5.11%
**ASDR**	Low-middle SDI	Both	Esophageal cancer	Diet low in vegetables	Percent	2021	22.60%	44.36%	-5.07%
**ASDR**	Low-middle SDI	Male	Esophageal cancer	Chewing tobacco	Percent	2021	14.42%	19.89%	9.31%
**ASDR**	Low-middle SDI	Female	Esophageal cancer	Chewing tobacco	Percent	2021	15.08%	21.31%	9.56%
**ASDR**	Low-middle SDI	Both	Esophageal cancer	Chewing tobacco	Percent	2021	14.72%	18.82%	10.53%
**ASDR**	Low SDI	Male	Esophageal cancer	Smoking	Percent	2021	15.65%	19.12%	12.17%
**ASDR**	Low SDI	Female	Esophageal cancer	Smoking	Percent	2021	3.42%	4.64%	2.52%
**ASDR**	Low SDI	Both	Esophageal cancer	Smoking	Percent	2021	10.11%	12.38%	7.84%
**ASDR**	Low SDI	Male	Esophageal cancer	High alcohol use	Percent	2021	10.69%	13.95%	7.07%
**ASDR**	Low SDI	Female	Esophageal cancer	High alcohol use	Percent	2021	2.98%	4.24%	1.81%
**ASDR**	Low SDI	Both	Esophageal cancer	High alcohol use	Percent	2021	7.24%	9.70%	4.84%
**ASDR**	Low SDI	Male	Esophageal cancer	Diet low in vegetables	Percent	2021	25.05%	48.21%	-5.87%
**ASDR**	Low SDI	Female	Esophageal cancer	Diet low in vegetables	Percent	2021	25.13%	48.46%	-5.77%
**ASDR**	Low SDI	Both	Esophageal cancer	Diet low in vegetables	Percent	2021	25.09%	48.32%	-5.82%
**ASDR**	Low SDI	Male	Esophageal cancer	Chewing tobacco	Percent	2021	7.47%	10.44%	4.76%
**ASDR**	Low SDI	Female	Esophageal cancer	Chewing tobacco	Percent	2021	5.89%	9.71%	3.48%

**ASDR**	Low SDI	Both	Esophageal cancer	Chewing tobacco	Percent	2021	6.75%	8.88%	4.80%
**ASDR**	Middle SDI	Male	Esophageal cancer	Smoking	Percent	2021	51.41%	59.34%	42.70%
**ASDR**	Middle SDI	Female	Esophageal cancer	Smoking	Percent	2021	5.92%	8.11%	4.14%
**ASDR**	Middle SDI	Both	Esophageal cancer	Smoking	Percent	2021	39.63%	46.67%	32.11%
**ASDR**	Middle SDI	Male	Esophageal cancer	High alcohol use	Percent	2021	17.37%	22.36%	13.04%
**ASDR**	Middle SDI	Female	Esophageal cancer	High alcohol use	Percent	2021	1.79%	2.62%	1.12%
**ASDR**	Middle SDI	Both	Esophageal cancer	High alcohol use	Percent	2021	13.60%	17.86%	10.04%
**ASDR**	Middle SDI	Male	Esophageal cancer	Diet low in vegetables	Percent	2021	8.14%	17.38%	-1.92%
**ASDR**	Middle SDI	Female	Esophageal cancer	Diet low in vegetables	Percent	2021	9.42%	20.80%	-2.13%
**ASDR**	Middle SDI	Both	Esophageal cancer	Diet low in vegetables	Percent	2021	8.39%	18.04%	-1.91%
**ASDR**	Middle SDI	Male	Esophageal cancer	Chewing tobacco	Percent	2021	2.26%	3.26%	1.39%
**ASDR**	Middle SDI	Female	Esophageal cancer	Chewing tobacco	Percent	2021	3.23%	4.78%	1.95%
**ASDR**	Middle SDI	Both	Esophageal cancer	Chewing tobacco	Percent	2021	2.52%	3.35%	1.73%
**ASDALYR**	Global	Male	Esophageal cancer	Smoking	Percent	2021	45.91%	53.25%	37.91%
**ASDALYR**	Global	Female	Esophageal cancer	Smoking	Percent	2021	7.66%	9.86%	5.73%
**ASDALYR**	Global	Both	Esophageal cancer	Smoking	Percent	2021	36.45%	42.70%	29.49%
**ASDALYR**	Global	Male	Esophageal cancer	High alcohol use	Percent	2021	19.99%	25.27%	15.16%
**ASDALYR**	Global	Female	Esophageal cancer	High alcohol use	Percent	2021	3.59%	4.91%	2.43%
**ASDALYR**	Global	Both	Esophageal cancer	High alcohol use	Percent	2021	16.15%	20.59%	12.09%
**ASDALYR**	Global	Male	Esophageal cancer	Diet low in vegetables	Percent	2021	9.99%	20.58%	-2.23%
**ASDALYR**	Global	Female	Esophageal cancer	Diet low in vegetables	Percent	2021	13.44%	27.29%	-3.05%
**ASDALYR**	Global	Both	Esophageal cancer	Diet low in vegetables	Percent	2021	10.78%	21.95%	-2.38%
**ASDALYR**	Global	Male	Esophageal cancer	Chewing tobacco	Percent	2021	3.33%	4.72%	2.00%
**ASDALYR**	Global	Female	Esophageal cancer	Chewing tobacco	Percent	2021	4.75%	7.41%	2.86%
**ASDALYR**	Global	Both	Esophageal cancer	Chewing tobacco	Percent	2021	3.68%	4.91%	2.52%
**ASDALYR**	Southeast Asia, East Asia, and Oceania	Male	Esophageal cancer	Smoking	Percent	2021	54.39%	62.44%	45.68%
**ASDALYR**	Southeast Asia, East Asia, and Oceania	Female	Esophageal cancer	Smoking	Percent	2021	6.58%	9.11%	4.51%
**ASDALYR**	Southeast Asia, East Asia, and Oceania	Both	Esophageal cancer	Smoking	Percent	2021	44.64%	51.70%	37.03%
**ASDALYR**	Southeast Asia, East Asia, and Oceania	Male	Esophageal cancer	High alcohol use	Percent	2021	20.55%	26.15%	15.70%

**ASDALYR**	Southeast Asia, East Asia, and Oceania	Female	Esophageal cancer	High alcohol use	Percent	2021	1.89%	2.78%	1.14%
**ASDALYR**	Southeast Asia, East Asia, and Oceania	Both	Esophageal cancer	High alcohol use	Percent	2021	17.06%	22.01%	12.96%
**ASDALYR**	Southeast Asia, East Asia, and Oceania	Male	Esophageal cancer	Diet low in vegetables	Percent	2021	3.99%	9.32%	-0.78%
**ASDALYR**	Southeast Asia, East Asia, and Oceania	Female	Esophageal cancer	Diet low in vegetables	Percent	2021	4.51%	10.81%	-0.83%
**ASDALYR**	Southeast Asia, East Asia, and Oceania	Both	Esophageal cancer	Diet low in vegetables	Percent	2021	4.03%	9.40%	-0.76%
**ASDALYR**	Southeast Asia, East Asia, and Oceania	Male	Esophageal cancer	Chewing tobacco	Percent	2021	1.16%	1.74%	0.64%
**ASDALYR**	Southeast Asia, East Asia, and Oceania	Female	Esophageal cancer	Chewing tobacco	Percent	2021	1.25%	2.10%	0.67%
**ASDALYR**	Southeast Asia, East Asia, and Oceania	Both	Esophageal cancer	Chewing tobacco	Percent	2021	1.20%	1.70%	0.74%
**ASDALYR**	Central Europe, Eastern Europe, and Central Asia	Male	Esophageal cancer	Smoking	Percent	2021	46.21%	54.06%	38.03%
**ASDALYR**	Central Europe, Eastern Europe, and Central Asia	Female	Esophageal cancer	Smoking	Percent	2021	8.12%	10.01%	6.29%
**ASDALYR**	Central Europe, Eastern Europe, and Central Asia	Both	Esophageal cancer	Smoking	Percent	2021	37.75%	44.08%	31.11%
**ASDALYR**	Central Europe, Eastern Europe, and Central Asia	Male	Esophageal cancer	High alcohol use	Percent	2021	25.86%	32.77%	18.83%
**ASDALYR**	Central Europe, Eastern Europe, and Central Asia	Female	Esophageal cancer	High alcohol use	Percent	2021	6.81%	9.19%	4.41%
**ASDALYR**	Central Europe, Eastern Europe, and Central Asia	Both	Esophageal cancer	High alcohol use	Percent	2021	22.09%	27.95%	16.14%
**ASDALYR**	Central Europe, Eastern Europe, and Central Asia	Male	Esophageal cancer	Diet low in vegetables	Percent	2021	13.91%	28.56%	-2.82%
**ASDALYR**	Central Europe, Eastern Europe, and Central Asia	Female	Esophageal cancer	Diet low in vegetables	Percent	2021	11.20%	23.35%	-2.29%
**ASDALYR**	Central Europe, Eastern Europe, and Central Asia	Both	Esophageal cancer	Diet low in vegetables	Percent	2021	13.30%	27.24%	-2.69%
**ASDALYR**	Central Europe, Eastern Europe, and Central Asia	Male	Esophageal cancer	Chewing tobacco	Percent	2021	0.71%	1.08%	0.39%
**ASDALYR**	Central Europe, Eastern Europe, and Central Asia	Female	Esophageal cancer	Chewing tobacco	Percent	2021	0.40%	0.64%	0.23%
**ASDALYR**	Central Europe, Eastern Europe, and Central Asia	Both	Esophageal cancer	Chewing tobacco	Percent	2021	0.65%	0.96%	0.39%
**ASDALYR**	High-income	Male	Esophageal cancer	Smoking	Percent	2021	42.27%	51.26%	33.36%
**ASDALYR**	High-income	Female	Esophageal cancer	Smoking	Percent	2021	25.31%	31.39%	19.23%
**ASDALYR**	High-income	Both	Esophageal cancer	Smoking	Percent	2021	38.79%	47.01%	30.48%
**ASDALYR**	High-income	Male	Esophageal cancer	High alcohol use	Percent	2021	25.10%	31.73%	18.37%
**ASDALYR**	High-income	Female	Esophageal cancer	High alcohol use	Percent	2021	14.44%	19.15%	10.14%
**ASDALYR**	High-income	Both	Esophageal cancer	High alcohol use	Percent	2021	23.15%	29.25%	16.97%
**ASDALYR**	High-income	Male	Esophageal cancer	Diet low in vegetables	Percent	2021	17.30%	35.05%	-3.70%
**ASDALYR**	High-income	Female	Esophageal cancer	Diet low in vegetables	Percent	2021	17.79%	35.63%	-3.86%
**ASDALYR**	High-income	Both	Esophageal cancer	Diet low in vegetables	Percent	2021	17.39%	35.06%	-3.73%

**ASDALYR**	High-income	Male	Esophageal cancer	Chewing tobacco	Percent	2021	2.16%	3.33%	1.22%
**ASDALYR**	High-income	Female	Esophageal cancer	Chewing tobacco	Percent	2021	0.40%	0.65%	0.23%
**ASDALYR**	High-income	Both	Esophageal cancer	Chewing tobacco	Percent	2021	1.82%	2.75%	1.06%
**ASDALYR**	Latin America and Caribbean	Male	Esophageal cancer	Smoking	Percent	2021	25.27%	31.60%	19.24%
**ASDALYR**	Latin America and Caribbean	Female	Esophageal cancer	Smoking	Percent	2021	12.58%	16.31%	9.34%
**ASDALYR**	Latin America and Caribbean	Both	Esophageal cancer	Smoking	Percent	2021	22.34%	27.96%	17.05%
**ASDALYR**	Latin America and Caribbean	Male	Esophageal cancer	High alcohol use	Percent	2021	18.76%	24.01%	13.42%
**ASDALYR**	Latin America and Caribbean	Female	Esophageal cancer	High alcohol use	Percent	2021	4.50%	6.47%	2.92%
**ASDALYR**	Latin America and Caribbean	Both	Esophageal cancer	High alcohol use	Percent	2021	15.72%	20.25%	11.15%
**ASDALYR**	Latin America and Caribbean	Male	Esophageal cancer	Diet low in vegetables	Percent	2021	22.41%	44.09%	-5.01%
**ASDALYR**	Latin America and Caribbean	Female	Esophageal cancer	Diet low in vegetables	Percent	2021	22.40%	44.05%	-4.99%
**ASDALYR**	Latin America and Caribbean	Both	Esophageal cancer	Diet low in vegetables	Percent	2021	22.40%	44.08%	-4.99%
**ASDALYR**	Latin America and Caribbean	Male	Esophageal cancer	Chewing tobacco	Percent	2021	0.83%	1.27%	0.45%
**ASDALYR**	Latin America and Caribbean	Female	Esophageal cancer	Chewing tobacco	Percent	2021	0.69%	1.08%	0.39%
**ASDALYR**	Latin America and Caribbean	Both	Esophageal cancer	Chewing tobacco	Percent	2021	0.79%	1.15%	0.49%
**ASDALYR**	North Africa and Middle East	Male	Esophageal cancer	Smoking	Percent	2021	43.51%	52.09%	34.57%
**ASDALYR**	North Africa and Middle East	Female	Esophageal cancer	Smoking	Percent	2021	4.87%	6.35%	3.47%
**ASDALYR**	North Africa and Middle East	Both	Esophageal cancer	Smoking	Percent	2021	28.41%	34.56%	22.57%
**ASDALYR**	North Africa and Middle East	Male	Esophageal cancer	High alcohol use	Percent	2021	2.27%	3.23%	1.30%
**ASDALYR**	North Africa and Middle East	Female	Esophageal cancer	High alcohol use	Percent	2021	0.19%	0.31%	0.10%
**ASDALYR**	North Africa and Middle East	Both	Esophageal cancer	High alcohol use	Percent	2021	1.48%	2.18%	0.87%
**ASDALYR**	North Africa and Middle East	Male	Esophageal cancer	Diet low in vegetables	Percent	2021	11.31%	23.91%	-2.41%
**ASDALYR**	North Africa and Middle East	Female	Esophageal cancer	Diet low in vegetables	Percent	2021	12.64%	26.35%	-2.44%
**ASDALYR**	North Africa and Middle East	Both	Esophageal cancer	Diet low in vegetables	Percent	2021	11.85%	24.69%	-2.53%
**ASDALYR**	North Africa and Middle East	Male	Esophageal cancer	Chewing tobacco	Percent	2021	2.70%	3.97%	1.52%
**ASDALYR**	North Africa and Middle East	Female	Esophageal cancer	Chewing tobacco	Percent	2021	1.19%	1.96%	0.62%
**ASDALYR**	North Africa and Middle East	Both	Esophageal cancer	Chewing tobacco	Percent	2021	2.13%	2.93%	1.36%
**ASDALYR**	South Asia	Male	Esophageal cancer	Smoking	Percent	2021	24.75%	29.53%	19.82%
**ASDALYR**	South Asia	Female	Esophageal cancer	Smoking	Percent	2021	3.44%	4.62%	2.44%

**ASDALYR**	South Asia	Both	Esophageal cancer	Smoking	Percent	2021	15.81%	19.26%	12.37%
**ASDALYR**	South Asia	Male	Esophageal cancer	High alcohol use	Percent	2021	10.37%	14.26%	6.21%
**ASDALYR**	South Asia	Female	Esophageal cancer	High alcohol use	Percent	2021	0.75%	1.14%	0.42%
**ASDALYR**	South Asia	Both	Esophageal cancer	High alcohol use	Percent	2021	6.40%	8.92%	3.84%
**ASDALYR**	South Asia	Male	Esophageal cancer	Diet low in vegetables	Percent	2021	22.65%	44.53%	-5.05%
**ASDALYR**	South Asia	Female	Esophageal cancer	Diet low in vegetables	Percent	2021	22.99%	44.93%	-5.11%
**ASDALYR**	South Asia	Both	Esophageal cancer	Diet low in vegetables	Percent	2021	22.79%	44.64%	-5.08%
**ASDALYR**	South Asia	Male	Esophageal cancer	Chewing tobacco	Percent	2021	24.53%	33.16%	16.14%
**ASDALYR**	South Asia	Female	Esophageal cancer	Chewing tobacco	Percent	2021	19.31%	26.73%	12.44%
**ASDALYR**	South Asia	Both	Esophageal cancer	Chewing tobacco	Percent	2021	22.40%	28.34%	16.24%
**ASDALYR**	Sub-Saharan Africa	Male	Esophageal cancer	Smoking	Percent	2021	14.61%	17.81%	11.57%
**ASDALYR**	Sub-Saharan Africa	Female	Esophageal cancer	Smoking	Percent	2021	3.18%	4.05%	2.42%
**ASDALYR**	Sub-Saharan Africa	Both	Esophageal cancer	Smoking	Percent	2021	10.10%	12.32%	7.90%
**ASDALYR**	Sub-Saharan Africa	Male	Esophageal cancer	High alcohol use	Percent	2021	15.93%	20.76%	10.83%
**ASDALYR**	Sub-Saharan Africa	Female	Esophageal cancer	High alcohol use	Percent	2021	4.61%	6.34%	2.94%
**ASDALYR**	Sub-Saharan Africa	Both	Esophageal cancer	High alcohol use	Percent	2021	11.48%	15.14%	7.87%
**ASDALYR**	Sub-Saharan Africa	Male	Esophageal cancer	Diet low in vegetables	Percent	2021	24.42%	47.21%	-5.61%
**ASDALYR**	Sub-Saharan Africa	Female	Esophageal cancer	Diet low in vegetables	Percent	2021	24.54%	47.48%	-5.62%
**ASDALYR**	Sub-Saharan Africa	Both	Esophageal cancer	Diet low in vegetables	Percent	2021	24.47%	47.31%	-5.61%
**ASDALYR**	Sub-Saharan Africa	Male	Esophageal cancer	Chewing tobacco	Percent	2021	2.26%	3.34%	1.30%
**ASDALYR**	Sub-Saharan Africa	Female	Esophageal cancer	Chewing tobacco	Percent	2021	2.97%	4.60%	1.71%
**ASDALYR**	Sub-Saharan Africa	Both	Esophageal cancer	Chewing tobacco	Percent	2021	2.55%	3.41%	1.74%
**ASDALYR**	High-middle SDI	Male	Esophageal cancer	Smoking	Percent	2021	53.28%	61.17%	44.71%
**ASDALYR**	High-middle SDI	Female	Esophageal cancer	Smoking	Percent	2021	8.79%	11.90%	6.28%
**ASDALYR**	High-middle SDI	Both	Esophageal cancer	Smoking	Percent	2021	44.85%	51.94%	37.17%
**ASDALYR**	High-middle SDI	Male	Esophageal cancer	High alcohol use	Percent	2021	22.48%	28.36%	17.19%
**ASDALYR**	High-middle SDI	Female	Esophageal cancer	High alcohol use	Percent	2021	3.46%	4.90%	2.17%
**ASDALYR**	High-middle SDI	Both	Esophageal cancer	High alcohol use	Percent	2021	19.27%	24.55%	14.55%
**ASDALYR**	High-middle SDI	Male	Esophageal cancer	Diet low in vegetables	Percent	2021	3.91%	8.75%	-0.81%

**ASDALYR**	High-middle SDI	Female	Esophageal cancer	Diet low in vegetables	Percent	2021	4.35%	10.32%	-0.91%
**ASDALYR**	High-middle SDI	Both	Esophageal cancer	Diet low in vegetables	Percent	2021	3.94%	8.74%	-0.80%
**ASDALYR**	High-middle SDI	Male	Esophageal cancer	Chewing tobacco	Percent	2021	1.03%	1.61%	0.55%
**ASDALYR**	High-middle SDI	Female	Esophageal cancer	Chewing tobacco	Percent	2021	0.68%	1.13%	0.35%
**ASDALYR**	High-middle SDI	Both	Esophageal cancer	Chewing tobacco	Percent	2021	0.99%	1.48%	0.56%
**ASDALYR**	High SDI	Male	Esophageal cancer	Smoking	Percent	2021	43.81%	52.47%	35.02%
**ASDALYR**	High SDI	Female	Esophageal cancer	Smoking	Percent	2021	23.98%	29.78%	18.05%
**ASDALYR**	High SDI	Both	Esophageal cancer	Smoking	Percent	2021	39.99%	47.98%	31.82%
**ASDALYR**	High SDI	Male	Esophageal cancer	High alcohol use	Percent	2021	24.48%	30.84%	18.07%
**ASDALYR**	High SDI	Female	Esophageal cancer	High alcohol use	Percent	2021	13.03%	17.03%	9.12%
**ASDALYR**	High SDI	Both	Esophageal cancer	High alcohol use	Percent	2021	22.53%	28.48%	16.67%
**ASDALYR**	High SDI	Male	Esophageal cancer	Diet low in vegetables	Percent	2021	15.43%	31.42%	-3.32%
**ASDALYR**	High SDI	Female	Esophageal cancer	Diet low in vegetables	Percent	2021	16.25%	32.83%	-3.56%
**ASDALYR**	High SDI	Both	Esophageal cancer	Diet low in vegetables	Percent	2021	15.55%	31.54%	-3.36%
**ASDALYR**	High SDI	Male	Esophageal cancer	Chewing tobacco	Percent	2021	2.10%	3.23%	1.20%
**ASDALYR**	High SDI	Female	Esophageal cancer	Chewing tobacco	Percent	2021	0.45%	0.73%	0.26%
**ASDALYR**	High SDI	Both	Esophageal cancer	Chewing tobacco	Percent	2021	1.80%	2.71%	1.05%
**ASDALYR**	Low-middle SDI	Male	Esophageal cancer	Smoking	Percent	2021	27.78%	33.15%	22.35%
**ASDALYR**	Low-middle SDI	Female	Esophageal cancer	Smoking	Percent	2021	3.96%	5.15%	2.90%
**ASDALYR**	Low-middle SDI	Both	Esophageal cancer	Smoking	Percent	2021	18.38%	22.18%	14.36%
**ASDALYR**	Low-middle SDI	Male	Esophageal cancer	High alcohol use	Percent	2021	12.39%	16.33%	8.62%
**ASDALYR**	Low-middle SDI	Female	Esophageal cancer	High alcohol use	Percent	2021	1.70%	2.51%	1.00%
**ASDALYR**	Low-middle SDI	Both	Esophageal cancer	High alcohol use	Percent	2021	8.26%	11.05%	5.67%
**ASDALYR**	Low-middle SDI	Male	Esophageal cancer	Diet low in vegetables	Percent	2021	22.31%	43.92%	-5.02%
**ASDALYR**	Low-middle SDI	Female	Esophageal cancer	Diet low in vegetables	Percent	2021	22.66%	44.31%	-5.07%
**ASDALYR**	Low-middle SDI	Both	Esophageal cancer	Diet low in vegetables	Percent	2021	22.45%	44.07%	-5.04%
**ASDALYR**	Low-middle SDI	Male	Esophageal cancer	Chewing tobacco	Percent	2021	14.44%	19.84%	9.31%
**ASDALYR**	Low-middle SDI	Female	Esophageal cancer	Chewing tobacco	Percent	2021	14.46%	20.51%	9.28%
**ASDALYR**	Low-middle SDI	Both	Esophageal cancer	Chewing tobacco	Percent	2021	14.48%	18.43%	10.39%

**ASDALYR**	Low SDI	Male	Esophageal cancer	Smoking	Percent	2021	15.10%	18.33%	11.85%
**ASDALYR**	Low SDI	Female	Esophageal cancer	Smoking	Percent	2021	3.14%	4.18%	2.34%
**ASDALYR**	Low SDI	Both	Esophageal cancer	Smoking	Percent	2021	9.96%	12.12%	7.77%
**ASDALYR**	Low SDI	Male	Esophageal cancer	High alcohol use	Percent	2021	11.52%	15.04%	7.64%
**ASDALYR**	Low SDI	Female	Esophageal cancer	High alcohol use	Percent	2021	3.23%	4.59%	1.91%
**ASDALYR**	Low SDI	Both	Esophageal cancer	High alcohol use	Percent	2021	7.99%	10.64%	5.26%
**ASDALYR**	Low SDI	Male	Esophageal cancer	Diet low in vegetables	Percent	2021	25.00%	48.13%	-5.86%
**ASDALYR**	Low SDI	Female	Esophageal cancer	Diet low in vegetables	Percent	2021	25.01%	48.24%	-5.74%
**ASDALYR**	Low SDI	Both	Esophageal cancer	Diet low in vegetables	Percent	2021	25.01%	48.18%	-5.81%
**ASDALYR**	Low SDI	Male	Esophageal cancer	Chewing tobacco	Percent	2021	7.44%	10.42%	4.68%
**ASDALYR**	Low SDI	Female	Esophageal cancer	Chewing tobacco	Percent	2021	5.59%	9.38%	3.31%
**ASDALYR**	Low SDI	Both	Esophageal cancer	Chewing tobacco	Percent	2021	6.64%	8.76%	4.71%
**ASDALYR**	Middle SDI	Male	Esophageal cancer	Smoking	Percent	2021	48.64%	56.15%	40.52%
**ASDALYR**	Middle SDI	Female	Esophageal cancer	Smoking	Percent	2021	5.44%	7.37%	3.93%
**ASDALYR**	Middle SDI	Both	Esophageal cancer	Smoking	Percent	2021	38.37%	45.00%	31.23%
**ASDALYR**	Middle SDI	Male	Esophageal cancer	High alcohol use	Percent	2021	18.98%	24.27%	14.22%
**ASDALYR**	Middle SDI	Female	Esophageal cancer	High alcohol use	Percent	2021	2.01%	2.88%	1.29%
**ASDALYR**	Middle SDI	Both	Esophageal cancer	High alcohol use	Percent	2021	15.17%	19.77%	11.45%
**ASDALYR**	Middle SDI	Male	Esophageal cancer	Diet low in vegetables	Percent	2021	8.06%	17.60%	-1.92%
**ASDALYR**	Middle SDI	Female	Esophageal cancer	Diet low in vegetables	Percent	2021	9.69%	20.74%	-2.24%
**ASDALYR**	Middle SDI	Both	Esophageal cancer	Diet low in vegetables	Percent	2021	8.40%	18.02%	-1.94%
**ASDALYR**	Middle SDI	Male	Esophageal cancer	Chewing tobacco	Percent	2021	2.57%	3.77%	1.50%
**ASDALYR**	Middle SDI	Female	Esophageal cancer	Chewing tobacco	Percent	2021	3.40%	5.03%	2.00%
**ASDALYR**	Middle SDI	Both	Esophageal cancer	Chewing tobacco	Percent	2021	2.78%	3.68%	1.87%

Supplementary Table S12 | The death rate (DR) and DALYR of males, females, and both sexes of five age-groups in world and regions with five SDI quintiles attributable to the four most detailed risks in 1990 and 2021

measure_name	location_name	sex_name	age_name	rei_name	year	val	upper	lower
**DR**	Global	Both	20-54 years	Alcohol use	1990	0.55	0.72	0.39
**DR**	Global	Both	20-54 years	Diet low in vegetables	1990	0.60	1.19	-0.13
**DR**	Global	Both	20-54 years	Smoking	1990	0.94	1.14	0.75
**DR**	Global	Both	20-54 years	Chewing tobacco	1990	0.10	0.14	0.07
**DALYR**	Global	Both	20-54 years	Diet low in vegetables	1990	25.68	50.81	-5.58
**DALYR**	Global	Both	20-54 years	Alcohol use	1990	23.56	31.12	17.03
**DALYR**	Global	Both	20-54 years	Smoking	1990	39.53	48.36	31.52
**DALYR**	Global	Both	20-54 years	Chewing tobacco	1990	4.46	6.07	3.05
**DR**	Global	Female	20-54 years	Alcohol use	1990	0.05	0.07	0.03
**DR**	Global	Female	20-54 years	Diet low in vegetables	1990	0.27	0.56	-0.05
**DR**	Global	Female	20-54 years	Smoking	1990	0.07	0.09	0.05
**DR**	Global	Female	20-54 years	Chewing tobacco	1990	0.06	0.09	0.04
**DALYR**	Global	Female	20-54 years	Diet low in vegetables	1990	11.82	24.05	-2.36
**DALYR**	Global	Female	20-54 years	Alcohol use	1990	2.09	2.87	1.36
**DALYR**	Global	Female	20-54 years	Smoking	1990	2.83	3.60	2.14
**DALYR**	Global	Female	20-54 years	Chewing tobacco	1990	2.48	3.76	1.49
**DR**	Global	Male	20-54 years	Alcohol use	1990	1.03	1.37	0.75
**DR**	Global	Male	20-54 years	Diet low in vegetables	1990	0.91	1.83	-0.20
**DR**	Global	Male	20-54 years	Smoking	1990	1.78	2.18	1.42
**DR**	Global	Male	20-54 years	Chewing tobacco	1990	0.15	0.22	0.09
**DALYR**	Global	Male	20-54 years	Diet low in vegetables	1990	39.19	78.69	-8.41
**DALYR**	Global	Male	20-54 years	Alcohol use	1990	44.50	59.06	32.41
**DALYR**	Global	Male	20-54 years	Smoking	1990	75.33	92.32	60.00
**DALYR**	Global	Male	20-54 years	Chewing tobacco	1990	6.40	9.30	3.81
**DR**	Global	Both	20-54 years	Alcohol use	2021	0.36	0.47	0.26
**DR**	Global	Both	20-54 years	Diet low in vegetables	2021	0.23	0.47	-0.05

**DR**	Global	Both	20-54 years	Smoking	2021	0.51	0.65	0.39
**DR**	Global	Both	20-54 years	Chewing tobacco	2021	0.09	0.12	0.06
**DALYR**	Global	Both	20-54 years	Diet low in vegetables	2021	9.95	19.97	-2.08
**DALYR**	Global	Both	20-54 years	Alcohol use	2021	15.03	19.79	10.95
**DALYR**	Global	Both	20-54 years	Smoking	2021	21.09	26.81	16.15
**DALYR**	Global	Both	20-54 years	Chewing tobacco	2021	3.94	5.26	2.72
**DR**	Global	Female	20-54 years	Alcohol use	2021	0.03	0.04	0.02
**DR**	Global	Female	20-54 years	Diet low in vegetables	2021	0.14	0.28	-0.03
**DR**	Global	Female	20-54 years	Smoking	2021	0.03	0.04	0.02
**DR**	Global	Female	20-54 years	Chewing tobacco	2021	0.05	0.08	0.03
**DALYR**	Global	Female	20-54 years	Diet low in vegetables	2021	6.05	12.10	-1.27
**DALYR**	Global	Female	20-54 years	Alcohol use	2021	1.34	1.84	0.91
**DALYR**	Global	Female	20-54 years	Smoking	2021	1.32	1.65	1.01
**DALYR**	Global	Female	20-54 years	Chewing tobacco	2021	2.09	3.23	1.18
**DR**	Global	Male	20-54 years	Alcohol use	2021	0.67	0.89	0.49
**DR**	Global	Male	20-54 years	Diet low in vegetables	2021	0.32	0.66	-0.07
**DR**	Global	Male	20-54 years	Smoking	2021	0.98	1.25	0.75
**DR**	Global	Male	20-54 years	Chewing tobacco	2021	0.13	0.20	0.08
**DALYR**	Global	Male	20-54 years	Diet low in vegetables	2021	13.79	28.09	-2.89
**DALYR**	Global	Male	20-54 years	Alcohol use	2021	28.47	37.59	20.74
**DALYR**	Global	Male	20-54 years	Smoking	2021	40.51	51.61	30.99
**DALYR**	Global	Male	20-54 years	Chewing tobacco	2021	5.76	8.42	3.42
**DR**	Global	Both	55-59 years	Diet low in vegetables	1990	5.34	10.52	-1.18
**DR**	Global	Both	55-59 years	Smoking	1990	9.84	11.98	7.95
**DR**	Global	Both	55-59 years	Chewing tobacco	1990	0.77	1.04	0.50
**DR**	Global	Both	55-59 years	Alcohol use	1990	4.41	5.79	3.19
**DALYR**	Global	Both	55-59 years	Diet low in vegetables	1990	180.38	355.21	-39.79
**DALYR**	Global	Both	55-59 years	Smoking	1990	332.46	404.90	268.84
**DALYR**	Global	Both	55-59 years	Chewing tobacco	1990	25.92	35.20	16.86

**DALYR**	Global	Both	55-59 years	Alcohol use	1990	149.00	195.71	107.72
**DR**	Global	Female	55-59 years	Diet low in vegetables	1990	2.65	5.33	-0.51
**DR**	Global	Female	55-59 years	Smoking	1990	1.01	1.33	0.71
**DR**	Global	Female	55-59 years	Chewing tobacco	1990	0.49	0.76	0.27
**DR**	Global	Female	55-59 years	Alcohol use	1990	0.43	0.60	0.27
**DALYR**	Global	Female	55-59 years	Diet low in vegetables	1990	89.62	180.10	-17.19
**DALYR**	Global	Female	55-59 years	Smoking	1990	34.10	45.12	24.08
**DALYR**	Global	Female	55-59 years	Chewing tobacco	1990	16.44	25.69	8.99
**DALYR**	Global	Female	55-59 years	Alcohol use	1990	14.41	20.31	9.13
**DR**	Global	Male	55-59 years	Diet low in vegetables	1990	8.01	16.05	-1.78
**DR**	Global	Male	55-59 years	Smoking	1990	18.61	22.74	14.88
**DR**	Global	Male	55-59 years	Chewing tobacco	1990	1.05	1.59	0.63
**DR**	Global	Male	55-59 years	Alcohol use	1990	8.37	11.14	6.05
**DALYR**	Global	Male	55-59 years	Diet low in vegetables	1990	270.57	541.60	-60.28
**DALYR**	Global	Male	55-59 years	Smoking	1990	628.96	768.17	503.77
**DALYR**	Global	Male	55-59 years	Chewing tobacco	1990	35.35	53.75	21.23
**DALYR**	Global	Male	55-59 years	Alcohol use	1990	282.77	376.44	204.44
**DR**	Global	Both	55-59 years	Diet low in vegetables	2021	1.58	3.29	-0.34
**DR**	Global	Both	55-59 years	Smoking	2021	5.23	6.69	3.93
**DR**	Global	Both	55-59 years	Chewing tobacco	2021	0.60	0.84	0.40
**DR**	Global	Both	55-59 years	Alcohol use	2021	2.67	3.65	1.93
**DALYR**	Global	Both	55-59 years	Diet low in vegetables	2021	53.49	111.51	-11.45
**DALYR**	Global	Both	55-59 years	Smoking	2021	177.22	226.55	133.27
**DALYR**	Global	Both	55-59 years	Chewing tobacco	2021	20.27	28.45	13.36
**DALYR**	Global	Both	55-59 years	Alcohol use	2021	90.50	123.85	65.55
**DR**	Global	Female	55-59 years	Diet low in vegetables	2021	0.87	1.77	-0.18
**DR**	Global	Female	55-59 years	Smoking	2021	0.40	0.52	0.30
**DR**	Global	Female	55-59 years	Chewing tobacco	2021	0.35	0.56	0.20
**DR**	Global	Female	55-59 years	Alcohol use	2021	0.22	0.31	0.15

**DALYR**	Global	Female	55-59 years	Diet low in vegetables	2021	29.32	59.92	-6.17
**DALYR**	Global	Female	55-59 years	Smoking	2021	13.63	17.81	10.15
**DALYR**	Global	Female	55-59 years	Chewing tobacco	2021	12.01	19.00	6.82
**DALYR**	Global	Female	55-59 years	Alcohol use	2021	7.54	10.53	5.06
**DR**	Global	Male	55-59 years	Diet low in vegetables	2021	2.32	4.79	-0.50
**DR**	Global	Male	55-59 years	Smoking	2021	10.21	13.12	7.66
**DR**	Global	Male	55-59 years	Chewing tobacco	2021	0.85	1.31	0.48
**DR**	Global	Male	55-59 years	Alcohol use	2021	5.20	7.15	3.76
**DALYR**	Global	Male	55-59 years	Diet low in vegetables	2021	78.44	162.27	-16.95
**DALYR**	Global	Male	55-59 years	Smoking	2021	346.09	444.36	259.17
**DALYR**	Global	Male	55-59 years	Chewing tobacco	2021	28.79	44.40	16.30
**DALYR**	Global	Male	55-59 years	Alcohol use	2021	176.12	242.16	127.24
**DR**	Global	Both	60-64 years	Diet low in vegetables	1990	7.29	14.47	-1.57
**DR**	Global	Both	60-64 years	Smoking	1990	14.20	17.23	11.30
**DR**	Global	Both	60-64 years	Chewing tobacco	1990	1.02	1.38	0.69
**DR**	Global	Both	60-64 years	Alcohol use	1990	5.55	7.32	3.93
**DALYR**	Global	Both	60-64 years	Diet low in vegetables	1990	212.58	421.09	-45.87
**DALYR**	Global	Both	60-64 years	Smoking	1990	414.02	502.34	329.17
**DALYR**	Global	Both	60-64 years	Chewing tobacco	1990	29.76	40.18	20.00
**DALYR**	Global	Both	60-64 years	Alcohol use	1990	161.76	213.43	114.47
**DR**	Global	Female	60-64 years	Diet low in vegetables	1990	3.86	8.03	-0.76
**DR**	Global	Female	60-64 years	Smoking	1990	1.83	2.53	1.26
**DR**	Global	Female	60-64 years	Chewing tobacco	1990	0.57	0.92	0.33
**DR**	Global	Female	60-64 years	Alcohol use	1990	0.67	0.98	0.43
**DALYR**	Global	Female	60-64 years	Diet low in vegetables	1990	112.52	233.68	-22.12
**DALYR**	Global	Female	60-64 years	Smoking	1990	53.23	73.65	36.76
**DALYR**	Global	Female	60-64 years	Chewing tobacco	1990	16.67	26.86	9.49
**DALYR**	Global	Female	60-64 years	Alcohol use	1990	19.59	28.64	12.40
**DR**	Global	Male	60-64 years	Diet low in vegetables	1990	10.88	21.93	-2.36

**DR**	Global	Male	60-64 years	Smoking	1990	27.12	33.03	21.53
**DR**	Global	Male	60-64 years	Chewing tobacco	1990	1.49	2.20	0.89
**DR**	Global	Male	60-64 years	Alcohol use	1990	10.64	14.18	7.57
**DALYR**	Global	Male	60-64 years	Diet low in vegetables	1990	317.12	639.86	-68.81
**DALYR**	Global	Male	60-64 years	Smoking	1990	790.96	965.32	628.12
**DALYR**	Global	Male	60-64 years	Chewing tobacco	1990	43.44	64.28	25.85
**DALYR**	Global	Male	60-64 years	Alcohol use	1990	310.30	413.42	220.73
**DR**	Global	Both	60-64 years	Diet low in vegetables	2021	2.21	4.58	-0.45
**DR**	Global	Both	60-64 years	Smoking	2021	7.88	9.93	6.00
**DR**	Global	Both	60-64 years	Chewing tobacco	2021	0.82	1.10	0.56
**DR**	Global	Both	60-64 years	Alcohol use	2021	3.57	4.76	2.54
**DALYR**	Global	Both	60-64 years	Diet low in vegetables	2021	64.52	133.87	-13.16
**DALYR**	Global	Both	60-64 years	Smoking	2021	229.76	289.00	175.07
**DALYR**	Global	Both	60-64 years	Chewing tobacco	2021	24.00	32.14	16.36
**DALYR**	Global	Both	60-64 years	Alcohol use	2021	104.18	138.67	74.20
**DR**	Global	Female	60-64 years	Diet low in vegetables	2021	1.16	2.36	-0.25
**DR**	Global	Female	60-64 years	Smoking	2021	0.67	0.89	0.49
**DR**	Global	Female	60-64 years	Chewing tobacco	2021	0.44	0.72	0.25
**DR**	Global	Female	60-64 years	Alcohol use	2021	0.35	0.48	0.23
**DALYR**	Global	Female	60-64 years	Diet low in vegetables	2021	33.87	68.99	-7.29
**DALYR**	Global	Female	60-64 years	Smoking	2021	19.61	25.92	14.41
**DALYR**	Global	Female	60-64 years	Chewing tobacco	2021	12.81	20.93	7.21
**DALYR**	Global	Female	60-64 years	Alcohol use	2021	10.31	13.98	6.75
**DR**	Global	Male	60-64 years	Diet low in vegetables	2021	3.32	6.96	-0.67
**DR**	Global	Male	60-64 years	Smoking	2021	15.51	19.59	11.81
**DR**	Global	Male	60-64 years	Chewing tobacco	2021	1.23	1.83	0.72
**DR**	Global	Male	60-64 years	Alcohol use	2021	6.98	9.35	4.96
**DALYR**	Global	Male	60-64 years	Diet low in vegetables	2021	96.94	202.92	-19.69
**DALYR**	Global	Male	60-64 years	Smoking	2021	452.03	570.22	344.63

**DALYR**	Global	Male	60-64 years	Chewing tobacco	2021	35.84	53.41	21.15
**DALYR**	Global	Male	60-64 years	Alcohol use	2021	203.46	272.32	144.69
**DR**	Global	Both	65-74 years	Alcohol use	1990	6.79	9.06	4.75
**DR**	Global	Both	65-74 years	Diet low in vegetables	1990	10.99	21.52	-2.41
**DR**	Global	Both	65-74 years	Smoking	1990	21.77	25.91	17.43
**DR**	Global	Both	65-74 years	Chewing tobacco	1990	1.22	1.65	0.86
**DALYR**	Global	Both	65-74 years	Smoking	1990	490.10	583.23	392.62
**DALYR**	Global	Both	65-74 years	Diet low in vegetables	1990	247.18	483.20	-54.23
**DALYR**	Global	Both	65-74 years	Chewing tobacco	1990	27.57	37.16	19.45
**DALYR**	Global	Both	65-74 years	Alcohol use	1990	153.94	205.37	107.52
**DR**	Global	Female	65-74 years	Alcohol use	1990	0.98	1.37	0.65
**DR**	Global	Female	65-74 years	Diet low in vegetables	1990	6.57	13.21	-1.24
**DR**	Global	Female	65-74 years	Smoking	1990	3.75	5.01	2.60
**DR**	Global	Female	65-74 years	Chewing tobacco	1990	0.77	1.14	0.46
**DALYR**	Global	Female	65-74 years	Smoking	1990	83.00	110.05	57.89
**DALYR**	Global	Female	65-74 years	Diet low in vegetables	1990	146.69	295.08	-27.61
**DALYR**	Global	Female	65-74 years	Chewing tobacco	1990	17.38	25.44	10.27
**DALYR**	Global	Female	65-74 years	Alcohol use	1990	21.98	30.94	14.50
**DR**	Global	Male	65-74 years	Alcohol use	1990	13.72	18.32	9.61
**DR**	Global	Male	65-74 years	Diet low in vegetables	1990	16.27	32.58	-3.71
**DR**	Global	Male	65-74 years	Smoking	1990	43.29	52.21	34.68
**DR**	Global	Male	65-74 years	Chewing tobacco	1990	1.75	2.57	1.12
**DALYR**	Global	Male	65-74 years	Smoking	1990	975.97	1175.45	782.97
**DALYR**	Global	Male	65-74 years	Diet low in vegetables	1990	367.11	735.44	-83.45
**DALYR**	Global	Male	65-74 years	Chewing tobacco	1990	39.73	58.24	25.47
**DALYR**	Global	Male	65-74 years	Alcohol use	1990	311.43	415.10	218.21
**DR**	Global	Both	65-74 years	Alcohol use	2021	5.45	7.39	3.88
**DR**	Global	Both	65-74 years	Diet low in vegetables	2021	3.34	6.92	-0.67
**DR**	Global	Both	65-74 years	Smoking	2021	15.18	19.08	11.60

**DR**	Global	Both	65-74 years	Chewing tobacco	2021	1.07	1.46	0.72
**DALYR**	Global	Both	65-74 years	Smoking	2021	339.61	425.34	259.23
**DALYR**	Global	Both	65-74 years	Diet low in vegetables	2021	74.87	155.00	-15.01
**DALYR**	Global	Both	65-74 years	Alcohol use	2021	122.52	166.01	87.22
**DALYR**	Global	Both	65-74 years	Chewing tobacco	2021	24.09	32.96	16.14
**DR**	Global	Female	65-74 years	Alcohol use	2021	0.54	0.77	0.35
**DR**	Global	Female	65-74 years	Diet low in vegetables	2021	1.82	3.67	-0.39
**DR**	Global	Female	65-74 years	Smoking	2021	1.45	1.98	1.02
**DR**	Global	Female	65-74 years	Chewing tobacco	2021	0.68	1.00	0.41
**DALYR**	Global	Female	65-74 years	Smoking	2021	32.13	43.74	22.80
**DALYR**	Global	Female	65-74 years	Diet low in vegetables	2021	40.56	81.64	-8.64
**DALYR**	Global	Female	65-74 years	Alcohol use	2021	12.18	17.27	7.90
**DALYR**	Global	Female	65-74 years	Chewing tobacco	2021	15.09	22.46	9.12
**DR**	Global	Male	65-74 years	Alcohol use	2021	10.90	14.82	7.75
**DR**	Global	Male	65-74 years	Diet low in vegetables	2021	5.02	10.50	-1.00
**DR**	Global	Male	65-74 years	Smoking	2021	30.44	38.09	23.21
**DR**	Global	Male	65-74 years	Chewing tobacco	2021	1.51	2.25	0.89
**DALYR**	Global	Male	65-74 years	Smoking	2021	681.08	851.01	519.18
**DALYR**	Global	Male	65-74 years	Diet low in vegetables	2021	112.96	238.30	-22.33
**DALYR**	Global	Male	65-74 years	Alcohol use	2021	245.06	332.98	173.96
**DALYR**	Global	Male	65-74 years	Chewing tobacco	2021	34.08	50.39	20.00
**DR**	Global	Both	75+ years	Alcohol use	1990	6.23	8.18	4.33
**DR**	Global	Both	75+ years	Diet low in vegetables	1990	13.29	26.58	-2.95
**DR**	Global	Both	75+ years	Smoking	1990	23.49	28.24	18.56
**DR**	Global	Both	75+ years	Chewing tobacco	1990	1.39	1.83	0.95
**DALYR**	Global	Both	75+ years	Smoking	1990	331.74	397.73	261.94
**DALYR**	Global	Both	75+ years	Diet low in vegetables	1990	186.35	372.10	-41.15
**DALYR**	Global	Both	75+ years	Alcohol use	1990	88.70	116.89	62.43
**DALYR**	Global	Both	75+ years	Chewing tobacco	1990	19.28	25.29	13.23

**DR**	Global	Female	75+ years	Alcohol use	1990	1.60	2.31	0.96
**DR**	Global	Female	75+ years	Diet low in vegetables	1990	9.32	18.79	-1.87
**DR**	Global	Female	75+ years	Smoking	1990	6.11	8.18	4.18
**DR**	Global	Female	75+ years	Chewing tobacco	1990	0.98	1.42	0.57
**DALYR**	Global	Female	75+ years	Smoking	1990	80.80	107.68	55.50
**DALYR**	Global	Female	75+ years	Diet low in vegetables	1990	128.09	258.27	-25.48
**DALYR**	Global	Female	75+ years	Alcohol use	1990	21.47	30.65	13.16
**DALYR**	Global	Female	75+ years	Chewing tobacco	1990	13.45	19.40	7.79
**DR**	Global	Male	75+ years	Alcohol use	1990	13.63	17.87	9.69
**DR**	Global	Male	75+ years	Diet low in vegetables	1990	19.65	39.44	-4.41
**DR**	Global	Male	75+ years	Smoking	1990	51.32	61.49	40.94
**DR**	Global	Male	75+ years	Chewing tobacco	1990	2.06	2.90	1.23
**DALYR**	Global	Male	75+ years	Smoking	1990	733.71	877.85	584.13
**DALYR**	Global	Male	75+ years	Diet low in vegetables	1990	279.67	561.11	-62.43
**DALYR**	Global	Male	75+ years	Alcohol use	1990	196.39	259.02	139.93
**DALYR**	Global	Male	75+ years	Chewing tobacco	1990	28.62	40.54	17.28
**DR**	Global	Both	75+ years	Alcohol use	2021	6.79	9.07	4.61
**DR**	Global	Both	75+ years	Diet low in vegetables	2021	6.52	13.53	-1.46
**DR**	Global	Both	75+ years	Smoking	2021	23.26	29.30	17.53
**DR**	Global	Both	75+ years	Chewing tobacco	2021	1.49	2.02	1.03
**DALYR**	Global	Both	75+ years	Diet low in vegetables	2021	84.18	175.47	-19.01
**DALYR**	Global	Both	75+ years	Alcohol use	2021	91.84	122.33	62.99
**DALYR**	Global	Both	75+ years	Smoking	2021	311.12	391.66	234.33
**DALYR**	Global	Both	75+ years	Chewing tobacco	2021	19.93	27.00	13.90
**DR**	Global	Female	75+ years	Alcohol use	2021	1.09	1.52	0.68
**DR**	Global	Female	75+ years	Diet low in vegetables	2021	4.02	8.87	-0.83
**DR**	Global	Female	75+ years	Smoking	2021	3.59	5.23	2.26
**DR**	Global	Female	75+ years	Chewing tobacco	2021	1.07	1.56	0.62
**DALYR**	Global	Female	75+ years	Diet low in vegetables	2021	50.61	112.56	-10.46

**DALYR**	Global	Female	75+ years	Alcohol use	2021	13.69	19.15	8.62
**DALYR**	Global	Female	75+ years	Smoking	2021	44.87	65.58	28.66
**DALYR**	Global	Female	75+ years	Chewing tobacco	2021	14.15	20.55	8.27
**DR**	Global	Male	75+ years	Alcohol use	2021	14.68	19.52	10.00
**DR**	Global	Male	75+ years	Diet low in vegetables	2021	9.99	21.37	-2.38
**DR**	Global	Male	75+ years	Smoking	2021	50.49	63.40	38.21
**DR**	Global	Male	75+ years	Chewing tobacco	2021	2.08	2.97	1.26
**DALYR**	Global	Male	75+ years	Diet low in vegetables	2021	130.63	278.10	-31.42
**DALYR**	Global	Male	75+ years	Alcohol use	2021	200.01	265.44	137.33
**DALYR**	Global	Male	75+ years	Smoking	2021	679.59	855.65	510.98
**DALYR**	Global	Male	75+ years	Chewing tobacco	2021	27.91	40.52	16.68
**DR**	High SDI	Both	20-54 years	Alcohol use	1990	0.46	0.57	0.36
**DR**	High SDI	Both	20-54 years	Smoking	1990	0.67	0.78	0.56
**DR**	High SDI	Both	20-54 years	Chewing tobacco	1990	0.03	0.04	0.01
**DR**	High SDI	Both	20-54 years	Diet low in vegetables	1990	0.30	0.59	-0.06
**DALYR**	High SDI	Both	20-54 years	Smoking	1990	27.92	32.57	23.10
**DALYR**	High SDI	Both	20-54 years	Chewing tobacco	1990	1.10	1.68	0.63
**DALYR**	High SDI	Both	20-54 years	Diet low in vegetables	1990	12.50	25.12	-2.68
**DALYR**	High SDI	Both	20-54 years	Alcohol use	1990	19.65	24.38	15.12
**DR**	High SDI	Female	20-54 years	Alcohol use	1990	0.07	0.10	0.05
**DR**	High SDI	Female	20-54 years	Smoking	1990	0.11	0.13	0.09
**DR**	High SDI	Female	20-54 years	Chewing tobacco	1990	0.00	0.00	0.00
**DR**	High SDI	Female	20-54 years	Diet low in vegetables	1990	0.08	0.16	-0.02
**DALYR**	High SDI	Female	20-54 years	Smoking	1990	4.56	5.45	3.66
**DALYR**	High SDI	Female	20-54 years	Chewing tobacco	1990	0.07	0.12	0.04
**DALYR**	High SDI	Female	20-54 years	Diet low in vegetables	1990	3.36	6.78	-0.74
**DALYR**	High SDI	Female	20-54 years	Alcohol use	1990	3.17	4.17	2.24
**DR**	High SDI	Male	20-54 years	Alcohol use	1990	0.84	1.04	0.65
**DR**	High SDI	Male	20-54 years	Smoking	1990	1.22	1.42	1.01

**DR**	High SDI	Male	20-54 years	Chewing tobacco	1990	0.05	0.08	0.03
**DR**	High SDI	Male	20-54 years	Diet low in vegetables	1990	0.51	1.02	-0.11
**DALYR**	High SDI	Male	20-54 years	Smoking	1990	50.72	59.01	41.90
**DALYR**	High SDI	Male	20-54 years	Chewing tobacco	1990	2.11	3.27	1.16
**DALYR**	High SDI	Male	20-54 years	Diet low in vegetables	1990	21.42	43.08	-4.58
**DALYR**	High SDI	Male	20-54 years	Alcohol use	1990	35.73	44.22	27.65
**DR**	High SDI	Both	20-54 years	Alcohol use	2021	0.33	0.41	0.25
**DR**	High SDI	Both	20-54 years	Smoking	2021	0.39	0.46	0.31
**DR**	High SDI	Both	20-54 years	Chewing tobacco	2021	0.02	0.04	0.01
**DR**	High SDI	Both	20-54 years	Diet low in vegetables	2021	0.19	0.39	-0.04
**DALYR**	High SDI	Both	20-54 years	Smoking	2021	15.99	19.07	12.91
**DALYR**	High SDI	Both	20-54 years	Chewing tobacco	2021	1.04	1.60	0.61
**DALYR**	High SDI	Both	20-54 years	Diet low in vegetables	2021	8.09	16.25	-1.73
**DALYR**	High SDI	Both	20-54 years	Alcohol use	2021	13.77	17.13	10.42
**DR**	High SDI	Female	20-54 years	Alcohol use	2021	0.06	0.08	0.04
**DR**	High SDI	Female	20-54 years	Smoking	2021	0.07	0.09	0.05
**DR**	High SDI	Female	20-54 years	Chewing tobacco	2021	0.00	0.00	0.00
**DR**	High SDI	Female	20-54 years	Diet low in vegetables	2021	0.06	0.13	-0.01
**DALYR**	High SDI	Female	20-54 years	Smoking	2021	2.91	3.57	2.27
**DALYR**	High SDI	Female	20-54 years	Chewing tobacco	2021	0.08	0.13	0.04
**DALYR**	High SDI	Female	20-54 years	Diet low in vegetables	2021	2.69	5.63	-0.57
**DALYR**	High SDI	Female	20-54 years	Alcohol use	2021	2.72	3.61	1.86
**DR**	High SDI	Male	20-54 years	Alcohol use	2021	0.57	0.71	0.43
**DR**	High SDI	Male	20-54 years	Smoking	2021	0.69	0.82	0.56
**DR**	High SDI	Male	20-54 years	Chewing tobacco	2021	0.05	0.07	0.03
**DR**	High SDI	Male	20-54 years	Diet low in vegetables	2021	0.31	0.63	-0.07
**DALYR**	High SDI	Male	20-54 years	Smoking	2021	28.36	33.87	22.91
**DALYR**	High SDI	Male	20-54 years	Chewing tobacco	2021	1.95	3.05	1.12
**DALYR**	High SDI	Male	20-54 years	Diet low in vegetables	2021	13.20	26.38	-2.84

**DALYR**	High SDI	Male	20-54 years	Alcohol use	2021	24.21	30.11	18.10
**DR**	High SDI	Both	55-59 years	Alcohol use	1990	3.65	4.62	2.74
**DR**	High SDI	Both	55-59 years	Diet low in vegetables	1990	2.50	5.05	-0.56
**DR**	High SDI	Both	55-59 years	Smoking	1990	7.02	8.16	5.82
**DR**	High SDI	Both	55-59 years	Chewing tobacco	1990	0.20	0.34	0.11
**DALYR**	High SDI	Both	55-59 years	Smoking	1990	237.42	275.88	196.59
**DALYR**	High SDI	Both	55-59 years	Alcohol use	1990	123.62	156.14	92.38
**DALYR**	High SDI	Both	55-59 years	Chewing tobacco	1990	6.91	11.46	3.75
**DALYR**	High SDI	Both	55-59 years	Diet low in vegetables	1990	84.64	170.70	-18.93
**DR**	High SDI	Female	55-59 years	Alcohol use	1990	0.54	0.71	0.38
**DR**	High SDI	Female	55-59 years	Diet low in vegetables	1990	0.69	1.43	-0.15
**DR**	High SDI	Female	55-59 years	Smoking	1990	1.15	1.39	0.91
**DR**	High SDI	Female	55-59 years	Chewing tobacco	1990	0.02	0.03	0.01
**DALYR**	High SDI	Female	55-59 years	Smoking	1990	38.99	46.94	30.84
**DALYR**	High SDI	Female	55-59 years	Alcohol use	1990	18.33	23.93	12.85
**DALYR**	High SDI	Female	55-59 years	Chewing tobacco	1990	0.57	1.02	0.28
**DALYR**	High SDI	Female	55-59 years	Diet low in vegetables	1990	23.52	48.43	-5.22
**DR**	High SDI	Male	55-59 years	Alcohol use	1990	6.91	8.72	5.12
**DR**	High SDI	Male	55-59 years	Diet low in vegetables	1990	4.39	8.85	-0.98
**DR**	High SDI	Male	55-59 years	Smoking	1990	13.15	15.26	10.92
**DR**	High SDI	Male	55-59 years	Chewing tobacco	1990	0.40	0.68	0.21
**DALYR**	High SDI	Male	55-59 years	Smoking	1990	444.88	516.13	368.79
**DALYR**	High SDI	Male	55-59 years	Alcohol use	1990	233.69	294.72	173.23
**DALYR**	High SDI	Male	55-59 years	Chewing tobacco	1990	13.53	22.86	7.18
**DALYR**	High SDI	Male	55-59 years	Diet low in vegetables	1990	148.55	299.01	-33.26
**DR**	High SDI	Both	55-59 years	Alcohol use	2021	2.29	2.89	1.66
**DR**	High SDI	Both	55-59 years	Diet low in vegetables	2021	1.40	2.84	-0.29
**DR**	High SDI	Both	55-59 years	Smoking	2021	3.64	4.34	2.93
**DR**	High SDI	Both	55-59 years	Chewing tobacco	2021	0.16	0.28	0.08

**DALYR**	High SDI	Both	55-59 years	Smoking	2021	123.30	147.29	99.37
**DALYR**	High SDI	Both	55-59 years	Alcohol use	2021	77.70	98.15	56.23
**DALYR**	High SDI	Both	55-59 years	Chewing tobacco	2021	5.55	9.61	2.81
**DALYR**	High SDI	Both	55-59 years	Diet low in vegetables	2021	47.36	96.37	-9.91
**DR**	High SDI	Female	55-59 years	Alcohol use	2021	0.42	0.55	0.29
**DR**	High SDI	Female	55-59 years	Diet low in vegetables	2021	0.44	0.89	-0.09
**DR**	High SDI	Female	55-59 years	Smoking	2021	0.70	0.86	0.55
**DR**	High SDI	Female	55-59 years	Chewing tobacco	2021	0.01	0.02	0.01
**DALYR**	High SDI	Female	55-59 years	Smoking	2021	23.64	29.35	18.77
**DALYR**	High SDI	Female	55-59 years	Alcohol use	2021	14.35	18.75	9.95
**DALYR**	High SDI	Female	55-59 years	Chewing tobacco	2021	0.45	0.84	0.23
**DALYR**	High SDI	Female	55-59 years	Diet low in vegetables	2021	14.80	30.15	-3.16
**DR**	High SDI	Male	55-59 years	Alcohol use	2021	4.18	5.27	3.02
**DR**	High SDI	Male	55-59 years	Diet low in vegetables	2021	2.37	4.85	-0.50
**DR**	High SDI	Male	55-59 years	Smoking	2021	6.60	7.88	5.31
**DR**	High SDI	Male	55-59 years	Chewing tobacco	2021	0.32	0.56	0.16
**DALYR**	High SDI	Male	55-59 years	Smoking	2021	223.74	267.33	180.25
**DALYR**	High SDI	Male	55-59 years	Alcohol use	2021	141.55	178.55	102.76
**DALYR**	High SDI	Male	55-59 years	Chewing tobacco	2021	10.68	18.92	5.25
**DALYR**	High SDI	Male	55-59 years	Diet low in vegetables	2021	80.17	164.22	-16.82
**DR**	High SDI	Both	60-64 years	Alcohol use	1990	4.64	5.97	3.32
**DR**	High SDI	Both	60-64 years	Diet low in vegetables	1990	3.60	7.39	-0.78
**DR**	High SDI	Both	60-64 years	Smoking	1990	10.66	12.29	8.76
**DR**	High SDI	Both	60-64 years	Chewing tobacco	1990	0.34	0.53	0.19
**DALYR**	High SDI	Both	60-64 years	Smoking	1990	311.16	358.05	256.10
**DALYR**	High SDI	Both	60-64 years	Alcohol use	1990	135.25	174.03	96.69
**DALYR**	High SDI	Both	60-64 years	Chewing tobacco	1990	9.91	15.50	5.63
**DALYR**	High SDI	Both	60-64 years	Diet low in vegetables	1990	105.12	215.14	-22.74
**DR**	High SDI	Female	60-64 years	Alcohol use	1990	0.80	1.06	0.55

**DR**	High SDI	Female	60-64 years	Diet low in vegetables	1990	1.15	2.31	-0.25
**DR**	High SDI	Female	60-64 years	Smoking	1990	2.09	2.53	1.62
**DR**	High SDI	Female	60-64 years	Chewing tobacco	1990	0.02	0.04	0.01
**DALYR**	High SDI	Female	60-64 years	Smoking	1990	60.85	73.81	47.35
**DALYR**	High SDI	Female	60-64 years	Alcohol use	1990	23.27	30.88	16.05
**DALYR**	High SDI	Female	60-64 years	Chewing tobacco	1990	0.72	1.31	0.35
**DALYR**	High SDI	Female	60-64 years	Diet low in vegetables	1990	33.46	67.33	-7.39
**DR**	High SDI	Male	60-64 years	Alcohol use	1990	8.90	11.45	6.35
**DR**	High SDI	Male	60-64 years	Diet low in vegetables	1990	6.34	13.05	-1.37
**DR**	High SDI	Male	60-64 years	Smoking	1990	20.21	23.18	16.71
**DR**	High SDI	Male	60-64 years	Chewing tobacco	1990	0.69	1.10	0.39
**DALYR**	High SDI	Male	60-64 years	Smoking	1990	589.59	676.42	488.67
**DALYR**	High SDI	Male	60-64 years	Alcohol use	1990	259.81	334.05	185.24
**DALYR**	High SDI	Male	60-64 years	Chewing tobacco	1990	20.14	32.01	11.27
**DALYR**	High SDI	Male	60-64 years	Diet low in vegetables	1990	184.84	380.53	-39.86
**DR**	High SDI	Both	60-64 years	Alcohol use	2021	3.37	4.32	2.48
**DR**	High SDI	Both	60-64 years	Diet low in vegetables	2021	2.16	4.43	-0.46
**DR**	High SDI	Both	60-64 years	Smoking	2021	6.01	7.20	4.81
**DR**	High SDI	Both	60-64 years	Chewing tobacco	2021	0.29	0.52	0.14
**DALYR**	High SDI	Both	60-64 years	Alcohol use	2021	98.66	126.78	72.65
**DALYR**	High SDI	Both	60-64 years	Smoking	2021	175.94	210.57	141.05
**DALYR**	High SDI	Both	60-64 years	Chewing tobacco	2021	8.34	15.12	4.22
**DALYR**	High SDI	Both	60-64 years	Diet low in vegetables	2021	63.09	129.94	-13.47
**DR**	High SDI	Female	60-64 years	Alcohol use	2021	0.63	0.85	0.43
**DR**	High SDI	Female	60-64 years	Diet low in vegetables	2021	0.70	1.43	-0.15
**DR**	High SDI	Female	60-64 years	Smoking	2021	1.18	1.47	0.88
**DR**	High SDI	Female	60-64 years	Chewing tobacco	2021	0.02	0.03	0.01
**DALYR**	High SDI	Female	60-64 years	Alcohol use	2021	18.60	24.98	12.61
**DALYR**	High SDI	Female	60-64 years	Smoking	2021	34.65	43.00	25.89

**DALYR**	High SDI	Female	60-64 years	Chewing tobacco	2021	0.57	1.01	0.29
**DALYR**	High SDI	Female	60-64 years	Diet low in vegetables	2021	20.38	41.95	-4.36
**DR**	High SDI	Male	60-64 years	Alcohol use	2021	6.23	7.95	4.58
**DR**	High SDI	Male	60-64 years	Diet low in vegetables	2021	3.68	7.50	-0.79
**DR**	High SDI	Male	60-64 years	Smoking	2021	11.06	13.28	8.86
**DR**	High SDI	Male	60-64 years	Chewing tobacco	2021	0.56	1.03	0.27
**DALYR**	High SDI	Male	60-64 years	Alcohol use	2021	182.32	232.97	134.28
**DALYR**	High SDI	Male	60-64 years	Smoking	2021	323.61	388.65	259.44
**DALYR**	High SDI	Male	60-64 years	Chewing tobacco	2021	16.45	30.22	7.96
**DALYR**	High SDI	Male	60-64 years	Diet low in vegetables	2021	107.72	219.73	-23.02
**DR**	High SDI	Both	65-74 years	Alcohol use	1990	5.42	7.18	3.72
**DR**	High SDI	Both	65-74 years	Smoking	1990	15.18	17.49	12.55
**DR**	High SDI	Both	65-74 years	Chewing tobacco	1990	0.44	0.68	0.27
**DR**	High SDI	Both	65-74 years	Diet low in vegetables	1990	5.04	10.12	-1.08
**DALYR**	High SDI	Both	65-74 years	Alcohol use	1990	123.03	162.82	84.62
**DALYR**	High SDI	Both	65-74 years	Smoking	1990	343.03	394.83	283.72
**DALYR**	High SDI	Both	65-74 years	Diet low in vegetables	1990	113.62	228.05	-24.26
**DALYR**	High SDI	Both	65-74 years	Chewing tobacco	1990	9.97	15.33	6.04
**DR**	High SDI	Female	65-74 years	Alcohol use	1990	1.13	1.53	0.75
**DR**	High SDI	Female	65-74 years	Smoking	1990	3.77	4.58	2.95
**DR**	High SDI	Female	65-74 years	Chewing tobacco	1990	0.04	0.07	0.02
**DR**	High SDI	Female	65-74 years	Diet low in vegetables	1990	2.01	4.05	-0.44
**DALYR**	High SDI	Female	65-74 years	Alcohol use	1990	25.38	34.54	16.77
**DALYR**	High SDI	Female	65-74 years	Smoking	1990	84.36	102.68	66.08
**DALYR**	High SDI	Female	65-74 years	Diet low in vegetables	1990	44.97	90.41	-9.81
**DALYR**	High SDI	Female	65-74 years	Chewing tobacco	1990	1.01	1.68	0.56
**DR**	High SDI	Male	65-74 years	Alcohol use	1990	11.08	14.69	7.62
**DR**	High SDI	Male	65-74 years	Smoking	1990	30.23	34.68	25.18
**DR**	High SDI	Male	65-74 years	Chewing tobacco	1990	0.97	1.51	0.56

**DR**	High SDI	Male	65-74 years	Diet low in vegetables	1990	9.04	18.28	-1.92
**DALYR**	High SDI	Male	65-74 years	Alcohol use	1990	251.86	333.92	172.57
**DALYR**	High SDI	Male	65-74 years	Smoking	1990	684.28	786.08	570.89
**DALYR**	High SDI	Male	65-74 years	Diet low in vegetables	1990	204.18	412.79	-43.32
**DALYR**	High SDI	Male	65-74 years	Chewing tobacco	1990	21.80	34.38	12.65
**DR**	High SDI	Both	65-74 years	Alcohol use	2021	4.92	6.29	3.63
**DR**	High SDI	Both	65-74 years	Smoking	2021	10.37	12.53	8.20
**DR**	High SDI	Both	65-74 years	Chewing tobacco	2021	0.39	0.63	0.20
**DR**	High SDI	Both	65-74 years	Diet low in vegetables	2021	3.48	7.09	-0.72
**DALYR**	High SDI	Both	65-74 years	Chewing tobacco	2021	8.78	13.94	4.45
**DALYR**	High SDI	Both	65-74 years	Smoking	2021	231.38	279.61	183.39
**DALYR**	High SDI	Both	65-74 years	Alcohol use	2021	109.98	141.13	81.41
**DALYR**	High SDI	Both	65-74 years	Diet low in vegetables	2021	77.56	157.50	-16.11
**DR**	High SDI	Female	65-74 years	Alcohol use	2021	0.90	1.19	0.62
**DR**	High SDI	Female	65-74 years	Smoking	2021	2.05	2.62	1.52
**DR**	High SDI	Female	65-74 years	Chewing tobacco	2021	0.03	0.05	0.02
**DR**	High SDI	Female	65-74 years	Diet low in vegetables	2021	1.17	2.40	-0.25
**DALYR**	High SDI	Female	65-74 years	Chewing tobacco	2021	0.73	1.24	0.39
**DALYR**	High SDI	Female	65-74 years	Smoking	2021	45.61	58.11	33.82
**DALYR**	High SDI	Female	65-74 years	Alcohol use	2021	20.13	26.52	13.79
**DALYR**	High SDI	Female	65-74 years	Diet low in vegetables	2021	26.04	53.40	-5.49
**DR**	High SDI	Male	65-74 years	Alcohol use	2021	9.36	11.94	6.92
**DR**	High SDI	Male	65-74 years	Smoking	2021	19.59	23.60	15.51
**DR**	High SDI	Male	65-74 years	Chewing tobacco	2021	0.79	1.28	0.39
**DR**	High SDI	Male	65-74 years	Diet low in vegetables	2021	6.04	12.35	-1.25
**DALYR**	High SDI	Male	65-74 years	Chewing tobacco	2021	17.69	28.40	8.77
**DALYR**	High SDI	Male	65-74 years	Smoking	2021	437.15	526.69	346.77
**DALYR**	High SDI	Male	65-74 years	Alcohol use	2021	209.50	267.39	155.28
**DALYR**	High SDI	Male	65-74 years	Diet low in vegetables	2021	134.63	274.70	-27.88

**DR**	High SDI	Both	75+ years	Alcohol use	1990	6.01	8.22	3.69
**DR**	High SDI	Both	75+ years	Smoking	1990	18.73	22.64	14.68
**DR**	High SDI	Both	75+ years	Chewing tobacco	1990	0.71	1.03	0.44
**DR**	High SDI	Both	75+ years	Diet low in vegetables	1990	7.92	15.90	-1.76
**DALYR**	High SDI	Both	75+ years	Chewing tobacco	1990	9.35	13.78	5.77
**DALYR**	High SDI	Both	75+ years	Smoking	1990	255.98	308.26	201.80
**DALYR**	High SDI	Both	75+ years	Alcohol use	1990	82.47	112.64	51.33
**DALYR**	High SDI	Both	75+ years	Diet low in vegetables	1990	106.14	212.99	-23.44
**DR**	High SDI	Female	75+ years	Alcohol use	1990	2.19	3.14	1.22
**DR**	High SDI	Female	75+ years	Smoking	1990	6.96	9.16	4.94
**DR**	High SDI	Female	75+ years	Chewing tobacco	1990	0.10	0.18	0.05
**DR**	High SDI	Female	75+ years	Diet low in vegetables	1990	4.95	9.95	-1.12
**DALYR**	High SDI	Female	75+ years	Chewing tobacco	1990	1.30	2.24	0.69
**DALYR**	High SDI	Female	75+ years	Smoking	1990	89.82	117.40	64.28
**DALYR**	High SDI	Female	75+ years	Alcohol use	1990	28.36	40.41	16.49
**DALYR**	High SDI	Female	75+ years	Diet low in vegetables	1990	63.53	127.40	-14.30
**DR**	High SDI	Male	75+ years	Alcohol use	1990	13.17	17.76	8.39
**DR**	High SDI	Male	75+ years	Smoking	1990	40.76	47.93	33.03
**DR**	High SDI	Male	75+ years	Chewing tobacco	1990	1.84	2.78	1.09
**DR**	High SDI	Male	75+ years	Diet low in vegetables	1990	13.47	27.03	-2.96
**DALYR**	High SDI	Male	75+ years	Chewing tobacco	1990	24.43	37.27	14.51
**DALYR**	High SDI	Male	75+ years	Smoking	1990	567.03	667.91	460.37
**DALYR**	High SDI	Male	75+ years	Alcohol use	1990	183.76	247.77	117.41
**DALYR**	High SDI	Male	75+ years	Diet low in vegetables	1990	185.92	372.63	-40.55
**DR**	High SDI	Both	75+ years	Alcohol use	2021	6.67	8.82	4.56
**DR**	High SDI	Both	75+ years	Smoking	2021	16.32	20.60	12.12
**DR**	High SDI	Both	75+ years	Chewing tobacco	2021	0.63	0.99	0.35
**DR**	High SDI	Both	75+ years	Diet low in vegetables	2021	6.90	14.25	-1.55
**DALYR**	High SDI	Both	75+ years	Smoking	2021	211.12	264.64	159.05

**DALYR**	High SDI	Both	75+ years	Chewing tobacco	2021	7.94	12.47	4.48
**DALYR**	High SDI	Both	75+ years	Alcohol use	2021	86.74	114.05	60.23
**DALYR**	High SDI	Both	75+ years	Diet low in vegetables	2021	86.47	178.01	-19.39
**DR**	High SDI	Female	75+ years	Alcohol use	2021	1.91	2.64	1.20
**DR**	High SDI	Female	75+ years	Smoking	2021	4.66	6.43	3.17
**DR**	High SDI	Female	75+ years	Chewing tobacco	2021	0.09	0.15	0.04
**DR**	High SDI	Female	75+ years	Diet low in vegetables	2021	3.63	7.61	-0.81
**DALYR**	High SDI	Female	75+ years	Smoking	2021	56.49	77.06	38.71
**DALYR**	High SDI	Female	75+ years	Chewing tobacco	2021	1.02	1.78	0.53
**DALYR**	High SDI	Female	75+ years	Alcohol use	2021	23.16	31.81	14.82
**DALYR**	High SDI	Female	75+ years	Diet low in vegetables	2021	42.93	89.45	-9.53
**DR**	High SDI	Male	75+ years	Alcohol use	2021	13.52	17.83	9.43
**DR**	High SDI	Male	75+ years	Smoking	2021	33.08	41.28	24.80
**DR**	High SDI	Male	75+ years	Chewing tobacco	2021	1.40	2.29	0.74
**DR**	High SDI	Male	75+ years	Diet low in vegetables	2021	11.61	24.07	-2.63
**DALYR**	High SDI	Male	75+ years	Smoking	2021	433.51	537.11	329.46
**DALYR**	High SDI	Male	75+ years	Chewing tobacco	2021	17.88	28.88	9.58
**DALYR**	High SDI	Male	75+ years	Alcohol use	2021	178.18	233.40	125.21
**DALYR**	High SDI	Male	75+ years	Diet low in vegetables	2021	149.09	308.10	-33.58
**DR**	High-middle SDI	Both	20-54 years	Diet low in vegetables	1990	0.81	1.61	-0.17
**DR**	High-middle SDI	Both	20-54 years	Smoking	1990	1.55	1.95	1.22
**DR**	High-middle SDI	Both	20-54 years	Chewing tobacco	1990	0.05	0.09	0.03
**DR**	High-middle SDI	Both	20-54 years	Alcohol use	1990	0.96	1.26	0.69
**DALYR**	High-middle SDI	Both	20-54 years	Alcohol use	1990	41.49	54.38	30.02
**DALYR**	High-middle SDI	Both	20-54 years	Smoking	1990	65.33	81.83	51.25
**DALYR**	High-middle SDI	Both	20-54 years	Chewing tobacco	1990	2.31	3.79	1.14
**DALYR**	High-middle SDI	Both	20-54 years	Diet low in vegetables	1990	34.31	68.96	-7.18
**DR**	High-middle SDI	Female	20-54 years	Diet low in vegetables	1990	0.24	0.53	-0.05
**DR**	High-middle SDI	Female	20-54 years	Smoking	1990	0.07	0.10	0.05

**DR**	High-middle SDI	Female	20-54 years	Chewing tobacco	1990	0.01	0.02	0.00
**DR**	High-middle SDI	Female	20-54 years	Alcohol use	1990	0.06	0.09	0.04
**DALYR**	High-middle SDI	Female	20-54 years	Alcohol use	1990	2.75	3.91	1.79
**DALYR**	High-middle SDI	Female	20-54 years	Smoking	1990	2.94	4.01	2.09
**DALYR**	High-middle SDI	Female	20-54 years	Chewing tobacco	1990	0.34	0.63	0.15
**DALYR**	High-middle SDI	Female	20-54 years	Diet low in vegetables	1990	10.36	22.33	-1.99
**DR**	High-middle SDI	Male	20-54 years	Diet low in vegetables	1990	1.35	2.72	-0.28
**DR**	High-middle SDI	Male	20-54 years	Smoking	1990	2.99	3.78	2.34
**DR**	High-middle SDI	Male	20-54 years	Chewing tobacco	1990	0.10	0.16	0.04
**DR**	High-middle SDI	Male	20-54 years	Alcohol use	1990	1.84	2.42	1.32
**DALYR**	High-middle SDI	Male	20-54 years	Alcohol use	1990	79.27	104.27	57.15
**DALYR**	High-middle SDI	Male	20-54 years	Smoking	1990	126.17	158.70	98.78
**DALYR**	High-middle SDI	Male	20-54 years	Chewing tobacco	1990	4.23	7.05	1.91
**DALYR**	High-middle SDI	Male	20-54 years	Diet low in vegetables	1990	57.66	116.09	-12.00
**DR**	High-middle SDI	Both	20-54 years	Diet low in vegetables	2021	0.11	0.24	-0.02
**DR**	High-middle SDI	Both	20-54 years	Smoking	2021	1.11	1.49	0.82
**DR**	High-middle SDI	Both	20-54 years	Chewing tobacco	2021	0.05	0.08	0.02
**DR**	High-middle SDI	Both	20-54 years	Alcohol use	2021	0.71	0.97	0.50
**DALYR**	High-middle SDI	Both	20-54 years	Alcohol use	2021	29.61	40.97	21.05
**DALYR**	High-middle SDI	Both	20-54 years	Smoking	2021	45.67	60.96	33.69
**DALYR**	High-middle SDI	Both	20-54 years	Chewing tobacco	2021	1.97	3.50	0.98
**DALYR**	High-middle SDI	Both	20-54 years	Diet low in vegetables	2021	4.47	10.28	-0.92
**DR**	High-middle SDI	Female	20-54 years	Diet low in vegetables	2021	0.03	0.07	-0.01
**DR**	High-middle SDI	Female	20-54 years	Smoking	2021	0.04	0.05	0.03
**DR**	High-middle SDI	Female	20-54 years	Chewing tobacco	2021	0.01	0.01	0.00
**DR**	High-middle SDI	Female	20-54 years	Alcohol use	2021	0.03	0.04	0.02
**DALYR**	High-middle SDI	Female	20-54 years	Alcohol use	2021	1.30	1.87	0.88
**DALYR**	High-middle SDI	Female	20-54 years	Smoking	2021	1.56	2.07	1.15
**DALYR**	High-middle SDI	Female	20-54 years	Chewing tobacco	2021	0.21	0.38	0.10

**DALYR**	High-middle SDI	Female	20-54 years	Diet low in vegetables	2021	1.34	2.94	-0.27
**DR**	High-middle SDI	Male	20-54 years	Diet low in vegetables	2021	0.18	0.41	-0.04
**DR**	High-middle SDI	Male	20-54 years	Smoking	2021	2.13	2.86	1.57
**DR**	High-middle SDI	Male	20-54 years	Chewing tobacco	2021	0.09	0.16	0.04
**DR**	High-middle SDI	Male	20-54 years	Alcohol use	2021	1.35	1.86	0.95
**DALYR**	High-middle SDI	Male	20-54 years	Alcohol use	2021	56.60	78.25	40.09
**DALYR**	High-middle SDI	Male	20-54 years	Smoking	2021	87.72	117.42	64.70
**DALYR**	High-middle SDI	Male	20-54 years	Chewing tobacco	2021	3.64	6.64	1.72
**DALYR**	High-middle SDI	Male	20-54 years	Diet low in vegetables	2021	7.45	17.36	-1.54
**DR**	High-middle SDI	Both	55-59 years	Diet low in vegetables	1990	6.46	13.03	-1.45
**DR**	High-middle SDI	Both	55-59 years	Smoking	1990	13.77	16.76	10.92
**DR**	High-middle SDI	Both	55-59 years	Chewing tobacco	1990	0.26	0.51	0.11
**DR**	High-middle SDI	Both	55-59 years	Alcohol use	1990	6.80	8.99	4.91
**DALYR**	High-middle SDI	Both	55-59 years	Diet low in vegetables	1990	218.41	440.09	-48.84
**DALYR**	High-middle SDI	Both	55-59 years	Smoking	1990	465.04	566.15	368.98
**DALYR**	High-middle SDI	Both	55-59 years	Alcohol use	1990	229.80	303.54	165.74
**DALYR**	High-middle SDI	Both	55-59 years	Chewing tobacco	1990	8.71	17.14	3.88
**DR**	High-middle SDI	Female	55-59 years	Diet low in vegetables	1990	2.45	5.17	-0.50
**DR**	High-middle SDI	Female	55-59 years	Smoking	1990	1.01	1.50	0.61
**DR**	High-middle SDI	Female	55-59 years	Chewing tobacco	1990	0.08	0.18	0.03
**DR**	High-middle SDI	Female	55-59 years	Alcohol use	1990	0.52	0.79	0.32
**DALYR**	High-middle SDI	Female	55-59 years	Diet low in vegetables	1990	82.61	174.67	-16.76
**DALYR**	High-middle SDI	Female	55-59 years	Smoking	1990	33.98	50.51	20.49
**DALYR**	High-middle SDI	Female	55-59 years	Alcohol use	1990	17.61	26.60	10.76
**DALYR**	High-middle SDI	Female	55-59 years	Chewing tobacco	1990	2.60	6.19	0.85
**DR**	High-middle SDI	Male	55-59 years	Diet low in vegetables	1990	10.69	21.82	-2.40
**DR**	High-middle SDI	Male	55-59 years	Smoking	1990	27.17	33.33	21.44
**DR**	High-middle SDI	Male	55-59 years	Chewing tobacco	1990	0.45	0.94	0.17
**DR**	High-middle SDI	Male	55-59 years	Alcohol use	1990	13.40	17.75	9.63

**DALYR**	High-middle SDI	Male	55-59 years	Diet low in vegetables	1990	361.04	737.05	-81.18
**DALYR**	High-middle SDI	Male	55-59 years	Smoking	1990	917.78	1125.89	724.38
**DALYR**	High-middle SDI	Male	55-59 years	Alcohol use	1990	452.66	599.10	325.12
**DALYR**	High-middle SDI	Male	55-59 years	Chewing tobacco	1990	15.13	31.64	5.72
**DR**	High-middle SDI	Both	55-59 years	Diet low in vegetables	2021	0.71	1.99	-0.14
**DR**	High-middle SDI	Both	55-59 years	Smoking	2021	8.65	11.79	6.34
**DR**	High-middle SDI	Both	55-59 years	Chewing tobacco	2021	0.20	0.41	0.08
**DR**	High-middle SDI	Both	55-59 years	Alcohol use	2021	4.30	6.20	3.00
**DALYR**	High-middle SDI	Both	55-59 years	Diet low in vegetables	2021	24.17	67.36	-4.62
**DALYR**	High-middle SDI	Both	55-59 years	Smoking	2021	293.04	399.11	214.93
**DALYR**	High-middle SDI	Both	55-59 years	Alcohol use	2021	145.66	209.77	101.43
**DALYR**	High-middle SDI	Both	55-59 years	Chewing tobacco	2021	6.61	13.88	2.85
**DR**	High-middle SDI	Female	55-59 years	Diet low in vegetables	2021	0.19	0.44	-0.04
**DR**	High-middle SDI	Female	55-59 years	Smoking	2021	0.34	0.51	0.22
**DR**	High-middle SDI	Female	55-59 years	Chewing tobacco	2021	0.04	0.07	0.02
**DR**	High-middle SDI	Female	55-59 years	Alcohol use	2021	0.18	0.28	0.11
**DALYR**	High-middle SDI	Female	55-59 years	Diet low in vegetables	2021	6.51	15.05	-1.24
**DALYR**	High-middle SDI	Female	55-59 years	Smoking	2021	11.39	17.34	7.53
**DALYR**	High-middle SDI	Female	55-59 years	Alcohol use	2021	6.25	9.41	3.86
**DALYR**	High-middle SDI	Female	55-59 years	Chewing tobacco	2021	1.26	2.44	0.52
**DR**	High-middle SDI	Male	55-59 years	Diet low in vegetables	2021	1.26	3.47	-0.24
**DR**	High-middle SDI	Male	55-59 years	Smoking	2021	17.30	23.58	12.61
**DR**	High-middle SDI	Male	55-59 years	Chewing tobacco	2021	0.36	0.81	0.14
**DR**	High-middle SDI	Male	55-59 years	Alcohol use	2021	8.58	12.44	5.92
**DALYR**	High-middle SDI	Male	55-59 years	Diet low in vegetables	2021	42.55	117.21	-8.09
**DALYR**	High-middle SDI	Male	55-59 years	Smoking	2021	586.27	798.42	427.89
**DALYR**	High-middle SDI	Male	55-59 years	Alcohol use	2021	290.81	420.82	200.36
**DALYR**	High-middle SDI	Male	55-59 years	Chewing tobacco	2021	12.19	27.45	4.59
**DR**	High-middle SDI	Both	60-64 years	Diet low in vegetables	1990	8.25	16.54	-1.82

**DR**	High-middle SDI	Both	60-64 years	Smoking	1990	18.28	22.61	14.44
**DR**	High-middle SDI	Both	60-64 years	Chewing tobacco	1990	0.30	0.58	0.13
**DR**	High-middle SDI	Both	60-64 years	Alcohol use	1990	7.97	10.64	5.71
**DALYR**	High-middle SDI	Both	60-64 years	Diet low in vegetables	1990	240.47	482.82	-53.17
**DALYR**	High-middle SDI	Both	60-64 years	Smoking	1990	533.16	659.11	421.13
**DALYR**	High-middle SDI	Both	60-64 years	Alcohol use	1990	232.50	310.11	166.43
**DALYR**	High-middle SDI	Both	60-64 years	Chewing tobacco	1990	8.65	16.90	3.85
**DR**	High-middle SDI	Female	60-64 years	Diet low in vegetables	1990	3.58	7.70	-0.72
**DR**	High-middle SDI	Female	60-64 years	Smoking	1990	1.68	2.50	1.02
**DR**	High-middle SDI	Female	60-64 years	Chewing tobacco	1990	0.10	0.24	0.04
**DR**	High-middle SDI	Female	60-64 years	Alcohol use	1990	0.82	1.22	0.49
**DALYR**	High-middle SDI	Female	60-64 years	Diet low in vegetables	1990	104.38	224.15	-21.02
**DALYR**	High-middle SDI	Female	60-64 years	Smoking	1990	49.02	72.87	29.70
**DALYR**	High-middle SDI	Female	60-64 years	Alcohol use	1990	23.72	35.47	14.24
**DALYR**	High-middle SDI	Female	60-64 years	Chewing tobacco	1990	2.86	6.92	1.02
**DR**	High-middle SDI	Male	60-64 years	Diet low in vegetables	1990	13.63	28.08	-2.99
**DR**	High-middle SDI	Male	60-64 years	Smoking	1990	37.45	46.67	29.39
**DR**	High-middle SDI	Male	60-64 years	Chewing tobacco	1990	0.53	1.16	0.20
**DR**	High-middle SDI	Male	60-64 years	Alcohol use	1990	16.24	21.78	11.42
**DALYR**	High-middle SDI	Male	60-64 years	Diet low in vegetables	1990	397.64	818.68	-87.26
**DALYR**	High-middle SDI	Male	60-64 years	Smoking	1990	1092.34	1360.82	857.16
**DALYR**	High-middle SDI	Male	60-64 years	Alcohol use	1990	473.62	635.34	333.24
**DALYR**	High-middle SDI	Male	60-64 years	Chewing tobacco	1990	15.32	33.94	5.81
**DR**	High-middle SDI	Both	60-64 years	Diet low in vegetables	2021	1.07	2.55	-0.17
**DR**	High-middle SDI	Both	60-64 years	Smoking	2021	13.01	17.38	9.57
**DR**	High-middle SDI	Both	60-64 years	Chewing tobacco	2021	0.25	0.48	0.12
**DR**	High-middle SDI	Both	60-64 years	Alcohol use	2021	5.65	7.92	3.88
**DALYR**	High-middle SDI	Both	60-64 years	Diet low in vegetables	2021	31.27	74.42	-5.08
**DALYR**	High-middle SDI	Both	60-64 years	Alcohol use	2021	164.72	230.54	113.12

**DALYR**	High-middle SDI	Both	60-64 years	Smoking	2021	378.94	506.65	278.70
**DALYR**	High-middle SDI	Both	60-64 years	Chewing tobacco	2021	7.35	13.88	3.38
**DR**	High-middle SDI	Female	60-64 years	Diet low in vegetables	2021	0.31	0.72	-0.06
**DR**	High-middle SDI	Female	60-64 years	Smoking	2021	0.58	0.93	0.36
**DR**	High-middle SDI	Female	60-64 years	Chewing tobacco	2021	0.06	0.12	0.02
**DR**	High-middle SDI	Female	60-64 years	Alcohol use	2021	0.32	0.50	0.19
**DALYR**	High-middle SDI	Female	60-64 years	Diet low in vegetables	2021	9.07	21.16	-1.73
**DALYR**	High-middle SDI	Female	60-64 years	Alcohol use	2021	9.21	14.52	5.55
**DALYR**	High-middle SDI	Female	60-64 years	Smoking	2021	16.97	27.26	10.63
**DALYR**	High-middle SDI	Female	60-64 years	Chewing tobacco	2021	1.66	3.39	0.70
**DR**	High-middle SDI	Male	60-64 years	Diet low in vegetables	2021	1.90	4.55	-0.31
**DR**	High-middle SDI	Male	60-64 years	Smoking	2021	26.51	35.59	19.33
**DR**	High-middle SDI	Male	60-64 years	Chewing tobacco	2021	0.46	0.94	0.19
**DR**	High-middle SDI	Male	60-64 years	Alcohol use	2021	11.45	16.13	7.84
**DALYR**	High-middle SDI	Male	60-64 years	Diet low in vegetables	2021	55.41	132.52	-9.07
**DALYR**	High-middle SDI	Male	60-64 years	Alcohol use	2021	333.73	469.66	228.20
**DALYR**	High-middle SDI	Male	60-64 years	Smoking	2021	772.36	1035.60	563.81
**DALYR**	High-middle SDI	Male	60-64 years	Chewing tobacco	2021	13.52	27.36	5.63
**DR**	High-middle SDI	Both	65-74 years	Diet low in vegetables	1990	13.12	26.54	-2.75
**DR**	High-middle SDI	Both	65-74 years	Smoking	1990	28.54	34.90	22.59
**DR**	High-middle SDI	Both	65-74 years	Alcohol use	1990	9.83	13.02	6.83
**DR**	High-middle SDI	Both	65-74 years	Chewing tobacco	1990	0.38	0.63	0.22
**DALYR**	High-middle SDI	Both	65-74 years	Diet low in vegetables	1990	294.54	596.05	-62.01
**DALYR**	High-middle SDI	Both	65-74 years	Alcohol use	1990	222.93	295.51	154.59
**DALYR**	High-middle SDI	Both	65-74 years	Smoking	1990	642.30	784.22	508.59
**DALYR**	High-middle SDI	Both	65-74 years	Chewing tobacco	1990	8.50	14.37	4.90
**DR**	High-middle SDI	Female	65-74 years	Diet low in vegetables	1990	6.85	14.19	-1.31
**DR**	High-middle SDI	Female	65-74 years	Smoking	1990	4.03	5.89	2.41
**DR**	High-middle SDI	Female	65-74 years	Alcohol use	1990	1.22	1.85	0.75

**DR**	High-middle SDI	Female	65-74 years	Chewing tobacco	1990	0.16	0.31	0.07
**DALYR**	High-middle SDI	Female	65-74 years	Diet low in vegetables	1990	152.36	315.41	-29.02
**DALYR**	High-middle SDI	Female	65-74 years	Alcohol use	1990	27.28	41.41	16.88
**DALYR**	High-middle SDI	Female	65-74 years	Smoking	1990	88.37	128.66	52.79
**DALYR**	High-middle SDI	Female	65-74 years	Chewing tobacco	1990	3.64	6.98	1.59
**DR**	High-middle SDI	Male	65-74 years	Diet low in vegetables	1990	21.71	44.37	-4.86
**DR**	High-middle SDI	Male	65-74 years	Smoking	1990	62.14	76.56	48.89
**DR**	High-middle SDI	Male	65-74 years	Alcohol use	1990	21.62	28.91	14.93
**DR**	High-middle SDI	Male	65-74 years	Chewing tobacco	1990	0.68	1.24	0.33
**DALYR**	High-middle SDI	Male	65-74 years	Diet low in vegetables	1990	489.41	1003.92	-109.07
**DALYR**	High-middle SDI	Male	65-74 years	Alcohol use	1990	491.08	656.44	338.81
**DALYR**	High-middle SDI	Male	65-74 years	Smoking	1990	1401.52	1723.17	1102.82
**DALYR**	High-middle SDI	Male	65-74 years	Chewing tobacco	1990	15.17	27.86	7.24
**DR**	High-middle SDI	Both	65-74 years	Diet low in vegetables	2021	1.61	3.83	-0.28
**DR**	High-middle SDI	Both	65-74 years	Smoking	2021	22.71	30.21	16.87
**DR**	High-middle SDI	Both	65-74 years	Alcohol use	2021	7.86	11.08	5.40
**DR**	High-middle SDI	Both	65-74 years	Chewing tobacco	2021	0.34	0.59	0.18
**DALYR**	High-middle SDI	Both	65-74 years	Alcohol use	2021	177.01	249.25	121.29
**DALYR**	High-middle SDI	Both	65-74 years	Diet low in vegetables	2021	36.16	86.36	-6.21
**DALYR**	High-middle SDI	Both	65-74 years	Chewing tobacco	2021	7.68	13.31	3.95
**DALYR**	High-middle SDI	Both	65-74 years	Smoking	2021	508.74	675.41	376.41
**DR**	High-middle SDI	Female	65-74 years	Diet low in vegetables	2021	0.58	1.43	-0.11
**DR**	High-middle SDI	Female	65-74 years	Smoking	2021	1.62	2.56	0.97
**DR**	High-middle SDI	Female	65-74 years	Alcohol use	2021	0.53	0.83	0.30
**DR**	High-middle SDI	Female	65-74 years	Chewing tobacco	2021	0.11	0.23	0.05
**DALYR**	High-middle SDI	Female	65-74 years	Alcohol use	2021	11.81	18.47	6.68
**DALYR**	High-middle SDI	Female	65-74 years	Diet low in vegetables	2021	12.95	31.56	-2.54
**DALYR**	High-middle SDI	Female	65-74 years	Chewing tobacco	2021	2.55	5.23	1.11
**DALYR**	High-middle SDI	Female	65-74 years	Smoking	2021	35.77	56.00	21.36

**DR**	High-middle SDI	Male	65-74 years	Diet low in vegetables	2021	2.82	6.89	-0.49
**DR**	High-middle SDI	Male	65-74 years	Smoking	2021	47.57	63.81	35.21
**DR**	High-middle SDI	Male	65-74 years	Alcohol use	2021	16.49	23.42	11.21
**DR**	High-middle SDI	Male	65-74 years	Chewing tobacco	2021	0.62	1.16	0.27
**DALYR**	High-middle SDI	Male	65-74 years	Alcohol use	2021	371.68	527.27	251.36
**DALYR**	High-middle SDI	Male	65-74 years	Diet low in vegetables	2021	63.52	155.37	-10.86
**DALYR**	High-middle SDI	Male	65-74 years	Chewing tobacco	2021	13.72	25.64	5.90
**DALYR**	High-middle SDI	Male	65-74 years	Smoking	2021	1066.12	1433.32	786.98
**DR**	High-middle SDI	Both	75+ years	Diet low in vegetables	1990	15.73	31.95	-3.34
**DR**	High-middle SDI	Both	75+ years	Smoking	1990	28.87	35.20	22.27
**DR**	High-middle SDI	Both	75+ years	Alcohol use	1990	8.24	11.05	5.71
**DR**	High-middle SDI	Both	75+ years	Chewing tobacco	1990	0.35	0.53	0.20
**DALYR**	High-middle SDI	Both	75+ years	Alcohol use	1990	118.57	160.24	81.74
**DALYR**	High-middle SDI	Both	75+ years	Diet low in vegetables	1990	221.76	450.67	-47.25
**DALYR**	High-middle SDI	Both	75+ years	Chewing tobacco	1990	4.84	7.62	2.75
**DALYR**	High-middle SDI	Both	75+ years	Smoking	1990	410.97	500.74	316.21
**DR**	High-middle SDI	Female	75+ years	Diet low in vegetables	1990	10.26	21.47	-1.99
**DR**	High-middle SDI	Female	75+ years	Smoking	1990	6.11	9.16	3.57
**DR**	High-middle SDI	Female	75+ years	Alcohol use	1990	1.70	2.58	1.04
**DR**	High-middle SDI	Female	75+ years	Chewing tobacco	1990	0.21	0.39	0.10
**DALYR**	High-middle SDI	Female	75+ years	Alcohol use	1990	23.40	35.32	14.34
**DALYR**	High-middle SDI	Female	75+ years	Diet low in vegetables	1990	141.83	296.71	-27.33
**DALYR**	High-middle SDI	Female	75+ years	Chewing tobacco	1990	2.91	5.24	1.39
**DALYR**	High-middle SDI	Female	75+ years	Smoking	1990	81.88	123.41	47.95
**DR**	High-middle SDI	Male	75+ years	Diet low in vegetables	1990	26.20	52.84	-5.65
**DR**	High-middle SDI	Male	75+ years	Smoking	1990	72.41	88.73	55.61
**DR**	High-middle SDI	Male	75+ years	Alcohol use	1990	20.74	27.89	14.44
**DR**	High-middle SDI	Male	75+ years	Chewing tobacco	1990	0.60	1.03	0.28
**DALYR**	High-middle SDI	Male	75+ years	Alcohol use	1990	300.67	400.16	209.37

**DALYR**	High-middle SDI	Male	75+ years	Diet low in vegetables	1990	374.72	759.26	-80.20
**DALYR**	High-middle SDI	Male	75+ years	Chewing tobacco	1990	8.54	15.07	4.00
**DALYR**	High-middle SDI	Male	75+ years	Smoking	1990	1040.73	1274.91	796.84
**DR**	High-middle SDI	Both	75+ years	Diet low in vegetables	2021	4.00	10.14	-0.84
**DR**	High-middle SDI	Both	75+ years	Smoking	2021	34.67	44.90	25.87
**DR**	High-middle SDI	Both	75+ years	Alcohol use	2021	9.36	12.88	6.13
**DR**	High-middle SDI	Both	75+ years	Chewing tobacco	2021	0.44	0.74	0.23
**DALYR**	High-middle SDI	Both	75+ years	Alcohol use	2021	127.14	175.56	82.78
**DALYR**	High-middle SDI	Both	75+ years	Diet low in vegetables	2021	49.68	127.37	-10.86
**DALYR**	High-middle SDI	Both	75+ years	Smoking	2021	463.75	605.25	345.78
**DALYR**	High-middle SDI	Both	75+ years	Chewing tobacco	2021	5.88	9.97	3.15
**DR**	High-middle SDI	Female	75+ years	Diet low in vegetables	2021	2.18	5.48	-0.42
**DR**	High-middle SDI	Female	75+ years	Smoking	2021	4.39	7.19	2.25
**DR**	High-middle SDI	Female	75+ years	Alcohol use	2021	1.00	1.54	0.57
**DR**	High-middle SDI	Female	75+ years	Chewing tobacco	2021	0.22	0.48	0.09
**DALYR**	High-middle SDI	Female	75+ years	Alcohol use	2021	12.80	19.82	7.38
**DALYR**	High-middle SDI	Female	75+ years	Diet low in vegetables	2021	26.20	64.57	-5.02
**DALYR**	High-middle SDI	Female	75+ years	Smoking	2021	55.29	90.85	28.48
**DALYR**	High-middle SDI	Female	75+ years	Chewing tobacco	2021	2.84	6.12	1.16
**DR**	High-middle SDI	Male	75+ years	Diet low in vegetables	2021	6.82	17.50	-1.33
**DR**	High-middle SDI	Male	75+ years	Smoking	2021	81.68	106.40	61.02
**DR**	High-middle SDI	Male	75+ years	Alcohol use	2021	22.33	31.00	14.59
**DR**	High-middle SDI	Male	75+ years	Chewing tobacco	2021	0.79	1.43	0.37
**DALYR**	High-middle SDI	Male	75+ years	Alcohol use	2021	304.65	420.92	199.95
**DALYR**	High-middle SDI	Male	75+ years	Diet low in vegetables	2021	86.13	219.94	-17.43
**DALYR**	High-middle SDI	Male	75+ years	Smoking	2021	1097.85	1442.82	819.80
**DALYR**	High-middle SDI	Male	75+ years	Chewing tobacco	2021	10.59	19.36	5.03
**DR**	Low SDI	Both	20-54 years	Alcohol use	1990	0.15	0.22	0.06
**DR**	Low SDI	Both	20-54 years	Smoking	1990	0.20	0.24	0.15

**DR**	Low SDI	Both	20-54 years	Chewing tobacco	1990	0.15	0.20	0.11
**DR**	Low SDI	Both	20-54 years	Diet low in vegetables	1990	0.52	1.00	-0.12
**DALYR**	Low SDI	Both	20-54 years	Alcohol use	1990	6.61	9.43	2.62
**DALYR**	Low SDI	Both	20-54 years	Diet low in vegetables	1990	22.26	42.91	-5.09
**DALYR**	Low SDI	Both	20-54 years	Smoking	1990	8.23	10.19	6.33
**DALYR**	Low SDI	Both	20-54 years	Chewing tobacco	1990	6.34	8.60	4.50
**DR**	Low SDI	Female	20-54 years	Alcohol use	1990	0.06	0.09	0.02
**DR**	Low SDI	Female	20-54 years	Smoking	1990	0.05	0.07	0.03
**DR**	Low SDI	Female	20-54 years	Chewing tobacco	1990	0.10	0.16	0.05
**DR**	Low SDI	Female	20-54 years	Diet low in vegetables	1990	0.45	0.88	-0.09
**DALYR**	Low SDI	Female	20-54 years	Alcohol use	1990	2.43	3.81	0.72
**DALYR**	Low SDI	Female	20-54 years	Diet low in vegetables	1990	19.43	38.35	-4.14
**DALYR**	Low SDI	Female	20-54 years	Smoking	1990	2.01	2.78	1.35
**DALYR**	Low SDI	Female	20-54 years	Chewing tobacco	1990	4.24	6.77	2.33
**DR**	Low SDI	Male	20-54 years	Alcohol use	1990	0.25	0.36	0.11
**DR**	Low SDI	Male	20-54 years	Smoking	1990	0.35	0.44	0.26
**DR**	Low SDI	Male	20-54 years	Chewing tobacco	1990	0.20	0.29	0.13
**DR**	Low SDI	Male	20-54 years	Diet low in vegetables	1990	0.59	1.15	-0.14
**DALYR**	Low SDI	Male	20-54 years	Alcohol use	1990	10.90	15.48	4.64
**DALYR**	Low SDI	Male	20-54 years	Diet low in vegetables	1990	25.16	48.92	-6.03
**DALYR**	Low SDI	Male	20-54 years	Smoking	1990	14.62	18.22	11.03
**DALYR**	Low SDI	Male	20-54 years	Chewing tobacco	1990	8.50	12.47	5.45
**DR**	Low SDI	Both	20-54 years	Alcohol use	2021	0.15	0.21	0.09
**DR**	Low SDI	Both	20-54 years	Smoking	2021	0.13	0.16	0.10
**DR**	Low SDI	Both	20-54 years	Chewing tobacco	2021	0.10	0.13	0.07
**DR**	Low SDI	Both	20-54 years	Diet low in vegetables	2021	0.39	0.77	-0.08
**DALYR**	Low SDI	Both	20-54 years	Alcohol use	2021	6.52	9.08	3.85
**DALYR**	Low SDI	Both	20-54 years	Diet low in vegetables	2021	16.96	33.33	-3.51
**DALYR**	Low SDI	Both	20-54 years	Smoking	2021	5.40	6.89	4.04

**DALYR**	Low SDI	Both	20-54 years	Chewing tobacco	2021	4.18	5.65	2.82
**DR**	Low SDI	Female	20-54 years	Alcohol use	2021	0.04	0.07	0.02
**DR**	Low SDI	Female	20-54 years	Smoking	2021	0.02	0.03	0.02
**DR**	Low SDI	Female	20-54 years	Chewing tobacco	2021	0.06	0.09	0.03
**DR**	Low SDI	Female	20-54 years	Diet low in vegetables	2021	0.30	0.59	-0.06
**DALYR**	Low SDI	Female	20-54 years	Alcohol use	2021	1.94	3.26	0.98
**DALYR**	Low SDI	Female	20-54 years	Diet low in vegetables	2021	13.09	26.00	-2.64
**DALYR**	Low SDI	Female	20-54 years	Smoking	2021	1.00	1.39	0.63
**DALYR**	Low SDI	Female	20-54 years	Chewing tobacco	2021	2.42	4.04	1.30
**DR**	Low SDI	Male	20-54 years	Alcohol use	2021	0.26	0.35	0.15
**DR**	Low SDI	Male	20-54 years	Smoking	2021	0.24	0.30	0.18
**DR**	Low SDI	Male	20-54 years	Chewing tobacco	2021	0.14	0.20	0.09
**DR**	Low SDI	Male	20-54 years	Diet low in vegetables	2021	0.49	0.97	-0.10
**DALYR**	Low SDI	Male	20-54 years	Alcohol use	2021	11.24	15.35	6.66
**DALYR**	Low SDI	Male	20-54 years	Diet low in vegetables	2021	20.94	41.66	-4.46
**DALYR**	Low SDI	Male	20-54 years	Smoking	2021	9.93	12.75	7.45
**DALYR**	Low SDI	Male	20-54 years	Chewing tobacco	2021	6.00	8.45	3.80
**DR**	Low SDI	Both	55-59 years	Alcohol use	1990	1.43	2.05	0.61
**DR**	Low SDI	Both	55-59 years	Diet low in vegetables	1990	5.16	10.08	-1.18
**DR**	Low SDI	Both	55-59 years	Smoking	1990	2.48	3.13	1.88
**DR**	Low SDI	Both	55-59 years	Chewing tobacco	1990	1.52	2.06	1.00
**DALYR**	Low SDI	Both	55-59 years	Chewing tobacco	1990	51.34	69.83	33.71
**DALYR**	Low SDI	Both	55-59 years	Smoking	1990	83.94	105.79	63.54
**DALYR**	Low SDI	Both	55-59 years	Alcohol use	1990	48.25	69.50	20.76
**DALYR**	Low SDI	Both	55-59 years	Diet low in vegetables	1990	174.63	341.27	-40.03
**DR**	Low SDI	Female	55-59 years	Alcohol use	1990	0.55	0.86	0.20
**DR**	Low SDI	Female	55-59 years	Diet low in vegetables	1990	4.64	9.11	-0.95
**DR**	Low SDI	Female	55-59 years	Smoking	1990	0.74	1.06	0.51
**DR**	Low SDI	Female	55-59 years	Chewing tobacco	1990	1.11	1.87	0.58

**DALYR**	Low SDI	Female	55-59 years	Chewing tobacco	1990	37.50	63.23	19.42
**DALYR**	Low SDI	Female	55-59 years	Smoking	1990	24.96	35.93	17.36
**DALYR**	Low SDI	Female	55-59 years	Alcohol use	1990	18.49	29.01	6.84
**DALYR**	Low SDI	Female	55-59 years	Diet low in vegetables	1990	156.86	308.13	-32.21
**DR**	Low SDI	Male	55-59 years	Alcohol use	1990	2.25	3.25	0.96
**DR**	Low SDI	Male	55-59 years	Diet low in vegetables	1990	5.66	11.19	-1.42
**DR**	Low SDI	Male	55-59 years	Smoking	1990	4.11	5.25	3.11
**DR**	Low SDI	Male	55-59 years	Chewing tobacco	1990	1.90	2.82	1.18
**DALYR**	Low SDI	Male	55-59 years	Chewing tobacco	1990	64.28	95.47	39.98
**DALYR**	Low SDI	Male	55-59 years	Smoking	1990	139.11	177.67	105.08
**DALYR**	Low SDI	Male	55-59 years	Alcohol use	1990	76.10	109.89	32.34
**DALYR**	Low SDI	Male	55-59 years	Diet low in vegetables	1990	191.24	378.32	-47.85
**DR**	Low SDI	Both	55-59 years	Alcohol use	2021	1.40	1.98	0.84
**DR**	Low SDI	Both	55-59 years	Diet low in vegetables	2021	3.95	7.69	-0.81
**DR**	Low SDI	Both	55-59 years	Smoking	2021	1.68	2.15	1.25
**DR**	Low SDI	Both	55-59 years	Chewing tobacco	2021	1.10	1.51	0.73
**DALYR**	Low SDI	Both	55-59 years	Chewing tobacco	2021	37.21	51.17	24.78
**DALYR**	Low SDI	Both	55-59 years	Smoking	2021	56.77	72.81	42.26
**DALYR**	Low SDI	Both	55-59 years	Alcohol use	2021	47.46	67.21	28.47
**DALYR**	Low SDI	Both	55-59 years	Diet low in vegetables	2021	133.65	260.68	-27.39
**DR**	Low SDI	Female	55-59 years	Alcohol use	2021	0.46	0.75	0.23
**DR**	Low SDI	Female	55-59 years	Diet low in vegetables	2021	3.18	6.34	-0.62
**DR**	Low SDI	Female	55-59 years	Smoking	2021	0.38	0.54	0.25
**DR**	Low SDI	Female	55-59 years	Chewing tobacco	2021	0.73	1.22	0.37
**DALYR**	Low SDI	Female	55-59 years	Chewing tobacco	2021	24.61	41.35	12.65
**DALYR**	Low SDI	Female	55-59 years	Smoking	2021	12.96	18.16	8.39
**DALYR**	Low SDI	Female	55-59 years	Alcohol use	2021	15.52	25.28	7.79
**DALYR**	Low SDI	Female	55-59 years	Diet low in vegetables	2021	107.66	214.71	-21.13
**DR**	Low SDI	Male	55-59 years	Alcohol use	2021	2.35	3.42	1.45

**DR**	Low SDI	Male	55-59 years	Diet low in vegetables	2021	4.72	9.42	-0.97
**DR**	Low SDI	Male	55-59 years	Smoking	2021	2.98	3.83	2.23
**DR**	Low SDI	Male	55-59 years	Chewing tobacco	2021	1.48	2.15	0.88
**DALYR**	Low SDI	Male	55-59 years	Chewing tobacco	2021	49.96	72.78	29.67
**DALYR**	Low SDI	Male	55-59 years	Smoking	2021	101.07	129.82	75.48
**DALYR**	Low SDI	Male	55-59 years	Alcohol use	2021	79.75	115.91	49.03
**DALYR**	Low SDI	Male	55-59 years	Diet low in vegetables	2021	159.93	319.30	-32.78
**DR**	Low SDI	Both	60-64 years	Alcohol use	1990	1.91	2.70	1.01
**DR**	Low SDI	Both	60-64 years	Diet low in vegetables	1990	7.21	13.88	-1.68
**DR**	Low SDI	Both	60-64 years	Smoking	1990	3.59	4.45	2.71
**DR**	Low SDI	Both	60-64 years	Chewing tobacco	1990	2.08	2.84	1.44
**DALYR**	Low SDI	Both	60-64 years	Chewing tobacco	1990	60.67	82.94	42.01
**DALYR**	Low SDI	Both	60-64 years	Smoking	1990	104.61	129.67	79.01
**DALYR**	Low SDI	Both	60-64 years	Alcohol use	1990	55.60	78.92	29.52
**DALYR**	Low SDI	Both	60-64 years	Diet low in vegetables	1990	210.13	403.64	-48.87
**DR**	Low SDI	Female	60-64 years	Alcohol use	1990	0.78	1.20	0.29
**DR**	Low SDI	Female	60-64 years	Diet low in vegetables	1990	6.46	12.10	-1.41
**DR**	Low SDI	Female	60-64 years	Smoking	1990	1.20	1.67	0.82
**DR**	Low SDI	Female	60-64 years	Chewing tobacco	1990	1.41	2.47	0.73
**DALYR**	Low SDI	Female	60-64 years	Chewing tobacco	1990	41.12	72.06	21.25
**DALYR**	Low SDI	Female	60-64 years	Smoking	1990	34.92	48.71	23.82
**DALYR**	Low SDI	Female	60-64 years	Alcohol use	1990	22.60	34.96	8.60
**DALYR**	Low SDI	Female	60-64 years	Diet low in vegetables	1990	188.28	352.92	-41.19
**DR**	Low SDI	Male	60-64 years	Alcohol use	1990	2.94	4.15	1.61
**DR**	Low SDI	Male	60-64 years	Diet low in vegetables	1990	7.89	15.56	-1.86
**DR**	Low SDI	Male	60-64 years	Smoking	1990	5.77	7.25	4.29
**DR**	Low SDI	Male	60-64 years	Chewing tobacco	1990	2.69	3.87	1.68
**DALYR**	Low SDI	Male	60-64 years	Chewing tobacco	1990	78.51	112.87	49.01
**DALYR**	Low SDI	Male	60-64 years	Smoking	1990	168.23	211.51	124.94

**DALYR**	Low SDI	Male	60-64 years	Alcohol use	1990	85.72	120.87	46.99
**DALYR**	Low SDI	Male	60-64 years	Diet low in vegetables	1990	230.07	453.57	-54.30
**DR**	Low SDI	Both	60-64 years	Alcohol use	2021	1.88	2.63	1.18
**DR**	Low SDI	Both	60-64 years	Diet low in vegetables	2021	5.54	10.80	-1.13
**DR**	Low SDI	Both	60-64 years	Smoking	2021	2.39	3.03	1.79
**DR**	Low SDI	Both	60-64 years	Chewing tobacco	2021	1.54	2.11	1.03
**DALYR**	Low SDI	Both	60-64 years	Chewing tobacco	2021	45.08	61.70	30.09
**DALYR**	Low SDI	Both	60-64 years	Smoking	2021	69.87	88.35	52.25
**DALYR**	Low SDI	Both	60-64 years	Diet low in vegetables	2021	161.76	315.42	-33.03
**DALYR**	Low SDI	Both	60-64 years	Alcohol use	2021	54.80	76.58	34.60
**DR**	Low SDI	Female	60-64 years	Alcohol use	2021	0.69	1.08	0.36
**DR**	Low SDI	Female	60-64 years	Diet low in vegetables	2021	4.54	9.27	-0.94
**DR**	Low SDI	Female	60-64 years	Smoking	2021	0.65	0.94	0.43
**DR**	Low SDI	Female	60-64 years	Chewing tobacco	2021	0.98	1.73	0.50
**DALYR**	Low SDI	Female	60-64 years	Chewing tobacco	2021	28.59	50.50	14.64
**DALYR**	Low SDI	Female	60-64 years	Smoking	2021	18.89	27.46	12.46
**DALYR**	Low SDI	Female	60-64 years	Diet low in vegetables	2021	132.42	270.93	-27.26
**DALYR**	Low SDI	Female	60-64 years	Alcohol use	2021	20.26	31.50	10.58
**DR**	Low SDI	Male	60-64 years	Alcohol use	2021	3.09	4.34	1.99
**DR**	Low SDI	Male	60-64 years	Diet low in vegetables	2021	6.57	12.70	-1.40
**DR**	Low SDI	Male	60-64 years	Smoking	2021	4.18	5.33	3.11
**DR**	Low SDI	Male	60-64 years	Chewing tobacco	2021	2.12	3.04	1.29
**DALYR**	Low SDI	Male	60-64 years	Chewing tobacco	2021	61.97	88.93	37.76
**DALYR**	Low SDI	Male	60-64 years	Smoking	2021	122.08	155.72	90.88
**DALYR**	Low SDI	Male	60-64 years	Diet low in vegetables	2021	191.80	371.52	-41.00
**DALYR**	Low SDI	Male	60-64 years	Alcohol use	2021	90.16	126.87	58.15
**DR**	Low SDI	Both	65-74 years	Alcohol use	1990	2.50	3.52	1.39
**DR**	Low SDI	Both	65-74 years	Smoking	1990	5.21	6.49	3.88
**DR**	Low SDI	Both	65-74 years	Chewing tobacco	1990	2.60	3.53	1.79

**DR**	Low SDI	Both	65-74 years	Diet low in vegetables	1990	10.60	20.36	-2.54
**DALYR**	Low SDI	Both	65-74 years	Chewing tobacco	1990	58.95	80.10	40.33
**DALYR**	Low SDI	Both	65-74 years	Smoking	1990	118.05	147.46	88.00
**DALYR**	Low SDI	Both	65-74 years	Alcohol use	1990	56.70	79.62	31.53
**DALYR**	Low SDI	Both	65-74 years	Diet low in vegetables	1990	239.24	460.83	-57.30
**DR**	Low SDI	Female	65-74 years	Alcohol use	1990	0.94	1.47	0.47
**DR**	Low SDI	Female	65-74 years	Smoking	1990	1.51	2.07	1.04
**DR**	Low SDI	Female	65-74 years	Chewing tobacco	1990	2.02	3.17	1.11
**DR**	Low SDI	Female	65-74 years	Diet low in vegetables	1990	9.24	17.70	-2.02
**DALYR**	Low SDI	Female	65-74 years	Chewing tobacco	1990	45.68	71.89	25.09
**DALYR**	Low SDI	Female	65-74 years	Smoking	1990	33.88	46.62	23.31
**DALYR**	Low SDI	Female	65-74 years	Alcohol use	1990	21.32	33.09	10.51
**DALYR**	Low SDI	Female	65-74 years	Diet low in vegetables	1990	208.16	399.13	-45.49
**DR**	Low SDI	Male	65-74 years	Alcohol use	1990	3.97	5.58	2.07
**DR**	Low SDI	Male	65-74 years	Smoking	1990	8.72	10.87	6.53
**DR**	Low SDI	Male	65-74 years	Chewing tobacco	1990	3.15	4.60	1.98
**DR**	Low SDI	Male	65-74 years	Diet low in vegetables	1990	11.88	23.43	-2.93
**DALYR**	Low SDI	Male	65-74 years	Chewing tobacco	1990	71.52	104.75	44.97
**DALYR**	Low SDI	Male	65-74 years	Smoking	1990	197.70	247.15	148.21
**DALYR**	Low SDI	Male	65-74 years	Alcohol use	1990	90.18	126.86	46.83
**DALYR**	Low SDI	Male	65-74 years	Diet low in vegetables	1990	268.64	528.95	-66.45
**DR**	Low SDI	Both	65-74 years	Alcohol use	2021	2.36	3.26	1.59
**DR**	Low SDI	Both	65-74 years	Smoking	2021	3.54	4.47	2.65
**DR**	Low SDI	Both	65-74 years	Chewing tobacco	2021	2.10	2.85	1.42
**DR**	Low SDI	Both	65-74 years	Diet low in vegetables	2021	8.17	15.69	-1.68
**DALYR**	Low SDI	Both	65-74 years	Alcohol use	2021	53.73	74.15	36.05
**DALYR**	Low SDI	Both	65-74 years	Smoking	2021	80.13	101.23	59.88
**DALYR**	Low SDI	Both	65-74 years	Chewing tobacco	2021	47.19	63.75	31.85
**DALYR**	Low SDI	Both	65-74 years	Diet low in vegetables	2021	184.62	354.62	-38.03

**DR**	Low SDI	Female	65-74 years	Alcohol use	2021	0.85	1.31	0.47
**DR**	Low SDI	Female	65-74 years	Smoking	2021	1.01	1.47	0.69
**DR**	Low SDI	Female	65-74 years	Chewing tobacco	2021	1.60	2.68	0.88
**DR**	Low SDI	Female	65-74 years	Diet low in vegetables	2021	7.03	13.66	-1.44
**DALYR**	Low SDI	Female	65-74 years	Alcohol use	2021	19.10	29.76	10.57
**DALYR**	Low SDI	Female	65-74 years	Smoking	2021	22.58	33.16	15.46
**DALYR**	Low SDI	Female	65-74 years	Chewing tobacco	2021	35.60	60.05	19.59
**DALYR**	Low SDI	Female	65-74 years	Diet low in vegetables	2021	157.94	307.69	-32.30
**DR**	Low SDI	Male	65-74 years	Alcohol use	2021	3.95	5.44	2.65
**DR**	Low SDI	Male	65-74 years	Smoking	2021	6.18	7.89	4.62
**DR**	Low SDI	Male	65-74 years	Chewing tobacco	2021	2.63	3.73	1.61
**DR**	Low SDI	Male	65-74 years	Diet low in vegetables	2021	9.37	18.29	-1.93
**DALYR**	Low SDI	Male	65-74 years	Alcohol use	2021	89.85	123.77	60.14
**DALYR**	Low SDI	Male	65-74 years	Smoking	2021	140.16	178.41	104.73
**DALYR**	Low SDI	Male	65-74 years	Chewing tobacco	2021	59.29	84.84	36.12
**DALYR**	Low SDI	Male	65-74 years	Diet low in vegetables	2021	212.45	414.15	-43.82
**DR**	Low SDI	Both	75+ years	Alcohol use	1990	2.08	2.89	1.13
**DR**	Low SDI	Both	75+ years	Smoking	1990	6.25	7.82	4.65
**DR**	Low SDI	Both	75+ years	Chewing tobacco	1990	3.32	4.43	2.19
**DR**	Low SDI	Both	75+ years	Diet low in vegetables	1990	12.89	24.43	-3.13
**DALYR**	Low SDI	Both	75+ years	Alcohol use	1990	30.72	42.63	16.79
**DALYR**	Low SDI	Both	75+ years	Smoking	1990	89.22	112.01	66.57
**DALYR**	Low SDI	Both	75+ years	Chewing tobacco	1990	46.68	62.99	30.93
**DALYR**	Low SDI	Both	75+ years	Diet low in vegetables	1990	184.07	349.54	-44.63
**DR**	Low SDI	Female	75+ years	Alcohol use	1990	0.90	1.40	0.43
**DR**	Low SDI	Female	75+ years	Smoking	1990	2.20	3.13	1.53
**DR**	Low SDI	Female	75+ years	Chewing tobacco	1990	3.20	5.04	1.72
**DR**	Low SDI	Female	75+ years	Diet low in vegetables	1990	12.42	24.42	-2.78
**DALYR**	Low SDI	Female	75+ years	Alcohol use	1990	12.88	20.17	6.16

**DALYR**	Low SDI	Female	75+ years	Smoking	1990	29.95	42.55	20.81
**DALYR**	Low SDI	Female	75+ years	Chewing tobacco	1990	44.48	70.62	23.85
**DALYR**	Low SDI	Female	75+ years	Diet low in vegetables	1990	174.43	343.86	-38.94
**DR**	Low SDI	Male	75+ years	Alcohol use	1990	3.33	4.66	1.80
**DR**	Low SDI	Male	75+ years	Smoking	1990	10.53	13.10	7.74
**DR**	Low SDI	Male	75+ years	Chewing tobacco	1990	3.44	4.98	2.06
**DR**	Low SDI	Male	75+ years	Diet low in vegetables	1990	13.39	26.20	-3.28
**DALYR**	Low SDI	Male	75+ years	Alcohol use	1990	49.59	69.30	26.86
**DALYR**	Low SDI	Male	75+ years	Smoking	1990	151.96	188.95	112.47
**DALYR**	Low SDI	Male	75+ years	Chewing tobacco	1990	49.00	71.66	29.08
**DALYR**	Low SDI	Male	75+ years	Diet low in vegetables	1990	194.27	380.70	-47.51
**DR**	Low SDI	Both	75+ years	Alcohol use	2021	2.21	3.05	1.41
**DR**	Low SDI	Both	75+ years	Smoking	2021	4.44	5.59	3.33
**DR**	Low SDI	Both	75+ years	Chewing tobacco	2021	3.12	4.20	2.10
**DR**	Low SDI	Both	75+ years	Diet low in vegetables	2021	11.46	22.20	-2.36
**DALYR**	Low SDI	Both	75+ years	Alcohol use	2021	31.31	43.12	19.90
**DALYR**	Low SDI	Both	75+ years	Smoking	2021	61.93	78.27	46.58
**DALYR**	Low SDI	Both	75+ years	Diet low in vegetables	2021	158.11	306.67	-32.53
**DALYR**	Low SDI	Both	75+ years	Chewing tobacco	2021	42.43	56.98	28.64
**DR**	Low SDI	Female	75+ years	Alcohol use	2021	0.98	1.57	0.52
**DR**	Low SDI	Female	75+ years	Smoking	2021	1.68	2.41	1.13
**DR**	Low SDI	Female	75+ years	Chewing tobacco	2021	2.93	4.62	1.57
**DR**	Low SDI	Female	75+ years	Diet low in vegetables	2021	11.38	22.08	-2.30
**DALYR**	Low SDI	Female	75+ years	Alcohol use	2021	13.46	21.09	6.97
**DALYR**	Low SDI	Female	75+ years	Smoking	2021	22.39	31.75	14.98
**DALYR**	Low SDI	Female	75+ years	Diet low in vegetables	2021	154.46	300.14	-31.20
**DALYR**	Low SDI	Female	75+ years	Chewing tobacco	2021	39.47	62.88	21.25
**DR**	Low SDI	Male	75+ years	Alcohol use	2021	3.59	4.98	2.28
**DR**	Low SDI	Male	75+ years	Smoking	2021	7.55	9.38	5.74

**DR**	Low SDI	Male	75+ years	Chewing tobacco	2021	3.33	4.86	1.99
**DR**	Low SDI	Male	75+ years	Diet low in vegetables	2021	11.55	22.58	-2.50
**DALYR**	Low SDI	Male	75+ years	Alcohol use	2021	51.50	71.57	32.77
**DALYR**	Low SDI	Male	75+ years	Smoking	2021	106.67	132.51	80.45
**DALYR**	Low SDI	Male	75+ years	Diet low in vegetables	2021	162.25	316.68	-35.04
**DALYR**	Low SDI	Male	75+ years	Chewing tobacco	2021	45.79	66.55	27.49
**DR**	Low-middle SDI	Both	20-54 years	Diet low in vegetables	1990	0.33	0.64	-0.08
**DR**	Low-middle SDI	Both	20-54 years	Alcohol use	1990	0.10	0.13	0.06
**DR**	Low-middle SDI	Both	20-54 years	Smoking	1990	0.25	0.30	0.20
**DR**	Low-middle SDI	Both	20-54 years	Chewing tobacco	1990	0.23	0.30	0.16
**DALYR**	Low-middle SDI	Both	20-54 years	Smoking	1990	10.46	12.64	8.28
**DALYR**	Low-middle SDI	Both	20-54 years	Diet low in vegetables	1990	14.17	27.82	-3.35
**DALYR**	Low-middle SDI	Both	20-54 years	Chewing tobacco	1990	9.91	12.98	7.05
**DALYR**	Low-middle SDI	Both	20-54 years	Alcohol use	1990	4.21	5.84	2.54
**DR**	Low-middle SDI	Female	20-54 years	Diet low in vegetables	1990	0.29	0.58	-0.07
**DR**	Low-middle SDI	Female	20-54 years	Alcohol use	1990	0.02	0.03	0.01
**DR**	Low-middle SDI	Female	20-54 years	Smoking	1990	0.04	0.06	0.03
**DR**	Low-middle SDI	Female	20-54 years	Chewing tobacco	1990	0.19	0.30	0.12
**DALYR**	Low-middle SDI	Female	20-54 years	Smoking	1990	1.84	2.55	1.28
**DALYR**	Low-middle SDI	Female	20-54 years	Diet low in vegetables	1990	12.87	25.61	-3.23
**DALYR**	Low-middle SDI	Female	20-54 years	Chewing tobacco	1990	8.07	12.68	5.03
**DALYR**	Low-middle SDI	Female	20-54 years	Alcohol use	1990	0.72	1.13	0.31
**DR**	Low-middle SDI	Male	20-54 years	Diet low in vegetables	1990	0.36	0.73	-0.08
**DR**	Low-middle SDI	Male	20-54 years	Alcohol use	1990	0.18	0.24	0.11
**DR**	Low-middle SDI	Male	20-54 years	Smoking	1990	0.45	0.55	0.36
**DR**	Low-middle SDI	Male	20-54 years	Chewing tobacco	1990	0.27	0.38	0.17
**DALYR**	Low-middle SDI	Male	20-54 years	Smoking	1990	18.91	22.93	15.01
**DALYR**	Low-middle SDI	Male	20-54 years	Diet low in vegetables	1990	15.44	31.43	-3.45
**DALYR**	Low-middle SDI	Male	20-54 years	Chewing tobacco	1990	11.71	16.11	7.25

**DALYR**	Low-middle SDI	Male	20-54 years	Alcohol use	1990	7.63	10.59	4.70
**DR**	Low-middle SDI	Both	20-54 years	Diet low in vegetables	2021	0.29	0.59	-0.06
**DR**	Low-middle SDI	Both	20-54 years	Alcohol use	2021	0.13	0.18	0.09
**DR**	Low-middle SDI	Both	20-54 years	Smoking	2021	0.18	0.22	0.14
**DR**	Low-middle SDI	Both	20-54 years	Chewing tobacco	2021	0.18	0.23	0.12
**DALYR**	Low-middle SDI	Both	20-54 years	Smoking	2021	7.31	9.04	5.67
**DALYR**	Low-middle SDI	Both	20-54 years	Diet low in vegetables	2021	12.59	25.45	-2.67
**DALYR**	Low-middle SDI	Both	20-54 years	Chewing tobacco	2021	7.52	9.99	5.16
**DALYR**	Low-middle SDI	Both	20-54 years	Alcohol use	2021	5.81	7.77	3.98
**DR**	Low-middle SDI	Female	20-54 years	Diet low in vegetables	2021	0.22	0.48	-0.05
**DR**	Low-middle SDI	Female	20-54 years	Alcohol use	2021	0.02	0.03	0.01
**DR**	Low-middle SDI	Female	20-54 years	Smoking	2021	0.02	0.03	0.02
**DR**	Low-middle SDI	Female	20-54 years	Chewing tobacco	2021	0.13	0.20	0.07
**DALYR**	Low-middle SDI	Female	20-54 years	Smoking	2021	0.90	1.30	0.63
**DALYR**	Low-middle SDI	Female	20-54 years	Diet low in vegetables	2021	9.77	21.06	-2.06
**DALYR**	Low-middle SDI	Female	20-54 years	Chewing tobacco	2021	5.33	8.48	3.04
**DALYR**	Low-middle SDI	Female	20-54 years	Alcohol use	2021	0.87	1.31	0.51
**DR**	Low-middle SDI	Male	20-54 years	Diet low in vegetables	2021	0.36	0.71	-0.08
**DR**	Low-middle SDI	Male	20-54 years	Alcohol use	2021	0.25	0.33	0.17
**DR**	Low-middle SDI	Male	20-54 years	Smoking	2021	0.33	0.40	0.26
**DR**	Low-middle SDI	Male	20-54 years	Chewing tobacco	2021	0.23	0.32	0.14
**DALYR**	Low-middle SDI	Male	20-54 years	Smoking	2021	13.74	16.94	10.69
**DALYR**	Low-middle SDI	Male	20-54 years	Diet low in vegetables	2021	15.40	30.53	-3.30
**DALYR**	Low-middle SDI	Male	20-54 years	Chewing tobacco	2021	9.72	13.92	6.06
**DALYR**	Low-middle SDI	Male	20-54 years	Alcohol use	2021	10.75	14.41	7.41
**DR**	Low-middle SDI	Both	55-59 years	Diet low in vegetables	1990	2.76	5.41	-0.66
**DR**	Low-middle SDI	Both	55-59 years	Smoking	1990	2.91	3.58	2.29
**DR**	Low-middle SDI	Both	55-59 years	Chewing tobacco	1990	2.09	2.91	1.35
**DR**	Low-middle SDI	Both	55-59 years	Alcohol use	1990	0.76	1.07	0.46

**DALYR**	Low-middle SDI	Both	55-59 years	Smoking	1990	98.40	120.88	77.22
**DALYR**	Low-middle SDI	Both	55-59 years	Diet low in vegetables	1990	93.28	182.94	-22.24
**DALYR**	Low-middle SDI	Both	55-59 years	Alcohol use	1990	25.78	36.09	15.60
**DALYR**	Low-middle SDI	Both	55-59 years	Chewing tobacco	1990	70.45	98.38	45.53
**DR**	Low-middle SDI	Female	55-59 years	Diet low in vegetables	1990	2.30	4.77	-0.57
**DR**	Low-middle SDI	Female	55-59 years	Smoking	1990	0.67	0.96	0.46
**DR**	Low-middle SDI	Female	55-59 years	Chewing tobacco	1990	1.74	2.84	0.96
**DR**	Low-middle SDI	Female	55-59 years	Alcohol use	1990	0.12	0.18	0.06
**DALYR**	Low-middle SDI	Female	55-59 years	Smoking	1990	22.70	32.42	15.56
**DALYR**	Low-middle SDI	Female	55-59 years	Diet low in vegetables	1990	77.75	161.29	-19.13
**DALYR**	Low-middle SDI	Female	55-59 years	Alcohol use	1990	3.99	6.14	1.92
**DALYR**	Low-middle SDI	Female	55-59 years	Chewing tobacco	1990	58.75	96.00	32.36
**DR**	Low-middle SDI	Male	55-59 years	Diet low in vegetables	1990	3.19	6.38	-0.75
**DR**	Low-middle SDI	Male	55-59 years	Smoking	1990	5.02	6.22	3.96
**DR**	Low-middle SDI	Male	55-59 years	Chewing tobacco	1990	2.41	3.53	1.46
**DR**	Low-middle SDI	Male	55-59 years	Alcohol use	1990	1.37	1.92	0.83
**DALYR**	Low-middle SDI	Male	55-59 years	Smoking	1990	169.68	210.05	133.73
**DALYR**	Low-middle SDI	Male	55-59 years	Diet low in vegetables	1990	107.91	216.04	-25.23
**DALYR**	Low-middle SDI	Male	55-59 years	Alcohol use	1990	46.29	65.03	28.09
**DALYR**	Low-middle SDI	Male	55-59 years	Chewing tobacco	1990	81.47	119.20	49.29
**DR**	Low-middle SDI	Both	55-59 years	Diet low in vegetables	2021	2.35	4.83	-0.50
**DR**	Low-middle SDI	Both	55-59 years	Smoking	2021	2.08	2.55	1.62
**DR**	Low-middle SDI	Both	55-59 years	Chewing tobacco	2021	1.62	2.23	1.07
**DR**	Low-middle SDI	Both	55-59 years	Alcohol use	2021	0.96	1.30	0.65
**DALYR**	Low-middle SDI	Both	55-59 years	Smoking	2021	70.52	86.34	54.90
**DALYR**	Low-middle SDI	Both	55-59 years	Diet low in vegetables	2021	79.57	163.72	-16.82
**DALYR**	Low-middle SDI	Both	55-59 years	Alcohol use	2021	32.49	44.12	21.84
**DALYR**	Low-middle SDI	Both	55-59 years	Chewing tobacco	2021	54.87	75.42	36.28
**DR**	Low-middle SDI	Female	55-59 years	Diet low in vegetables	2021	1.75	3.67	-0.39

**DR**	Low-middle SDI	Female	55-59 years	Smoking	2021	0.35	0.53	0.22
**DR**	Low-middle SDI	Female	55-59 years	Chewing tobacco	2021	1.22	2.00	0.67
**DR**	Low-middle SDI	Female	55-59 years	Alcohol use	2021	0.13	0.21	0.08
**DALYR**	Low-middle SDI	Female	55-59 years	Smoking	2021	11.82	18.04	7.52
**DALYR**	Low-middle SDI	Female	55-59 years	Diet low in vegetables	2021	59.10	124.05	-13.19
**DALYR**	Low-middle SDI	Female	55-59 years	Alcohol use	2021	4.55	7.01	2.65
**DALYR**	Low-middle SDI	Female	55-59 years	Chewing tobacco	2021	41.28	67.46	22.86
**DR**	Low-middle SDI	Male	55-59 years	Diet low in vegetables	2021	2.97	5.87	-0.63
**DR**	Low-middle SDI	Male	55-59 years	Smoking	2021	3.88	4.73	3.02
**DR**	Low-middle SDI	Male	55-59 years	Chewing tobacco	2021	2.04	3.05	1.15
**DR**	Low-middle SDI	Male	55-59 years	Alcohol use	2021	1.81	2.46	1.22
**DALYR**	Low-middle SDI	Male	55-59 years	Smoking	2021	131.20	160.29	102.22
**DALYR**	Low-middle SDI	Male	55-59 years	Diet low in vegetables	2021	100.73	198.84	-21.32
**DALYR**	Low-middle SDI	Male	55-59 years	Alcohol use	2021	61.36	83.32	41.31
**DALYR**	Low-middle SDI	Male	55-59 years	Chewing tobacco	2021	68.92	103.31	38.85
**DR**	Low-middle SDI	Both	60-64 years	Diet low in vegetables	1990	3.81	7.47	-0.87
**DR**	Low-middle SDI	Both	60-64 years	Smoking	1990	4.41	5.46	3.49
**DR**	Low-middle SDI	Both	60-64 years	Chewing tobacco	1990	2.93	3.92	1.97
**DR**	Low-middle SDI	Both	60-64 years	Alcohol use	1990	1.00	1.42	0.62
**DALYR**	Low-middle SDI	Both	60-64 years	Smoking	1990	128.63	159.09	101.95
**DALYR**	Low-middle SDI	Both	60-64 years	Diet low in vegetables	1990	111.06	217.65	-25.49
**DALYR**	Low-middle SDI	Both	60-64 years	Alcohol use	1990	29.23	41.48	17.97
**DALYR**	Low-middle SDI	Both	60-64 years	Chewing tobacco	1990	85.43	114.39	57.58
**DR**	Low-middle SDI	Female	60-64 years	Diet low in vegetables	1990	2.92	6.16	-0.73
**DR**	Low-middle SDI	Female	60-64 years	Smoking	1990	0.91	1.32	0.64
**DR**	Low-middle SDI	Female	60-64 years	Chewing tobacco	1990	2.13	3.79	1.21
**DR**	Low-middle SDI	Female	60-64 years	Alcohol use	1990	0.17	0.26	0.09
**DALYR**	Low-middle SDI	Female	60-64 years	Smoking	1990	26.57	38.46	18.69
**DALYR**	Low-middle SDI	Female	60-64 years	Diet low in vegetables	1990	85.16	179.78	-21.31

**DALYR**	Low-middle SDI	Female	60-64 years	Alcohol use	1990	4.84	7.48	2.50
**DALYR**	Low-middle SDI	Female	60-64 years	Chewing tobacco	1990	62.21	110.75	35.27
**DR**	Low-middle SDI	Male	60-64 years	Diet low in vegetables	1990	4.64	9.36	-1.04
**DR**	Low-middle SDI	Male	60-64 years	Smoking	1990	7.67	9.55	6.11
**DR**	Low-middle SDI	Male	60-64 years	Chewing tobacco	1990	3.67	5.32	2.22
**DR**	Low-middle SDI	Male	60-64 years	Alcohol use	1990	1.78	2.54	1.10
**DALYR**	Low-middle SDI	Male	60-64 years	Smoking	1990	223.86	278.71	178.21
**DALYR**	Low-middle SDI	Male	60-64 years	Diet low in vegetables	1990	135.23	272.78	-30.42
**DALYR**	Low-middle SDI	Male	60-64 years	Alcohol use	1990	51.99	73.83	32.09
**DALYR**	Low-middle SDI	Male	60-64 years	Chewing tobacco	1990	107.09	155.23	64.68
**DR**	Low-middle SDI	Both	60-64 years	Diet low in vegetables	2021	3.00	6.07	-0.64
**DR**	Low-middle SDI	Both	60-64 years	Smoking	2021	2.87	3.57	2.21
**DR**	Low-middle SDI	Both	60-64 years	Chewing tobacco	2021	2.05	2.75	1.38
**DR**	Low-middle SDI	Both	60-64 years	Alcohol use	2021	1.18	1.60	0.79
**DALYR**	Low-middle SDI	Both	60-64 years	Diet low in vegetables	2021	87.70	176.96	-18.57
**DALYR**	Low-middle SDI	Both	60-64 years	Smoking	2021	83.88	104.20	64.58
**DALYR**	Low-middle SDI	Both	60-64 years	Alcohol use	2021	34.54	46.67	23.00
**DALYR**	Low-middle SDI	Both	60-64 years	Chewing tobacco	2021	59.78	80.37	40.32
**DR**	Low-middle SDI	Female	60-64 years	Diet low in vegetables	2021	2.10	4.40	-0.46
**DR**	Low-middle SDI	Female	60-64 years	Smoking	2021	0.44	0.62	0.29
**DR**	Low-middle SDI	Female	60-64 years	Chewing tobacco	2021	1.36	2.43	0.70
**DR**	Low-middle SDI	Female	60-64 years	Alcohol use	2021	0.19	0.29	0.11
**DALYR**	Low-middle SDI	Female	60-64 years	Diet low in vegetables	2021	61.21	128.29	-13.47
**DALYR**	Low-middle SDI	Female	60-64 years	Smoking	2021	12.80	18.02	8.46
**DALYR**	Low-middle SDI	Female	60-64 years	Alcohol use	2021	5.58	8.55	3.25
**DALYR**	Low-middle SDI	Female	60-64 years	Chewing tobacco	2021	39.65	70.79	20.53
**DR**	Low-middle SDI	Male	60-64 years	Diet low in vegetables	2021	3.96	7.98	-0.85
**DR**	Low-middle SDI	Male	60-64 years	Smoking	2021	5.45	6.75	4.20
**DR**	Low-middle SDI	Male	60-64 years	Chewing tobacco	2021	2.78	4.06	1.65

**DR**	Low-middle SDI	Male	60-64 years	Alcohol use	2021	2.23	3.02	1.48
**DALYR**	Low-middle SDI	Male	60-64 years	Diet low in vegetables	2021	115.76	233.20	-24.94
**DALYR**	Low-middle SDI	Male	60-64 years	Smoking	2021	159.17	197.31	122.71
**DALYR**	Low-middle SDI	Male	60-64 years	Alcohol use	2021	65.22	88.15	43.19
**DALYR**	Low-middle SDI	Male	60-64 years	Chewing tobacco	2021	81.10	118.61	48.20
**DR**	Low-middle SDI	Both	65-74 years	Diet low in vegetables	1990	5.47	10.64	-1.32
**DR**	Low-middle SDI	Both	65-74 years	Alcohol use	1990	1.17	1.60	0.75
**DR**	Low-middle SDI	Both	65-74 years	Smoking	1990	6.26	7.73	4.83
**DR**	Low-middle SDI	Both	65-74 years	Chewing tobacco	1990	3.54	4.76	2.51
**DALYR**	Low-middle SDI	Both	65-74 years	Smoking	1990	142.03	175.15	109.85
**DALYR**	Low-middle SDI	Both	65-74 years	Alcohol use	1990	26.63	36.82	17.11
**DALYR**	Low-middle SDI	Both	65-74 years	Diet low in vegetables	1990	123.61	240.61	-29.92
**DALYR**	Low-middle SDI	Both	65-74 years	Chewing tobacco	1990	80.35	107.05	57.05
**DR**	Low-middle SDI	Female	65-74 years	Diet low in vegetables	1990	4.54	9.28	-1.16
**DR**	Low-middle SDI	Female	65-74 years	Alcohol use	1990	0.21	0.31	0.12
**DR**	Low-middle SDI	Female	65-74 years	Smoking	1990	1.42	2.01	0.99
**DR**	Low-middle SDI	Female	65-74 years	Chewing tobacco	1990	3.00	4.97	1.75
**DALYR**	Low-middle SDI	Female	65-74 years	Smoking	1990	31.79	44.91	22.06
**DALYR**	Low-middle SDI	Female	65-74 years	Alcohol use	1990	4.76	7.00	2.71
**DALYR**	Low-middle SDI	Female	65-74 years	Diet low in vegetables	1990	101.87	207.65	-26.01
**DALYR**	Low-middle SDI	Female	65-74 years	Chewing tobacco	1990	67.21	110.84	38.92
**DR**	Low-middle SDI	Male	65-74 years	Diet low in vegetables	1990	6.37	12.86	-1.48
**DR**	Low-middle SDI	Male	65-74 years	Alcohol use	1990	2.11	2.92	1.34
**DR**	Low-middle SDI	Male	65-74 years	Smoking	1990	11.01	13.59	8.58
**DR**	Low-middle SDI	Male	65-74 years	Chewing tobacco	1990	4.07	5.88	2.59
**DALYR**	Low-middle SDI	Male	65-74 years	Smoking	1990	250.41	309.29	195.40
**DALYR**	Low-middle SDI	Male	65-74 years	Alcohol use	1990	48.12	66.65	30.64
**DALYR**	Low-middle SDI	Male	65-74 years	Diet low in vegetables	1990	144.98	292.12	-33.61
**DALYR**	Low-middle SDI	Male	65-74 years	Chewing tobacco	1990	93.26	135.24	59.60

**DR**	Low-middle SDI	Both	65-74 years	Diet low in vegetables	2021	4.52	9.19	-0.96
**DR**	Low-middle SDI	Both	65-74 years	Alcohol use	2021	1.44	1.93	0.95
**DR**	Low-middle SDI	Both	65-74 years	Smoking	2021	4.22	5.16	3.29
**DR**	Low-middle SDI	Both	65-74 years	Chewing tobacco	2021	2.86	4.02	1.93
**DALYR**	Low-middle SDI	Both	65-74 years	Smoking	2021	95.39	116.46	74.29
**DALYR**	Low-middle SDI	Both	65-74 years	Chewing tobacco	2021	64.54	90.41	43.23
**DALYR**	Low-middle SDI	Both	65-74 years	Diet low in vegetables	2021	101.78	207.05	-21.55
**DALYR**	Low-middle SDI	Both	65-74 years	Alcohol use	2021	32.64	43.69	21.61
**DR**	Low-middle SDI	Female	65-74 years	Diet low in vegetables	2021	3.45	7.30	-0.78
**DR**	Low-middle SDI	Female	65-74 years	Alcohol use	2021	0.24	0.35	0.14
**DR**	Low-middle SDI	Female	65-74 years	Smoking	2021	0.71	1.08	0.49
**DR**	Low-middle SDI	Female	65-74 years	Chewing tobacco	2021	2.28	3.75	1.32
**DALYR**	Low-middle SDI	Female	65-74 years	Smoking	2021	15.84	24.01	10.91
**DALYR**	Low-middle SDI	Female	65-74 years	Chewing tobacco	2021	50.80	83.43	29.70
**DALYR**	Low-middle SDI	Female	65-74 years	Diet low in vegetables	2021	77.20	162.83	-17.37
**DALYR**	Low-middle SDI	Female	65-74 years	Alcohol use	2021	5.34	8.02	3.27
**DR**	Low-middle SDI	Male	65-74 years	Diet low in vegetables	2021	5.68	11.26	-1.20
**DR**	Low-middle SDI	Male	65-74 years	Alcohol use	2021	2.74	3.68	1.81
**DR**	Low-middle SDI	Male	65-74 years	Smoking	2021	8.04	9.72	6.26
**DR**	Low-middle SDI	Male	65-74 years	Chewing tobacco	2021	3.50	4.90	2.04
**DALYR**	Low-middle SDI	Male	65-74 years	Smoking	2021	181.88	219.88	141.37
**DALYR**	Low-middle SDI	Male	65-74 years	Chewing tobacco	2021	79.48	111.88	46.06
**DALYR**	Low-middle SDI	Male	65-74 years	Diet low in vegetables	2021	128.51	255.06	-27.11
**DALYR**	Low-middle SDI	Male	65-74 years	Alcohol use	2021	62.33	83.37	41.07
**DR**	Low-middle SDI	Both	75+ years	Diet low in vegetables	1990	7.84	15.39	-1.85
**DR**	Low-middle SDI	Both	75+ years	Alcohol use	1990	1.00	1.36	0.63
**DR**	Low-middle SDI	Both	75+ years	Smoking	1990	8.06	9.99	6.12
**DR**	Low-middle SDI	Both	75+ years	Chewing tobacco	1990	4.81	6.43	3.28
**DALYR**	Low-middle SDI	Both	75+ years	Smoking	1990	113.14	140.17	86.08

**DALYR**	Low-middle SDI	Both	75+ years	Chewing tobacco	1990	67.49	90.51	45.74
**DALYR**	Low-middle SDI	Both	75+ years	Diet low in vegetables	1990	109.83	215.61	-26.04
**DALYR**	Low-middle SDI	Both	75+ years	Alcohol use	1990	14.46	19.75	8.98
**DR**	Low-middle SDI	Female	75+ years	Diet low in vegetables	1990	7.35	15.00	-1.77
**DR**	Low-middle SDI	Female	75+ years	Alcohol use	1990	0.23	0.35	0.14
**DR**	Low-middle SDI	Female	75+ years	Smoking	1990	2.09	2.99	1.45
**DR**	Low-middle SDI	Female	75+ years	Chewing tobacco	1990	4.97	7.39	2.85
**DALYR**	Low-middle SDI	Female	75+ years	Smoking	1990	28.45	40.63	19.70
**DALYR**	Low-middle SDI	Female	75+ years	Chewing tobacco	1990	68.99	105.31	39.80
**DALYR**	Low-middle SDI	Female	75+ years	Diet low in vegetables	1990	101.85	205.94	-24.58
**DALYR**	Low-middle SDI	Female	75+ years	Alcohol use	1990	3.27	4.91	1.99
**DR**	Low-middle SDI	Male	75+ years	Diet low in vegetables	1990	8.35	16.80	-1.95
**DR**	Low-middle SDI	Male	75+ years	Alcohol use	1990	1.80	2.46	1.10
**DR**	Low-middle SDI	Male	75+ years	Smoking	1990	14.27	17.67	10.88
**DR**	Low-middle SDI	Male	75+ years	Chewing tobacco	1990	4.65	6.66	2.81
**DALYR**	Low-middle SDI	Male	75+ years	Smoking	1990	201.44	249.02	153.65
**DALYR**	Low-middle SDI	Male	75+ years	Chewing tobacco	1990	65.93	95.01	39.51
**DALYR**	Low-middle SDI	Male	75+ years	Diet low in vegetables	1990	118.15	236.55	-27.56
**DALYR**	Low-middle SDI	Male	75+ years	Alcohol use	1990	26.13	35.72	16.03
**DR**	Low-middle SDI	Both	75+ years	Diet low in vegetables	2021	7.00	14.08	-1.52
**DR**	Low-middle SDI	Both	75+ years	Alcohol use	2021	1.34	1.81	0.87
**DR**	Low-middle SDI	Both	75+ years	Smoking	2021	5.91	7.33	4.53
**DR**	Low-middle SDI	Both	75+ years	Chewing tobacco	2021	4.61	6.34	3.20
**DALYR**	Low-middle SDI	Both	75+ years	Smoking	2021	81.14	100.49	62.29
**DALYR**	Low-middle SDI	Both	75+ years	Diet low in vegetables	2021	95.61	192.79	-20.78
**DALYR**	Low-middle SDI	Both	75+ years	Chewing tobacco	2021	62.63	85.71	43.46
**DALYR**	Low-middle SDI	Both	75+ years	Alcohol use	2021	19.09	25.96	12.38
**DR**	Low-middle SDI	Female	75+ years	Diet low in vegetables	2021	5.95	12.21	-1.33
**DR**	Low-middle SDI	Female	75+ years	Alcohol use	2021	0.25	0.37	0.15

**DR**	Low-middle SDI	Female	75+ years	Smoking	2021	1.29	2.02	0.90
**DR**	Low-middle SDI	Female	75+ years	Chewing tobacco	2021	4.20	6.75	2.38
**DALYR**	Low-middle SDI	Female	75+ years	Smoking	2021	17.24	26.61	11.85
**DALYR**	Low-middle SDI	Female	75+ years	Diet low in vegetables	2021	80.24	164.40	-17.97
**DALYR**	Low-middle SDI	Female	75+ years	Chewing tobacco	2021	56.43	90.72	32.26
**DALYR**	Low-middle SDI	Female	75+ years	Alcohol use	2021	3.45	5.20	2.02
**DR**	Low-middle SDI	Male	75+ years	Diet low in vegetables	2021	8.28	16.60	-1.78
**DR**	Low-middle SDI	Male	75+ years	Alcohol use	2021	2.68	3.63	1.75
**DR**	Low-middle SDI	Male	75+ years	Smoking	2021	11.56	14.27	8.86
**DR**	Low-middle SDI	Male	75+ years	Chewing tobacco	2021	5.12	7.20	3.17
**DALYR**	Low-middle SDI	Male	75+ years	Smoking	2021	159.56	196.95	122.27
**DALYR**	Low-middle SDI	Male	75+ years	Diet low in vegetables	2021	114.49	229.21	-24.75
**DALYR**	Low-middle SDI	Male	75+ years	Chewing tobacco	2021	70.23	99.89	43.35
**DALYR**	Low-middle SDI	Male	75+ years	Alcohol use	2021	38.28	52.08	25.03
**DR**	Middle SDI	Both	20-54 years	Alcohol use	1990	0.67	0.94	0.47
**DR**	Middle SDI	Both	20-54 years	Smoking	1990	1.27	1.60	0.99
**DR**	Middle SDI	Both	20-54 years	Chewing tobacco	1990	0.09	0.14	0.06
**DR**	Middle SDI	Both	20-54 years	Diet low in vegetables	1990	0.81	1.63	-0.18
**DALYR**	Middle SDI	Both	20-54 years	Diet low in vegetables	1990	35.09	70.02	-7.71
**DALYR**	Middle SDI	Both	20-54 years	Alcohol use	1990	29.46	40.95	20.73
**DALYR**	Middle SDI	Both	20-54 years	Smoking	1990	53.76	67.88	41.97
**DALYR**	Middle SDI	Both	20-54 years	Chewing tobacco	1990	4.10	5.99	2.51
**DR**	Middle SDI	Female	20-54 years	Alcohol use	1990	0.04	0.06	0.02
**DR**	Middle SDI	Female	20-54 years	Smoking	1990	0.06	0.09	0.04
**DR**	Middle SDI	Female	20-54 years	Chewing tobacco	1990	0.03	0.05	0.02
**DR**	Middle SDI	Female	20-54 years	Diet low in vegetables	1990	0.35	0.74	-0.07
**DALYR**	Middle SDI	Female	20-54 years	Diet low in vegetables	1990	15.14	31.90	-3.08
**DALYR**	Middle SDI	Female	20-54 years	Alcohol use	1990	1.78	2.68	0.94
**DALYR**	Middle SDI	Female	20-54 years	Smoking	1990	2.56	3.59	1.57

**DALYR**	Middle SDI	Female	20-54 years	Chewing tobacco	1990	1.47	2.20	0.77
**DR**	Middle SDI	Male	20-54 years	Alcohol use	1990	1.28	1.81	0.89
**DR**	Middle SDI	Male	20-54 years	Smoking	1990	2.42	3.06	1.89
**DR**	Middle SDI	Male	20-54 years	Chewing tobacco	1990	0.15	0.23	0.08
**DR**	Middle SDI	Male	20-54 years	Diet low in vegetables	1990	1.26	2.55	-0.27
**DALYR**	Middle SDI	Male	20-54 years	Diet low in vegetables	1990	54.26	109.86	-11.80
**DALYR**	Middle SDI	Male	20-54 years	Alcohol use	1990	56.05	78.51	39.18
**DALYR**	Middle SDI	Male	20-54 years	Smoking	1990	102.95	130.11	80.31
**DALYR**	Middle SDI	Male	20-54 years	Chewing tobacco	1990	6.63	10.09	3.68
**DR**	Middle SDI	Both	20-54 years	Alcohol use	2021	0.42	0.58	0.30
**DR**	Middle SDI	Both	20-54 years	Smoking	2021	0.64	0.85	0.48
**DR**	Middle SDI	Both	20-54 years	Chewing tobacco	2021	0.08	0.11	0.05
**DR**	Middle SDI	Both	20-54 years	Diet low in vegetables	2021	0.21	0.44	-0.04
**DALYR**	Middle SDI	Both	20-54 years	Diet low in vegetables	2021	9.12	18.68	-1.90
**DALYR**	Middle SDI	Both	20-54 years	Alcohol use	2021	17.79	24.34	12.65
**DALYR**	Middle SDI	Both	20-54 years	Smoking	2021	26.18	34.72	19.50
**DALYR**	Middle SDI	Both	20-54 years	Chewing tobacco	2021	3.45	4.84	2.23
**DR**	Middle SDI	Female	20-54 years	Alcohol use	2021	0.02	0.03	0.01
**DR**	Middle SDI	Female	20-54 years	Smoking	2021	0.02	0.03	0.02
**DR**	Middle SDI	Female	20-54 years	Chewing tobacco	2021	0.03	0.05	0.02
**DR**	Middle SDI	Female	20-54 years	Diet low in vegetables	2021	0.10	0.21	-0.02
**DALYR**	Middle SDI	Female	20-54 years	Diet low in vegetables	2021	4.46	9.23	-0.98
**DALYR**	Middle SDI	Female	20-54 years	Alcohol use	2021	0.93	1.30	0.62
**DALYR**	Middle SDI	Female	20-54 years	Smoking	2021	0.98	1.32	0.71
**DALYR**	Middle SDI	Female	20-54 years	Chewing tobacco	2021	1.34	2.10	0.69
**DR**	Middle SDI	Male	20-54 years	Alcohol use	2021	0.81	1.12	0.58
**DR**	Middle SDI	Male	20-54 years	Smoking	2021	1.24	1.65	0.92
**DR**	Middle SDI	Male	20-54 years	Chewing tobacco	2021	0.13	0.19	0.07
**DR**	Middle SDI	Male	20-54 years	Diet low in vegetables	2021	0.32	0.69	-0.07

**DALYR**	Middle SDI	Male	20-54 years	Diet low in vegetables	2021	13.71	29.08	-2.80
**DALYR**	Middle SDI	Male	20-54 years	Alcohol use	2021	34.37	47.18	24.50
**DALYR**	Middle SDI	Male	20-54 years	Smoking	2021	50.96	67.72	37.94
**DALYR**	Middle SDI	Male	20-54 years	Chewing tobacco	2021	5.54	8.13	3.16
**DR**	Middle SDI	Both	55-59 years	Alcohol use	1990	5.72	8.09	3.87
**DR**	Middle SDI	Both	55-59 years	Diet low in vegetables	1990	8.18	16.07	-1.74
**DR**	Middle SDI	Both	55-59 years	Smoking	1990	14.34	18.50	11.11
**DR**	Middle SDI	Both	55-59 years	Chewing tobacco	1990	0.73	1.12	0.43
**DALYR**	Middle SDI	Both	55-59 years	Smoking	1990	484.87	626.32	375.60
**DALYR**	Middle SDI	Both	55-59 years	Chewing tobacco	1990	24.64	37.97	14.64
**DALYR**	Middle SDI	Both	55-59 years	Diet low in vegetables	1990	276.40	542.96	-59.00
**DALYR**	Middle SDI	Both	55-59 years	Alcohol use	1990	193.19	273.57	130.65
**DR**	Middle SDI	Female	55-59 years	Alcohol use	1990	0.40	0.68	0.19
**DR**	Middle SDI	Female	55-59 years	Diet low in vegetables	1990	4.26	8.99	-0.82
**DR**	Middle SDI	Female	55-59 years	Smoking	1990	1.15	1.76	0.62
**DR**	Middle SDI	Female	55-59 years	Chewing tobacco	1990	0.39	0.64	0.19
**DALYR**	Middle SDI	Female	55-59 years	Smoking	1990	38.79	59.62	20.90
**DALYR**	Middle SDI	Female	55-59 years	Chewing tobacco	1990	13.31	21.77	6.25
**DALYR**	Middle SDI	Female	55-59 years	Diet low in vegetables	1990	144.05	304.00	-27.75
**DALYR**	Middle SDI	Female	55-59 years	Alcohol use	1990	13.36	22.99	6.41
**DR**	Middle SDI	Male	55-59 years	Alcohol use	1990	10.77	15.31	7.34
**DR**	Middle SDI	Male	55-59 years	Diet low in vegetables	1990	11.90	23.95	-2.74
**DR**	Middle SDI	Male	55-59 years	Smoking	1990	26.89	34.89	20.81
**DR**	Middle SDI	Male	55-59 years	Chewing tobacco	1990	1.05	1.79	0.56
**DALYR**	Middle SDI	Male	55-59 years	Smoking	1990	908.94	1178.84	703.66
**DALYR**	Middle SDI	Male	55-59 years	Chewing tobacco	1990	35.41	60.60	19.04
**DALYR**	Middle SDI	Male	55-59 years	Diet low in vegetables	1990	402.22	809.36	-92.48
**DALYR**	Middle SDI	Male	55-59 years	Alcohol use	1990	364.15	517.49	248.30
**DR**	Middle SDI	Both	55-59 years	Alcohol use	2021	2.90	4.17	2.00

**DR**	Middle SDI	Both	55-59 years	Diet low in vegetables	2021	1.42	3.13	-0.33
**DR**	Middle SDI	Both	55-59 years	Smoking	2021	6.08	8.33	4.35
**DR**	Middle SDI	Both	55-59 years	Chewing tobacco	2021	0.49	0.73	0.30
**DALYR**	Middle SDI	Both	55-59 years	Smoking	2021	206.17	282.05	147.65
**DALYR**	Middle SDI	Both	55-59 years	Chewing tobacco	2021	16.44	24.78	10.26
**DALYR**	Middle SDI	Both	55-59 years	Diet low in vegetables	2021	47.99	106.12	-11.30
**DALYR**	Middle SDI	Both	55-59 years	Alcohol use	2021	98.45	141.44	67.73
**DR**	Middle SDI	Female	55-59 years	Alcohol use	2021	0.14	0.23	0.09
**DR**	Middle SDI	Female	55-59 years	Diet low in vegetables	2021	0.67	1.43	-0.14
**DR**	Middle SDI	Female	55-59 years	Smoking	2021	0.32	0.48	0.21
**DR**	Middle SDI	Female	55-59 years	Chewing tobacco	2021	0.24	0.40	0.12
**DALYR**	Middle SDI	Female	55-59 years	Smoking	2021	10.91	16.42	7.18
**DALYR**	Middle SDI	Female	55-59 years	Chewing tobacco	2021	8.07	13.53	3.90
**DALYR**	Middle SDI	Female	55-59 years	Diet low in vegetables	2021	22.63	48.44	-4.89
**DALYR**	Middle SDI	Female	55-59 years	Alcohol use	2021	4.92	7.71	2.92
**DR**	Middle SDI	Male	55-59 years	Alcohol use	2021	5.78	8.33	3.99
**DR**	Middle SDI	Male	55-59 years	Diet low in vegetables	2021	2.20	5.06	-0.53
**DR**	Middle SDI	Male	55-59 years	Smoking	2021	12.09	16.63	8.64
**DR**	Middle SDI	Male	55-59 years	Chewing tobacco	2021	0.74	1.19	0.39
**DALYR**	Middle SDI	Male	55-59 years	Smoking	2021	409.78	563.23	292.74
**DALYR**	Middle SDI	Male	55-59 years	Chewing tobacco	2021	25.16	40.36	13.17
**DALYR**	Middle SDI	Male	55-59 years	Diet low in vegetables	2021	74.43	171.65	-18.07
**DALYR**	Middle SDI	Male	55-59 years	Alcohol use	2021	195.98	282.75	135.05
**DR**	Middle SDI	Both	60-64 years	Alcohol use	1990	7.45	10.63	4.98
**DR**	Middle SDI	Both	60-64 years	Diet low in vegetables	1990	11.87	23.66	-2.57
**DR**	Middle SDI	Both	60-64 years	Smoking	1990	21.52	27.44	16.43
**DR**	Middle SDI	Both	60-64 years	Chewing tobacco	1990	1.03	1.56	0.62
**DALYR**	Middle SDI	Both	60-64 years	Smoking	1990	627.25	799.33	478.96
**DALYR**	Middle SDI	Both	60-64 years	Chewing tobacco	1990	29.96	45.41	18.21

**DALYR**	Middle SDI	Both	60-64 years	Diet low in vegetables	1990	345.79	688.73	-74.87
**DALYR**	Middle SDI	Both	60-64 years	Alcohol use	1990	217.18	309.49	144.93
**DR**	Middle SDI	Female	60-64 years	Alcohol use	1990	0.66	1.23	0.29
**DR**	Middle SDI	Female	60-64 years	Diet low in vegetables	1990	6.86	14.82	-1.38
**DR**	Middle SDI	Female	60-64 years	Smoking	1990	2.39	3.77	1.18
**DR**	Middle SDI	Female	60-64 years	Chewing tobacco	1990	0.56	0.95	0.25
**DALYR**	Middle SDI	Female	60-64 years	Smoking	1990	69.58	109.97	34.41
**DALYR**	Middle SDI	Female	60-64 years	Chewing tobacco	1990	16.19	27.79	7.42
**DALYR**	Middle SDI	Female	60-64 years	Diet low in vegetables	1990	199.91	431.33	-40.33
**DALYR**	Middle SDI	Female	60-64 years	Alcohol use	1990	19.34	35.92	8.47
**DR**	Middle SDI	Male	60-64 years	Alcohol use	1990	14.11	19.89	9.34
**DR**	Middle SDI	Male	60-64 years	Diet low in vegetables	1990	16.77	33.65	-3.75
**DR**	Middle SDI	Male	60-64 years	Smoking	1990	40.29	52.09	30.75
**DR**	Middle SDI	Male	60-64 years	Chewing tobacco	1990	1.49	2.54	0.77
**DALYR**	Middle SDI	Male	60-64 years	Smoking	1990	1174.30	1516.19	896.26
**DALYR**	Middle SDI	Male	60-64 years	Chewing tobacco	1990	43.48	74.05	22.35
**DALYR**	Middle SDI	Male	60-64 years	Diet low in vegetables	1990	488.89	979.89	-109.14
**DALYR**	Middle SDI	Male	60-64 years	Alcohol use	1990	411.25	580.12	272.10
**DR**	Middle SDI	Both	60-64 years	Alcohol use	2021	3.89	5.53	2.64
**DR**	Middle SDI	Both	60-64 years	Diet low in vegetables	2021	1.97	4.44	-0.44
**DR**	Middle SDI	Both	60-64 years	Smoking	2021	9.35	12.50	6.74
**DR**	Middle SDI	Both	60-64 years	Chewing tobacco	2021	0.76	1.10	0.48
**DALYR**	Middle SDI	Both	60-64 years	Smoking	2021	272.09	363.27	195.98
**DALYR**	Middle SDI	Both	60-64 years	Chewing tobacco	2021	22.30	32.24	14.05
**DALYR**	Middle SDI	Both	60-64 years	Diet low in vegetables	2021	57.46	129.71	-12.73
**DALYR**	Middle SDI	Both	60-64 years	Alcohol use	2021	113.25	161.30	76.84
**DR**	Middle SDI	Female	60-64 years	Alcohol use	2021	0.22	0.33	0.12
**DR**	Middle SDI	Female	60-64 years	Diet low in vegetables	2021	0.92	1.88	-0.21
**DR**	Middle SDI	Female	60-64 years	Smoking	2021	0.53	0.82	0.34

**DR**	Middle SDI	Female	60-64 years	Chewing tobacco	2021	0.38	0.60	0.20
**DALYR**	Middle SDI	Female	60-64 years	Smoking	2021	15.46	24.00	9.93
**DALYR**	Middle SDI	Female	60-64 years	Chewing tobacco	2021	10.99	17.63	5.74
**DALYR**	Middle SDI	Female	60-64 years	Diet low in vegetables	2021	26.71	54.92	-6.08
**DALYR**	Middle SDI	Female	60-64 years	Alcohol use	2021	6.35	9.79	3.57
**DR**	Middle SDI	Male	60-64 years	Alcohol use	2021	7.75	11.07	5.22
**DR**	Middle SDI	Male	60-64 years	Diet low in vegetables	2021	3.08	7.40	-0.65
**DR**	Middle SDI	Male	60-64 years	Smoking	2021	18.61	25.10	13.37
**DR**	Middle SDI	Male	60-64 years	Chewing tobacco	2021	1.17	1.84	0.66
**DALYR**	Middle SDI	Male	60-64 years	Smoking	2021	541.89	730.08	389.45
**DALYR**	Middle SDI	Male	60-64 years	Chewing tobacco	2021	34.19	53.65	19.40
**DALYR**	Middle SDI	Male	60-64 years	Diet low in vegetables	2021	89.79	215.90	-18.91
**DALYR**	Middle SDI	Male	60-64 years	Alcohol use	2021	225.65	322.75	151.80
**DR**	Middle SDI	Both	65-74 years	Alcohol use	1990	9.51	13.26	6.50
**DR**	Middle SDI	Both	65-74 years	Smoking	1990	35.02	43.23	27.13
**DR**	Middle SDI	Both	65-74 years	Chewing tobacco	1990	1.29	1.79	0.88
**DR**	Middle SDI	Both	65-74 years	Diet low in vegetables	1990	18.83	37.05	-4.00
**DALYR**	Middle SDI	Both	65-74 years	Smoking	1990	786.17	972.75	609.70
**DALYR**	Middle SDI	Both	65-74 years	Chewing tobacco	1990	29.09	40.45	19.83
**DALYR**	Middle SDI	Both	65-74 years	Diet low in vegetables	1990	422.86	833.75	-89.90
**DALYR**	Middle SDI	Both	65-74 years	Alcohol use	1990	215.15	301.72	146.51
**DR**	Middle SDI	Female	65-74 years	Alcohol use	1990	0.97	1.59	0.50
**DR**	Middle SDI	Female	65-74 years	Smoking	1990	5.12	7.53	2.64
**DR**	Middle SDI	Female	65-74 years	Chewing tobacco	1990	0.87	1.37	0.47
**DR**	Middle SDI	Female	65-74 years	Diet low in vegetables	1990	12.37	26.07	-2.37
**DALYR**	Middle SDI	Female	65-74 years	Smoking	1990	113.25	166.46	58.31
**DALYR**	Middle SDI	Female	65-74 years	Chewing tobacco	1990	19.58	31.13	10.44
**DALYR**	Middle SDI	Female	65-74 years	Diet low in vegetables	1990	276.23	582.71	-52.88
**DALYR**	Middle SDI	Female	65-74 years	Alcohol use	1990	21.59	35.85	11.00

**DR**	Middle SDI	Male	65-74 years	Alcohol use	1990	18.89	26.56	12.86
**DR**	Middle SDI	Male	65-74 years	Smoking	1990	67.84	85.16	52.20
**DR**	Middle SDI	Male	65-74 years	Chewing tobacco	1990	1.74	2.79	1.07
**DR**	Middle SDI	Male	65-74 years	Diet low in vegetables	1990	25.93	52.30	-6.07
**DALYR**	Middle SDI	Male	65-74 years	Smoking	1990	1525.04	1911.40	1172.43
**DALYR**	Middle SDI	Male	65-74 years	Chewing tobacco	1990	39.53	62.92	24.24
**DALYR**	Middle SDI	Male	65-74 years	Diet low in vegetables	1990	583.86	1181.77	-136.06
**DALYR**	Middle SDI	Male	65-74 years	Alcohol use	1990	427.67	602.83	291.32
**DR**	Middle SDI	Both	65-74 years	Alcohol use	2021	6.56	9.43	4.32
**DR**	Middle SDI	Both	65-74 years	Smoking	2021	20.57	27.48	15.06
**DR**	Middle SDI	Both	65-74 years	Chewing tobacco	2021	1.07	1.49	0.70
**DR**	Middle SDI	Both	65-74 years	Diet low in vegetables	2021	3.14	7.09	-0.62
**DALYR**	Middle SDI	Both	65-74 years	Smoking	2021	459.31	614.30	335.54
**DALYR**	Middle SDI	Both	65-74 years	Diet low in vegetables	2021	70.38	158.07	-13.91
**DALYR**	Middle SDI	Both	65-74 years	Alcohol use	2021	147.30	210.39	97.59
**DALYR**	Middle SDI	Both	65-74 years	Chewing tobacco	2021	24.03	33.75	15.63
**DR**	Middle SDI	Female	65-74 years	Alcohol use	2021	0.39	0.63	0.21
**DR**	Middle SDI	Female	65-74 years	Smoking	2021	1.29	2.05	0.76
**DR**	Middle SDI	Female	65-74 years	Chewing tobacco	2021	0.65	0.98	0.36
**DR**	Middle SDI	Female	65-74 years	Diet low in vegetables	2021	1.60	3.68	-0.35
**DALYR**	Middle SDI	Female	65-74 years	Smoking	2021	28.74	45.22	16.95
**DALYR**	Middle SDI	Female	65-74 years	Diet low in vegetables	2021	35.76	80.93	-7.69
**DALYR**	Middle SDI	Female	65-74 years	Alcohol use	2021	8.70	13.98	4.71
**DALYR**	Middle SDI	Female	65-74 years	Chewing tobacco	2021	14.69	22.13	8.00
**DR**	Middle SDI	Male	65-74 years	Alcohol use	2021	13.26	19.19	8.73
**DR**	Middle SDI	Male	65-74 years	Smoking	2021	41.51	55.61	30.20
**DR**	Middle SDI	Male	65-74 years	Chewing tobacco	2021	1.51	2.32	0.82
**DR**	Middle SDI	Male	65-74 years	Diet low in vegetables	2021	4.80	11.84	-0.93
**DALYR**	Middle SDI	Male	65-74 years	Smoking	2021	926.99	1244.58	675.04

**DALYR**	Middle SDI	Male	65-74 years	Diet low in vegetables	2021	108.00	259.44	-20.63
**DALYR**	Middle SDI	Male	65-74 years	Alcohol use	2021	297.84	428.05	196.82
**DALYR**	Middle SDI	Male	65-74 years	Chewing tobacco	2021	34.17	51.92	18.34
**DR**	Middle SDI	Both	75+ years	Alcohol use	1990	7.89	11.10	5.41
**DR**	Middle SDI	Both	75+ years	Smoking	1990	37.75	46.53	29.47
**DR**	Middle SDI	Both	75+ years	Chewing tobacco	1990	1.65	2.22	1.15
**DR**	Middle SDI	Both	75+ years	Diet low in vegetables	1990	23.36	47.14	-4.88
**DALYR**	Middle SDI	Both	75+ years	Smoking	1990	545.03	673.63	423.76
**DALYR**	Middle SDI	Both	75+ years	Diet low in vegetables	1990	334.49	672.33	-69.98
**DALYR**	Middle SDI	Both	75+ years	Alcohol use	1990	116.47	164.01	79.01
**DALYR**	Middle SDI	Both	75+ years	Chewing tobacco	1990	22.89	31.19	15.81
**DR**	Middle SDI	Female	75+ years	Alcohol use	1990	1.06	1.78	0.55
**DR**	Middle SDI	Female	75+ years	Smoking	1990	7.11	10.51	4.02
**DR**	Middle SDI	Female	75+ years	Chewing tobacco	1990	1.49	2.26	0.87
**DR**	Middle SDI	Female	75+ years	Diet low in vegetables	1990	17.58	35.91	-3.19
**DALYR**	Middle SDI	Female	75+ years	Smoking	1990	96.52	143.54	54.48
**DALYR**	Middle SDI	Female	75+ years	Diet low in vegetables	1990	248.66	505.51	-44.76
**DALYR**	Middle SDI	Female	75+ years	Alcohol use	1990	15.23	25.31	7.80
**DALYR**	Middle SDI	Female	75+ years	Chewing tobacco	1990	20.29	30.71	11.62
**DR**	Middle SDI	Male	75+ years	Alcohol use	1990	17.21	24.43	11.72
**DR**	Middle SDI	Male	75+ years	Smoking	1990	79.53	98.19	62.74
**DR**	Middle SDI	Male	75+ years	Chewing tobacco	1990	1.87	2.86	1.07
**DR**	Middle SDI	Male	75+ years	Diet low in vegetables	1990	31.23	62.96	-7.24
**DALYR**	Middle SDI	Male	75+ years	Smoking	1990	1156.67	1428.81	906.64
**DALYR**	Middle SDI	Male	75+ years	Diet low in vegetables	1990	451.53	905.40	-104.20
**DALYR**	Middle SDI	Male	75+ years	Alcohol use	1990	254.52	361.77	171.34
**DALYR**	Middle SDI	Male	75+ years	Chewing tobacco	1990	26.44	40.25	15.05
**DR**	Middle SDI	Both	75+ years	Alcohol use	2021	8.04	11.34	5.39
**DR**	Middle SDI	Both	75+ years	Smoking	2021	32.54	41.90	24.41

**DR**	Middle SDI	Both	75+ years	Chewing tobacco	2021	1.62	2.21	1.12
**DR**	Middle SDI	Both	75+ years	Diet low in vegetables	2021	7.34	16.34	-1.63
**DALYR**	Middle SDI	Both	75+ years	Diet low in vegetables	2021	95.11	215.07	-21.56
**DALYR**	Middle SDI	Both	75+ years	Alcohol use	2021	111.58	157.50	75.31
**DALYR**	Middle SDI	Both	75+ years	Smoking	2021	441.88	569.93	330.71
**DALYR**	Middle SDI	Both	75+ years	Chewing tobacco	2021	21.66	29.37	14.95
**DR**	Middle SDI	Female	75+ years	Alcohol use	2021	0.61	1.02	0.33
**DR**	Middle SDI	Female	75+ years	Smoking	2021	2.92	4.61	1.55
**DR**	Middle SDI	Female	75+ years	Chewing tobacco	2021	1.28	1.90	0.73
**DR**	Middle SDI	Female	75+ years	Diet low in vegetables	2021	4.27	10.15	-0.83
**DALYR**	Middle SDI	Female	75+ years	Diet low in vegetables	2021	54.17	129.51	-10.49
**DALYR**	Middle SDI	Female	75+ years	Alcohol use	2021	8.22	13.56	4.37
**DALYR**	Middle SDI	Female	75+ years	Smoking	2021	37.70	60.29	20.18
**DALYR**	Middle SDI	Female	75+ years	Chewing tobacco	2021	16.73	24.66	9.56
**DR**	Middle SDI	Male	75+ years	Alcohol use	2021	17.78	24.92	11.97
**DR**	Middle SDI	Male	75+ years	Smoking	2021	71.41	91.70	53.14
**DR**	Middle SDI	Male	75+ years	Chewing tobacco	2021	2.08	3.11	1.27
**DR**	Middle SDI	Male	75+ years	Diet low in vegetables	2021	11.36	27.20	-2.54
**DALYR**	Middle SDI	Male	75+ years	Diet low in vegetables	2021	148.85	353.10	-34.94
**DALYR**	Middle SDI	Male	75+ years	Alcohol use	2021	247.22	346.81	167.58
**DALYR**	Middle SDI	Male	75+ years	Smoking	2021	972.27	1247.23	720.04
**DALYR**	Middle SDI	Male	75+ years	Chewing tobacco	2021	28.13	42.45	17.12

Supplementary Table S13｜Result of decomposition analysis for the driving factor of EC deaths globally and among SDI quintiles, where all risk factors and individual factors were included. The risk-deleted effect for individual factors demonstrates the influences from both total risk-deleted effect and the effects from other individual risk factors.

(A) All risk factors

Location	Sex	Risks	Overall difference	Population	Aging	Risk effect	Risk-deleted effect
**Global**	Female	All risk factors	-1267.84(-3.41%)	21002.71(56.48%)	10180.6(27.38%)	-16444.27(-44.22%)	-16006.88 (-43.05%)
**Global**	Both	All risk factors	85339.25(40.84%)	136096.44(65.14%)	67655.18(32.38%)	-78903.53(-37.76%)	-39508.84 (-18.91%)
**Global**	Male	All risk factors	86607.09(50.42%)	114285.2(66.54%)	63784.25(37.14%)	-36173.35(-21.06%)	-55289 (-32.19%)
**Low SDI**	Female	All risk factors	1830.77(75.95%)	2955.84(122.62%)	-76.96(-3.19%)	-933.05(-38.71%)	-115.06 (-4.77%)
**Low SDI**	Both	All risk factors	5339.54(79.2%)	8234.49(122.13%)	-546.77(-8.11%)	-2058.99(-30.54%)	-289.19 (-4.29%)
**Low SDI**	Male	All risk factors	3508.78(81%)	5248.64(121.17%)	-549.05(-12.67%)	-928.58(-21.44%)	-262.23 (-6.05%)
**Low-middle SDI**	Female	All risk factors	3712.75(83.54%)	4516.36(101.62%)	1330.55(29.94%)	-1460.02(-32.85%)	-674.14 (-15.17%)
**Low-middle SDI**	Both	All risk factors	13127.3(93.52%)	14149.12(100.8%)	3468.04(24.71%)	-1728.37(-12.31%)	-2761.5 (-19.67%)
**Low-middle SDI**	Male	All risk factors	9414.54(98.15%)	9492.61(98.96%)	1879.54(19.59%)	1008.08(10.51%)	-2965.69 (-30.92%)
**Middle SDI**	Female	All risk factors	-4208.6(-30.8%)	7808.91(57.14%)	5918.9(43.31%)	-9720.45(-71.13%)	-8215.97 (-60.12%)
**Middle SDI**	Both	All risk factors	28174.91(33.96%)	58004.61(69.92%)	43406.41(52.32%)	-60341.37(-72.74%)	-12894.74 (-15.54%)
**Middle SDI**	Male	All risk factors	32383.51(46.74%)	48165.51(69.51%)	37723.02(54.44%)	-31777.22(-45.86%)	-21727.8 (-31.36%)
**High-middle SDI**	Female	All risk factors	-3970.43(-40.61%)	3011.84(30.8%)	2933.37(30%)	-3144.38(-32.16%)	-6771.26 (-69.25%)
**High-middle SDI**	Both	All risk factors	23739.77(34.97%)	29476.01(43.42%)	27248.21(40.13%)	-25762.92(-37.95%)	-7221.53 (-10.64%)
**High-middle SDI**	Male	All risk factors	27710.2(47.68%)	26987.71(46.44%)	28140.92(48.42%)	-15179.11(-26.12%)	-12239.31 (-21.06%)
**High SDI**	Female	All risk factors	1363.28(19.85%)	2268.8(33.03%)	2464.85(35.88%)	-1295.48(-18.86%)	-2074.9 (-30.21%)

**High SDI**	Both	All risk factors	14921.41(40.09%)	14119.94(37.94%)	14074.24(37.81%)	1091.62(2.93%)	-14364.38 (-38.59%)
**High SDI**	Male	All risk factors	13558.13(44.67%)	12633.79(41.63%)	14355.73(47.3%)	3953.72(13.03%)	-17385.1 (-57.28%)

(B) Individual factors

Location	Sex	Risks	Overall difference	Population	Aging	Risk effect	Risk-deleted effect
**Global**	Female	Diet low in vegetables	-5468.39(-23.73%)	11918.27(51.72%)	5556.15(24.11%)	-9933.82(-43.11%)	-13009.01 (-56.45%)
**Global**	Both	Diet low in vegetables	-17514.63(-23.52%)	38574.73(51.81%)	18953.4(25.46%)	-18878.05(-25.36%)	-56164.7 (-75.44%)
**Global**	Male	Diet low in vegetables	-12046.23(-23.43%)	26608.56(51.76%)	14419.18(28.05%)	-10388.21(-20.21%)	-42685.76 (-83.03%)
**Low SDI**	Female	Diet low in vegetables	1367.75(76.61%)	2193.63(122.87%)	-56.86(-3.19%)	-709.11(-39.72%)	-59.91 (-3.36%)
**Low SDI**	Both	Diet low in vegetables	3202.07(78.84%)	4954.79(122%)	-320.09(-7.88%)	-1262.82(-31.09%)	-169.81 (-4.18%)
**Low SDI**	Male	Diet low in vegetables	1834.32(80.59%)	2754.44(121.02%)	-283.91(-12.47%)	-525.65(-23.1%)	-110.57 (-4.86%)
**Low-middle SDI**	Female	Diet low in vegetables	2294.17(88.6%)	2669.01(103.08%)	776.78(30%)	-993.9(-38.38%)	-157.72 (-6.09%)
**Low-middle SDI**	Both	Diet low in vegetables	6028.84(98.74%)	6235.86(102.13%)	1534.23(25.13%)	-1302.84(-21.34%)	-438.41 (-7.18%)
**Low-middle SDI**	Male	Diet low in vegetables	3734.67(106.21%)	3537.7(100.61%)	702(19.96%)	-229.37(-6.52%)	-275.65 (-7.84%)
**Middle SDI**	Female	Diet low in vegetables	-5054.41(-51.52%)	5044.84(51.42%)	3757.58(38.3%)	-6203.65(-63.24%)	-7653.18 (-78.01%)
**Middle SDI**	Both	Diet low in vegetables	-14369.35(-45.62%)	16238.64(51.56%)	12064.66(38.3%)	-13220.27(-41.97%)	-29452.38 (-93.51%)
**Middle SDI**	Male	Diet low in vegetables	-9314.94(-42.95%)	10994.02(50.7%)	8383.1(38.66%)	-7027.26(-32.4%)	-21664.81 (-99.90%)
**High-middle SDI**	Female	Diet low in vegetables	-4598.72(-74.44%)	1562.84(25.3%)	1483.83(24.02%)	-1701.57(-27.54%)	-5943.82 (-96.21%)
**High-middle SDI**	Both	Diet low in vegetables	-15731.53(-70.1%)	6275.65(27.97%)	5931.71(26.43%)	-2861.05(-12.75%)	-25077.85 (-111.75%)
**High-middle SDI**	Male	Diet low in vegetables	-11132.81(-68.45%)	4804.96(29.55%)	5005.21(30.78%)	-1765.37(-10.86%)	-19177.61 (-117.92%)
**High SDI**	Female	Diet low in vegetables	520.31(19.47%)	879.77(32.92%)	982.81(36.78%)	-756.59(-28.31%)	-585.68 (-21.92%)

**High SDI**	Both	Diet low in vegetables	3344.32(32.41%)	3800.46(36.83%)	3931.31(38.1%)	-1589.44(-15.4%)	-2798.01 (-27.12%)
**High SDI**	Male	Diet low in vegetables	2824.01(36.94%)	3059.55(40.02%)	3508.17(45.88%)	-1547.26(-20.24%)	-2196.46 (-28.73%)
**Global**	Female	Smoking	286.99(2.41%)	6886.34(57.89%)	3684.22(30.97%)	-5992.52(-50.38%)	-4291.04 (-36.07%)
**Global**	Both	Smoking	69012.58(50.58%)	91841.01(67.31%)	46832.41(34.32%)	-65388.39(-47.92%)	-4272.45 (-3.13%)
**Global**	Male	Smoking	68725.59(55.18%)	84026.32(67.46%)	48579.23(39%)	-39166.08(-31.44%)	-24713.89 (-19.84%)
**Low SDI**	Female	Smoking	124.46(44.8%)	312.36(112.43%)	-7.96(-2.86%)	-92.26(-33.21%)	-87.68 (-31.56%)
**Low SDI**	Both	Smoking	1006.83(53.4%)	2147.68(113.92%)	-152.35(-8.08%)	-505.91(-26.83%)	-482.57 (-25.60%)
**Low SDI**	Male	Smoking	882.37(54.89%)	1816.9(113.03%)	-196.34(-12.21%)	-288.88(-17.97%)	-449.3 (-27.95%)
**Low-middle SDI**	Female	Smoking	228.82(33.72%)	601.94(88.72%)	191.3(28.19%)	-194.78(-28.71%)	-369.63 (-54.48%)
**Low-middle SDI**	Both	Smoking	3950.73(63.48%)	5814.89(93.43%)	1477.52(23.74%)	-699.5(-11.24%)	-2642.19 (-42.46%)
**Low-middle SDI**	Male	Smoking	3721.9(67.12%)	5092.07(91.83%)	1057.97(19.08%)	582.46(10.5%)	-3010.59 (-54.29%)
**Middle SDI**	Female	Smoking	-481.79(-13.93%)	2155.23(62.31%)	1738.39(50.26%)	-3063.74(-88.58%)	-1311.68 (-37.92%)
**Middle SDI**	Both	Smoking	27986.71(51.27%)	40730.32(74.62%)	31186.73(57.13%)	-49475.6(-90.64%)	5545.26 (10.16%)
**Middle SDI**	Male	Smoking	28468.5(55.68%)	36631.28(71.65%)	29596.06(57.89%)	-30575.19(-59.8%)	-7183.65 (-14.05%)
**High-middle SDI**	Female	Smoking	74.83(2.29%)	1236.72(37.93%)	1277.82(39.19%)	-1822.7(-55.9%)	-617.02 (-18.92%)
**High-middle SDI**	Both	Smoking	28337.35(61.42%)	22174.73(48.07%)	20807.21(45.1%)	-28448.63(-61.67%)	13804.04 (29.92%)
**High-middle SDI**	Male	Smoking	28262.52(65.92%)	21253.21(49.57%)	22760.81(53.09%)	-20926.03(-48.81%)	5174.54 (12.07%)
**High SDI**	Female	Smoking	339.96(8.07%)	1330.44(31.59%)	1444.28(34.29%)	-910.17(-21.61%)	-1524.6 (-36.20%)
**High SDI**	Both	Smoking	7715.68(27.99%)	10038.83(36.42%)	10085.19(36.59%)	185.69(0.67%)	-12594.04 (-45.69%)
**High SDI**	Male	Smoking	7375.72(31.59%)	9321.64(39.92%)	10845.82(46.45%)	1960.64(8.4%)	-14752.38 (-63.18%)
**Global**	Female	Alcohol use	1025.45(27.09%)	2420.74(63.96%)	1177.73(31.12%)	-2542.41(-67.18%)	-30.6 (-0.81%)

**Global**	Both	Alcohol use	29603.26(57.34%)	35745.7(69.24%)	16492.05(31.95%)	-31071.58(-60.19%)	8437.09 (16.34%)
**Global**	Male	Alcohol use	28577.81(59.74%)	32930.69(68.84%)	16795.58(35.11%)	-24621.77(-51.47%)	3473.3 (7.26%)
**Low SDI**	Female	Alcohol use	206.52(107.57%)	256.06(133.38%)	-9.14(-4.76%)	-93.85(-48.89%)	53.44 (27.84%)
**Low SDI**	Both	Alcohol use	1243.16(122.49%)	1384.68(136.43%)	-100.45(-9.9%)	-420(-41.38%)	378.93 (37.34%)
**Low SDI**	Male	Alcohol use	1036.64(125.97%)	1116.44(135.67%)	-119.29(-14.5%)	-262.58(-31.91%)	302.07 (36.71%)
**Low-middle SDI**	Female	Alcohol use	216.17(173.91%)	157.17(126.45%)	40.49(32.57%)	-67.49(-54.3%)	86 (69.19%)
**Low-middle SDI**	Both	Alcohol use	2731.19(189.76%)	1811.03(125.83%)	383.86(26.67%)	-534.79(-37.16%)	1071.09 (74.42%)
**Low-middle SDI**	Male	Alcohol use	2515.02(191.25%)	1600.51(121.71%)	279.54(21.26%)	-260.57(-19.82%)	895.54 (68.10%)
**Middle SDI**	Female	Alcohol use	109.98(13.44%)	578.39(70.7%)	405.56(49.58%)	-974.45(-119.12%)	100.47 (12.28%)
**Middle SDI**	Both	Alcohol use	10896.61(58.85%)	14311.35(77.29%)	9836.36(53.13%)	-19420.63(-104.89%)	6169.53 (33.32%)
**Middle SDI**	Male	Alcohol use	10786.63(60.95%)	13065.98(73.83%)	9356.45(52.87%)	-14860.61(-83.97%)	3224.82 (18.22%)
**High-middle SDI**	Female	Alcohol use	-140.61(-11.83%)	426.87(35.9%)	384.78(32.36%)	-767.84(-64.58%)	-184.43 (-15.51%)
**High-middle SDI**	Both	Alcohol use	8796.79(45.62%)	8771.37(45.49%)	7436.91(38.57%)	-10949.41(-56.78%)	3537.91 (18.35%)
**High-middle SDI**	Male	Alcohol use	8937.4(49.4%)	8507.6(47.02%)	8082.44(44.67%)	-8776.22(-48.5%)	1123.57 (6.21%)
**High SDI**	Female	Alcohol use	632.51(43.36%)	526.09(36.06%)	526.8(36.11%)	-562.56(-38.56%)	142.18 (9.75%)
**High SDI**	Both	Alcohol use	5921.18(52.2%)	4453.02(39.26%)	4064.23(35.83%)	-3152.72(-27.79%)	556.66 (4.91%)
**High SDI**	Male	Alcohol use	5288.67(53.5%)	4164.74(42.13%)	4313.52(43.64%)	-3023.4(-30.59%)	-166.19 (-1.68%)
**Global**	Female	Chewing tobacco	2662.4(83.48%)	2491.1(78.11%)	1111.22(34.84%)	-3000.76(-94.09%)	2060.85 (64.62%)
**Global**	Both	Chewing tobacco	8214.19(84.46%)	7381.03(75.89%)	3433.53(35.3%)	-6400.61(-65.81%)	3800.24 (39.08%)
**Global**	Male	Chewing tobacco	5551.79(84.94%)	4915.59(75.21%)	2462.95(37.68%)	-3837.86(-58.72%)	2011.1 (30.77%)
**Low SDI**	Female	Chewing tobacco	298.28(72.91%)	497.3(121.55%)	-10.93(-2.67%)	-157.01(-38.38%)	-31.07 (-7.60%)

**Low SDI**	Both	Chewing tobacco	816.48(74.09%)	1327.6(120.47%)	-84.84(-7.7%)	-341.33(-30.97%)	-84.95 (-7.71%)
**Low SDI**	Male	Chewing tobacco	518.19(74.78%)	826.42(119.26%)	-85.08(-12.28%)	-157.59(-22.74%)	-65.55 (-9.46%)
**Low-middle SDI**	Female	Chewing tobacco	1430.57(81.01%)	1781.15(100.86%)	530.71(30.05%)	-639.23(-36.2%)	-242.06 (-13.71%)
**Low-middle SDI**	Both	Chewing tobacco	3636.4(86.14%)	4169.26(98.76%)	1010.74(23.94%)	-819.91(-19.42%)	-723.68 (-17.14%)
**Low-middle SDI**	Male	Chewing tobacco	2205.83(89.83%)	2368.91(96.47%)	447.83(18.24%)	-125.43(-5.11%)	-485.48 (-19.77%)
**Middle SDI**	Female	Chewing tobacco	843.98(105.15%)	789.59(98.38%)	600.01(74.76%)	-1391.86(-173.42%)	846.24 (105.44%)
**Middle SDI**	Both	Chewing tobacco	2685.34(101.15%)	2350.39(88.53%)	1677.85(63.2%)	-3092.97(-116.5%)	1750.08 (65.92%)
**Middle SDI**	Male	Chewing tobacco	1841.36(99.41%)	1551.07(83.74%)	1084.08(58.53%)	-1821.13(-98.32%)	1027.34 (55.46%)
**High-middle SDI**	Female	Chewing tobacco	64.29(41.79%)	69.3(45.04%)	63.34(41.17%)	-128.72(-83.67%)	60.37 (39.25%)
**High-middle SDI**	Both	Chewing tobacco	535.77(64.6%)	403.59(48.66%)	339.18(40.9%)	-508.53(-61.32%)	301.53 (36.36%)
**High-middle SDI**	Male	Chewing tobacco	471.48(69.8%)	341.04(50.49%)	303.49(44.93%)	-391.07(-57.89%)	218.02 (32.27%)
**High SDI**	Female	Chewing tobacco	24.83(43.45%)	20.65(36.14%)	22.77(39.84%)	-20.67(-36.17%)	2.08 (3.64%)
**High SDI**	Both	Chewing tobacco	538.81(58.83%)	371.89(40.61%)	375.77(41.03%)	-220.27(-24.05%)	11.42 (1.25%)
**High SDI**	Male	Chewing tobacco	513.99(59.86%)	377.25(43.93%)	453.58(52.82%)	-227.13(-26.45%)	-89.72 (-10.45%)

Supplementary Table S14 | The BAPC prediction results (predicted values and 95% uncertainty intervals) for ASRs of esophageal cancer in world and seven GBD super regions from 2022 to 2035.

(1) ASDALYRs, both sexes

Years	Central Europe, Eastern Europe, and Central Asia			High-income			Latin America and Caribbean			North Africa and Middle East			South Asia			Southeast Asia, East Asia, and Oceania			Sub-Saharan Africa			Global		
	pred	lower UI	upper UI	pred	lower UI	upper UI	pred	lower UI	upper UI	pred	lower UI	upper UI	pred	lower UI	upper UI	pred	lower UI	upper UI	pred	lower UI	upper UI	pred	lower UI	upper UI
2022	81.84973	75.58345	88.116	81.1279	75.84522	86.41058	80.97077	75.8014	86.14014	47.10477	44.85701	49.35253	90.91861	81.22821	100.609	255.8612	221.0087	290.7137	205.7632	194.1606	217.3658	147.2836	136.9947	157.5726
2023	81.31436	73.50481	89.12392	79.41896	73.63447	85.20345	80.52204	74.68114	86.36294	46.55079	44.02939	49.0722	90.71696	80.38713	101.0468	254.675	216.8136	292.5363	204.2629	191.5085	217.0173	146.6059	134.9831	158.2288
2024	80.80802	70.64844	90.9676	77.77361	71.15058	84.39664	80.08827	73.14504	87.0315	46.02109	43.06562	48.97655	90.52138	79.16977	101.873	253.4714	210.7234	296.2194	202.7668	188.1455	217.3881	145.8782	132.1166	159.6398
2025	80.33297	67.19835	93.46759	76.17845	68.42821	83.9287	79.64934	71.22467	88.07402	45.47932	41.95353	49.00512	90.34066	77.58388	103.0974	252.2525	202.8012	301.7038	201.2772	184.1298	218.4246	145.0861	128.4717	161.7006
2026	79.92529	63.30963	96.54094	74.65224	65.54437	83.7601	79.23325	69.0044	89.4621	44.95831	40.74654	49.17008	90.18004	75.66255	104.6975	251.1271	193.3248	308.9294	199.8036	179.5697	220.0375	144.3441	124.2627	164.4256
2027	79.63701	59.08604	100.188	73.19615	62.55154	83.84075	78.85457	66.54226	91.16687	44.46437	39.46822	49.46052	90.04357	73.44548	106.6417	250.1498	182.5425	317.7571	198.3823	174.5943	222.1702	143.6834	119.6075	167.7594
2028	79.44687	54.54983	104.3439	71.82792	59.50901	84.14684	78.5321	63.89446	93.16974	44.00665	38.15218	49.86113	89.92172	70.96311	108.8803	249.3555	170.6779	328.0332	197.0336	169.3099	224.7574	143.1004	114.6073	171.5936
2029	79.32791	49.69176	108.9641	70.54154	56.4393	84.64379	78.26371	61.08165	95.44577	43.57081	36.79976	50.34187	89.80902	68.24089	111.3771	248.7295	157.8264	339.6326	195.7457	163.7653	227.7261	142.5216	109.2595	175.7836
2030	79.28405	44.502	114.0661	69.32203	53.34667	85.29738	78.03198	58.10058	97.96338	43.13013	35.39559	50.86468	89.71196	65.30505	114.1189	248.2639	144.0169	352.511	194.5162	157.9961	231.0363	141.9085	103.5669	180.25
2031	79.34791	38.96519	119.7306	68.18578	50.25189	86.11966	77.86036	54.97174	100.749	42.71913	33.97015	51.46811	89.63517	62.17224	117.0981	248.0961	129.3038	366.8884	193.3625	152.0385	234.6865	141.3943	97.63349	185.1552
2032	79.57186	33.0493	126.0944	67.13318	47.15784	87.10851	77.75683	51.69312	103.8205	42.34241	32.52563	52.15919	89.58287	58.85266	120.3131	248.3343	113.6669	383.0016	192.3226	145.9348	238.7104	141.0594	91.49969	190.619
2033	79.94349	26.68285	133.2041	66.17289	44.07486	88.27092	77.73821	48.26887	107.2075	41.99801	31.06805	52.92798	89.54442	55.34626	123.7426	249.0447	97.04408	401.0453	191.4184	139.7167	243.12	140.9173	85.1823	196.6524
2034	80.44373	19.78482	141.1168	65.29456	40.99487	89.59425	77.81007	44.68477	110.9354	41.6717	29.58984	53.75357	89.51661	51.6533	127.3799	250.1812	79.25406	421.1084	190.6377	133.3793	247.896	140.8638	78.60499	203.1227
2035	81.07715	13.62868	149.9559	64.48181	37.90323	91.06039	77.95919	40.90806	115.0103	41.34616	28.07901	54.61331	89.50899	47.77647	131.2415	251.6953	60.12674	443.3263	189.9762	126.9162	253.0363	140.8213	71.70653	209.9361

(2) ASDALYRs, male

Years	Central Europe, Eastern Europe, and Central Asia			High-income			Latin America and Caribbean			North Africa and Middle East			South Asia			Southeast Asia, East Asia, and Oceania			Sub-Saharan Africa			Global		
	pred	lower UI	upper UI	pred	lower UI	upper UI	pred	lower UI	upper UI	pred	lower UI	upper UI	pred	lower UI	upper UI	pred	lower UI	upper UI	pred	lower UI	upper UI	pred	lower UI	upper UI
2022	146.8036	140.3245	153.2828	137.7074	133.2402	142.1745	135.9148	131.0982	140.7315	56.96643	55.79379	58.13907	108.297	102.825	113.769	438.0453	410.4501	465.6406	264.4956	257.4988	271.4924	236.7457	228.3668	245.1245
2023	144.9807	134.8025	155.159	134.6178	128.4491	140.7865	135.0098	128.1473	141.8723	56.50018	54.68342	58.31693	108.8023	101.9903	115.6143	437.5511	400.4525	474.6497	263.1285	253.5064	272.7506	235.8774	223.6945	248.0603
2024	143.1345	128.2817	157.9874	131.625	123.2691	139.981	134.0964	125.0332	143.1597	56.05299	53.43988	58.6661	109.2957	100.6743	117.9172	436.7532	387.0723	486.4341	261.755	248.6973	274.8126	234.828	217.7217	251.9343
2025	141.2455	121.0709	161.4201	128.7083	117.8533	139.5634	133.1348	121.6804	144.5893	55.57758	52.06138	59.09378	109.7746	98.95734	120.592	435.6586	371.0932	500.224	260.3836	243.2966	277.4706	233.587	210.7585	256.4155
2026	139.3917	113.3953	165.3881	125.8952	112.3223	139.468	132.1739	118.0671	146.2808	55.11763	50.60641	59.62885	110.2451	96.90717	123.583	434.4338	353.1225	515.745	259.0204	237.4312	280.6095	232.3447	203.1301	261.5593
2027	137.6686	105.4112	169.926	123.189	106.7344	139.6436	131.2345	114.1982	148.2708	54.67612	49.08413	60.2681	110.7066	94.56625	126.8469	433.1459	333.508	532.7838	257.7054	231.2101	284.2007	231.1344	194.9408	267.3279
2028	136.0149	97.14137	174.8885	120.6171	101.1585	140.0756	130.3433	110.1267	150.56	54.26099	47.52033	61.00166	111.147	91.95797	130.336	431.7555	312.4936	551.0174	256.4736	224.728	288.2192	229.928	186.2884	273.5676
2029	134.3335	88.57363	180.0933	118.1646	95.60957	140.7196	129.4808	105.865	153.0966	53.85547	45.91078	61.80015	111.5556	89.09723	134.014	430.1612	290.1799	570.1425	255.296	217.9984	292.5935	228.5838	177.1293	280.0383
2030	132.5991	79.74427	185.4538	115.806	90.08429	141.5278	128.6111	101.4086	155.8136	53.427	44.23405	62.61996	111.9303	85.99944	137.8612	428.3324	266.6692	589.9956	254.1756	211.0478	297.3033	227.0393	167.4693	286.6092
2031	130.8704	70.72665	191.0141	113.5637	84.61004	142.5174	127.7755	96.80953	158.7416	53.0243	42.53179	63.51681	112.2766	82.68106	141.8721	426.4788	242.176	610.7817	253.131	203.9035	302.3586	225.5231	157.5032	293.543
2032	129.2289	61.58147	196.8764	111.4372	79.19125	143.6832	126.9812	92.08237	161.8801	52.64743	40.80086	64.49401	112.593	79.15147	146.0345	424.7543	216.8373	632.6713	252.2034	196.6049	307.8019	224.1579	147.3112	301.0046
2033	127.6334	52.31415	202.9526	109.4386	73.84642	145.0308	126.2493	87.25895	165.2397	52.28583	39.04172	65.52993	112.8648	75.41324	150.3163	423.188	190.7545	655.6215	251.4265	189.1944	313.6586	222.9602	136.9469	308.9735
2034	126.0019	42.92081	209.0829	107.5473	68.56612	146.5285	125.5718	82.34357	168.8001	51.9228	37.24794	66.59766	113.0837	71.47286	154.6946	421.616	163.9048	679.3272	250.7758	181.6601	319.8914	221.7288	126.3107	317.1468
2035	124.298	33.42578	215.1701	105.7345	63.33419	148.1347	124.9175	77.32241	172.5126	51.54125	35.41129	67.67121	113.2516	67.34181	159.1613	419.9008	136.2834	703.5182	250.251	174.0024	326.4996	220.3167	115.3492	325.2841

(3) ASDALYRs, female

Years	Central Europe, Eastern Europe, and Central Asia			High-income			Latin America and Caribbean			North Africa and Middle East			South Asia			Southeast Asia, East Asia, and Oceania			Sub-Saharan Africa			Global		
	pred	lower UI	upper UI	pred	lower UI	upper UI	pred	lower UI	upper UI	pred	lower UI	upper UI	pred	lower UI	upper UI	pred	lower UI	upper UI	pred	lower UI	upper UI	pred	lower UI	upper UI
2022	31.44602	30.15153	32.74051	30.29502558	29.49310972	31.09694144	32.87231	31.80542	33.93921	37.33601	36.41709	38.25493	73.6498	70.58623	76.71337	88.4541	83.0668	93.8414	152.8577	148.6444	157.071	66.28033	64.23878	68.32189
2023	31.68389	29.22999	34.1378	29.73574939	28.51944671	30.95205207	32.83797	30.8977	34.77823	36.70418	35.38783	38.02053	72.72413	68.46205	76.98622	86.97838	79.14251	94.81426	151.3677	145.3579	157.3775	65.81254	62.71214	68.91295
2024	31.91719	28.01535	35.81904	29.18866857	27.45665362	30.92068352	32.80766	29.78676	35.82856	36.09017	34.28348	37.89686	71.795	65.98372	77.60628	85.509	74.53383	96.48418	149.8555	141.5335	158.1774	65.35594	60.89992	69.81197
2025	32.14881	26.56242	37.73521	28.6476085	26.33417065	30.96104636	32.77245	28.51076	37.03415	35.46856	33.1099	37.82721	70.87791	63.27502	78.4808	84.03469	69.46941	98.59997	148.3118	137.3194	159.3042	64.89224	58.87135	70.91313
2026	32.36934	24.88677	39.85192	28.12104977	25.17730494	31.06479461	32.73555	27.09509	38.37601	34.86004	31.90046	37.81963	69.97237	60.39798	79.54675	82.58272	64.10093	101.0645	146.7414	132.8059	160.6769	64.45289	56.69671	72.20907

2027	32.59011	23.00893	42.17129	27.60548047	23.99289504	31.2180659	32.70005	25.55564	39.84446	34.27445	30.67132	37.87758	69.08154	57.39176	80.77133	81.1645	58.51363	103.8154	145.1699	128.0686	162.2711	64.05557	54.41184	73.6993
2028	32.7996	20.93602	44.66318	27.10410903	22.79339858	31.41481947	32.66897	23.90637	41.43156	33.7204	29.44318	37.99763	68.19099	54.27288	82.10911	79.79495	52.7741	106.8158	143.5986	123.1512	164.046	63.69383	52.03222	75.35544
2029	32.9949	18.67864	47.31116	26.61376239	21.58274944	31.64477534	32.64557	22.15782	43.13332	33.18268	28.20948	38.15589	67.29202	51.05566	83.52839	78.46334	46.91258	110.0141	142.021	118.0784	165.9636	63.3422	49.55046	77.13394
2030	33.18621	16.25177	50.12066	26.12894716	20.36140613	31.8964882	32.62177	20.3107	44.93283	32.63927	26.95533	38.3232	66.39284	47.76372	85.02196	77.16161	40.95288	113.3703	140.4235	112.8622	167.9848	62.98916	46.96791	79.01041
2031	33.36884	13.65827	53.07942	25.65836008	19.13944552	32.17727463	32.59824	18.37069	46.82579	32.10901	25.69772	38.5203	65.49042	44.40811	86.57274	75.91706	34.93007	116.904	138.8136	107.5269	170.1004	62.66786	44.31455	81.02118
2032	33.54864	10.90277	56.1945	25.20026138	17.91749561	32.48302714	32.576	16.34188	48.81013	31.60101	24.44364	38.75838	64.58731	41.00115	88.17346	74.73882	28.85959	120.6181	137.2179	102.1074	172.3284	62.39693	41.60276	83.19109
2033	33.7076	7.987783	59.42742	24.75592857	16.70003673	32.81182041	32.55828	14.23035	50.88621	31.1213	23.20328	39.03932	63.67161	37.54872	89.7945	73.63398	22.75445	124.5135	135.643	96.62537	174.6607	62.15902	38.82634	85.49169
2034	33.84715	4.923734	62.77057	24.32075538	15.48663888	33.15487189	32.55119	12.04162	53.06077	30.65555	21.96867	39.34244	62.73872	34.06046	91.41698	72.58809	16.61537	128.5608	134.0794	91.08697	177.0718	61.92788	35.97357	87.88219
2035	33.98054	1.720164	66.24092	23.88921934	14.27611725	33.50232143	32.54791	9.774865	55.32096	30.18528	20.72819	39.64237	61.79809	30.55247	93.04371	71.59	10.44397	132.736	132.5106	85.49229	179.5289	61.69548	33.04442	90.34654

(4) ASDRs, both

Years	Central Europe, Eastern Europe, and Central Asia			High-income			Latin America and Caribbean			North Africa and Middle East			South Asia			Southeast Asia, East Asia, and Oceania			Sub-Saharan Africa			Global		
	pred	lower UI	upper UI	pred	lower UI	upper UI	pred	lower UI	upper UI	pred	lower UI	upper UI	pred	lower UI	upper UI	pred	lower UI	upper UI	pred	lower UI	upper UI	pred	lower UI	upper UI
2022	3.032634	2.763748	3.301672	3.634351	3.1801	4.088602	3.203112	2.946299	3.459926	2.075269	1.83353	2.317008	3.52668	3.235816	3.817543	11.62437	10.41574	12.833	8.439579	7.897266	8.981892	6.256879	5.903431	6.610327
2023	3.002128	2.688732	3.315699	3.583084	3.120314	4.04586	3.172818	2.901358	3.444278	2.053478	1.804081	2.302874	3.511692	3.195336	3.828049	11.62426	10.2322	13.01632	8.40404	7.802752	9.005328	6.245725	5.823815	6.667635
2024	2.974127	2.594304	3.354183	3.532497	3.057531	4.007475	3.143063	2.847425	3.438701	2.032284	1.769137	2.295431	3.497045	3.140249	3.853841	11.62241	9.941274	13.30354	8.381759	7.623115	9.140404	6.233117	5.705469	6.760764
2025	2.946904	2.482915	3.411185	3.483325	2.988303	3.978367	3.113493	2.784426	3.44256	2.011435	1.7282	2.29467	3.483649	3.072083	3.895214	11.61893	9.555267	13.68259	8.352244	7.461089	9.243398	6.219227	5.555182	6.883272
2026	2.919723	2.357189	3.482611	3.434734	2.911233	3.958262	3.084093	2.713246	3.45494	1.991109	1.681663	2.300556	3.471209	2.992255	3.950162	11.61659	9.09079	14.1424	8.31414	7.306634	9.321645	6.205733	5.379797	7.03167
2027	2.895066	2.221312	3.569245	3.385201	2.825097	3.94534	3.055141	2.635267	3.475015	1.971428	1.630207	2.312649	3.459643	2.902355	4.016931	11.61671	8.55982	14.6736	8.273565	7.138844	9.408285	6.192094	5.1821	7.202088
2028	2.874869	2.078949	3.671294	3.336967	2.732823	3.941152	3.027211	2.55233	3.502092	1.95263	1.575017	2.330244	3.448697	2.804154	4.09324	11.62076	7.973127	15.2684	8.232817	6.955422	9.510212	6.178599	4.967134	7.390064
2029	2.858086	1.930058	3.786705	3.29148	2.63651	3.946501	3.000403	2.465441	3.535365	1.934799	1.51684	2.352759	3.438396	2.698886	4.177906	11.63081	7.336419	15.9252	8.19266	6.757384	9.627935	6.16514	4.73718	7.593099
2030	2.843508	1.773861	3.913847	3.248306	2.536403	3.960269	2.974466	2.374949	3.573983	1.91775	1.455891	2.379608	3.42931	2.587595	4.271025	11.6487	6.651099	16.64631	8.153533	6.545956	9.76111	6.152436	4.493809	7.811062
2031	2.830833	1.609826	4.052677	3.207016	2.432567	3.981535	2.949341	2.281178	3.617505	1.901671	1.392556	2.410785	3.421217	2.470461	4.371974	11.67928	5.916943	17.44162	8.115512	6.322134	9.908889	6.143339	4.238793	8.047885
2032	2.821682	1.438348	4.206233	3.166363	2.324389	4.00842	2.925225	2.184581	3.665873	1.886666	1.327112	2.446219	3.414209	2.347716	4.480703	11.72476	5.129815	18.3197	8.079298	6.087373	10.07122	6.13796	3.971137	8.304783
2033	2.817244	1.259366	4.376801	3.127906	2.213884	4.042024	2.902622	2.085956	3.719318	1.872911	1.260118	2.485767	3.408062	2.219618	4.596507	11.78702	4.285879	19.28822	8.045752	5.843618	10.24789	6.136534	3.692034	8.581035
2034	2.816686	1.071238	4.564337	3.092618	2.10223	4.083115	2.88167	1.985549	3.777847	1.860439	1.191731	2.529339	3.402712	2.086276	4.719149	11.86712	3.378173	20.35705	8.015217	5.591749	10.43868	6.138107	3.400787	8.875428
2035	2.819269	0.872092	4.76928	3.060186	1.989341	4.131157	2.862196	1.883222	3.841254	1.849076	1.12173	2.576758	3.39857	1.947834	4.849307	11.96635	2.395079	21.54009	7.987856	5.331978	10.64373	6.142595	3.096462	9.188728

(5) ASDRs, male

Years	Central Europe, Eastern Europe, and Central Asia			High-income			Latin America and Caribbean			North Africa and Middle East			South Asia			Southeast Asia, East Asia, and Oceania			Sub-Saharan Africa			Global		
	pred	lower UI	upper UI	pred	lower UI	upper UI	pred	lower UI	upper UI	pred	lower UI	upper UI	pred	lower UI	upper UI	pred	lower UI	upper UI	pred	lower UI	upper UI	pred	lower UI	upper UI
2022	5.5361	5.327394	5.744806	6.260133	5.764308	6.755958	5.304553	5.168287	5.440819	2.560783	2.471391	2.650175	4.21926	4.070802	4.367717	19.75055	18.7284	20.77269	10.58078	10.18794	10.97362	10.13171	9.821835	10.44158
2023	5.447566	5.097153	5.797979	6.167031	5.540214	6.793849	5.261209	5.063226	5.459192	2.545847	2.421403	2.670292	4.237291	4.031802	4.44278	19.82645	18.30258	21.35032	10.58644	9.982949	11.18994	10.13434	9.649059	10.61963
2024	5.361012	4.84294	5.879083	6.073839	5.362606	6.785072	5.217739	4.945681	5.489796	2.530901	2.364616	2.697186	4.254837	3.97636	4.533315	19.88668	17.71449	22.05886	10.58933	9.77212	11.40653	10.13023	9.423039	10.83741
2025	5.272164	4.568645	5.975683	5.982564	5.208678	6.75645	5.173499	4.818209	5.528788	2.515646	2.302326	2.728966	4.272058	3.908089	4.636026	19.93388	17.00278	22.86497	10.58498	9.572268	11.59769	10.12102	9.157548	11.0845
2026	5.179337	4.276754	6.08192	5.891511	5.058733	6.724288	5.128445	4.682422	5.574469	2.500345	2.235612	2.765078	4.288935	3.828865	4.749004	19.97166	16.18819	23.75512	10.57431	9.37069	11.77792	10.10894	8.859323	11.35856
2027	5.089507	3.975218	6.203796	5.797711	4.901193	6.694229	5.083146	4.539939	5.626354	2.485224	2.165236	2.805211	4.305491	3.739832	4.871151	20.00073	15.28158	24.71988	10.56012	9.155418	11.96483	10.09265	8.528927	11.65638
2028	5.006268	3.669876	6.34266	5.705328	4.737011	6.673645	5.038477	4.392781	5.684173	2.470417	2.092008	2.848825	4.321488	3.6418	5.001176	20.01608	14.29184	25.74032	10.54435	8.922257	12.16645	10.07046	8.170968	11.96996
2029	4.925332	3.35935	6.491313	5.616998	4.568137	6.665859	4.994355	4.241631	5.74708	2.455841	2.016249	2.895434	4.336645	3.535211	5.138079	20.01679	13.22697	26.80661	10.52755	8.670408	12.3847	10.0415	7.788295	12.29471
2030	4.84353	3.042989	6.644072	5.532084	4.39438	6.669788	4.950284	4.086523	5.814045	2.441225	1.937939	2.944512	4.351107	3.420622	5.281592	20.00676	12.09531	27.91821	10.51055	8.401101	12.61999	10.00813	7.384898	12.63137
2031	4.760061	2.7214	6.798722	5.449487	4.215305	6.683669	4.906125	3.927644	5.884606	2.426829	1.857386	2.996271	4.364859	3.298281	5.431437	19.99249	10.90395	29.08103	10.49337	8.115233	12.8715	9.974851	6.964474	12.98523
2032	4.679159	2.397546	6.960772	5.366605	4.029449	6.703762	4.862278	3.765604	5.958951	2.412839	1.774835	3.050844	4.377868	3.168392	5.587343	19.975	9.655286	30.29471	10.47667	7.814286	13.13906	9.940906	6.525738	13.35607
2033	4.602862	2.073698	7.132025	5.286513	3.840704	6.732322	4.819561	3.601599	6.037524	2.39933	1.690651	3.10801	4.389752	3.031132	5.748372	19.95075	8.353782	31.54772	10.46133	7.500439	13.42222	9.905037	6.071075	13.739
2034	4.527953	1.749163	7.306742	5.211093	3.651509	6.770678	4.778032	3.435994	6.12007	2.386197	1.604932	3.167463	4.400314	2.886741	5.913888	19.91729	7.003309	32.83127	10.44744	7.174818	13.72007	9.865093	5.601303	14.12888
2035	4.452308	1.424	7.480617	5.139814	3.462015	6.817612	4.737316	3.268687	6.205946	2.373212	1.517596	3.228828	4.409756	2.735627	6.083884	19.87546	5.607812	34.14311	10.43534	6.83831	14.03237	9.82144	5.118057	14.52482

(6) ASDRs, female

Years	Central Europe, Eastern Europe, and Central Asia			High-income			Latin America and Caribbean			North Africa and Middle East			South Asia			Southeast Asia, East Asia, and Oceania			Sub-Saharan Africa			Global		
	pred	lower UI	upper UI	pred	lower UI	upper UI	pred	lower UI	upper UI	pred	lower UI	upper UI	pred	lower UI	upper UI	pred	lower UI	upper UI	pred	lower UI	upper UI	pred	lower UI	upper UI
2022	1.242942	1.18933	1.296555	1.407332	1.370454	1.44421	1.421369	1.370248	1.472491	1.61853	1.552993	1.684067	2.861745	2.766213	2.957277	4.60532	4.342467	4.868172	6.571871	6.408307	6.735435	2.93989	2.858253	3.021526
2023	1.247561	1.164932	1.330191	1.386716	1.331357	1.442074	1.403659	1.332774	1.474544	1.591209	1.502248	1.680171	2.814097	2.669944	2.95825	4.556494	4.155641	4.957347	6.514915	6.280791	6.749039	2.91817	2.784328	3.052011
2024	1.252688	1.134955	1.370422	1.366591	1.289195	1.443986	1.386202	1.292015	1.480388	1.564252	1.448032	1.680471	2.766536	2.563451	2.969621	4.50519	3.930548	5.079832	6.456353	6.137415	6.775291	2.896731	2.698234	3.095229
2025	1.257965	1.100158	1.415772	1.346778	1.244846	1.44871	1.368935	1.248878	1.488992	1.537569	1.391365	1.683772	2.720636	2.451727	2.989544	4.450491	3.6778	5.223183	6.396097	5.981882	6.810312	2.874536	2.602488	3.146584
2026	1.263037	1.060669	1.465406	1.327344	1.198901	1.455786	1.351886	1.203939	1.499833	1.511311	1.333	1.689621	2.675763	2.335885	3.015642	4.393862	3.404746	5.382978	6.333912	5.816052	6.851772	2.852585	2.499559	3.20561
2027	1.268107	1.016934	1.51928	1.308168	1.151585	1.464751	1.335069	1.157571	1.512567	1.485511	1.2734	1.697623	2.631637	2.216476	3.046798	4.336213	3.115911	5.556514	6.270251	5.641761	6.898741	2.831186	2.390415	3.271958
2028	1.27346	0.96961	1.577311	1.289325	1.103322	1.475328	1.318539	1.110139	1.526939	1.46021	1.21309	1.707329	2.587665	2.094001	3.081328	4.278369	2.815086	5.741651	6.205552	5.460839	6.950264	2.810914	2.276714	3.345115
2029	1.279074	0.918922	1.639227	1.270817	1.054355	1.487279	1.302308	1.061867	1.542748	1.435416	1.152369	1.718463	2.543803	1.969216	3.11839	4.219584	2.504084	5.935084	6.139946	5.274387	7.005505	2.791041	2.158592	3.42349
2030	1.284759	0.864834	1.704684	1.252554	1.004773	1.500336	1.286319	1.012861	1.559776	1.411081	1.091358	1.730804	2.50089	1.843398	3.158382	4.159315	2.184509	6.13412	6.073287	5.082966	7.063609	2.771075	2.036192	3.505957
2031	1.290202	0.807165	1.773239	1.234644	0.954771	1.514516	1.270567	0.963232	1.577902	1.387335	1.030252	1.744417	2.45836	1.716472	3.200248	4.099073	1.858713	6.339433	6.005426	4.886962	7.123891	2.752072	1.910517	3.593628
2032	1.295443	0.74599	1.844895	1.216958	0.90434	1.529575	1.255037	0.913074	1.597	1.364188	0.969153	1.759224	2.416253	1.588676	3.24383	4.039565	1.528117	6.551013	5.936826	4.687241	7.186411	2.734315	1.781685	3.686945
2033	1.300576	0.68158	1.919571	1.199515	0.853657	1.545374	1.239777	0.862554	1.617	1.341642	0.908271	1.775012	2.374273	1.460327	3.288218	3.981561	1.19421	6.768912	5.867963	4.484896	7.251031	2.717792	1.650139	3.785444
2034	1.305573	0.614063	1.997082	1.182314	0.802841	1.561787	1.224801	0.811779	1.637823	1.31968	0.847714	1.791646	2.332216	1.33174	3.332691	3.924518	0.857677	6.99136	5.798941	4.280524	7.317359	2.70176	1.515735	3.887786
2035	1.310356	0.543482	2.077229	1.165316	0.75195	1.578681	1.210043	0.760773	1.659313	1.298261	0.787504	1.809018	2.290465	1.203504	3.377427	3.868057	0.519181	7.216933	5.729567	4.074354	7.38478	2.686035	1.378602	3.993468

(7) ASIRs, both

Years	Central Europe, Eastern Europe, and Central Asia			High-income			Latin America and Caribbean			North Africa and Middle East			South Asia			Southeast Asia, East Asia, and Oceania			Sub-Saharan Africa			Global		
	pred	lower UI	upper UI	pred	lower UI	upper UI	pred	lower UI	upper UI	pred	lower UI	upper UI	pred	lower UI	upper UI	pred	lower UI	upper UI	pred	lower UI	upper UI	pred	lower UI	upper UI
2022	3.07162	2.766553	3.376832	4.404901	4.196847	4.612954	3.120729	2.642817	3.59864	1.965799	1.732632	2.198966	3.351813	3.08102	3.622606	12.2537	10.97787	13.52954	7.850406	7.339125	8.361687	6.662265	6.278176	7.046353
2023	3.04403	2.682878	3.405345	4.341728	4.105587	4.577869	3.094617	2.613363	3.57587	1.948427	1.707764	2.189091	3.341353	3.046389	3.636318	12.29654	10.83675	13.75634	7.817623	7.263974	8.371271	6.662777	6.214317	7.111238
2024	3.01756	2.58412	3.451203	4.28173	4.002737	4.560722	3.069177	2.578054	3.5603	1.931645	1.677599	2.185691	3.331096	2.997855	3.664337	12.33846	10.58855	14.08837	7.783936	7.162108	8.405764	6.661393	6.112846	7.209939
2025	2.991439	2.47008	3.513063	4.224133	3.890123	4.558142	3.0439	2.536271	3.551529	1.915254	1.641685	2.188823	3.321872	2.936844	3.706899	12.37988	10.24289	14.51688	7.74973	7.035182	8.464277	6.658096	5.978769	7.337423
2026	2.965652	2.342104	3.589533	4.167801	3.768885	4.566717	3.018787	2.487784	3.549789	1.899435	1.60038	2.198491	3.313422	2.864675	3.76217	12.42423	9.815232	15.03322	7.714927	6.885999	8.543856	6.655108	5.81871	7.491507
2027	2.942825	2.203545	3.682513	4.11066	3.638833	4.582487	2.994143	2.432616	3.55567	1.884305	1.554299	2.214311	3.305729	2.782849	3.828609	12.47357	9.317372	15.62976	7.680162	6.718174	8.64215	6.652631	5.635949	7.669313
2028	2.924619	2.057412	3.792321	4.055525	3.504776	4.606274	2.970454	2.371514	3.569394	1.870061	1.504491	2.235631	3.298514	2.692968	3.904059	12.52945	8.759769	16.29914	7.646163	6.535886	8.75644	6.650826	5.435653	7.865999
2029	2.909777	1.903388	3.916753	4.004345	3.369634	4.639056	2.947809	2.305039	3.590579	1.856751	1.451609	2.261893	3.291711	2.59612	3.987302	12.59308	8.147084	17.03907	7.613229	6.341608	8.88485	6.648803	5.219525	8.078081
2030	2.897279	1.740816	4.054439	3.956568	3.233424	4.679713	2.926017	2.233465	3.618569	1.844217	1.395861	2.292574	3.285844	2.493298	4.078391	12.6659	7.480037	17.85177	7.581623	6.136652	9.026595	6.646414	4.98851	8.304317
2031	2.887208	1.569312	4.205936	3.911642	3.095738	4.727546	2.905031	2.15713	3.652933	1.832643	1.337603	2.327682	3.280747	2.384698	4.176796	12.75365	6.758518	18.74877	7.551365	5.921726	9.181003	6.64713	4.744949	8.549312
2032	2.881231	1.389027	4.374576	3.867942	2.955245	4.780639	2.885051	2.076491	3.693611	1.822122	1.277079	2.367164	3.276547	2.270565	4.28253	12.86041	5.978797	19.74202	7.523085	5.697853	9.348317	6.652475	4.488886	8.816065
2033	2.880265	1.199738	4.562468	3.827226	2.813972	4.840481	2.866605	1.992336	3.74088	1.812796	1.214713	2.410883	3.272985	2.15112	4.39485	12.98914	5.136761	20.84151	7.497589	5.466548	9.528629	6.663006	4.221911	9.1041
2034	2.883306	0.99942	4.769478	3.79074	2.673034	4.908447	2.849867	1.905088	3.79468	1.804674	1.150714	2.458747	3.26995	2.026454	4.513446	13.13985	4.223998	22.05651	7.475199	5.228382	9.722015	6.67667	3.94264	9.4107
2035	2.88976	0.786159	4.996492	3.758132	2.532004	4.984288	2.83471	1.814792	3.85469	1.797608	1.084845	2.510624	3.267862	1.896754	4.63897	13.31254	3.227866	23.39964	7.456091	4.983327	9.928856	6.691857	3.649345	9.734369

(8) ASIRs, male

Years	Central Europe, Eastern Europe, and Central Asia			High-income			Latin America and Caribbean			North Africa and Middle East			South Asia			Southeast Asia, East Asia, and Oceania			Sub-Saharan Africa			Global		
	pred	lower UI	upper UI	pred	lower UI	upper UI	pred	lower UI	upper UI	pred	lower UI	upper UI	pred	lower UI	upper UI	pred	lower UI	upper UI	pred	lower UI	upper UI	pred	lower UI	upper UI
2022	5.594057	5.37676	5.811355	7.439352	7.294993	7.583711	5.181241	4.743502	5.618981	2.398408	2.314135	2.482682	3.988968	3.85192	4.126016	20.44897	19.41515	21.48279	9.891019	9.647434	10.1346	10.69702	10.3779	11.01613
2023	5.505945	5.136611	5.87528	7.325423	7.098257	7.552588	5.14316	4.60803	5.67829	2.387779	2.270522	2.505035	4.00916	3.817726	4.200595	20.58908	19.05657	22.12158	9.885304	9.513053	10.25755	10.71598	10.22842	11.20355
2024	5.419689	4.870373	5.969005	7.216311	6.889667	7.542956	5.105303	4.491019	5.719587	2.37721	2.220513	2.533907	4.028863	3.76793	4.289796	20.71568	18.53516	22.8962	9.877776	9.348108	10.40744	10.72778	10.02587	11.42969
2025	5.33132	4.582772	6.079869	7.110826	6.673136	7.548515	5.066683	4.386242	5.747124	2.366443	2.165311	2.567574	4.048237	3.705941	4.390534	20.83141	17.88784	23.77498	9.869078	9.159719	10.57844	10.73403	9.783493	11.68456
2026	5.23981	4.277087	6.202534	7.006348	6.448105	7.564591	5.027284	4.284462	5.770106	2.355735	2.10592	2.60555	4.067245	3.633458	4.501032	20.9403	17.13451	24.74609	9.859002	8.950861	10.76714	10.73764	9.50837	11.96691
2027	5.151971	3.961061	6.34288	6.898698	6.211816	7.585581	4.987723	4.17958	5.795866	2.345281	2.043016	2.647545	4.085891	3.551506	4.620276	21.0439	16.28557	25.80224	9.848106	8.723972	10.97224	10.73833	9.201704	12.27496

2028	5.070748	3.640093	6.501404	6.793492	5.972024	7.61496	4.948721	4.069254	5.828189	2.335185	1.977302	2.693068	4.103877	3.460776	4.746978	21.1379	15.3494	26.92641	9.837097	8.481852	11.19234	10.73427	8.868057	12.60048
2029	4.991438	3.312717	6.670159	6.694528	5.73346	7.655595	4.91021	3.952475	5.867944	2.325347	1.909033	2.741661	4.120922	3.361669	4.880175	21.22025	14.33191	28.10859	9.825972	8.225889	11.42605	10.72354	8.509176	12.93791
2030	4.911194	2.978726	6.843661	6.600792	5.495725	7.705859	4.871795	3.82884	5.914751	2.315529	1.83819	2.792868	4.137186	3.254725	5.019647	21.29352	13.2394	29.34765	9.815198	7.957312	11.67308	10.70711	8.127732	13.28648
2031	4.829892	2.639218	7.020566	6.510676	5.257398	7.763954	4.833356	3.698517	5.968196	2.305985	1.765058	2.846912	4.152667	3.140181	5.165152	21.36491	12.07832	30.6515	9.804695	7.676482	11.93291	10.69032	7.727939	13.6527
2032	4.751594	2.296862	7.206326	6.420701	5.01557	7.825832	4.79529	3.562185	6.028394	2.296875	1.689837	2.903913	4.167313	3.018206	5.316421	21.43814	10.85127	32.02502	9.795027	7.384157	12.2059	10.6749	7.30999	14.03981
2033	4.677724	1.953575	7.401872	6.334528	4.774314	7.894741	4.758466	3.42114	6.095792	2.288239	1.612822	2.963655	4.180677	2.888915	5.472438	21.51159	9.562081	33.46109	9.787056	7.081864	12.49225	10.66035	6.876637	14.44407
2034	4.604734	1.608733	7.600734	6.25471	4.536061	7.973359	4.723029	3.276099	6.169958	2.279958	1.534074	3.025841	4.19257	2.752554	5.632586	21.58067	8.211838	34.9495	9.780907	6.77021	12.7916	10.64271	6.427117	14.8583
2035	4.530728	1.262707	7.798749	6.18056	4.30026	8.060859	4.68868	3.127366	6.249994	2.271839	1.453517	3.090161	4.203242	2.609565	5.79692	21.64304	6.801809	36.48426	9.776867	6.449599	13.10413	10.61958	5.961185	15.27798

(9) ASIRs, female

Years	Central Europe, Eastern Europe, and Central Asia			High-income			Latin America and Caribbean			North Africa and Middle East			South Asia			Southeast Asia, East Asia, and Oceania			Sub-Saharan Africa			Global		
	pred	lower UI	upper UI	pred	lower UI	upper UI	pred	lower UI	upper UI	pred	lower UI	upper UI	pred	lower UI	upper UI	pred	lower UI	upper UI	pred	lower UI	upper UI	pred	lower UI	upper UI
2022	1.226884	1.120354	1.333414	1.787415	1.741999	1.83283	1.34679	1.297966	1.395613	1.55227	1.489126	1.615413	2.735769	2.646055	2.825483	5.018837	4.73226	5.305414	6.040298	5.887558	6.193039	3.145774	3.055515	3.236033
2023	1.233657	1.093661	1.373654	1.763182	1.694088	1.832275	1.332495	1.265101	1.399889	1.529016	1.442839	1.615192	2.69481	2.560172	2.829448	4.992152	4.564294	5.420011	5.989808	5.771314	6.208301	3.131808	2.990364	3.273252
2024	1.240492	1.067853	1.413131	1.739655	1.642197	1.837113	1.318412	1.229093	1.407731	1.506129	1.393022	1.619236	2.653794	2.464583	2.843005	4.963683	4.355372	5.571993	5.937984	5.640302	6.235666	3.117997	2.912314	3.32368
2025	1.247161	1.04027	1.454053	1.716612	1.587461	1.845763	1.304485	1.19077	1.4182	1.483544	1.340685	1.626403	2.614125	2.36382	2.86443	4.932223	4.115643	5.748803	5.884746	5.497993	6.2715	3.102966	2.82361	3.382321
2026	1.25345	1.008668	1.498233	1.694179	1.530664	1.857694	1.290728	1.150644	1.430811	1.46139	1.286551	1.636229	2.575242	2.258916	2.891569	4.899572	3.852568	5.946575	5.829874	5.34614	6.313608	3.088052	2.727063	3.449041
2027	1.25957	0.972294	1.546845	1.672152	1.472027	1.872277	1.277138	1.109042	1.445235	1.4397	1.231063	1.648337	2.536995	2.150432	2.923557	4.8669	3.57061	6.16319	5.773784	5.186455	6.361114	3.073839	2.623891	3.523788
2028	1.265773	0.9313	1.600246	1.650629	1.412082	1.889177	1.263773	1.066297	1.461248	1.418503	1.174694	1.662312	2.498848	2.038831	2.958864	4.835183	3.273391	6.396974	5.716861	5.020609	6.413113	3.06094	2.515819	3.606062
2029	1.272058	0.885895	1.658221	1.629617	1.351131	1.908102	1.250647	1.022616	1.478677	1.397787	1.117699	1.677875	2.460624	1.924685	2.996564	4.803432	2.962116	6.644748	5.659214	4.849603	6.468825	3.048322	2.40268	3.693964
2030	1.278292	0.836216	1.720368	1.609006	1.289286	1.928727	1.237708	0.97809	1.497326	1.377497	1.060187	1.694807	2.423037	1.809133	3.036941	4.770914	2.637797	6.904032	5.600712	4.673949	6.527475	3.035168	2.284307	3.78603
2031	1.284237	0.78229	1.786184	1.588982	1.226818	1.951145	1.224946	0.932812	1.517079	1.35774	1.002334	1.713147	2.385595	1.692124	3.079066	4.739488	2.302546	7.17643	5.541205	4.493982	6.588427	3.022903	2.161947	3.883859
2032	1.289926	0.724311	1.85554	1.569357	1.163677	1.975037	1.212334	0.886857	1.53781	1.338524	0.94423	1.732817	2.348414	1.573925	3.122903	4.710129	1.957429	7.462828	5.481103	4.310476	6.65173	3.012064	2.035867	3.988262
2033	1.295408	0.66261	1.928206	1.55015	1.100066	2.000234	1.199928	0.840387	1.559468	1.319843	0.886061	1.753626	2.311232	1.454849	3.167615	4.683619	1.603514	7.763723	5.420828	4.124415	6.717241	3.002598	1.90649	4.098707
2034	1.300649	0.597396	2.003901	1.531351	1.036113	2.02659	1.187748	0.793502	1.581993	1.301661	0.827906	1.775417	2.273782	1.335143	3.21242	4.659138	1.240869	8.077406	5.360472	3.936336	6.784609	2.993496	1.773462	4.213529
2035	1.305587	0.528799	2.082375	1.512924	0.971885	2.053964	1.175737	0.746225	1.605249	1.283919	0.769778	1.79806	2.236413	1.215365	3.257462	4.636048	0.869576	8.40252	5.299877	3.746455	6.8533	2.984296	1.636726	4.331867

Supplementary Table S15 | The BAPC prediction results (predicted values and 95% uncertainty intervals) for absolute numbers of incidence, deaths and DALYs of esophageal cancer in world and seven GBD super regions from 2022 to 2035.

(1) DALYs, both sexes

Years	Central Europe, Eastern Europe, and Central Asia			High-income			Latin America and Caribbean			North Africa and Middle East			South Asia			Southeast Asia, East Asia, and Oceania			Sub-Saharan Africa			Global		
	pred	lower UI	upper UI	pred	lower UI	upper UI	pred	lower UI	upper UI	pred	lower UI	upper UI	pred	lower UI	upper UI	pred	lower UI	upper UI	pred	lower UI	upper UI	pred	lower UI	upper UI
2022	517224.9	477934.6	556515.2	1642147	1535888	1748406	537091.9	500199.2	573984.5	246059.4	234353.9	257764.9	1471997	1315318	1628677	7827486	6759019	8895952	1156684	1091688	1221680	13321125	12390773	14251477
2023	518403.8	469150.3	567657.3	1630154	1512133	1748176	549146.2	506802.7	591489.7	253016.5	239339.1	266693.8	1508938	1337428	1680448	8008374	6814170	9202578	1193094	1118876	1267312	13585600	12509323	14661878
2024	519441.4	455000	583882.9	1618059	1481036	1755083	561428	510420.4	612435.6	260332.5	243626.9	277038.1	1547087	1353573	1740600	8193002	6805063	9580941	1231415	1143003	1319826	13849357	12544494	15154219
2025	520502.3	436659.1	604345.4	1605447	1442957	1767937	573708.7	510935.9	636481.5	267713.4	246955.6	288471.2	1586388	1363164	1809611	8380650	6727793	10033506	1271409	1163643	1379175	14110823	12497637	15724009
2026	521970.7	415152.5	628789	1592523	1399176	1785870	586108.1	508575.3	663640.9	275247	249441.8	301052.3	1626785	1366081	1887489	8570202	6582857	10557548	1312899	1180712	1445085	14377233	12381004	16373462
2027	524258.8	391127.9	657389.6	1579095	1350524	1807667	598670.6	503493.4	693847.9	282876.4	251055.6	314697.2	1668242	1362401	1974083	8754996	6368345	11141648	1355849	1194311	1517386	14644908	12196388	17093429
2028	527162.4	364608.1	689716.7	1565846	1298505	1833187	611636.9	496017.2	727256.7	290799.4	252059.8	329539.1	1710832	1352379	2069286	8935991	6089399	11782583	1400925	1205166	1596684	14913557	11951161	17875953
2029	530404.8	335409.2	725400.5	1552933	1243849	1862016	625080.4	486252.2	763908.5	299057.9	252513.3	345602.6	1754683	1336195	2173170	9120965	5752930	12489000	1448533	1213596	1683470	15182225	11647930	18716520
2030	534052.8	303450.3	764655.3	1539958	1186617	1893298	638783.3	473998.4	803568.1	307367.9	252160.8	362575	1799723	1313752	2285694	9312382	5359017	13265746	1498461	1219258	1777664	15448763	11285816	19611709
2031	538393.7	268621.5	808165.9	1527220	1127274	1927167	652877	459241.9	846512.1	315850.7	251061.7	380639.7	1846009	1284920	2407098	9513632	4905873	14121391	1550683	1221875	1879491	15725732	10872135	20579329
2032	543811.3	230666.8	856955.9	1514606	1065879	1963332	667390.8	441827.5	892954.1	324427.9	249096.5	399759.4	1893490	1249394	2537586	9722488	4387222	15057755	1605300	1221217	1989384	16016158	10405193	21627124
2033	550276.9	189061.7	911492.1	1502630	1002997	2002263	682579.7	421741.1	943418.3	333232.6	246382.8	420082.4	1942121	1206877	2677364	9942959	3799819	16086100	1663088	1217583	2108593	16322471	9885917	22759024
2034	557648.2	143124.6	972224.1	1491335	938725.2	2043945	698590.8	398791.6	998390	342302.7	242924.6	441680.8	1992089	1157105	2827072	10181225	3137581	17224938	1724510	1210885	2238136	16639533	9308011	23971056
2035	565902.1	102638.9	1040003	1480322	872803.1	2087842	715241.9	372523.8	1057960	351390.3	238498.1	464282.4	2043366	1099554	2987178	10437136	2389368	18486096	1789417	1200493	2378342	16959947	8662895	25256999

(2) DALYs, male

Years	Central Europe, Eastern Europe, and Central Asia			High-income			Latin America and Caribbean			North Africa and Middle East			South Asia			Southeast Asia, East Asia, and Oceania			Sub-Saharan Africa			Global		
	pred	lower UI	upper UI	pred	lower UI	upper UI	pred	lower UI	upper UI	pred	lower UI	upper UI	pred	lower UI	upper UI	pred	lower UI	upper UI	pred	lower UI	upper UI	pred	lower UI	upper UI
2022	402951.4	370966.6	434936.1	1298491	1210206	1386776	420517	389933.4	451100.6	148947.9	142644.1	155251.7	870200.8	767734.4	972667.3	6432416	5534386	7330446	714446.5	674792.2	754100.9	10177260	9441842	10912678
2023	402028	363483.3	440572.7	1288021	1190855	1385187	429116.4	395600.9	462631.9	153677.4	146122.6	161232.3	898067.4	787191.3	1008944	6590582	5593659	7587506	737674.8	692658.7	782690.9	10384884	9537992	11231777
2024	400911.1	352182.4	449639.9	1277355	1165744	1388966	437794.8	399442.5	476147.2	158676.5	149192.7	168160.4	926905.8	804152.4	1049659	6751013	5603278	7898748	762198.3	708999.8	815396.8	10589761	9568936	11610587
2025	399704.7	337868.6	461540.8	1266237	1135123	1397351	446367.3	401183.1	491551.5	163758	151699.5	175816.6	956629.6	818004.2	1095255	6913849	5559394	8268305	787903	723540	852265.9	10791967	9536188	12047747
2026	398764	321424.2	476103.7	1254849	1099976	1409721	454945.7	400946.7	508944.8	168986.2	153728.6	184243.9	987240	828426.2	1146054	7078478	5461497	8695459	814696.6	736192	893201.3	10998034	9449450	12546619
2027	398401.6	303423.7	493379.5	1243101	1061019	1425183	463560.8	398858	528263.6	174300.8	155233.3	193368.3	1018689	835226.7	1202152	7239085	5307147	9171023	842569.4	746989	938149.7	11204151	9309034	13099269
2028	398448.1	283950.5	512945.8	1231515	1019436	1443593	472382.8	395205.8	549559.7	179812	156359.4	203264.6	1051059	838438	1263679	7395301	5099762	9690839	871960.8	756391.1	987530.6	11409124	9120291	13697956
2029	398651.9	262921.9	534381.9	1220197	975807.2	1464587	481418.9	390105.7	572732.2	185566.5	157155.6	213977.5	1084437	838089.4	1330785	7553347	4845242	10261452	903113	764577.4	1041648	11611899	8885281	14338517
2030	398990.2	240343.7	557636.8	1208872	930225.8	1487518	490489.9	383471	597508.8	191395.8	157463.4	225328.2	1118722	834013	1403431	7715848	4544397	10887300	935955.9	771373.3	1100539	11811301	8603889	15018712
2031	399707.6	216291.8	583123.3	1197769	883064	1512474	499716.3	375388.9	624043.7	197398	157331.5	237464.5	1153962	826109.8	1481814	7886041	4196404	11575678	970523.2	776629.5	1164417	12018403	8282762	15754044
2032	401086.5	190749.5	611423.4	1186851	834417.1	1539285	509106.5	365828.7	652384.4	203493.8	156651.5	250336.1	1190072	814144.2	1565999	8062068	3796541	12327594	1006911	780210.4	1233611	12235529	7920515	16550543
2033	403134	163524	642743.9	1176523	784729.4	1568317	518827	354893.5	682760.5	209733.2	155465.1	264001.4	1227035	797933.6	1656137	8246423	3342734	13150112	1045614	782307.4	1308921	12464636	7518284	17410987
2034	405679.8	134278	677081.6	1166810	734069.1	1599551	528925.2	342562.9	715287.4	216160.4	153786.3	278534.5	1264995	777335.6	1752655	8443755	2831099	14056482	1086939	782870.9	1391006	12700757	7070779	18330734
2035	408548.6	102638.9	714458.2	1157424	682244.7	1632602	539239	328651.7	749826.4	222640	151484.6	293795.5	1303889	752008.8	1855768	8653954	2253877	15055059	1130906	781567.3	1480245	12937392	6571114	19303670

(3) DALYs, female

Years	Central Europe, Eastern Europe, and Central Asia			High-income			Latin America and Caribbean			North Africa and Middle East			South Asia			Southeast Asia, East Asia, and Oceania			Sub-Saharan Africa			Global		
	pred	lower UI	upper UI	pred	lower UI	upper UI	pred	lower UI	upper UI	pred	lower UI	upper UI	pred	lower UI	upper UI	pred	lower UI	upper UI	pred	lower UI	upper UI	pred	lower UI	upper UI
2022	114273.5	106968	121579.1	343656.2	325682.6	361629.7	116574.9	110265.8	122883.9	97111.51	91709.85	102513.2	601796.5	547583.5	656009.5	1395070	1224633	1565506	442237.4	416896	467578.7	3143865	2948932	3338799
2023	116375.8	105667	127084.6	342133.1	321277.7	362988.6	120029.8	111201.8	128857.8	99339.05	93216.52	105461.6	610870.5	550236.4	671504.6	1417791	1220511	1615072	455418.8	426217	484620.6	3200716	2971331	3430100
2024	118530.3	102817.5	134243	340704.4	315291.8	366116.9	123633.1	110977.8	136288.4	101656	94434.25	108877.7	620180.9	549421	690940.8	1441989	1201785	1682193	469216.2	434003.3	504429.1	3259595	2975558	3543633

2025	120797.6	98790.54	142804.6	339209.9	307833.8	370586	127341.4	109752.8	144930	103955.3	95256.08	112654.6	629758	545159.6	714356.3	1466801	1168400	1765202	483506	440102.7	526909.3	3318856	2961449	3676262
2026	123206.8	93728.25	152685.3	337674.4	299199.8	376149.1	131162.4	107628.6	154696.1	106260.8	95713.19	116808.4	639545.1	537654.8	741435.5	1491724	1121360	1862089	498202.1	444520.4	551883.9	3379199	2931554	3826843
2027	125857.2	87704.22	164010.1	335994.8	289505.2	382484.4	135109.9	104635.4	165584.3	108575.6	95822.3	121328.9	649552.9	527174.7	771931	1515911	1061198	1970625	513279.4	447322.2	579236.6	3440757	2887354	3994160
2028	128714.2	80657.57	176770.9	334331.5	279069	389593.9	139254.2	100811.4	177697	110987.4	95700.4	126274.5	659773.8	513940.7	805606.8	1540690	989636.6	2091744	528964.1	448775.1	609153	3504433	2830869	4177997
2029	131752.9	72487.34	191018.5	332735.2	268041.8	397428.7	143661.5	96146.54	191176.4	113491.4	95357.74	131625.1	670245.1	498106.1	842384.1	1567618	907688.5	2227548	545420	449018.7	641821.3	3570326	2762649	4378003
2030	135062.6	63106.6	207018.5	331085.6	256391.4	405779.8	148293.4	90527.41	206059.3	115972.2	94697.47	137246.9	681001.1	479739	882263.3	1596533	814620.7	2378446	562505.3	447884.7	677125.9	3637462	2681927	4592997
2031	138686.1	52329.69	225042.6	329451.4	244210.1	414692.6	153160.7	83853.02	222468.4	118452.7	93730.18	143175.2	692046.9	458809.9	925284	1627591	709469.1	2545713	580159.5	445245	715073.9	3707329	2589372	4825285
2032	142724.9	39917.22	245532.5	327754.6	231461.5	424047.8	158284.3	75998.85	240569.8	120934.1	92445	149423.3	703418.4	435249.5	971587.3	1660421	590680.8	2730161	598389.8	441007	755772.5	3780630	2484678	5076581
2033	147142.9	25537.67	268748.1	326106.9	218267.8	433946.1	163752.7	66847.58	260657.8	123499.4	90917.69	156081	715085.2	408943.7	1021227	1696536	457084.9	2935987	617473.6	435275.2	799672	3857835	2367633	5348037
2034	151968.4	8846.54	295142.6	324525.1	204656.1	444394.2	169665.6	56228.72	283102.6	126142.3	89138.28	163146.4	727093.5	379769.7	1074417	1737469	306481.5	3168457	637571.7	428013.9	847129.4	3938777	2237232	5640321
2035	157353.6	0	325544.8	322898.9	190558.4	455239.4	176002.8	43872.08	308133.5	128750.2	87013.56	170486.9	739477.4	347545.1	1131410	1783182	135490.9	3431038	658511.3	418925.6	898097.1	4022555	2091780	5953329

(4) Incidence, both

Years	Central Europe, Eastern Europe, and Central Asia			High-income			Latin America and Caribbean			North Africa and Middle East			South Asia			Southeast Asia, East Asia, and Oceania			Sub-Saharan Africa			Global		
	pred	lower UI	upper UI	pred	lower UI	upper UI	pred	lower UI	upper UI	pred	lower UI	upper UI	pred	lower UI	upper UI	pred	lower UI	upper UI	pred	lower UI	upper UI	pred	lower UI	upper UI
2022	20016.42	18216.01	21817.22	97931.93	86168.14	109695.7	20146.99	18509.07	21784.92	9475.033	8344.574	10605.49	51230.12	47228.13	55232.1	364934.8	325744	404125.6	39998.86	37476.24	42521.48	598139.2	563710.6	632567.8
2023	20047.58	17911.55	22184.06	98207.19	86173.3	110241.1	20603.27	18819.55	22386.99	9791.747	8577.457	11006.04	52531.22	48026.7	57035.74	377559.6	331669.2	423450.1	41372.76	38521.53	44224	614255.2	572977.1	655533.3
2024	20085.77	17454.98	22717.12	98544.74	85954.68	111134.8	21072.96	19072.14	23073.78	10129.87	8794.786	11464.96	53887.79	48620.3	59155.28	390778.9	334445	447112.9	42828.57	39478.1	46179.03	630834.1	578965.9	682702.2
2025	20126.03	16868.21	23384.6	98896.61	85475.26	112318	21548.38	19255.67	23841.09	10483.13	8985.751	11980.51	55298.37	49005.23	61591.52	404568.7	333963.5	475173.9	44354.93	40328.32	48381.54	647801.1	581801.8	713800.5
2026	20167.42	16167.85	24167.96	99198.6	84696.55	113700.6	22024.04	19365.12	24682.95	10847.39	9143.239	12551.53	56749.14	49176.92	64321.37	418756.7	330159.7	507353.7	45937.83	41058.94	50816.72	665091	581621.2	748560.7
2027	20225.29	15375.96	25075.83	99359.4	83543.73	115175.1	22496.85	19399.98	25593.72	11218.63	9262.088	13175.16	58232.79	49136.75	67328.84	432903	322774.3	543031.8	47567.2	41662.02	53472.37	682304.4	578175.2	786433.5
2028	20309.11	14511.24	26108.49	99479.72	82105.87	116853.6	22977.37	19373.69	26581.04	11607.02	9351.208	13862.82	59761.9	48906.93	70616.88	447117.6	312037.4	582197.9	49273.19	42166.97	56379.4	699569.9	571924.8	827215
2029	20410.73	13571.68	27251.64	99644.51	80463.82	118825.2	23472.38	19294.14	27650.61	12020.15	9415.949	14624.35	61349.57	48502.69	74196.44	461980.1	298318	625642.3	51082.02	42593.31	59570.73	717239.5	563263.7	871215.2
2030	20524.81	12553.03	28498.87	99822.11	78607.58	121036.6	23974.68	19155.55	28793.81	12450.96	9448.536	15453.39	62993.56	47920.5	78066.62	477684.9	281512.6	673857.1	52984.93	42925.54	63044.31	735340.2	552162	918518.4
2031	20652.31	11450.7	29856.72	99968.09	76514.42	123421.8	24478.83	18952.83	30004.84	12894.6	9442.758	16346.45	64689.71	47149.61	82229.82	494283.1	261286.8	727279.4	54971.93	43144.35	66799.51	754049.9	538560.8	969539
2032	20804.61	10262.65	31350.43	99996.17	74132.46	125859.9	24982.01	18683.05	31280.97	13346.07	9392.683	17299.46	66434.17	46176.01	86692.32	511475.8	237056.1	785895.6	57036.44	43232.31	70840.57	773138.9	522039	1024239
2033	20990.95	8986.649	33000.85	99990.68	71543	128438.4	25494.64	18354.58	32634.75	13816.16	9304.89	18327.46	68232.54	44996.32	91468.76	529550.2	208580.2	850520.2	59211.7	43204.02	75219.37	792806.4	502761.6	1082851
2034	21205.84	7608	34811.41	100026.9	68810.91	131242.8	26023.74	17970.95	34076.75	14313.26	9182.791	19444.62	70095.97	43606.01	96585.92	549098	175512.8	922697.6	61525.39	43063.52	79987.26	813286	480739.5	1145833
2035	21443.43	6109.427	36788.06	100081.6	65926.88	134236.6	26563.01	17524.53	35601.9	14829.73	9016.24	20645.25	72020.93	41986.65	102055.2	570200	137032	1003411	63969.93	42784.55	85155.32	834407.4	455610.1	1213205

(5) Incidence, male

Years	Central Europe, Eastern Europe, and Central Asia			High-income			Latin America and Caribbean			North Africa and Middle East			South Asia			Southeast Asia, East Asia, and Oceania			Sub-Saharan Africa			Global		
	pred	lower UI	upper UI	pred	lower UI	upper UI	pred	lower UI	upper UI	pred	lower UI	upper UI	pred	lower UI	upper UI	pred	lower UI	upper UI	pred	lower UI	upper UI	pred	lower UI	upper UI
2022	15255.82	14107.74	16403.9	75519.85	65125.69	85914	15359.17	14281.31	16437.03	5689.524	5067.192	6311.856	30038.38	27647.72	32429.04	286488.1	257266.8	315709.5	24126.75	22709.47	25544.02	447143.3	421174.9	473111.7
2023	15206.79	13777.51	16636.08	75744.49	65247	86241.97	15705.38	14519.08	16891.67	5900.675	5227.976	6573.373	31046.74	28370.22	33723.27	296722.1	262313.9	331130.2	25003.17	23369.35	26636.99	459752.5	428691.7	490813.3
2024	15159.44	13318.41	17000.54	76014.17	65218.16	86810.19	16060.21	14711.63	17408.8	6127.336	5381.755	6872.916	32099.05	28995.43	35202.67	307394.6	264944.2	349845	25933.21	23968	27898.41	472631.4	433717	511545.8
2025	15109.52	12755.9	17463.35	76293.97	65007.42	87580.52	16418	14851.43	17984.57	6365.136	5521.391	7208.881	33189.87	29508.68	36871.06	318544.8	265134.4	371955.2	26911.89	24498.02	29325.77	485841.8	436426.8	535256.8
2026	15056.51	12107.99	18005.39	76525.81	64571.92	88479.69	16774.68	14935.56	18613.8	6611.215	5642.164	7580.266	34313.72	29903.21	38724.22	330036.8	262822.5	397251.2	27932.22	24953.9	30910.53	499323.4	436885.2	561761.7
2027	15015.7	11396.41	18635.52	76626.84	63835.19	89418.49	17128.44	14964.63	19292.24	6862.94	5740.641	7985.239	35466.29	30176.64	40755.94	341485.7	257760.4	425211	28989.27	25332.04	32646.49	512709.6	434828	590591.2
2028	14995.06	10637.28	19353.57	76684.11	62864.76	90503.45	17487.06	14949.3	20024.82	7127.162	5822.716	8431.607	36657.14	30340.49	42973.8	352900.6	250083.1	455718.2	30101.3	25649.83	34552.78	525993.3	430537.9	621448.7
2029	14985.94	9827.748	20145.08	76774.19	61728.83	91819.56	17855.17	14895.45	20814.9	7409.073	5891.951	8926.195	37895.43	30402.29	45388.56	364751.7	240111.9	489391.6	31284.21	25917.95	36650.48	539477.1	424357.2	654597
2030	14983.88	8964.735	21004.25	76875.13	60422.63	93327.62	18227.54	14798.99	21656.09	7703.928	5942.958	9464.899	39176.75	30354.9	47998.6	377258.8	227823.4	526694.1	32534.61	26127.11	38942.11	553283.2	416347.1	690219.4
2031	14990.69	8045.721	21937.2	76946.39	58925.04	94967.75	18600.16	14656.2	22544.12	8008.504	5971.442	10045.57	40500.29	30191.4	50809.17	390481.5	212974	567989	33848.17	26265.14	41431.21	567561.6	406465.3	728657.9
2032	15018.04	7070.748	22967.7	76910.45	57189.66	96631.25	18971.5	14465.39	23477.61	8319.538	5973.322	10665.75	41861	29900.49	53821.51	404162.7	195103.5	613221.9	35222.46	26320.48	44124.44	582101.3	394356.4	769846.3
2033	15074.5	6038.369	24114.09	76841.37	55287.3	98395.45	19349.35	14233.19	24465.5	8644.418	5953.028	11335.84	43262.32	29478.46	57046.18	418462.3	174039.9	662884.8	36678.03	26299.8	47056.26	596989	380126.7	813851.2

2034	15153.97	4936.23	25376.41	76807.48	53277.1	100337.9	19738.62	13962.31	25514.94	8988.889	5912.669	12065.52	44714.6	28923.55	60505.66	433836.9	149573.6	718110.5	38232.77	26203.52	50262.03	612412.4	363808.8	861016
2035	15250.52	3750.58	26756.99	76793.99	51155.6	102432.4	20134.96	13647.56	26622.35	9347.65	5845.293	12850.86	46215.84	28222.69	64208.99	450380.1	121133.4	779656.7	39885.42	26015.02	53755.82	628285.3	345160.8	911409.8

(6) Incidence, female

Years	Central Europe, Eastern Europe, and Central Asia			High-income			Latin America and Caribbean			North Africa and Middle East			South Asia			Southeast Asia, East Asia, and Oceania			Sub-Saharan Africa			Global		
	pred	lower UI	upper UI	pred	lower UI	upper UI	pred	lower UI	upper UI	pred	lower UI	upper UI	pred	lower UI	upper UI	pred	lower UI	upper UI	pred	lower UI	upper UI	pred	lower UI	upper UI
2022	4760.599	4108.276	5413.321	22412.08	21042.45	23781.71	4787.823	4227.757	5347.889	3785.509	3277.383	4293.636	21191.74	19580.41	22803.07	78446.68	68477.25	88416.11	15872.11	14766.77	16977.46	150995.9	142535.7	159456.1
2023	4840.785	4134.043	5547.975	22462.71	20926.3	23999.11	4897.891	4300.465	5495.317	3891.073	3349.482	4432.664	21484.48	19656.48	23312.47	80837.57	69355.25	92319.9	16369.59	15152.18	17587.01	154502.7	144285.4	164720
2024	4926.331	4136.579	5716.579	22530.56	20736.52	24324.61	5012.749	4360.516	5664.982	4002.538	3413.03	4592.045	21788.74	19624.86	23952.61	83384.32	69500.77	97267.87	16895.36	15510.1	18280.62	158202.7	145248.9	171156.4
2025	5016.509	4112.315	5921.252	22602.65	20467.84	24737.45	5130.378	4404.237	5856.519	4117.994	3464.36	4771.629	22108.5	19496.56	24720.45	86023.94	68829.11	103218.8	17443.04	15830.3	19055.77	161959.4	145375	178543.7
2026	5110.908	4059.857	6162.573	22672.79	20124.62	25220.96	5249.361	4429.568	6069.153	4236.172	3501.075	4971.269	22435.43	19273.7	25597.15	88719.86	67337.19	110102.5	18005.61	16105.04	19906.19	165767.5	144736	186799
2027	5209.586	3979.552	6440.311	22732.57	19708.54	25756.59	5368.412	4435.348	6301.476	4355.686	3521.447	5189.925	22766.5	18960.11	26572.9	91417.31	65013.88	117820.7	18577.93	16329.98	20825.88	169594.8	143347.2	195842.3
2028	5314.045	3873.952	6754.926	22795.61	19241.11	26350.11	5490.305	4424.392	6556.218	4479.854	3528.492	5431.216	23104.76	18566.44	27643.08	94217.02	61954.37	126479.7	19171.88	16517.14	21826.62	173576.6	141386.8	205766.4
2029	5424.793	3743.936	7106.557	22870.32	18734.99	27005.64	5617.201	4398.69	6835.711	4611.075	3523.998	5698.152	23454.14	18100.4	28807.88	97228.43	58206.14	136250.7	19797.81	16675.37	22920.25	177762.4	138906.5	216618.2
2030	5540.925	3588.292	7494.617	22946.99	18184.95	27709.02	5747.137	4356.556	7137.718	4747.032	3505.578	5988.486	23816.81	17565.6	30068.02	100426.1	53689.23	147163	20450.32	16798.43	24102.21	182056.9	135814.9	228299
2031	5661.621	3404.983	7919.511	23021.7	17589.38	28454.01	5878.672	4296.625	7460.718	4886.099	3471.316	6300.883	24189.43	16958.2	31420.65	103801.6	48312.79	159290.4	21123.75	16879.21	25368.3	186488.3	132095.5	240881.1
2032	5786.571	3191.907	8382.727	23085.72	16942.8	29228.64	6010.514	4217.659	7803.368	5026.534	3419.36	6633.707	24573.16	16275.52	32870.81	107313.1	41952.56	172673.6	21813.98	16911.84	26716.13	191037.6	127682.6	254392.5
2033	5916.449	2948.28	8886.764	23149.31	16255.7	30042.92	6145.296	4121.387	8169.245	5171.74	3351.862	6991.618	24970.22	15517.85	34422.58	111087.8	34540.3	187635.4	22533.67	16904.22	28163.12	195817.4	122634.9	268999.9
2034	6051.872	2671.769	9435.005	23219.38	15533.81	30904.95	6285.119	4008.633	8561.815	5324.368	3270.122	7379.1	25381.36	14682.46	36080.26	115261.1	25939.19	204587.1	23292.62	16860	29725.23	200873.6	116930.7	284816.5
2035	6192.91	2358.847	10031.06	23287.63	14771.28	31804.22	6428.058	3876.968	8979.549	5482.077	3170.947	7794.389	25805.09	13763.96	37846.21	119819.9	15898.58	223753.9	24084.51	16769.53	31399.49	206122.1	110449.3	301794.9

(7) Death, both

Years	Central Europe, Eastern Europe, and Central Asia			High-income			Latin America and Caribbean			North Africa and Middle East			South Asia			Southeast Asia, East Asia, and Oceania			Sub-Saharan Africa			Global		
	pred	lower UI	upper UI	pred	lower UI	upper UI	pred	lower UI	upper UI	pred	lower UI	upper UI	pred	lower UI	upper UI	pred	lower UI	upper UI	pred	lower UI	upper UI	pred	lower UI	upper UI
2022	19928.54	18229.83	21627.69	80976.66	77193.53	84759.8	20921.88	19243.86	22599.9	9702.514	8556.303	10848.72	52566.12	48368.17	56764.07	339432.1	302410.6	376453.5	41532.7	38749.44	44315.95	559003.7	527482.1	590525.2
2023	19965.97	17944.8	21987.66	81353.06	76989.12	85717.04	21393.87	19562.32	23225.43	10014.26	8783.717	11244.81	53864.24	49142.17	58586.3	350353.5	306925.6	393781.5	42945.79	39808.98	46082.61	573386.1	534701.1	612071
2024	20016.42	17515.47	22518.05	81787.61	76538.81	87036.51	21883.75	19822.07	23945.42	10349.25	8997.004	11701.49	55228.35	49709.45	60747.25	361869.8	308451.9	415287.7	44442.1	40807.7	48076.5	588331.1	538559.2	638103
2025	20071.52	16959.95	23183.95	82226.69	75824.66	88628.88	22381.01	20009.25	24752.77	10699.8	9184.107	12215.49	56651.07	50061.17	63240.98	373887.5	306843.1	440931.8	46010.12	41703.46	50316.77	603709.9	539261.8	668158.1
2026	20126	16291.67	23961.41	82614.63	74826.97	90402.5	22878.45	20117.58	25639.32	11060.89	9337.082	12784.69	58113.4	50188.82	66037.98	386188.9	302012.4	470365.4	47633.16	42472.36	52793.96	619372.1	536928	701816.3
2027	20192.75	15531.56	24855.26	82878.54	73506.83	92250.54	23371.74	20145.84	26597.64	11427.96	9450.336	13405.59	59604.15	50091.4	69116.89	398326.2	293715.5	502936.8	49298.77	43102.56	55494.98	634846.2	531261.2	738431.2
2028	20285.55	14702.11	25870.57	83115.52	71991.92	94239.48	23874.53	20110.59	27638.47	11813.11	9534.635	14091.58	61143.79	49798.25	72489.32	410500.1	282256.8	538743.4	51043.22	43629.08	58457.36	650359.9	522779.1	777940.7
2029	20400.2	13803.94	26998.38	83400.12	70368.23	96432.43	24395.69	20021.54	28769.83	12225.17	9596.397	14853.94	62752.24	49329.94	76174.53	423331.3	268031.7	578630.8	52897.41	44076.15	61718.67	666370.8	511936.7	820804.9
2030	20528.7	12830.29	28229.42	83691.12	68608.07	98774.68	24925.67	19870.83	29980.52	12655.85	9626.722	15684.99	64423.5	48679.18	80167.82	436951.2	250899.4	623003	54849.24	44425.86	65272.61	682928	498697.9	867158
2031	20667.97	11774.23	29564.59	83945.15	66676.81	101214.1	25457.49	19652.02	31262.97	13099.4	9618.592	16580.22	66150.84	47832.66	84469.03	451338.2	230499	672177.4	56885.69	44656.8	69114.59	700112.2	482912.7	917311.8
2032	20827.23	10633.8	31024.83	84092.15	64520.8	103664.2	25987.25	19361.34	32613.19	13550.18	9565.603	17534.76	67927.99	46774.09	89081.89	466128.6	206245.9	726011.4	58997.87	44750.07	73245.67	717541	464048.3	971033.7
2033	21018.54	9409.145	32633.72	84211.91	62220.87	106203.8	26527.97	19009.12	34047.01	14020.86	9476.351	18565.88	69765.63	45502.48	94028.78	481658.8	177938.7	785379.8	61224.26	44724.72	77723.8	735451.4	442261.2	1028642
2034	21239.65	8088.199	34398.77	84368.21	59831.5	108905.8	27088.37	18599.59	35577.49	14521.08	9354.264	19689.45	71679.77	44014.73	99344.8	498575.9	145251.6	851916.2	63596.52	44587.62	82605.41	754221.4	417615.1	1090828
2035	21483.67	6653.791	36323.58	84528.02	57327.25	111729.8	27659.92	18123.31	37197.05	15041.84	9188.242	20898.17	73663.56	42288.22	105038.9	516940.8	107358.5	926563.8	66103.71	44309.75	87897.66	773807.7	389781.1	1157834

(8) Death, male

Years	Central Europe, Eastern Europe, and Central Asia			High-income			Latin America and Caribbean			North Africa and Middle East			South Asia			Southeast Asia, East Asia, and Oceania			Sub-Saharan Africa			Global		
	pred	lower UI	upper UI	pred	lower UI	upper UI	pred	lower UI	upper UI	pred	lower UI	upper UI	pred	lower UI	upper UI	pred	lower UI	upper UI	pred	lower UI	upper UI	pred	lower UI	upper UI
2022	14981.29	13853.8	16108.77	62007.02	59436.07	64577.97	15860.07	14757.88	16962.27	5849.03	5216.344	6481.717	30917.96	28375.98	33459.94	268525.7	240901.5	296149.8	24871.08	23225.77	26516.38	417437.4	393201.2	441673.6
2023	14939.19	13553.09	16325.31	62333.19	59320.79	65345.59	16219.25	15003.16	17435.33	6058.316	5374.682	6741.95	31946.23	29110.65	34781.8	277567.9	244969.9	310165.9	25768.88	23885.94	27651.83	428870.1	399326.6	458413.6
2024	14902.04	13134.81	16669.41	62695.32	59012.8	66377.85	16590.11	15202.55	17977.66	6284.482	5526.934	7042.031	33025.08	29751.44	36298.72	287065.5	246725.8	327405.1	26717.58	24511.16	28924	440654.1	402895.5	478412.8

2025	14863.25	12618.74	17108.02	63056.31	58505.06	67607.57	16965.09	15346.76	18583.41	6522.204	5665.066	7379.343	34145.08	30278.59	38011.57	296998.4	246123.6	347873.3	27714.55	25071.98	30357.12	452801.8	404118.9	501484.6
2026	14818.72	12018.59	17619.25	63369.38	57778.41	68960.35	17338.97	15432.04	19245.89	6767.981	5783.763	7752.2	35297.89	30681.97	39913.8	307192.6	243089.3	371295.9	28752.12	25554.04	31950.2	465191.6	403049.3	527333.9
2027	14781.67	11354.64	18209.27	63569.78	56793.58	70345.98	17709.09	15458.54	19959.63	7018.837	5879.316	8158.357	36477.32	30957.28	41997.37	317251.1	237383	397119.3	29824.4	25950.93	33697.87	477398	399387	555409.1
2028	14762.99	10645.52	18881.2	63738.42	55656.01	71820.83	18085.28	15438.98	20731.59	7282.916	5958.858	8606.974	37697.99	31120.25	44275.74	327256.6	229198.9	425314.3	30952.66	26282.65	35622.67	489492.4	393461.5	585523.3
2029	14757.09	9890.66	19624.46	63939.64	54438.64	73440.63	18473.52	15380.42	21566.62	7566.254	6026.651	9105.857	38972.65	31181.08	46764.23	337720.3	218883.8	456556.8	32155.02	26562.44	37747.6	501861.3	385664.8	618057.8
2030	14757.67	9085.839	20430.68	64143.68	53121.31	75166.06	18866.9	15277.29	22456.51	7863.284	6076.544	9650.024	40293.99	31129.83	49458.15	348818.3	206383	491253.7	33426.46	26780.18	40072.74	514648.3	376055.6	653240.9
2031	14763.27	8227.391	21300.68	64313.12	51672.98	76953.25	19260.32	15124.91	23395.74	8170.171	6103.689	10236.65	41659.69	30958.1	52361.27	360553.5	191426.5	529680.5	34761.05	26922.88	42599.22	527932.4	364522.8	691342.1
2032	14784.03	7316.227	22254.16	64385.93	50045.11	78726.75	19651.51	14920.98	24382.04	8483.281	6103.696	10862.87	43063.42	30653.4	55473.45	372606.9	173544.2	571669.5	36155.1	26978.41	45331.79	541363.8	350611.3	732116.3
2033	14830.68	6353.86	23310.72	64430.54	48307.58	80553.49	20049.99	14673.47	25426.52	8811.301	6082.188	11540.62	44512.15	30214.07	58810.24	385174.2	152595.9	617752.6	37631.75	26955.76	48307.74	555050.3	334417.2	775683.4
2034	14899.44	5330.656	24472.48	64503.12	46509.03	82497.21	20461.79	14385.72	26537.87	9160.787	6041.404	12280.77	46019.17	29639.45	62398.88	398769.9	128399.3	669151.6	39210.92	26856.87	51564.97	569304.9	316022.9	822586.8
2035	14983.56	4233.912	25738.77	64580.59	44631.42	84529.76	20880.93	14051.18	27710.68	9525.51	5973.753	13078.32	47580.23	28913.93	66246.53	413485.5	100376.9	726620.5	40889.68	26663.9	55115.46	584159.7	295221.6	873097.8

(9) Death, female

Years	Central Europe, Eastern Europe, and Central Asia			High-income			Latin America and Caribbean			North Africa and Middle East			South Asia			Southeast Asia, East Asia, and Oceania			Sub-Saharan Africa			Global		
	pred	lower UI	upper UI	pred	lower UI	upper UI	pred	lower UI	upper UI	pred	lower UI	upper UI	pred	lower UI	upper UI	pred	lower UI	upper UI	pred	lower UI	upper UI	pred	lower UI	upper UI
2022	4947.251	4376.027	5518.922	18969.64	17757.46	20181.82	5061.807	4485.98	5637.633	3853.484	3339.959	4367.008	21648.16	19992.19	23304.13	70906.4	61509.06	80303.73	16661.62	15523.67	17799.57	141566.3	134280.9	148851.6
2023	5026.779	4391.71	5662.344	19019.87	17668.33	20371.44	5174.623	4559.152	5790.093	3955.948	3409.035	4502.861	21918.01	20031.52	23804.5	72785.6	61955.64	83615.56	17176.91	15923.04	18430.78	144515.9	135374.5	153657.4
2024	5114.376	4380.664	5848.633	19092.29	17526.01	20658.66	5293.639	4619.518	5967.76	4064.764	3470.07	4659.458	22203.27	19958.01	24448.53	74804.33	61726.01	87882.64	17724.52	16296.54	19152.51	147677	135663.7	159690.3
2025	5208.27	4341.209	6075.93	19170.37	17319.59	21021.31	5415.922	4662.488	6169.356	4177.593	3519.041	4836.144	22505.99	19782.57	25229.41	76889.02	60719.54	93058.5	18295.57	16631.48	19959.66	150908.2	135142.9	166673.5
2026	5307.284	4273.075	6342.158	19245.25	17048.57	21442.15	5539.482	4685.537	6393.426	4292.905	3553.319	5032.491	22815.51	19506.85	26124.17	78996.31	58923.1	99069.51	18881.04	16918.33	20843.76	154180.5	133878.8	174482.3
2027	5411.082	4176.918	6645.994	19308.76	16713.25	21904.56	5662.652	4687.295	6638.009	4409.127	3571.02	5247.235	23126.82	19134.13	27119.52	81075.02	56332.54	105817.5	19474.37	17151.64	21797.1	157448.2	131874.3	183022.2
2028	5522.561	4056.6	6989.371	19377.1	16335.91	22418.65	5789.248	4671.613	6906.882	4530.19	3575.777	5484.604	23445.79	18678.01	28213.58	83243.53	53057.96	113429.1	20090.56	17346.43	22834.69	160867.5	129317.6	192417.3
2029	5643.113	3913.277	7373.922	19460.48	15929.59	22991.8	5922.165	4641.121	7203.208	4658.914	3569.746	5748.082	23779.58	18148.86	29410.31	85610.95	49147.92	122074	20742.39	17513.72	23971.07	164509.5	126271.9	202747.1
2030	5771.024	3744.448	7798.733	19547.44	15486.76	23608.62	6058.774	4593.542	7524.005	4792.571	3550.178	6034.964	24129.5	17549.34	30709.67	88132.87	44516.43	131749.3	21422.78	17645.68	25199.87	168279.7	122642.3	213917.1
2031	5904.703	3546.838	8263.904	19632.03	15003.83	24260.83	6197.168	4527.111	7867.226	4929.233	3514.903	6343.563	24491.16	16874.56	32107.75	90784.68	39072.43	142496.9	22124.64	17733.92	26515.36	172179.8	118389.9	225969.7
2032	6043.196	3317.57	8770.676	19706.22	14475.69	24937.44	6335.741	4440.36	8231.153	5066.902	3461.908	6671.897	24864.57	16120.7	33608.44	93521.77	32701.69	154341.8	22842.78	17771.67	27913.89	176177.2	113437	238917.4
2033	6187.859	3055.286	9323	19781.38	13913.29	25650.26	6477.976	4335.649	8620.486	5209.561	3394.163	7025.258	25253.48	15288.41	35218.55	96484.54	25342.8	167627.2	23592.51	17768.96	29416.06	180401.1	107844	252958.2
2034	6340.202	2757.543	9926.287	19865.08	13322.47	26408.61	6626.573	4213.872	9039.622	5360.294	3312.86	7408.678	25660.6	14375.27	36945.92	99806.07	16852.31	182764.6	24385.6	17730.76	31040.44	184916.5	101592.1	268240.9
2035	6500.112	2419.878	10584.8	19947.43	12695.84	27200.06	6778.987	4072.13	9486.373	5516.328	3214.489	7819.848	26083.33	13374.29	38792.37	103455.3	6981.673	199943.4	25214.03	17645.85	32782.2	189647.9	94559.47	284736.4

Supplementary Table S16｜The PPRD values of global neoplasm ASIR in 204 countries for both sexes in 2020 and 2021.

location_name	APC	Joinpoints	pprdASIR2020	pprdASIR2021	pprdsumASIR
**People's Republic of China**	0.2388	4	0.32%	0.02%	0.17%
**Democratic People's Republic of Korea**	-0.9337*	6	0.05%	0.00%	0.02%
**Taiwan (Province of China)**	-2.0922*	1	-1.74%	1.11%	-0.33%
**Kingdom of Cambodia**	-0.4569*	6	0.07%	0.34%	0.20%
**Republic of Indonesia**	-0.2917*	4	0.16%	0.06%	0.11%
**Lao People's Democratic Republic**	-1.0660*	3	0.34%	0.45%	0.40%
**Malaysia**	0.9304	1	-7.58%	1.40%	-3.08%
**Republic of Maldives**	-1.3791*	3	-3.80%	0.61%	-1.60%
**Republic of the Union of Myanmar**	-0.4996*	6	0.17%	0.13%	0.15%
**Republic of the Philippines**	-0.5270*	1	-0.59%	0.67%	0.04%
**Democratic Socialist Republic of Sri Lanka**	-2.3920*	4	-7.30%	-1.20%	-4.29%
**Kingdom of Thailand**	1.2248*	2	0.77%	-0.74%	0.01%
**Democratic Republic of Timor-Leste**	-0.0737	2	0.05%	-0.28%	-0.12%
**Socialist Republic of Viet Nam**	-0.1077	6	0.53%	-0.54%	-0.01%
**Republic of Fiji**	0.0644	2	-0.10%	-0.93%	-0.52%
**Republic of Kiribati**	-0.4991*	3	0.02%	0.11%	0.06%
**Republic of the Marshall Islands**	-0.5619*	3	0.04%	-0.21%	-0.09%
**Federated States of Micronesia**	0.0881	5	0.19%	0.07%	0.13%
**Independent State of Papua New Guinea**	-0.0243	2	0.66%	-0.60%	0.03%
**Independent State of Samoa**	-0.6156*	4	0.26%	-0.33%	-0.04%
**Solomon Islands**	-0.4893*	3	1.08%	-0.24%	0.42%
**Kingdom of Tonga**	-0.3411*	4	0.22%	0.63%	0.42%

**Republic of Vanuatu**	-0.5288*	3	0.63%	0.55%	0.59%
**Republic of Armenia**	-0.7564	2	1.81%	1.44%	1.63%
**Republic of Azerbaijan**	-3.1912*	3	-0.13%	-0.48%	-0.30%
**Georgia**	-1.8386*	4	1.43%	-0.96%	0.25%
**Republic of Kazakhstan**	-0.7643	4	0.89%	-1.49%	-0.30%
**Kyrgyz Republic**	-3.3817*	0	-0.22%	-0.02%	-0.12%
**Mongolia**	-1.7705*	2	-1.52%	-0.81%	-1.17%
**Republic of Tajikistan**	-2.2645*	4	0.88%	2.53%	1.69%
**Turkmenistan**	1.5589	4	-2.57%	-4.70%	-3.64%
**Republic of Uzbekistan**	-3.5506*	5	1.00%	3.38%	2.17%
**Republic of Albania**	-1.8651*	6	-1.82%	0.01%	-0.91%
**Bosnia and Herzegovina**	-0.2795*	3	2.36%	-4.06%	-0.84%
**Republic of Bulgaria**	0.3684*	2	2.73%	0.34%	1.53%
**Republic of Croatia**	-1.6033*	1	-0.18%	2.09%	0.95%
**Czech Republic**	-0.4759	3	-1.17%	0.26%	-0.46%
**Hungary**	-2.2268*	1	2.31%	2.98%	2.64%
**North Macedonia**	-2.4927*	3	-1.43%	-0.56%	-1.01%
**Montenegro**	-3.1929*	4	3.64%	-2.31%	0.71%
**Republic of Poland**	0.6184	2	-1.61%	-1.89%	-1.75%
**Romania**	0.5090*	3	-0.68%	-0.32%	-0.50%
**Republic of Serbia**	-0.8550*	3	4.50%	-0.35%	2.08%
**Slovak Republic**	-1.0588*	2	-2.12%	-0.68%	-1.40%
**Republic of Slovenia**	-3.3661*	4	-1.93%	1.05%	-0.46%
**Republic of Belarus**	-0.2713*	0	6.67%	6.10%	6.39%

**Republic of Estonia**	1.7548*	4	0.25%	-4.83%	-2.31%
**Republic of Latvia**	0.1006	2	-4.71%	-4.54%	-4.62%
**Republic of Lithuania**	-0.4111	2	4.73%	2.28%	3.51%
**Republic of Moldova**	0.9413*	2	-3.46%	-4.38%	-3.92%
**Russian Federation**	0.3631	3	-1.28%	-3.33%	-2.31%
**Ukraine**	0.8347	2	-8.41%	-9.79%	-9.11%
**Brunei Darussalam**	-3.9894*	4	-1.77%	1.36%	-0.23%
**Japan**	-1.6561*	2	-0.57%	2.07%	0.74%
**Republic of Korea**	-1.0841*	4	-1.23%	2.43%	0.59%
**Republic of Singapore**	-0.7222	1	-4.83%	-9.81%	-7.31%
**Australia**	-0.7449*	1	-3.03%	0.47%	-1.29%
**New Zealand**	-0.8172*	0	-2.77%	-1.24%	-2.01%
**Principality of Andorra**	-10.6487*	5	-6.45%	3.61%	-1.70%
**Republic of Austria**	-4.1293*	2	0.84%	-0.42%	0.22%
**Kingdom of Belgium**	-2.2087*	2	-5.05%	1.88%	-1.62%
**Republic of Cyprus**	-5.4584*	4	-1.44%	0.71%	-0.40%
**Kingdom of Denmark**	-0.1053	1	-1.04%	-2.73%	-1.89%
**Republic of Finland**	0.0457	0	-1.18%	-1.99%	-1.58%
**French Republic**	-2.1548*	4	0.64%	-1.03%	-0.19%
**Federal Republic of Germany**	-0.0033	5	-0.15%	-1.98%	-1.07%
**Hellenic Republic**	1.3113*	2	-0.21%	-1.77%	-0.99%
**Republic of Iceland**	-1.2548*	6	0.74%	-0.84%	-0.05%
**Ireland**	-6.2625*	1	-0.02%	0.08%	0.03%
**State of Israel**	-1.8730*	4	-0.80%	-1.66%	-1.22%

**Republic of Italy**	-2.0437*	0	-1.99%	0.73%	-0.65%
**Grand Duchy of Luxembourg**	-4.3624*	2	-3.22%	1.76%	-0.79%
**Republic of Malta**	-0.9220*	1	-4.21%	2.61%	-0.81%
**Kingdom of the Netherlands**	-2.6938*	3	-1.42%	0.50%	-0.47%
**Kingdom of Norway**	2.2719*	1	-1.88%	-4.54%	-3.22%
**Portuguese Republic**	-1.5378*	2	-0.36%	0.86%	0.24%
**Kingdom of Spain**	-3.0568*	3	-2.57%	1.58%	-0.53%
**Kingdom of Sweden**	0.4211	2	-2.22%	-7.67%	-4.95%
**Swiss Confederation**	-4.5902*	1	-2.74%	3.00%	0.07%
**United Kingdom of Great Britain and Northern Ireland**	-2.2544*	3	-2.10%	0.99%	-0.57%
**Argentine Republic**	-2.3407*	2	1.44%	-4.27%	-1.38%
**Republic of Chile**	-2.9146*	3	-1.44%	1.09%	-0.19%
**Eastern Republic of Uruguay**	-0.9064	3	-3.36%	2.94%	-0.22%
**Canada**	-0.5819*	1	-3.95%	-3.01%	-3.48%
**United States of America**	-0.7523*	1	-1.04%	0.78%	-0.13%
**Antigua and Barbuda**	-0.7145*	2	-1.79%	1.79%	-0.01%
**Commonwealth of the Bahamas**	-0.3892	2	-1.00%	-1.80%	-1.40%
**Barbados**	-0.4628*	0	4.16%	1.38%	2.77%
**Belize**	-2.0725*	4	-2.58%	-1.06%	-1.83%
**Republic of Cuba**	0.4545	2	0.23%	-1.83%	-0.80%
**Commonwealth of Dominica**	0.1772	3	0.04%	-0.39%	-0.17%
**Dominican Republic**	0.1806	6	4.17%	-2.16%	1.00%
**Grenada**	-7.9814*	2	4.13%	11.53%	7.68%
**Republic of Guyana**	0.9041*	3	-3.64%	-5.30%	-4.47%

**Republic of Haiti**	-0.4453*	1	-0.54%	-1.11%	-0.82%
**Jamaica**	0.1719	0	-4.41%	-2.94%	-3.67%
**Saint Lucia**	1.7446*	1	-2.09%	-4.71%	-3.41%
**Saint Vincent and the Grenadines**	-0.2846	5	0.36%	1.30%	0.83%
**Republic of Suriname**	-4.8669*	5	2.33%	-2.71%	-0.13%
**Republic of Trinidad and Tobago**	1.6933*	1	-4.11%	-6.48%	-5.31%
**Plurinational State of Bolivia**	-1.1627*	4	0.06%	-0.02%	0.02%
**Republic of Ecuador**	-2.2387*	3	-2.96%	-1.54%	-2.26%
**Republic of Peru**	3.8404*	1	-1.64%	-7.60%	-4.68%
**Republic of Colombia**	-1.1003	2	5.17%	6.39%	5.78%
**Republic of Costa Rica**	-1.7116*	0	0.51%	4.23%	2.36%
**Republic of El Salvador**	-0.4088	5	1.99%	-0.27%	0.86%
**Republic of Guatemala**	-2.4856*	3	-2.63%	-1.29%	-1.97%
**Republic of Honduras**	-0.8236*	4	-0.23%	-1.22%	-0.72%
**United Mexican States**	-1.0810*	1	1.20%	3.86%	2.52%
**Republic of Nicaragua**	-1.7174*	2	-3.32%	-2.94%	-3.13%
**Republic of Panama**	-1.8206*	2	0.11%	-0.42%	-0.15%
**Bolivarian Republic of Venezuela**	-1.8392*	1	8.42%	10.51%	9.46%
**Federative Republic of Brazil**	-1.3843*	3	0.89%	0.17%	0.53%
**Republic of Paraguay**	0.4446*	1	-1.34%	-2.55%	-1.95%
**People's Democratic Republic of Algeria**	0.4075*	5	0.04%	-0.14%	-0.05%
**Kingdom of Bahrain**	1.2233*	4	-0.24%	0.49%	0.13%
**Arab Republic of Egypt**	-0.9325*	1	-0.68%	0.46%	-0.11%
**Islamic Republic of Iran**	-4.1181*	4	-2.07%	1.22%	-0.46%

**Republic of Iraq**	3.9626*	3	2.30%	-0.95%	0.65%
**Hashemite Kingdom of Jordan**	0.9631	4	0.42%	0.36%	0.39%
**State of Kuwait**	1.9581	5	4.85%	-4.24%	0.26%
**Lebanese Republic**	0.4715	4	-0.26%	-0.33%	-0.29%
**State of Libya**	0.6329*	4	-1.15%	-3.03%	-2.09%
**Kingdom of Morocco**	0.005	6	0.35%	-0.22%	0.07%
**Palestine**	0.6488	4	-0.75%	0.81%	0.03%
**Sultanate of Oman**	-4.2737*	5	1.28%	-1.42%	-0.04%
**State of Qatar**	0.8789	5	2.54%	0.82%	1.67%
**Kingdom of Saudi Arabia**	-0.6533*	6	0.07%	0.12%	0.10%
**Syrian Arab Republic**	0.0893	3	0.53%	-1.15%	-0.31%
**Republic of Tunisia**	-0.1224*	1	-0.76%	-0.57%	-0.67%
**Republic of Turkey**	-0.5551*	4	0.30%	-0.18%	0.06%
**United Arab Emirates**	-14.3118*	3	-8.07%	2.58%	-3.15%
**Republic of Yemen**	0.7850*	4	0.54%	-0.39%	0.07%
**Islamic Republic of Afghanistan**	-0.6902*	5	-0.09%	-0.25%	-0.17%
**People's Republic of Bangladesh**	0.2264	3	-0.35%	-0.77%	-0.56%
**Kingdom of Bhutan**	0.1031*	3	0.49%	-0.38%	0.05%
**Republic of India**	-1.0896	4	0.08%	0.05%	0.06%
**Federal Democratic Republic of Nepal**	0.0918	4	0.08%	-0.44%	-0.18%
**Islamic Republic of Pakistan**	-0.6131*	4	0.17%	0.09%	0.13%
**Republic of Angola**	0.0732	3	0.47%	-0.26%	0.10%
**Central African Republic**	-0.8007*	2	-0.21%	-0.96%	-0.58%
**Republic of the Congo**	-0.6334*	4	0.42%	-0.15%	0.14%

**Democratic Republic of the Congo**	-0.0697	2	-0.20%	-0.35%	-0.28%
**Republic of Equatorial Guinea**	0.3861	5	0.65%	-0.80%	-0.08%
**Gabonese Republic**	-0.126	2	0.22%	-1.21%	-0.50%
**Republic of Burundi**	-0.2459	4	-0.20%	0.09%	-0.06%
**Union of the Comoros**	-0.0562	3	0.26%	0.22%	0.24%
**Republic of Djibouti**	-0.6435*	2	0.02%	0.43%	0.23%
**State of Eritrea**	-0.8979*	5	0.09%	0.01%	0.05%
**Federal Democratic Republic of Ethiopia**	0.6745*	6	0.10%	0.03%	0.07%
**Republic of Kenya**	-0.2499	5	0.15%	-0.29%	-0.07%
**Republic of Madagascar**	-0.4681*	2	0.14%	0.20%	0.17%
**Republic of Malawi**	-0.0921	5	0.09%	-0.71%	-0.31%
**Republic of Mauritius**	6.2205*	4	-3.14%	-5.91%	-4.57%
**Republic of Mozambique**	-1.2920*	4	0.14%	-0.90%	-0.38%
**Republic of Rwanda**	0.3204*	5	0.45%	0.26%	0.35%
**Republic of Seychelles**	-8.1187*	5	-3.92%	2.31%	-0.94%
**Federal Republic of Somalia**	-1.3388*	6	0.14%	-0.09%	0.02%
**United Republic of Tanzania**	-0.2885*	3	0.21%	-0.02%	0.09%
**Republic of Uganda**	-0.3901*	4	0.54%	0.19%	0.37%
**Republic of Zambia**	-1.0101*	3	0.51%	-0.42%	0.05%
**Republic of Botswana**	-0.4858	5	0.45%	-0.28%	0.08%
**Kingdom of Lesotho**	-1.4974*	5	1.32%	-1.05%	0.14%
**Republic of Namibia**	-0.0557	4	-0.03%	-1.66%	-0.84%
**Republic of South Africa**	-2.3715*	3	1.43%	2.93%	2.17%
**Kingdom of Eswatini**	-2.8469*	4	0.57%	-1.40%	-0.40%

**Republic of Zimbabwe**	-0.4401*	5	0.46%	-1.45%	-0.49%
**Republic of Benin**	0.4059*	3	0.71%	0.18%	0.44%
**Burkina Faso**	-0.0168	6	-0.42%	-0.04%	-0.23%
**Republic of Cameroon**	0.2554*	5	-0.06%	-0.74%	-0.40%
**Republic of Cabo Verde**	-2.6433	4	-0.90%	0.56%	-0.18%
**Republic of Chad**	1.3457*	5	0.10%	-0.51%	-0.21%
**Republic of C 么 te d'Ivoire**	-1.6395*	4	0.28%	0.78%	0.53%
**Republic of the Gambia**	1.6738*	2	-1.65%	-3.44%	-2.55%
**Republic of Ghana**	-0.7937*	5	0.05%	0.02%	0.03%
**Republic of Guinea**	-1.1849*	4	0.04%	0.19%	0.12%
**Republic of Guinea-Bissau**	-0.3948	6	0.25%	-0.18%	0.03%
**Republic of Liberia**	1.2793*	3	0.43%	0.38%	0.40%
**Republic of Mali**	-0.6003*	6	-0.30%	-0.57%	-0.43%
**Islamic Republic of Mauritania**	3.2760*	5	-0.21%	-0.35%	-0.28%
**Republic of the Niger**	0.7166*	4	0.03%	-0.39%	-0.18%
**Federal Republic of Nigeria**	0	6	-0.31%	-0.14%	-0.22%
**Democratic Republic of Sao Tome and Principe**	0.4016	4	0.23%	-0.10%	0.07%
**Republic of Senegal**	2.2930*	2	-0.44%	-2.48%	-1.47%
**Republic of Sierra Leone**	0.9577*	4	-0.19%	-0.64%	-0.42%
**Togolese Republic**	1.1481*	3	-0.35%	-0.82%	-0.59%
**American Samoa**	-0.7068*	3	6.15%	0.95%	3.56%
**Bermuda**	-0.7883*	3	-4.31%	1.84%	-1.25%
**Cook Islands**	-0.7403*	4	0.08%	0.25%	0.16%
**Greenland**	-1.2131*	0	4.28%	-4.10%	0.12%

**Guam**	-7.5797*	3	-5.28%	2.90%	-1.35%
**Principality of Monaco**	-0.8057*	5	-0.39%	0.20%	-0.10%
**Republic of Nauru**	-0.2866*	6	0.23%	0.15%	0.19%
**Republic of Niue**	0.5802*	6	-0.01%	-0.07%	-0.04%
**Northern Mariana Islands**	-0.5035*	3	-0.82%	0.22%	-0.30%
**Republic of Palau**	-0.7633*	1	-0.70%	0.57%	-0.07%
**Puerto Rico**	-5.8336*	3	1.84%	8.04%	4.84%
**Saint Kitts and Nevis**	-0.3409	4	2.15%	2.87%	2.51%
**Republic of San Marino**	-23.6280*	1	-11.81%	8.93%	-2.86%
**Tokelau**	0.3486*	5	0.06%	-0.10%	-0.02%
**Tuvalu**	-0.142	4	0.22%	0.02%	0.12%
**United States Virgin Islands**	-7.8895*	4	-3.73%	1.94%	-1.01%
**Republic of South Sudan**	1.0314*	3	0.12%	-0.75%	-0.32%
**Republic of Sudan**	-0.2356*	5	0.14%	-0.06%	0.04%

Supplementary Table S17｜The PPRD values of global neoplasm ASDR in 204 countries for both sexes in 2020 and 2021.

location_name	APC	Joinpoints	pprdASDR2020	pprdASDR2021	pprdsumASDR
**People's Republic of China**	-0.7432*	5	0.56%	0.86%	0.71%
**Democratic People's Republic of Korea**	-1.0107*	6	0.10%	-0.08%	0.01%
**Taiwan (Province of China)**	-2.3353*	1	-1.32%	1.84%	0.24%
**Kingdom of Cambodia**	-0.5175*	6	0.20%	0.04%	0.12%
**Republic of Indonesia**	-0.3745*	4	0.39%	0.22%	0.30%
**Lao People's Democratic Republic**	-1.0651*	4	0.23%	0.22%	0.23%
**Malaysia**	-0.2800*	0	-4.43%	6.55%	1.06%
**Republic of Maldives**	-1.5375*	3	-4.03%	0.88%	-1.60%
**Republic of the Union of Myanmar**	-0.3838	6	-0.06%	-0.20%	-0.13%
**Republic of the Philippines**	-0.6611*	1	-0.30%	0.79%	0.24%
**Democratic Socialist Republic of Sri Lanka**	-2.6352*	4	-8.05%	-1.65%	-4.89%
**Kingdom of Thailand**	0.9130*	2	0.85%	-0.30%	0.27%
**Democratic Republic of Timor-Leste**	-0.0739	2	-0.04%	-0.41%	-0.23%
**Socialist Republic of Viet Nam**	-0.3290*	6	0.37%	-0.51%	-0.07%
**Republic of Fiji**	0.1153	3	-0.42%	-1.36%	-0.89%
**Republic of Kiribati**	-0.4940*	4	0.17%	0.04%	0.10%
**Republic of the Marshall Islands**	-0.5748*	3	-0.16%	-0.10%	-0.13%
**Federated States of Micronesia**	0.085	5	0.14%	-0.02%	0.06%
**Independent State of Papua New Guinea**	0.064	2	0.45%	-0.82%	-0.19%
**Independent State of Samoa**	-0.7118*	4	0.35%	-0.32%	0.01%
**Solomon Islands**	-0.6021*	4	0.84%	-0.05%	0.39%
**Kingdom of Tonga**	-0.3814*	4	0.46%	0.74%	0.60%

**Republic of Vanuatu**	-0.4841*	4	0.18%	0.01%	0.10%
**Republic of Armenia**	-0.9215	2	2.87%	2.41%	2.64%
**Republic of Azerbaijan**	-3.3071*	3	0.01%	-0.57%	-0.27%
**Georgia**	-1.7505*	4	1.85%	-0.86%	0.50%
**Republic of Kazakhstan**	-0.5296	3	0.83%	-1.75%	-0.45%
**Kyrgyz Republic**	-3.3192*	0	-0.78%	-0.69%	-0.74%
**Mongolia**	-1.7561*	2	-1.58%	-0.53%	-1.06%
**Republic of Tajikistan**	-2.2367*	4	0.87%	2.40%	1.63%
**Turkmenistan**	1.6727	4	-2.57%	-4.89%	-3.74%
**Republic of Uzbekistan**	-3.6612*	5	0.94%	3.57%	2.23%
**Republic of Albania**	-1.7919*	6	-1.33%	0.34%	-0.50%
**Bosnia and Herzegovina**	0.0017	2	1.54%	-5.21%	-1.84%
**Republic of Bulgaria**	0.2193	2	2.80%	0.98%	1.89%
**Republic of Croatia**	-1.7415*	1	0.08%	1.89%	0.98%
**Czech Republic**	-0.0174	1	-1.36%	-0.92%	-1.14%
**Hungary**	-2.1578*	1	2.19%	2.44%	2.31%
**North Macedonia**	-2.4427*	3	-1.21%	-0.72%	-0.97%
**Montenegro**	1.2151*	2	2.58%	-7.36%	-2.42%
**Republic of Poland**	0.605	2	-1.36%	-1.69%	-1.53%
**Romania**	0.4609*	3	-0.52%	-0.59%	-0.55%
**Republic of Serbia**	-0.8448*	1	3.79%	-0.56%	1.62%
**Slovak Republic**	-1.0892*	2	-1.81%	-0.86%	-1.34%
**Republic of Slovenia**	-3.3079*	4	-1.60%	0.73%	-0.45%
**Republic of Belarus**	-0.6332*	1	6.67%	6.37%	6.52%

**Republic of Estonia**	1.5984*	4	0.06%	-4.65%	-2.31%
**Republic of Latvia**	0.0253	2	-4.75%	-4.90%	-4.82%
**Republic of Lithuania**	-0.4799*	2	4.55%	1.70%	3.13%
**Republic of Moldova**	0.7275*	2	-3.30%	-4.28%	-3.79%
**Russian Federation**	-0.024	3	-0.74%	-2.56%	-1.65%
**Ukraine**	0.6579	2	-8.16%	-9.32%	-8.74%
**Brunei Darussalam**	-3.6538*	4	-2.50%	0.24%	-1.16%
**Japan**	-0.5539	5	-1.03%	0.65%	-0.19%
**Republic of Korea**	-1.3218*	4	-1.41%	2.18%	0.37%
**Republic of Singapore**	-1.624	1	-3.15%	-7.28%	-5.20%
**Australia**	-0.8395*	1	-2.54%	1.35%	-0.60%
**New Zealand**	-1.3055*	0	-0.33%	1.78%	0.72%
**Principality of Andorra**	-10.2755*	5	-6.82%	3.15%	-2.10%
**Republic of Austria**	-3.9316*	2	1.18%	-0.54%	0.34%
**Kingdom of Belgium**	-2.1601*	2	-4.73%	1.09%	-1.85%
**Republic of Cyprus**	-5.5928*	3	-1.57%	0.50%	-0.57%
**Kingdom of Denmark**	-1.9599*	6	-0.66%	-0.20%	-0.43%
**Republic of Finland**	-0.1757	1	-1.01%	-1.70%	-1.36%
**French Republic**	-1.9972*	3	0.29%	-1.30%	-0.50%
**Federal Republic of Germany**	-0.0283	5	-0.14%	-1.96%	-1.05%
**Hellenic Republic**	1.3720*	3	-0.46%	-2.12%	-1.29%
**Republic of Iceland**	-1.7516*	6	0.34%	-0.86%	-0.26%
**Ireland**	-7.6704*	3	-0.85%	0.59%	-0.16%
**State of Israel**	-2.1692*	5	-0.27%	-0.99%	-0.63%

**Republic of Italy**	-1.9737*	1	-2.43%	-0.13%	-1.29%
**Grand Duchy of Luxembourg**	-1.7104*	1	-5.88%	-4.45%	-5.17%
**Republic of Malta**	-1.3646*	1	-3.65%	3.45%	-0.12%
**Kingdom of the Netherlands**	-2.6764*	2	-1.20%	1.01%	-0.11%
**Kingdom of Norway**	2.5873*	1	-2.01%	-4.71%	-3.38%
**Portuguese Republic**	-1.7403*	2	0.31%	1.52%	0.91%
**Kingdom of Spain**	-2.9641*	3	-2.21%	1.05%	-0.60%
**Kingdom of Sweden**	0.4054	2	-1.81%	-7.25%	-4.53%
**Swiss Confederation**	-3.6987*	2	-2.34%	1.78%	-0.32%
**United Kingdom of Great Britain and Northern Ireland**	-2.6704*	3	-2.15%	1.13%	-0.53%
**Argentine Republic**	-2.3941*	2	1.18%	-4.35%	-1.55%
**Republic of Chile**	-3.2681*	3	-1.40%	1.10%	-0.17%
**Eastern Republic of Uruguay**	-1.0105	3	-3.48%	2.77%	-0.37%
**Canada**	-0.7589*	1	-3.61%	-2.67%	-3.14%
**United States of America**	-0.7977*	1	-0.84%	1.12%	0.14%
**Antigua and Barbuda**	-0.8217	3	-2.23%	1.44%	-0.40%
**Commonwealth of the Bahamas**	-0.4038	2	-1.09%	-1.97%	-1.53%
**Barbados**	-0.5245*	0	4.33%	1.86%	3.10%
**Belize**	-1.9946*	4	-3.04%	-1.21%	-2.14%
**Republic of Cuba**	0.3001	2	0.19%	-1.71%	-0.76%
**Commonwealth of Dominica**	0.1914	3	-0.05%	-0.46%	-0.26%
**Dominican Republic**	0.1051	6	4.11%	-1.74%	1.19%
**Grenada**	-7.7651*	2	3.81%	10.86%	7.19%
**Republic of Guyana**	0.7814*	3	-2.89%	-4.65%	-3.78%

**Republic of Haiti**	-0.6610*	2	0.14%	-0.28%	-0.07%
**Jamaica**	0.1034	0	-3.78%	-2.29%	-3.04%
**Saint Lucia**	1.7209*	1	-1.45%	-3.95%	-2.71%
**Saint Vincent and the Grenadines**	-0.3151	5	0.63%	1.47%	1.05%
**Republic of Suriname**	-5.0864*	5	2.51%	-2.44%	0.10%
**Republic of Trinidad and Tobago**	1.5641*	1	-3.93%	-6.26%	-5.10%
**Plurinational State of Bolivia**	-1.3927*	4	0.18%	-0.27%	-0.04%
**Republic of Ecuador**	-2.2378*	3	-3.63%	-2.68%	-3.16%
**Republic of Peru**	3.4979*	1	-1.27%	-7.32%	-4.34%
**Republic of Colombia**	-1.2064	2	4.77%	6.43%	5.59%
**Republic of Costa Rica**	-1.9185*	1	0.94%	4.59%	2.75%
**Republic of El Salvador**	-0.6236	5	2.53%	0.13%	1.34%
**Republic of Guatemala**	-2.5747*	3	-2.96%	-1.37%	-2.17%
**Republic of Honduras**	-0.8867*	4	0.17%	-1.01%	-0.41%
**United Mexican States**	-1.2175*	1	0.83%	3.39%	2.10%
**Republic of Nicaragua**	-1.8339*	2	-2.63%	-3.69%	-3.15%
**Republic of Panama**	-1.9085*	2	-0.40%	-1.22%	-0.81%
**Bolivarian Republic of Venezuela**	0.7192	2	-4.44%	-5.15%	-4.80%
**Federative Republic of Brazil**	-1.4472*	3	0.82%	-0.13%	0.35%
**Republic of Paraguay**	0.3979*	1	-1.31%	-2.99%	-2.15%
**People's Democratic Republic of Algeria**	0.2099*	5	0.78%	-0.94%	-0.08%
**Kingdom of Bahrain**	1.1091*	6	-0.12%	0.64%	0.27%
**Arab Republic of Egypt**	-0.9867*	1	-0.91%	-0.04%	-0.48%
**Islamic Republic of Iran**	-4.2779*	4	-2.13%	1.24%	-0.48%

**Republic of Iraq**	4.1353*	3	2.55%	-1.59%	0.44%
**Hashemite Kingdom of Jordan**	0.7525	4	0.45%	0.28%	0.37%
**State of Kuwait**	1.9581	5	5.02%	-4.40%	0.26%
**Lebanese Republic**	0.257	4	-0.23%	-0.51%	-0.37%
**State of Libya**	0.5825*	4	-1.05%	-2.70%	-1.88%
**Kingdom of Morocco**	-0.1366	6	0.75%	0.02%	0.38%
**Palestine**	0.498	4	-1.13%	0.48%	-0.32%
**Sultanate of Oman**	-4.5100*	5	2.39%	-1.54%	0.47%
**State of Qatar**	0.8988	5	3.18%	1.34%	2.25%
**Kingdom of Saudi Arabia**	-0.8033*	6	0.00%	-0.12%	-0.06%
**Syrian Arab Republic**	-0.1395	3	-0.16%	-0.94%	-0.55%
**Republic of Tunisia**	-0.2826	2	-0.47%	-0.47%	-0.47%
**Republic of Turkey**	-0.6152*	4	0.48%	-0.39%	0.05%
**United Arab Emirates**	-14.4560*	3	-8.18%	3.02%	-3.02%
**Republic of Yemen**	0.7964*	4	0.54%	-0.57%	-0.02%
**Islamic Republic of Afghanistan**	-0.9357*	6	0.08%	-0.07%	0.00%
**People's Republic of Bangladesh**	0.3268	3	-0.26%	-1.03%	-0.65%
**Kingdom of Bhutan**	0.0985*	3	0.17%	-0.49%	-0.16%
**Republic of India**	-1.3131	3	-0.23%	0.21%	-0.01%
**Federal Democratic Republic of Nepal**	0.096	4	0.08%	-0.47%	-0.19%
**Islamic Republic of Pakistan**	-0.5429*	5	0.19%	-0.10%	0.04%
**Republic of Angola**	0.0789	3	0.43%	-0.34%	0.05%
**Central African Republic**	-0.7840*	2	-0.28%	-0.95%	-0.61%
**Republic of the Congo**	-0.6311*	4	0.38%	-0.23%	0.08%

**Democratic Republic of the Congo**	-0.0852	2	-0.09%	-0.26%	-0.17%
**Republic of Equatorial Guinea**	0.2991	5	0.58%	-0.82%	-0.12%
**Gabonese Republic**	-0.1486	2	0.32%	-1.26%	-0.47%
**Republic of Burundi**	-0.2299	4	-0.19%	0.10%	-0.05%
**Union of the Comoros**	-0.0795	4	0.32%	0.27%	0.30%
**Republic of Djibouti**	-0.6158*	2	0.03%	0.35%	0.19%
**State of Eritrea**	-0.8859*	5	0.11%	0.00%	0.05%
**Federal Democratic Republic of Ethiopia**	0.6410*	6	0.20%	-0.10%	0.05%
**Republic of Kenya**	-0.2536	5	0.16%	-0.35%	-0.09%
**Republic of Madagascar**	-0.4495*	2	0.16%	0.21%	0.19%
**Republic of Malawi**	-0.0723	3	0.07%	-0.81%	-0.37%
**Republic of Mauritius**	5.8567*	4	-3.15%	-5.50%	-4.36%
**Republic of Mozambique**	-1.2676*	4	0.26%	-0.90%	-0.32%
**Republic of Rwanda**	0.3387*	5	0.40%	0.23%	0.31%
**Republic of Seychelles**	-8.2364*	5	-4.77%	2.74%	-1.18%
**Federal Republic of Somalia**	-1.4086*	6	0.18%	-0.09%	0.04%
**United Republic of Tanzania**	-0.2764*	3	0.21%	-0.07%	0.07%
**Republic of Uganda**	-0.3830*	4	0.52%	0.07%	0.30%
**Republic of Zambia**	-0.9848*	3	0.58%	-0.60%	-0.01%
**Republic of Botswana**	-0.5097	5	0.58%	-0.39%	0.10%
**Kingdom of Lesotho**	-1.5094*	5	1.52%	-1.38%	0.08%
**Republic of Namibia**	0.0076	3	-0.07%	-1.85%	-0.96%
**Republic of South Africa**	-2.3703*	3	1.10%	2.41%	1.75%
**Kingdom of Eswatini**	-2.8077*	4	0.60%	-2.12%	-0.74%

**Republic of Zimbabwe**	-0.4630*	5	0.51%	-1.84%	-0.66%
**Republic of Benin**	0.3776*	3	0.63%	0.12%	0.37%
**Burkina Faso**	0.016	6	-0.41%	0.00%	-0.20%
**Republic of Cameroon**	0.2556*	5	-0.05%	-0.75%	-0.40%
**Republic of Cabo Verde**	-3.0784	4	-1.09%	0.61%	-0.25%
**Republic of Chad**	1.3393*	5	0.07%	-0.64%	-0.29%
**Republic of C 么 te d'Ivoire**	-1.6484*	4	-0.10%	0.25%	0.08%
**Republic of the Gambia**	1.5892*	2	-1.27%	-3.44%	-2.37%
**Republic of Ghana**	-0.7284*	5	0.19%	-0.12%	0.04%
**Republic of Guinea**	-1.1701*	4	0.09%	0.11%	0.10%
**Republic of Guinea-Bissau**	-0.4035	6	0.22%	-0.10%	0.06%
**Republic of Liberia**	1.2658*	3	0.34%	0.30%	0.32%
**Republic of Mali**	-0.7266*	6	-0.25%	-0.66%	-0.45%
**Islamic Republic of Mauritania**	3.1843*	5	-0.08%	-0.33%	-0.21%
**Republic of the Niger**	0.6871*	4	-0.02%	-0.33%	-0.18%
**Federal Republic of Nigeria**	0.0272	6	-0.24%	-0.14%	-0.19%
**Democratic Republic of Sao Tome and Principe**	0.9037	4	0.55%	-0.51%	0.02%
**Republic of Senegal**	2.3074*	2	-0.46%	-2.48%	-1.48%
**Republic of Sierra Leone**	0.9086*	4	-0.18%	-0.61%	-0.40%
**Togolese Republic**	1.1270*	3	-0.36%	-0.74%	-0.55%
**American Samoa**	-0.7669*	3	5.60%	1.11%	3.37%
**Bermuda**	-0.9603*	3	-3.98%	1.91%	-1.04%
**Cook Islands**	-0.9709*	4	-0.11%	0.19%	0.04%
**Greenland**	-1.3029*	0	4.23%	-4.16%	0.06%

**Guam**	-6.3119*	4	-4.95%	2.14%	-1.52%
**Principality of Monaco**	-0.9492*	5	-0.44%	0.20%	-0.12%
**Republic of Nauru**	-0.3827*	6	0.18%	-0.05%	0.06%
**Republic of Niue**	0.5015*	6	0.30%	0.13%	0.22%
**Northern Mariana Islands**	1.0123	6	-1.76%	0.16%	-0.80%
**Republic of Palau**	-0.9304*	2	-0.63%	0.92%	0.14%
**Puerto Rico**	-5.9585*	3	1.54%	7.92%	4.63%
**Saint Kitts and Nevis**	-0.4042	4	2.11%	2.72%	2.42%
**Republic of San Marino**	-25.1786*	2	-12.35%	10.04%	-2.78%
**Tokelau**	0.1978	5	0.09%	-0.12%	-0.01%
**Tuvalu**	-0.2848*	4	0.27%	-0.02%	0.13%
**United States Virgin Islands**	-7.8905*	4	-4.16%	2.28%	-1.08%
**Republic of South Sudan**	0.9663*	4	0.12%	-0.74%	-0.31%
**Republic of Sudan**	-0.2537*	5	0.08%	-0.20%	-0.06%

Supplementary Table S18 | The PPRD values of global neoplasm ASDALYR in 204 countries for both sexes in 2020 and 2021.

location_name	APC	Joinpoints	pprdASDALYR2020	pprdASDALYR2021	pprdsumASDALYR
People's Republic of China	-0.2421	4	0.18%	0.06%	0.12%
Democratic People's Republic of Korea	-0.9667*	5	0.04%	0.04%	0.04%
Taiwan (Province of China)	-2.4730*	1	-1.28%	1.63%	0.16%
Kingdom of Cambodia	-0.6601*	6	0.16%	0.28%	0.22%
Republic of Indonesia	-0.5663*	4	0.27%	0.04%	0.16%
Lao People's Democratic Republic	-1.2074*	3	0.22%	0.23%	0.23%
Malaysia	0.6206*	2	-5.31%	1.72%	-1.79%
Republic of Maldives	-1.8835*	3	-3.81%	1.78%	-1.04%
Republic of the Union of Myanmar	-0.7599*	6	0.04%	0.17%	0.10%
Republic of the Philippines	-0.6498*	1	-0.71%	0.71%	0.00%
Democratic Socialist Republic of Sri Lanka	-2.8558*	4	-5.48%	0.27%	-2.65%
Kingdom of Thailand	0.9406*	2	0.63%	-1.07%	-0.22%
Democratic Republic of Timor-Leste	-0.0455	4	0.08%	-0.07%	0.00%
Socialist Republic of Viet Nam	-0.4015*	6	0.40%	-0.38%	0.01%
Republic of Fiji	-0.1513	2	0.38%	-0.18%	0.10%
Republic of Kiribati	-0.5206*	3	0.05%	0.11%	0.08%
Republic of the Marshall Islands	-0.6597*	3	0.04%	-0.02%	0.01%
Federated States of Micronesia	-0.0289	5	0.05%	0.00%	0.03%
Independent State of Papua New Guinea	0.1795	3	0.52%	-0.94%	-0.21%
Independent State of Samoa	-0.5136*	4	-0.05%	-0.16%	-0.11%
Solomon Islands	-0.5436*	3	0.92%	-0.35%	0.29%
Kingdom of Tonga	-0.4119*	4	0.41%	0.61%	0.51%

**Republic of Vanuatu**	-0.5156*	3	0.29%	0.33%	0.31%
**Republic of Armenia**	-0.2191	4	0.75%	-0.34%	0.20%
**Republic of Azerbaijan**	-3.8167*	3	-0.40%	0.72%	0.15%
**Georgia**	-2.1574*	4	0.09%	-1.43%	-0.66%
**Republic of Kazakhstan**	-0.6782	4	0.26%	-1.65%	-0.69%
**Kyrgyz Republic**	-3.7247*	0	0.96%	2.31%	1.62%
**Mongolia**	-1.9362*	2	-1.53%	-1.59%	-1.56%
**Republic of Tajikistan**	-2.1932*	4	0.59%	2.27%	1.43%
**Turkmenistan**	1.054	4	-2.12%	-3.81%	-2.97%
**Republic of Uzbekistan**	-3.2190*	5	0.63%	2.89%	1.74%
**Republic of Albania**	-2.3581*	5	-2.25%	-0.25%	-1.26%
**Bosnia and Herzegovina**	-0.5285*	3	2.21%	-4.27%	-1.02%
**Republic of Bulgaria**	0.3832*	2	2.12%	-0.32%	0.90%
**Republic of Croatia**	-1.9825*	1	-0.01%	2.44%	1.21%
**Czech Republic**	-1.0328*	3	-1.63%	0.98%	-0.33%
**Hungary**	-2.7851*	1	1.64%	3.70%	2.66%
**North Macedonia**	-2.7303*	3	-1.14%	-0.02%	-0.59%
**Montenegro**	-3.6730*	4	3.24%	-1.91%	0.71%
**Republic of Poland**	0.4782	2	-2.07%	-2.43%	-2.25%
**Romania**	0.2022	3	-0.67%	-0.08%	-0.38%
**Republic of Serbia**	-1.1814*	3	4.80%	0.28%	2.55%
**Slovak Republic**	-1.4292*	2	-2.14%	0.08%	-1.04%
**Republic of Slovenia**	-4.0882*	4	-2.81%	1.77%	-0.57%
**Republic of Belarus**	-0.6155*	1	6.48%	6.08%	6.28%

**Republic of Estonia**	1.0282	4	0.97%	-4.20%	-1.63%
**Republic of Latvia**	-0.3974*	0	-4.05%	-2.45%	-3.25%
**Republic of Lithuania**	-0.2824	5	2.55%	-0.20%	1.18%
**Republic of Moldova**	0.7551*	2	-5.33%	-6.21%	-5.77%
**Russian Federation**	-0.0104	3	-0.58%	-2.48%	-1.53%
**Ukraine**	0.7419	2	-7.91%	-8.74%	-8.33%
**Brunei Darussalam**	-3.1847*	3	-1.00%	0.63%	-0.20%
**Japan**	-1.0063	5	-0.87%	0.60%	-0.14%
**Republic of Korea**	-1.3298*	4	-1.62%	1.62%	-0.01%
**Republic of Singapore**	-1.9799	1	-2.84%	-7.34%	-5.07%
**Australia**	-0.9844*	1	-2.27%	1.07%	-0.61%
**New Zealand**	-1.4855*	0	-0.87%	0.97%	0.05%
**Principality of Andorra**	-10.9209*	5	-6.75%	3.50%	-1.92%
**Republic of Austria**	-4.8996*	2	0.19%	-0.11%	0.05%
**Kingdom of Belgium**	-3.0669*	3	-5.64%	3.62%	-1.08%
**Republic of Cyprus**	-5.6980*	4	-0.71%	0.33%	-0.20%
**Kingdom of Denmark**	-0.2863	2	-1.51%	-3.51%	-2.51%
**Republic of Finland**	-0.4103	1	-1.23%	-1.72%	-1.48%
**French Republic**	-2.3642*	6	0.25%	-1.11%	-0.42%
**Federal Republic of Germany**	-0.0963	5	-0.29%	-1.88%	-1.09%
**Hellenic Republic**	1.3616*	2	-0.49%	-2.31%	-1.40%
**Republic of Iceland**	-0.699	6	1.47%	-1.40%	0.04%
**Ireland**	-6.8287*	3	-0.61%	0.34%	-0.15%
**State of Israel**	-2.0323*	4	-1.56%	-2.52%	-2.04%

**Republic of Italy**	-3.2813*	2	-2.99%	2.35%	-0.37%
**Grand Duchy of Luxembourg**	-2.1900*	1	-6.02%	-2.70%	-4.38%
**Republic of Malta**	-1.1478*	1	-4.22%	2.39%	-0.93%
**Kingdom of the Netherlands**	-2.8947*	2	-1.74%	0.61%	-0.58%
**Kingdom of Norway**	1.8605*	1	-1.58%	-4.26%	-2.93%
**Portuguese Republic**	-1.9546*	2	-0.38%	1.29%	0.45%
**Kingdom of Spain**	-3.3693*	3	-3.33%	1.82%	-0.80%
**Kingdom of Sweden**	0.0187	2	-2.61%	-8.34%	-5.48%
**Swiss Confederation**	-4.6482*	1	-4.16%	3.14%	-0.60%
**United Kingdom of Great Britain and Northern Ireland**	-3.3277*	3	-2.54%	1.61%	-0.50%
**Argentine Republic**	-2.7355*	3	2.23%	-3.11%	-0.40%
**Republic of Chile**	-2.4047*	2	-2.08%	0.17%	-0.97%
**Eastern Republic of Uruguay**	-1.1758	3	-2.91%	3.31%	0.18%
**Canada**	-0.7290*	1	-3.56%	-2.72%	-3.14%
**United States of America**	-1.0083*	1	-0.21%	1.37%	0.57%
**Antigua and Barbuda**	-0.7492*	2	-0.61%	1.02%	0.20%
**Commonwealth of the Bahamas**	-0.3523	3	-0.69%	-1.44%	-1.06%
**Barbados**	-0.5300*	0	3.91%	0.08%	2.00%
**Belize**	-2.5293*	4	-2.19%	-0.05%	-1.14%
**Republic of Cuba**	0.2988	2	0.34%	-1.68%	-0.67%
**Commonwealth of Dominica**	0.0478	3	-0.04%	-0.40%	-0.22%
**Dominican Republic**	0.076	6	3.05%	-2.92%	0.07%
**Grenada**	-8.0756*	1	3.77%	11.41%	7.43%
**Republic of Guyana**	1.0531*	3	-4.46%	-6.15%	-5.31%

**Republic of Haiti**	-0.4743*	1	-0.44%	-0.88%	-0.66%
**Jamaica**	-1.563	3	5.59%	9.35%	7.46%
**Saint Lucia**	1.7323*	1	-3.63%	-6.27%	-4.96%
**Saint Vincent and the Grenadines**	-0.5403*	5	0.25%	1.28%	0.76%
**Republic of Suriname**	-4.5382*	5	2.47%	-2.37%	0.11%
**Republic of Trinidad and Tobago**	2.2126*	1	-5.64%	-8.62%	-7.14%
**Plurinational State of Bolivia**	-0.3758*	3	-0.02%	-0.75%	-0.38%
**Republic of Ecuador**	-2.6406*	3	-1.92%	0.01%	-0.97%
**Republic of Peru**	3.6156*	1	-1.14%	-6.68%	-3.96%
**Republic of Colombia**	-1.2729	2	5.13%	6.87%	5.99%
**Republic of Costa Rica**	-1.9069*	0	1.99%	8.01%	4.97%
**Republic of El Salvador**	-0.4623	5	1.67%	-0.61%	0.53%
**Republic of Guatemala**	-2.4378*	3	-2.58%	-1.22%	-1.91%
**Republic of Honduras**	-0.7798*	4	-0.14%	-0.79%	-0.46%
**United Mexican States**	-0.9941*	1	2.22%	4.15%	3.18%
**Republic of Nicaragua**	-1.6880*	2	-3.58%	-3.42%	-3.50%
**Republic of Panama**	-1.9929*	2	1.16%	-0.16%	0.50%
**Bolivarian Republic of Venezuela**	-1.8823*	1	9.18%	11.42%	10.29%
**Federative Republic of Brazil**	-1.5952*	1	1.01%	1.22%	1.11%
**Republic of Paraguay**	0.3109*	1	-1.28%	-2.19%	-1.74%
**People's Democratic Republic of Algeria**	0.2607*	4	0.28%	-0.24%	0.02%
**Kingdom of Bahrain**	0.6917*	3	0.13%	1.22%	0.68%
**Arab Republic of Egypt**	-1.2471*	1	0.31%	1.73%	1.01%
**Islamic Republic of Iran**	-4.3208*	4	-2.35%	1.37%	-0.53%

**Republic of Iraq**	2.7413*	3	2.03%	-0.76%	0.62%
**Hashemite Kingdom of Jordan**	0.9396*	6	0.07%	0.23%	0.15%
**State of Kuwait**	1.4727	5	4.53%	-3.32%	0.58%
**Lebanese Republic**	0.5037	4	0.13%	-0.18%	-0.03%
**State of Libya**	0.8054*	3	-1.61%	-3.46%	-2.54%
**Kingdom of Morocco**	-0.1131	6	0.30%	-0.22%	0.04%
**Palestine**	0.5193	4	-1.16%	0.57%	-0.30%
**Sultanate of Oman**	-5.6909*	6	-0.70%	0.39%	-0.17%
**State of Qatar**	-0.8986	5	0.78%	1.07%	0.93%
**Kingdom of Saudi Arabia**	-0.8172*	6	0.00%	0.00%	0.00%
**Syrian Arab Republic**	0.5961*	2	-1.41%	-2.70%	-2.06%
**Republic of Tunisia**	-0.2335	3	-1.13%	-0.58%	-0.85%
**Republic of Turkey**	-1.0263*	4	0.38%	0.53%	0.45%
**United Arab Emirates**	-13.5870*	3	-7.74%	2.66%	-2.92%
**Republic of Yemen**	0.8190*	4	0.52%	-0.51%	0.00%
**Islamic Republic of Afghanistan**	-0.8136*	5	0.01%	-0.17%	-0.08%
**People's Republic of Bangladesh**	-0.3048	3	0.03%	-0.34%	-0.15%
**Kingdom of Bhutan**	-0.1789*	3	0.51%	0.07%	0.29%
**Republic of India**	-1.2855*	3	-0.09%	0.05%	-0.02%
**Federal Democratic Republic of Nepal**	-0.1519	4	0.21%	-0.28%	-0.03%
**Islamic Republic of Pakistan**	-0.7118*	4	0.22%	0.07%	0.14%
**Republic of Angola**	-0.1226	3	0.36%	-0.08%	0.14%
**Central African Republic**	-0.8555*	2	-0.15%	-0.86%	-0.51%
**Republic of the Congo**	-0.6993*	5	0.33%	0.00%	0.16%

**Democratic Republic of the Congo**	-0.1263*	2	-0.36%	-0.47%	-0.42%
**Republic of Equatorial Guinea**	0.4477	5	0.52%	-0.72%	-0.10%
**Gabonese Republic**	-0.2881	4	0.00%	-1.28%	-0.64%
**Republic of Burundi**	-0.4567*	4	-0.04%	0.39%	0.17%
**Union of the Comoros**	-0.0923	3	0.17%	0.06%	0.12%
**Republic of Djibouti**	-0.9011*	2	0.09%	0.57%	0.33%
**State of Eritrea**	-1.0158*	5	0.04%	0.17%	0.11%
**Federal Democratic Republic of Ethiopia**	0.6011*	6	0.03%	0.04%	0.03%
**Republic of Kenya**	-0.3962	5	0.07%	-0.35%	-0.14%
**Republic of Madagascar**	-0.5582*	2	0.02%	0.11%	0.06%
**Republic of Malawi**	-0.1948	5	-0.05%	-0.69%	-0.37%
**Republic of Mauritius**	6.7157	5	-2.76%	-7.33%	-5.12%
**Republic of Mozambique**	-2.0668*	5	0.06%	-0.03%	0.01%
**Republic of Rwanda**	0.0234	5	0.50%	0.61%	0.55%
**Republic of Seychelles**	-8.5053*	5	-2.09%	1.22%	-0.51%
**Federal Republic of Somalia**	-0.9397*	5	0.14%	-0.25%	-0.05%
**United Republic of Tanzania**	-0.3963	5	0.10%	-0.04%	0.03%
**Republic of Uganda**	-0.4861*	4	0.32%	0.16%	0.24%
**Republic of Zambia**	-1.1710*	3	0.27%	-0.41%	-0.07%
**Republic of Botswana**	-0.5634	5	0.23%	-0.07%	0.08%
**Kingdom of Lesotho**	-1.4275*	5	0.90%	-0.60%	0.15%
**Republic of Namibia**	-0.1193	4	-0.12%	-1.38%	-0.75%
**Republic of South Africa**	-2.0538*	4	0.35%	1.87%	1.10%
**Kingdom of Eswatini**	-3.0236*	4	0.56%	-0.28%	0.14%

**Republic of Zimbabwe**	-0.4409*	5	0.21%	-0.83%	-0.31%
**Republic of Benin**	0.3952*	3	0.48%	-0.08%	0.20%
**Burkina Faso**	-0.4274*	6	-0.14%	0.37%	0.12%
**Republic of Cameroon**	-0.0024	6	0.07%	-0.25%	-0.09%
**Republic of Cabo Verde**	-2.17	4	-0.88%	0.49%	-0.20%
**Republic of Chad**	1.3542*	4	-0.04%	-0.68%	-0.36%
**Republic of Cote d'Ivoire**	-1.7846*	4	-0.05%	0.60%	0.27%
**Republic of the Gambia**	1.7992*	2	-1.54%	-3.70%	-2.63%
**Republic of Ghana**	-1.1500*	5	0.06%	0.01%	0.03%
**Republic of Guinea**	-1.2846*	4	0.09%	0.01%	0.05%
**Republic of Guinea-Bissau**	-0.3993	6	0.29%	-0.14%	0.08%
**Republic of Liberia**	0.9613*	3	0.95%	1.45%	1.20%
**Republic of Mali**	-0.6152*	6	-0.30%	-0.65%	-0.47%
**Islamic Republic of Mauritania**	3.1973*	5	-0.30%	-0.19%	-0.25%
**Republic of the Niger**	0.4809	4	0.13%	-0.32%	-0.10%
**Federal Republic of Nigeria**	-0.1324	6	-0.19%	0.04%	-0.07%
**Democratic Republic of Sao Tome and Principe**	0.7703	4	0.27%	-0.14%	0.07%
**Republic of Senegal**	2.1863*	2	-0.68%	-2.59%	-1.64%
**Republic of Sierra Leone**	0.9391*	4	-0.23%	-0.59%	-0.41%
**Togolese Republic**	1.0817*	3	-0.52%	-1.04%	-0.78%
**American Samoa**	-0.6872*	3	6.33%	0.74%	3.55%
**Bermuda**	-1.1124*	3	-4.61%	2.44%	-1.10%
**Cook Islands**	-1.0121*	4	0.06%	0.14%	0.10%
**Greenland**	-1.4557*	1	3.57%	-4.30%	-0.34%

**Guam**	-6.8477*	4	-3.89%	2.45%	-0.84%
**Principality of Monaco**	-1.0877*	5	-0.47%	0.35%	-0.06%
**Republic of Nauru**	-0.3074*	6	0.04%	-0.08%	-0.02%
**Republic of Niue**	0.5261*	6	0.25%	-0.07%	0.09%
**Northern Mariana Islands**	1.2129	5	2.42%	-0.81%	0.80%
**Republic of Palau**	-0.1364	5	-0.29%	0.16%	-0.07%
**Puerto Rico**	-5.9484*	3	1.95%	7.40%	4.59%
**Saint Kitts and Nevis**	-0.1265	5	1.47%	2.23%	1.85%
**Republic of San Marino**	-23.5002*	2	-11.33%	8.26%	-2.84%
**Tokelau**	0.4831*	6	0.49%	-0.23%	0.13%
**Tuvalu**	-0.3015*	5	0.09%	0.18%	0.14%
**United States Virgin Islands**	-8.3594*	4	-3.10%	1.70%	-0.80%
**Republic of South Sudan**	1.1162*	3	0.17%	-0.75%	-0.29%
**Republic of Sudan**	-0.4969*	5	0.15%	-0.07%	0.04%
